# Cationic and Neutral Heterometallic Ir-Group 12 Element Polyhydride Compounds: Synthesis, Structure and Reactivity

**DOI:** 10.1021/acs.inorgchem.5c04368

**Published:** 2026-01-01

**Authors:** Amber M. Walsh, Carlos Martín-Fernández, John P. Lowe, Stuart A. Macgregor, Mary F. Mahon, Michael K. Whittlesey

**Affiliations:** † Department of Chemistry, 1555 University of Bath, Bath BA2 7AY, U.K.; ‡ EaStCHEM School of Chemistry, 7486 University of St Andrews, North Haugh, St Andrews KY16 9ST, U.K.

## Abstract

The preparation and reactivity of some Ir–Zn and Ir–Cd heterometallic hydride complexes are described. Treatment of [Ir­(IPr)_2_H_2_]­[BAr^F^_4_] (**1**; IPr = 1,3-bis­(2,6-diisopropylphenyl)­imidazol-2-ylidene; Ar^F^ = 3,5-C_6_H_3_(CF_3_)_2_) with M′R_2_ (M′ = Zn; R = Ph, Me, Et; M′ = Cd, R = Me) and Me_3_SiCH=CH_2_ results in dehydrogenation of an IPr isopropyl substituent, along with R–H elimination, to form square-pyramidal [Ir­(IPr)­(IPr″)­(M′R)­H]­[BAr^F^_4_] (M′R = ZnPh (**4a**); ZnMe (**4b**); ZnEt (**4c**); CdMe (**8**); IPr″ = dehydrogenated IPr) featuring apical M′R ligands. Heating **1** with 2 equiv ZnPh_2_ under H_2_ forms [Ir­(IPr)_2_(ZnPh)_2_H_4_]­[BAr^F^_4_] (**5**) featuring trans ZnPh ligands. Exposure of **4b**-**c** and **8** to H_2_ yields [Ir­(IPr)­(IPr″)­(M′R)­H_3_]­[BAr^F^_4_] (**9b**-**c**, **10**) as intermediates to highly fluxional [Ir­(IPr)_2_(M′R)­(η^2^-H_2_)­H_3_]­[BAr^F^_4_] (**11b**-**c, 12**). Reacting **11b**–**c** with Lewis bases (L) effects [ZnR]^+^ abstraction to give pentahydride Ir­(IPr)_2_H_5_ (**13**); with **11c** and L = IMes (1,3-bis­(2,4,6-trimethylphenyl)­imidazol-2-ylidene), [L_2_ZnEt]­[BAr^F^_4_] was characterized, whereas with L = PMe_3_, both **13** and [Ir­(IPr)_2_(ZnEt)­(PMe_3_)­H_3_]­[BAr^F^_4_] (**14c**) were formed. Reactions of **13** with M′R_2_ similarly proceed with R–H elimination to form Ir­(IPr)_2_(M′R)­H_4_ (**15a**-**c**, **16**). Crystallographic and computational analyses characterize a range of hydride ligands, the nature of which depends subtly on the surrounding coordination environment. The new polyhydride complexes reported here add to the small number of such species featuring N-heterocyclic carbene ligands.

## Graphical abstract

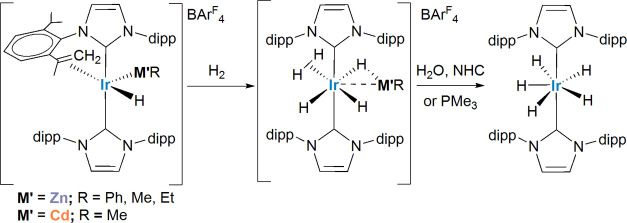

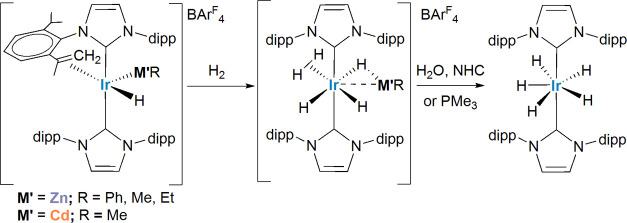

## Introduction

Polyhydride transition metal (TM) compounds containing traditional Lewis-basic ancillary ligands such as phosphines and carbonyls are well-established.^1,2^ Until recently, examples of such TM–H_*x*_ (*x* > 2) species featuring Lewis-acidic (LA) ligands based on s- and p-block metals were known, although not that common.^3–9^ This situation has changed over the past few years due to the renaissance of interest in TM-LA heterometallic chemistry.^10,11^ Scheme 1 shows some recent examples which are notable for their unusual bonding interactions (**I**),^12^ unexpected stoichiometric reactivity (**II**)^13^ and catalytic activity with traditionally challenging substrates (**III**)^14^ respectively.

**Scheme 1:**
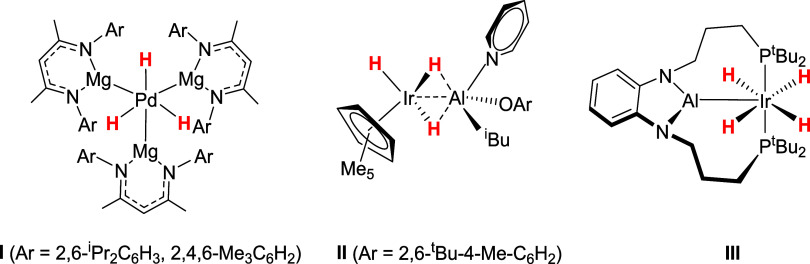
Recent Examples of TM-LA Polyhydride Complexes Notable for Their Structure (**I**), Stoichiometric Reactivity (**II**) and Catalysis (**III**)

We recently reported^15^ the synthesis of [Ir­(IPr)_2_(ZnMe)_2_H_4_]­[BAr^F^_4_] (**3**; IPr = 1,3-bis­(2,6-diisopropylphenyl)­imidazol-2-ylidene; Ar^F^ = 3,5-C_6_H_3_(CF_3_)_2_), which was identified as a ′classical′ tetrahydride species (Scheme 2) on the basis of the hydride *T*_1_ values and the splitting of the IPr carbenic carbon resonance into a ^2^ *J*_CH_-coupled quintet in the ^13^C­{selective-^1^H} NMR spectrum. NMR spectroscopy also revealed that all four hydride ligands in **3** were in exchange, even at −50 °C. In stark contrast, intermolecular H/D exchange of **3** with D_2_ required heating at 40 °C, while elimination of H_2_ to afford the dihydride species [Ir­(IPr)_2_(ZnMe)_2_H_2_]­[BAr^F^_4_] proceeded at 60 °C, but only upon heating solid **3** under constant vacuum for at least a week. Computational studies identified the important role of electrostatic H^δ−^···Zn^δ+^Me interactions in helping to stabilize **3**.

**Scheme 2:**
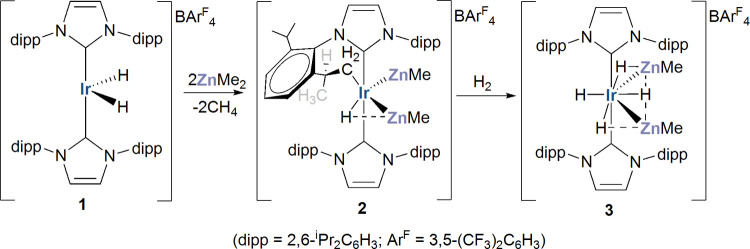
Synthesis of the Heterometallic Tetrahydride Species **3** ^a^

a Agostic interactions in **1** are omitted for clarity.

As shown in Scheme 2, the precursor to **3** is the cyclometalated carbene monohydride salt [Ir­(IPr)­(IPr′)­(ZnMe)_2_H]­[BAr^F^_4_] (**2**), which itself is formed in an alkane elimination reaction following the addition of 2 equiv of ZnMe_2_ to the dihydride precursor **1**.^16^ Herein, we show that subjecting the latter to not only ZnMe_2_, but also ZnEt_2_ and ZnPh_2_, in the presence of an alkene affords a series of dehydrogenated carbene monohydride [Ir­(IPr)­(IPr″)­(ZnR)­H]­[BAr^F^_4_] products (where IPr″ has one dehydrogenated ^i^Pr substituent) which act as precursors to an array of new cationic, as well as neutral, heterometallic Ir–ZnR polyhydride species. Throughout, R–H elimination proves an efficient strategy^17^ for the preparation of Ir–ZnR heterometallic species. As in our previous study,^15^ CdMe_2_ shows analogous reactivity to that seen with ZnMe_2_.

## Results and Discussion

### Reactivity of 1 with ZnPh_2_: Formation of [Ir­(IPr)­(IPr″)­(ZnPh)­H]­[BAr^F^_4_] (**4a**), [Ir­(IPr)_2_(ZnPh)_2_H_4_]­[BAr^F^_4_] (**5**) and [Ir­(IPr)­(IPr″)­H_2_]­[BAr^F^_4_] (**7**)

In contrast to the near instantaneous room temperature reaction of **1** and ZnMe_2_, the reaction with ZnPh_2_ (2 equiv, C_6_H_5_F) proceeded only upon heating (80 °C) to give (over ca. 4 h) a ca. 1:1.4:5.4 ratio of three hydride-containing products, [Ir­(IPr)­(IPr″)­(ZnPh)­H]­[BAr^F^_4_] (**4a**), [Ir­(IPr)_2_(ZnPh)_2_H_4_]­[BAr^F^_4_] (**5**) and [Ir­(IPr)­(IPr″)­H_2_]­[BAr^F^_4_] (**7**). This ratio changed with additional heating (Scheme 3), leaving **4a** as the major species after 15 h.

**Scheme 3:**
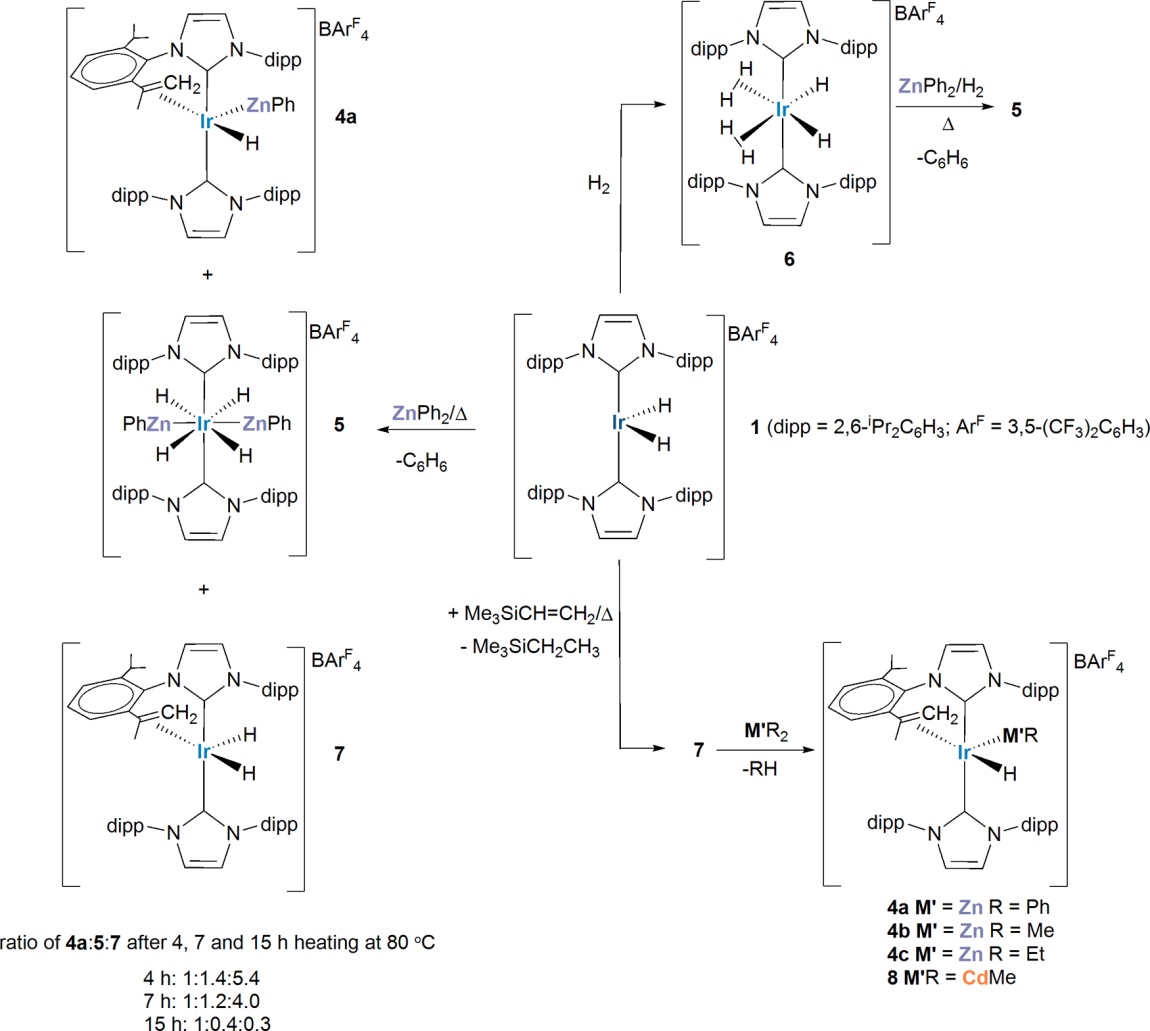
Reactivity of [Ir­(IPr)_2_H_2_]­[BAr^F^_4_] (**1**) with ZnPh_2_ and Separate Syntheses of the Resulting Products and Their Derivatives^a^

a Any agostic interactions are omitted for clarity.

The three products were identified following independent syntheses. The nonzinc-containing dihydride salt **7** was reported previously as the main product formed upon stirring Ir­(IPr)_2_H_2_Cl with Na­[BAr^F^_4_] in C_6_H_5_F for ca. 12 h at room temperature; shorter reaction times (3–4 h) gave **1**.^16^ However, in our hands, **1** was the only observable species even after stirring Ir­(IPr)_2_H_2_Cl and Na­[BAr^F^_4_] for 24 h at room temperature, or upon heating for 12 h at 60 °C. Instead, we prepared **7** in 73% isolated yield by heating **1** at 80 °C with a hydrogen acceptor, Me_3_SiCH=CH_2_. By ^1^H NMR spectroscopy, **7** showed two Ir–H resonances (Figure S40) at δ −13.6 and δ −41.2 (d, ^2^ *J*_HH_ = 8.2 Hz) in a 1:1 ratio, the very low frequency resonance reflecting the presence of an apical hydride ligand in the structurally determined square-pyramidal structure.^18,19^ We are unable to explain why our data differ to those reported, a singlet of integral 2 at δ −34.0.^16,18,20^

Clean formation of the Ir–ZnPh salt [Ir­(IPr)­(IPr″)­(ZnPh)­H]­[BAr^F^_4_] (**4a**) was achieved upon (i) heating **7** with ZnPh_2_ or (ii) heating **1** with a mixture of ZnPh_2_ and Me_3_SiCH=CH_2_. It was identified by the presence of three ^1^H NMR resonances (δ 4.01, 3.18 and 1.48) for the dehydrogenated isopropyl substituent of the IPr″ ligand in a 1:1:3:1 ratio with a single hydride resonance at δ −7.41 (Figure S3). The X-ray structure and computational analysis of **4a** (and other species in Scheme 3 that have been characterized crystallographically) are discussed in a later section (Figures 1 and 2).

**Figure 1:**
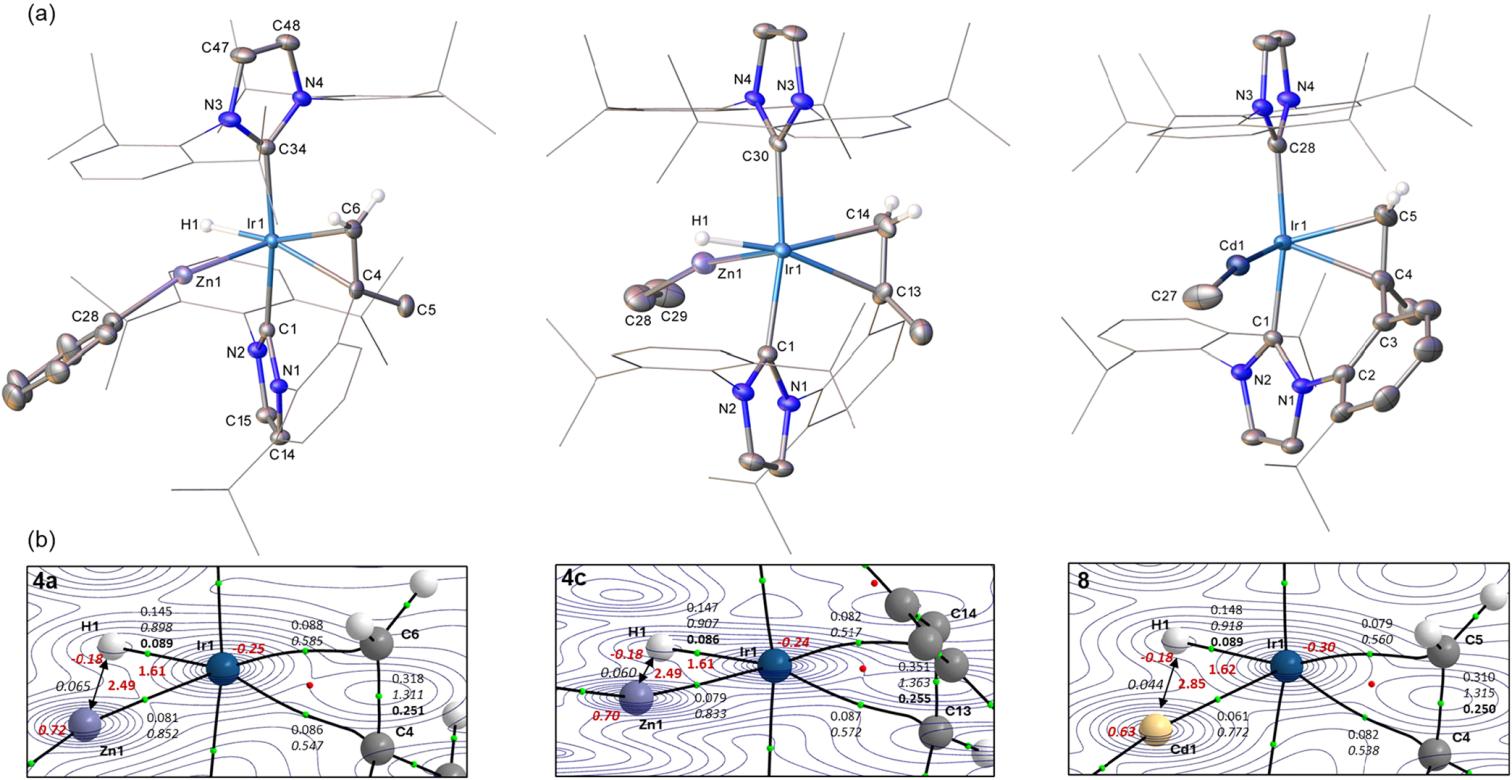
(a) Structures of the cations in [Ir­(IPr)­(IPr″)­(ZnPh)­H]­[BAr^F^_4_] (**4a**), [Ir­(IPr)­(IPr″)­(ZnEt)­H]­[BAr^F^_4_] (**4c**), and [Ir­(IPr)­(IPr″)­(CdMe)­H]­[BAr^F^_4_] (**8**). In all cases, ellipsoids are displayed at 30% probability and nondehydrogenated IPr substituents in wireframe. Nonhydride and -alkene H atoms, as well as the minor disordered atoms in **4c** and **8**, are omitted for clarity. The hydride ligand in **8** was not located. (b) QTAIM molecular graphs (optimized H atom positions) with density contours in the {M′Ir1H1} plane (M′ = Zn, **4a**, **4c**; M′ = Cd, **8**), showing computed Ir–H and Zn–H distances (Å, in red, plain text) and QTAIM atomic charges (in red, italics). BCPs (green spheres) show the associated ρ­(r) (au) in plain text, delocalization indices in italics and, for bond paths to hydrogens, ellipticities in bold. M′···H1 delocalization indices also indicated.

**Figure 2:**
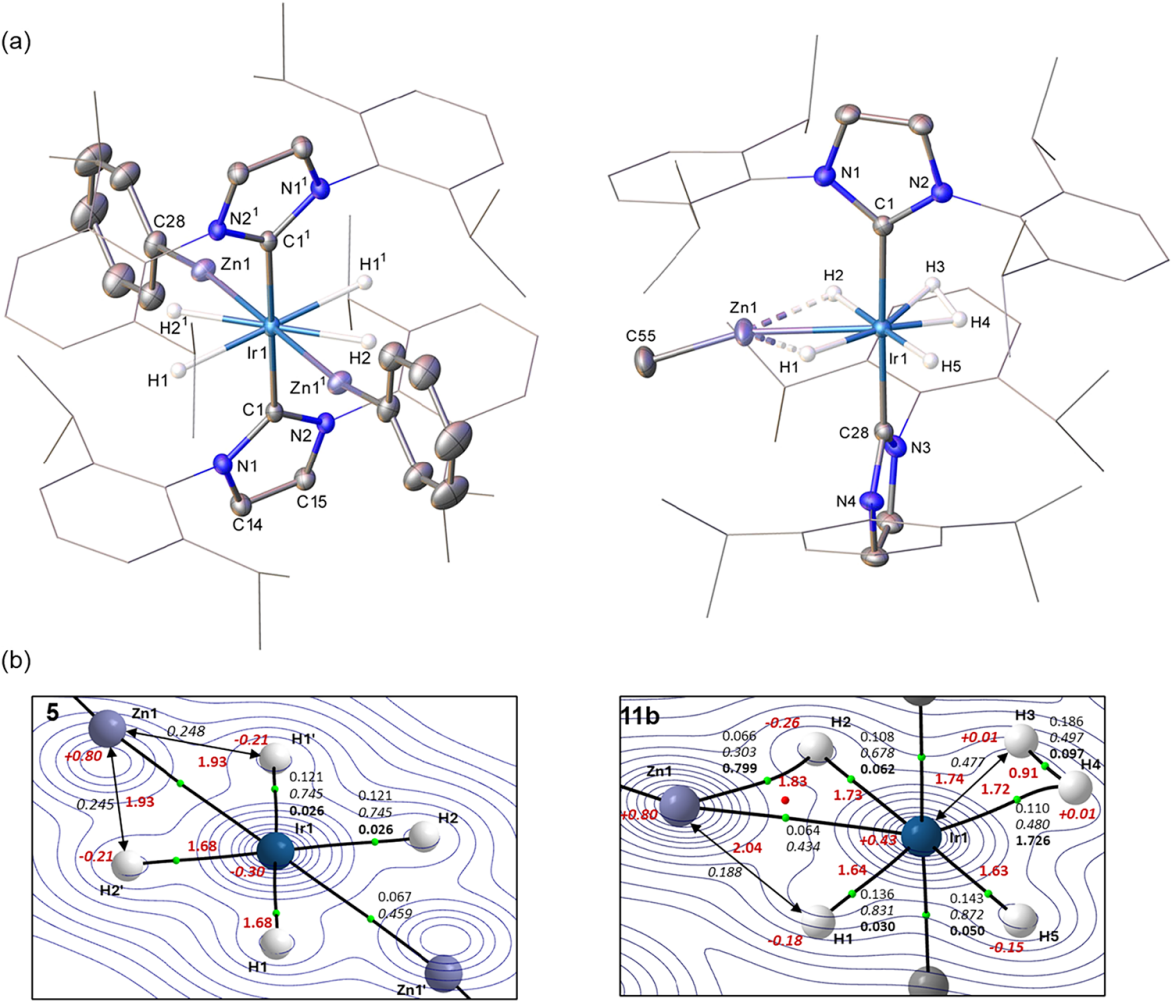
(a) Structures of the cations in [Ir­(IPr)_2_(ZnPh)_2_H_4_]­[BAr^F^_4_] (**5**) and [Ir­(IPr)_2_(ZnMe)­H_5_]­[BAr^F^_4_] (**11b**). Ellipsoids are depicted at 30% probability in both cases. Carbene substituents have been depicted as wireframes for visual ease. In **5**, symmetry operation for atoms with superscripted labels: ^1^ 1/2 – *x*, 3/2 – *y*, 1 – *z*; in **11b** non-IPr H atoms and minor disordered atoms are omitted for clarity. (b) QTAIM molecular graphs (optimized H atom positions) with density contours in the {Zn1Ir1H1} plane showing computed Ir–H and Zn–H distances (Å, in red, plain text) and QTAIM atomic charges (in red, italics). BCPs (green spheres) show the associated ρ­(r) (au) in plain text, delocalization indices in italics and, for bond paths to hydrogens, ellipticities in bold. Zn···H1 delocalization indices also indicated.

The formation of the third of the reaction products shown in Scheme 3, the dizinc tetrahydride salt [Ir­(IPr)_2_(ZnPh)_2_H_4_]­[BAr^F^_4_] (**5**), can be attributed to the reaction of [Ir­(IPr)_2_(η^2^-H_2_)_2_H_2_]­[BAr^F^_4_] (**6**), generated by addition to **1** of the H_2_ released in the formation of **4a** and **7**,^15^ with 2 equiv ZnPh_2_, followed by the elimination of two molecules of benzene. In line with this, directly heating **1** at 80 °C with 2 equiv ZnPh_2_ under 1 atm H_2_ cleanly formed **5**. This showed a ^1^H NMR spectrum very different (a single, temperature invariant Ir–H resonance (Figure S31) in a 4:8:24:24 ratio with the ^i^Pr methine and methyl resonances) to that of the ZnMe analogue **3** (one broad, room temperature Ir–H resonance that decoalesced into a 1:1:2 set of resonances at −50 °C), suggestive of an alternative geometry to the 1,3-arrangement of ZnR groups in **3**. A 1,4-arrangement of ZnPh groups was subsequently confirmed by X-ray crystallography (Figure 2, vide infra).

### Synthesis of [Ir­(IPr)­(IPr″)­(M′R)­H]­[BAr^F^_4_] (M′R = ZnMe, ZnEt, CdMe) and Reactions with H_2_

The formation of mono Ir–M′R species [Ir­(IPr)­(IPr″)­(M′R)­H]­[BAr^F^_4_] was not limited to the ZnPh derivative **4a**. Treatment of **7** with ZnMe_2_, ZnEt_2_ and CdMe_2_ (all at room temperature cf. **4a**) gave [Ir­(IPr)­(IPr″)­(ZnMe)­H]­[BAr^F^_4_] (**4b**), [Ir­(IPr)­(IPr″)­(ZnEt)­H]­[BAr^F^_4_] (**4c**) and [Ir­(IPr)­(IPr″)­(CdMe)­H]­[BAr^F^_4_] (**8**) respectively (Scheme 3).^21^ The products were identified in the first instance by the similarity of their Ir–H chemical shifts to that of **4a** (**4b**: δ −9.32; **4c**: δ −8.07; **8**: δ −9.29; Figures S10, S21, S51)^22^ and confirmed in the cases of **4c** and **8** by X-ray crystallography (vide infra).

Exposure of THF solutions of **4b**-**c** and **8** to 1 atm H_2_ generated mixtures of the trihydride salts [Ir­(IPr)­(IPr″)­(M′R)­H_3_]­[BAr^F^_4_] (**9b**-**c, 10**) and pentahydride salts [Ir­(IPr)_2_(M′R)­H_5_]­[BAr^F^_4_] (**11b**-**c, 12**) over minutes at room temperature, with near complete conversion to the latter observed over ca. 1 h (Scheme 4 and Figures S64 and S68). As a result of this onward reactivity, we were unable to isolate **9b**-**c** or **10**, although spectroscopically clean samples could be prepared by addition of H_2_ to samples of **4b**-**c** and **8**, followed by rapid insertion into a precooled NMR probe at 248 K (Figures S65, S69 and S72).^23^ As for the CdMe derivatives of **2** and **3** in Scheme 2, the *J*_HCd_ splittings on the three hydride resonances of [Ir­(IPr)­(IPr″)­(CdMe)­H_3_]­[BAr^F^_4_] (**10**: δ −8.24 (628 Hz), −12.08 (42 Hz), −14.02 (114 Hz); Figure S74) afforded information about structure.^24^ The resonance at ca. δ −8 with the largest coupling was assigned to the hydride bridging Ir and Cd (H_a_, Scheme 4a). This also exhibited a doublet trans-^2^ *J*_HH_ splitting of 11 Hz^25,26^ to H_b_, for which ^2^ *J*_HCd_ = 114 Hz. The remaining hydride H_c_ showed no coupling to either H_a_ or H_b_, but an NOE interaction between H_c_ and H_b_ confirmed their presence in the same molecule.^27–30^ In the absence of crystallographic data, full DFT optimizations on possible structures for **10** (Scheme 4b) supported the presence of two hydrides, H_a_ and H_c_, on either side of the Ir–Cd vector, with H_a_ (trans to the terminal Ir–H_b_ bond), interacting more strongly with the Cd center than H_c_ (trans to the alkene; computed distances: Cd–H_a_ = 1.90 Å; Cd–H_c_ = 2.65 Å). Alternative isomers (with CdMe adjacent to the alkene moiety and a dihydrogen hydride form) were significantly higher in energy (Figure S152).

**Scheme 4:**
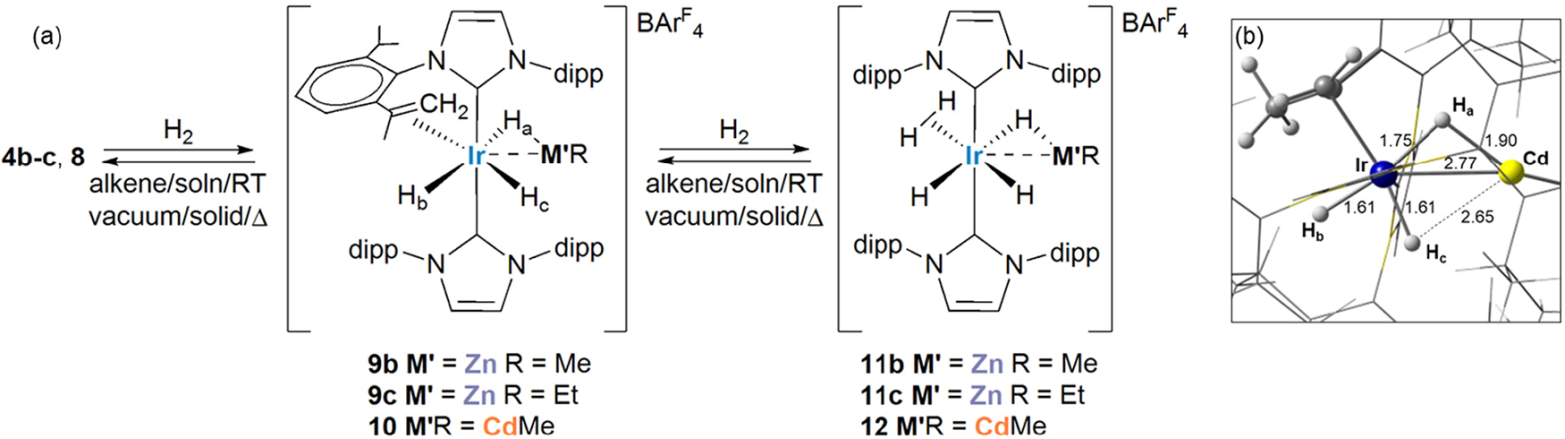
(a) Formation and Reactivity of Ir–Zn/Cd Tri- and Pentahydride Salts; (b) Detail of the Computed Structure of the Cation of **10** ^a^

a Selected distances in Å; method: BP86/SDD­(Ir, Cd), 6-31G** other atoms; axial ligands shown in wireframe for clarity.

Generation of **9b**-**c** and **10** as trihydride species in which the IPr″ ligand is retained fits with other Ir complexes in which preferential addition of H_2_ to the metal center rather than a dehydrogenated NHC (or, indeed, dehydrogenated phosphine) ligand is also observed.^31,32^

NMR studies (Figures S75–S95) of the pentahydride salts **11b**-**c** and **12** showed them to (i) all feature two intact IPr ligands, (ii) all be highly fluxional (single hydride resonances of relative integral 5 observed at ca. δ −8 to −9 between 323 and 228 K) and (iii) exhibit short *T*_1_ times (**11b**: 32 ms (248 K, 400 MHz); **11c**: 45 ms (248 K, 500 MHz); **12**: 45 ms (248 K, 400 MHz)), consistent with nonclassical structures (vide infra).^33^ Given the fluxionality and nonclassical properties, the formation of free HD in the ^1^H NMR spectrum of a sample of the ZnEt derivative **11c** exposed to 1 atm D_2_ was unsurprising (Figure S96). The elimination of three molecules of H_2_ from **11c** to reform **4c** could be brought about at room temperature in solution by addition of styrene or in the solid-state by heating **11c** at 60 °C under dynamic vacuum overnight (Figures S97 and S98).

### Crystallographic and Computational Studies of **4a**, **4c**, **5**, **8** and **11b**

The X-ray crystal structures of the cations in the three Ir–MR′ salts **4a**, **4c** and **8** are shown in Figure 1a. They all display distorted square-pyramidal structures, with MR′ groups in the apical site tilted over toward the hydride ligand in the basal plane (e.g., ∠alkene centroid–Ir1–Zn1 = 110.80(5)° in **4a**). As in other IPr″ species,^16,32^ the C=C bond of the dehydrogenated arm sits almost perfectly parallel to the basal plane (e.g., C1–Ir1–alkene centroid–C6 angle of 177.6° in **4a**). Salts **4a** and **4c**, which show similar and rather short Ir–Zn distances (2.3523(3) and 2.3651(6) Å respectively) as a result of the ZnR ligands being trans to a vacant site, provide additional examples of square-pyramidal TM species with apical ZnR ligands (TM = Ru, Rh),^34–38^ and compare with related TM–HgR (TM = Rh, Ir)^39,40^ compounds. Salts **4a**, **4c** and **8** appear to be the first to present this motif in the presence of a hydride ligand, and the apical positioning of the M′R ligands indicates a higher trans influence than hydride.

The similarities between **4a**, **4c** and **8** are also reflected in the DFT-calculated structures, where the H atoms are optimized around fixed heavy atom positions derived from the X-ray structures. These and the associated QTAIM molecular graphs are displayed in Figure 1b and show computed Ir–H distances around 1.61 Å consistent with terminal hydride character. These align with both the relatively large electron densities, ρ­(r), and delocalization indices, DI, at the associated bond critical points (BCPs: ρ­(r) ≈ 0.15 au; DI ≈ 0.90) although the BCP ellipticities (ε ≈ 0.09) are somewhat larger than expected.^15^ This implies some perturbation of the electron topology likely due to the cis-M′R groups, although the long Zn1···H1 distances in **4a** and **4c** and the low associated DIs (<0.07) indicate minimal interaction that becomes weaker still in **8**. The Ir–Zn interactions in **4a** and **4c** are not significantly affected by the change in substituent (ρ­(r) ≈ 0.08; DI ≈ 0.84) but appear somewhat stronger than the Ir–Cd interaction in **8** (ρ­(r) = 0.061; DI = 0.772). This is consistent with the elongated Ir–Cd distances of the two molecules in the unit cell of **8** (2.6499(15)/2.6497(2) Å), expected on the basis of the respective covalent radii (Zn 1.22 Å, Cd 1.44 Å).^41–43^

The X-ray structure of the cation in **5** displayed two trans-ZnPh ligands and two pairs of cis-hydride ligands placed on either side of the Zn–Ir–Zn vector in the equatorial plane (Figure 2a). This 1,4-trans arrangement contrasts with the 1,3-geometry found in [Ir­(IPr)_2_(ZnMe)_2_H_4_]­[BAr^F^_4_], (**3**, Scheme 2) where one hydride sits between the two ZnMe ligands.^15^ This difference may be a result of the increased sterics of the phenyl substituents on Zn.^44^ Thus, the two axial IPr ligands in **5** exhibit an eclipsed arrangement of the dipp substituents that accommodates the ZnPh groups with the Ph substituents lying perpendicular to the imidazolylidene rings. The Ir–Zn distances in **5** (2.4607(3)/2.4608(3) Å) are somewhat shorter than those in **3** (2.4878(4)/2.4942(5) Å),^15^ while still being significantly longer than the apical Ir–Zn distances in **4a** and **4c**.

In the X-ray crystal structure of the ZnMe pentahydride salt **11b**, two hydrides (H1 and H2) were located bridging the Ir1–Zn1 bond (2.4766(4) Å), alongside a terminal hydride ligand (H5), all three of which had credible *U*_iso_ values (<0.05). H3 and H4 were tentatively assigned to an η^2^-H_2_ ligand, as the *U*_iso_ values were slightly larger (0.16047 and 0.09576) and restraints were necessary for placement of H3. Further insight into the H atom positions was provided by the DFT-optimized geometries in Figure 2b. For **5**, long Ir–H distances of 1.68 Å are computed, reflecting the trans-H–Ir–H arrangement. The computed Zn1···H1/H2 distances (1.93 Å) are much shorter than in **4a** and **4c** above and the associated DIs are significant (ca. 0.24) indicative of some bridging character;^13,45^ overall the computed metrics are very similar to the symmetrical trans-H–Ir–H unit in **3**.^15^ A degree of hydride-bridging character may also attenuate the trans influence of the ZnR ligand noted in **4a** and **4c**, that allows them to adopt a mutually trans arrangement in **5**. This is also reflected in the reduced ρ­(r) and DI values associated with the Ir–Zn BCPs and the longer Ir–Zn distances compared to the equivalent metrics in **4a** and **4c**.

Calculations on **11b** support the [Ir­(IPr)_2_(ZnMe)­(η^2^-H_2_)­H_3_]^+^ formulation proposed from the X-ray study. The Ir–H5 bond exhibits terminal character (Ir–H5 = 1.63 Å; ρ­(r) = 0.143, DI = 0.872, ε = 0.050) but the trans H5–Ir–H2 unit is now unsymmetrical due to the H2–Zn1 bridging interaction that weakens the Ir–H2 bond (Ir1–H2 = 1.73 Å; ρ­(r) = 0.108, DI = 0.678). In this case, a Zn1–H2 bond path is seen (Zn1–H2 = 1.83 Å; ρ­(r) = 0.066, DI = 0.303) as well as a far greater ellipticity at the Zn1–H2 BCP (0.799, cf. 0.062 for Ir1–H2). The Ir1–H1 bond is similar to Ir1–H5 reflecting its location trans to a weaker trans influence η^2^-H_2_ ligand and a relatively weak H1···Zn1 interaction (H1···Zn1 = 2.04 Å; DI = 0.188). Similar patterns have been seen in [Ru­(IPr)_2_(CO)­(ZnH)­H_3_]^+^ and [Ru­(IPr)_2_(CO)­(ZnEt)­(L)­H_2_]^+^ (L = η^2^-H_2_, vacant)^46,34^ that also feature an unsymmetrical {TM­(H)_2_M′} motif. As noted previously,^15^ an increase in bridging character (in **11b**: H5 < H1 < H2) aligns with a larger (more negative) QTAIM charge and hence greater hydridic nature.

### Lewis Base-Induced Elimination of “[M′R]­[BAr^F^_4_]” from [Ir­(IPr)_2_(M′R)­H_5_]­[BAr^F^_4_]: Formation of Ir­(IPr)_2_H_5_

During early studies of the reactions of **4b**-**c** with H_2_, the formation of **11b**-**c** was commonly accompanied by small, although inconsistent, amounts of the neutral pentahydride complex Ir­(IPr)_2_H_5_ (**13**), which we postulate forms through adventitious moisture-induced elimination of “[ZnR]­[BAr^F^_4_]” (Scheme 5).^47^ In support of this, dissolution of a crystalline sample of **11c** in degassed, but undried THF, gave a 14% yield of **13**, whereas a yield of <1% was formed in Na/K-dried THF (Figures S100–S103).

**Scheme 5:**
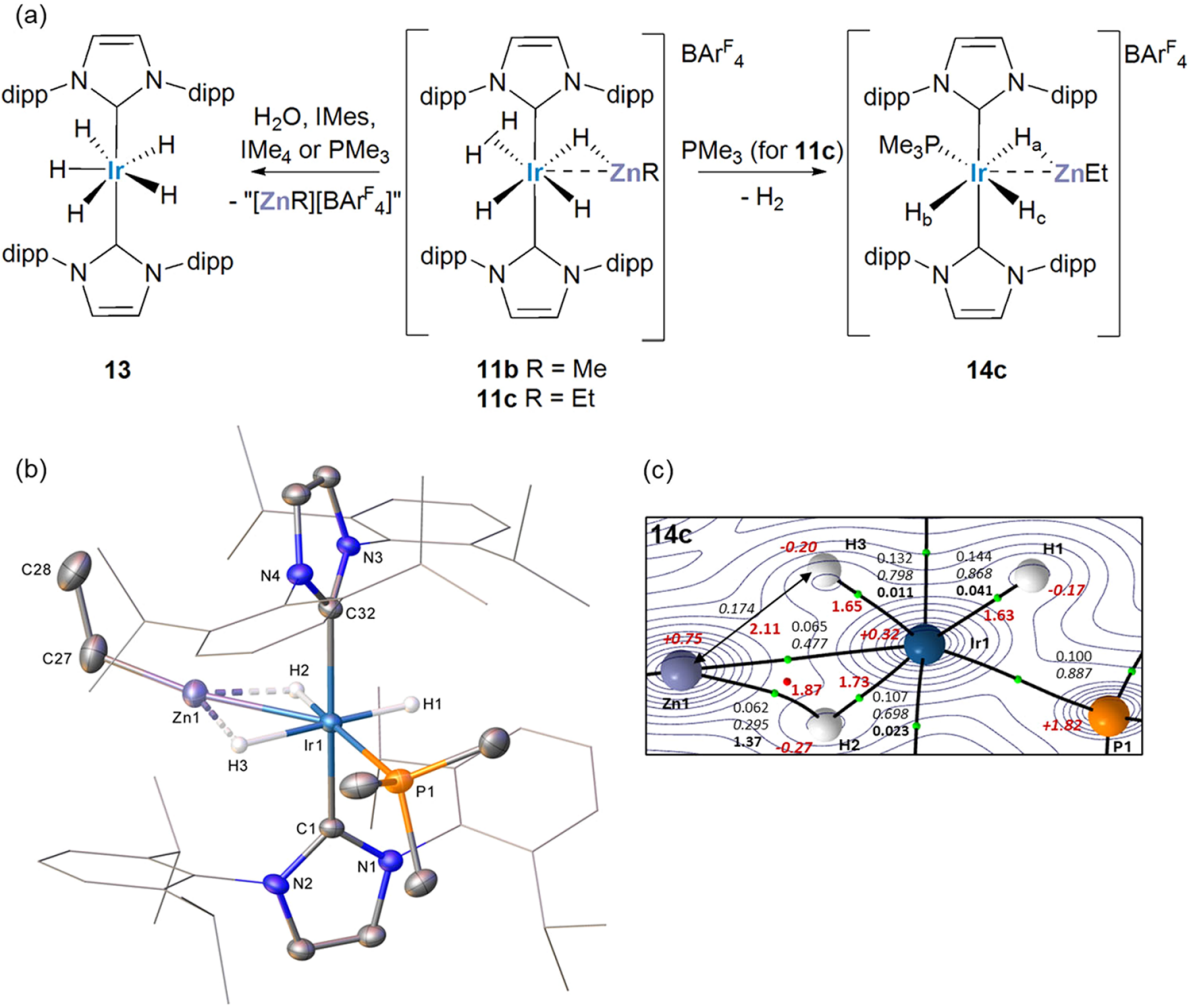
(a) [ZnR]^+^ Loss and Phosphine Substitution Reactions of [Ir­(IPr)_2_(ZnR)­H_5_]­[BAr^F^_4_], **11b** and **11c**; (b) Plot Depicting the Structure of the Cation in [Ir­(IPr)_2_(PMe_3_)­(ZnEt)­H_3_]­[BAr^F^_4_] (**14c**);^a^ (c) QTAIM Molecular Graph (Optimized H Atom Positions) of **14c** ^b^

a Ellipsoids are depicted at 30% probability. The majority of hydrogen atoms have been omitted for clarity. Carbene substituents have been depicted as wireframes, also for visual ease.
b Density contours in the {Zn1Ir1H2} plane showing computed Ir–H and Zn–H distances (Å, in red, plain text) and QTAIM atomic charges (in red, italics). BCPs (green spheres) show the associated ρ­(r) (au) in plain text, delocalization indices in italics and, for bond paths to hydrogens, ellipticities in bold. Zn···H1 delocalization indices also indicated.

We were unable to determine the structure of the “[ZnR]­[BAr^F^_4_]” species eliminated in the reaction. However, when **11c** was dissolved in Na/K-dried THF in the presence of 1,3-bis­(2,4,6-trimethylphenyl)­imidazol-2-ylidene (IMes, 2 equiv), quantitative conversion to **13** was accompanied by ^1^H NMR resonances matching those of the bis-carbene adduct, [(IMes)_2_ZnEt]­[BAr^F^_4_] (Figure S104).^48^ Changing IMes to IMe_4_ (1,3,4,5-tetramethylimidazol-2-ylidene) or PMe_3_ similarly gave **13** (Figures S105 and S113), although we were again unable to identify the eliminated carbene/phosphine [ZnEt]­[BAr^F^_4_] adduct in either reaction. DFT calculations showed the formation of [L_2_ZnMe]^+^ from the cation of **11b** to be exergonic for all added ligands L, and most favorable when L = IMes (Δ*G* = −40.7 kcal/mol; all structures fully optimized and energies including a correction for THF solvent, see Table S2).^49,50^

With PMe_3_, a small amount of the phosphine substitution product [Ir­(IPr)_2_(PMe_3_)­(ZnEt)­H_3_]­[BAr^F^_4_] (**14c**) was also formed (Scheme 5). X-ray crystallography showed this to be isostructural to **11b** with PMe_3_ in place of the dihydrogen ligand. The Ir(1)–Zn(1) distance of 2.4796(9) Å suggests a similar degree of Ir···Zn interaction as in **5** and **11b** above. The Ir–Zn vector is straddled by hydrides H2 and H3 which have Ir–H distances of 1.56(5) and 1.86(7) Å respectively, in line with terminal and bridging character. This trend was confirmed in the computed structure and associated QTAIM metrics in Scheme 5c, while noting the relatively large esds on the experimental Ir–H distances. Unlike in **11c**, there was no evidence for fluxionality in the three hydride resonances of **14c** (δ −9.37, −11.38 and −12.01) over the temperature range 328 to 193 K. Assignment of the hydride resonances as H_a_ to H_c_ (Scheme 5a) was done on the basis of ^2^ *J*_HP_ values and ^1^H,^1^H-NOESY interactions (Figure S119).

### Synthesis of **13** and Reactivity with M′R_2_

Formulation of **13** as Ir­(IPr)_2_H_5_ was confirmed through an alternative synthesis involving the deprotonation of the bis­(dihydrogen) dihydride salt **6** ^15^ by KN­(SiMe_3_)_2_, which afforded the complex in 71% isolated yield. In accord with the well-known phosphine analogues Ir­(PCy_3_)_2_H_5_ and Ir­(P^i^Pr_3_)_2_H_5_,^51–53^ the IMes derivative exhibited properties consistent with a ′classical′ pentahydride, namely a long *T*_1_ value (938 ms, 228 K, 400 MHz; 799 ms at 298 K) for the single hydride^54,55^ resonance at δ –10 (the appearance of which was unchanged between 298 and 228 K) and the splitting of the Ir–*C*_IPr_ resonance into a sextet (^2^ *J*_CH_ = 4 Hz) in the ^13^C­{selective-^1^H} NMR spectrum (Figure S111).^56–58^ Full DFT optimizations with the BP86 functional also identified a pentagonal bipyramidal pentahydride ground state structure (see Supporting Information).^59–61^ In contrast to the many known examples of late transition metal polyhydride compounds with phosphine ligands,^1,2^ **13** represents one of the very few known NHC analogues.^58,62–66^

Prompted by the range of Ir–ZnR/CdR products accessible from cationic **1**, the reactivity of neutral **13** with ZnR_2_ and CdMe_2_ was also investigated (Scheme 6). In all cases, R–H elimination took place upon heating (50–100 °C) with a slight excess of ZnR_2_ (R = Ph, Et, Me) or CdMe_2_ to yield the neutral mono-M′R tetrahydride products Ir­(IPr)_2_(ZnR)­H_4_ (**15a**-**c**) and Ir­(IPr)_2_(CdMe)­H_4_ (**16**). These were isolated in good yields (50–60%) as colorless microcrystalline solids that were fully characterized by 1- and 2-D NMR methods (Figures S123–S148), as well as by X-ray crystallography in the case of the ZnMe derivative **15b**.

**Scheme 6:**
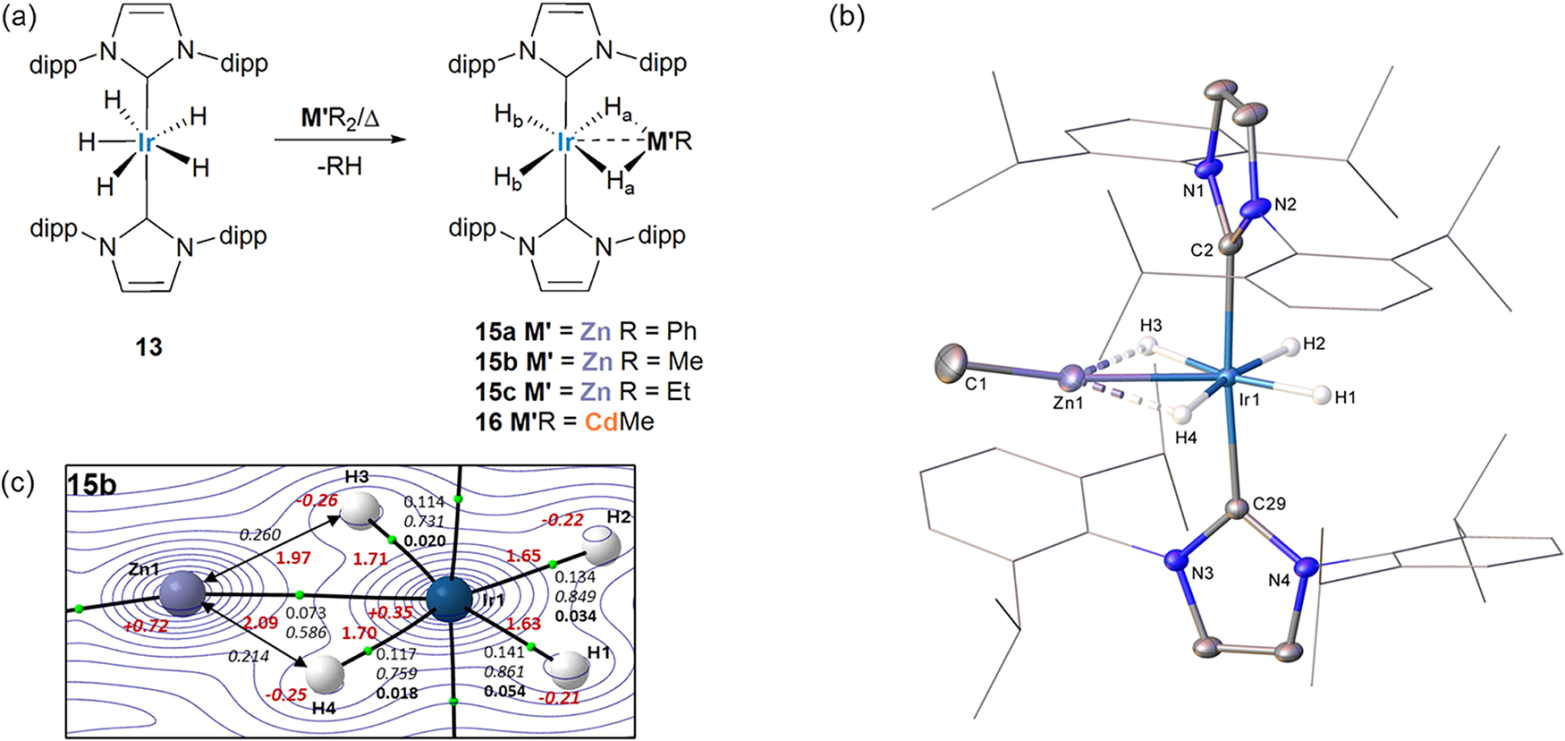
(a) Reaction of Ir­(IPr)_2_H_5_ (**13**) with ZnR_2_ (R = Ph, Me, Et) and CdMe_2_ to form Ir­(IPr)_2_(M′R)­H_4_ (**15a-c, 16**); (b) Molecular Structure of Ir­(IPr)_2_(ZnMe)­H_4_ (**15b**);^a^ (c) QTAIM Molecular Graph (Optimized H Atom Positions) of **15b** ^b^

a Ellipsoids are depicted at 30% probability. The majority of hydrogen atoms, plus solvent, have been omitted for clarity. Carbene substituents have been depicted as wireframes, also for visual ease.
b Density contours in the {Zn1Ir1H3} plane showing computed Ir–H and Zn–H distances (Å, in red, plain text) and QTAIM atomic charges (in red, italics). BCPs (green spheres) show the associated ρ­(r) (au) in plain text, delocalization indices in italics and, for bond paths to hydrogens, ellipticities in bold. Zn···H3//H4 delocalization indices also indicated.

At room temperature, the ^1^H NMR spectra of the four compounds (Figures S123, S129, S134 and S141) all exhibited two hydride resonances (1:1 relative ratio) between ca. δ −10.5 and −11.5 with *T*_1_ times ranging from 340 to 488 ms (228 K, 400 MHz). In all cases, ROESY measurements showed the hydrides to be in exchange either at or just below room temperature (Figures S128, S133, S140 and S148); variable temperature NMR measurements on **15b** showed retention of two separate resonances at all temperatures across the range 318–228 K (Figure S130). From the *J*_HCd_ couplings of 350 and 95 Hz in **16**, the hydrides were assigned to those bridging between Ir and Cd (H_a_, Scheme 6a) and those terminally bound to Ir (H_b_).^67,68^ The IR spectra (Figure S149) were consistent with this arrangement, showing a very weak feature at ca. 2100 cm^–1^ consistent with Ir–H_terminal_ and a stronger band at ca. 1700 cm^–1^ assigned to Ir–H_bridging_.

The structure of **15b** determined from the X-ray data (Scheme 6b) shows some asymmetry in the Ir1–H3 and Ir1–H4 distances (1.85(5) and 1.59(5) Å) and, to a lesser extent, the Ir1–H3···Zn1 and Ir1–H4···Zn1 angles (78.14°, 82.37°). The Ir···Zn distance was 2.4183(5) Å. The presence of two distinct pairs of hydrides is confirmed computationally with two shorter (terminal) Ir–H1 and Ir–H2 bonds (average data: Ir–H = 1.64 Å, ρ­(r) = 0.137 au; DI = 0.855, ε = 0.044) opposite to longer Ir1–H3/Ir1–H4 bonds (average data: Ir–H = 1.70 Å, ρ­(r) = 0.116 au; DI = 0.745, ε = 0.019). While no Zn1–H3/H4 bond paths are seen, the computed Zn1–H3/H4 distances (1.97/2.09 Å) and DIs (0.260/0.214) indicate some interaction is present and this is larger for H3.

## Conclusions

In conclusion, we have shown that dehydrogenation of [Ir­(IPr)_2_H_2_]­[BAr^F^_4_] (**1**) opens up further avenues for M′R_2_ species (M′= Zn; R = Ph, Me, Et; M′ = Cd, R = Me) to react with cationic Ir–H species via R–H elimination. The resulting products are the square-pyramidal salts [Ir­(IPr)­(IPr″)­(M′R)­H] [BAr^F^_4_] (**4a**–**c**, **8**) which feature a dehydrogenated IPr″ ligand and axial M′R groups, showcasing the high trans influence of these *Z*-type ligands. Compounds **4b**–**c** and **8** all add three molecules of H_2_ to form the highly fluxional dihydrogen trihydride salts [Ir­(IPr)_2_(M′R)­(η^2^-H_2_)­H_3_]­[BAr^F^_4_] (**11b**-**c**, **12**). The presence of water or addition of other Lewis bases induces loss of the [M′R]^+^ moiety and formation of the classical pentahydride complex Ir­(IPr)_2_H_5_ (**13**), which also reacts with M′R_2_ (again via R–H elimination) to generate the complementary series of neutral heterometallic complexes, Ir­(IPr)_2_(M′R)­H_4_ (**15a**-**c**, **16**). The elimination of [M′R]^+^ from **11b**-**c** and **12** adds to our previous observations of [ZnR]^+^ and ZnR_2_ loss from cationic and neutral ruthenium–zinc heterometallic species, respectively.^46,50^ The breadth of such degradation reactions in transition metal-Lewis acid heterometallic chemistry is worthy of further investigation given the obvious detrimental influence it could have on catalytic applications.^69^

Reaction of **1** with ZnPh_2_ and H_2_ produces [Ir­(IPr)_2_(ZnPh)_2_H_4_]­[BAr^F^_4_], **5**, which exhibits a trans-1,4-arrangement of ZnPh groups. This contrasts with the 1,3-arrangement of ZnMe ligands in [Ir­(IPr)_2_(ZnMe)_2_H_4_]­[BAr^F^_4_], that we speculate reflects the greater steric impact of the ZnPh ligand. This may also account for the somewhat more forcing conditions required for the reactions of ZnPh_2_. QTAIM analyses of the crystallographically characterized species highlight subtle trends in the {L_n_Ir­(H)_2_M′R} moiety, where two hydrides straddle the Ir···Zn vector. Higher trans influence ligands, L, tend to weaken the Ir–H bond and so promote bridging interactions with the M′R unit; these patterns confirm and add to similar trends reported in earlier work on related TM–Zn systems.^15,46^

## Experimental Section

For general information, experimental procedures, characterization data and computational details, see the Supporting Information. *Caution*! *Dimethyl cadmium is extremely hazardous and should be handled with utmost care using appropriate PPE*. To minimize the hazards, reactions with CdMe_2_ were typically conducted on NMR tube scales using a maximum of 10 μL of a 2.4 M toluene solution of the reagent. Otherwise, no uncommon hazards are noted.

## Supplementary Material





## Notes

Computed geometries (XYZ)
Experimental procedures, characterization data, NMR/IR spectra, computational details (PDF)
