# Supplementary material for: Cationic and Neutral Heterometallic Ir-Group 12 Element Polyhydride Compounds: Synthesis, Structure and Reactivity
Source: Inorg Chem. 2026 Jan 1;65(2):1162–71. doi: 10.1021/acs.inorgchem.5c04368 (PMC12820928; doi:10.1021/acs.inorgchem.5c04368)
Supplement: Supplementary file 2 [file ic5c04368_si_002.pdf]

SUPPORTING INFORMATION

Cationic and Neutral Heterometallic Ir–Group 12 Element Polyhydride Compounds: Synthesis, Structure and Reactivity

Amber M. Walsh,[a] Carlos Martín-Fernández,[b] John P. Lowe,[a] Stuart A. Macgregor,[b,*] Mary F. Mahon[a] and Michael K. Whittlesey[a]

[a] *Department of Chemistry, University of Bath, Bath, BA2 7AY, United Kingdom*

[b] *EaStCHEM School of Chemistry, University of St Andrews, North Haugh, St Andrews KY16 9ST, United Kingdom*

*(Corresponding author): sam38@st-andrews.ac.uk

CONTENTS

S1 Experimental S2

S1.1 General comments S2

S1.2 Experimental details and characterization data S2

S1.3 X-ray crystallography S18

S1.4 NMR and IR spectra S24

S2 Computational studies S173

S2.1 Computational details S173

S2.2 Computational studies on ′[ZnMe]$^+$ ′ abstraction S174

S2.3 Isomers of [**5**]$^+$ S178

S2.4 Alternative Isomers of **[10]$^+$.** S181

S2.5 Functional testing on the structures of **13** and **15b** S182

S2.6 Effect of solvent on the computed structure of **13** S184

S2.6 QTAIM analyses S185

S2.7 Cartesian coordinates (Å) and energies (a.u.) for the computed structures S192

S3 References S252

S1 Experimental

S1.1 General comments

All manipulations were carried out under argon using standard Schlenk, high vacuum and glovebox techniques using dry and degassed solvents. C_6D_6, $C_6D_5CD_3$ and THF-d_8 were vacuum transferred from potassium or NaK, CD_2Cl_2 and C_6D_5F from CaH_2. NMR spectra were recorded on Bruker Avance 400 and 500 MHz NMR spectrometers and referenced as follows: THF-d_8 (^{1}H, δ 3.58; ^{13}C, δ 67.6), C_6D_6 (^{1}H, δ 7.15; ^{13}C, δ 128.0), $C_6D_5CD_3$ (^{1}H, δ 2.09; ^{13}C, δ 20.4), CD_2Cl_2 (^{1}H, δ 5.32; ^{13}C, δ 54.0), C_6D_5F (^{1}H, δ 7.11; ^{13}C, δ 163.2).[1] ^{1}H NMR spectra recorded unlocked in C_6H_5F were referenced to the most upfield shifted multiplet set to δ 7.11. ^{31}P{^{1}H} NMR spectra were referenced to H_3PO_4 at δ = 0. ^{113}Cd{^{1}H} NMR spectra (referenced to the proton resonance of TMS according to the unified scale) were recorded with inverse-gated decoupling. IR spectra were recorded on a Bruker ALPHA ATR-IR spectrometer inside a glovebox. Electrospray ionisation (ESI) mass spectrometry was performed on a Bruker MaXis HD ESI-QTOF mass spectrometer. Elemental analyses were performed by Elemental Microanalysis Ltd, Okehampton, Devon, U.K.

$[Ir(IPr)_2H_2][BAr^F_4]$ (**1**),[2] $[Ir(IPr)_2(\eta^2\text{-}H_2)_2H_2][BAr^F_4]$ (**6**),[3] IMes,[4] and IMe_4[5] were prepared according to the literature. $ZnPh_2$, $ZnMe_2$ (1.2 M solution in toluene), $ZnEt_2$ (1.0 M solution in hexane), H_2, D_2 and PMe_3 were used as received. $CdMe_2$ (2.4 M in toluene) was provided by Professor Andrew Johnson (*CAUTION: Dimethyl cadmium is extremely hazardous and should be handled with utmost care employing appropriate safety measures*).[6] To minimize the hazards associated with cadmium compounds, reactions were typically conducted on NMR tube scales using a maximum of 10 µL of the toluene solution of $CdMe_2$ in order to provide enough of the Ir–Cd heterometallics to allow definitive spectroscopic characterization.

S1.2 Experimental details and characterization data

[Ir(IPr)(IPr′′)(ZnPh)H][BAr^F$_4$] (4a). A C_6H_5F (1 mL) solution of [Ir(IPr)(IPr′′)H$_2$][BAr^F$_4$] (**7**; 30 mg, 0.016 mmol) and $ZnPh_2$ (7 mg, 0.033 mmol) was heated at 80 °C for 12 h. The resulting solution was concentrated and layered with hexane to yield yellow crystals of **4a**. Yield: 21 mg (66%). ^{1}H NMR (THF-d_8, 500 MHz, 248 K): δ 7.83 (br s, 8H, BAr^F$_4$), 7.73 (d, $^3J_{HH}$ = 1.9 Hz, 1H, NC*H*=CHN), 7.69 (br s, 1H, NC*H*=CHN), 7.68 (br s, 1H, NC*H*=CHN), 7.65 (d, $^3J_{HH}$ = 1.9 Hz, 1H, NC*H*=CHN), 7.63 (br s, 4H, BAr^F$_4$), 7.54 (t, $^3J_{HH}$ = 7.6 Hz, 1H, Ar), 7.45 (t, $^3J_{HH}$ = 7.6 Hz, 1H, Ar), 7.34 (m, 1H, Ar),# 7.07 (d, $^3J_{HH}$ = 7.8 Hz, 1H, Ar),* 7.02 (t, $^3J_{HH}$ = 7.7 Hz, 1H, Ar), 6.88 (t, $^3J_{HH}$ = 7.2 Hz, 4H, Ar), 6.15 (d, $^3J_{HH}$ = 6.6 Hz, 2H, Ar), 4.01 (s, 1H, C(Me)=C*H*H), 3.18 (overlapping m, 2H, C*H*Me$_2$ + C(Me)=CH*H*), 2.98 (sept, $^3J_{HH}$ = 6.6 Hz, 1H, C*H*Me$_2$), 2.87 (sept, $^3J_{HH}$ = 6.6 Hz, 1H, C*H*Me$_2$), 2.73 (sept, $^3J_{HH}$ = 7.0 Hz, 1H, C*H*Me$_2$), 2.57 (sept, $^3J_{HH}$ = 7.0 Hz, 1H, C*H*Me$_2$), 2.37 (sept, $^3J_{HH}$ = 6.6 Hz, 1H, C*H*Me$_2$), 2.22 (sept, $^3J_{HH}$ = 6.6 Hz, 1H, C*H*Me$_2$), 1.48 (s, 3H, C(*Me*)=CH$_2$), 1.46 (d, $^3J_{HH}$ = 6.9 Hz, 3H, CH*Me$_2$*), 1.43 (d, $^3J_{HH}$ = 6.9 Hz, 3H, CH*Me$_2$*), 1.33-1.19 (m, 15H, CH*Me$_2$*), 1.07 (d, $^3J_{HH}$ = 6.8 Hz, 3H, CH*Me$_2$*), 0.98 (d, $^3J_{HH}$ = 6.8 Hz, 3H, CH*Me$_2$*), 0.89 (d, $^3J_{HH}$ = 6.4 Hz, 3H, CH*Me$_2$*), 0.83 (d, $^3J_{HH}$ = 6.8 Hz, 3H, CH*Me$_2$*), 0.45 (d, $^3J_{HH}$ = 6.8 Hz, 3H, CH*Me$_2$*), 0.09 (d, $^3J_{HH}$ = 7.0 Hz, 3H, CH*Me$_2$*), 0.00 (d, $^3J_{HH}$ = 6.7 Hz, 3H, CH*Me$_2$*), −7.41 (s, 1H, Ir−*H*). #The low aromatic integration results from partial overlap with residual C_6H_5F. ^{13}C{^{1}H} NMR (THF-d_8, 126 MHz, 248 K): δ 175.4 (s, Ir−C_{IPr}), 170.8 (s, Ir−C_{IPr}), 163.0 (1:1:1:1 quart, $^1J_{CB}$ = 50 Hz, BAr^F$_4$), 148.6 (s), 147.0 (s), 146.8 (s), 145.8 (s), 145.4 (s), 145.3 (s), 141.9 (s), 140.5 (s), 140.0 (s), 138.7 (s), 137.7 (s), 137.6 (s), 136.6 (s), 135.6 (br s, BAr^F$_4$), 135.2 (s), 133.6 (s), 132.5 (s), 131.9 (s), 120.1 (br quart, $^2J_{CF}$ = 31 Hz, BAr^F$_4$), 129.4 (s, NCH=*C*HN), 129.1 (s), 128.9 (s), 127.9 (s), 127.5 (s, NCH=*C*HN), 126.2 (s), 126.1 (s), 125.8 (s), 125.6 (quart, $^1J_{CF}$ = 272 Hz, BAr^F$_4$), 123.6 (s, NCH=*C*HN), 118.1 (br s, BAr^F$_4$), 87.6 (s, *C*(Me)=CH$_2$), 64.9 (s, C(Me)=*C*H$_2$), 30.4 (s, *C*HMe$_2$), 30.0 (s, *C*HMe$_2$),

29.8 (s, *C*HMe$_2$), 29.0 (s, *C*HMe$_2$), 28.8 (s, *C*HMe$_2$), 27.0 (s, CH*Me*$_2$), 26.5 (s, CH*Me*$_2$), 26.1 (s, CH*Me*$_2$), 26.0 (s, CH*Me*$_2$), 24.4 (s, CH*Me*$_2$), 24.1 (s, CH*Me*$_2$), 24.0 (s, CH*Me*$_2$), 23.3 (s, CH*Me*$_2$), 23.2 (s, CH*Me*$_2$), 23.1 (s, C(*Me*)=CH$_2$), 22.0 (s, CH*Me*$_2$). Anal. Calcd for $C_{92}H_{88}BN_4F_{24}ZnIr$ (1974.07): C, 55.97; H, 4.50; N, 2.84. Found: C, 56.07; H, 4.32; N, 2.82.

[Ir(IPr)(IPr″)(ZnMe)H][BAr$^F{}_4$] (4b). A solution of **7** (30 mg, 0.0164 mmol) in C_6H_5F (1 mL) was treated with $ZnMe_2$ (15 µL of 1.2 M solution in toluene, 0.0180 mmol) and stirred at room temperature for 2 h. The resulting solution was reduced to dryness, the residue redissolved in C_6H_5F and layered with hexane to yield yellow crystals of **4b**. Yield: 21 mg (68%). ^{1}H NMR (THF-d_8, 500 MHz, 248 K): δ 7.84 (br s, 8H, BAr$^F{}_4$), 7.74 (d, $^3J_{HH}$ = 1.7 Hz, 1H, NC*H*=CHN), 7.72 (d, $^3J_{HH}$ = 2.0 Hz, 1H, NC*H*=CHN), 7.69 (d, $^3J_{HH}$ = 1.9 Hz, 1H, NC*H*=CHN), 7.67 (d, $^3J_{HH}$ = 1.9 Hz, 1H, NC*H*=CHN), 7.63 (br s, 4H, BAr$^F{}_4$), 7.54 (t, $^3J_{HH}$ = 8.0 Hz, 1H, Ar), 7.51-7.45 (m, 2H, Ar), 7.43-7.39 (m, 3H, Ar), 7.35-7.27 (m, 5H, Ar), 7.14 (dd, $^4J_{HH}$ = 2.7 Hz, $^3J_{HH}$ = 6.3 Hz, 1H, Ar), 3.78 (s, 1H, C(Me)=C*H*H), 3.16 (sept, $^3J_{HH}$ = 7.0 Hz, 1H, C*H*Me$_2$), 3.13 (s, 1H, C(Me)=C*H*H), 3.05 (sept, $^3J_{HH}$ = 7.0 Hz, 1H, C*H*Me$_2$), 2.78 (sept, $^3J_{HH}$ = 6.5 Hz, 1H, C*H*Me$_2$), 2.47 (sept, $^3J_{HH}$ = 7.0 Hz, 1H, C*H*Me$_2$), 2.39 (sept, $^3J_{HH}$ = 6.5 Hz, 1H, C*H*Me$_2$), 2.29 (sept, $^3J_{HH}$ = 7.0 Hz, 1H, C*H*Me$_2$), 2.15 (sept, $^3J_{HH}$ = 7.0 Hz, 1H, C*H*Me$_2$), 1.47 (d, $^3J_{HH}$ = 6.7 Hz, 3H, CH*Me*$_2$), 1.44 (d, $^3J_{HH}$ = 6.7 Hz, 3H, CH*Me*$_2$), 1.41 (s, 3H, C(*Me*)=CH$_2$), 1.30 (d, $^3J_{HH}$ = 6.7 Hz, 3H, CH*Me*$_2$), 1.26 (d, $^3J_{HH}$ = 6.9 Hz, 3H, CH*Me*$_2$), 1.23 (d, $^3J_{HH}$ = 6.7 Hz, 3H, CH*Me*$_2$), 1.09 (d, $^3J_{HH}$ = 6.4 Hz, 6H, CH*Me*$_2$), 1.03 (d, $^3J_{HH}$ = 6.7 Hz, 3H, CH*Me*$_2$), 0.99 (overlapping d, 6H, CH*Me*$_2$), 0.80 (d, $^3J_{HH}$ = 6.7 Hz, 3H, CH*Me*$_2$), 0.66 (d, $^3J_{HH}$ = 6.7 Hz, 3H, CH*Me*$_2$), 0.41 (d, $^3J_{HH}$ = 6.7 Hz, 3H, CH*Me*$_2$), 0.27 (d, $^3J_{HH}$ = 6.7 Hz, 3H, CH*Me*$_2$), −1.30 (s, 3H, Zn−*Me*), −9.32 (s, 1H, Ir−*H*). ^{13}C{^{1}H} NMR (THF-d_8, 126 MHz, 248 K): δ 176.9 (s, Ir−C_{IPr}), 170.3 (s, Ir−C_{IPr}), 163.0 (1:1:1:1 quart, $^1J_{CB}$ = 50 Hz, BAr$^F{}_4$), 148.0 (s), 147.4 (s), 147.0 (s), 146.5 (s), 145.8 (s), 145.2 (s), 140.5 (s), 139.6 (s), 138.9 (s), 137.6 (s), 137.0 (s), 135.7 (br s, BAr$^F{}_4$), 133.4 (s), 131.9 (s), 131.6 (s),

130.1 (br quart, $^{2}J_{CF}$ = 32 Hz, $BAr^F{}_4$), 129.6 (s), 128.9 (s, NCH=*C*HN), 128.2 (s, NCH=*C*HN), 127.4 (s), 127.1 (s, NCH=*C*HN), 126.7 (s), 126.3 (s), 126.0 (s), 125.8 (s), 125.7 (quart, $^{1}J_{CF}$ = 272 Hz, $BAr^F{}_4$), 125.3 (s), 123.9 (s, NCH=*C*HN), 118.5 (br s, $BAr^F{}_4$), 88.1 (s, *C*(Me)=CH_2), 66.2 (s, C(Me)=*C*H_2), 30.2 (s, *C*HMe_2), 30.1 (s, *C*HMe_2), 29.9 (s, *C*HMe_2), 29.7 (s, *C*HMe_2), 29.6 (s, *C*HMe_2), 29.1 (s, *C*HMe_2), 29.0 (s, *C*HMe_2), 27.2 (s, CH*Me*$_2$), 26.3 (s, CH*Me*$_2$), 26.1 (s, CH*Me*$_2$), 25.8 (s, CH*Me*$_2$), 25.6 (s, CH*Me*$_2$), 25.5 (s, CH*Me*$_2$), 25.3 (s, CH*Me*$_2$), 25.1 (s, CH*Me*$_2$), 24.3 (s, CH*Me*$_2$), 24.2 (s, CH*Me*$_2$), 23.6 (s, CH*Me*$_2$), 23.5 (s, C(*Me*)=CH_2), 23.2 (s, CH*Me*$_2$), 22.8 (s, CH*Me*$_2$), 22.6 (s, CH*Me*$_2$), −9.4 (s, Zn–*Me*). Anal. Calcd. For $C_{87}H_{86}BN_4F_{24}ZnIr$ (1912.00): C 54.65, H 4.53, N 2.93. Found: C 56.02, H 4.54, N 2.93. Repeated attempts at analysis consistently gave high %C. ESI-TOF MS: $[M]^+$ *m*/*z* = 1047.4892 (theoretical *m*/*z* = 1047.4829).

[Ir(IPr)(IPr″)(ZnEt)H][BAr$^F{}_4$] (4c). A solution of **7** (30 mg, 0.0164 mmol) in C_6H_5F (1 mL) was treated with $ZnEt_2$ (18 μL of 1.0 M solution in hexane, 0.0180 mmol) and stirred at room temperature for 2 h. The resulting solution was reduced to dryness, the residue redissolved in C_6H_5F and layered with hexane to yield yellow crystals of **4c**. Yield: 19 mg (59%). ^{1}H NMR (THF-d_8, 500 MHz, 248 K): δ 7.83 (br s, 8H, $BAr^F{}_4$), 7.78 (br d, $^{3}J_{HH}$ = 2.0 Hz, 1H, NC*H*=CHN), 7.71 (br s, 1H, NC*H*=CHN), 7.67 (br s, 1H, NC*H*=CHN), 7.64 (br s, 1H, NC*H*=CHN),* 7.63 (br s, 4H, $BAr^F{}_4$), 7.58-7.48 (m, 3H, Ar), 7.44 (br d, $^{3}J_{HH}$ = 8.2 Hz, 1H, Ar), 7.41 (d, $^{3}J_{HH}$ = 7.8 Hz, 1H, Ar), 7.38-7.32 (m, 3H, Ar), 7.31-7.24 (m, 3H, Ar), 7.06 (br d, $^{3}J_{HH}$ = 7.4 Hz, 1H, Ar), 3.82 (s, 1H, C(Me)=C*H*H), 3.22 (sept, $^{3}J_{HH}$ = 6.5 Hz, 1H, C*H*Me_2), 3.12 (sept, $^{3}J_{HH}$ = 6.5 Hz, 1H, C*H*Me_2), 3.08 (s, 1H, C(Me)=C*H*H), 2.87 (sept, $^{3}J_{HH}$ = 6.6 Hz, 1H, C*H*Me_2), 2.61 (sept, $^{3}J_{HH}$ = 6.6 Hz, 1H, C*H*Me_2), 2.43 (sept, $^{3}J_{HH}$ = 6.6 Hz, 1H, C*H*Me_2), 2.38 (sept, $^{3}J_{HH}$ = 6.6 Hz, 1H, C*H*Me_2), 2.19 (sept, $^{3}J_{HH}$ = 6.6 Hz, 1H, C*H*Me_2), 1.44 (d, $^{3}J_{HH}$ = 6.8 Hz, 3H, CH*Me*$_2$), 1.42 (d, $^{3}J_{HH}$ = 6.4 Hz, 3H, CH*Me*$_2$), 1.41 (s, 3H, C(*Me*)=CH_2), 1.34 (d, $^{3}J_{HH}$ = 6.8 Hz, 3H, CH*Me*$_2$), 1.31 (d, $^{3}J_{HH}$ = 7.0 Hz, 3H, CH*Me*$_2$), 1.28

(d, $^3J_{HH}$ = 6.6 Hz, 3H, CH*Me*$_2$), 1.23 (d, $^3J_{HH}$ = 6.6 Hz, 3H, CH*Me*$_2$), 1.16 (d, $^3J_{HH}$ = 6.6 Hz, 3H, CH*Me*$_2$), 1.04-0.93 (m, 12H, CH*Me*$_2$), 0.81 (d, $^3J_{HH}$ = 6.7 Hz, 3H, CH*Me*$_2$), 0.48 (t, $^3J_{HH}$ = 7.8 Hz, 3H, ZnCH$_2$*Me*), 0.39 (d, $^3J_{HH}$ = 6.9 Hz, 3H, CH*Me*$_2$), 0.13 (d, $^3J_{HH}$ = 6.8 Hz, 3H, CH*Me*$_2$), –0.50 (dquart, $^2J_{HH}$ = 12.7 Hz, $^3J_{HH}$ = 7.8 Hz, 1H, Zn–C*H*HMe), –0.58 (dquart, $^2J_{HH}$ = 12.7 Hz, $^3J_{HH}$ = 7.8 Hz, 1H, Zn–CH*H*Me), –8.06 (s, 1H, Ir–*H*). *Resonance partially obscured by BAr$^F_4$. ^{13}C{^{1}H} NMR (THF-*d*$_8$, 101 MHz, 248 K): δ 176.1 (s, Ir–C_{IPr}), 170.9 (s, Ir–C_{IPr}), 163.0 (1:1:1:1 quart, $^1J_{CB}$ = 50 Hz, BAr$^F_4$), 148.3 (s), 147.4 (s), 146.8 (s), 145.4 (s), 145.3 (s), 140.6 (s), 139.9 (s), 138.7 (s), 137.7 (s), 136.8 (s), 135.6 (br s, BAr$^F_4$), 135.4 (s), 133.2 (s), 132.2 (s), 131.8 (s), 130.1 (br quart, $^2J_{CF}$ = 31 Hz, BAr$^F_4$), 129.4 (s), 128.9 (s, NCH=*C*HN), 127.9 (s), 127.4 (s), 126.9 (s), 126.0 (s), 125.8 (s, NCH=*C*HN), 125.7 (s), 125.6 (quart, $^1J_{CF}$ = 272 Hz, BAr$^F_4$), 125.5 (s), 124.5 (s), 123.5 (s, NCH=*C*HN), 118.4 (br s, BAr$^F_4$), 86.5 (s, *C*(Me)=CH$_2$), 64.6 (s, C(Me)=*C*H$_2$), 30.1 (s, *C*HMe$_2$), 30.0 (s, *C*HMe$_2$), 29.8 (s, *C*HMe$_2$), 29.6 (s, *C*HMe$_2$), 29.0 (s, *C*HMe$_2$), 27.0 (s, CH*Me*$_2$), 26.3 (s, CH*Me*$_2$), 26.2 (s, CH*Me*$_2$), 26.0 (s, CH*Me*$_2$), 25.8 (s, CH*Me*$_2$), 24.3 (s, CH*Me*$_2$), 24.1 (s, CH*Me*$_2$), 23.7 (s, CH*Me*$_2$), 23.6 (s, CH*Me*$_2$), 23.5 (s, CH*Me*$_2$), 23.1 (s, CH*Me*$_2$), 22.9 (s, C(*Me*)=CH$_2$), 11.8 (s, Zn–*C*H$_2$Me), 4.5 (s, Zn–CH$_2$*Me*). Anal. Calcd. For $C_{88}H_{88}BN_4F_{24}ZnIr$ (1926.02): C 54.87, H 4.61, N 2.91. Found: C 55.40, H 4.71, N 2.74.

[Ir(IPr)$_2$(ZnPh)$_2$H$_4$][BAr$^F_4$] (5). A J. Young's resealable ampule was charged with a C_6H_5F (2 mL) solution of **1** (60 mg, 0.033 mmol) and ZnPh$_2$ (38 mg, 0.18 mmol), degassed via three freeze-pump-thaw cycles and placed under 1 atm H$_2$, which resulted in a rapid change from yellow-orange to colorless due to formation of **6**. Upon heating at 80 °C for 15 h, a pale-pink solution formed, which was concentrated and layered with hexane to yield colorless crystals of **5**. Yield: 41 mg (60 %). ^{1}H NMR (THF-*d*$_8$, 500 MHz, 323 K): δ 7.77 (br s, 8H, BAr$^F_4$), 7.61 (t, $^3J_{HH}$ = 7.6 Hz, 4H, Ar), 7.55 (br s, 4H, BAr$^F_4$), 7.51 (s, 4H, NC*H*=CHN), 7.45 (d, $^3J_{HH}$ = 7.8 Hz, 9H, Ar), 7.24-7.17 (m, 7H, ZnPh),# 2.41 (br sept, $^3J_{HH}$ =

6.7 Hz, 8H, C*H*Me$_2$), 0.94 (d, $^3J_{HH}$ = 6.9 Hz, 24H, CH*Me*$_2$), 0.92 (d, $^3J_{HH}$ = 6.9 Hz, 24H, CH*Me*$_2$), −10.29 (s, 4H, Ir–*H*). #Remaining aromatic protons obscured by residual C_6H_5F. $^{13}C\{^1H\}$ NMR (THF-d_8, 101 MHz, 323 K): δ 163.1 (1:1:1:1 quart, $^1J_{CB}$ = 50 Hz, BAr^F_4), 157.1 (s), 154.6 (s, Ir–C_{IPr}), 146.5 (s), 138.9 (s), 137.7 (s), 135.8 (br s, BAr^F_4), 132.7 (s), 130.2 (br quart, $^2J_{CF}$ = 31 Hz, BAr^F_4), 129.1 (s), 128.5 (s), 127.3 (s, NCH=*C*HN), 127.1 (s), 125.7 (quart, $^1J_{CF}$ = 270 Hz, BAr^F_4), 118.3 (br s, BAr^F_4), 30.2 (s, *C*HMe$_2$), 25.8 (s, CH*Me*$_2$), 23.4 (s, CH*Me*$_2$). ATR-IR (cm^{-1}): 1738 (ν_{Ir-H}). Anal. Calcd. For $C_{98}H_{98}BNF_{24}Zn_2Ir$ (2121.62): C 55.48, H 4.66, N 2.64. Found: C 52.79, H 4.37, N 2.50. Repeated attempts at consistently gave low %C. ESI-TOF MS: $[M]^+$ *m*/*z* = 1255.5043 (theoretical *m*/*z* = 1255.5059).

[Ir(IPr)(IPr″)H$_2$][BAr$^F_4$] (7). A C_6H_5F (2 mL) solution of **1** (200 mg, 0.109 mmol) was treated with $Me_3SiCH=CH_2$ (47 µL, 0.321 mmol) and stirred at 80 °C for 3 h. The resulting solution was reduced to dryness, the residue redissolved in C_6H_5F and layered with hexane to yield yellow crystals of **7**. Yield: 147 mg (73%). ^1H NMR (THF-d_8, 500 MHz, 318 K): δ 7.78 (br s, 8H, BAr^F_4), 7.73 (d, $^3J_{HH}$ = 1.9 Hz, 1H, NC*H*=CHN), 7.56-7.54 (m, 7H, BAr^F_4 + NC*H*=CHN), 7.46-7.40 (m, 2H, Ar), 7.33-7.28 (m, 3H, Ar),* 7.19 (br d, $^3J_{HH}$ = 7.6 Hz, 2H, Ar), 7.12 (m, 1H, Ar),* 7.03-6.96 (m, 3H, Ar),* 6.64 (dd, J_{HH} = 7.4 Hz, J_{HH} = 1.4 Hz, 1H, Ar), 3.51 (s, 1H, C(Me)=C*H*H), 3.30 (s, 1H, C(Me)=CH*H*), 3.26 (sept, $^3J_{HH}$ = 6.8 Hz, 1H, C*H*Me$_2$), 2.76 (sept, $^3J_{HH}$ = 6.7 Hz, 2H, C*H*Me$_2$), 2.53 (sept, $^3J_{HH}$ = 6.7 Hz, 2H, C*H*Me$_2$), 2.40 (sept, $^3J_{HH}$ = 6.8 Hz, 1H, C*H*Me$_2$), 2.28 (sept, $^3J_{HH}$ = 7.1 Hz, 1H, C*H*Me$_2$), 1.65 (s, 3H, C(*Me*)=CHH), 1.43 (d, $^3J_{HH}$ = 6.8 Hz, 3H, CH*Me*$_2$), 1.28 (d, $^3J_{HH}$ = 6.9 Hz, 3H, CH*Me*$_2$), 1.17-1.09 (m, 18H, CH*Me*$_2$), 1.00 (br d, $^3J_{HH}$ = 6.7 Hz, 6H, CH*Me*$_2$), 0.97 (d, $^3J_{HH}$ = 6.9 Hz, 6H, CH*Me*$_2$), 0.90 (d, $^3J_{HH}$ = 6.9 Hz, 3H, CH*Me*$_2$), 0.28 (d, $^3J_{HH}$ = 6.9 Hz, 3H, CH*Me*$_2$), −13.63 (d, $^2J_{HH}$ = 8.2 Hz, 1H, Ir–*H*), −41.24 (d, $^2J_{HH}$ = 8.2 Hz, 1H, Ir–*H*). *Resonances partially overlap with residual C_6H_5F and prevent integration of aromatic signals. $^{13}C\{^1H\}$ NMR (THF-d_8, 126 MHz, 318 K): δ 173.2 (s, Ir–C_{IPr}), 169.3 (s, Ir–C_{IPr}), 163.0 (1:1:1:1 quart,

$^1J_{CB}$ = 50 Hz, BAr$^F{}_4$), 146.4 (s), 146.3 (s), 146.1 (s), 145.2 (s), 141.5 (s), 138.2 (s), 137.2 (s), 135.8 (br s, BAr$^F{}_4$), 135.7 (s), 134.5 (s), 131.7 (s), 131.3 (s), 130.5 (s), 130.2 (br quart, $^2J_{CF}$ = 31 Hz, BAr$^F{}_4$), 129.4 (s), 128.9 (s, NCH=*C*HN), 127.8 (s), 126.2 (s), 125.8 (s, NCH=*C*HN), 125.7 (quart, $^1J_{CF}$ = 272 Hz, BAr$^F{}_4$), 125.0 (s), 124.6 (s), 123.2 (s, NCH=*C*HN), 118.3 (br s, BAr$^F{}_4$), 97.0 (s, *C*(Me)=CH$_2$),* 29.9 (s, *C*HMe$_2$), 29.7 (s, *C*HMe$_2$), 28.9 (s, *C*HMe$_2$), 28.7 (s, *C*HMe$_2$), 26.6 (s, CH*Me*$_2$), 26.0 (s, CH*Me*$_2$), 25.8 (s, CH*Me*$_2$), 25.0 (s, C(*Me*)=CH$_2$), 24.6 (s, CH*Me*$_2$), 24.5 (s, CH*Me*$_2$), 24.2 (s, CH*Me*$_2$), 23.8 (s, CH*Me*$_2$), 23.2 (s, CH*Me*$_2$). *A resonance at δ 67.2 for Ir–C(Me)=*C*H$_2$, obscured by THF, was identified by ^{13}C-^{1}H HSQC. Anal. Calcd. For $C_{86}H_{84}BNF_{24}Ir$ (1832.6): C 56.36, H 4.62, N 3.06. Found: C 56.02, H 4.54, N 2.93.

[Ir(IPr)(IPr″)(CdMe)H][BAr$^F{}_4$] (8). CdMe$_2$ (9.5 µL of 2.4 M solution in toluene, 0.0228 mmol) was added to a C_6H_5F (1 mL) solution of **7** (40 mg, 0.0219 mmol) and the reaction followed by NMR spectroscopy. Within ca. 5 min, **8** was present, but as the minor species alongside another hydride containing product at δ −6.55 (ratio of hydride signals 1:1.41).[7] After 4 h, the ratio was 1:0.26. The solution was then reduced to dryness, the residue redissolved in C_6H_5F and layered with hexane to yield yellow crystals comprized overwhelmingly of **8** (ratio of hydride signals 1:0.08). Yield: 29 mg (68%). ^{1}H NMR of **8** (THF-d_8, 400 MHz, 248 K): δ 7.85 (br s, 8H, BAr$^F{}_4$), 7.73 (br m, 1H, NC*H*=CHN), 7.68-7.63 (m, 7H, NC*H*=CHN and BAr$^F{}_4$), 7.57-7.26 (m, 13H, Ar), 7.20-7.08 (m, 4H, Ar), 3.87 (s, 1H, C(Me)=C*H*H),‡ 3.23-2.97 (overlapping m, 3H, C*H*Me$_2$ + C(Me)=C*H*H), 2.87 (sept, $^3J_{HH}$ = 6.6 Hz, 1H, C*H*Me$_2$), 2.50 (sept, $^3J_{HH}$ = 6.7 Hz, 1H, C*H*Me$_2$), 2.39 (sept, $^3J_{HH}$ = 6.1 Hz, 1H, C*H*Me$_2$), 2.30 (sept, $^3J_{HH}$ = 6.7 Hz, 1H, C*H*Me$_2$), 2.22 (sept, $^3J_{HH}$ = 6.7 Hz, 1H, C*H*Me$_2$), 1.52-1.39 (overlapping m, 9H, C(*Me*)=CH$_2$ + CH*Me*$_2$), 1.33-1.22 (m, 9H, CH*Me*$_2$), 1.14-0.94 (m, 15H, CH*Me*$_2$), 0.81 (d, $^3J_{HH}$ = 6.7 Hz, 3H, CH*Me*$_2$), 0.77 (d, $^3J_{HH}$ = 6.9 Hz, 3H, CH*Me*$_2$), 0.40 (d, $^3J_{HH}$ = 6.5 Hz, CH*Me*$_2$), 0.21 (d, $^3J_{HH}$ = 6.5 Hz, 3H, CH*Me*$_2$), –0.86 (s + sat ($^2J_{HCd}$ = 67 Hz), 3H, Cd–*Me*), −9.47 (s, 1H, Ir–*H*). ‡Accurate integration of aromatic region

complicated by the presence of the proposed second, minor isomer (selected resonances: δ −0.97 (s + sat ($^2J_{HCd}$ = 69 Hz), Cd–*Me*), −6.37 (s, Ir–*H*)). $^{13}C\{^1H\}$ NMR (THF-d_8, 101 MHz, 248 K): δ 176.5 (s, Ir–C_{IPr}), 170.3 (s, Ir–C_{IPr}), 163.0 (1:1:1:1 quart, $^1J_{CB}$ = 50 Hz, BAr^F_4), 148.0 (s), 147.0 (s), 146.6 (s), 145.7 (s), 145.2 (s), 140.3 (s), 139.9 (s), 138.9 (s), 147.6 (s), 137.3 (s), 135.6 (br s, BAr^F_4), 133.1 (s), 132.0 (s), 131.6 (s), 130.1 (br quart, $^2J_{CF}$ = 31 Hz, BAr^F_4), 129.6 (s), 129.3 (s), 128.7 (s), 128.3 (s), 128.1 (s), 127.1 (s), 126.2 (s), 125.9 (s), 125.7 (s), 125.6 (quart, $^1J_{CF}$ = 272 Hz, BAr^F_4), 125.3 (s), 123.7 (s), 118.5 (br s, BAr^F_4), 116.2 (s), 116.0 (s), 86.7 (s, *C*(Me)=CH_2), 65.5 (s, C(Me)=*C*H_2), 30.5 (s), 30.1 (s), 29.9 (s), 29.6 (s), 29.1 (s), 29.0 (s), 27.1 (s), 26.3 (s), 26.2 (s), 25.2 (s), 24.4 (s), 24.1 (s), 23.7 (s), 23.2 (s), 22.6 (s), −8.3 (s + sat ($^1J_{C\text{–}113Cd}$ = 724 Hz, $^1J_{C\text{–}111Cd}$ = 694 Hz), Cd–*Me*). Minor isomer: δ −5.34 (s; $^1J_{CCd}$ satellites not observable; Cd–*Me*).

[Ir(IPr)(IPr″)(ZnPh)H_3][BAr^F_4] (9a). A degassed (freeze-pump-thaw x 3) THF-d_8 (0.5 mL) solution of **4a** (20 mg, 0.010 mmol) in a J. Youngs resealable NMR tube was placed under 1 atm H_2 (resulting in a rapid color change from yellow-orange to colorless) before being quickly introduced into a pre-cooled NMR probe at 248 K for ^1H NMR analysis. ^1H NMR (THF-d_8, 500 MHz, 248 K): δ 7.91 (br m, 1H, NC*H*=CHN), 7.83 (br s, 8H, BAr^F_4), 7.81 (br s, 1H, NC*H*=CHN),* 7.74 (br m, 2H, NC*H*=CHN), 7.63 (br s, 4H, BAr^F_4), 7.55 (t, J_{HH} = 7.7 Hz, 1H, Ar), 7.49-7.31 (m, 7H, Ar), 7.29-7.19 (m, 2H, Ar), 7.18-7.07 (m, 5H, Ar), 6.87 (br m, 2H, Ar), 4.47 (s, 1H, C(Me)=C*H*H), 3.60 (s, 1H, C(Me)=C*H*H),# 3.34 (sept, $^3J_{HH}$ = 6.6 Hz, 1H, C*H*Me_2), 3.01 (sept, $^3J_{HH}$ = 6.6 Hz, 1H, C*H*Me_2), 2.85 (m, 1H, C*H*Me_2), 2.54 (sept, $^3J_{HH}$ = 6.6 Hz, 1H, C*H*Me_2), 2.23 (m, 2H, C*H*Me_2), 2.07 (sept, $^3J_{HH}$ = 6.8 Hz, 1H, C*H*Me_2), 1.69 (s, 3H, C(*Me*)=CH_2),# 1.46 (app t, J_{HH} = 7.0 Hz, 6H, CHMe_2), 1.32 (m, 6H, CHMe_2), 1.17 (d, $^3J_{HH}$ = 6.6 Hz, 3H, CHMe_2), 1.05 (d, $^3J_{HH}$ = 6.9 Hz, 3H, CHMe_2), 0.97-0.87 (m, 6H, CHMe_2), 0.77 (app t, $^3J_{HH}$ = 6.6 Hz, 6H, CHMe_2), 0.66 (m, 6H, CHMe_2), 0.51 (d, $^3J_{HH}$ = 6.6 Hz, 3H, CHMe_2), 0.28 (d, $^3J_{HH}$ = 6.6 Hz, 3H, CHMe_2), −9.37 (d, $^2J_{HH}$ = 15.5 Hz,

1H, Ir–*H*), –12.21 (s, 1H, Ir–*H*), –13.82 (d, $^2J_{HH}$ = 15.5 Hz, 1H, Ir–*H*). *Signal partially obscured by BAr^F_4. #Resonances partially obscured by THF.

[Ir(IPr)(IPr″)(ZnMe)H_3][BAr^F_4] (9b). A sample of **4b** (20 mg, 0.010 mmol) in THF-d_8 (0.5 mL) in a J. Youngs resealable NMR tube was freeze-pump-thaw degassed (x3) and placed under 1 atm H_2 to give a colorless solution. A ^{1}H NMR spectrum recorded after 5 min showed the presence of **9b** alongside unreacted **4b**, [Ir(IPr_2)(ZnMe)(η^2-H_2)H_3][BAr^F_4] (**11b**) and Ir$(IPr)_2H_5$ (**13**). After 1 h, **11b** was the major product in solution.

A spectroscopically clean sample of **9b** was prepared by placing a degassed (freeze-pump-thaw x 3) THF-d_8 (0.5 mL) solution of **4b** (20 mg, 0.010 mmol) in a J. Youngs resealable NMR tube under 1 atm H_2 followed by rapid insertion into a pre-cooled NMR probe at 228 K for ^{1}H NMR analysis. ^{1}H NMR (THF-d_8, 400 MHz, 228 K): δ 7.86 (br s, 8H, BAr^F_4), 7.81 (br s, 2H), 7.67 (br s, 4H, BAr^F_4), 7.57-7.51 (br m, 2H), 7.50-7.28 (br m, 7H), 7.25-7.10 (br m, 5H), 4.29 (s, 1H, C(Me)=C*H*H), 3.54 (s, 1H, C(Me)=C*H*H), 3.06 (sept, $^3J_{HH}$ = 6.3 Hz, 1H, C*H*Me$_2$), 3.00 (sept, $^3J_{HH}$ = 6.5 Hz, 1H, C*H*Me$_2$), 2.70 (sept, $^3J_{HH}$ = 6.1 Hz, 1H, C*H*Me$_2$), 2.36 (sept, $^3J_{HH}$ = 6.4 Hz, 1H, C*H*Me$_2$), 2.29 (sept, $^3J_{HH}$ = 6.3 Hz, 1H, C*H*Me$_2$), 1.86 (sept, $^3J_{HH}$ = 6.1 Hz, 1H, C*H*Me$_2$), 2.21 (sept, $^3J_{HH}$ = 6.1 Hz, 1H, C*H*Me$_2$), 1.62 (br s, 3H, C(*Me*)=CH$_2$), 1.46 (m, 6H, CH*Me$_2$*), 1.31 (d, $^3J_{HH}$ = 6.1 Hz, 3H, CH*Me$_2$*), 1.25 (d, $^3J_{HH}$ = 6.7 Hz, 3H, CH*Me$_2$*), 1.22 (d, $^3J_{HH}$ = 6.3 Hz, 3H, CH*Me$_2$*), 1.09 (d, $^3J_{HH}$ = 6.2 Hz, 3H, CH*Me$_2$*), 1.02 (d, $^3J_{HH}$ = 6.4 Hz, 3H, CH*Me$_2$*), 0.99 (d, $^3J_{HH}$ = 6.7 Hz, 3H, CH*Me$_2$*), 0.95 (m, 6H, CH*Me$_2$*), 0.74 (m, 6H, CH*Me$_2$*), 0.58 (d, $^3J_{HH}$ = 6.2 Hz, 3H, CH*Me$_2$*), 0.26 (d, $^3J_{HH}$ = 6.2 Hz, 3H, CH*Me$_2$*), –1.27 (s, 3H, Zn–*Me*), –9.51 (d, $^2J_{HH}$ = 16.7 Hz, 1H, Ir–*H*), –12.52 (s, 1H, Ir–*H*), –14.13 (d, $^2J_{HH}$ = 16.7 Hz, 1H, Ir–*H*).

[Ir(IPr)(IPr″)(ZnEt)H_3][BAr^F_4] (9c). A spectroscopically clean sample of **9c** was prepared through addition of 1 atm H_2 to a degassed (freeze-pump-thaw x 3) THF-d_8 (0.5 mL) solution of **4c** (20 mg, 0.010 mmol) in a J. Youngs resealable NMR tube, followed by

rapid insertion into a pre-cooled NMR probe at 248 K for ^{1}H NMR analysis. ^{1}H NMR (THF-d_8, 400 MHz, 248 K): δ 7.84 (br s, 8H, BAr$^F{}_4$), 7.78 (br s, 1H, NC*H*=CHN),* 7.77 (br s, 1H, NC*H*=CHN), 7.71 (br s, 1H, NC*H*=CHN), 7.68 (br s, 1H, NC*H*=CHN), 7.64 (br s, 4H, BAr$^F{}_4$), 7.54-7.51 (m, 2H, Ar), 7.50-7.28 (m, 6H, Ar), 7.24-7.06 (m, 4H, Ar), 4.31 (s, 1H, C(Me)=C*H*H), 3.52 (s, 1H, C(Me)=C*H*H), 3.16 (sept, $^3J_{HH}$ = 6.5 Hz, 1H, C*H*Me$_2$), 2.98 (sept, $^3J_{HH}$ = 6.4 Hz, 1H, C*H*Me$_2$), 2.76 (sept, $^3J_{HH}$ = 6.8 Hz, 1H, C*H*Me$_2$), 2.26 (m, 2H, C*H*Me$_2$), 1.96 (sept, $^3J_{HH}$ = 6.6 Hz, 1H, C*H*Me$_2$), 1.64 (br s, 3H, C(*Me*)=CH$_2$), 1.46 (app t, $^3J_{HH}$ = 67.1 Hz, 6H, CH*Me*$_2$), 1.34-1.25 (m, 9H, CH*Me*$_2$), 1.13 (app t, $^3J_{HH}$ = 6.1 Hz, 6H, CH*Me*$_2$), 1.00 (br d, $^3J_{HH}$ = 6.4 Hz, 3H, CH*Me*$_2$), 0.93 (d, $^3J_{HH}$ = 6.7 Hz, 3H, CH*Me*$_2$), 0.86-0.71 (m, 9H, CH*Me*$_2$), 0.52 (d, $^3J_{HH}$ = 6.5 Hz, 3H, CH*Me*$_2$), 0.27 (d, $^3J_{HH}$ = 6.5 Hz, 3H, CH*Me*$_2$), −0.32 (dquart, $^2J_{HH}$ = 13.7 Hz, $^3J_{HH}$ = 8.0 Hz, 1H, Zn–C*H*HMe), −0.51 (dquart, $^2J_{HH}$ = 13.7 Hz, $^3J_{HH}$ = 8.0 Hz, 1H, Zn–CH*H*Me), −9.62 (d, $^2J_{HH}$ = 16.0 Hz, 1H, Ir–*H*), −12.41 (s, 1H, Ir–*H*), −14.16 (d, $^2J_{HH}$ = 16.0 Hz, 1H, Ir–*H*).

[Ir(IPr)(IPr″)(CdMe)H$_3$][BAr$^F{}_4$] (10). A J Youngs resealable NMR tube containing a THF-d_8 (0.5 mL) solution of **8** (20 mg, 0.0102 mmol) was degassed by three freeze-pump-thaw cycles, placed under 1 atm H$_2$ (resulting in a color change from yellow-orange to colorless) and then quickly introduced into a pre-cooled NMR probe at 248 K for ^{1}H NMR analysis. ^{1}H NMR (THF-d_8, 400 MHz, 248 K): δ 7.85 (br s, 8H, BAr$^F{}_4$), 7.80 (br s, 1H, NC*H*=CHN), 7.77 (s, 1H, NC*H*=CHN), 7.70 (s, 1H, NC*H*=CHN), 7.65 (overlapping m, 5H, NC*H*=CHN + BAr$^F{}_4$), 7.56-7.42 (m, 3H, Ar), 7.42-7.26 (m, 8H, Ar), 7.22 (d, $^3J_{HH}$ = 7.0 Hz, 1H, Ar), 4.31 (s, 1H, C(Me)=C*H*H), 3.54 (s, 1H, C(Me)=C*H*H), 3.05 (sept, $^3J_{HH}$ = 6.2 Hz, 1H, C*H*Me$_2$), 2.99 (sept, $^3J_{HH}$ = 6.3 Hz, 1H, C*H*Me$_2$), 2.77 (sept, $^3J_{HH}$ = 6.8 Hz, 1H, C*H*Me$_2$), 2.46 (sept, $^3J_{HH}$ = 6.8 Hz, 1H, C*H*Me$_2$), 2.30 (sept, $^3J_{HH}$ = 6.3 Hz, 1H, C*H*Me$_2$), 2.20 (sept, $^3J_{HH}$ = 6.3 Hz, 1H, C*H*Me$_2$), 1.96 (sept, $^3J_{HH}$ = 6.3 Hz, 1H, C*H*Me$_2$), 1.63 (s, 3H, C(*Me*)=CH$_2$) 1.50-1.39 (m, 6H, CH*Me*$_2$), 1.36-1.19 (m, 9H, CH*Me*$_2$), 1.12 (d, $^3J_{HH}$ = 6.0 Hz,

3H, $CHMe_2$), 1.06-0.91 (m, 12H, $CHMe_2$), 0.86 (d, $^3J_{HH}$ = 6.0 Hz, 3H, $CHMe_2$), 0.75 (d, $^3J_{HH}$ = 6.0 Hz, 3H, $CHMe_2$), 0.56 (d, $^3J_{HH}$ = 6.4 Hz, $CHMe_2$), 0.39 (d, $^3J_{HH}$ = 6.0 Hz, 3H, $CHMe_2$), –0.86 (s + sat ($^2J_{HCd}$ = 67 Hz), 3H, Cd–*Me*), –8.24 (d + sat, $^2J_{HH}$ = 10.5 Hz ($^2J_{HCd}$ = 628 Hz), 1H, Ir–*H*), –12.08 (s + sat ($^2J_{HCd}$ = 42 Hz), 1H, Ir–*H*), –14.02 (d + sat, $^2J_{HH}$ = 10.5 Hz ($^2J_{HCd}$ = 114 Hz), 1H, Ir–*H*).

[Ir(IPr)$_2$(ZnMe)(η^2-H$_2$)H$_3$][BAr$^F{}_4$] (11b). A solution of **9b** (60 mg, 0.0164 mmol) in C_6H_5F (3 mL) was degassed via three freeze-pump-thaw cycles and exposed to 1 atm H_2, resulting in a rapid change from yellow-orange to colorless. After stirring for 3 h at room temperature, the solution was reduced to dryness and the residue dissolved in C_6H_5F and layered with hexane to yield **11b** as orange crystals. Yield: 19 mg (59%). ^{1}H NMR (THF, 500 MHz, 226 K): δ 7.86 (br s, 8H, BAr$^F{}_4$), 7.66 (m, 8H, NC*H*=CHN + BAr$^F{}_4$), 7.56 (t, $^3J_{HH}$ = 7.7 Hz, 4H, Ar), 7.39 (d, $^3J_{HH}$ = 8.0 Hz, 8H, Ar), 2.15 (sept, $^3J_{HH}$ = 6.8 Hz, 8H, C*H*Me$_2$), 1.07 (d, $^3J_{HH}$ = 6.6 Hz, 24H, $CHMe_2$), 0.99 (d, $^3J_{HH}$ = 6.6 Hz, 24H, $CHMe_2$), –0.67 (s, 3H, Zn–*Me*), –8.95 (s, 5H, Ir–*H*). ^{13}C{^{1}H} NMR (THF, 101 MHz, 226 K): δ 162.7 (1:1:1:1 quart, $^1J_{CB}$ = 51 Hz, BAr$^F{}_4$), 154.1 (s, Ir–C_{IPr}), 145.4 (s, C_{Ar}), 138.2 (s, C_{Ar}), 135.2 (br s, BAr$^F{}_4$), 131.5 (s, C_{Ar}), 129.7 (br quart, $^2J_{CF}$ = 33 Hz, BAr$^F{}_4$), 126.0 (m, C_{Ar} + NCH=*C*HN), 125.2 (quart, $^1J_{CF}$ = 272 Hz, BAr$^F{}_4$), 118.1 (br s, BAr$^F{}_4$), 29.6 (s, *C*HMe$_2$) 25.5 (s, $CHMe_2$), 22.8 (s, $CHMe_2$), 4.55 (s, Zn–*Me*). Anal. Calcd. For $C_{87}H_{92}BN_4F_{24}ZnIr$ (1918.07): C 54.48, H 4.83, N 2.92. Found: C 54.59, H 4.89, N 2.76.

[Ir(IPr)$_2$(ZnEt)(η^2-H$_2$)H$_3$][BAr$^F{}_4$] (11c). As for **11b** but using **9c** (60 mg, 0.0164 mmol) to yield orange crystals of **11c**. Yield: 36 mg (61%). ^{1}H NMR (THF, 500 MHz, 226 K): δ 7.86 (br s, 8H, BAr$^F{}_4$), 7.66 (m, 8H, NC*H*=CHN + BAr$^F{}_4$), 7.55 (t, $^3J_{HH}$ = 7.6 Hz, 4H, Ar), 7.39 (d, $^3J_{HH}$ = 7.7 Hz, 8H, Ar), 7.40 (d, $^3J_{HH}$ = 7.8 Hz, 1H, Ar), 2.13 (sept, $^3J_{HH}$ = 6.7 Hz, 8H, C*H*Me$_2$), 1.09 (d, $^3J_{HH}$ = 6.6 Hz, 24H, $CHMe_2$), 1.00 (m, 27H, $CHMe_2$ + Zn–CH$_2$*Me*), 0.03 (quart, $^3J_{HH}$ = 8.0 Hz, 2H, Zn–*C*H$_2$Me), –9.00 (s, 5H, Ir–*H*). ^{13}C{^{1}H}

NMR (THF, 101 MHz, 226 K): δ 163.0 (1:1:1:1 quart, $^{1}J_{CB}$ = 50 Hz, BAr^F_4), 154.7 (s, Ir–C_{IPr}), 145.6 (s, C_{Ar}), 138.5 (s, C_{Ar}), 135.6 (br s, BAr^F_4), 131.8 (s, C_{Ar}), 130.1 (br quart, $^{2}J_{CF}$ = 33 Hz, BAr^F_4), 126.4 (m, C_{Ar} + NCH=*C*HN), 125.6 (quart, $^{1}J_{CF}$ = 272 Hz, BAr^F_4), 118.5 (br s, BAr^F_4), 30.0 (s, *C*HMe$_2$) 25.9 (s, CH*Me*$_2$), 23.0 (s, CH*Me*$_2$), 18.5 (s, Zn–*C*H$_2$Me), 10.9 (s, Zn–CH$_2$*Me*). ATR-IR (cm^{-1}): 1662 (ν_{Ir-H}). Anal. Calcd. For $C_{88}H_{94}BN_4F_{24}ZnIr \cdot C_6H_5F$ (2028.17): C 55.66, H 4.92, N 2.76. Found: C 56.03, H 4.90, N 2.90.

[Ir(IPr)$_2$(CdMe)(η^2-H$_2$)H$_3$][BAr$^F_4$] (12). A solution of **8** (20 mg, 0.0102 mmol) in C_6H_5F (0.5 mL) was degassed via three freeze-pump-thaw cycles and exposed to 1 atm H_2. After stirring for 3 h at room temperature, the solution was reduced to dryness, the residue redissolved in fluorobenzene and layered with hexane to yield **12** as orange crystals. Yield: 12 mg (60 %). ^{1}H NMR (C_6D_5F, 500 MHz, 298 K): δ 8.55 (br s, 8H, BAr^F_4), 7.85 (br s, 4H, BAr^F_4), 7.55 (t, $^{3}J_{HH}$ = 7.9 Hz, 4H, Ar), 7.33 (d, $^{3}J_{HH}$ = 7.9 Hz, 8H, Ar), 6.91 (s, 4H, NC*H*=CHN), 2.30 (sept, $^{3}J_{HH}$ = 6.7 Hz, 8H, C*H*Me$_2$), 1.20 (d, $^{3}J_{HH}$ = 6.7 Hz, 24H, CH*Me*$_2$), 1.07 (d, $^{3}J_{HH}$ = 6.7 Hz, 24H, CH*Me*$_2$), –0.09 (s + sat ($^{2}J_{HCd}$ = 73 Hz), 3H, Cd–*Me*), –8.23 (s + sat ($^{2}J_{HCd}$ = 158 Hz), 5H, Ir–*H*). ^{13}C{^{1}H} NMR (C_6D_5F, 126 MHz, 298 K): δ 162.7 (1:1:1:1 quart, $^{1}J_{CB}$ = 50 Hz, BAr^F_4), 155.2 (s, Ir–C_{IPr}), 145.0 (s), 137.5 (s), 135.3 (br s, BAr^F_4), 131.1 (s), 125.6 (s), 125.2 (quart, $^{1}J_{CF}$ = 273 Hz, BAr^F_4), 124.2 (s, N*C*H=CHN), 117.8 (br s, BAr^F_4), 29.0 (s, *C*HMe$_2$), 25.0 (s, CH*Me*$_2$), 22.4 (s, CH*Me*$_2$), 6.3 (s, Cd–*Me*). *BAr^F_4 resonance at ca. 130 ppm obscured by C_6D_5F. ^{113}Cd{^{1}H} NMR (C_6D_5F, 111 MHz, 298 K): δ –60.7 (s).

Solution and solid-state dehydrogenation of 11c. Two J. Young's resealable NMR tubes were prepared containing 10 mg (0.005 mmol) of **11c**. THF-*d*$_8$ (0.6 mL) and styrene (3 μL, 0.026 mmol) were added to one tube and the reaction monitored by ^{1}H NMR spectroscopy at room temperature. The second sample was placed under an active vacuum and heated at 60 °C. Periodically, it was redissolved in THF-*d*$_8$ and the dehydrogenation of the starting material analyzed by ^{1}H NMR spectroscopy.

Reactivity of 11c with NHCs. 11c (15 mg, 0.008 mmol) was combined with IMe_4 (2 mg, 0.016 mmol) and IMes (4.6 mg, 0.015 mmol) in C_6H_5F (0.6 mL) in two separate J. Youngs NMR tubes and the reactions monitored by ^1H NMR spectroscopy.

Preparation of [(IMes)$_2$ZnEt][B(C$_6$F$_5$)$_4$]. A modification of the literature preparation of [(IMes)$_2$ZnMe][B(C$_6$F$_5$)$_4$] was used.[8] Fluorobenzene (0.5 mL) was vacuum transferred into a J. Youngs resealable NMR tube containing (IMes)ZnEt$_2$[9] (13.5 mg, 32 mmol) and [Ph$_3$C][B(C$_6$F$_5$)$_4$] (29 mg, 32 mmol), which was then shaken for 10 min at –40 °C. The tube was rapidly taken back into a glovebox and IMes (9.3 mg, 32 mmol) added. After standing for 20 min at –32 °C in the glovebox freezer, the solution was concentrated and layered with hexane at –32 °C to afford [(IMes)$_2$ZnEt][B(C$_6$F$_5$)$_4$] as a yellow solid. ^1H NMR (THF-d_8, 400 MHz, 298 K): δ 7.42 (s, 4H, Ar), 7.01 (s, 8H, Ar), 2.41 (s, 12H, *p*-Me), 1.76 (s, 24H, *o*-Me), 0.54 (t, $^3J_{HH}$ = 8.0 Hz, 3H, Zn–CH$_2$*Me*), –0.48 (d, $^3J_{HH}$ = 8.0 Hz, 2H, Zn–C*H*$_2$Me).

Ir(IPr)$_2$H$_5$ (13). A J. Young's resealable ampule was charged with a C_6H_5F (5 mL) solution of **1** (200 mg, 0.110 mmol) and KHMDS (52 mg, 0.250 mmol), degassed (freeze-pump-thaw x 3) and placed under 1 atm H_2, resulting in a rapid change from yellow-orange to colorless. After stirring at room temperature for 1 h, solvent was removed under vacuum and the product extracted with hexane to yield **13** as a colorless solid. Yield: 75 mg (71 %). ^1H NMR (C_6D_6, 400 MHz, 298 K): δ 7.31 (t, $^3J_{HH}$ = 7.6 Hz, 4H, Ar), 7.13 (d, $^3J_{HH}$ = 7.6 Hz, 8H, Ar), 6.29 (s, 4H, NC*H*=CHN), 2.64 (sept, $^3J_{HH}$ = 6.9 Hz, 8H, C*H*Me$_2$), 1.24 (d, $^3J_{HH}$ = 6.7 Hz, 24H, CH*Me*$_2$), 1.04 (d, $^3J_{HH}$ = 7.0 Hz, 24H, CH*Me*$_2$), –9.66 (s, 5H, Ir–*H*; T_1 938 ms (228 K, 400 MHz, $C_6D_5CD_3$)). $^{13}C\{^1H\}$ NMR (C_6D_6, 126 MHz, 298 K): δ 168.4 (s, Ir–C_{IPr}), 146.0 (s), 140.2 (s), 128.8 (s), 123.8 (s), 121.3 (s, NCH=*C*HN), 28.8 (s, *C*HMe$_2$), 25.2 (s, CH*Me*$_2$), 23.4 (s, CH*Me*$_2$). ATR-IR (cm^{-1}): 1958 ($\nu_{Ir–H}$), 1923 ($\nu_{Ir–H}$). Anal. Calcd for $C_{54}H_{77}N_4Ir$ (974.38): C, 66.56; H, 7.97; N, 5.75. Found: C, 66.14; H, 7.82; N, 5.70.

$[Ir(IPr)_2(ZnEt)(PMe_3)H_3][BAr^F_4]$ (14). A solution of **11c** (100 mg, 0.0518 mmol) and PMe_3 (10.5 μL, 0.104 mmol) was stirred in C_6H_5F (4 mL) in a J. Youngs ampule for 3 h. After being reduced to dryness, the residue was redissolved in C_6H_5F and layered with hexane affording orange crystals of **14**; **13** remained in solution. Yield: 33 mg (31%). ^1H NMR (CD_2Cl_2, 400 MHz, 298 K): δ 7.72 (br s, 8H, BAr^F_4), 7.56 (br s, 4H, BAr^F_4), 7.53 (t, $^3J_{HH}$ = 7.7 Hz, 2H, Ar), 7.47 (t, $^3J_{HH}$ = 7.7 Hz, 2H, Ar), 7.38 (d, $^3J_{HH}$ = 7.7 Hz, 2H, Ar), 7.33 (d, $^3J_{HH}$ = 7.4 Hz, 2H, Ar), 7.23 (d, $^3J_{HH}$ = 7.7 Hz, 2H, Ar), 7.09 (d, $^3J_{HH}$ = 7.4 Hz, 2H, Ar), 6.97-6.93 (m, 4H, NC*H*=CHN), 2.85 (sept, $^3J_{HH}$ = 7.1 Hz, 2H, C*H*Me$_2$), 2.36 (sept, $^3J_{HH}$ = 6.7 Hz, 4H, C*H*Me$_2$), 2.18 (br sept, $^3J_{HH}$ = 6.3 Hz, 2H, C*H*Me$_2$), 1.29 (d, $^3J_{HH}$ = 6.7 Hz, 6H, CH*Me$_2$*), 1.27 (d, $^3J_{HH}$ = 6.7 Hz, 6H, CH*Me$_2$*), 1.07-0.79 (overlapping m, 48H, CH*Me$_2$* + P*Me$_3$* + Zn–CH$_2$*Me*), –0.21 (quart, $^3J_{HH}$ = 8.1 Hz, 2H, Zn–*CH$_2$*Me), –9.37 (dt, $^2J_{HP}$ = 15.5 Hz, $^2J_{HH}$ = 4.8 Hz, 1H, Ir–*H*), –11.38 (dd, $^2J_{HP}$ = 18.1 Hz, $^2J_{HH}$ = 4.9 Hz, 1H, Ir–*H*), –12.01 (br dd, $^2J_{HP}$ = 89.4 Hz, $^2J_{HH}$ = 3.8 Hz, 1H, Ir–*H*). $^{13}C\{^1H\}$ NMR (CD_2Cl_2, 101 MHz, 298 K):* δ 162.3 (1:1:1:1 quart, $^1J_{CB}$ = 50 Hz, BAr^F_4), 146.8 (s), 146.4 (s), 145.4 (s), 144.3 (s), 139.1 (s), 138.1 (s), 135.4 (br s, BAr^F_4), 131.8 (s), 130.7 (s), 129.4 (br quart, $^2J_{CF}$ = 33 Hz, BAr^F_4), 126.8 (s), 126.5 (s), 126.0 (br s, NCH=*C*HN), 125.9 (s), 125.0 (quart, $^1J_{CF}$ = 272 Hz, BAr^F_4), 125.1 (s, C_{Ar}), 118.0 (br s, BAr^F_4), 30.1 (s, *C*HMe$_2$), 29.6 (s, *C*HMe$_2$), 29.3 (s, *C*HMe$_2$), 29.2 (s, *C*HMe$_2$), 27.3 (s, *C*HMe$_2$), 26.8 (s, *C*HMe$_2$), 26.6 (s, *C*HMe$_2$), 26.0 (s, *C*HMe$_2$), 23.4 (s, *C*HMe$_2$), 23.1 (d, $^1J_{CP}$ = 33 Hz, PMe_3), 23.0 (s, *C*HMe$_2$), 22.8 (s, *C*HMe$_2$), 22.3 (s, *C*HMe$_2$), 18.8 (d, $^3J_{CP}$ = 7 Hz, Zn–*CH$_2$*Me), 10.7 (s, Zn–CH$_2$*Me*). *Ir–C_{IPr} not observed. $^{31}P\{^1H\}$ NMR (CD_2Cl_2, 162 MHz, 298 K): δ –54.6 (s). ATR-IR (cm^{-1}): 1916 ($\nu_{Ir–H}$), 1667 ($\nu_{Ir–H}$). ESI-TOF MS: $[M]^+$ *m*/*z* = 1141.5772 (theoretical *m*/*z* = 1141.5731).

$Ir(IPr)_2(ZnPh)H_4$ (15a). A toluene (0.5 mL) solution of **13** (20 mg, 0.021 mmol) and $ZnPh_2$ (6 mg, 0.027 mmol) was heated at 100 °C for 2 h. The resulting suspension was filtered, the filtrate reduced to dryness and recrystallized as a concentrated solution in hexane

at −40 ^{0}C to yield **15a** as colorless crystals. Yield: 14 mg (63 %). ^{1}H NMR ($C_6D_5CD_3$, 400 MHz, 278 K): δ 7.46 (br d, J_{HH} = 6.9 Hz, 2H, Ar), 7.36 (t, J_{HH} = 7.3 Hz, 2H, Ar), 7.28-7.22 (m, 6H, Ar),* 6.37 (s, 4H, NC*H*=CHN), 2.76 (br sept, $^3J_{HH}$ = 6.7 Hz, 8H, C*H*Me$_2$), 1.08 (br d, $^3J_{HH}$ = 6.7 Hz, 24H, CH*Me*$_2$), 1.01 (br d, $^3J_{HH}$ = 6.7 Hz, 24H, CH*Me*$_2$), −10.48 (s, 2H, Ir–*H*; T_1 635 ms (228 K, 400 MHz, $C_6D_5CD_3$)), −11.33 (s, 2H, Ir–*H*; T_1 523 ms (228 K, 400 MHz, $C_6D_5CD_3$)). *Remaining aromatic protons are obscured by toluene. ^{13}C{^{1}H} NMR ($C_6D_5CD_3$, 101 MHz, 278 K): δ 171.0 (s, Ir–C_{IPr}), 166.0 (s, Zn–C_{ipso}), 145.7 (s), 140.5 (s), 137.2 (s), 127.1 (s), 126.2 (s),* 124.3 (s), 122.2 (s, NCH=*C*HN), 29.0 (s, *C*HMe$_2$), 25.4 (s, CH*Me*$_2$), 23.2 (s, CH*Me*$_2$). *Remaining aromatic resonances are obscured by toluene. ATR-IR (cm^{-1}): 2084 (ν_{Ir-H}), 1683 ($\nu_{Ir-H-Zn}$). Anal. Calcd for $C_{60}H_{81}N_4ZnIr$ (1115.91): C, 64.58; H, 7.32; N, 5.02. Found: C, 65.27; H, 7.75; N, 4.71.

Ir(IPr)$_2$(ZnMe)H$_4$ (15b). A C_6H_6 (0.5 mL) solution of **13** (20 mg, 0.021 mmol) was treated with $ZnMe_2$ (50 µL of 0.57 M solution in heptane, 0.029 mmol) and then heated at 50 °C for 3 h. The resulting suspension was filtered, the filtrate reduced to dryness and a concentrated hexane solution of the residue left at −40 ^{0}C to yield colorless crystals of **15b**. Yield: 12 mg (55 %). ^{1}H NMR (C_6D_6, 400 MHz, 298 K): δ 7.29 (t, $^3J_{HH}$ = 7.7 Hz, 4H, Ar),* 6.36 (s, 4H, NC*H*=CHN), 2.73 (sept, $^3J_{HH}$ = 6.8 Hz, 8H, C*H*Me$_2$), 1.22 (d, $^3J_{HH}$ = 6.8 Hz, 24H, CH*Me*$_2$), 0.99 (d, $^3J_{HH}$ = 6.8 Hz, 24H, CH*Me*$_2$), −0.62 (s, 3H, Zn–*Me*), −10.58 (s, 2H, Ir–*H*; T_1 459 ms (228 K, 400 MHz, THF-d_8)), −11.37 (s, 2H, Ir–*H*; T_1 348 ms (228 K, 400 MHz, THF-d_8)).*Remaining Ar protons are obscured by C_6D_5H. ^{13}C{^{1}H} NMR (C_6D_6, 101 MHz, 298 K): δ 171.7 (s, Ir–C_{IPr}), 146.0 (s), 140.6 (s), 129.0 (s), 124.3 (s), 122.1 (s, NC*H*=CHN), 28.7 (s, *C*HMe$_2$), 25.2 (s, CH*Me*$_2$), 23.0(s, CH*Me*$_2$), 8.0 (s, Zn–*Me*). ATR-IR (cm^{-1}): 2087 (ν_{Ir-H}), 1703 ($\nu_{Ir-H-Zn}$). Anal. Calcd for $C_{55}H_{79}N_4ZnIr$ (1053.81): C, 62.68; H, 7.56; N, 5.32. Found: C, 62.39; H, 7.43; N, 5.21.

Ir(IPr)$_2$(ZnEt)H$_4$ (15c). $ZnEt_2$ (30 μL of 0.97 M solution in hexane, 0.029 mmol) was added to a C_6H_6 (0.5 mL) solution of **13** (20 mg, 0.021 mmol) and the mixture heated at 80 °C for 2 h. The resulting suspension was filtered, the filtrate reduced to dryness and a concentrated hexane solution of the residue left at −40 ^{0}C to yield colorless crystals of **15c**. Yield: 14 mg (62 %). ^{1}H NMR (C_6D_6, 400 MHz, 298 K): δ 7.29 (t, $^3J_{HH}$ = 7.7 Hz, 4H, Ar),* 6.36 (s, 4H, NC*H*=CHN), 2.71 (sept, $^3J_{HH}$ = 6.7 Hz, 8H, C*H*Me$_2$), 1.42 (t, $^3J_{HH}$ = 8.1 Hz, 3H, ZnCH$_2$*Me*), 1.22 (d, $^3J_{HH}$ = 6.7 Hz, 24H, CH*Me*$_2$), 1.00 (d, $^3J_{HH}$ = 6.7 Hz, 24H, CH*Me*$_2$), 0.0 (quart, $^3J_{HH}$ = 8.1 Hz, 2H, Zn–*CH*$_2$Me), −10.64 (s, 2H, Ir–*H*; T_1 587 ms (228 K, 400 MHz, $C_6D_5CD_3$)), −11.46 (s, 2H, Ir–*H*; T_1 488 ms (228 K, 400 MHz, $C_6D_5CD_3$)).* Remaining aromatic protons are obscured by C_6D_5H. ^{13}C{^{1}H} NMR (C_6D_6, 101 MHz, 298 K): δ 170.0 (s, Ir–C_{IPr}), 145.8 (s), 140.5 (s), 128.9 (s), 124.2 (s), 122.0 (s, NC*H*=CHN), 29.0 (s, *C*HMe$_2$), 25.5 (s, CH*Me*$_2$), 23.2 (s, CH*Me*$_2$), 20.2 (s, Zn–*C*H$_2$Me), 11.3 (s, Zn–CH$_2$*Me*). ATR-IR (cm^{-1}): 2089 (ν_{Ir-H}), 1704 ($\nu_{Ir-H-Zn}$). Anal. Calcd for $C_{56}H_{81}N_4ZnIr$ (1067.84): C, 62.98; H, 7.65; N, 5.25. Found: C, 62.54; H, 7.56; N, 5.04.

Ir(IPr)$_2$(CdMe)H$_4$ (16). A solution of **13** (20 mg, 0.021 mmol) in C_6H_6 (0.5 mL) was treated with $CdMe_2$ (10 μL of 2.4 M solution in hexane, 0.024 mmol) and heated at 50 °C for 3 h. The resulting suspension was then filtered, the filtrate reduced to dryness and recrystallized from a concentrated hexane solution at −40 ^{0}C to yield colorless crystals of **16**. Yield: 11 mg (49 %). ^{1}H NMR (C_6D_6, 400 MHz, 298 K): δ 7.29 (t, $^3J_{HH}$ = 7.7 Hz, 4H, Ar),* 6.33 (s, 4H, NC*H*=CHN), 2.72 (sept, $^3J_{HH}$ = 6.9 Hz, 8H, C*H*Me$_2$), 1.23 (d, $^3J_{HH}$ = 6.9 Hz, 24H, CH*Me*$_2$), 0.98 (d, $^3J_{HH}$ = 6.9 Hz, 24H, CH*Me*$_2$), −0.41 (s + sat ($^2J_{HCd}$ = 53 Hz), 3H, Cd–*Me*), −9.83 (s + sat ($J_{H-113Cd}$ = 347 Hz, $J_{H-111Cd}$ = 332 Hz), 2H, Ir–*H*), −10.72 (s + sat (J_{HCd} = 94 Hz), 2H, Ir–*H*). *Remaining aromatic protons are obscured by C_6D_5H. ^{13}C{^{1}H} NMR (C_6D_6, 101 MHz, 298 K): δ 171.7 (s, Ir–C_{IPr}), 145.9 (s), 140.7 (s), 128.9 (s), 124.2 (s),

121.8 (s, NC*H*=CHN), 29.0 (s, *C*HMe$_2$), 25.4 (s, *Me*), 23.2 (s, *Me*), 12.5 (s, Cd–*Me*). ^{113}Cd{^{1}H} NMR (C_6D_6, 111 MHz, 298 K): δ −163.1 (s). ATR-IR (cm^{-1}): 2095 ($\nu_{Ir–H}$), 1726 ($\nu_{Ir–H–Cd}$).

S1.3 X-ray crystallography

Data for **4a**, **5**, **8**, **14** and **15b** were obtained using an Agilent SuperNova instrument and a Cu-Kα source, while those for **4c** and **11b** were gathered on the same instrument using Mo-Kα radiation. All experiments were conducted at 150 K and all structures were solved using SHELXT.[10] Refinements were effected using SHELXL[11] via the Olex2[12] interface. Where disorder prevailed, appropriate distance and ADP restraints were included, in these regions, to assist convergence. The following narrative is confined to additional, noteworthy information.

There is one cation, one anion and a region of solvent in the asymmetric unit of **4a**. The hydride (H1) was located in the cation and refined freely while the hydrogens attached to C6 were located and refined subject to being located 0.98 Å from the parent atom. In the anion, the fluorines attached to C80 and C91 were treated for a 55:45 split and the entire CF_3 group which includes C67 was modeled to take account of 50:50 disorder. The solvent region was diffuse and hence addressed using the solvent mask algorithm in Olex2. A consequent allowance has been made for one molecule of hexane per unit cell, in the formula as presented. The asymmetric unit of **5** contains half of a cation, half of an anion and a region of solvent. The remainder of the cation arises by virtue of an inversion center co-incident with Ir1, while the [BAr$^F_4$]$^-$ is completed as a result of the 2-fold rotation axis on which B1 is located. The cation hydrides (H1 and H2) were located and refined freely. In the anion, the fluorines attached to C37, C45 and C48 were treated for respective disorders of 60:40, 50:50 and 60:40. The solvent region was diffuse, disordered and was also noted to straddle a

crystallographic 2-fold rotation axis. It was clear that the electron density approximated to half of a molecule of fluorobenzene in which the fluorine was disordered over at least two positions. Hence, this region of the Fourier map was addressed using the solvent mask algorithm in Olex2, with a concomitant allowance made for four molecules of fluorobenzene, per unit cell, in the formula as presented.

In **4c**, there is one cation, one anion and one molecule of fluorobenzene in the asymmetric unit, all of which exhibited some disorder. Thus, in the cation, a persistent electron density peak proximate to the iridium (in an ordered model) was investigated as representing some disorder of this transition metal. This split ultimately refined in a ratio of 96:04. Thereafter, residual electron density adjacent to the zinc center was modeled at a similar disorder level to that of the iridium after which the hydride assigned as H1 was refined freely (at full-site occupancy) and remained stable in the ensuing least-squares cycles. Obviously, the proposed metal disorder would necessitate additional disorder in the cation, but location of ligand fractions at a 4% occupancy level was (unsurprisingly) not attempted. Regarding the hydride, location of same and consequent inclusion in the model was only possible after modeling the metal center disorder – and this was undertaken because the raw frames of data revealed that the crystal was entirely single in nature and devoid of any surface microcrystallites. The hydrogen atoms attached to C14 were located and refined at a distance of 0.98 Å from the parent atom. In the $[BAr^F{}_4]^-$, the fluorines in the CF_3 groups based on C62, C76 and C87 were treated for respective disorders of 60:40, 50:50 and 75:25 while the entire CF_3 moiety which included C63 was refined to account for 75:25 disorder. Finally, the solvent was modeled as two components (55:45 ratio), and the rings therein treated as regular hexagons in the refinement.

One cation, one anion and a region of disordered solvent constitute the asymmetric unit in the structure of **8**. The Cd–Me ligand in the former was modeled to take account of

60:40 disorder. Given that the Cd-Ir distances for each component are significantly different, it is likely that the Ir center is also disordered to a degree. However, efforts to split the associated electron density were not successful, possibly because of inevitable proximity of putative fractional centers. Consequently, a disorder treatment for Ir1 was abandoned on the basis that it would require imposition of significant restraints which could be deemed artificial in the absence of definitive evidence from the electron density itself. Moreover, it is not unreasonable to rationalize, given the 'windscreen-wiper' Cd−Me disorder present, why the Ir1−Cd1 and Ir1−Cd1a distances are observed to be different in the absence of being able to model iridium disorder. The hydrogen atoms attached to C5 were located and refined at a distance of 0.98 Å from the parent atom. Residual electron density ripples proximate to the iridium, unfortunately, precluded hydride location. CF_3 disorder was modeled for the fluorines attached to C74 and C82 in a 60:40 ratio, while the whole of the trifluoromethyl groups containing C61, C69 and C77 were also treated for 60:40 disorder. The solvent was, very evidently, a molecule of fluorobenzene. However, the associated ADPs remained correlated after a valiant attempt to model via 2-component disorder. Hence, this region of the electron density map was addressed using the solvent mask algorithm available in Olex2.

The asymmetric unit in the structure of **11b** contains one cation and one anion. In the cation, the methyl groups attached to C13 were treated for 50:50 disorder, while the Zn1 and C55 atoms were modeled to take account of an 84:15 split. Once the latter disorder modeling was effected, assignment of one (H5) terminal and two bridging (H1, H2) hydrides could be readily made, plus a tentative assignment for a dihydrogen ligand attached to Ir1 (H3 and H4). Positions H1, H2, H4 and H5 have the greatest credibility based on their associated U_{iso} values. The U_{iso} for H3, by comparison, is significantly greater. However, given that H1-H5 have been refined with just one single restraint (of Ir1−H3 and Ir1−H4 being similar) and with full-site occupancy (rather than 84%, as they are relative to the major component of

Zn1) it is reasonable to suggest that these positions, collectively, offer a snapshot of what is happening around the metal core, in this fluxional system. However, these assignments must also be tempered against a backdrop of the well-known problems in assignment of hydrogen positions from X-ray data. In the anion, five of the CF_3 moieties succumbed to positional averaging over two sites. In particular, the fluorines attached to C79 and C86 were split in respective ratios of 65:35 and 60:40, while the entire functionalities that included C63, C78 and C87 were treated to take account of 55:45, 55:45 and 65:35 fractions.

One cation and one anion constitute the asymmetric unit in the structure of **14**. The hydrides in the cation were located and refined without restraints. In the anion, the fluorines attached to C65, C70, C73, C86 and C89 were each modeled to take account of 60:40 disorder. Finally, in **15b**, the asymmetric unit contains one molecule of the bimetallic complex and one molecule of fluorobenzene. The hydrides in the former were located and refined without restraints. The solvent was treated for three component disorder (41:32:27 ratio).

Table S1. Crystal data and structural refinement details.

Identification code	**4a**	**5**	**4c**	**8**
Empirical formula	$C_{92}H_{88}BF_{24}IrN_4Zn$	$C_{104}H_{103}BF_{25}IrN_4Zn_2$	$C_{94}H_{93}BF_{25}IrN_4Zn$	$C_{93}H_{91}BCdF_{25}IrN_4$
Formula weight	1974.04	2217.65	2022.10	2055.10
Crystal system	triclinic	monoclinic	monoclinic	monoclinic
Space group	*P*-1	*C*2/*c*	$P2_1/n$	$P2_1/n$
a/ Å	14.2626(2)	29.9562(2)	20.6387(7)	20.7380(2)
b/ Å	15.7896(2)	12.5900(1)	20.2427(7)	20.3079(1)
c/ Å	23.2264(3)	26.3575(1)	23.6642(9)	23.6207(2)
α/ °	108.060(1)	90	90	90
β/ °	98.236(1)	92.172(1)	113.159(4)	113.507(1)
γ/ °	93.194(1)	90	90	90
U/ $Å^3$	4893.67(12)	9933.55(11)	9089.8(6)	9122.21(14)
Z	2	4	4	4
ρ_{calc}/ g cm^{-3}	1.340	1.483	1.478	1.496
μ/ mm^{-1}	3.666	3.929	1.825	5.503
F(000)	1992.0	4488.0	4088.0	4128.0
Crystal size/ mm^3	0.207 × 0.193 × 0.152	0.18 × 0.141 × 0.127	0.172 × 0.052 × 0.021	0.177 × 0.126 × 0.077
2θ range for data collection/°	6.892 to 146.71	6.712 to 146.52	6.372 to 55.004	7.308 to 146.646
Index ranges	−17 ≤ h ≤ 17, −15 ≤ k ≤ 19, −28 ≤ l ≤ 27	−36 ≤ h ≤ 37, −15 ≤ k ≤ 15, −32 ≤ l ≤ 23	−26 ≤ h ≤ 26, −26 ≤ k ≤ 25, −30 ≤ l ≤ 25	−24 ≤ h ≤ 25, −20 ≤ k ≤ 25, −29 ≤ l ≤ 29
Reflections collected	70541	54819	88060	98403
Independent reflections, R_{int}	19518, 0.0378	9918, 0.0333	20832, 0.0869	18167, 0.0523
Data/restraints/parameters	19518/201/1219	9918/200/685	20832/477/1320	18167/579/1268
Goodness–of–fit on F^2	1.020	1.040	1.012	1.032
Final *R*1, *wR*2 [I>=2σ(I)]	0.0311, 0.0792	0.0311, 0.0828	0.0536, 0.0989	0.0484, 0.1170
Final *R*1, *wR*2 [all data]	0.0320, 0.0800	0.0340, 0.0854	0.0950, 0.1136	0.0531, 0.1204
Largest diff. peak/hole/ e $Å^{-3}$	1.16/−1.04	1.93/−0.38	1.05/−1.18	1.73/−2.38

Table S1 contd. Crystal data and structural refinement details.

Identification code	**11b**	**14**	**15b**
Empirical formula	$C_{87}H_{92}BF_{24}IrN_4Zn$	$C_{91}H_{101}BF_{24}IrN_4PZn$	$C_{61}H_{84}FIrN_4Zn$
Formula weight	1918.02	2006.10	1149.89
Crystal system	monoclinic	orthorhombic	orthorhombic
Space group	$P2_1/n$	$Pna2_1$	$P2_12_12_1$
a/ Å	13.33646(11)	28.5854(4)	12.6387(1)
b/ Å	16.62169(14)	20.37308(19)	12.8067(1)
c/ Å	39.9234(3)	15.6245(2)	35.1063(2)
α/ °	90	90	90
β/ °	97.9617(8)	90	90
γ/ °	90	90	90
U/ Å^3	8764.70(13)	9099.25(19)	5682.31(7)
Z	4	4	4
ρ_{calc}/ g cm^{-3}	1.454	1.464	1.344
μ/ mm^{-1}	1.887	4.110	5.309
F(000)	3880.0	4072.0	2376.0
Crystal size/ mm^3	0.256 × 0.182 × 0.142	0.193 × 0.084 × 0.081	0.184 × 0.168 × 0.078
2θ range for data collection/°	6.638 to 60.058	7.13 to 146.704	7.348 to 147.038
Index ranges	$-18 \leq h \leq 18$, $-23 \leq k \leq 22$, $-52 \leq l \leq 54$	$-35 \leq h \leq 35$, $-25 \leq k \leq 17$, $-19 \leq l \leq 19$	$-15 \leq h \leq 15$, $-15 \leq k \leq 15$, $-43 \leq l \leq 42$
Reflections collected	87335	116509	125041
Independent reflections, R_{int}	24038, 0.0325	18158, 0.0533	11378, 0.0369
Data/restraints/parameters	24038/436/1301	18158/256/1276	11378/555/737
Goodness–of–fit on F^2	1.028	1.019	1.024
Final R1, wR2 [I>=2σ(I)]	0.0331, 0.0736	0.0315, 0.0771	0.0175, 0.0458
Final R1, wR2 [all data]	0.0439, 0.0789	0.0371, 0.0806	0.0178, 0.0459
Largest diff. peak/hole/ e Å^{-3}	0.80/−0.95	0.70/−1.06	0.94/−0.60
Flack Parameter	−	0.228(6)	−0.0335(17)

S1.4. NMR and IR spectra

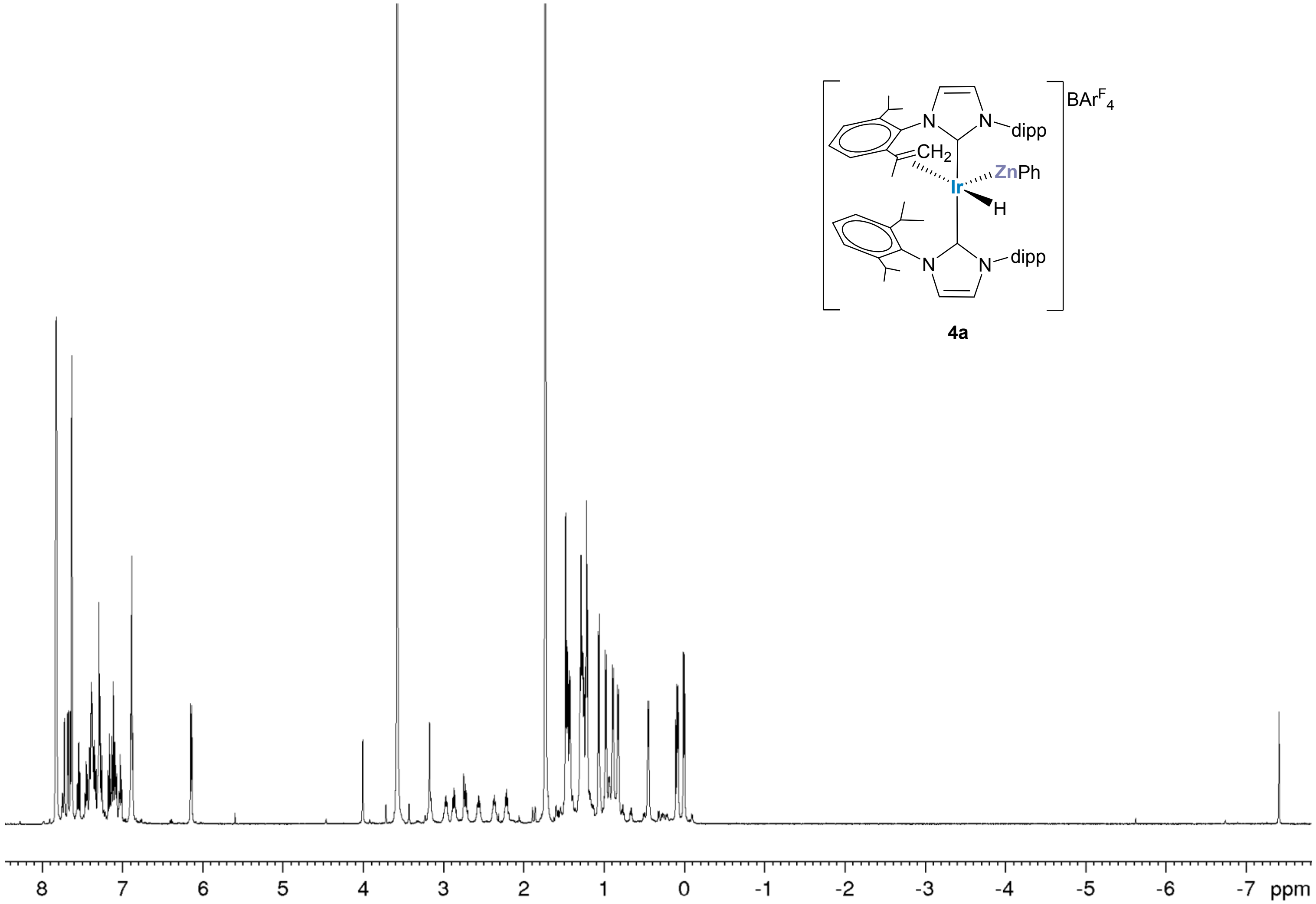

Figure S1. ^{1}H NMR spectrum (THF-d_8, 500 MHz, 248 K) of [Ir(IPr)(IPr'')(ZnPh)H][BAr$^F_4$] (**4a**).

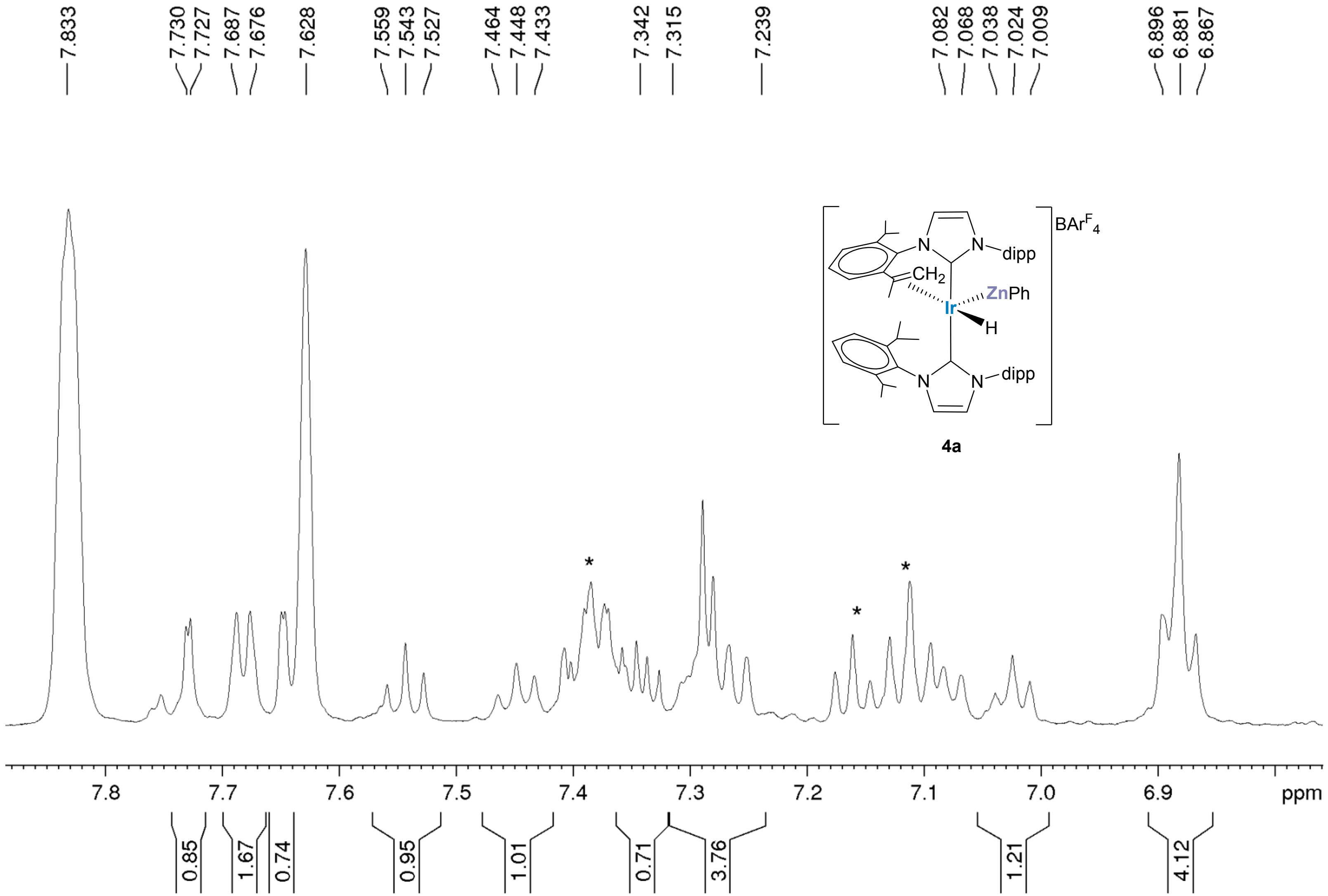

Figure S2. Aromatic region of the ^{1}H NMR spectrum (THF-d_8, 500 MHz, 248 K) of [Ir(IPr)(IPr")(ZnPh)H][BAr$^F{}_4$] (**4a**) (* = residual C_6H_5F).

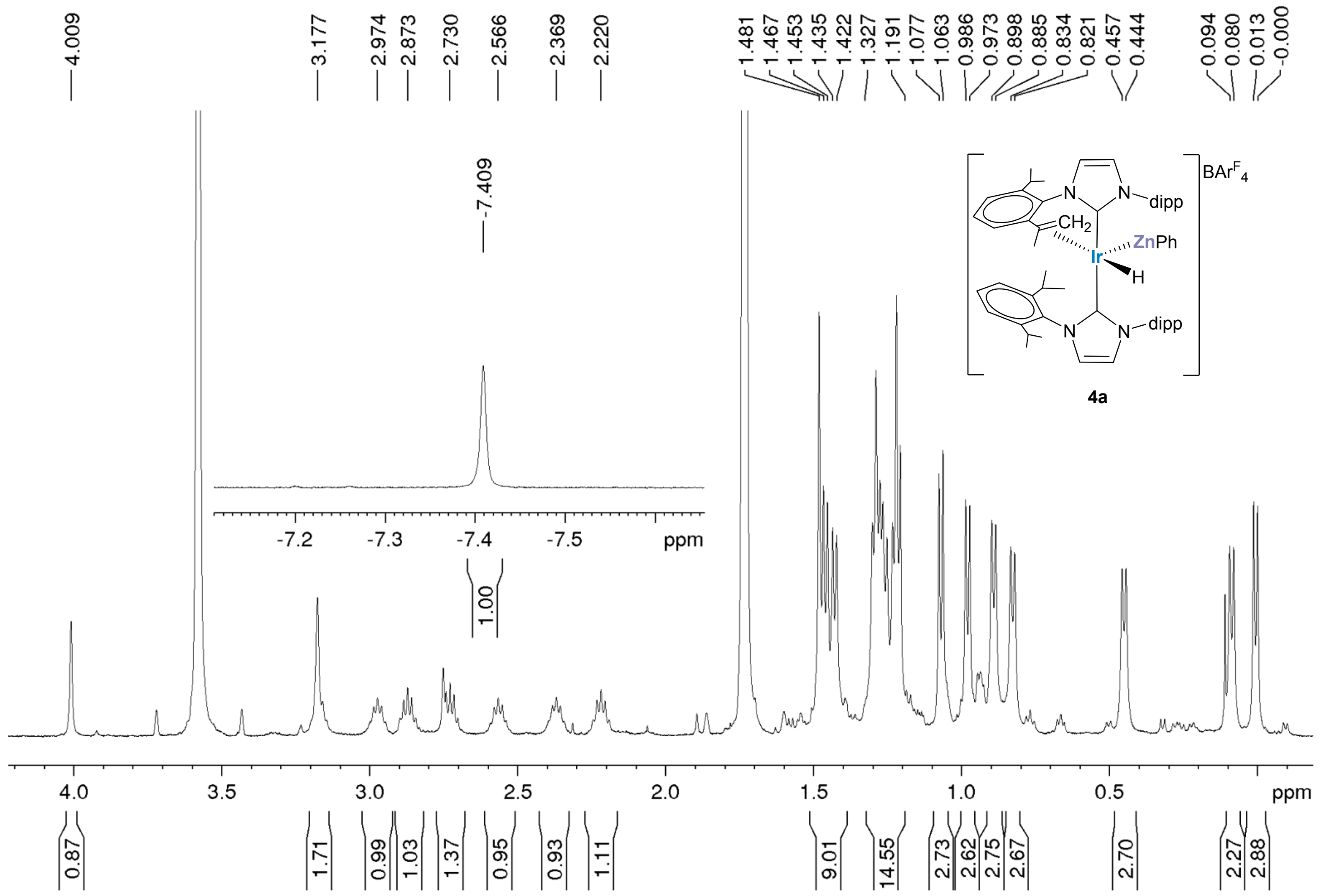

Figure S3. Alkyl region of the ^{1}H NMR spectrum (THF-d_8, 500 MHz, 248 K) of [Ir(IPr)(IPr'')(ZnPh)H][BAr$^F{}_4$] (**4a**), with inset of the Ir–H resonance.

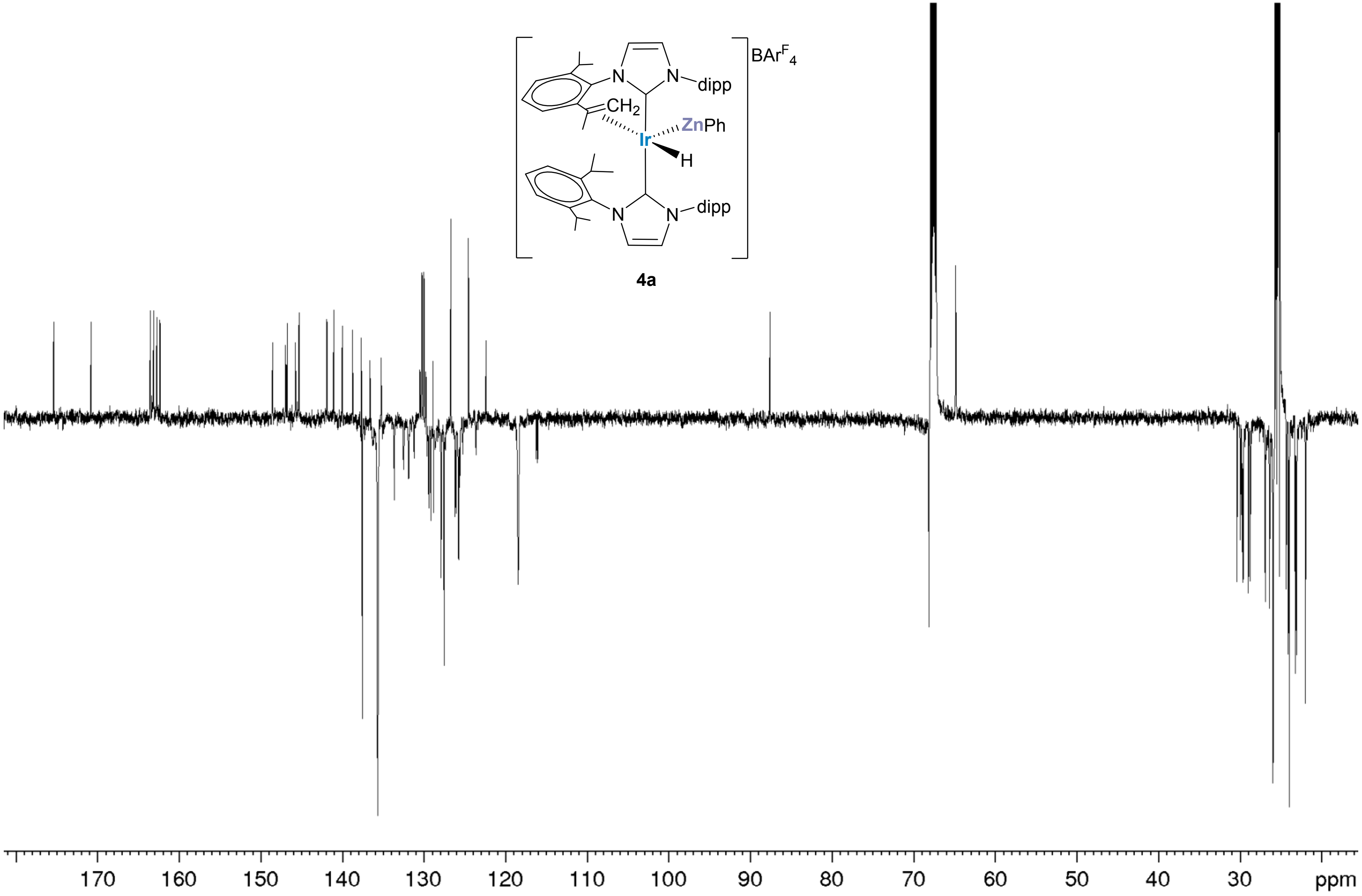

Figure S4. $^{13}C\{^1H\}$ DEPTQ NMR spectrum (THF-d_8, 126 MHz, 248 K) of $[Ir(IPr)(IPr'')(ZnPh)H][BAr^F_4]$ (**4a**).

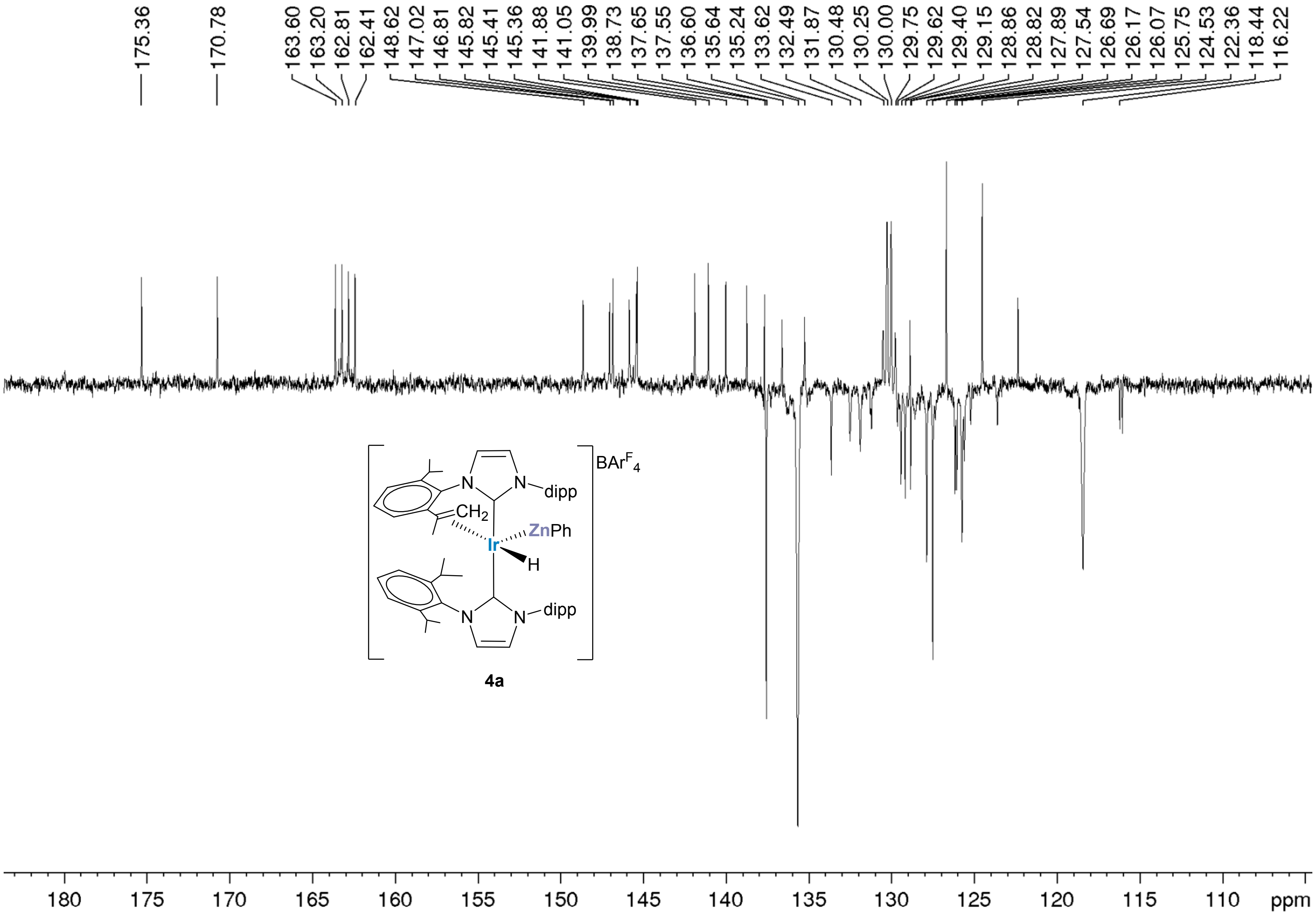

Figure S5. Aromatic region of the $^{13}C\{^{1}H\}$ DEPTQ NMR spectrum (THF-d_8, 126 MHz, 248 K) of [Ir(IPr)(IPr'')(ZnPh)H][BAr$^{F}_{4}$] (**4a**).

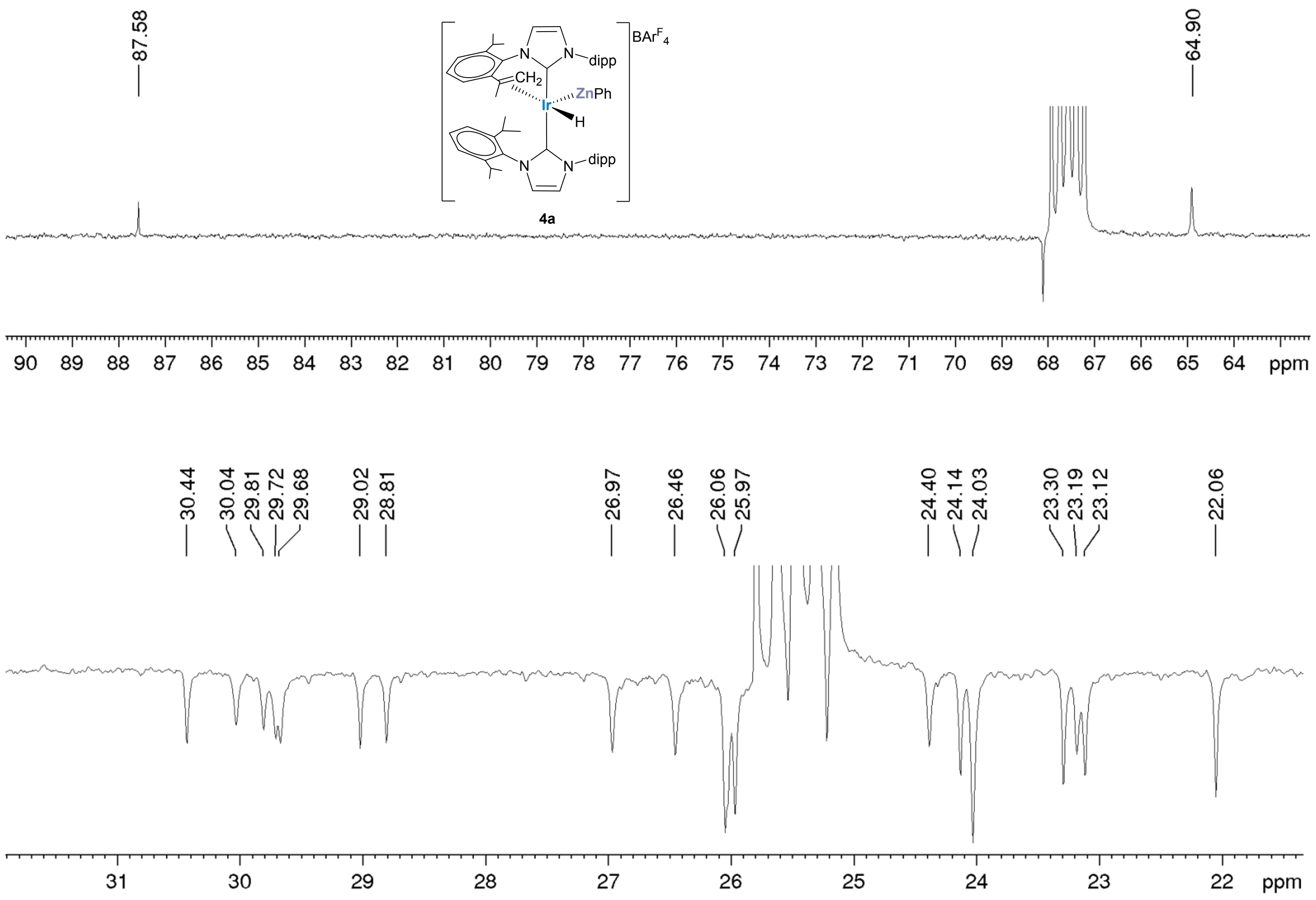

Figure S6. (Top) Alkenic and (bottom) alkyl regions of the $^{13}C\{^{1}H\}$ DEPTQ NMR spectrum (THF-d_8, 126 MHz, 248 K) of $[Ir(IPr)(IPr'')(ZnPh)H][BAr^F_4]$ (**4a**).

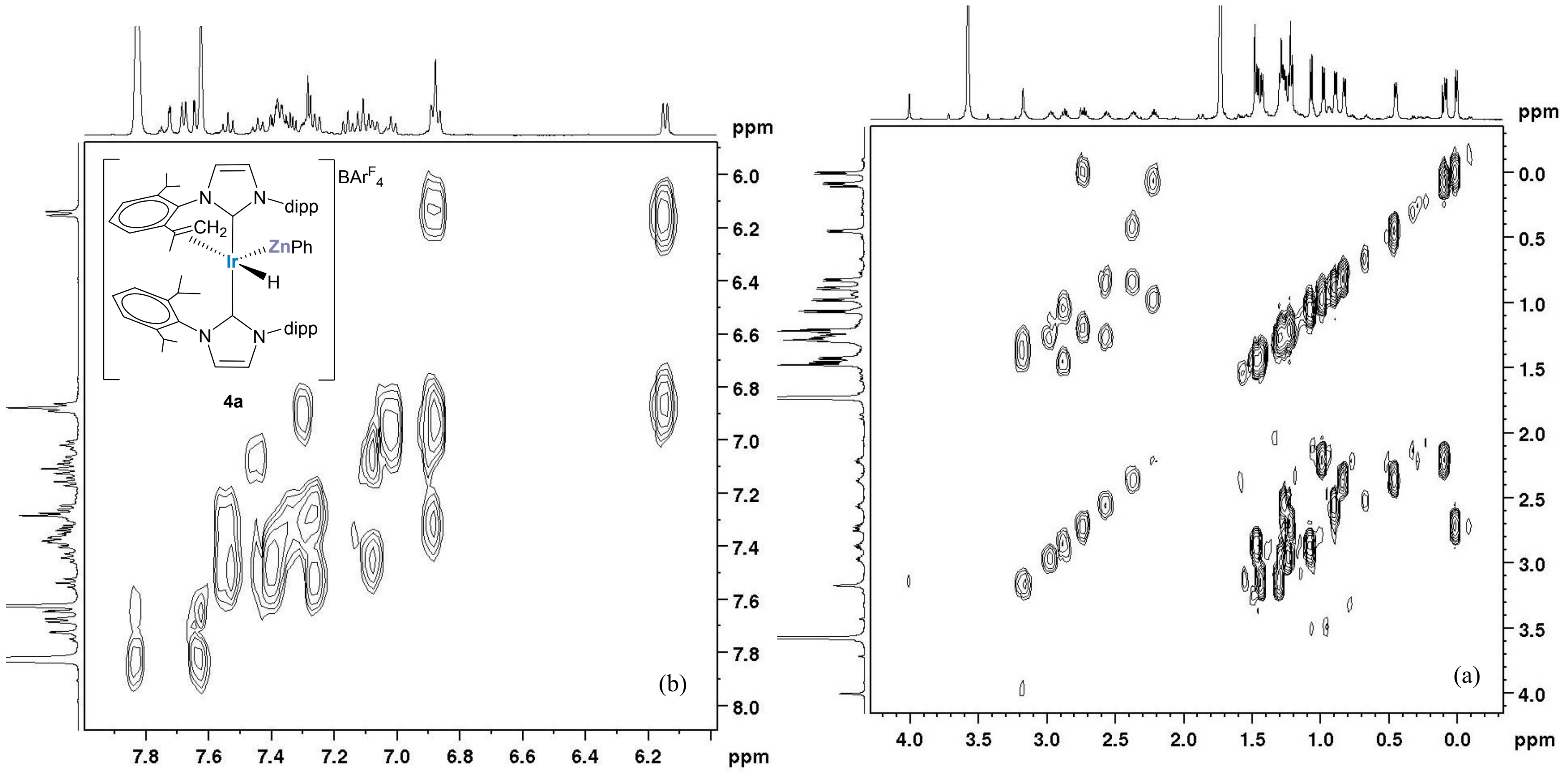

Figure S7. (a) Alkyl and (b) aryl regions of the ^{1}H COSY spectrum (THF-d_8, 500 MHz, 248 K) of [Ir(IPr)(IPr'')(ZnPh)H][BAr$^{F}_{4}$] (**4a**).

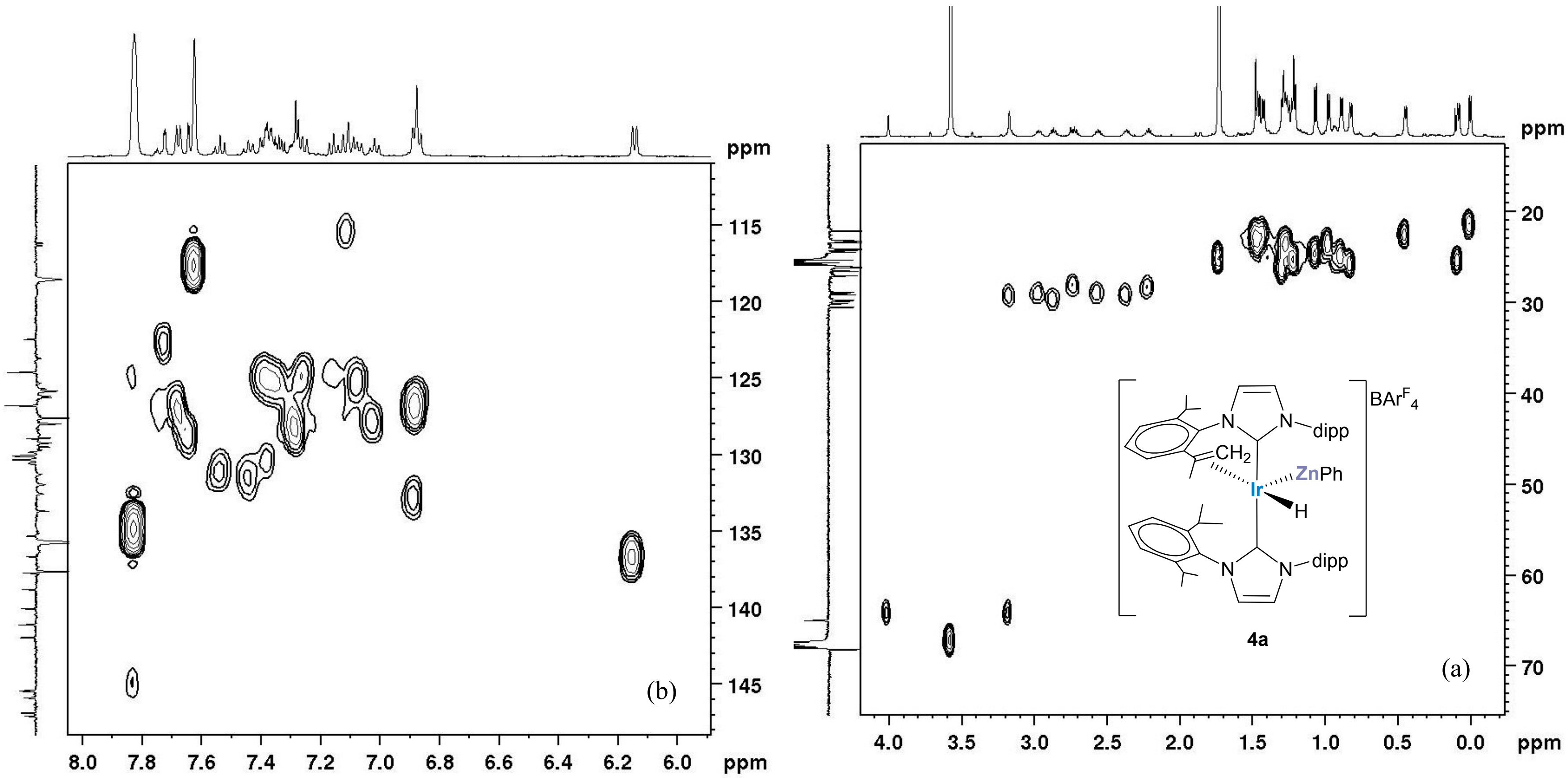

Figure S8. (a) Alkyl and (b) aryl regions of the ^{13}C-^{1}H HSQC spectrum (THF-d_8, 248 K) of [Ir(IPr)(IPr'')(ZnPh)H][BAr$^F{}_4$] (**4a**).

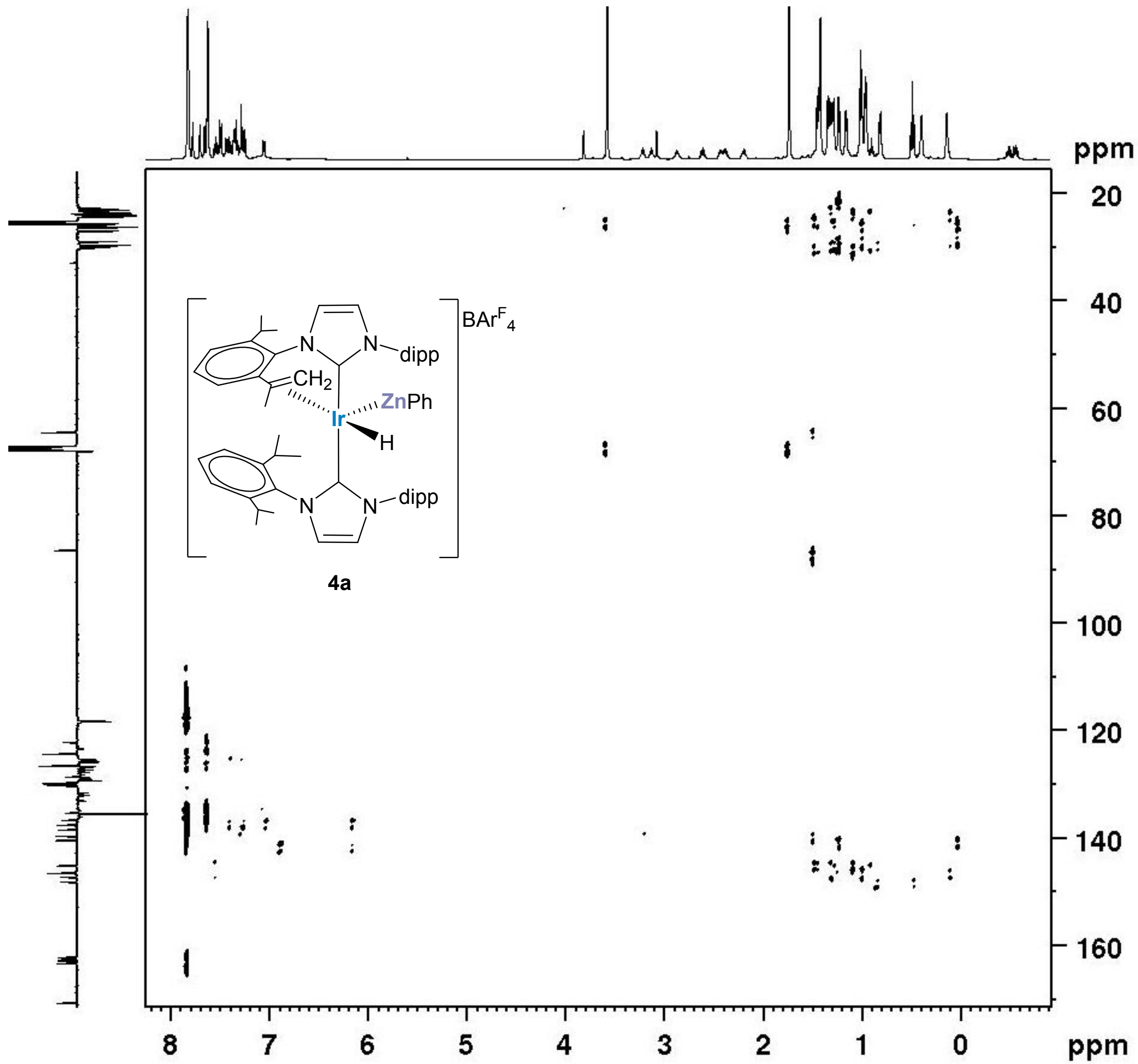

Figure S9. ^{13}C-^{1}H HMBC spectrum (THF-d_8, 248 K) of [Ir(IPr)(IPr'')(ZnPh)H][BAr$^F{}_4$] (**4a**).

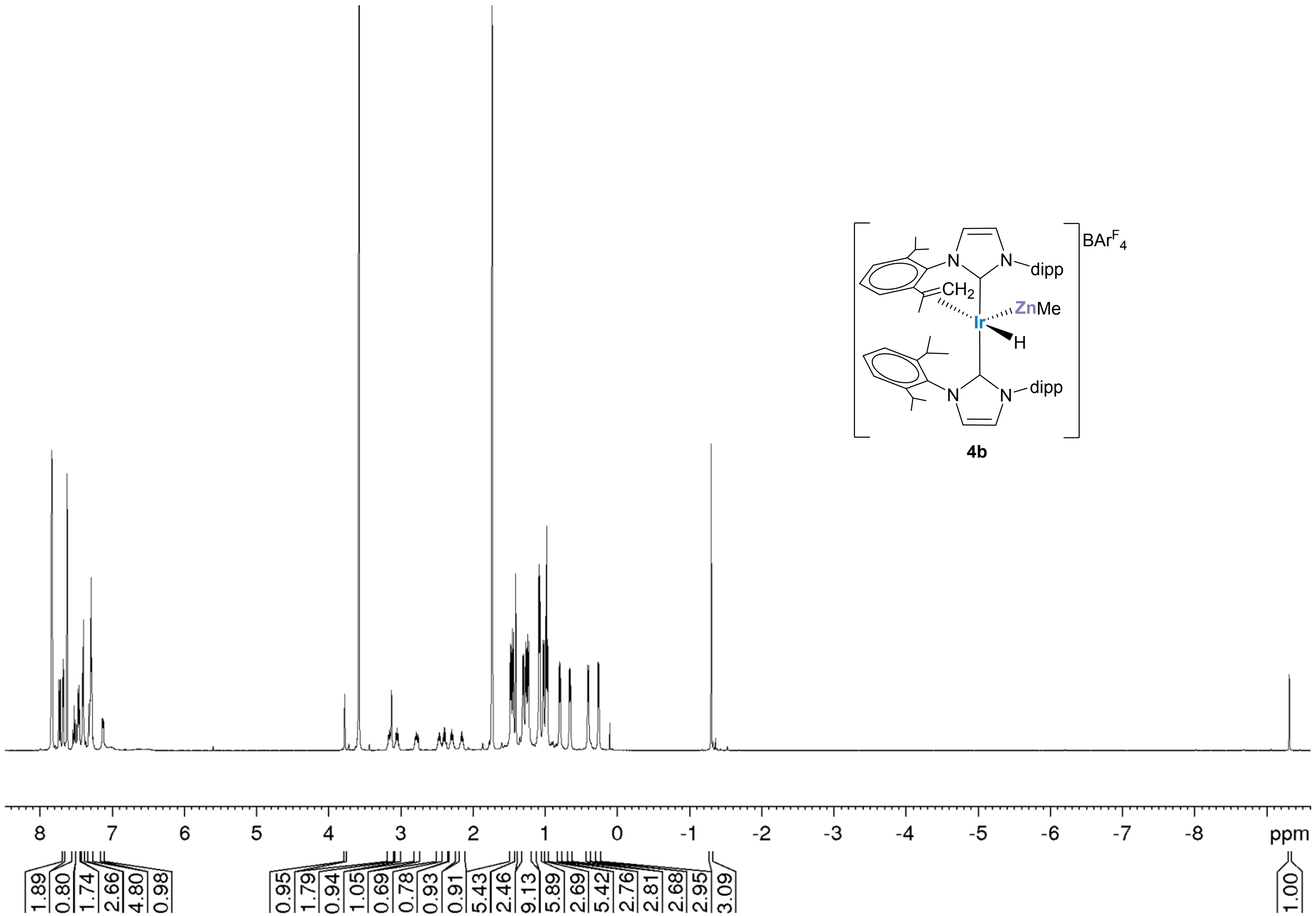

Figure S10. ^{1}H NMR spectrum (THF-d_8, 500 MHz, 248 K) of [Ir(IPr)(IPr'')(ZnMe)H][BAr$^F_4$] (**4b**).

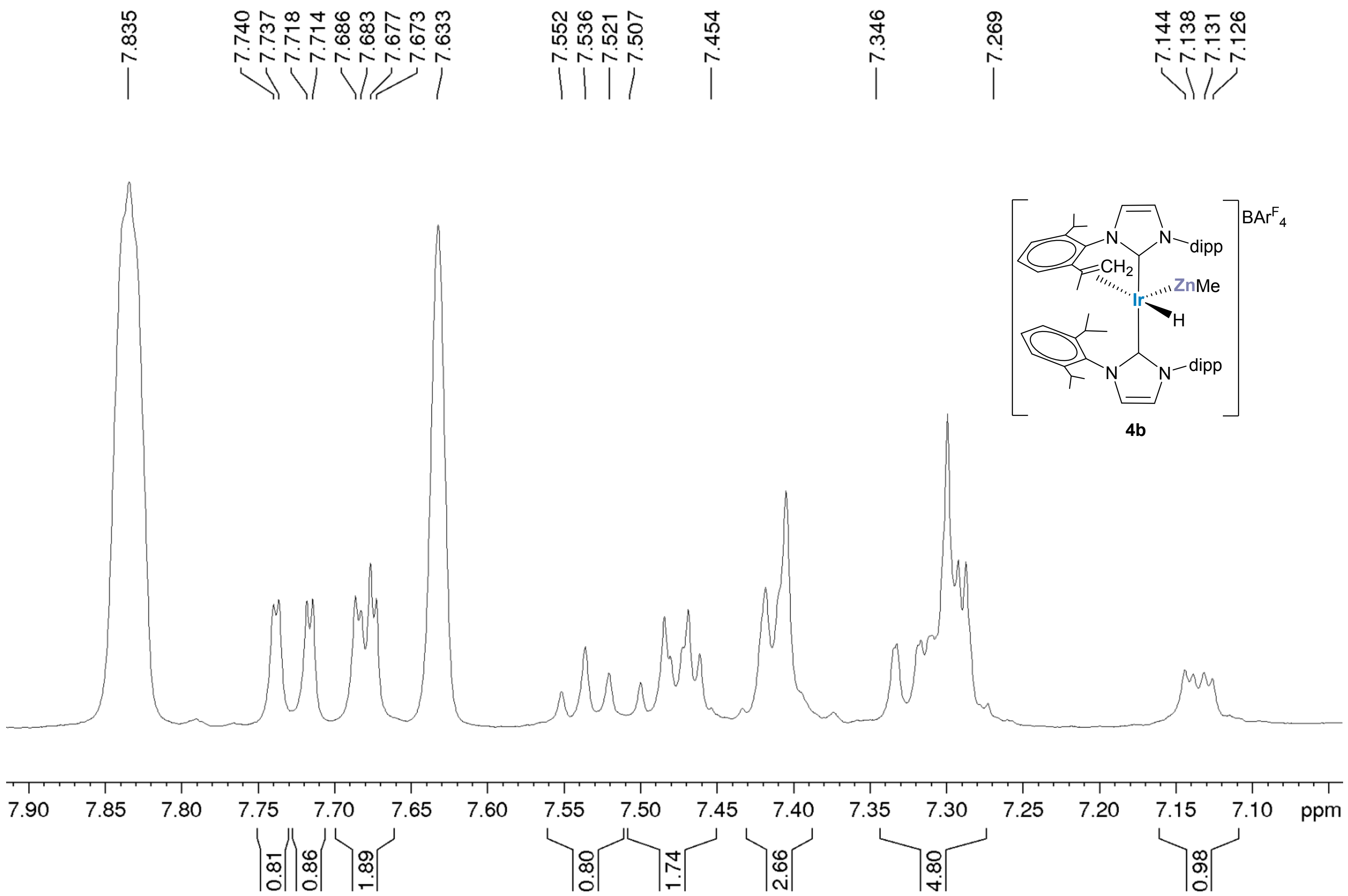

Figure S11. Aromatic region of the ^1H NMR spectrum (THF-d_8, 500 MHz, 248 K) of [Ir(IPr)(IPr'')(ZnMe)H][BAr$^F_4$] (**4b**).

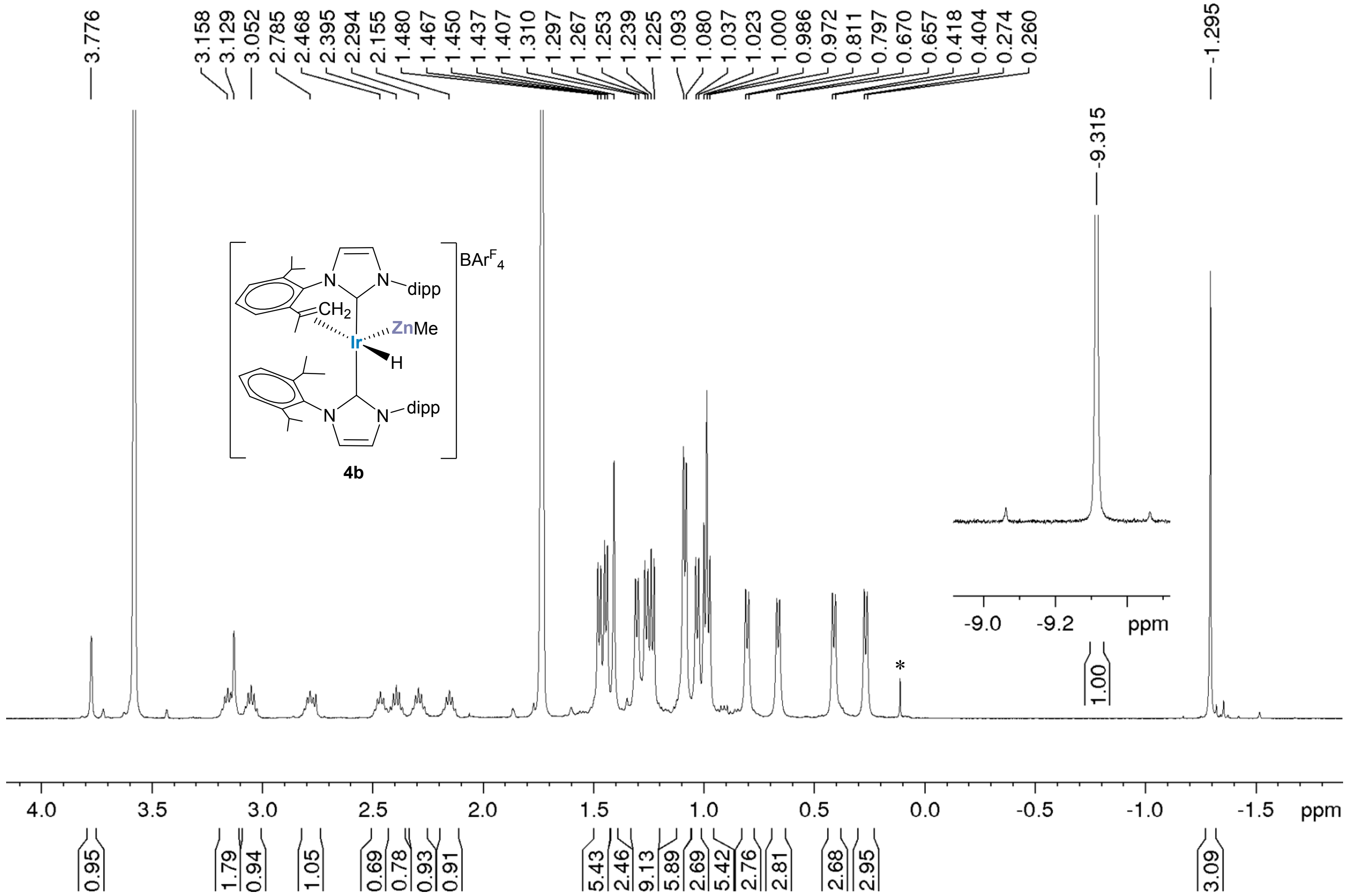

Figure S12. Alkyl region of the ^{1}H NMR spectrum (THF-d_8, 500 MHz, 248 K) of [Ir(IPr)(IPr")(ZnMe)H][BAr$^F_4$] (**4b**) with inset of the Ir−H resonance.

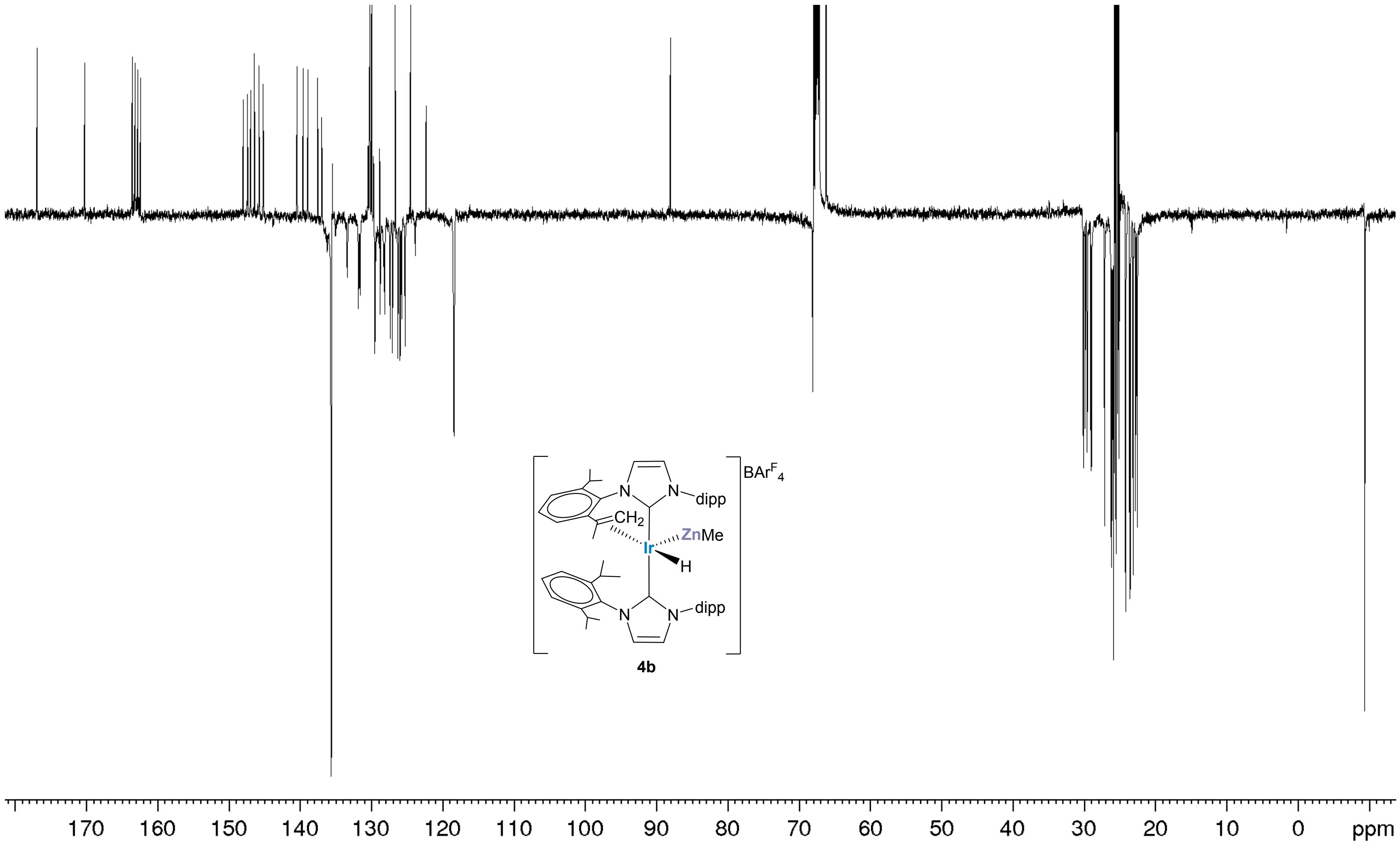

Figure S13. $^{13}C\{^1H\}$ DEPTQ NMR spectrum (THF-d_8, 126 MHz, 248 K) of [Ir(IPr)(IPr'')(ZnMe)H][BAr$^F_4$] (**4b**).

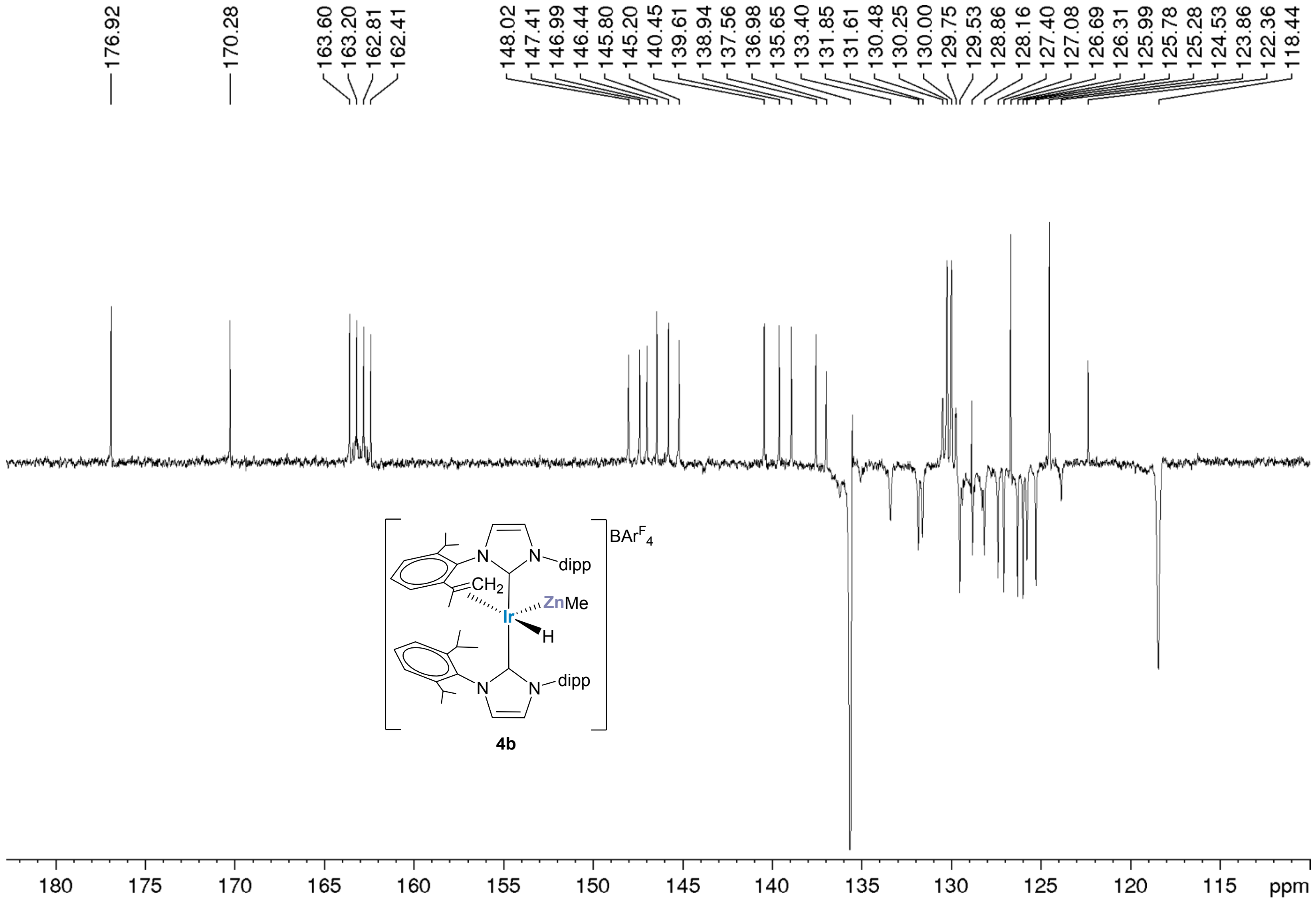

Figure S14. Aromatic region of the $^{13}C\{^{1}H\}$ DEPTQ NMR spectrum (THF-d_8, 126 MHz, 248 K) of [Ir(IPr)(IPr'')(ZnMe)H][BAr$^F{}_4$] (**4b**).

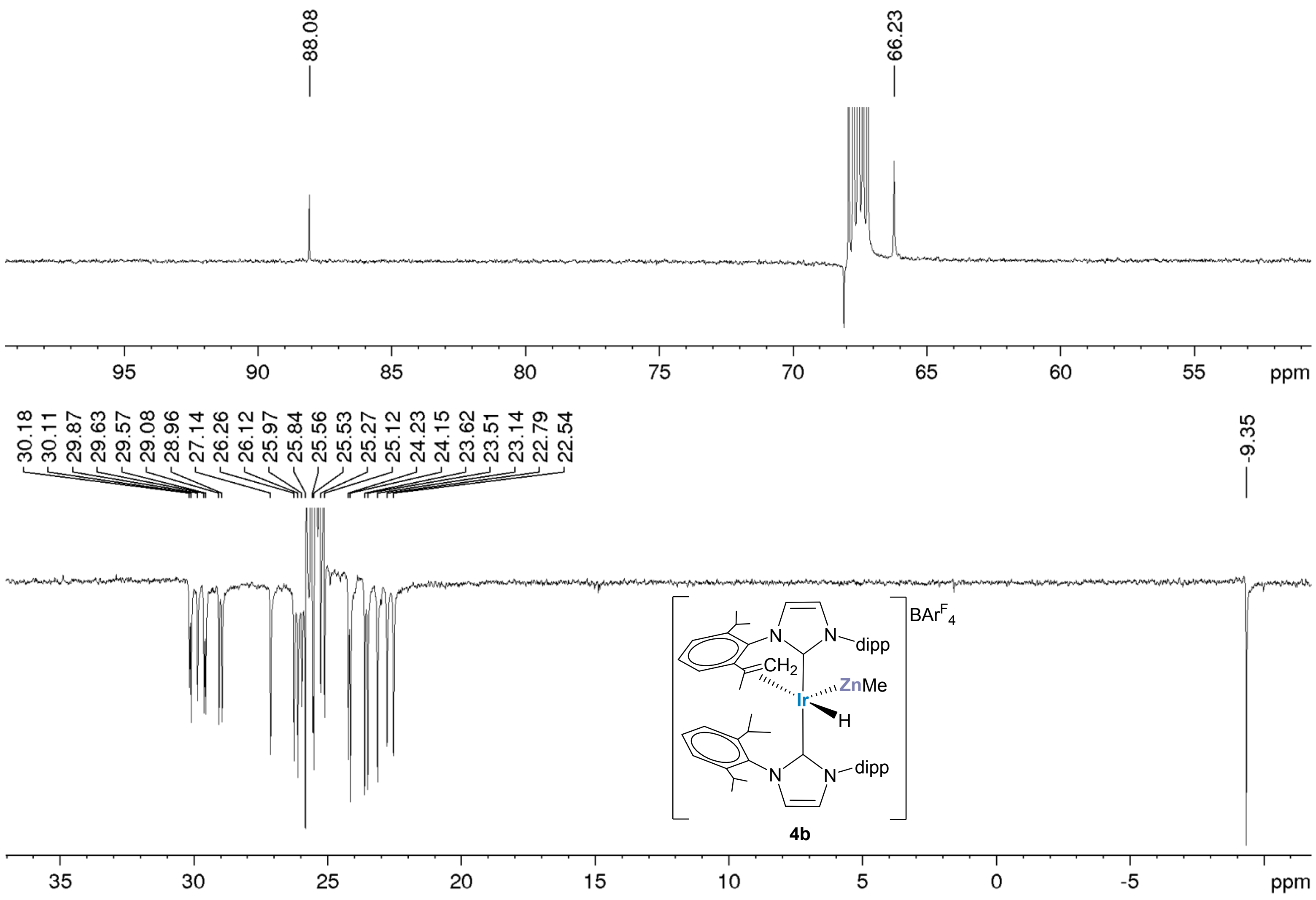

Figure S15. (Top) Alkenic and (bottom) alkyl regions of the $^{13}C\{^{1}H\}$ DEPTQ NMR spectrum (THF-d_8, 126 MHz, 248 K) of $[Ir(IPr)(IPr'')(ZnMe)H][BAr^{F}_{4}]$ (**4b**).

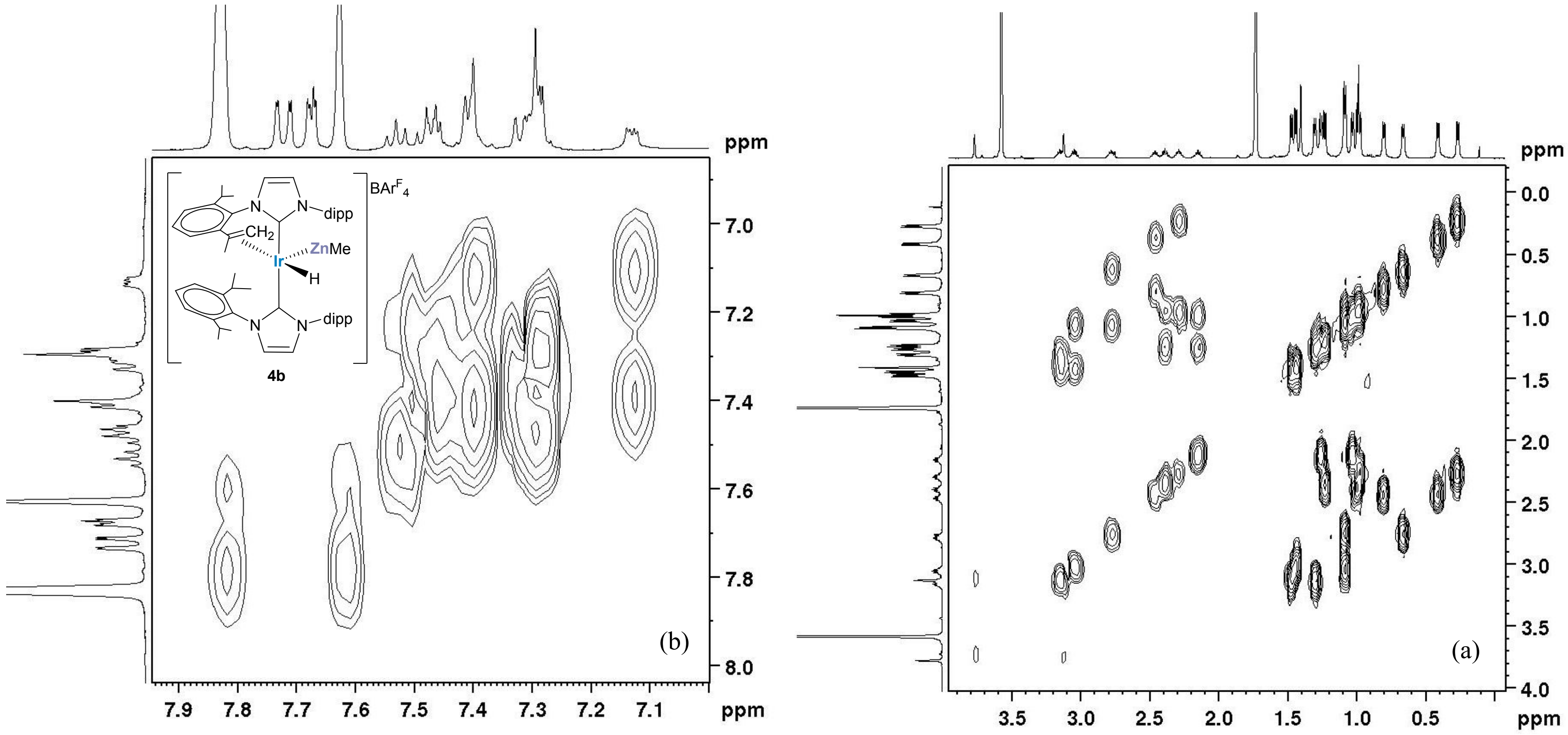

Figure S16. (a) Alkyl and (b) aryl regions of the ^{1}H COSY spectrum (THF-d_8, 500 MHz, 248 K) of [Ir(IPr)(IPr'')(ZnMe)H][BAr$^F_4$] (**4b**).

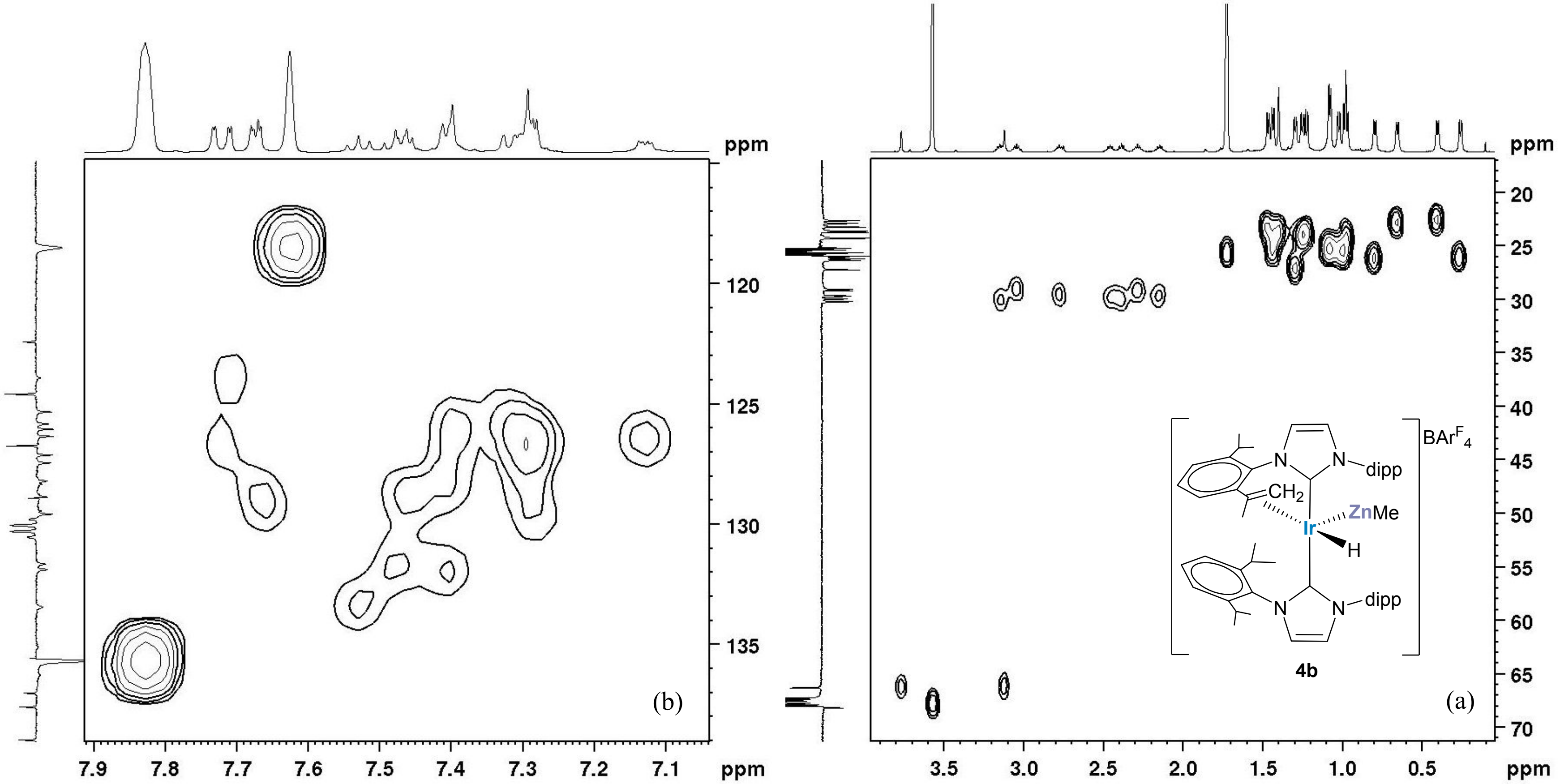

Figure S17. (a) Alkyl and (b) aryl regions of the ^{13}C-^{1}H HSQC spectrum (THF-d_8, 248 K) of [Ir(IPr)(IPr'')(ZnMe)H][BAr$^{F}_{4}$] (**4b**).

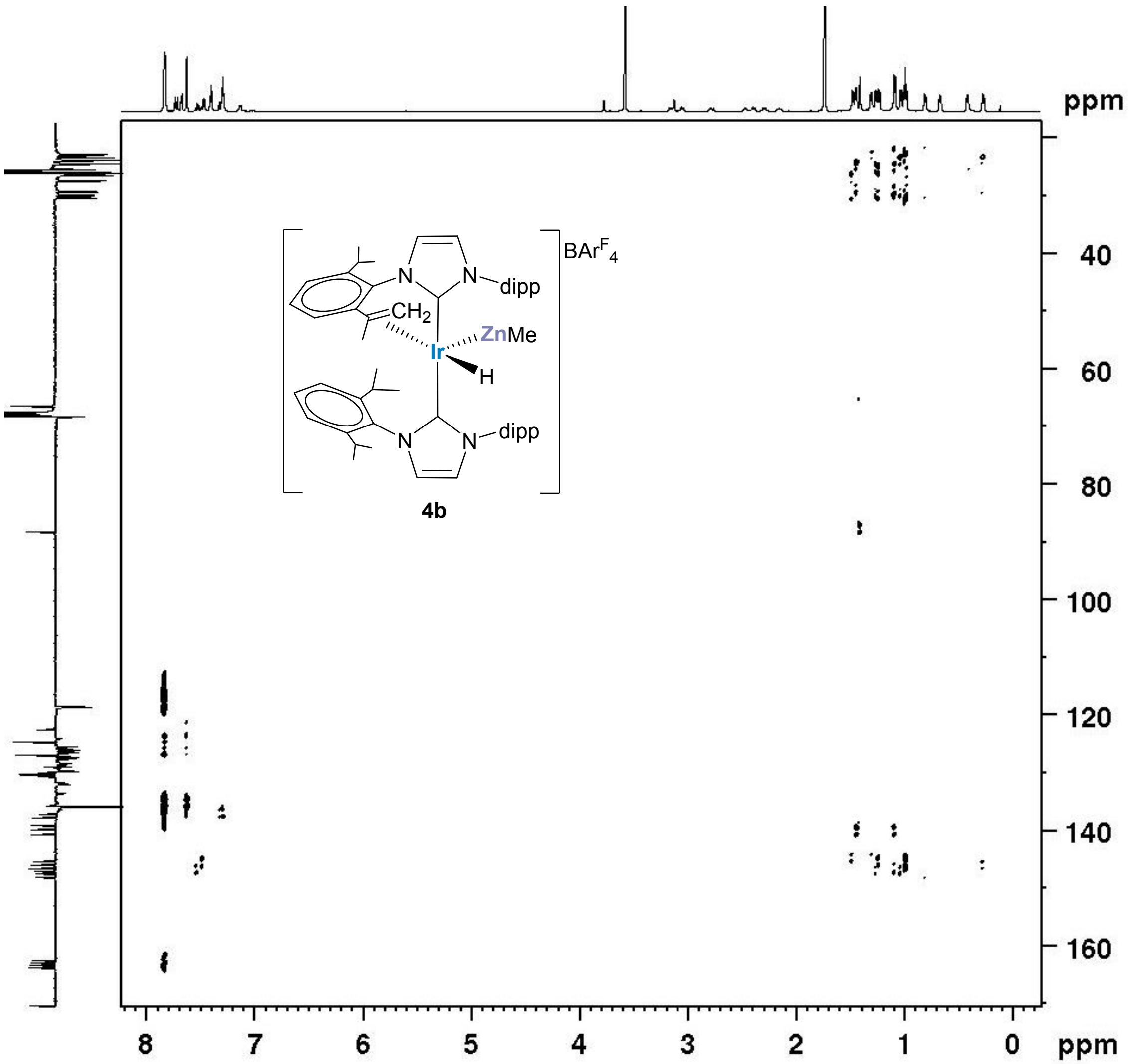

Figure S18. ^{13}C-^{1}H HMBC spectrum (THF-d_8, 248 K) of [Ir(IPr)(IPr'')(ZnMe)H][BAr$^{F}_{4}$] (**4b**).

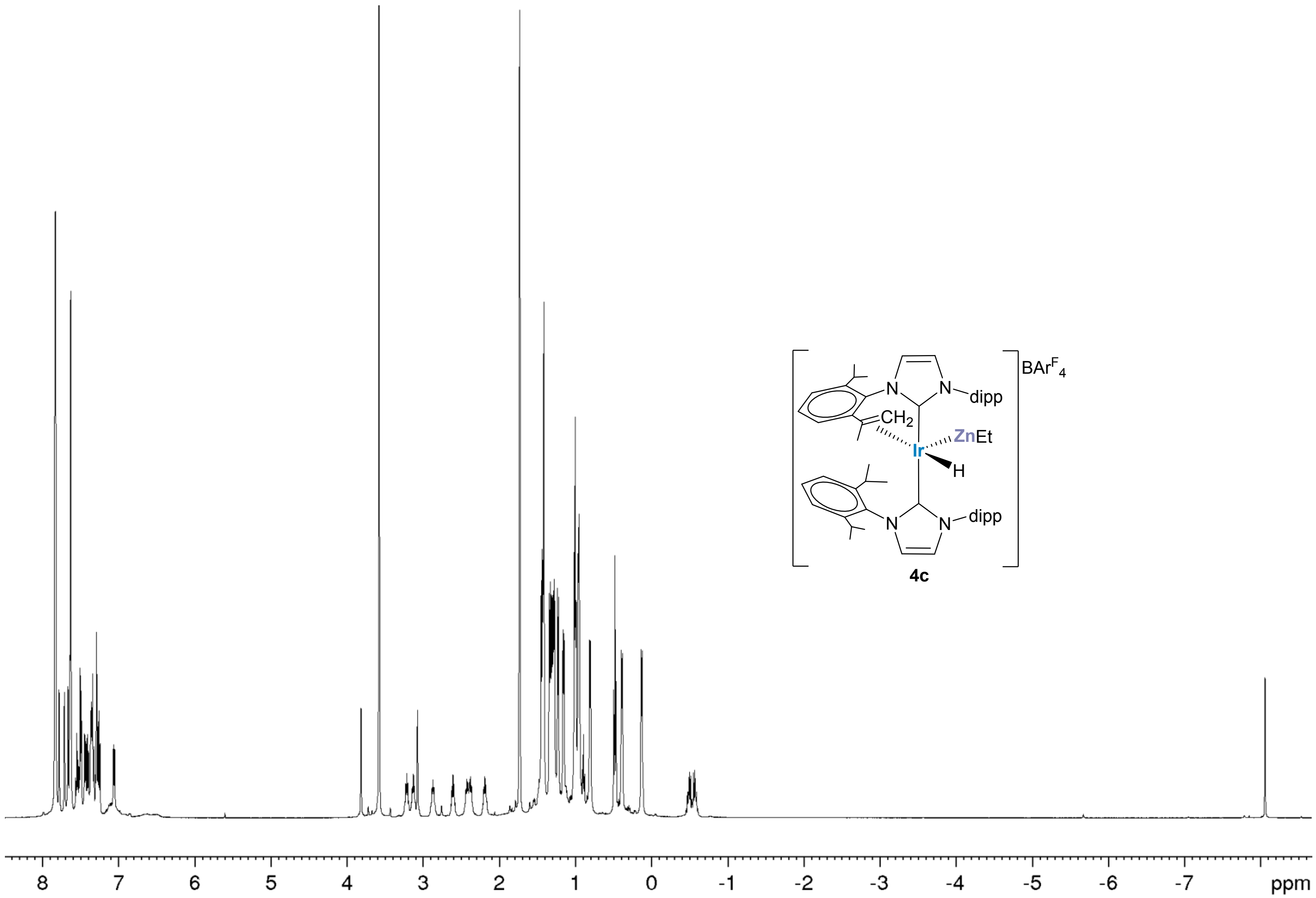

Figure S19. ^{1}H NMR spectrum (THF-d_8, 500 MHz, 248 K) of [Ir(IPr)(IPr'')(ZnEt)H][BAr$^F_4$] (**4c**).

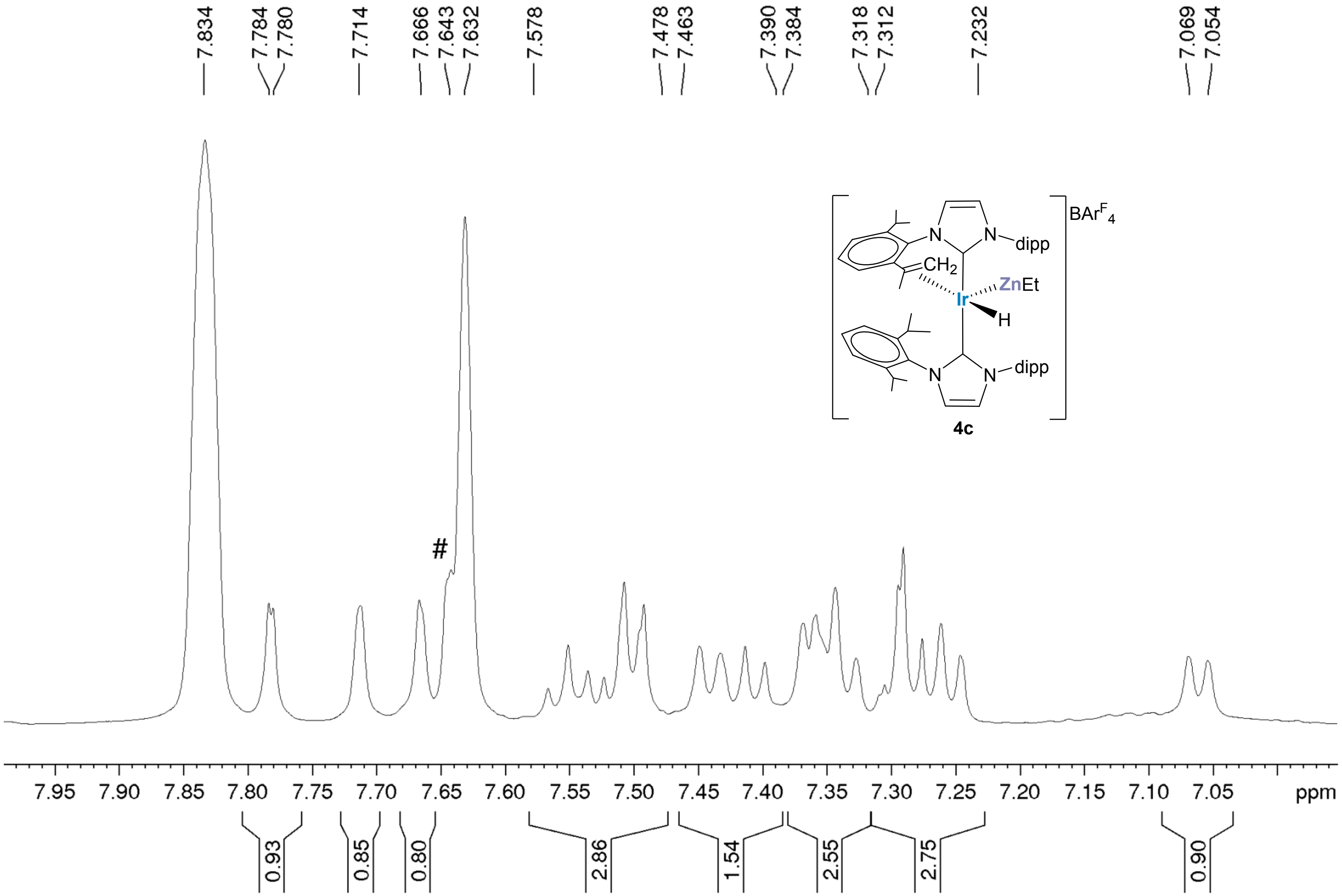

Figure S20. Aromatic region of the ^{1}H NMR spectrum (THF-d_8, 500 MHz, 248 K) of [Ir(IPr)(IPr'')(ZnEt)H][BAr$^{F}{}_{4}$] (**4c**). (# denotes one NC*H*=CHN resonance partially obscured by BAr$^{F}{}_{4}$).

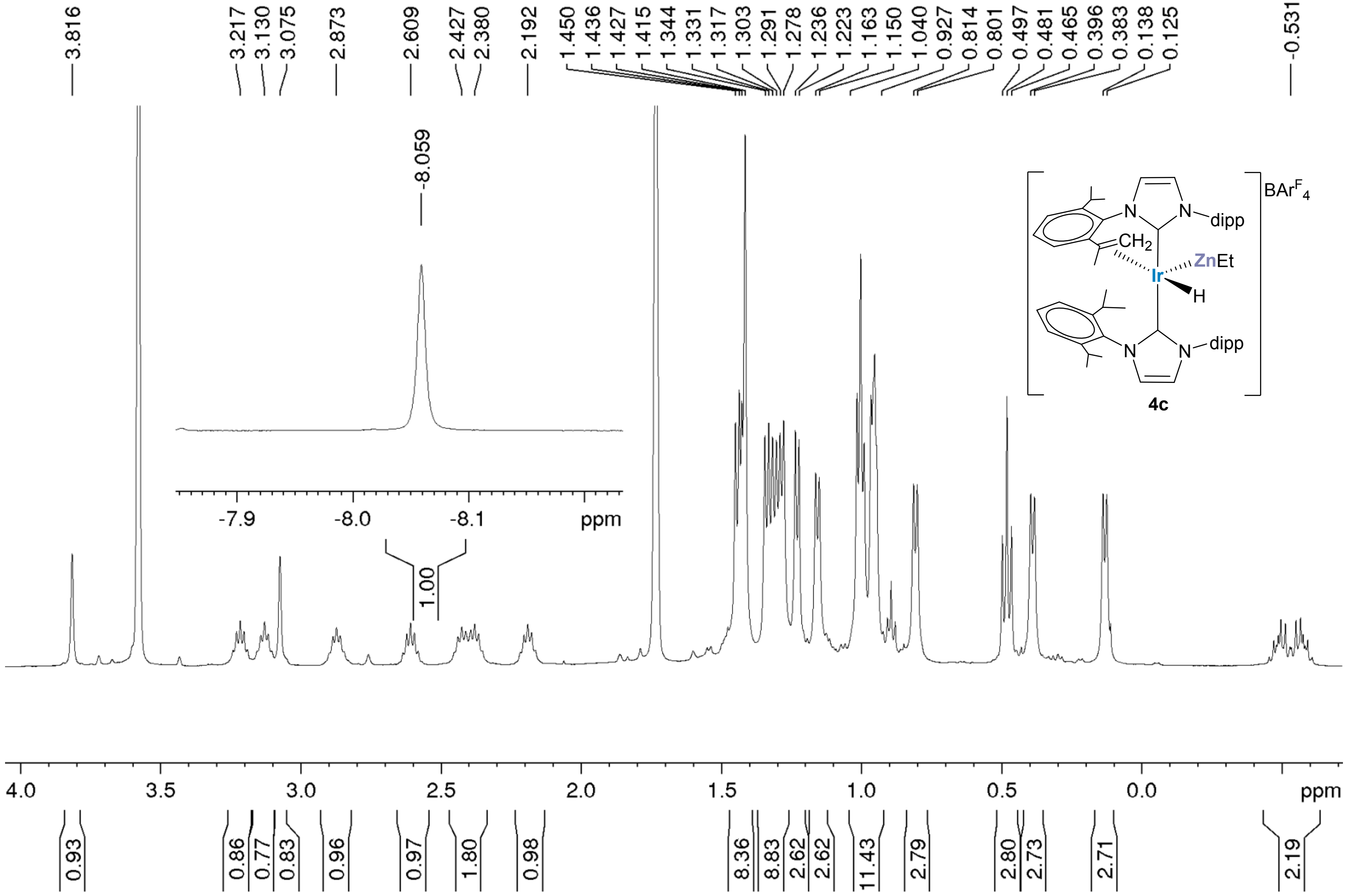

Figure S21. Alkyl region of the ^{1}H NMR spectrum (THF-d_8, 500 MHz, 248 K) of [Ir(IPr)(IPr'')(ZnEt)H][BAr$^F_4$] (**4c**) with inset of the Ir–*H* resonance.

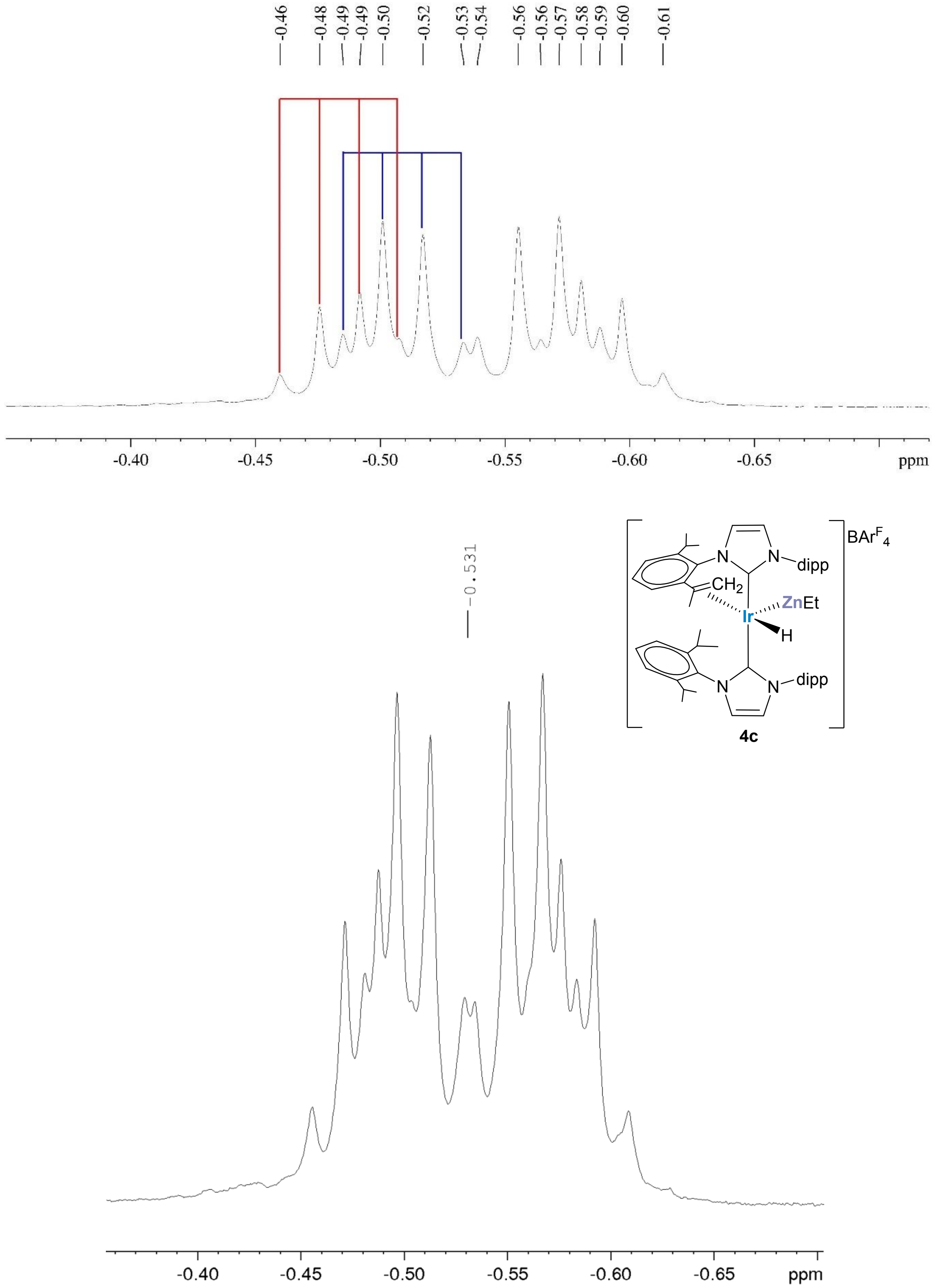

Figure S22. ^{1}H NMR resonance (THF-d_8, 500 MHz, 248 K) of the diastereotopic Zn–CH_2 protons in the [Ir(IPr)(IPr'')(ZnEt)H][BAr$^F{}_4$] (**4c**); top diagram is a schematic to highlight the splitting pattern within the multiplet.

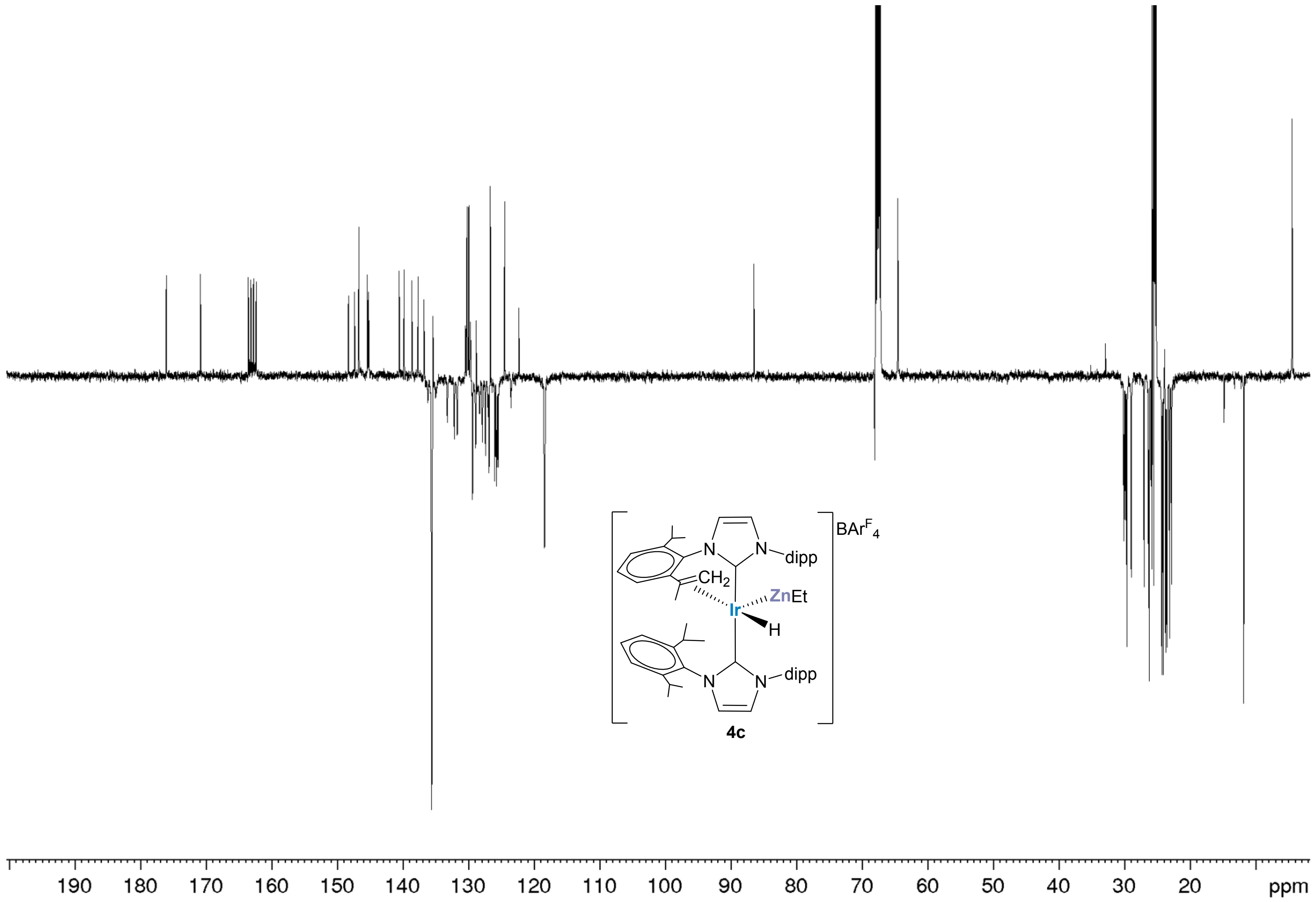

Figure S23. $^{13}C\{^{1}H\}$ DEPTQ NMR spectrum (THF-d_8, 126 MHz, 248 K) of [Ir(IPr)(IPr'')(ZnEt)H][BAr$^F{}_4$] (**4c**).

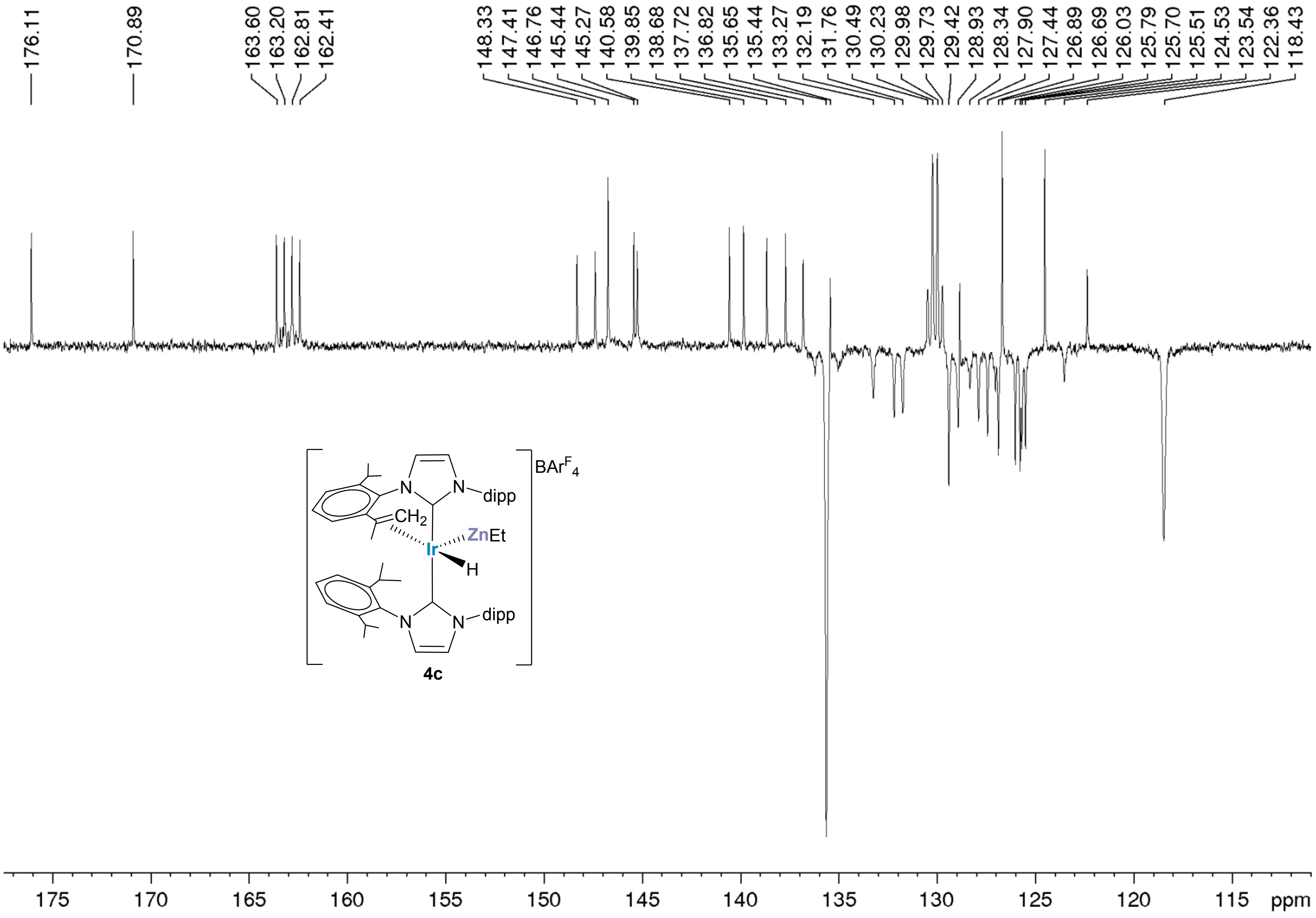

Figure S24. Aromatic region of the $^{13}C\{^{1}H\}$ DEPTQ NMR spectrum (THF-d_8, 126 MHz, 248 K) of [Ir(IPr)(IPr'')(ZnEt)H][BAr$^F{}_4$] (**4c**).

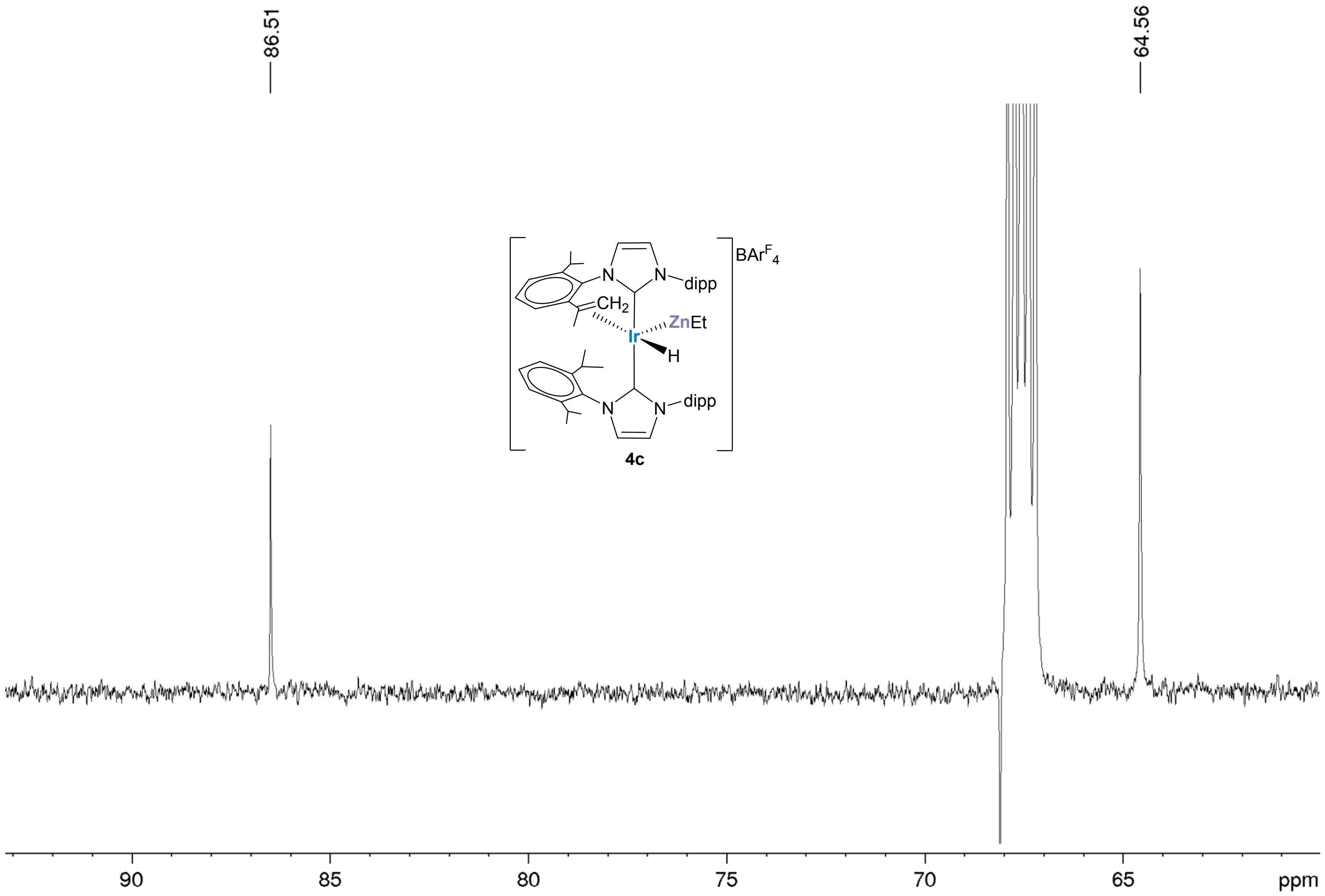

Figure S25. Alkenic region of the $^{13}C\{^1H\}$ DEPTQ NMR spectrum (THF-d_8, 126 MHz, 248 K) of [Ir(IPr)(IPr'')(ZnEt)H][BAr$^F_4$] (**4c**).

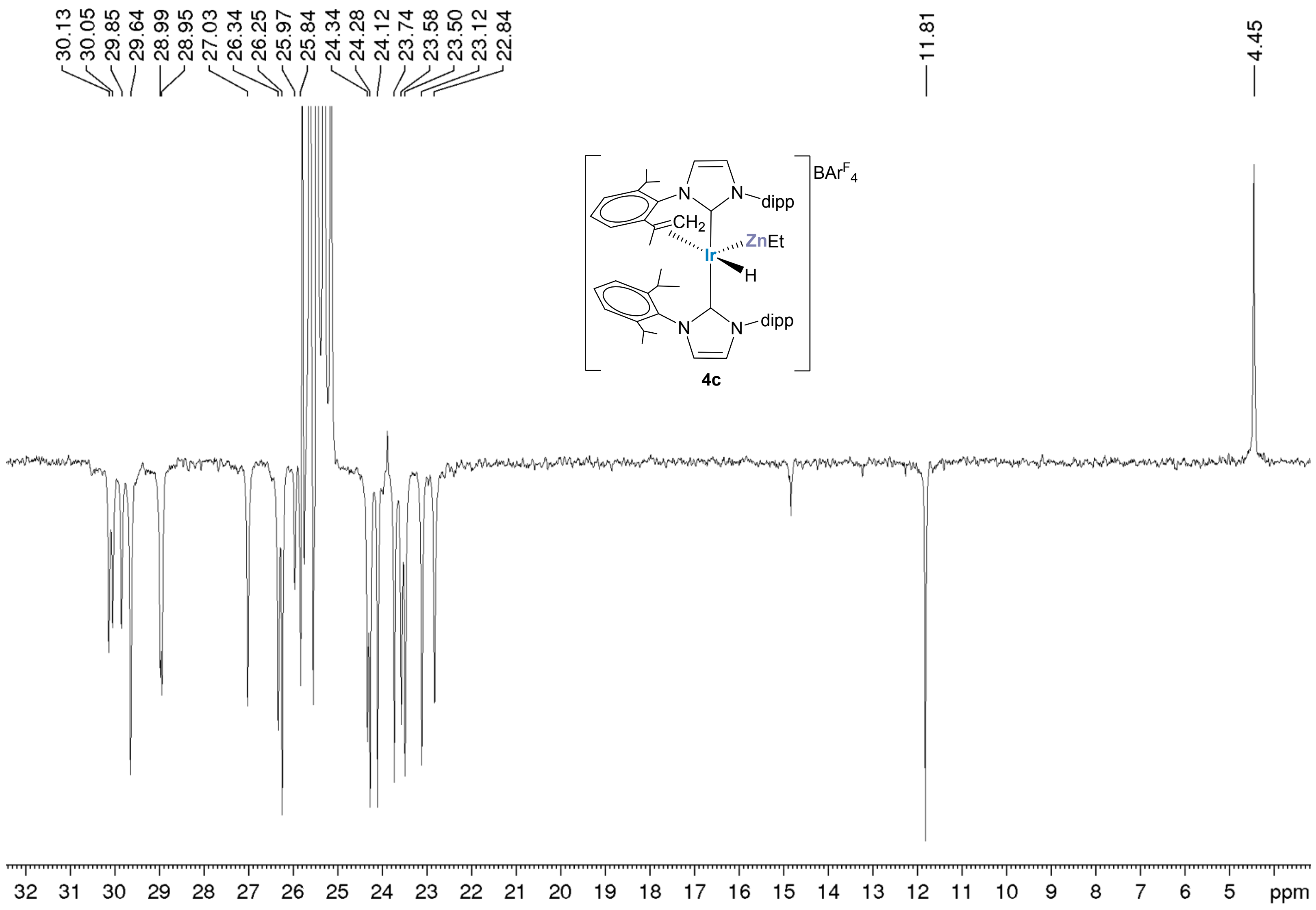

Figure S26. Low frequency region of the $^{13}C\{^1H\}$ DEPTQ NMR spectrum (THF-d_8, 126 MHz, 248 K) of [Ir(IPr)(IPr'')(ZnEt)H][BAr$^F_4$] (**4c**).

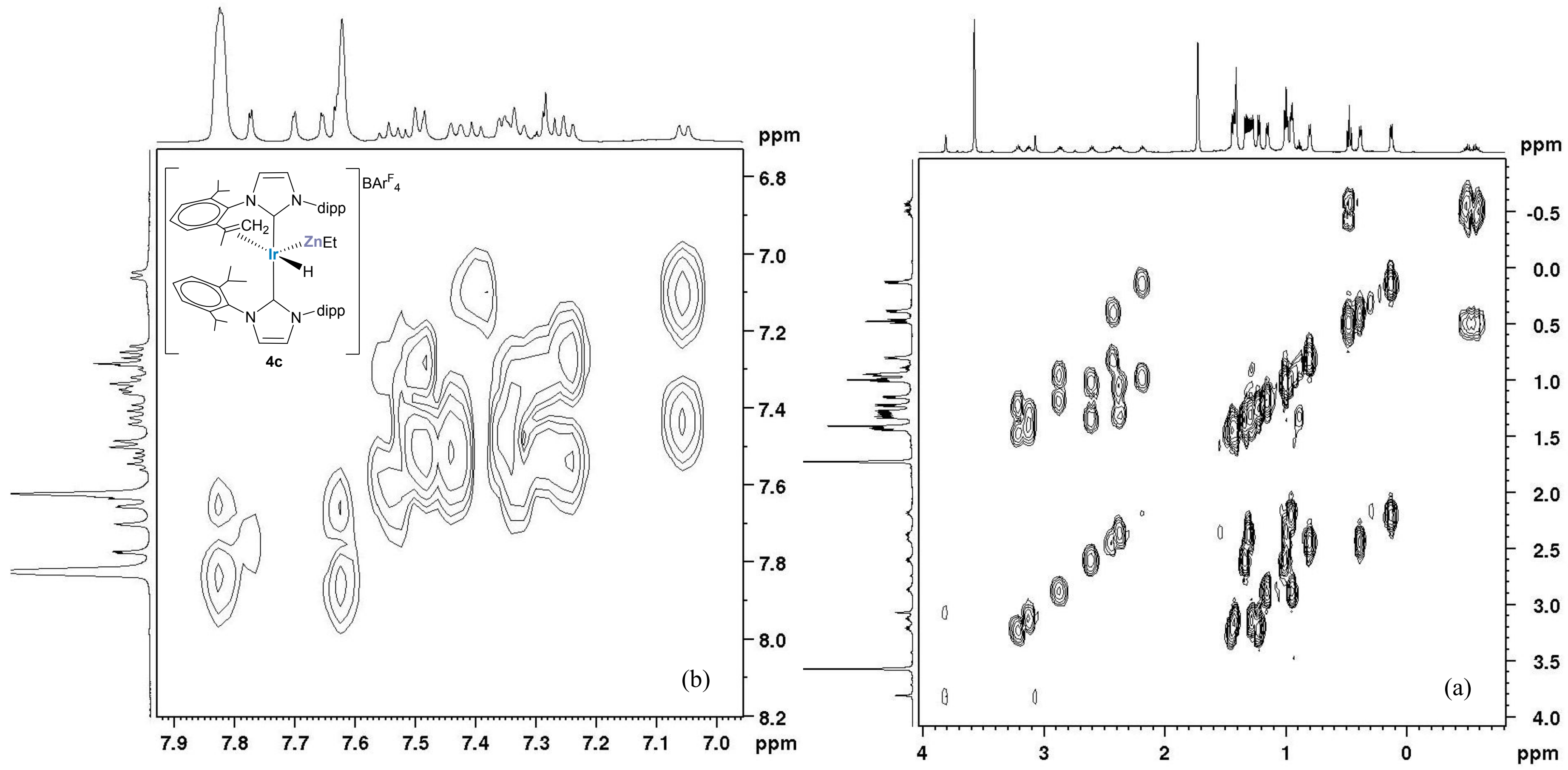

Figure S27. (a) Alkyl and (b) aryl regions of the ^{1}H COSY spectrum (THF-d_8, 500 MHz, 248 K) of [Ir(IPr)(IPr'')(ZnEt)H][BAr$^F_4$] (**4c**).

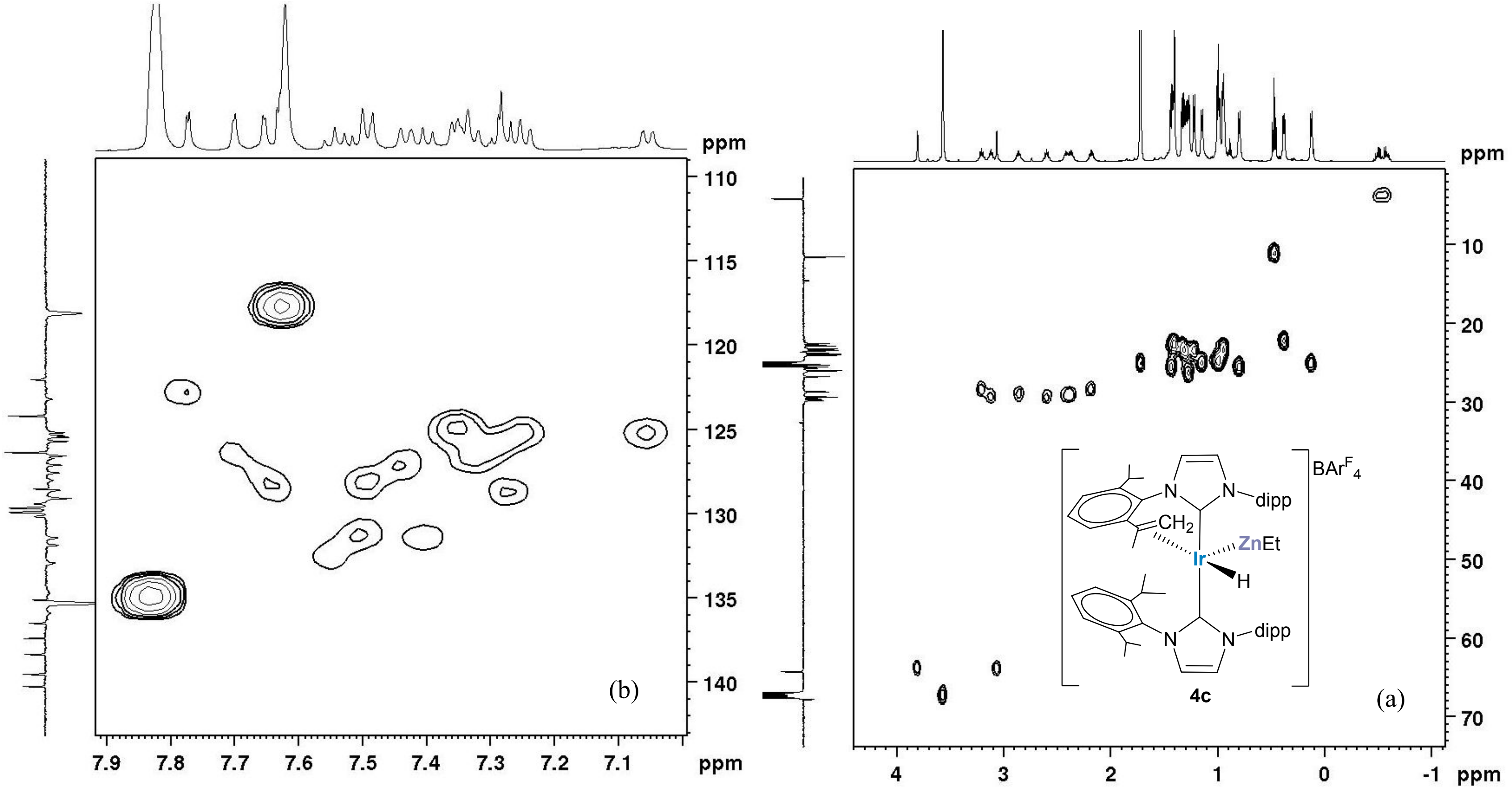

Figure S28. (a) Alkyl and (b) aryl regions of the ^{13}C-^{1}H HSQC spectrum (THF-d_8, 248 K) of [Ir(IPr)(IPr'')(ZnEt)H][BAr$^F{}_4$] (**4c**).

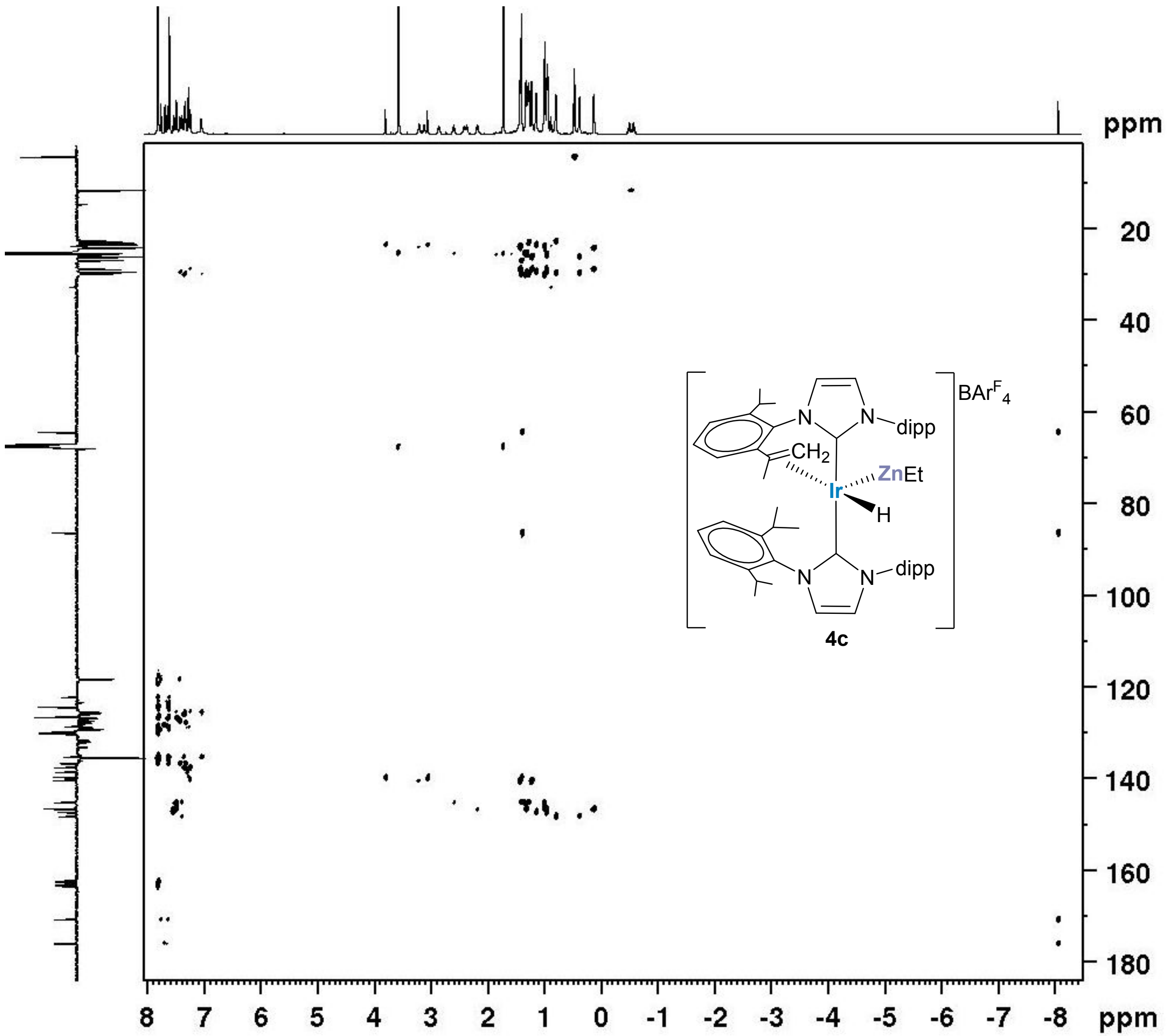

Figure S29. ^{13}C-^{1}H HMBC spectrum (THF-d_8, 248 K) of [Ir(IPr)(IPr'')(ZnEt)H][BAr^F$_4$] (**4c**).

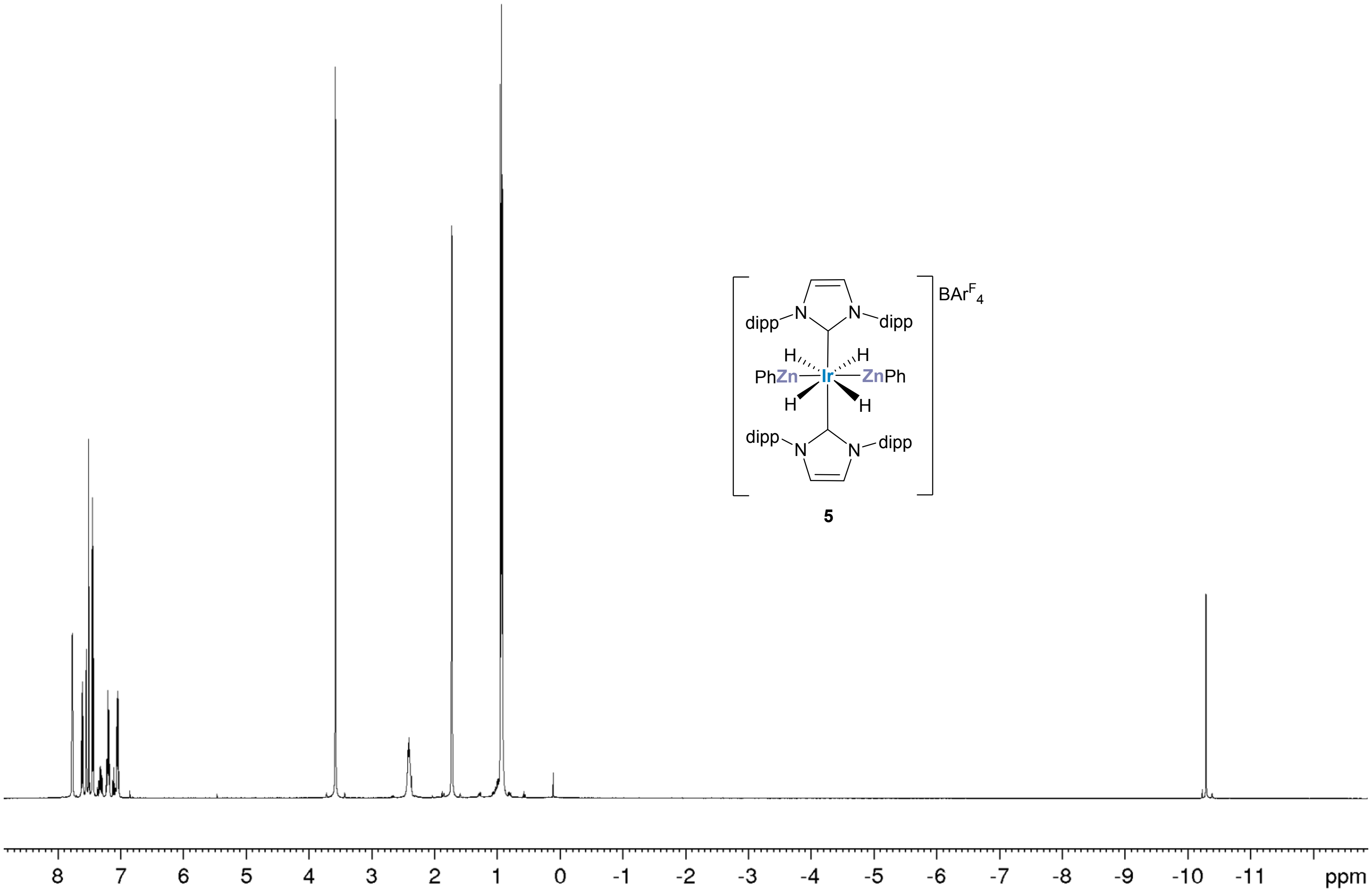

Figure S30. ^{1}H NMR spectrum (THF-d_8, 500 MHz, 323 K) of [Ir(IPr)$_2$(ZnPh)$_2$H$_4$][BAr$^F{}_4$] (**5**).

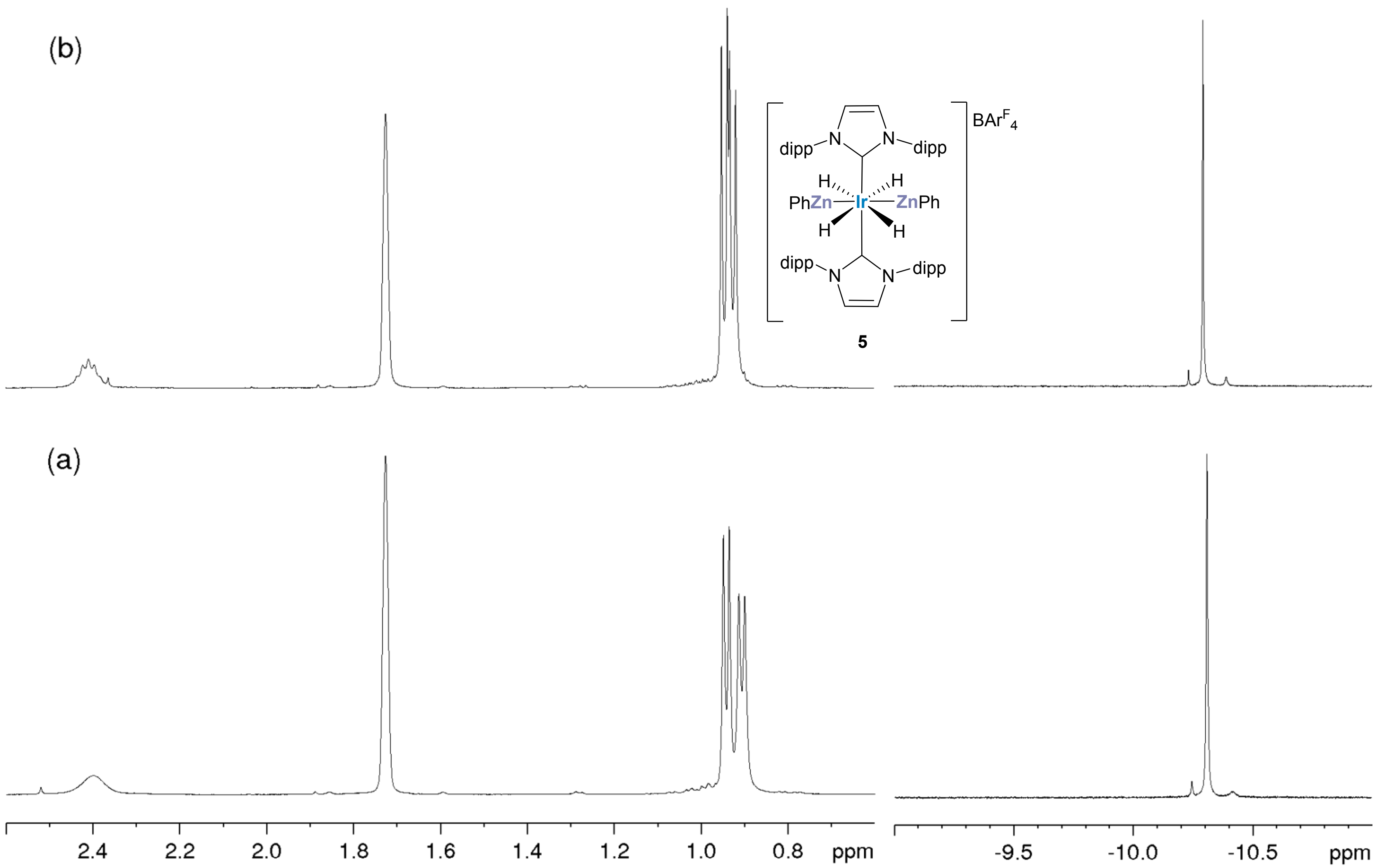

Figure S31. Alkyl (left) and hydride (right) ^{1}H NMR resonances (THF-d_8, 500 MHz) of $[Ir(IPr)_2(ZnPh)_2H_4][BAr^{F}_4]$ (**5**) at (a) 298 K and (b) 323 K.

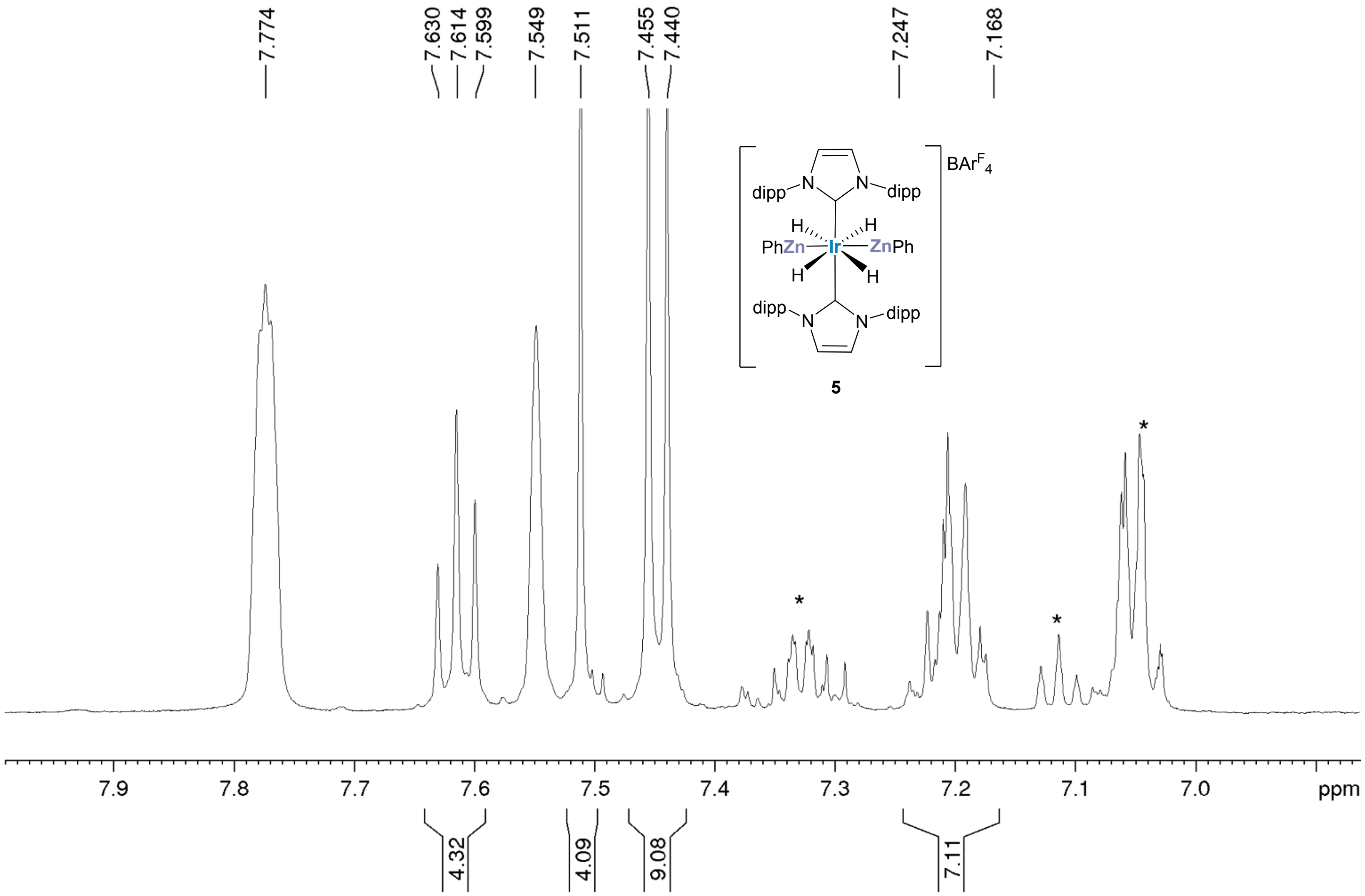

Figure S32. Aromatic region of the ^{1}H NMR spectrum (THF-d_8, 500 MHz, 323 K) of $[Ir(IPr)_2(ZnPh)_2H_4][BAr^F{}_4]$ (**5**) (* = residual C_6H_5F).

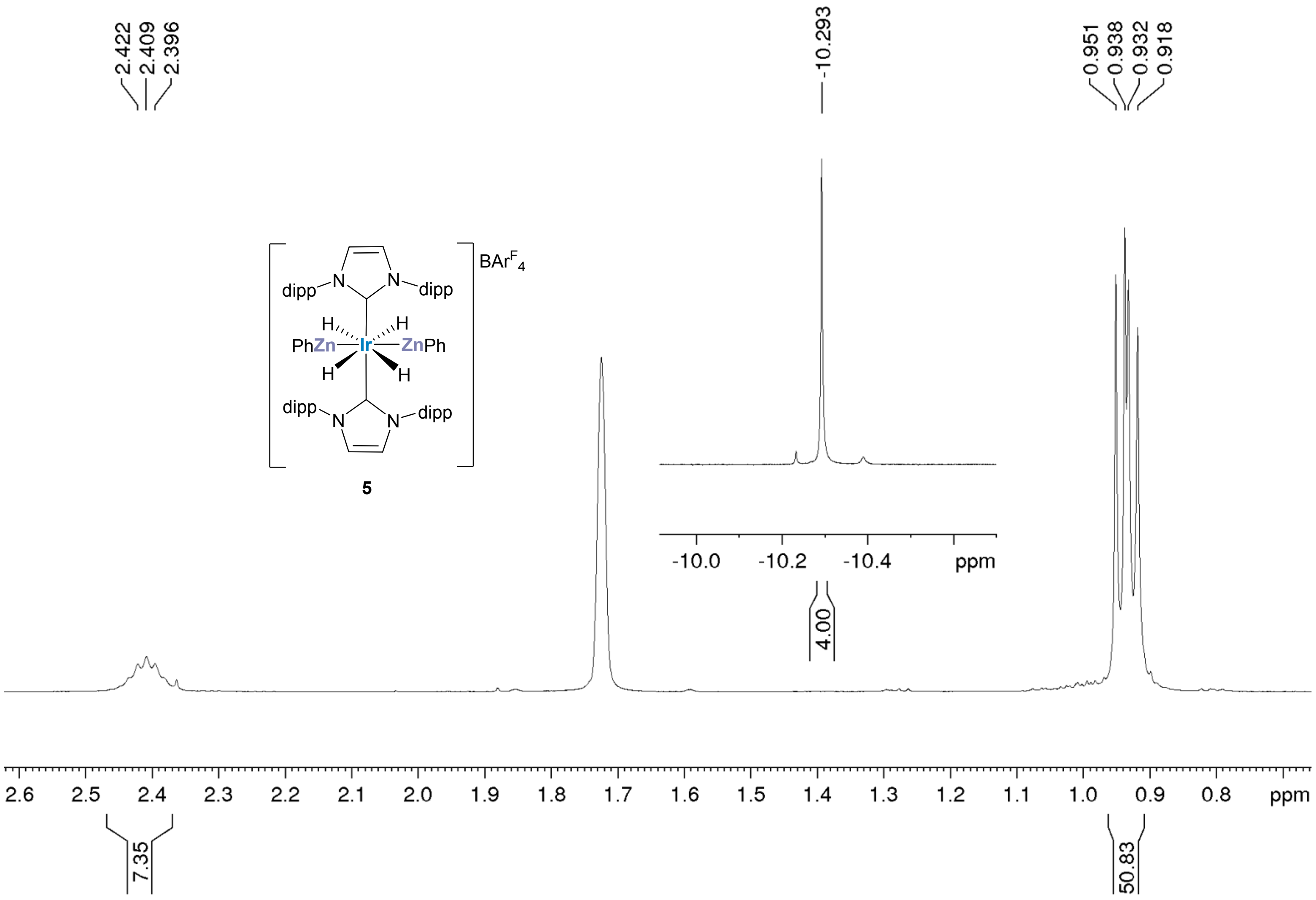

Figure S33. Alkyl region of the ^{1}H NMR spectrum (THF-d_8, 500 MHz, 323 K) of $[Ir(IPr)_2(ZnPh)_2H_4][BAr^F{}_4]$ (**5**), with Ir–*H* resonance shown in inset.

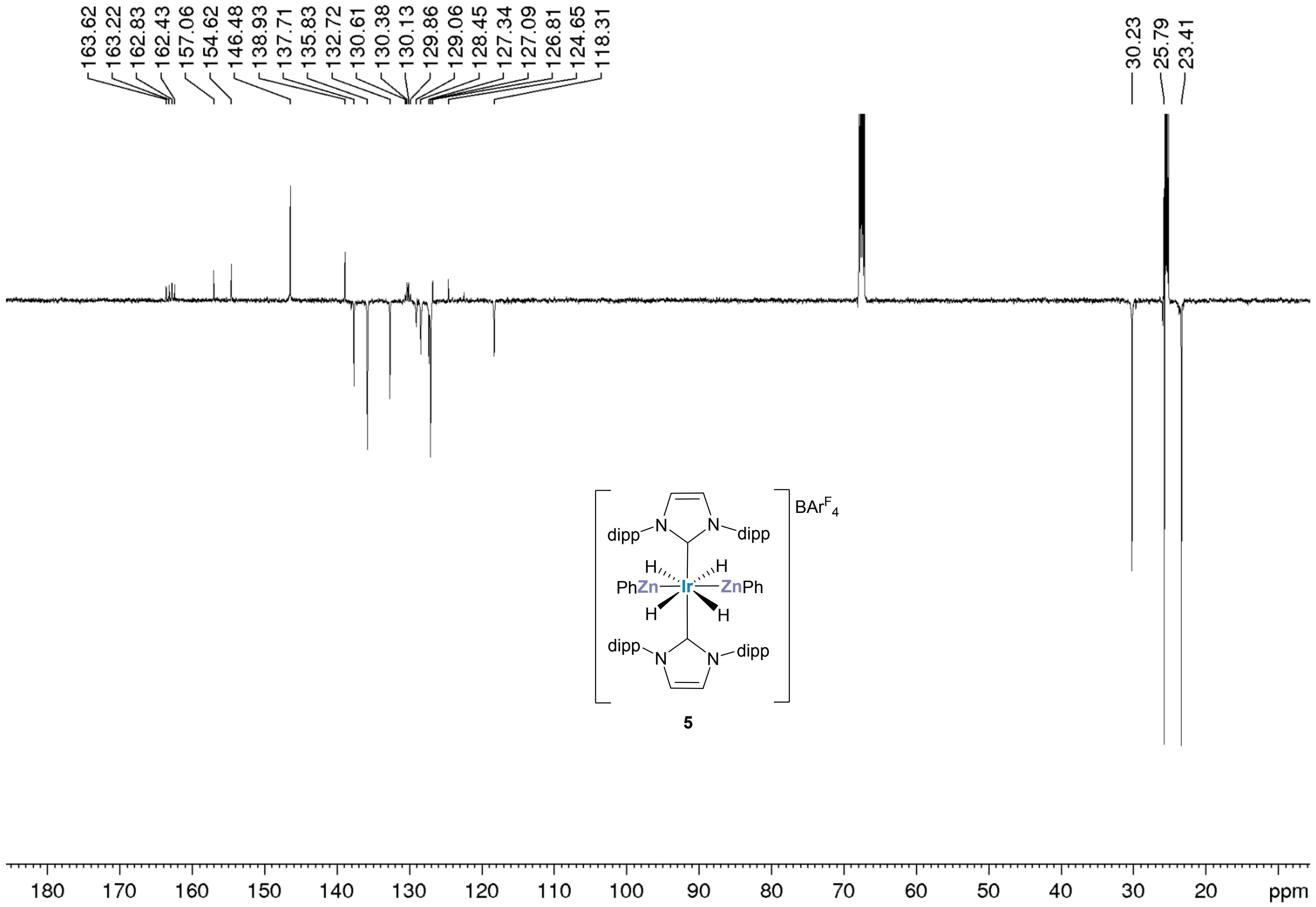

Figure S34. $^{13}C\{^{1}H\}$ DEPTQ NMR spectrum (THF-d_8, 126 MHz, 323 K) of $[Ir(IPr)_2(ZnPh)_2H_4][BAr^F{}_4]$ (**5**).

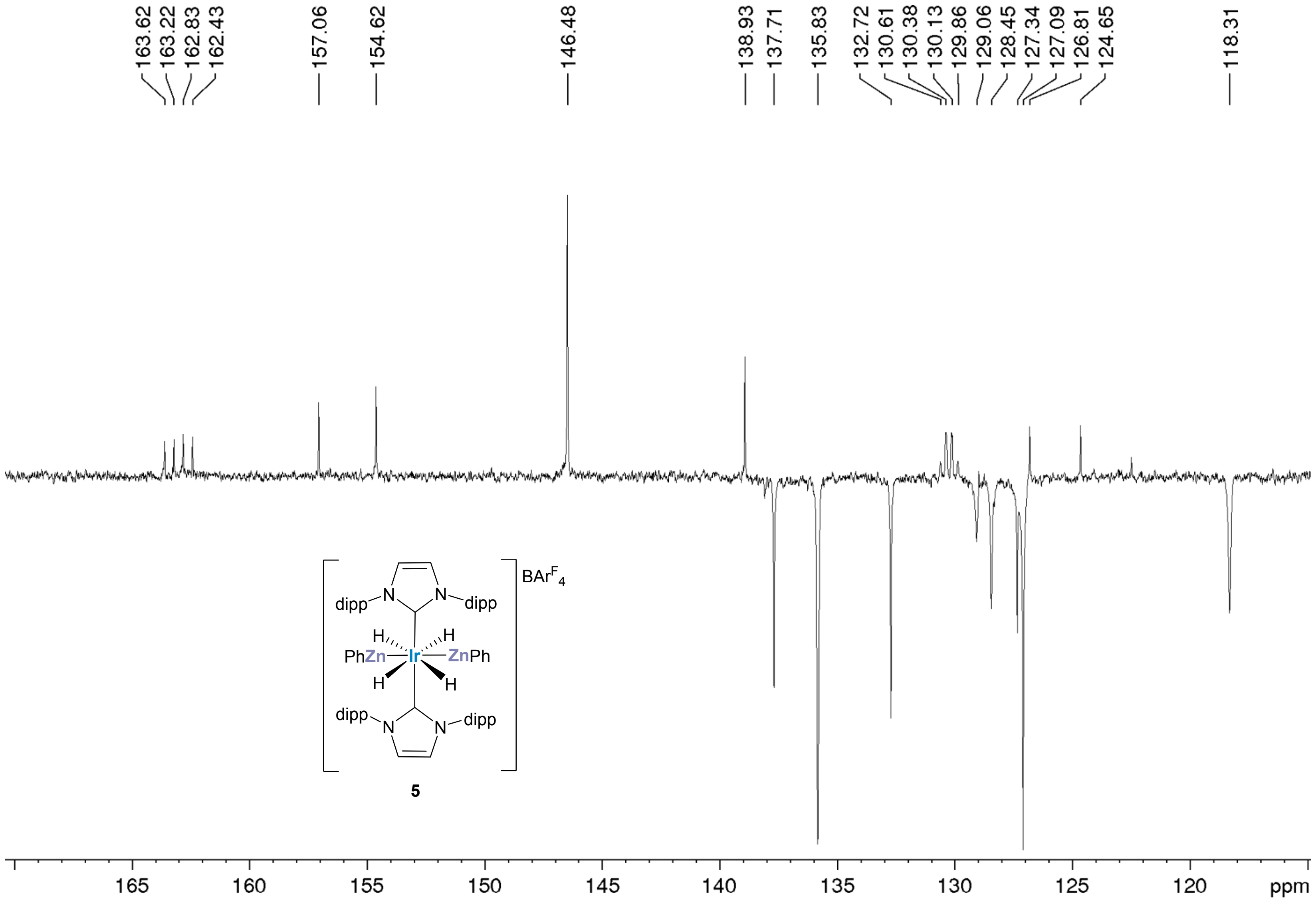

Figure S35. Aromatic region of the $^{13}C\{^{1}H\}$ DEPTQ NMR spectrum (THF-d_8, 126 MHz, 323 K) of $[Ir(IPr)_2(ZnPh)_2H_4][BAr^F{}_4]$ (**5**).

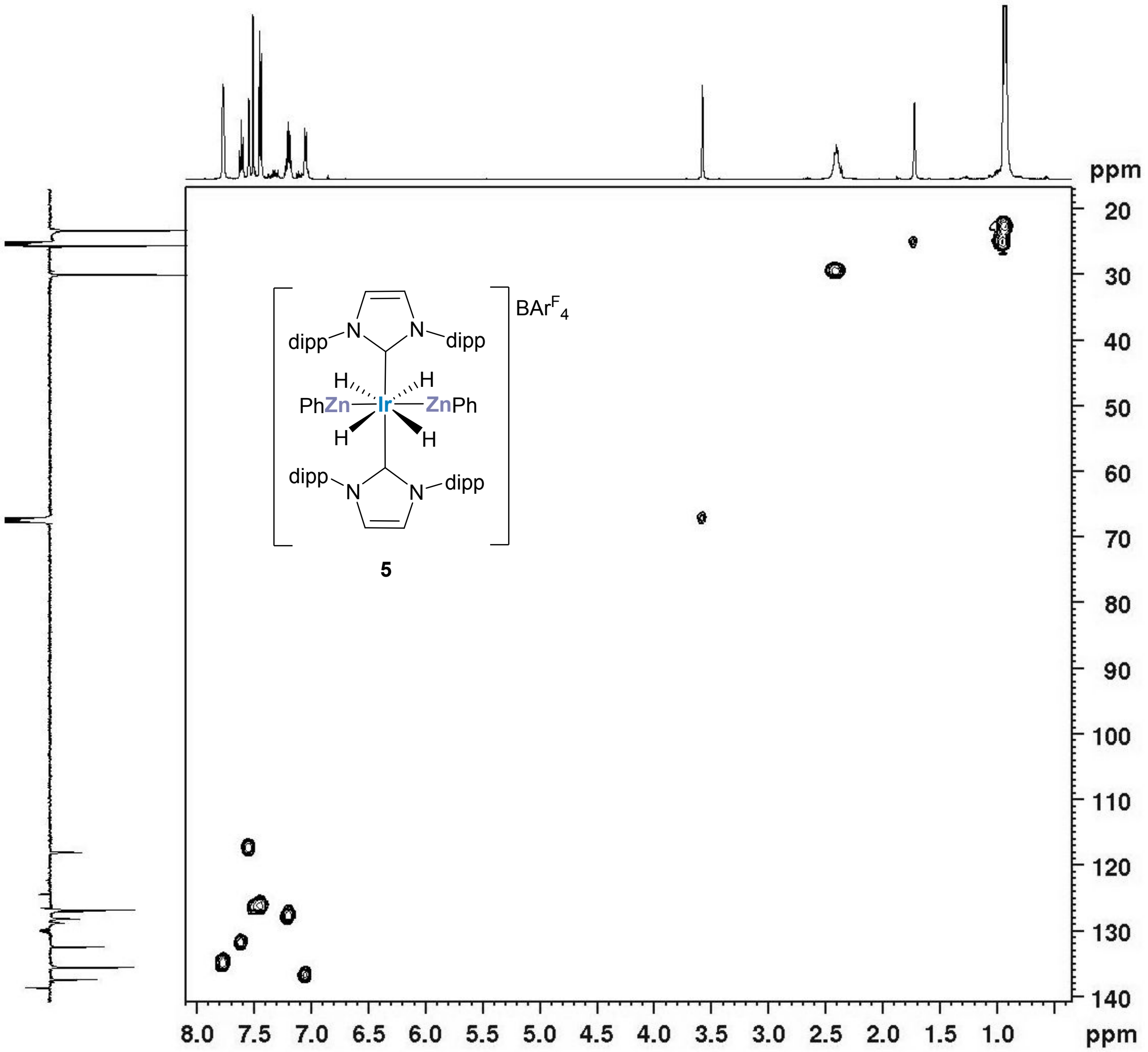

Figure S36. ^{13}C-^{1}H HSQC spectrum (THF-d_8, 323 K) of $[Ir(IPr)_2(ZnPh)_2H_4][BAr^F{}_4]$ (**5**).

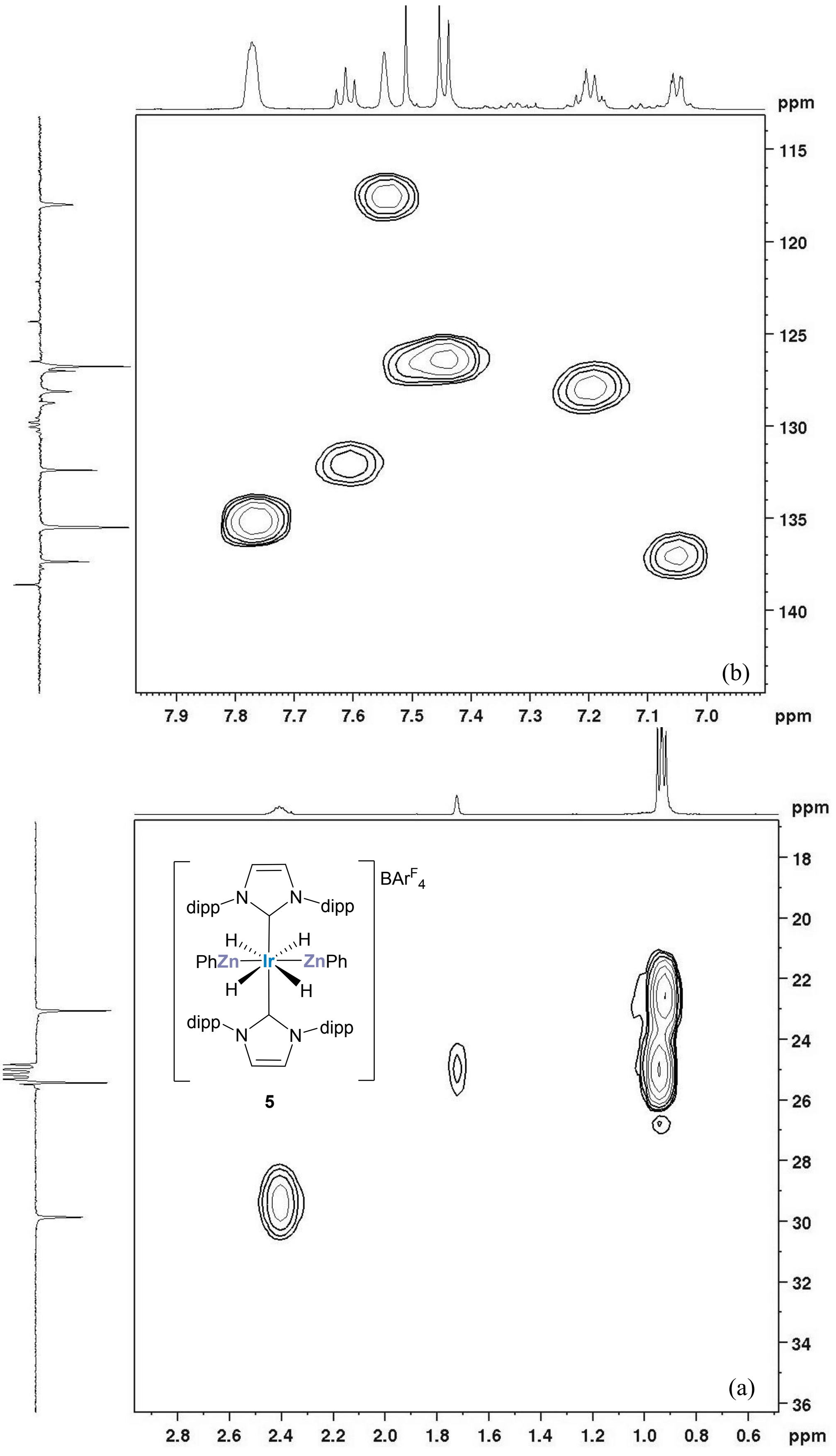

Figure S37. (a) Alkyl and (b) aryl regions of the ^{13}C-^{1}H HSQC spectrum (THF-d_8, 323 K) of $[Ir(IPr)_2(ZnPh)_2H_4][BAr^F{}_4]$ (**5**).

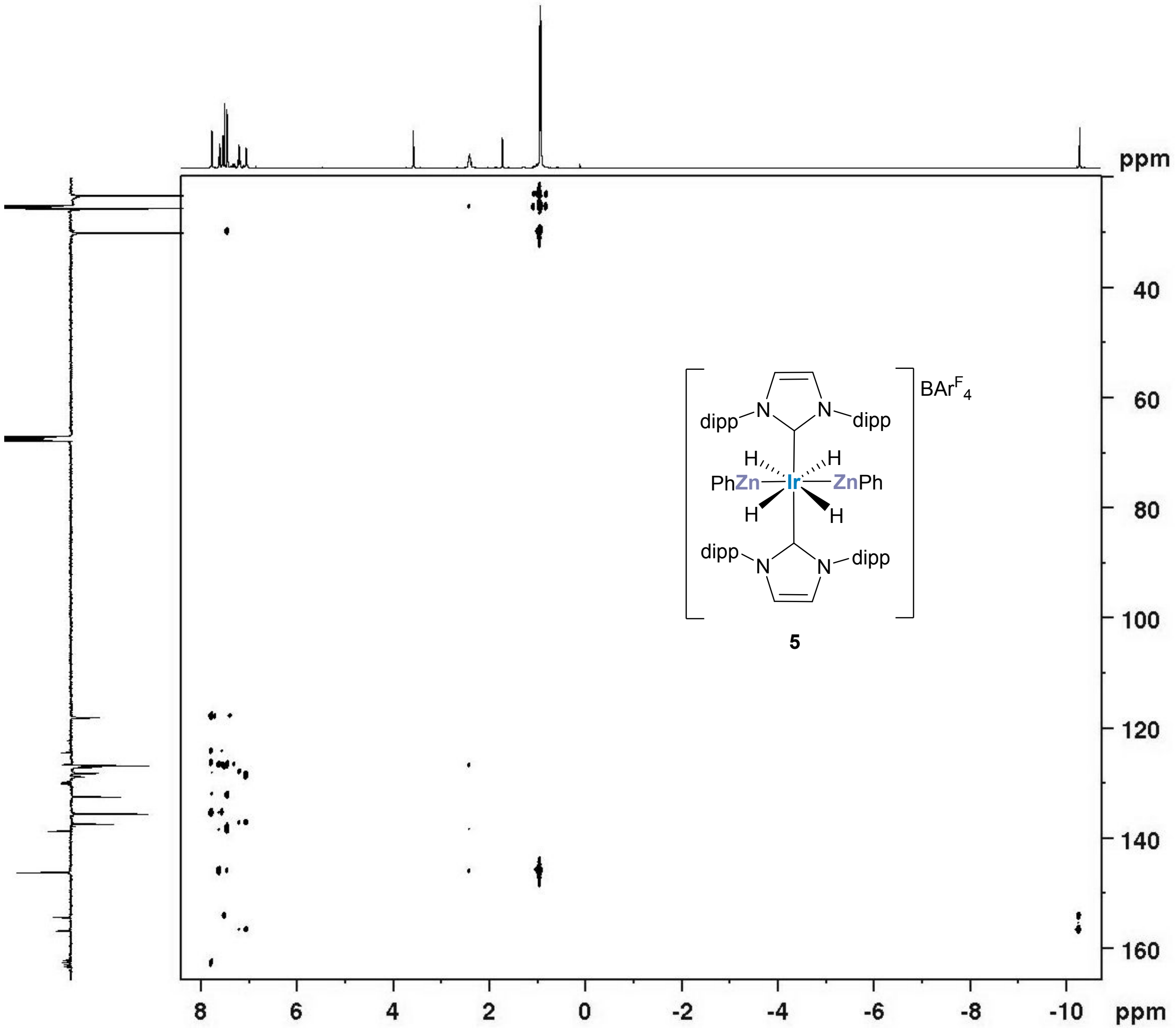

Figure S38. ^{13}C-^{1}H HMBC spectrum (THF-d_8, 323 K) of $[Ir(IPr)_2(ZnPh)_2H_4][BAr^F{}_4]$ (**5**).

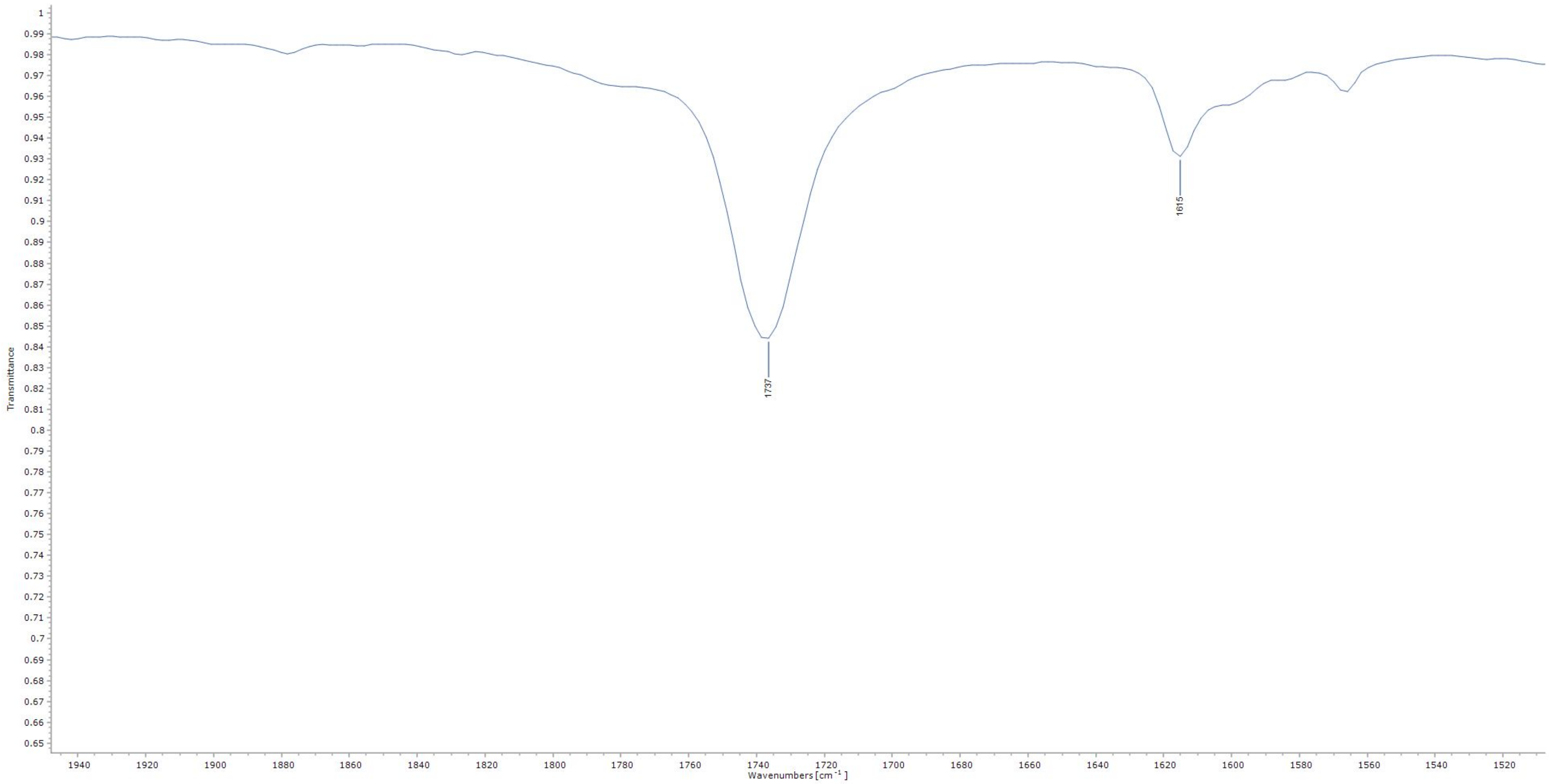

Figure S39. Hydride region of the ATR-IR spectrum of $[Ir(IPr)_2(ZnPh)_2H_4][BAr^F{}_4]$ (**5**).

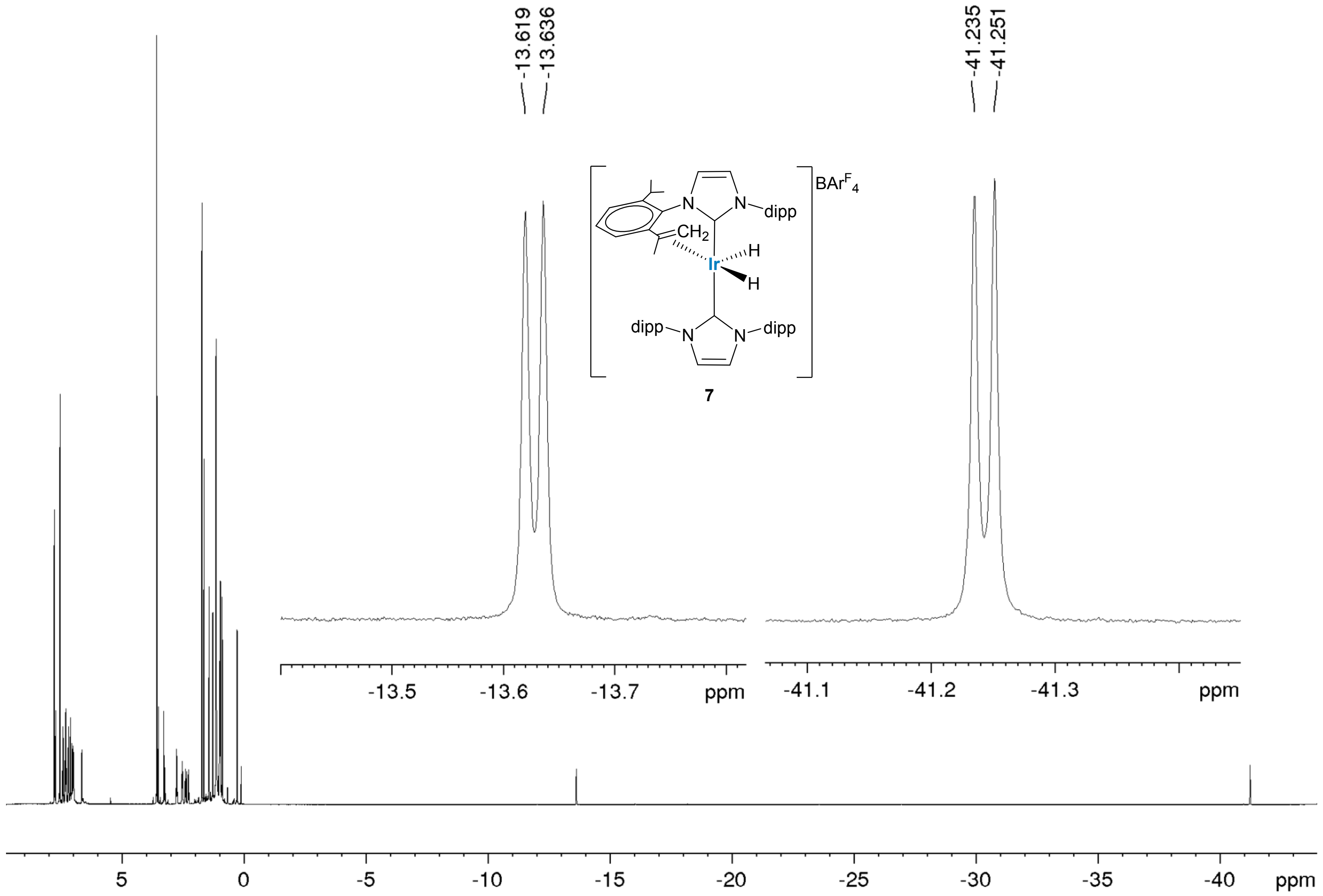

Figure S40. ^{1}H NMR spectrum (THF-d_8, 500 MHz, 318 K) of [Ir(IPr)(IPr'')H_2][BAr$^F_4$] (**7**). Insets show expansions of the two Ir–*H* resonances.

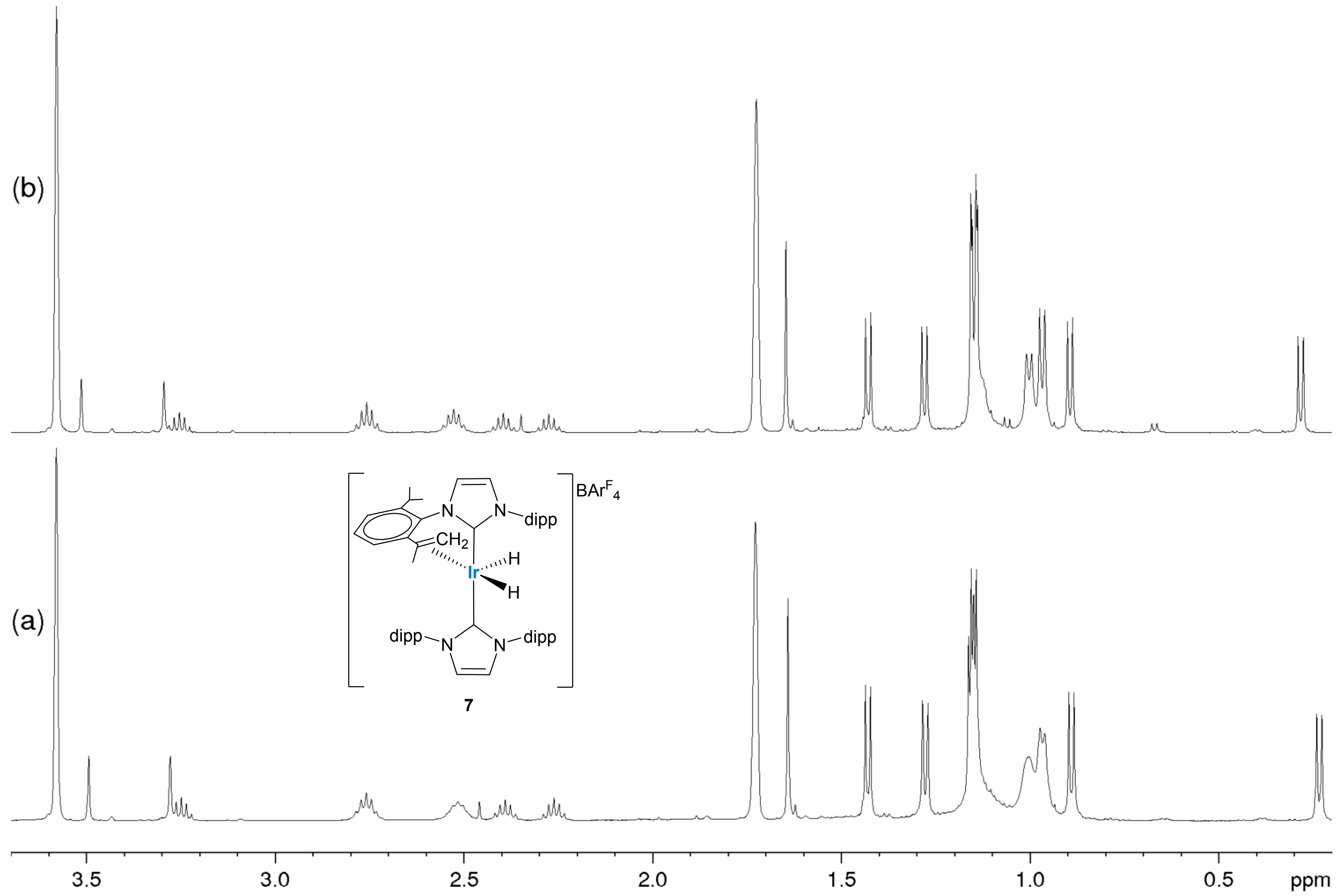

Figure S41. Alkyl region of the ^{1}H NMR spectrum (THF-d_8, 500 MHz) of [Ir(IPr)(IPr'')H_2][BAr$^F_4$] (**7**) at (a) 298 K and (b) 318 K.

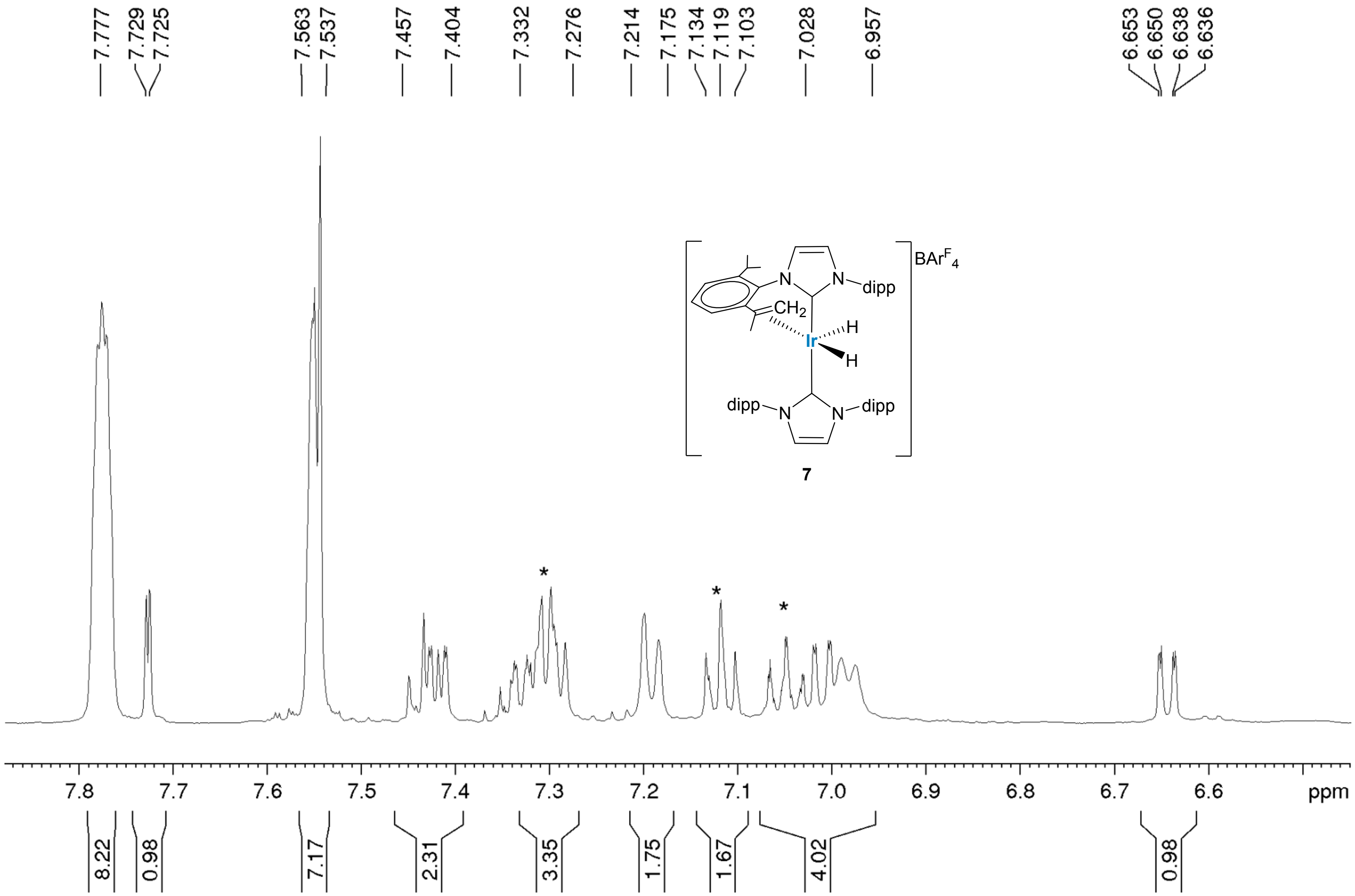

Figure S42. Aromatic region of the ^{1}H NMR spectrum (THF-d_8, 500 MHz, 318 K) of [Ir(IPr)(IPr'')H_2][BAr$^F_4$] (**7**) (* = residual C_6H_5F).

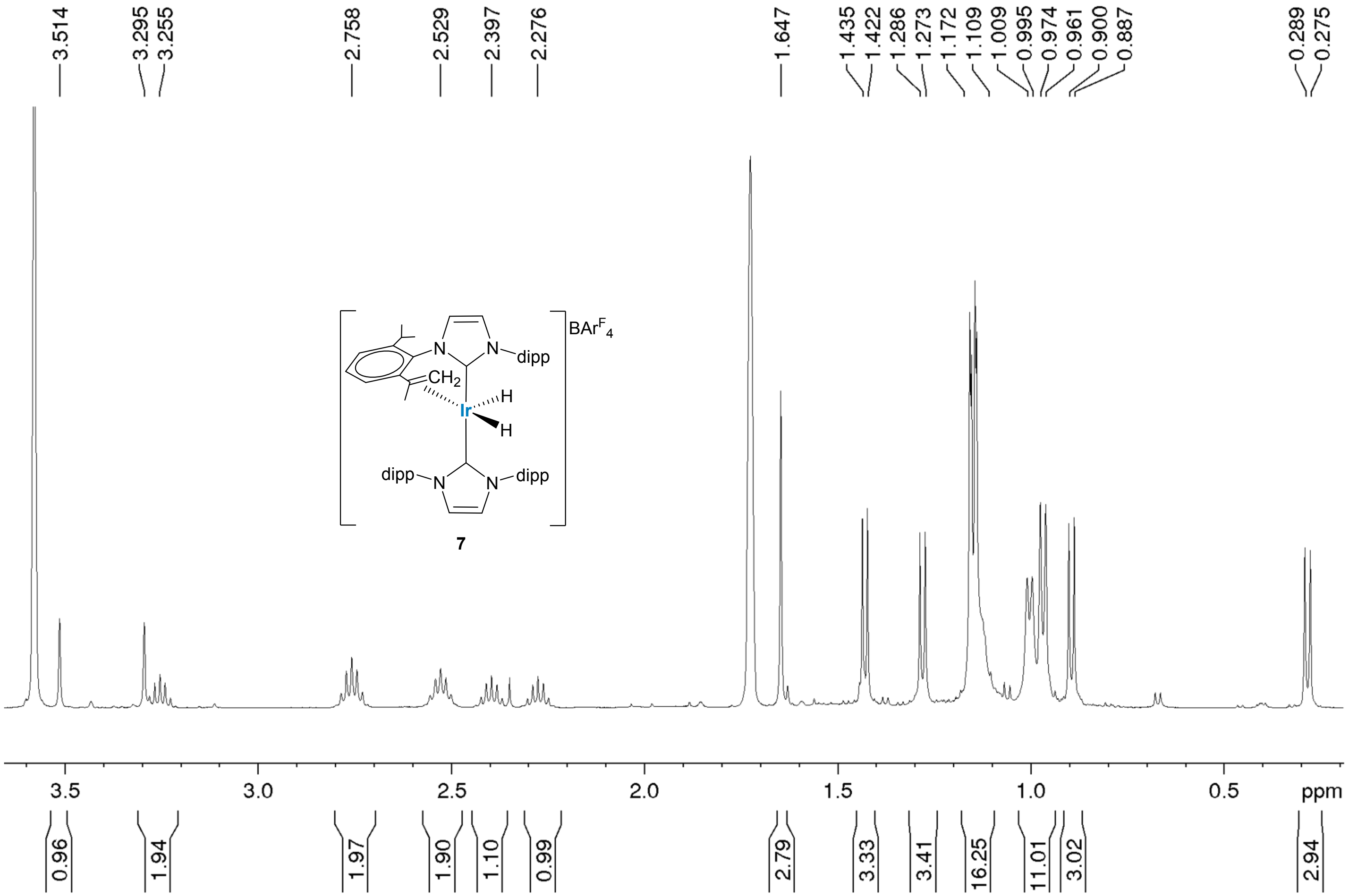

Figure S43. Alkyl region of the ^{1}H NMR spectrum (THF-d_8, 500 MHz, 318 K) of [Ir(IPr)(IPr'')H_2][BAr$^F_4$] (**7**).

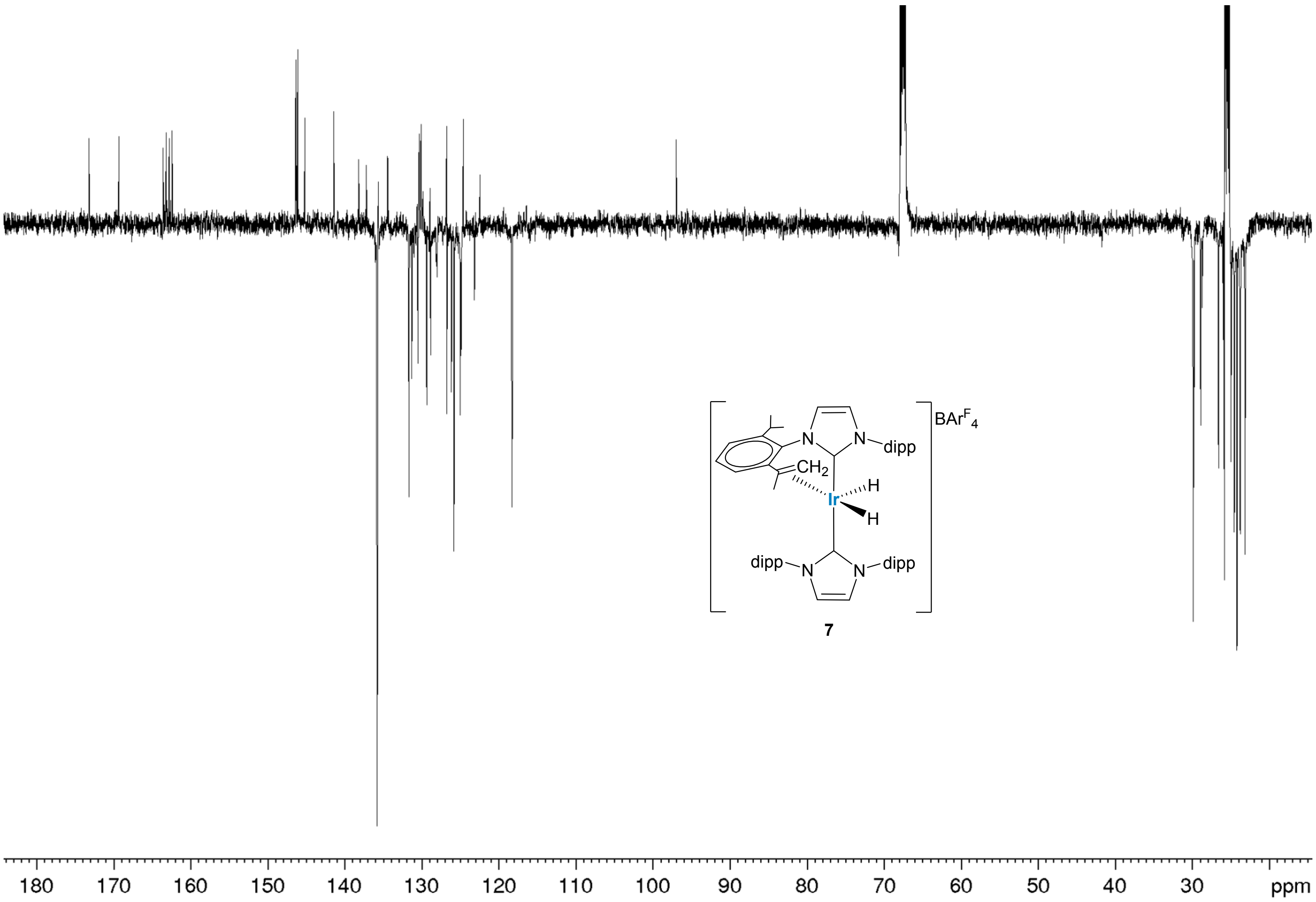

Figure S44. $^{13}C\{^{1}H\}$ DEPTQ NMR spectrum (THF-d_8, 126 MHz, 318 K) of [Ir(IPr)(IPr'')H_2][BAr^{F}_4] (**7**).

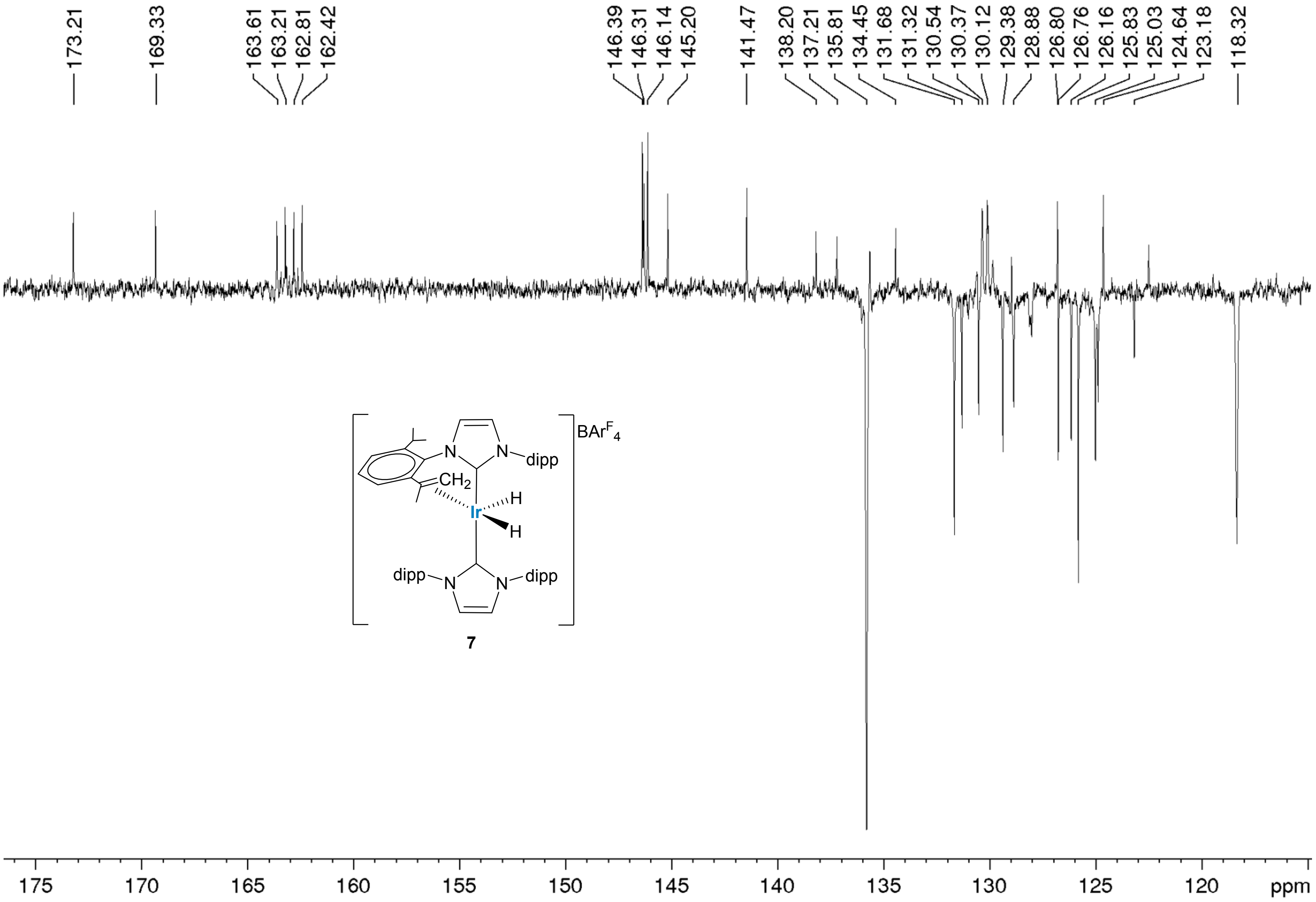

Figure S45. Aromatic region of the $^{13}C\{^{1}H\}$ DEPTQ NMR spectrum (THF-d_8, 126 MHz, 318 K) of [Ir(IPr)(IPr'')H_2][BAr$^F{}_4$] (**7**).

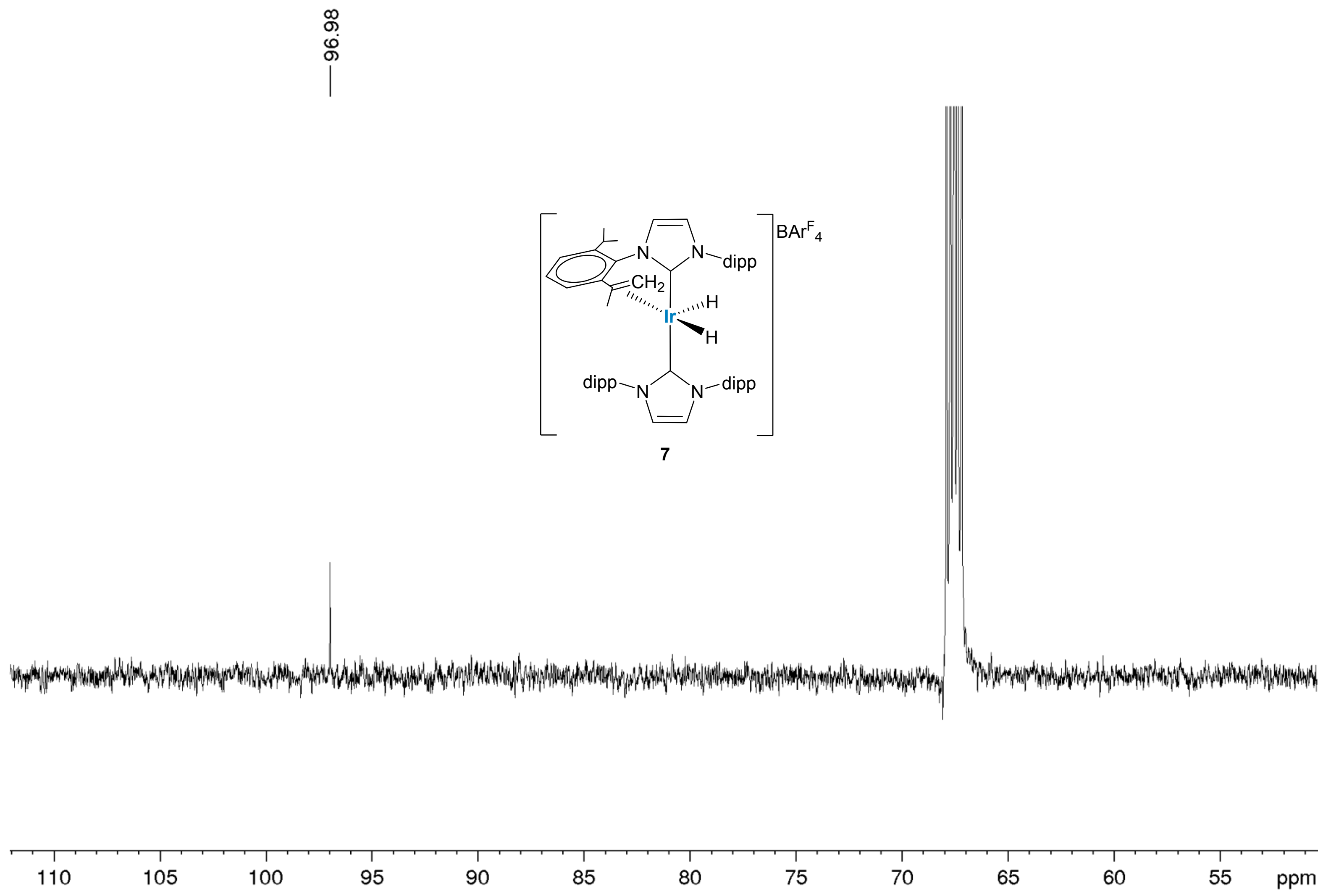

Figure S46. Alkenic region of the $^{13}C\{^1H\}$ DEPTQ NMR spectrum (THF-d_8, 126 MHz, 319 K) of [Ir(IPr)(IPr'')H_2][BAr$^F_4$] (**7**). The second alkenic carbon is obscured by THF, but identifiable from the ^{13}C-^1H HSQC spectrum (Figure S49).

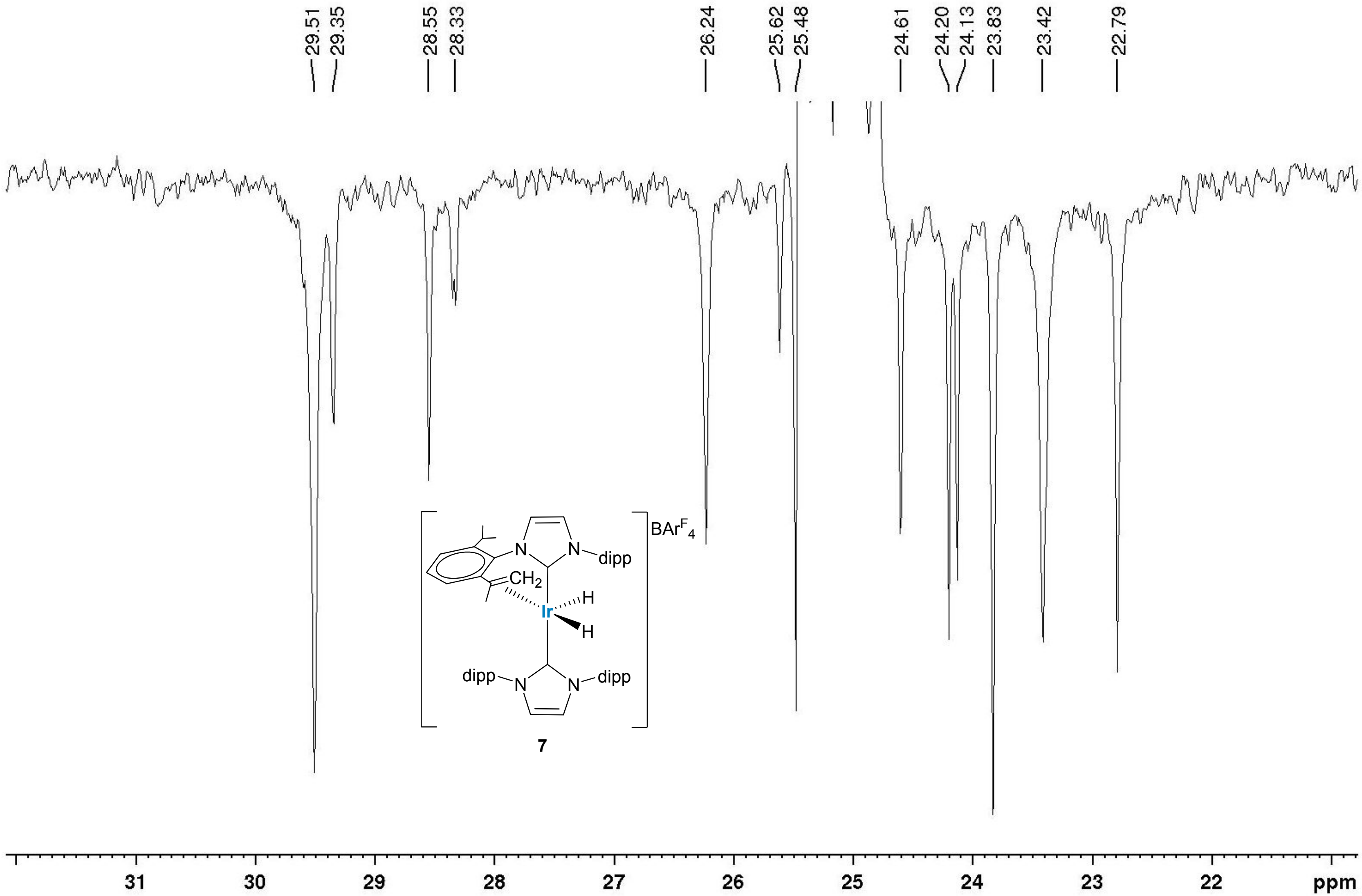

Figure S47. Alkyl region of the $^{13}C\{^{1}H\}$ DEPTQ NMR spectrum (THF-d_8, 126 MHz, 318 K) of [Ir(IPr)(IPr'')H_2][$BAr^F{}_4$] (**7**).

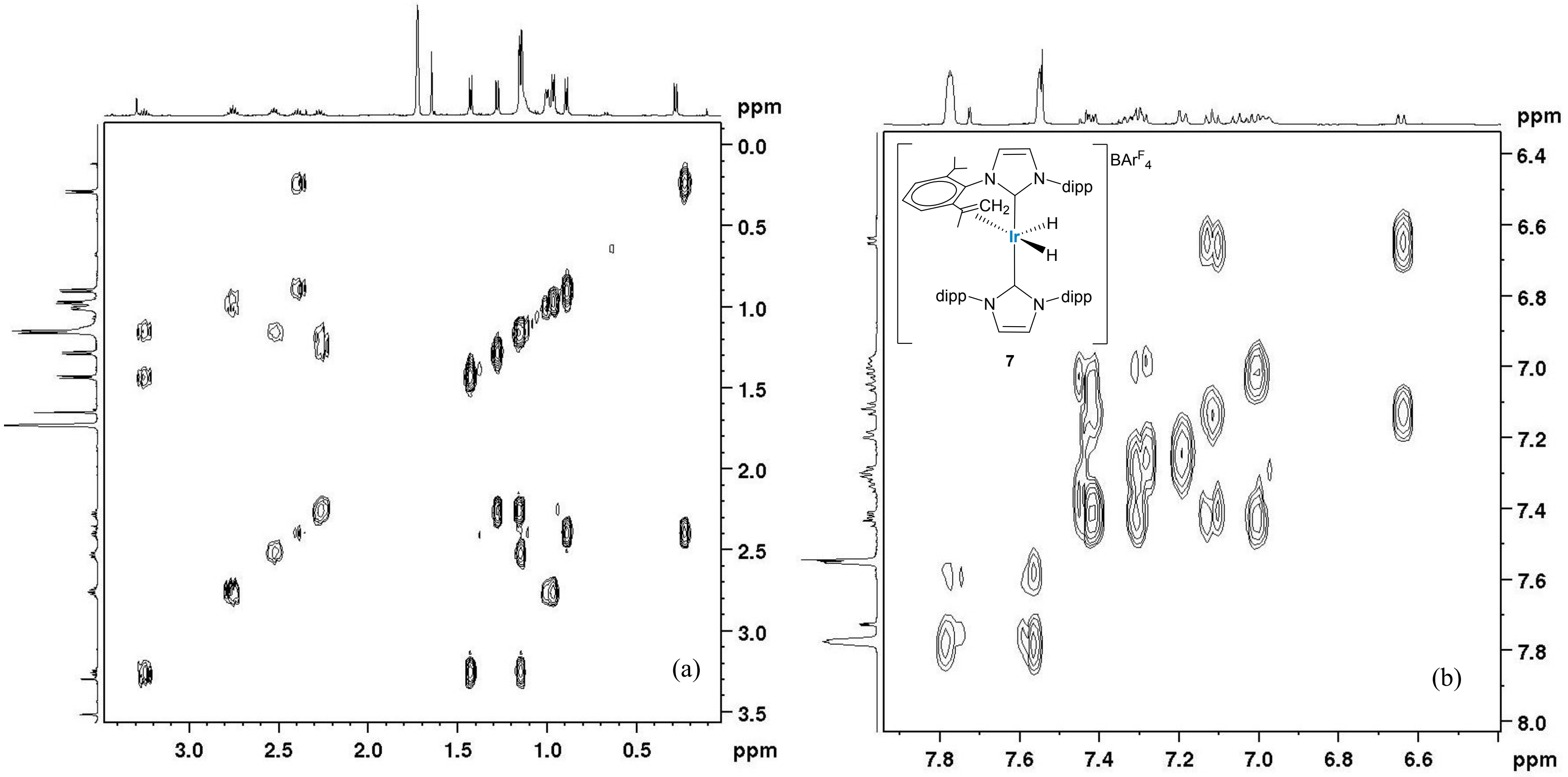

Figure S48. (a) Alkyl and (b) aryl regions of the ^{1}H COSY spectrum (THF-d_8, 500 MHz, 318 K) of $[Ir(IPr)(IPr'')H_2][BAr^F{}_4]$ (**7**).

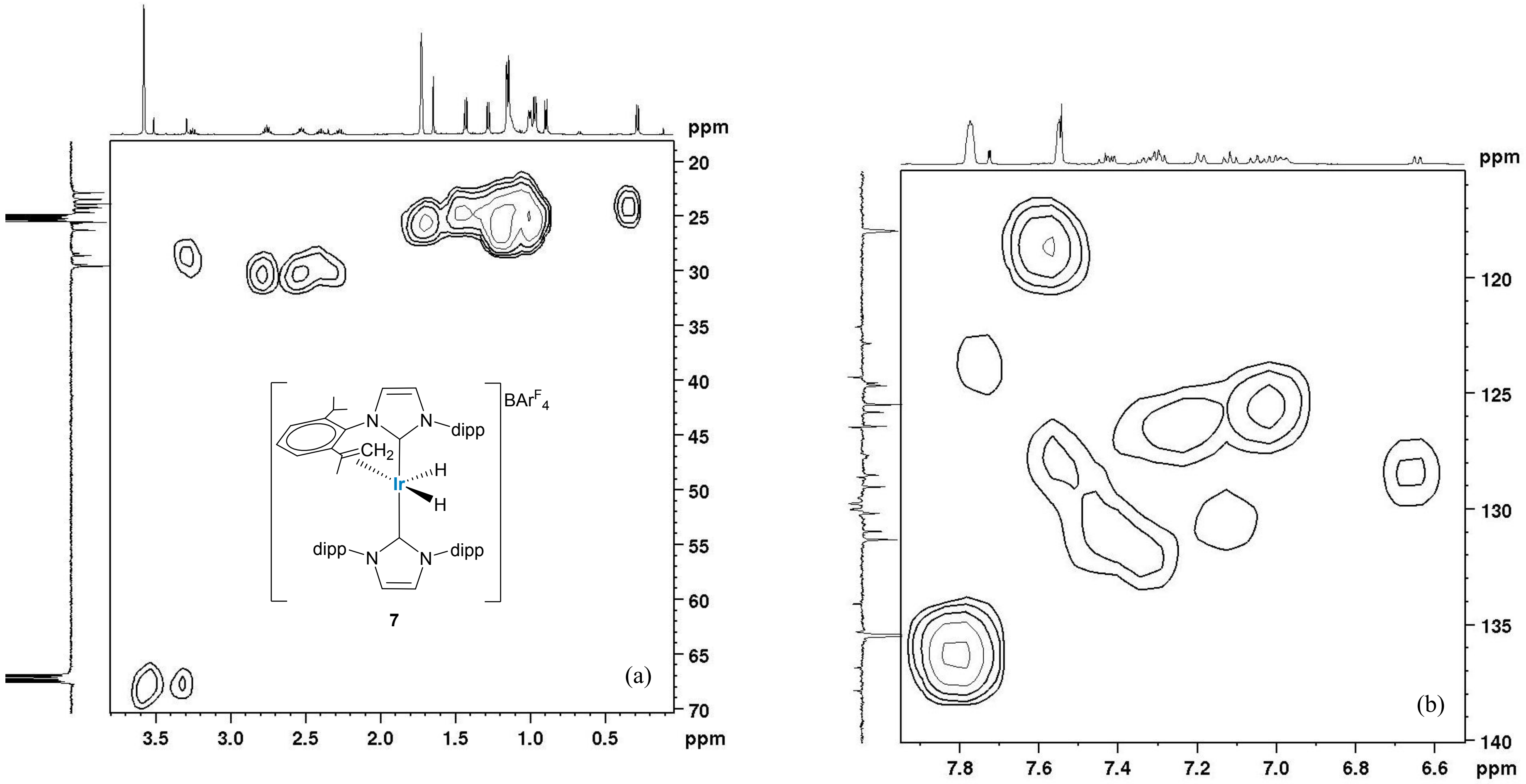

Figure S49. (a) Alkyl and (b) aryl regions of the ^{13}C-^{1}H HSQC spectrum (THF-d_8, 318 K) of $[Ir(IPr)(IPr'')H_2][BAr^F{}_4]$ (**7**).

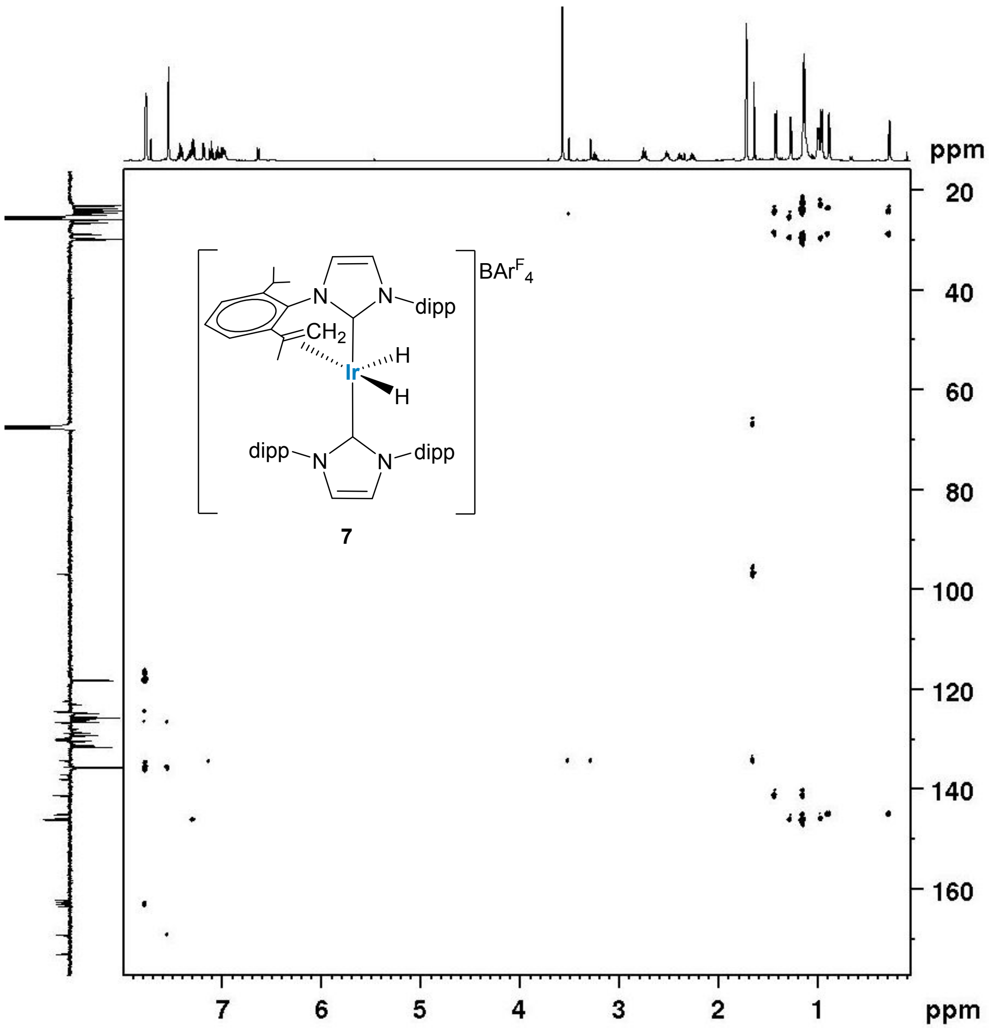

Figure S50. ^{13}C-^{1}H HMBC spectrum (THF-d_8, 318 K) of $[Ir(IPr)(IPr'')H_2][BAr^F{}_4]$ (**7**).

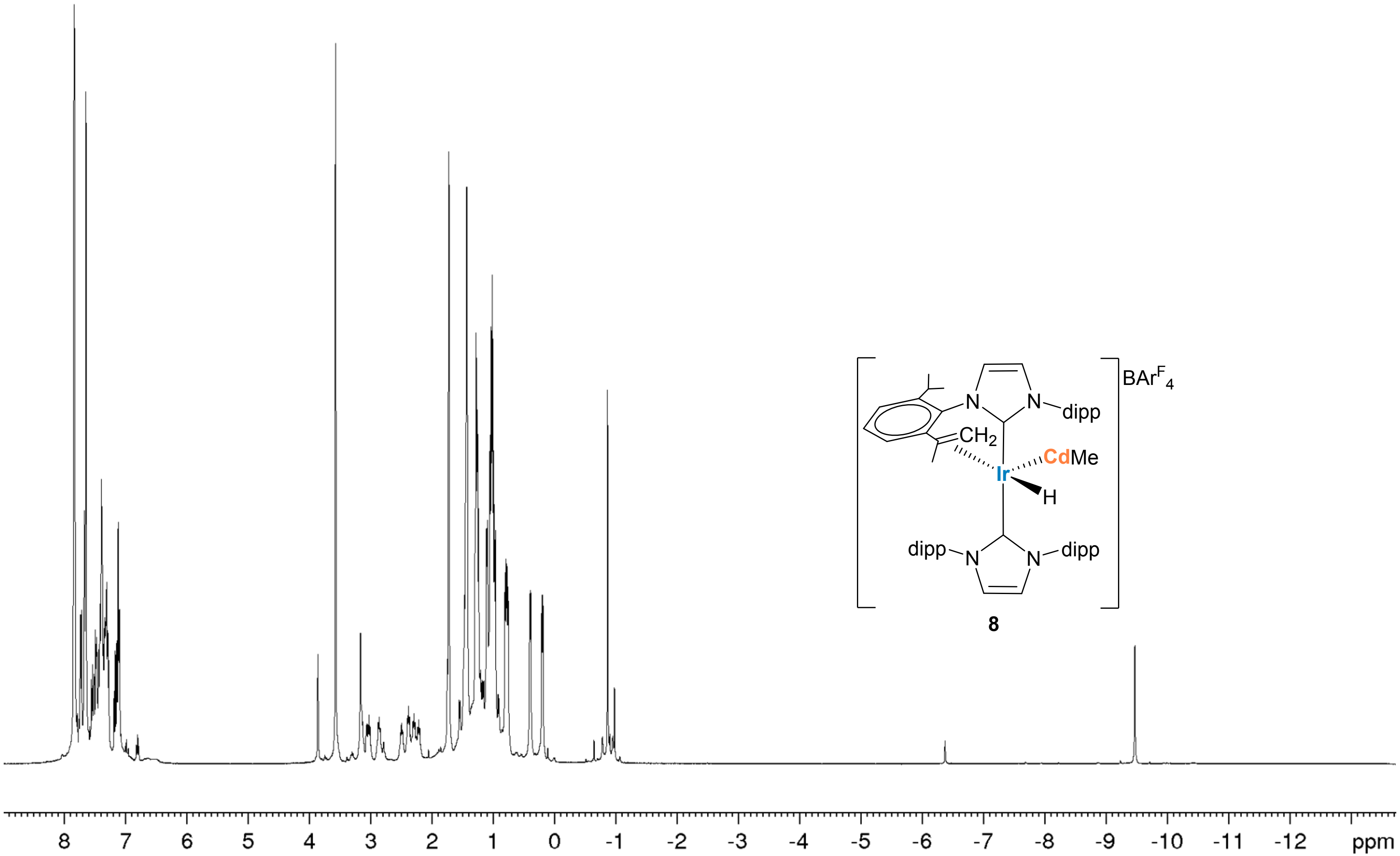

Figure S51. ^{1}H NMR spectrum (THF-d_8, 400 MHz, 248 K) of [Ir(IPr)(IPr'')(CdMe)H][BAr$^F_4$] (**8**). The minor hydride resonance at ca. δ –6.5 arises from a proposed isomer.

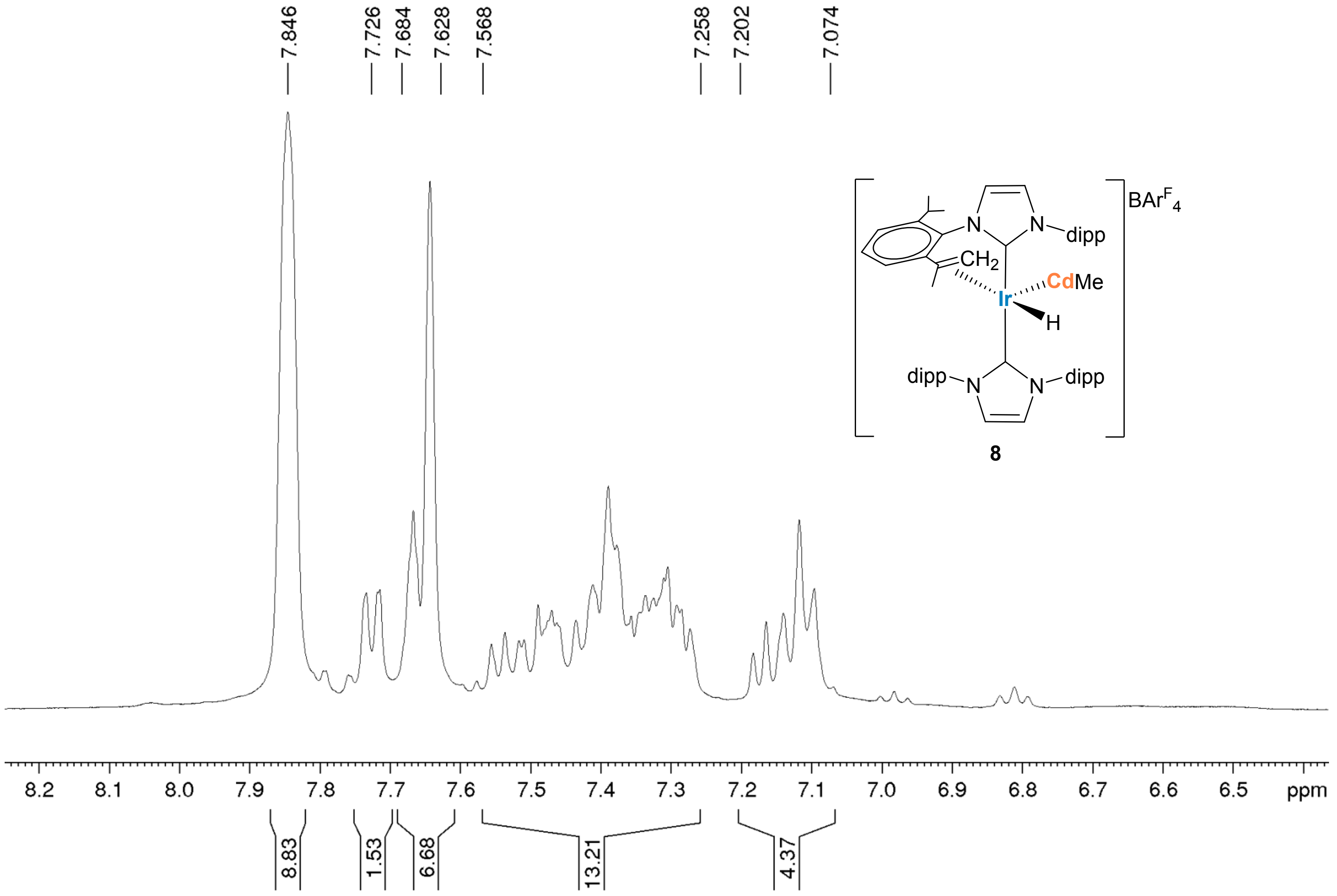

Figure S52. Aromatic region of the ^{1}H NMR spectrum (THF-d_8, 400 MHz, 248 K) of [Ir(IPr)(IPr'')(CdMe)H][BAr$^F{}_4$] (**8**). Integrals (relative to hydride resonance at δ –9.5) are slightly complicated by small amounts of the second isomer apparent in the preceding spectrum.

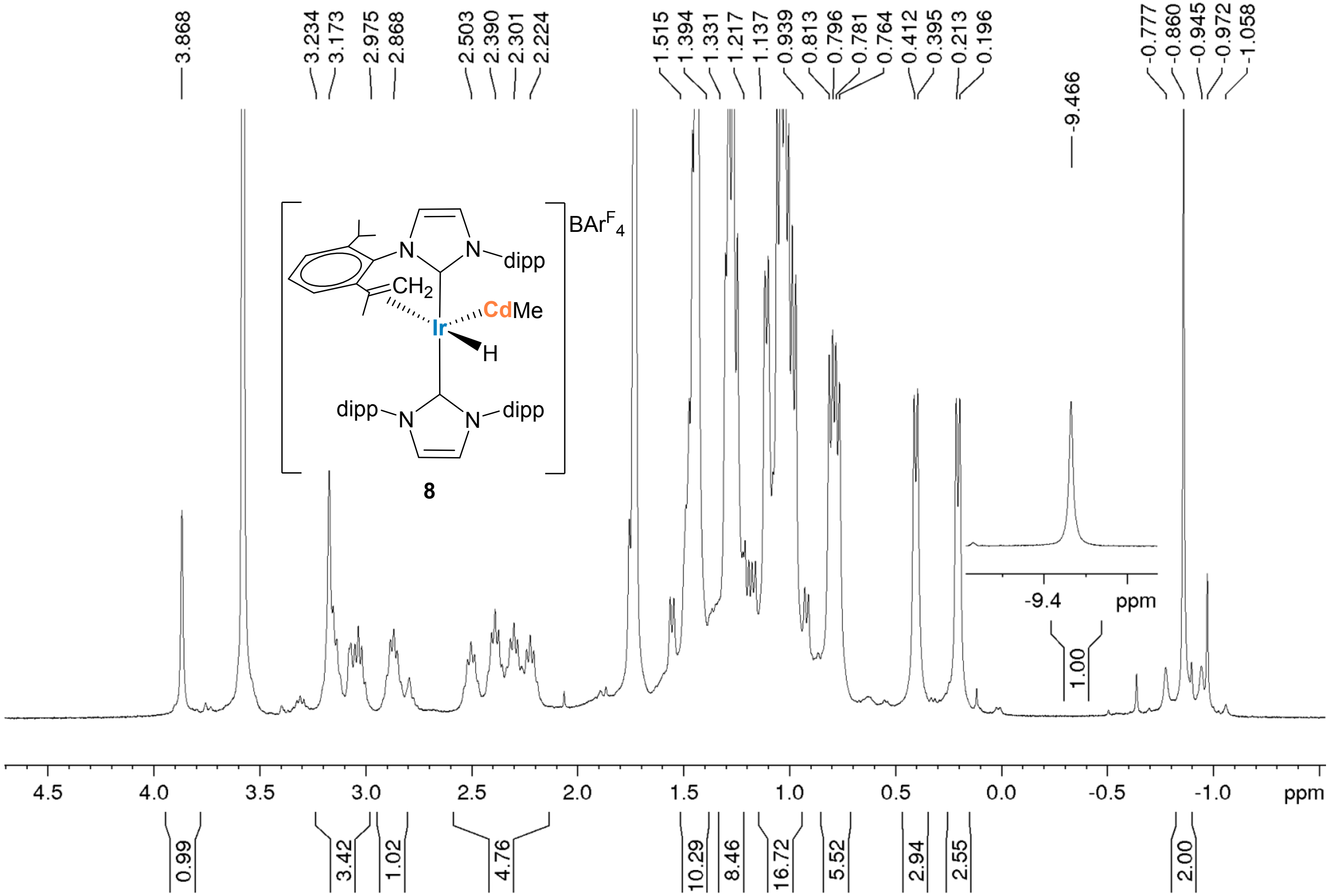

Figure S53. Alkyl region of the ^{1}H NMR spectrum (THF-d_8, 400 MHz, 248 K) of [Ir(IPr)(IPr'')(CdMe)H][BAr$^F_4$] (**8**). Integrals (relative to hydride resonance shown in inset) are slightly complicated by small amounts of the second isomer apparent in the preceding spectra. The low integral for the Cd–*Me* signal is due to only the main component of the resonance being integrated due to overlap of the Cd satellites.

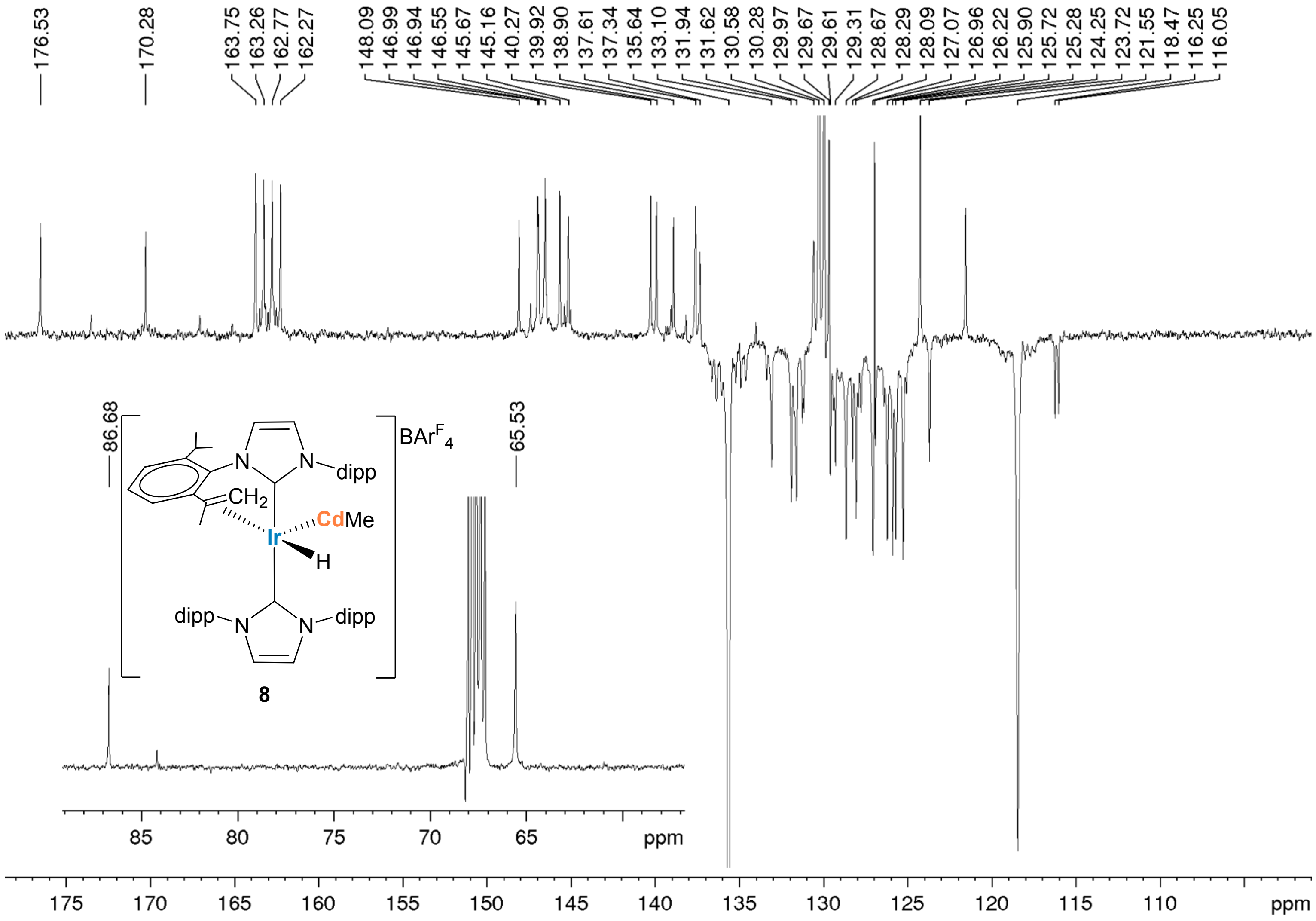

Figure S54. Higher frequency regions of the $^{13}C\{^{1}H\}$ DEPTQ NMR spectrum (THF-d_8, 101 MHz, 248 K) of [Ir(IPr)(IPr'')(CdMe)H][BAr$^{F}_{4}$] (**8**). Smaller resonances are attributed to the minor isomer (see Figure S51).

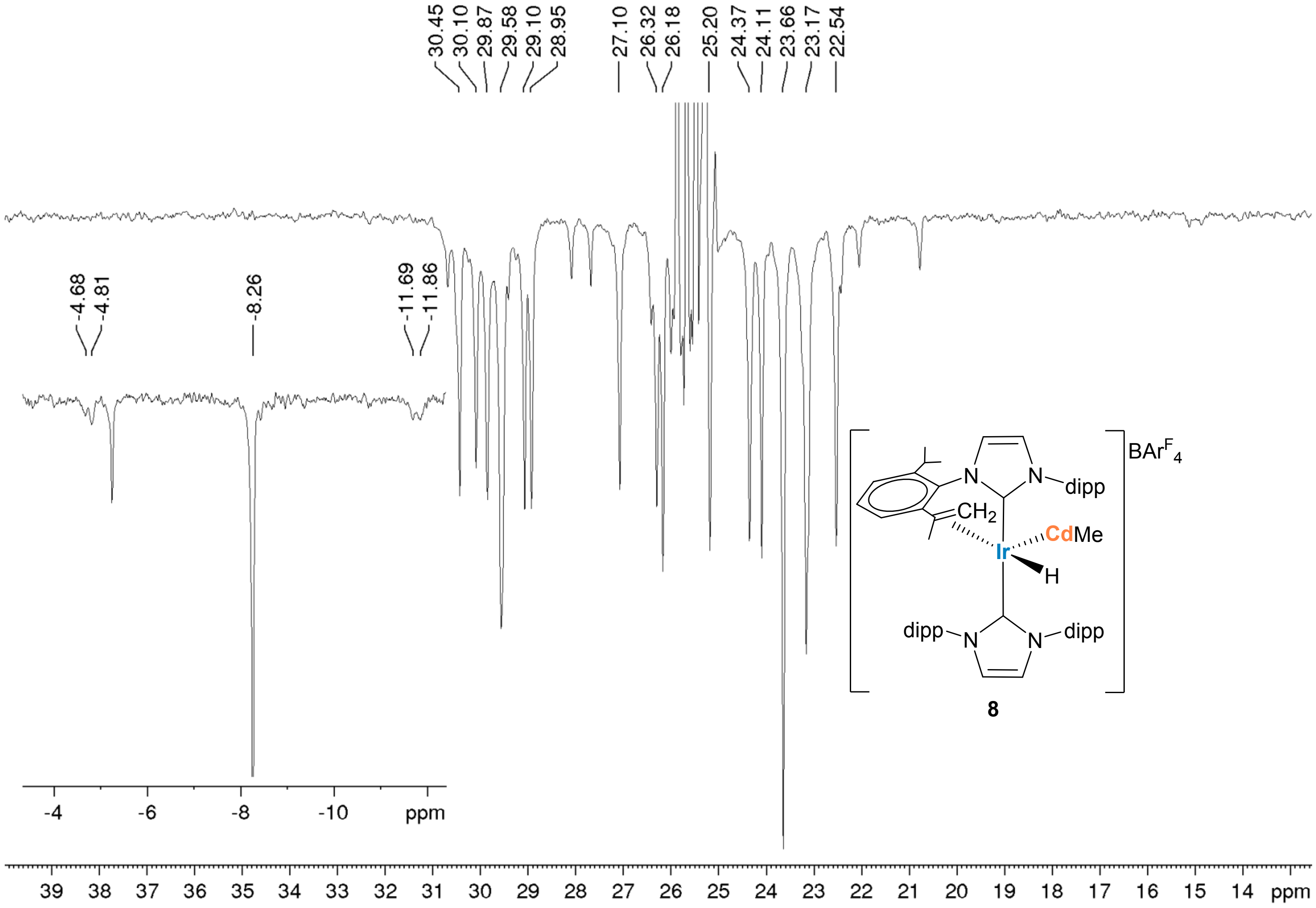

Figure S55. Lower frequency regions of the $^{13}C\{^1H\}$ DEPTQ NMR spectrum (THF-d_8, 101 MHz, 248 K) of [Ir(IPr)(IPr'')(CdMe)H][BAr$^F{}_4$] (**8**). Smaller resonances (e.g. Cd–*Me* resonance at ca. δ –5) are attributed to the minor isomer.

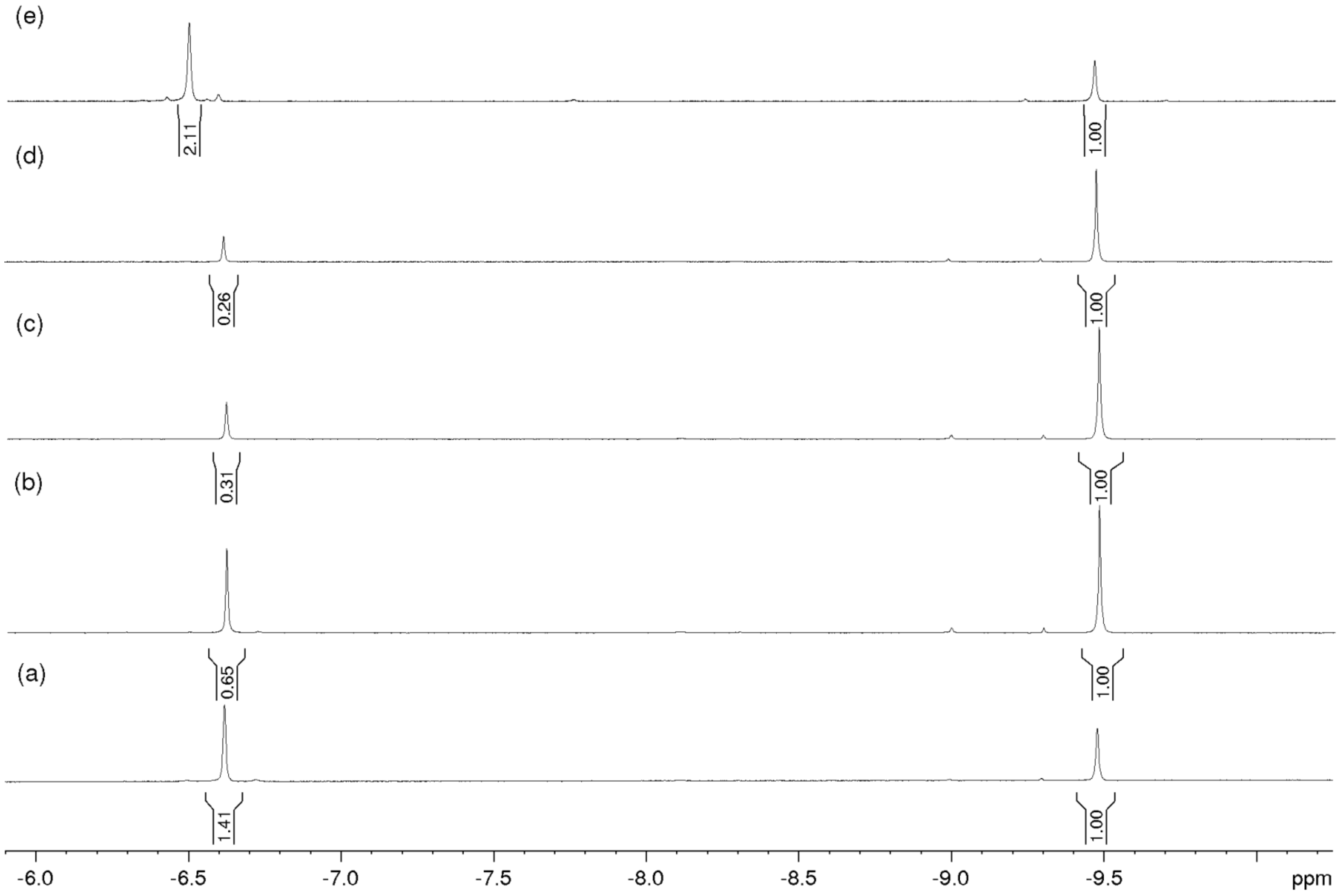

Figure S56. Hydride region of the ^{1}H NMR spectrum (C_6H_5F, 500 MHz, 298 K) of the reaction of [Ir(IPr)(IPr'')H_2][$BAr^F{}_4$] (**7**) and 1 equiv $CdMe_2$. Spectra recorded (a) 5 min, (b) 1 h, (c) 3 h and (d) 4 h after mixing. Dominance of the proposed isomer at ca. δ –6.5 is clear at early times, followed by conversion through to **8** at ca. δ –9.5. The spectrum in (e) was recorded at 248 K (C_6H_5F, 400 MHz) after condensing $CdMe_2$ into a solution of **7**; formation of **8** is clear even under these low temperature conditions.

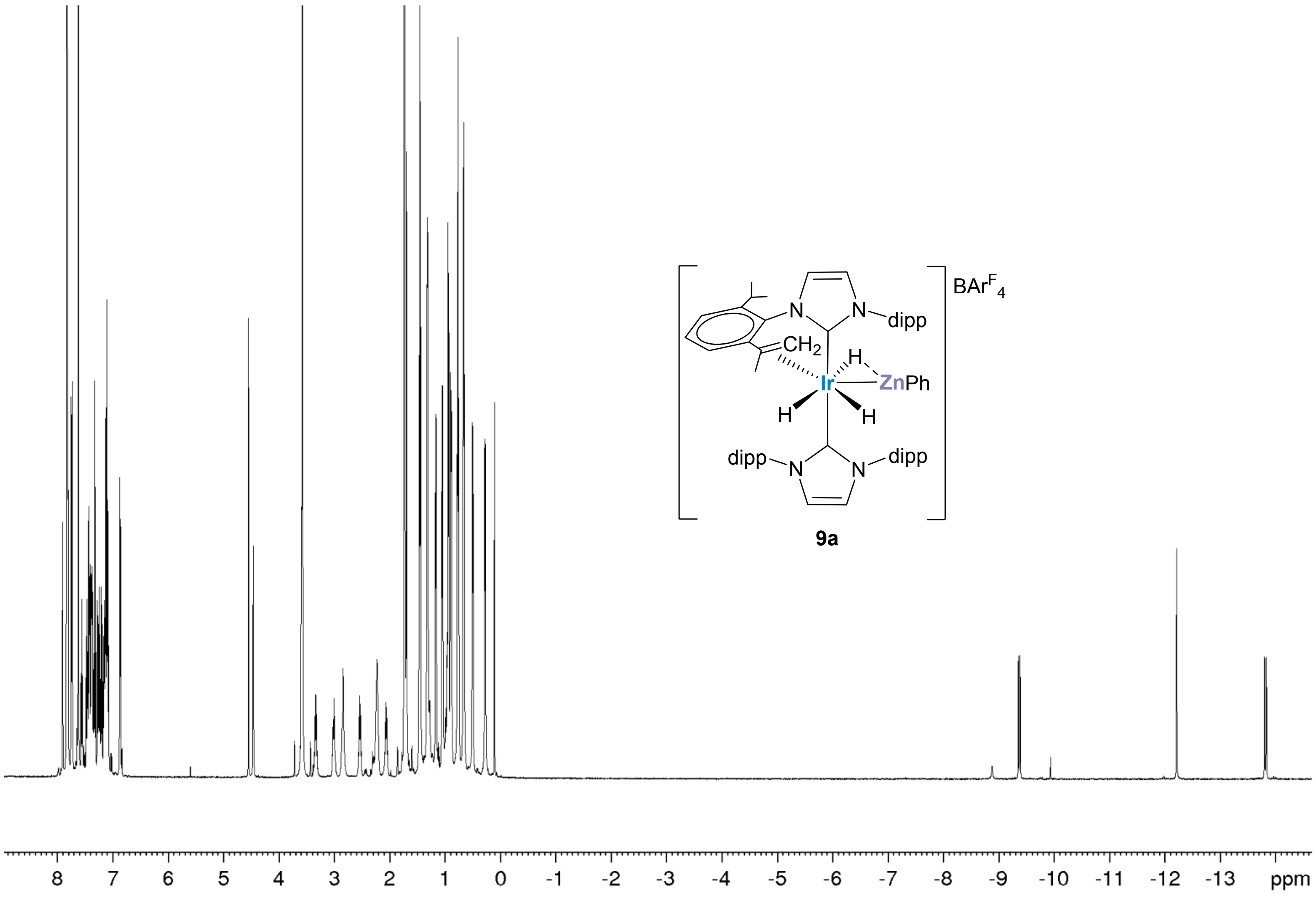

Figure S57. ^{1}H NMR spectrum (THF-d_8, 500 MHz, 248 K) of [Ir(IPr)(IPr'')(ZnPh)H_3][BAr^F_4] (**9a**) generated by low temperature addition of H_2 to [Ir(IPr)(IPr'')(ZnPh)H][BAr^F_4] (**4a**).

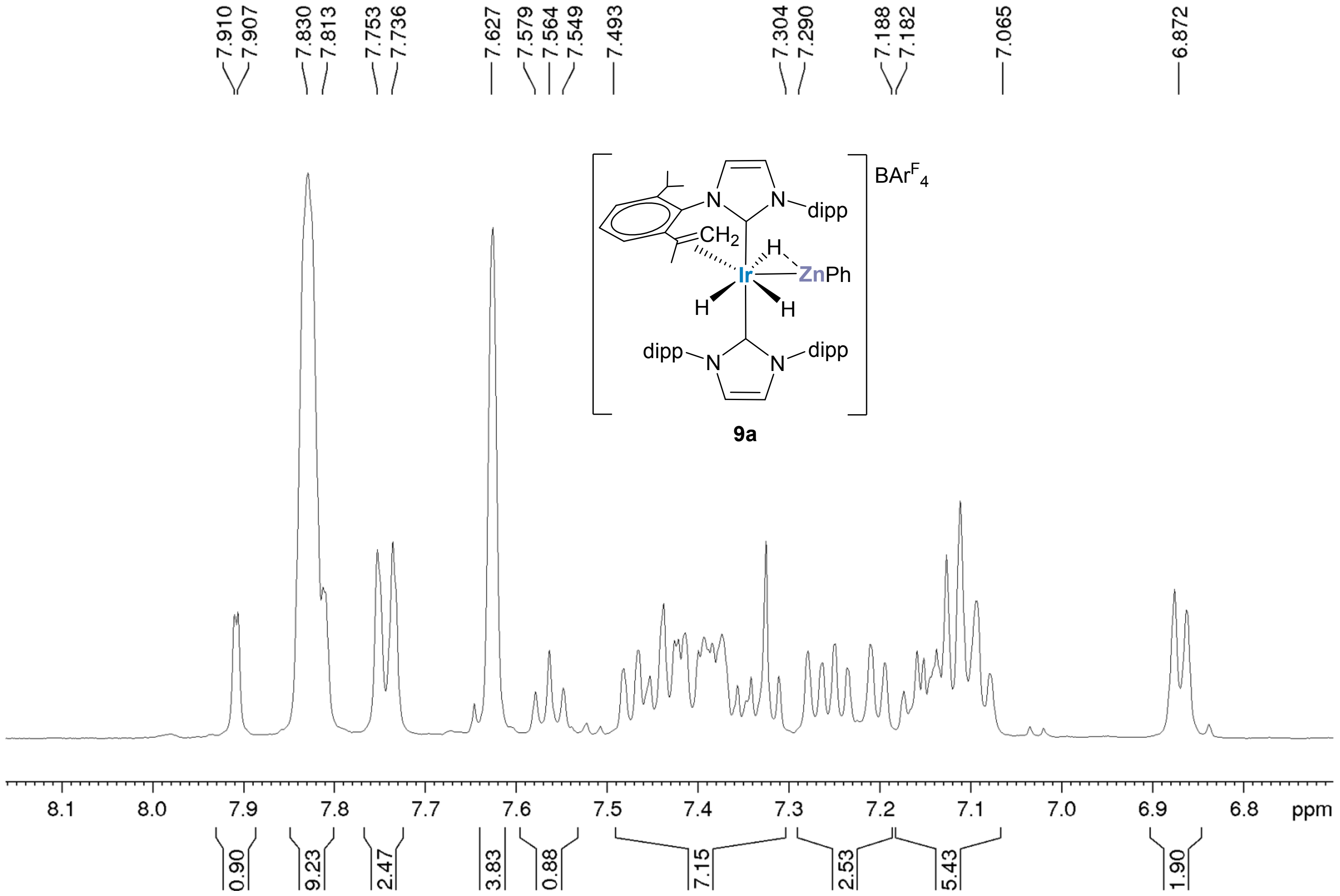

Figure S58. ^{1}H NMR aromatic resonances (THF-d_8, 500 MHz, 248 K) of [Ir(IPr)(IPr'')(ZnPh)H_3][BAr$^F{}_4$] (**9a**) generated by low temperature addition of H_2 to [Ir(IPr)(IPr'')(ZnPh)H][BAr$^F{}_4$] (**4a**).

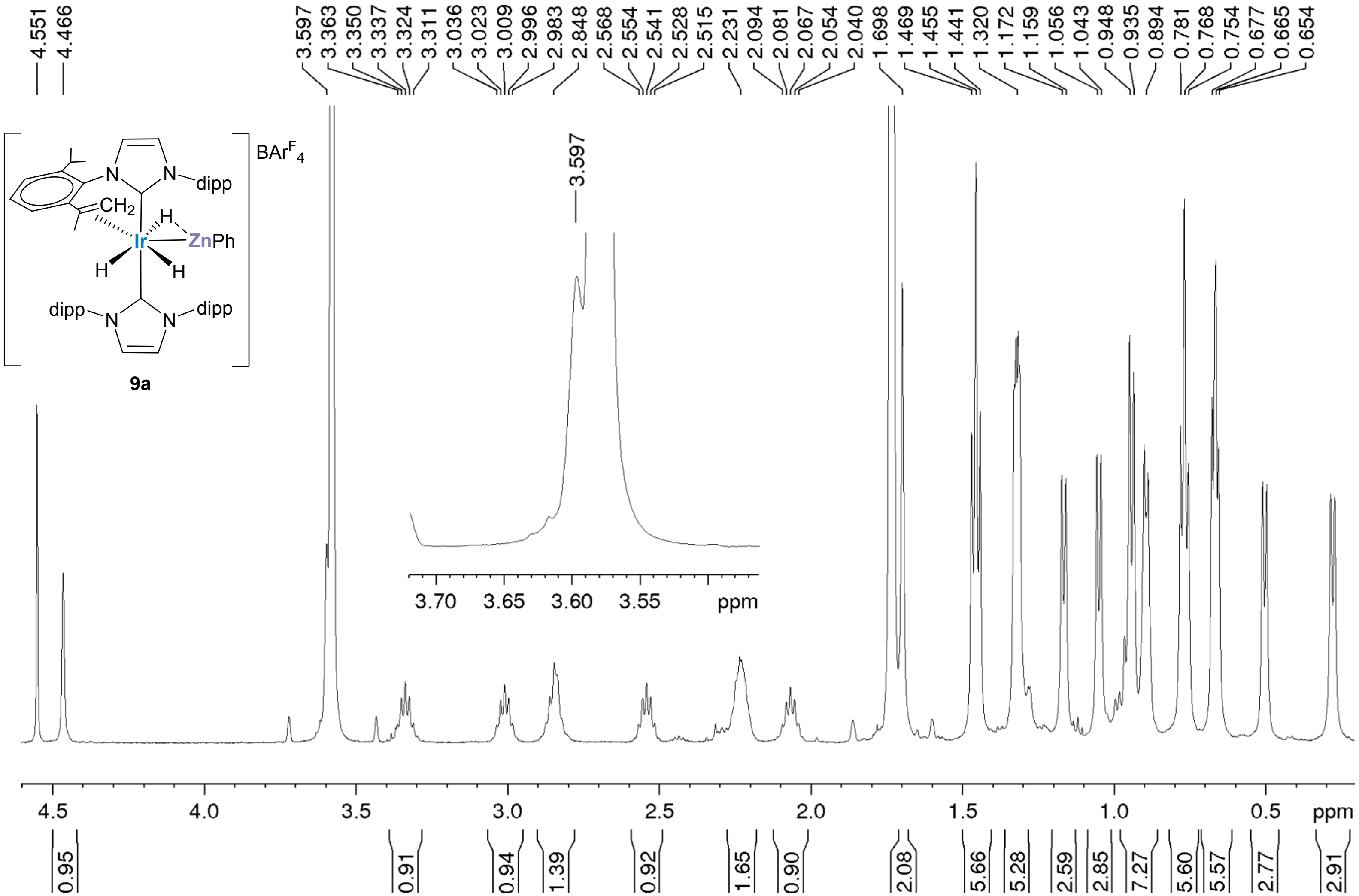

Figure S59. ^{1}H NMR alkyl resonances (THF-d_8, 500 MHz, 248 K) of [Ir(IPr)(IPr'')(ZnPh)H_3][BAr$^F{}_4$] (**9a**) generated by low temperature addition of H_2 to [Ir(IPr)(IPr'')(ZnPh)H][BAr$^F{}_4$] (**4a**). Inset shows what is assigned as the partially obscured –C(Me)=CH*H* signal.

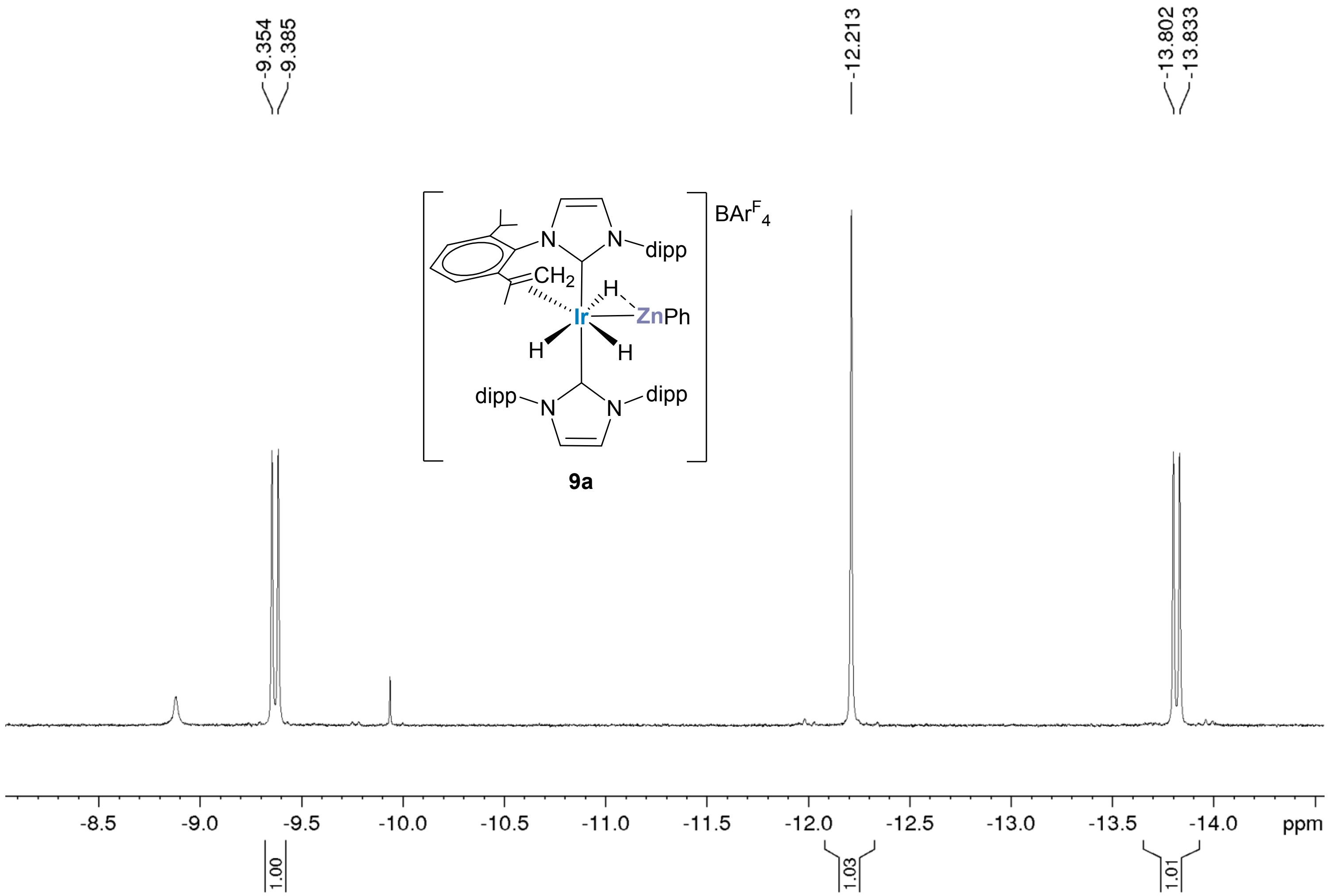

Figure S60. ^{1}H NMR hydride resonances (THF-d_8, 500 MHz, 248 K) of [Ir(IPr)(IPr'')(ZnPh)H_3][BAr$^F{}_4$] (**9a**) generated by low temperature addition of H_2 to [Ir(IPr)(IPr'')(ZnPh)H][BAr$^F{}_4$] (**4a**). Minor resonances at ca. δ −9 and −10 are assigned to [Ir(IPr)$_2$(ZnPh)(η^2-H_2)H_3][BAr$^F{}_4$] (**11a**; by comparison to **11b** and **11c**) and Ir(IPr)$_2H_5$ (**13**) respectively.

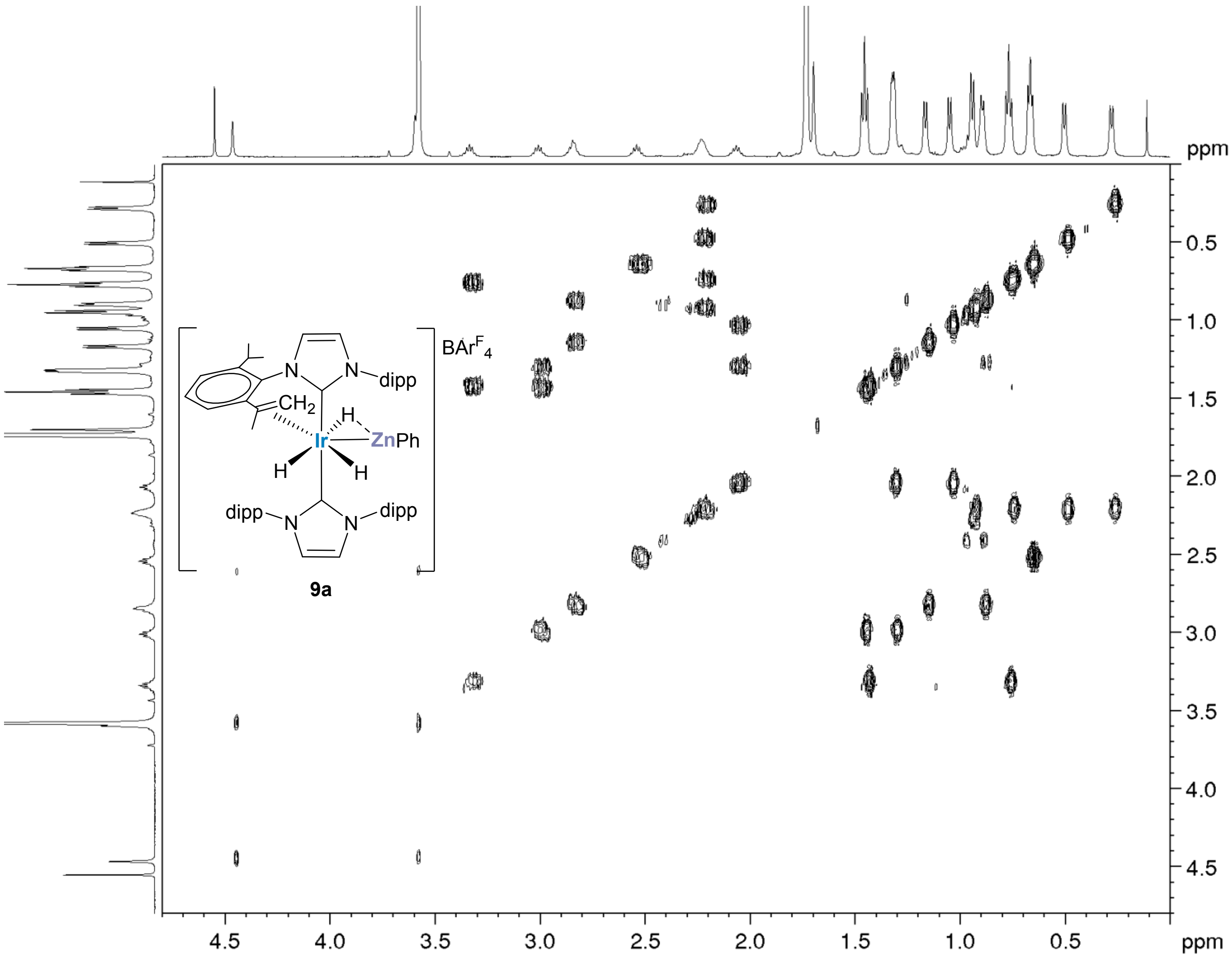

Figure S61. Alkyl region of the ^{1}H COSY NMR spectrum (THF-d_8, 500 MHz, 248 K) of [Ir(IPr)(IPr'')(ZnPh)H_3][BAr$^F{}_4$] (**9a**) generated by low temperature addition of H_2 to [Ir(IPr)(IPr'')(ZnPh)H][BAr$^F{}_4$] (**4a**).

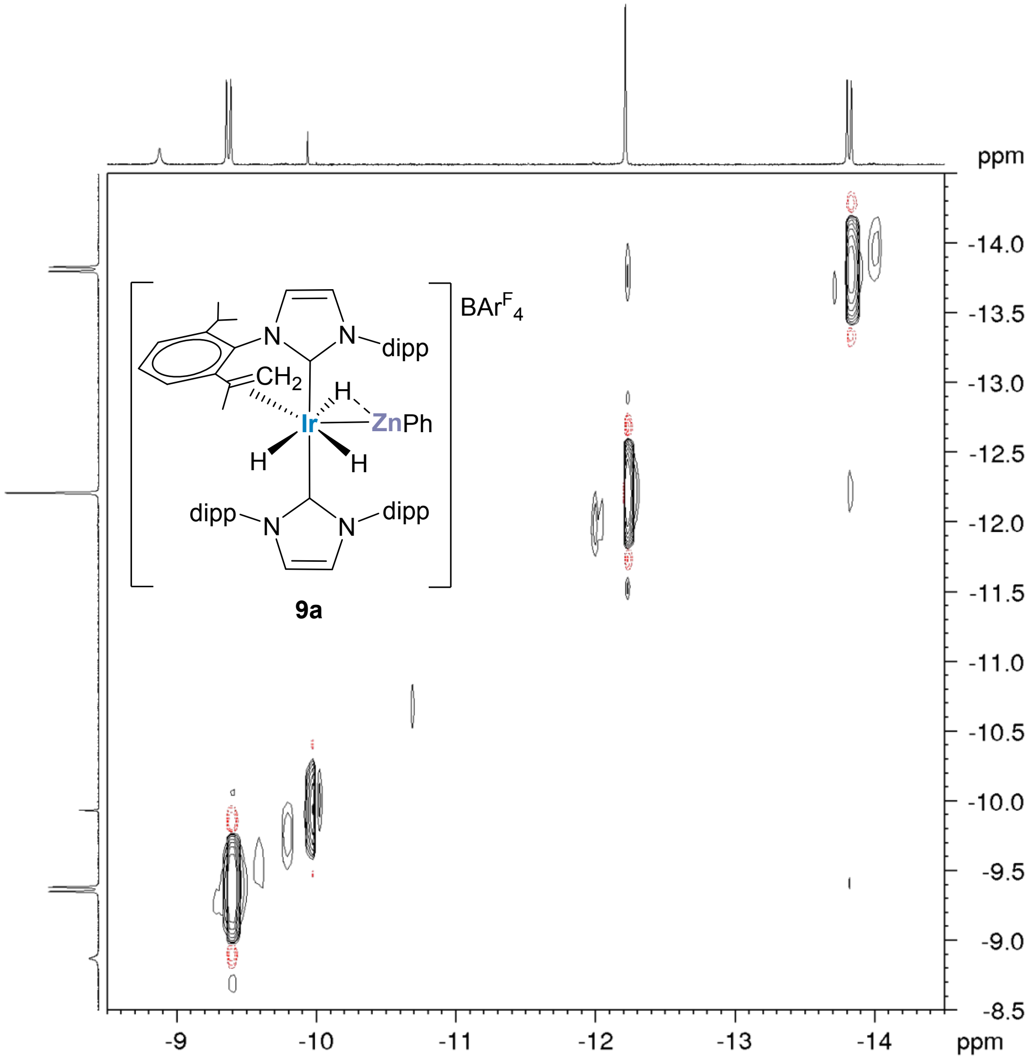

Figure S62. Hydride region of the ^{1}H NOESY NMR spectrum (THF-d_8, 500 MHz, 248 K) of [Ir(IPr)(IPr'')(ZnPh)H_3][BAr$^F{}_4$] (**9a**) generated by low temperature addition of H_2 to [Ir(IPr)(IPr'')(ZnPh)H][BAr$^F{}_4$] (**4a**).

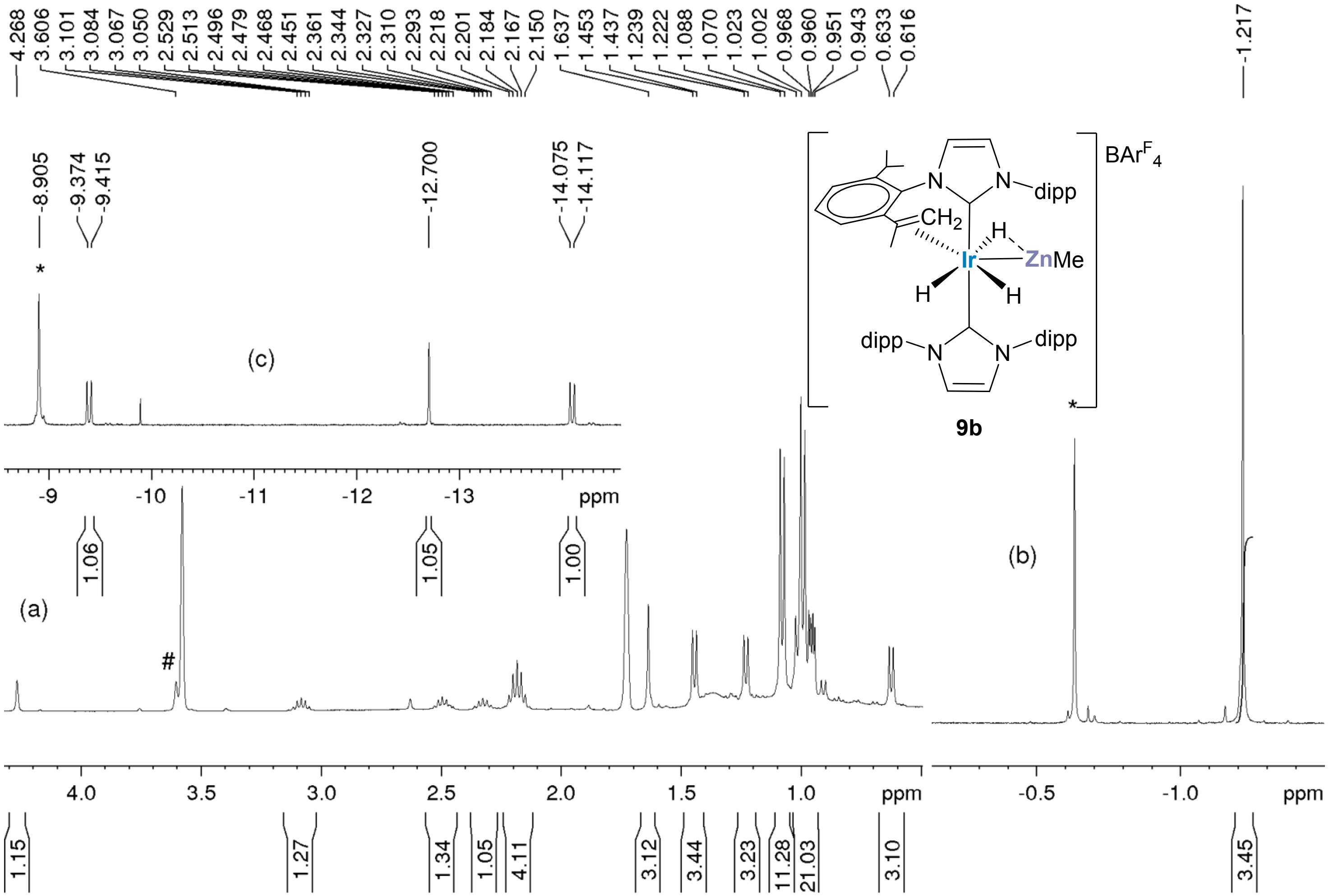

Figure S63. (a) Alkyl, (b) ZnMe and (c) hydride regions of a ^{1}H NMR spectrum (THF-d_8, 400 MHz, 298 K) containing [Ir(IPr)(IPr'')(ZnMe)H_3][BAr$^F_4$] (**9b**) recorded 5 min after room temperature addition of 1 atm H_2 to [Ir(IPr)(IPr'')(ZnMe)H][BAr$^F_4$] (**4b**) (# = –C(Me)=CH*H* signal (δ 3.6), * = [Ir(IPr)$_2$(ZnMe)(η^2-H_2)H_3][BAr$^F_4$] (**11b**)).

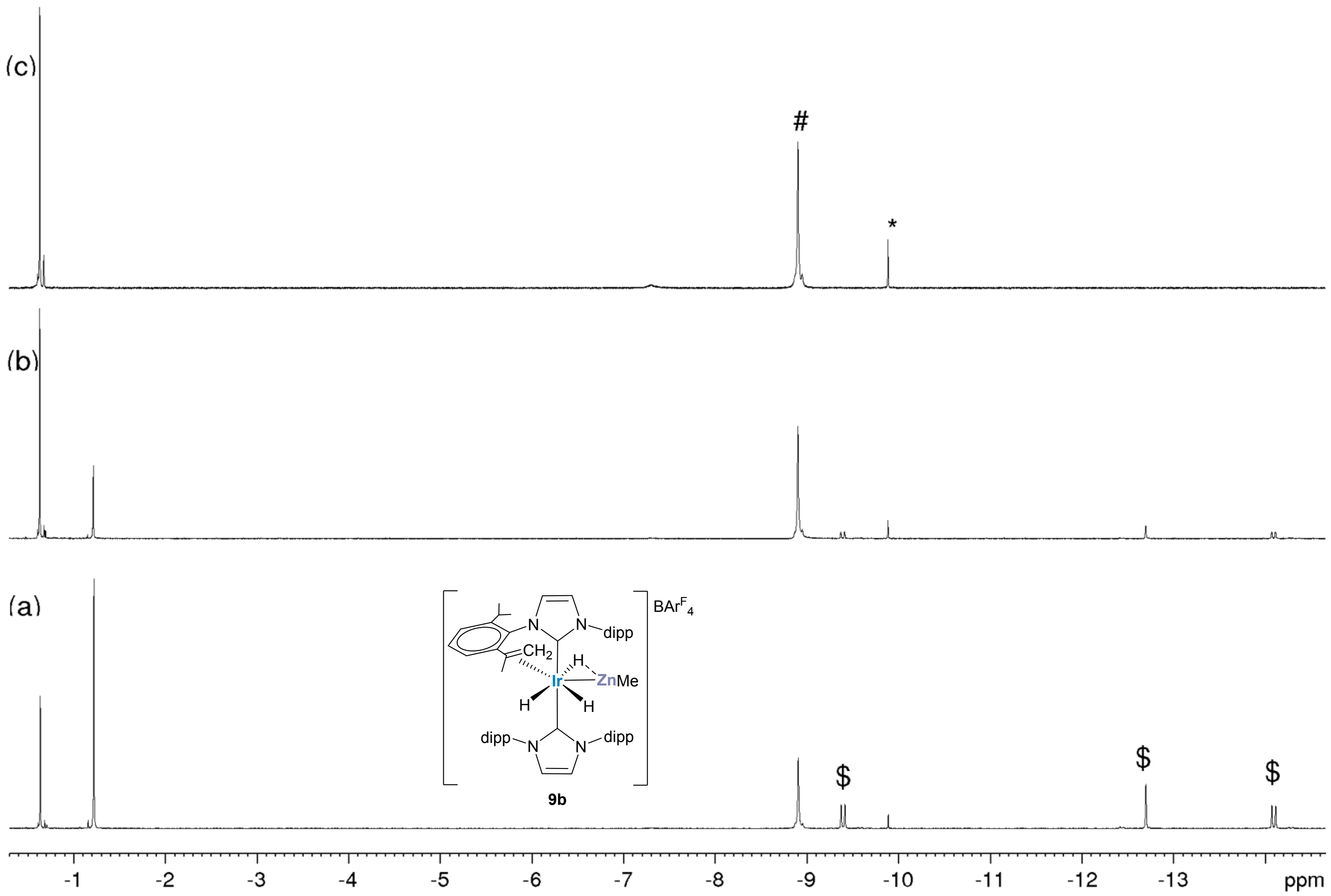

Figure S64. Low frequency region of the ^{1}H NMR spectrum (THF-d_8, 400 MHz, 298 K) recorded (a) 20 min, (b) 1 h and (c) 12 h after room temperature addition of H_2 to [Ir(IPr)(IPr'')(ZnMe)H][BAr$^F_4$] (**4b**), showing (a) initially [Ir(IPr)(IPr'')(ZnMe)H$_3$][BAr$^F_4$] (**9b**; $) initially, which then reacts onwards in (b) and (c) to form [Ir(IPr)$_2$(ZnMe)(η^2-H$_2$)H$_3$][BAr$^F_4$] (**11b**; #). Note the presence of Ir(IPr)$_2$H$_5$ (**13**;*).

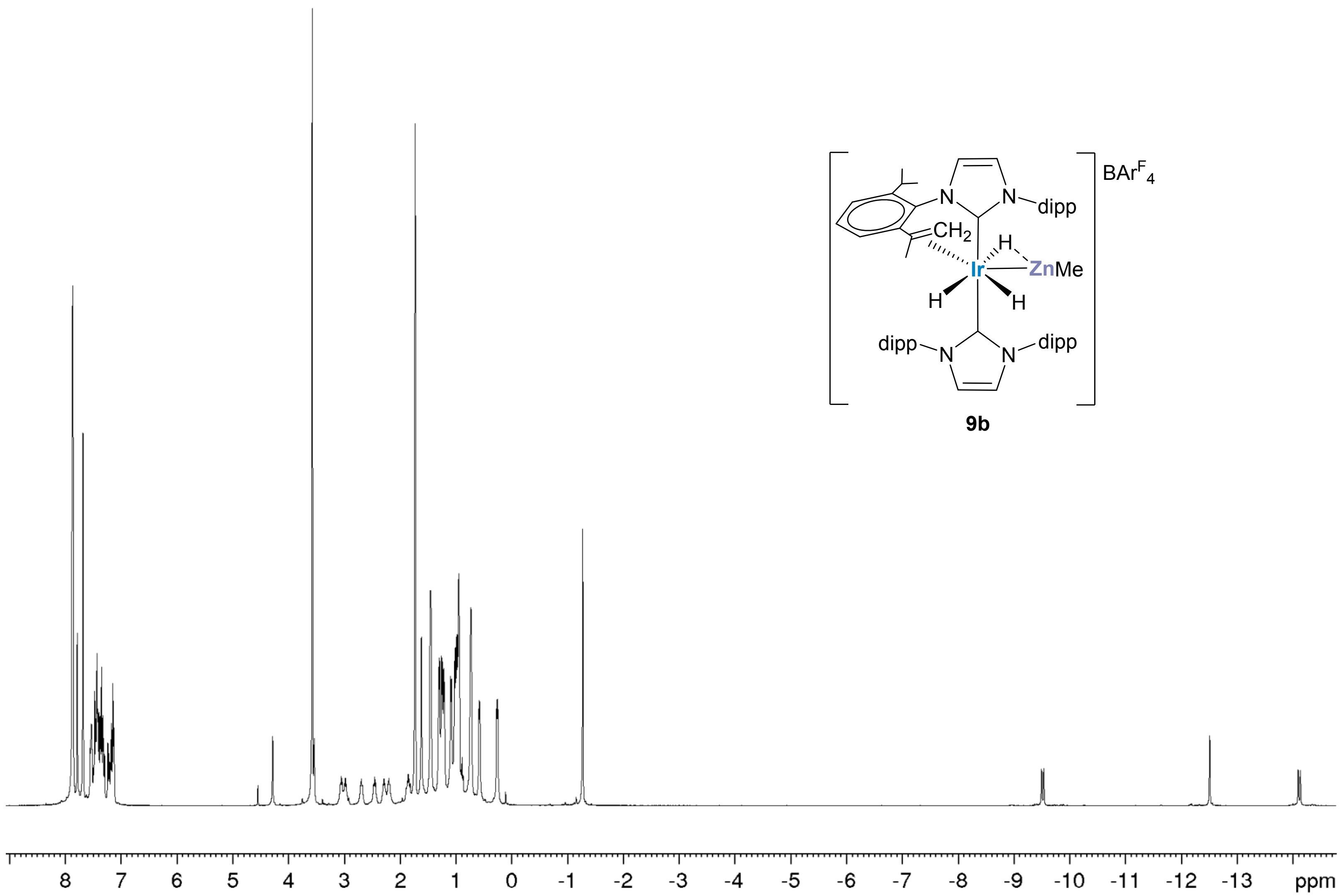

Figure S65. ^{1}H NMR spectrum (THF-d_8, 400 MHz, 228 K) of [Ir(IPr)(IPr'')(ZnMe)H_3][BAr$^F{}_4$] (**9b**) generated by low temperature addition of H_2 to [Ir(IPr)(IPr'')(ZnMe)H][BAr$^F{}_4$] (**4b**).

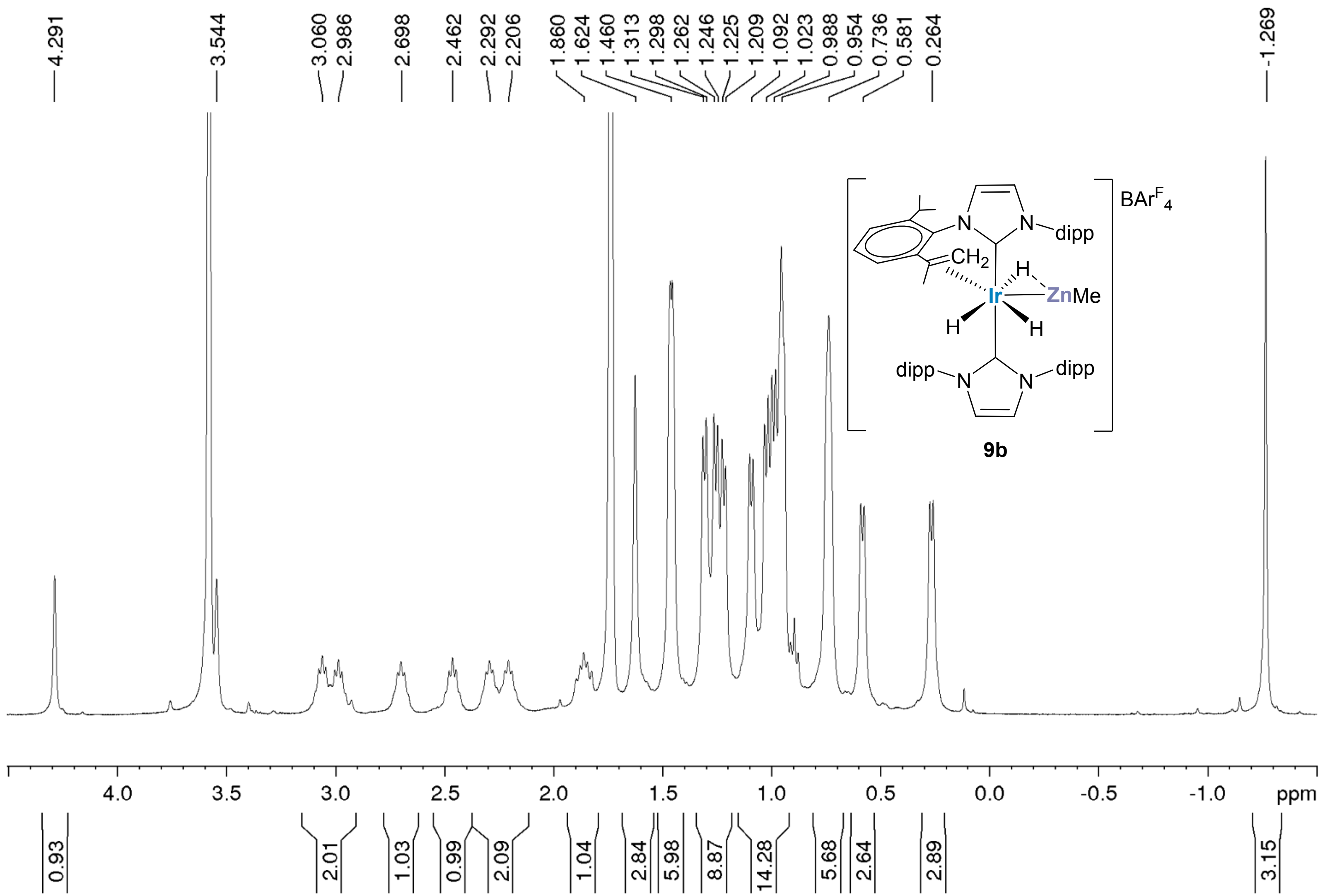

Figure S66. ^{1}H NMR alkyl resonances (THF-d_8, 400 MHz, 228 K) of [Ir(IPr)(IPr'')(ZnMe)H_3][BAr$^F{}_4$] (**9b**) generated by low temperature addition of H_2 to [Ir(IPr)(IPr'')(ZnMe)H][BAr$^F{}_4$] (**4b**).

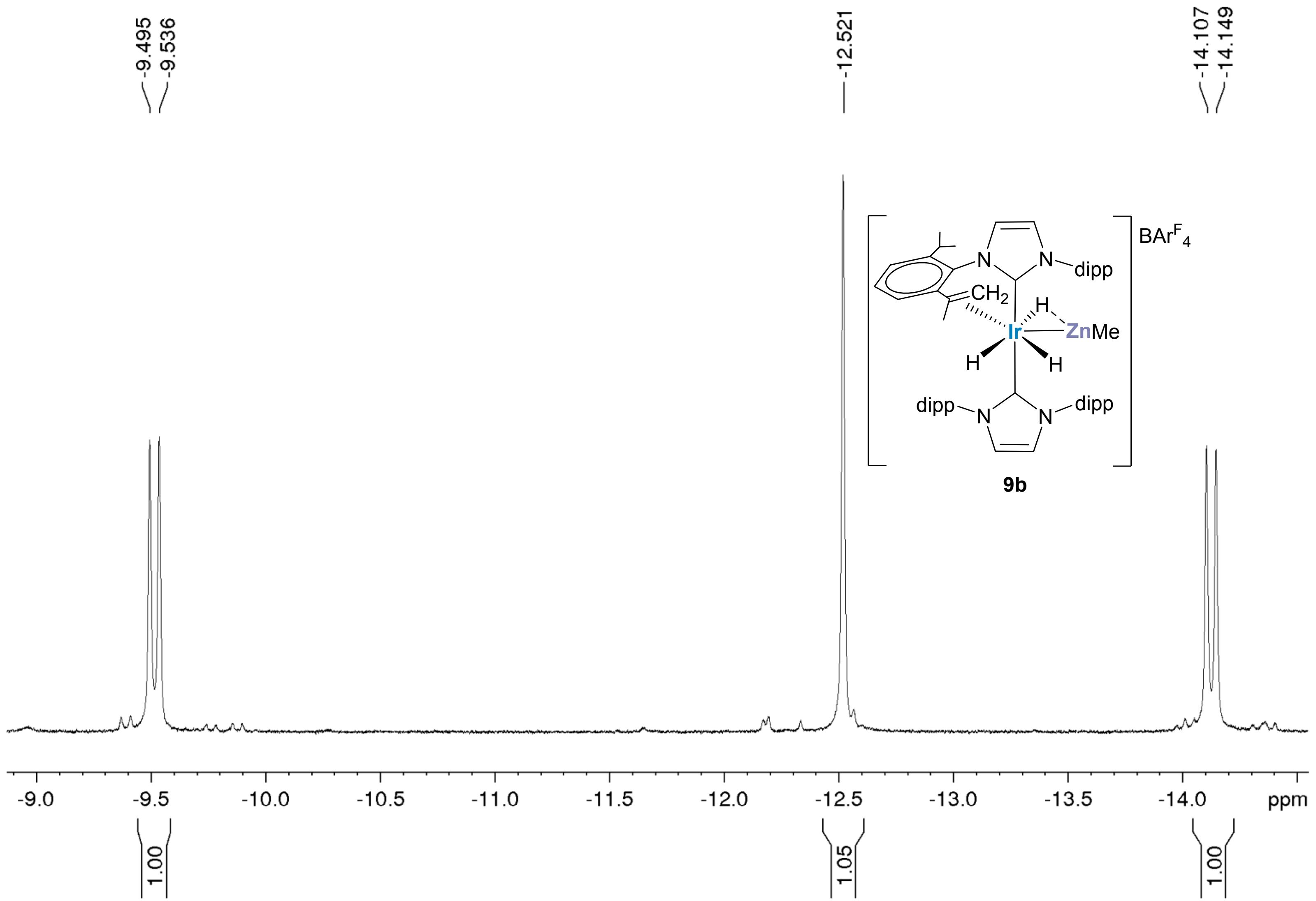

Figure S67. ^1H NMR hydride resonances (THF-d_8, 400 MHz, 228 K) of $[Ir(IPr)(IPr'')(ZnMe)H_3][BAr^F_4]$ (**9b**) generated by low temperature addition of H_2 to $[Ir(IPr)(IPr'')(ZnMe)H][BAr^F_4]$ (**4b**).

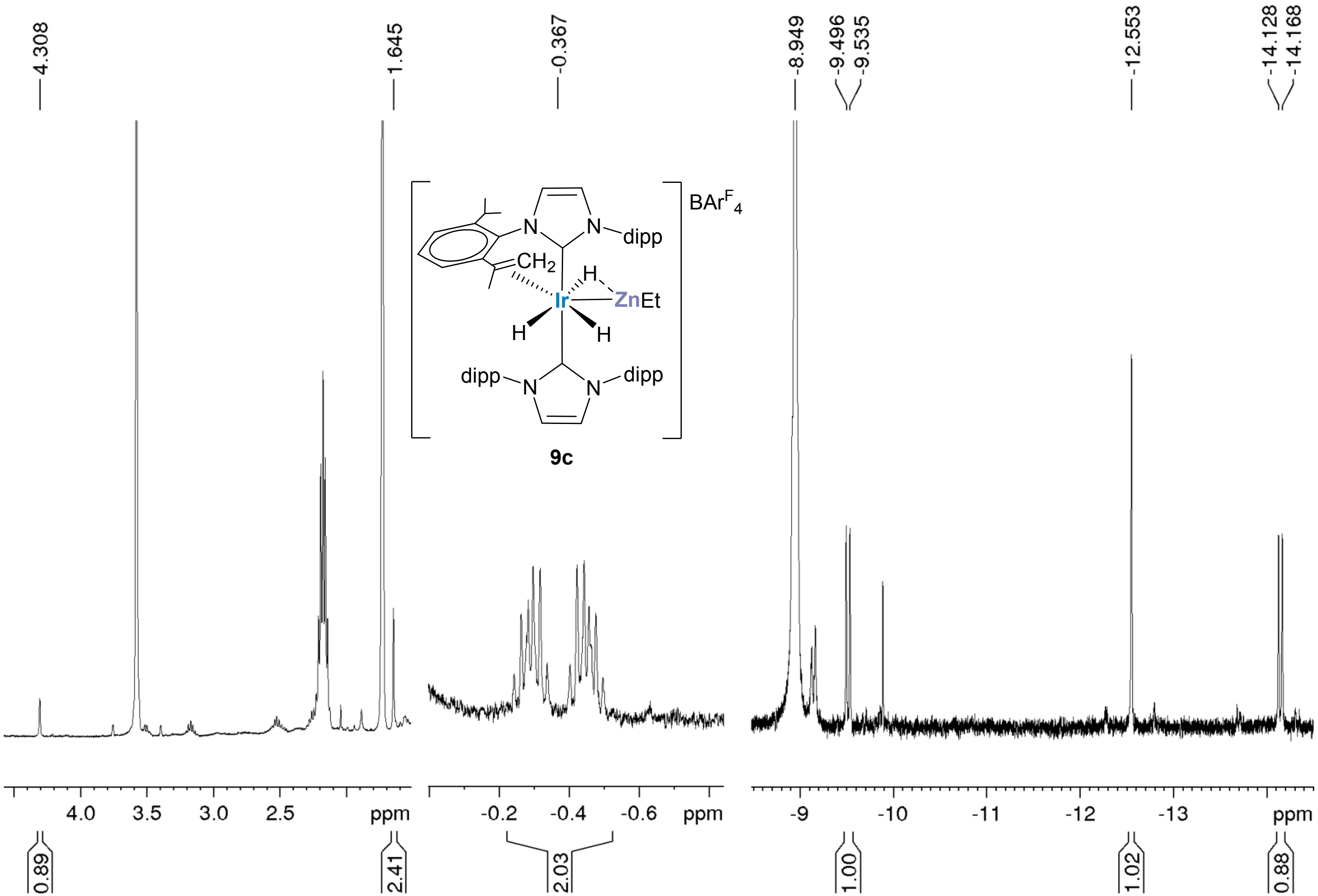

Figure S68. Diagnostic ^{1}H NMR resonances (THF-d_8, 400 MHz, 298 K) of [Ir(IPr)(IPr'')(ZnEt)H_3][BAr$^F_4$] (**9c**) formed upon room temperature addition of H_2 to [Ir(IPr)(IPr'')(ZnEt)H][BAr$^F_4$] (**4c**). The hydride resonance at ca. δ −9 arises from [Ir(IPr)$_2$(ZnEt)(η^2-H_2)H_3][BAr$^F_4$] (**11c**).

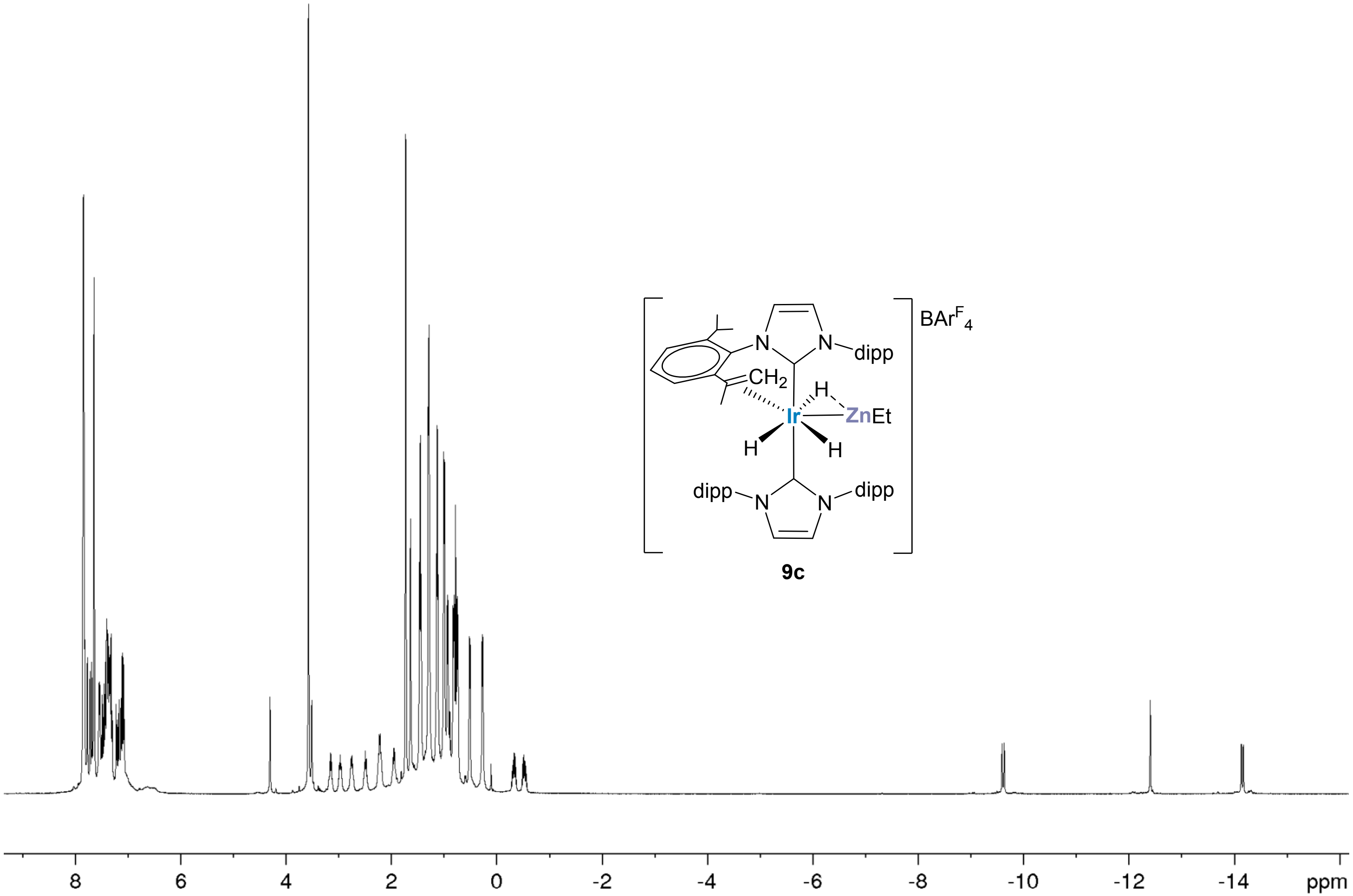

Figure S69. ¹H NMR spectrum (THF-d_8, 400 MHz, 248 K) of [Ir(IPr)(IPr'')(ZnEt)H_3][BAr^F_4] (**9c**) generated by low temperature addition of H_2 to [Ir(IPr)(IPr'')(ZnEt)H][BAr^F_4] (**4c**).

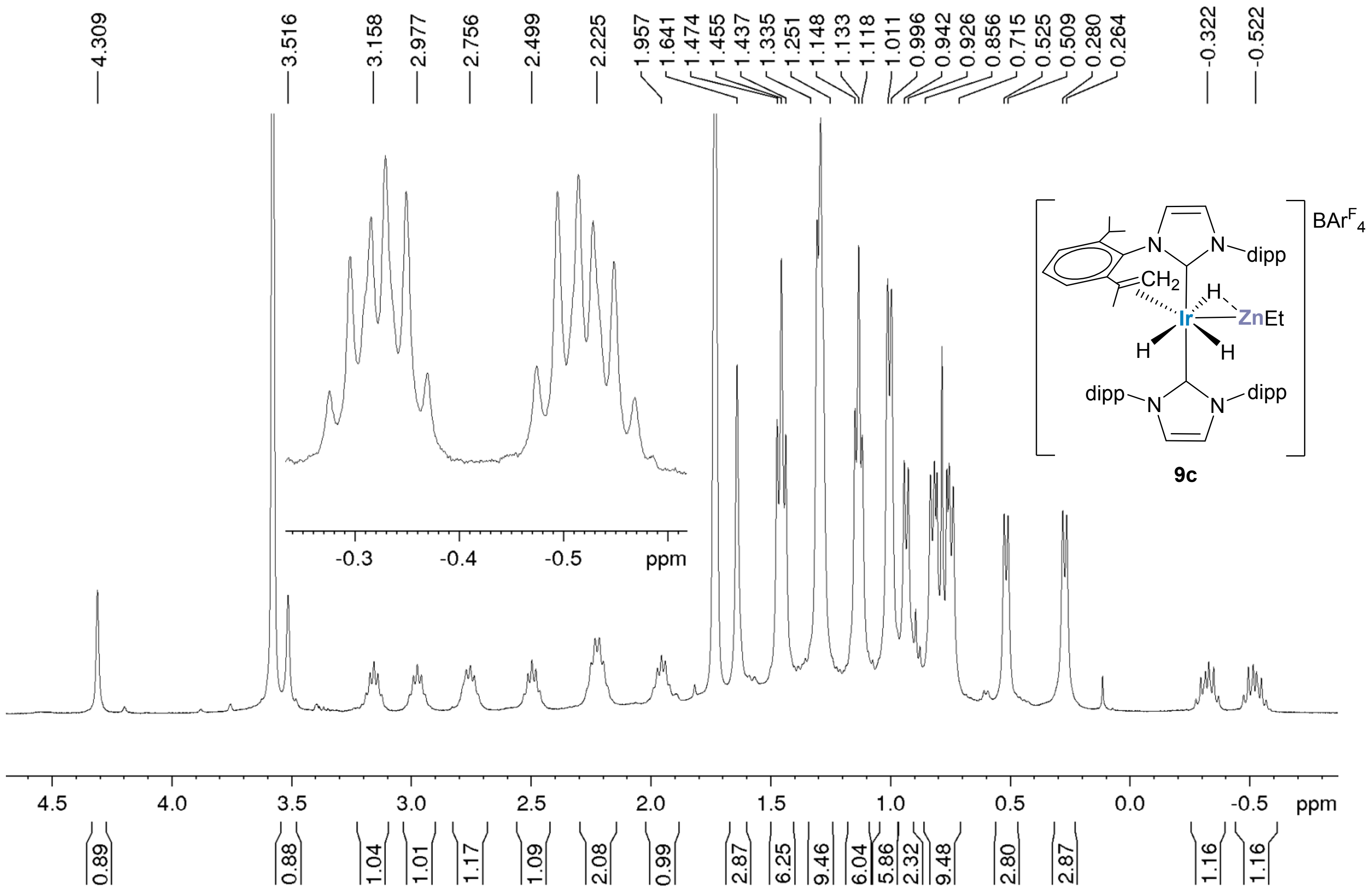

Figure S70. ^{1}H NMR alkyl resonances (THF-d_8, 400 MHz, 248 K) of [Ir(IPr)(IPr'')(ZnEt)H_3][BAr$^F_4$] (**9c**) generated by low temperature addition of H_2 to [Ir(IPr)(IPr'')(ZnEt)H][BAr$^F_4$] (**4c**). Inset shows expansion of the diastereotopic Zn–CH_2 protons.

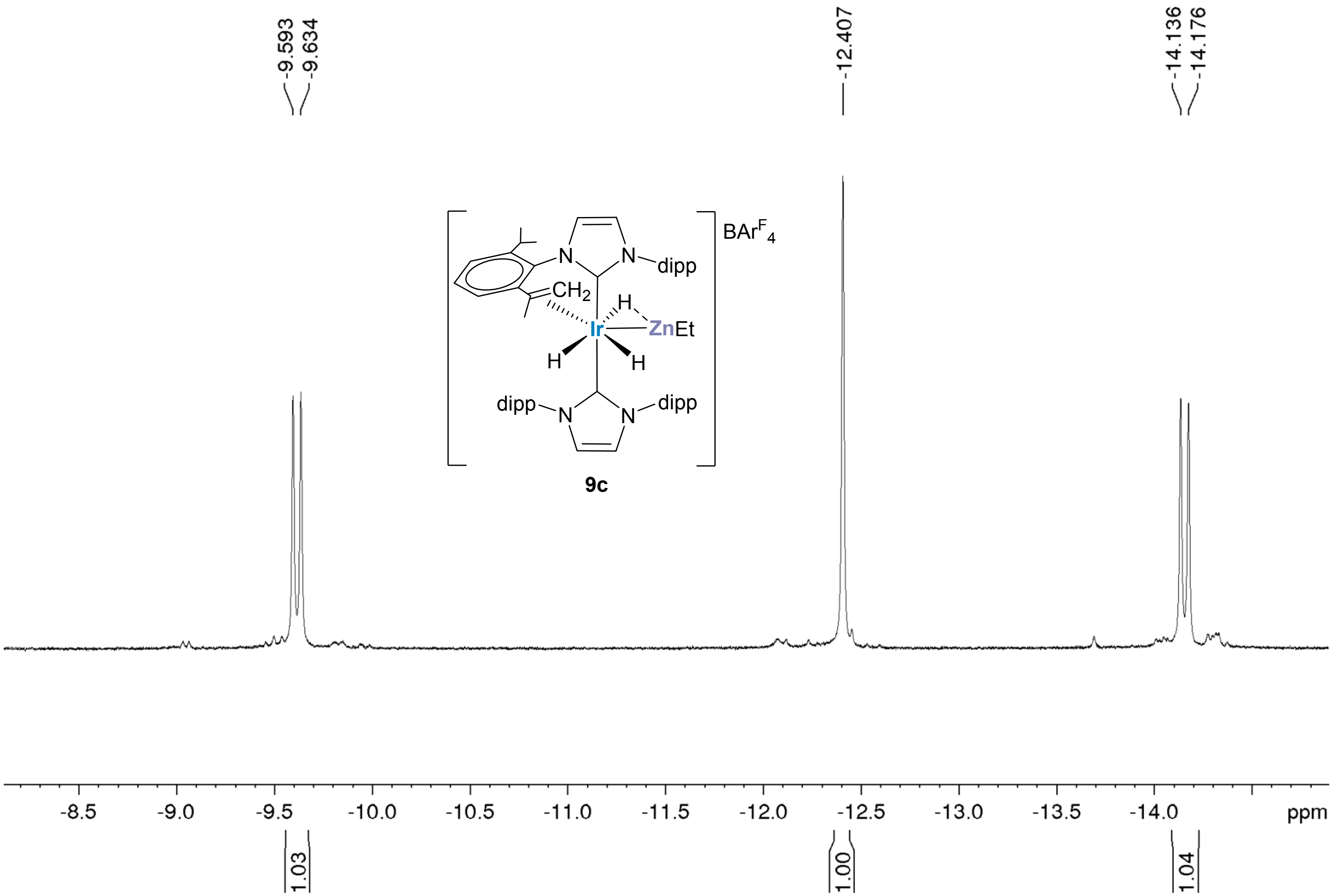

Figure S71. ^{1}H NMR hydride resonances (THF-d_8, 400 MHz, 248 K) of [Ir(IPr)(IPr'')(ZnEt)H_3][BAr$^{F}{}_{4}$] (**9c**) generated by low temperature addition of H_2 to [Ir(IPr)(IPr'')(ZnEt)H][BAr$^{F}{}_{4}$] (**4c**).

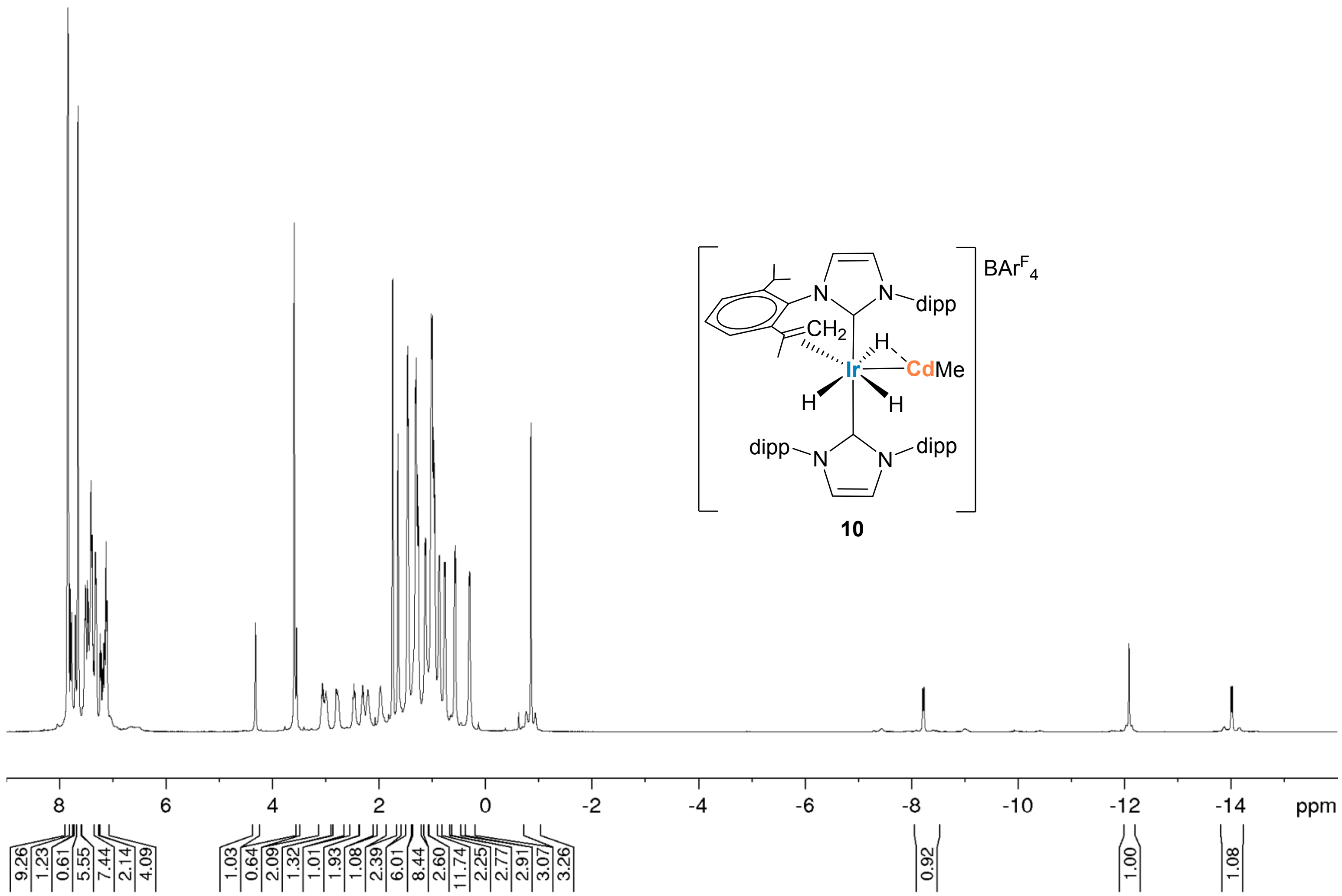

Figure S72. ^{1}H NMR spectrum (THF-d_8, 400 MHz, 248 K) of [Ir(IPr)(IPr'')(CdMe)H_3][BAr$^F{}_4$] (**10**) generated by low temperature addition of H_2 to [Ir(IPr)(IPr'')(CdMe)H][BAr$^F{}_4$] (**8**).

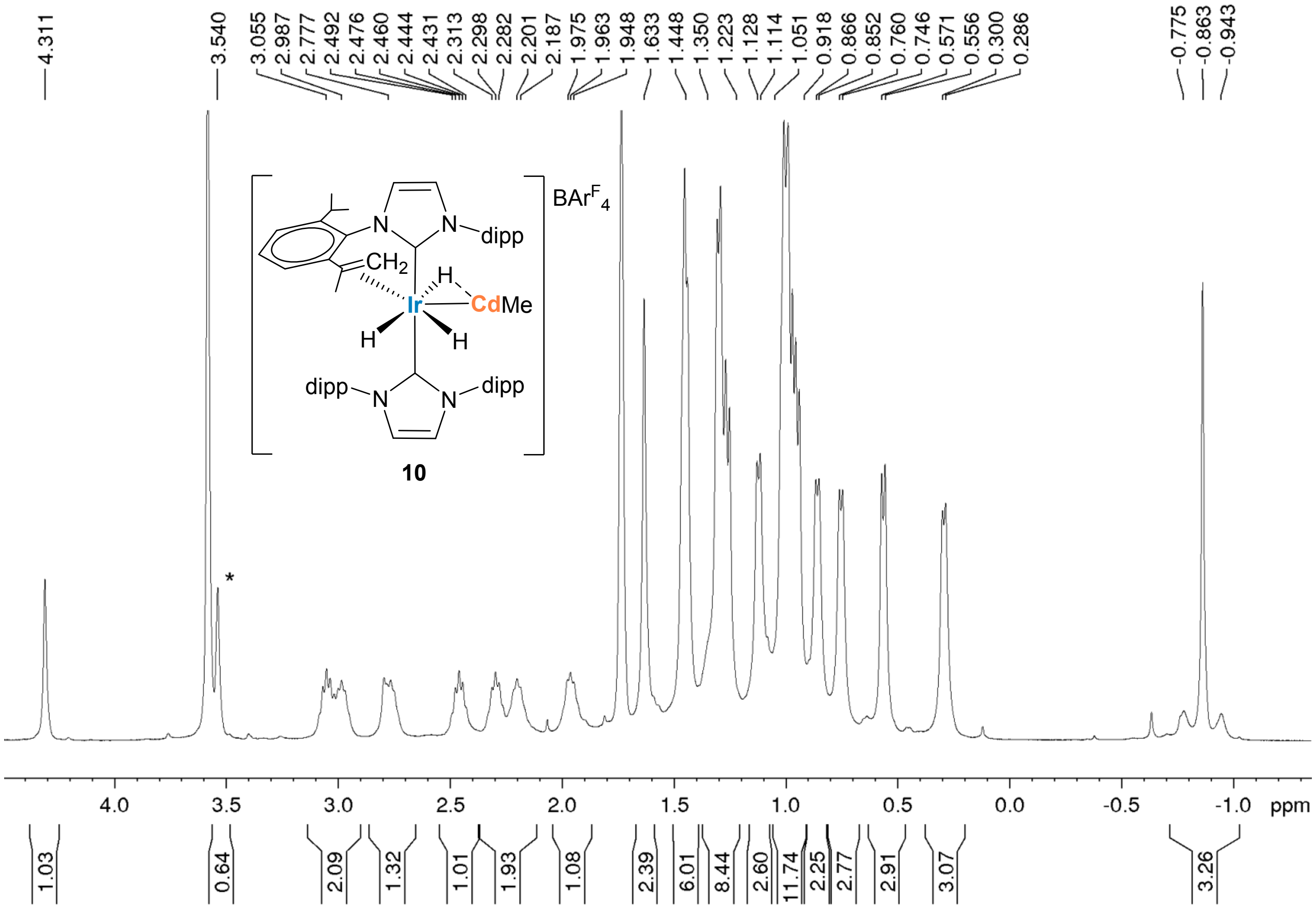

Figure S73. Alkyl region of the ^{1}H NMR spectrum (THF-d_8, 400 MHz, 248 K) of [Ir(IPr)(IPr")(CdMe)H_3][BAr$^F{}_4$] (**10**) generated by low temperature addition of H_2 to [Ir(IPr)(IPr")(CdMe)H][BAr$^F{}_4$] (**8**). Note the low integral of the –C(Me)=C*H*H resonance at δ 3.54 (denoted *) is due to partial overlap with residual protio THF in the solvent at δ 3.58.

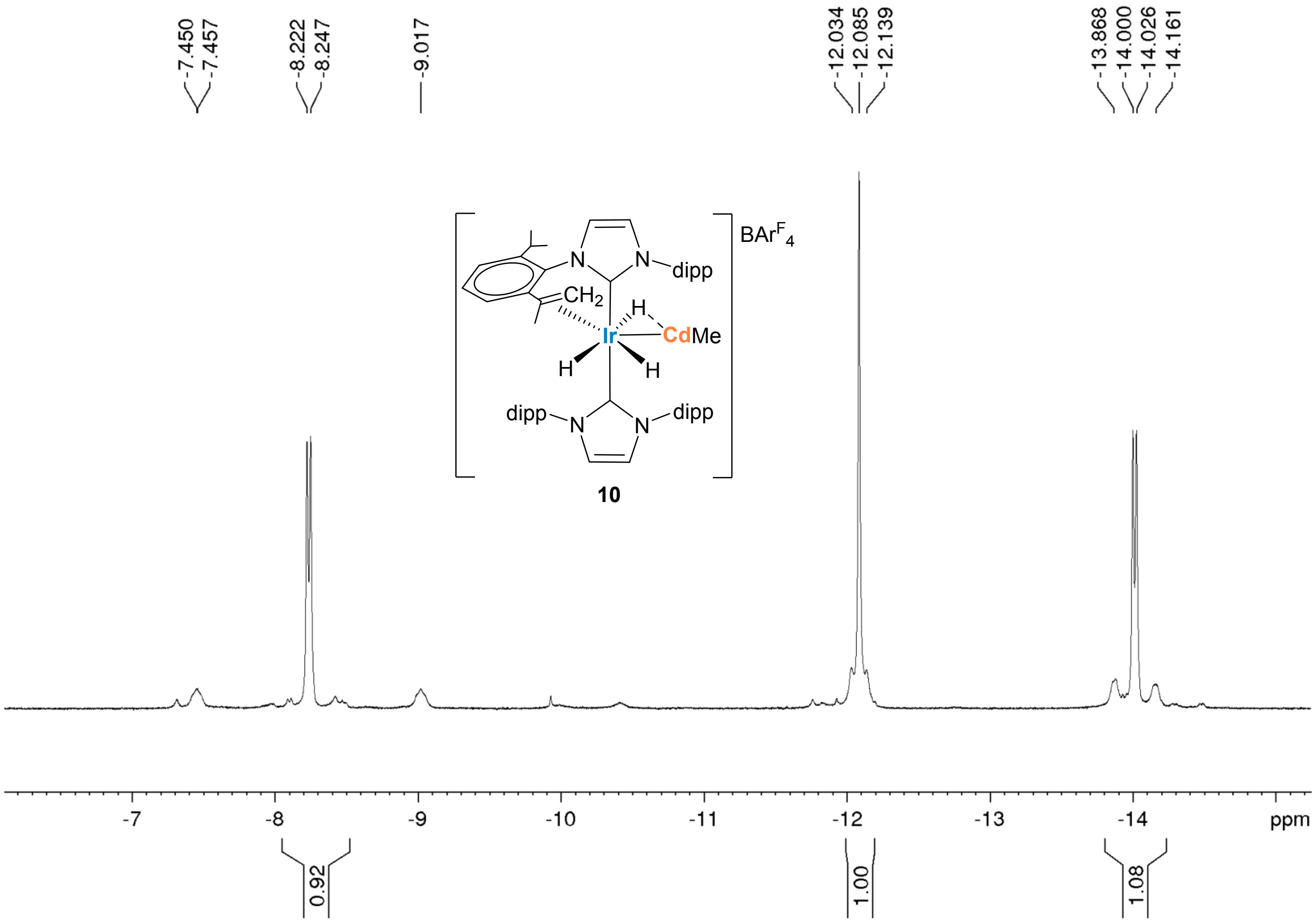

Figure S74. Hydride region of the ^{1}H NMR spectrum (THF-d_8, 400 MHz, 248 K) of $[Ir(IPr)(IPr'')(CdMe)H_3][BAr^F_4]$ (**10**) generated by low temperature addition of H_2 to $[Ir(IPr)(IPr'')(CdMe)H][BAr^F_4]$ (**8**).

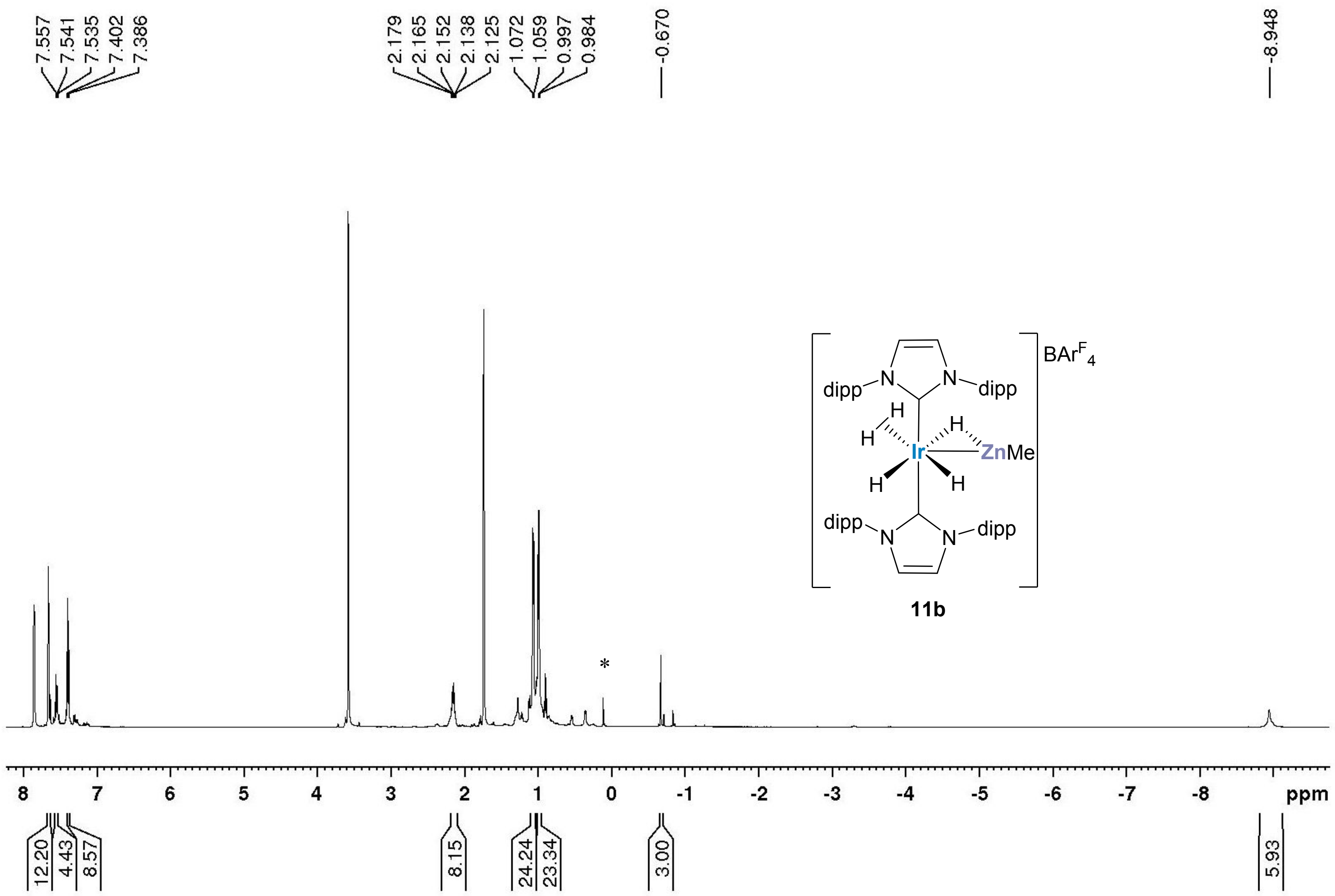

Figure S75. ^{1}H NMR spectrum (THF-d_8, 500 MHz, 226 K) of [Ir(IPr)$_2$(ZnMe)(η^2-H$_2$)H$_3$][BAr$^F{}_4$] (**11b**) (* = silicone grease).

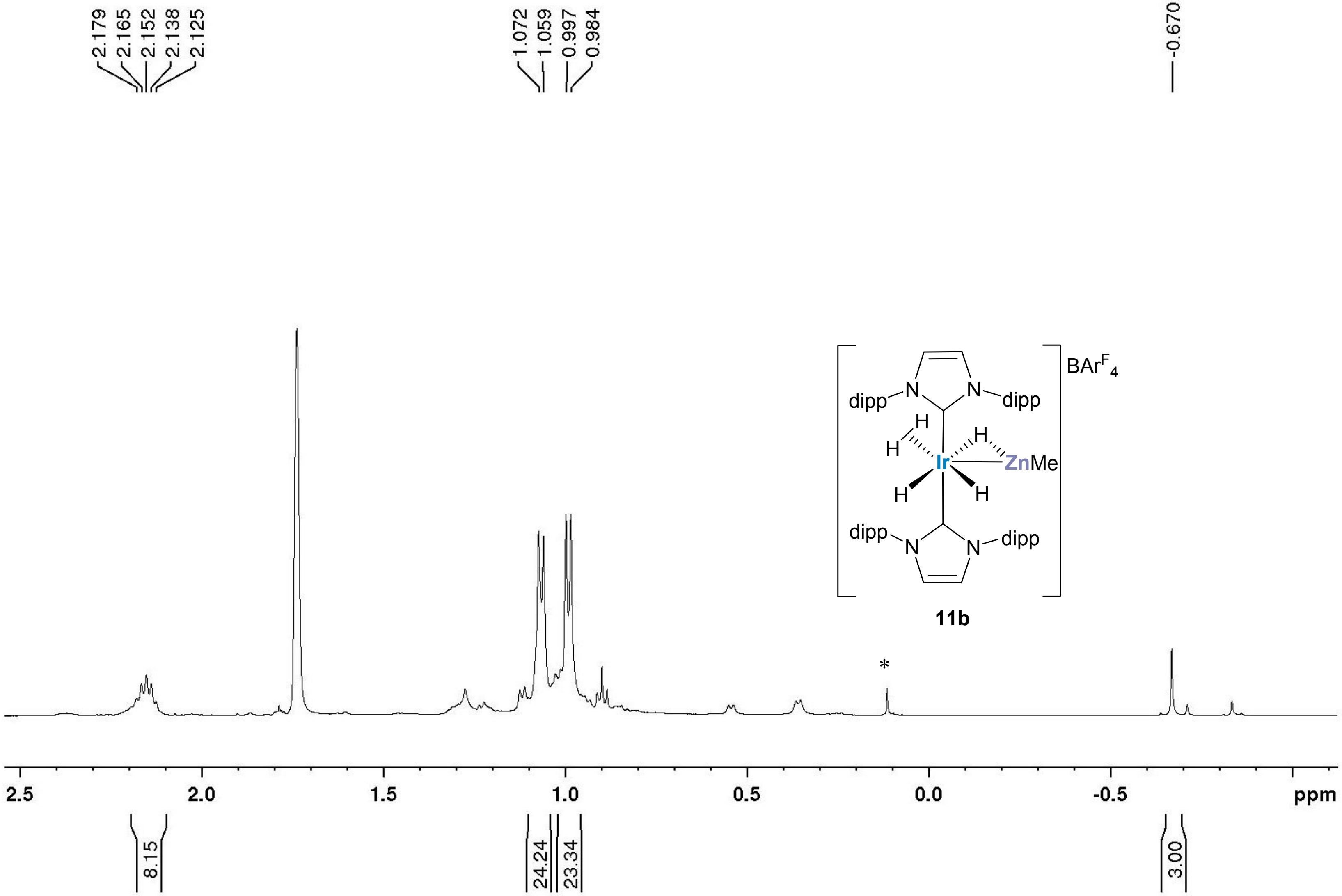

Figure S76. Alkyl region of the ^{1}H NMR spectrum (THF-d_8, 500 MHz, 226 K) of [Ir(IPr)$_2$(ZnMe)(η^2-H$_2$)H$_3$][BAr$^F{}_4$] (**11b**) (* = silicone grease).

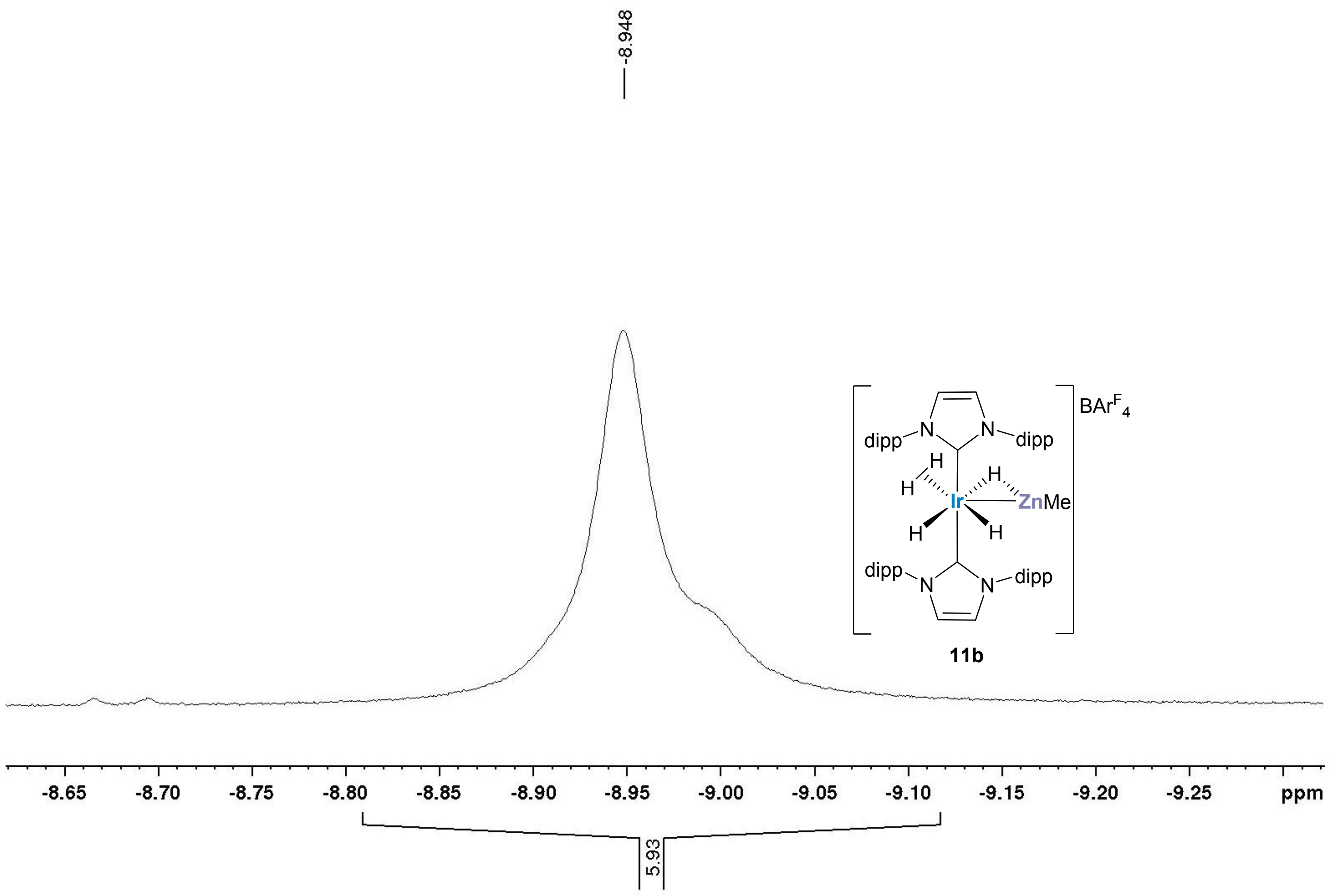

Figure S77. Hydride region of the ^{1}H NMR spectrum (THF-d_8, 500 MHz, 226 K) of [Ir(IPr)$_2$(ZnMe)(η^2-H_2)H_3][BAr$^F_4$] (**11b**).

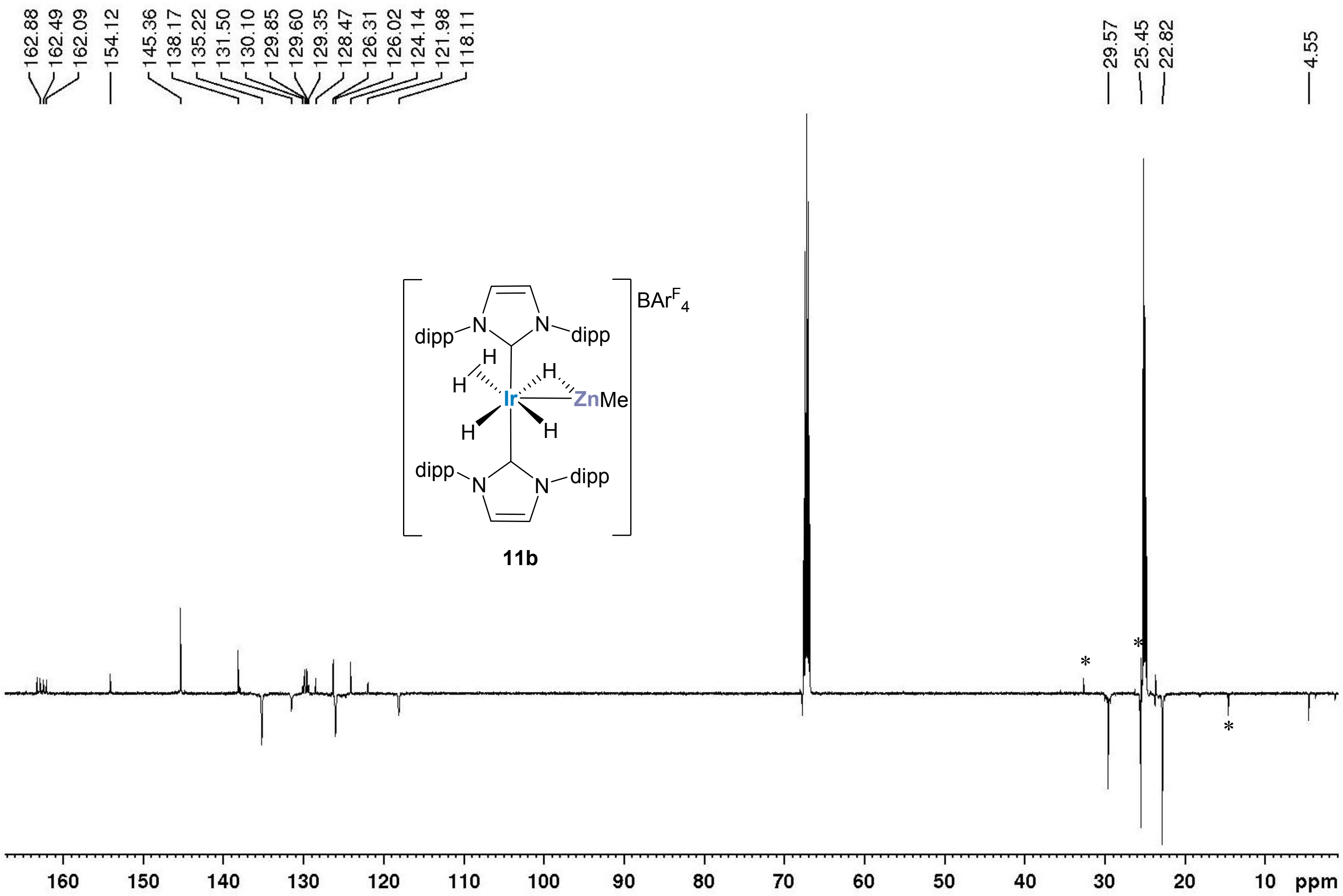

Figure S78. $^{13}C\{^{1}H\}$ DEPTQ NMR spectrum (THF-d_8, 126 MHz, 226 K) of $[Ir(IPr)_2(ZnMe)(\eta^2\text{-}H_2)H_3][BAr^F{}_4]$ (**11b**) (* = hexane).

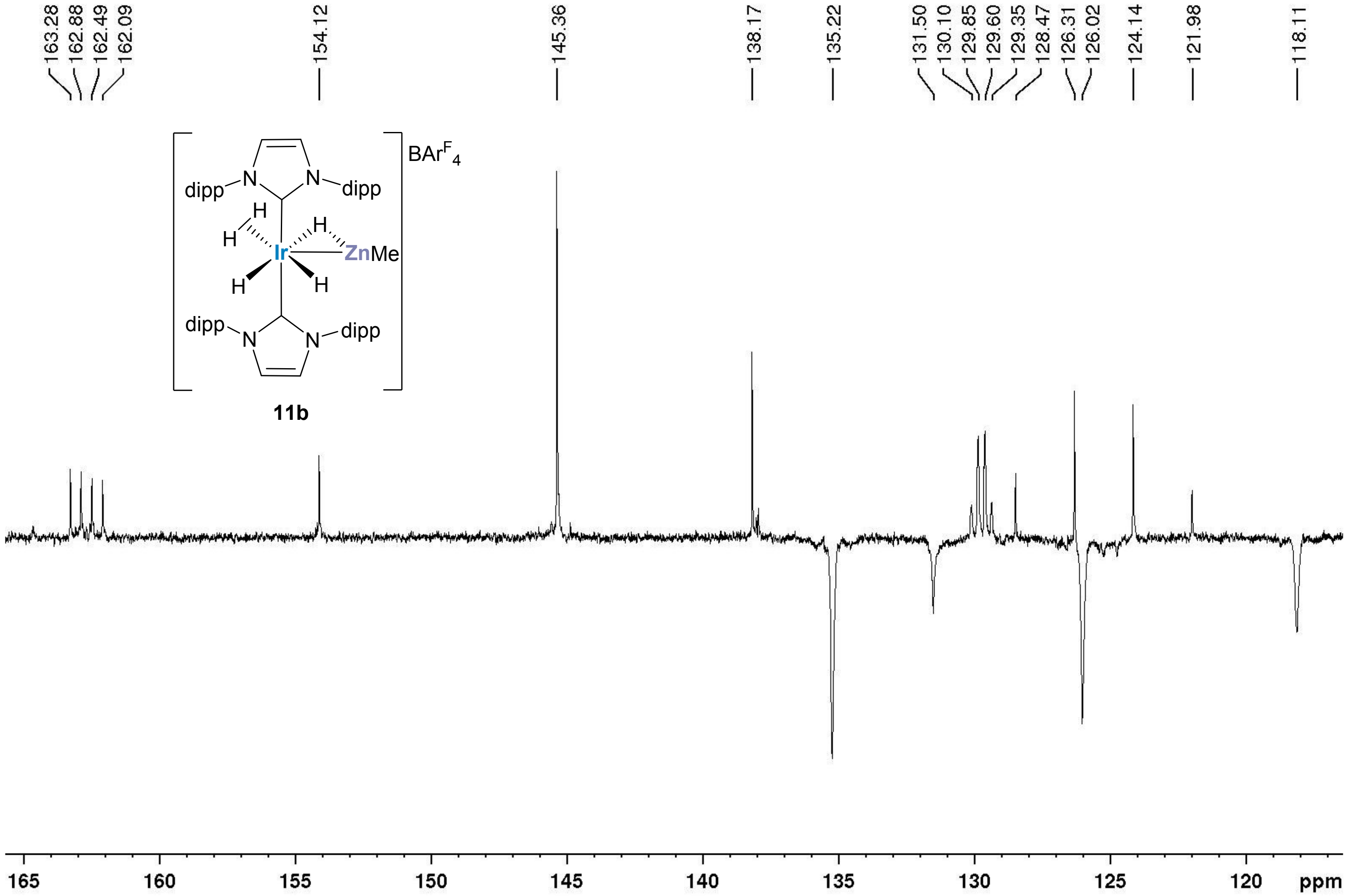

Figure S79. Aromatic region of the $^{13}C\{^{1}H\}$ DEPTQ NMR spectrum (THF-d_8, 126 MHz, 226 K) of $[Ir(IPr)_2(ZnMe)(\eta^2\text{-}H_2)H_3][BAr^F{}_4]$ (**11b**).

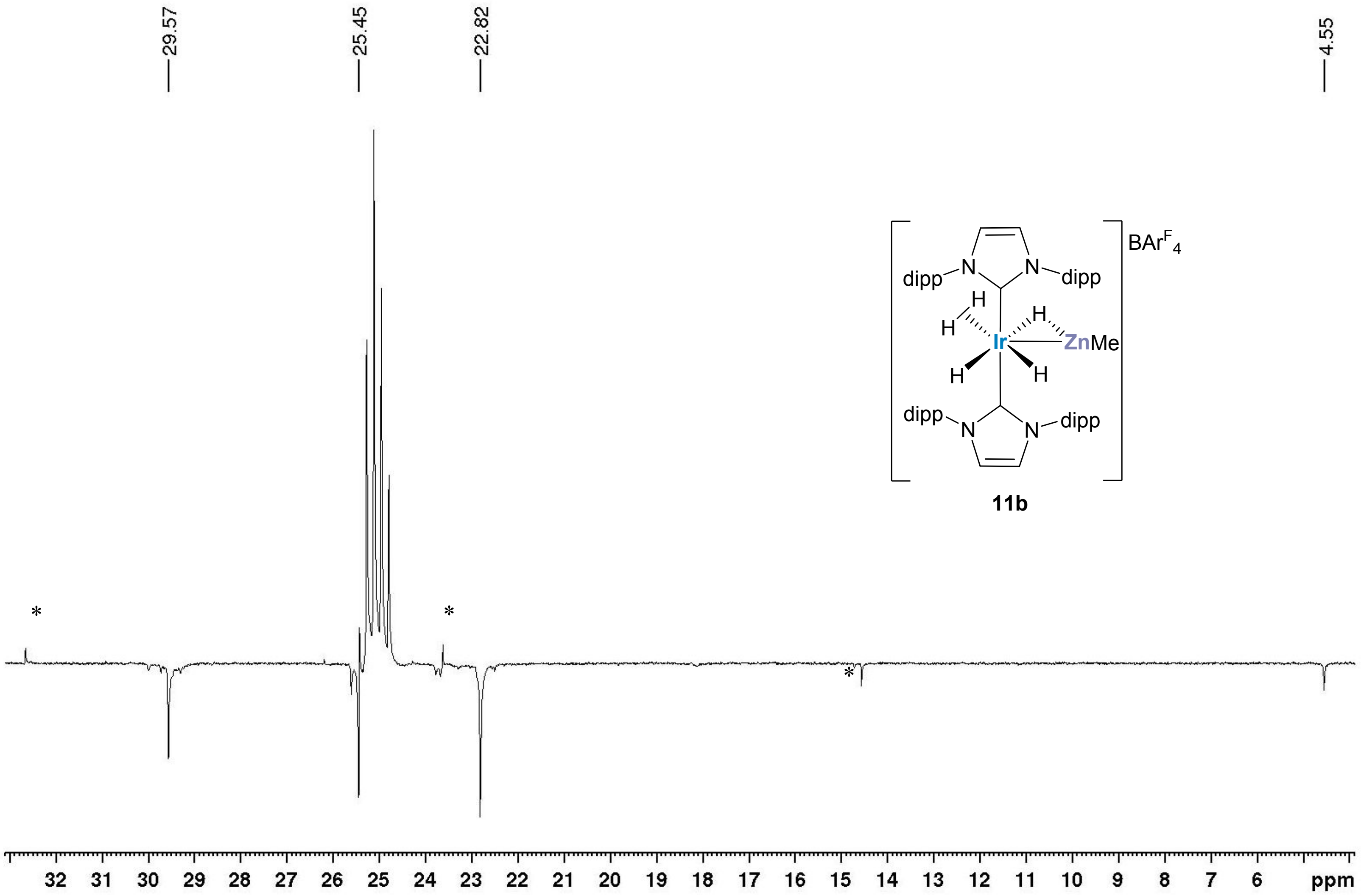

Figure S80. Alkyl region of the $^{13}C\{^{1}H\}$ DEPTQ NMR spectrum (THF-d_8, 126 MHz, 226 K) of $[Ir(IPr)_2(ZnMe)(\eta^2\text{-}H_2)H_3][BAr^F{}_4]$ (**11b**) (* = hexane).

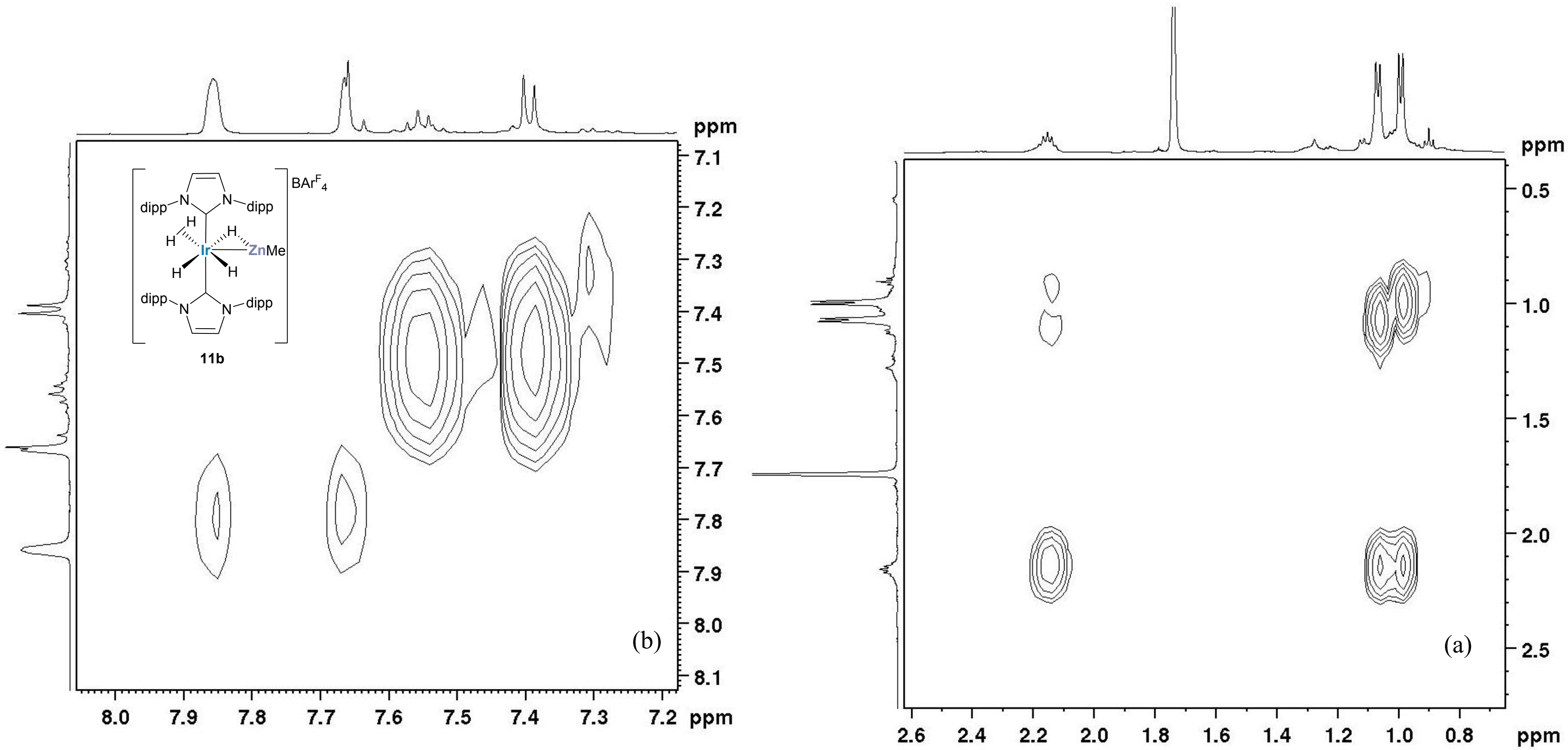

Figure S81. (a) Alkyl and (b) aryl regions of the ^{1}H COSY spectrum (THF-d_8, 500 MHz, 226 K) of $[Ir(IPr)_2(ZnMe)(\eta^2\text{-}H_2)H_3][BAr^F{}_4]$ (**11b**).

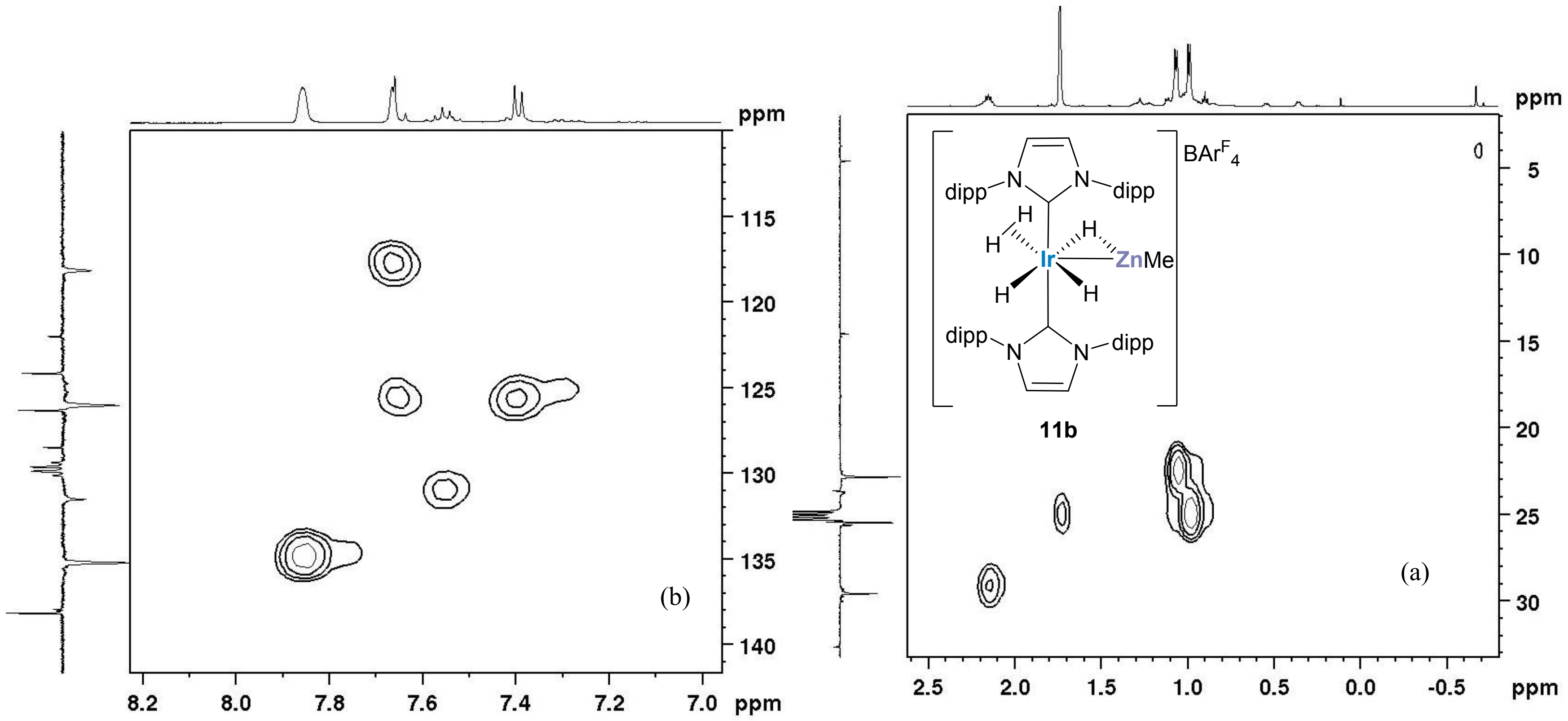

Figure S82. (a) Alkyl and (b) aryl regions of the ^{13}C-^{1}H HSQC spectrum (THF-d_8, 226 K) of [Ir(IPr)$_2$(ZnMe)(η^2-H$_2$)H$_3$][BAr$^F{}_4$] (**11b**).

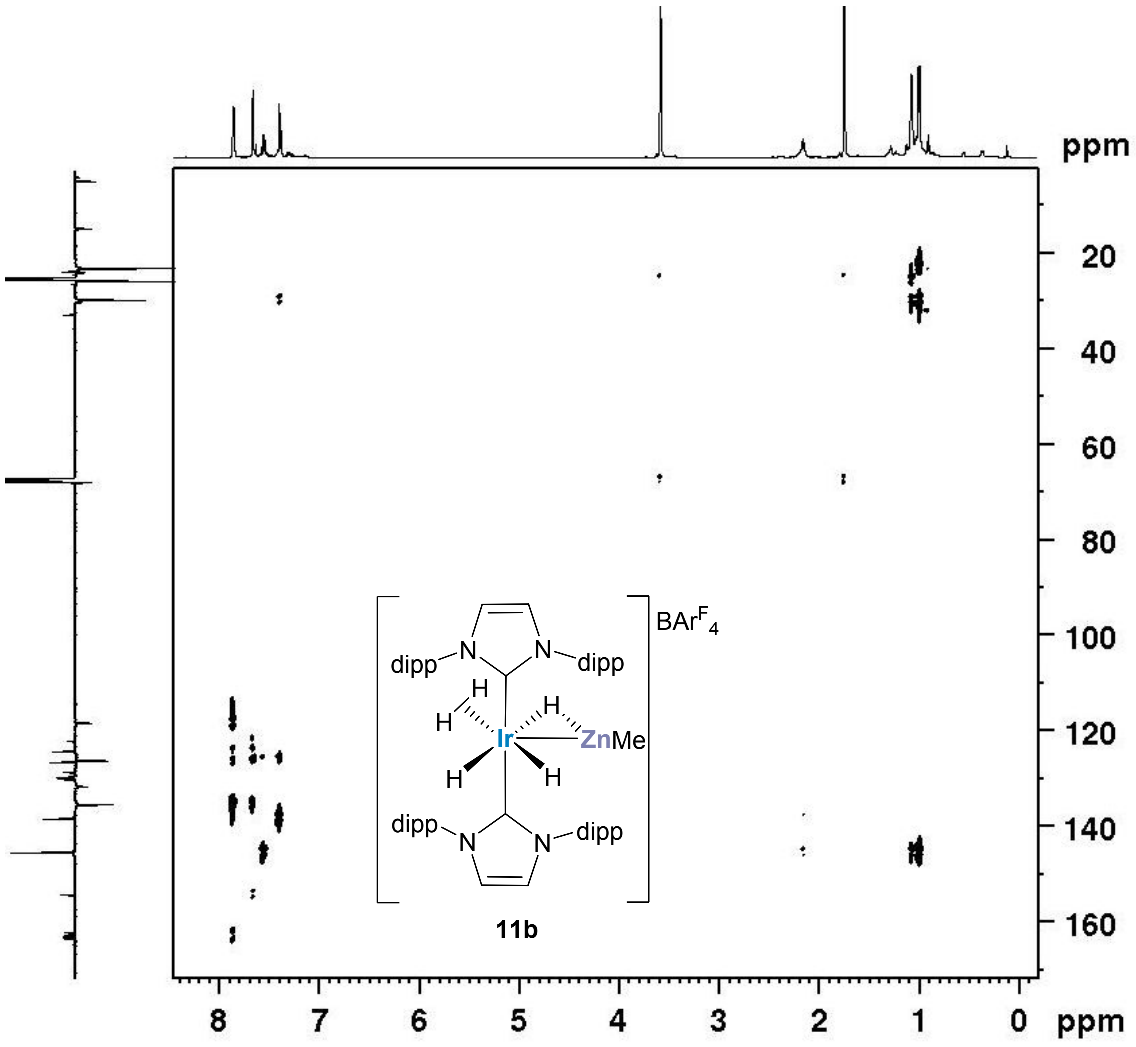

Figure S83. ^{13}C-^{1}H HMBC spectrum (THF-d_8, 226 K) of [Ir(IPr)$_2$(ZnMe)(η^2-H_2)H_3][BAr$^F{}_4$] (**11b**).

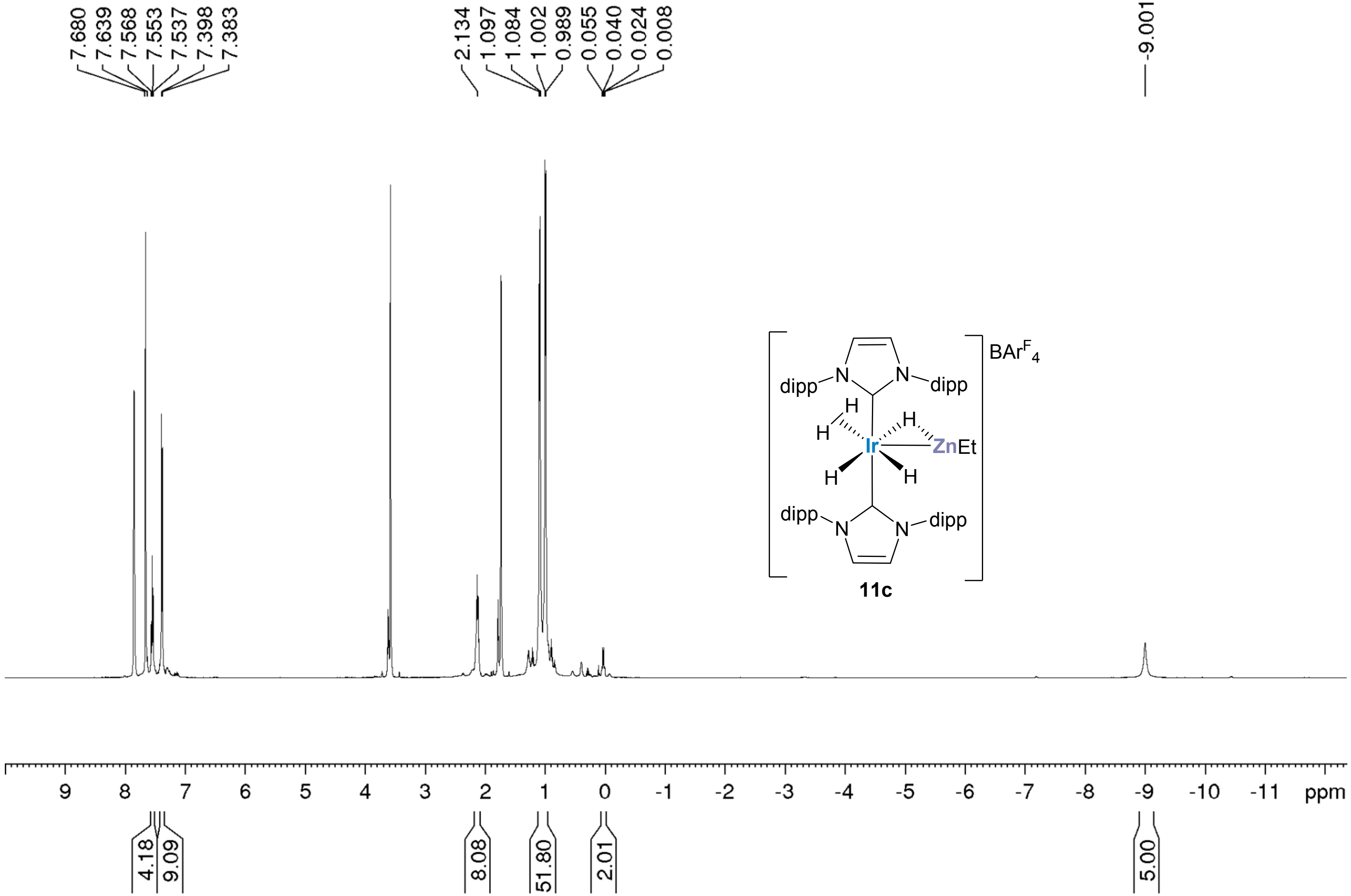

Figure S84. ^{1}H NMR spectrum (THF-d_8, 500 MHz, 226 K) of $[Ir(IPr)_2(ZnEt)(\eta^2\text{-}H_2)H_3][BAr^F_4]$ (**11c**).

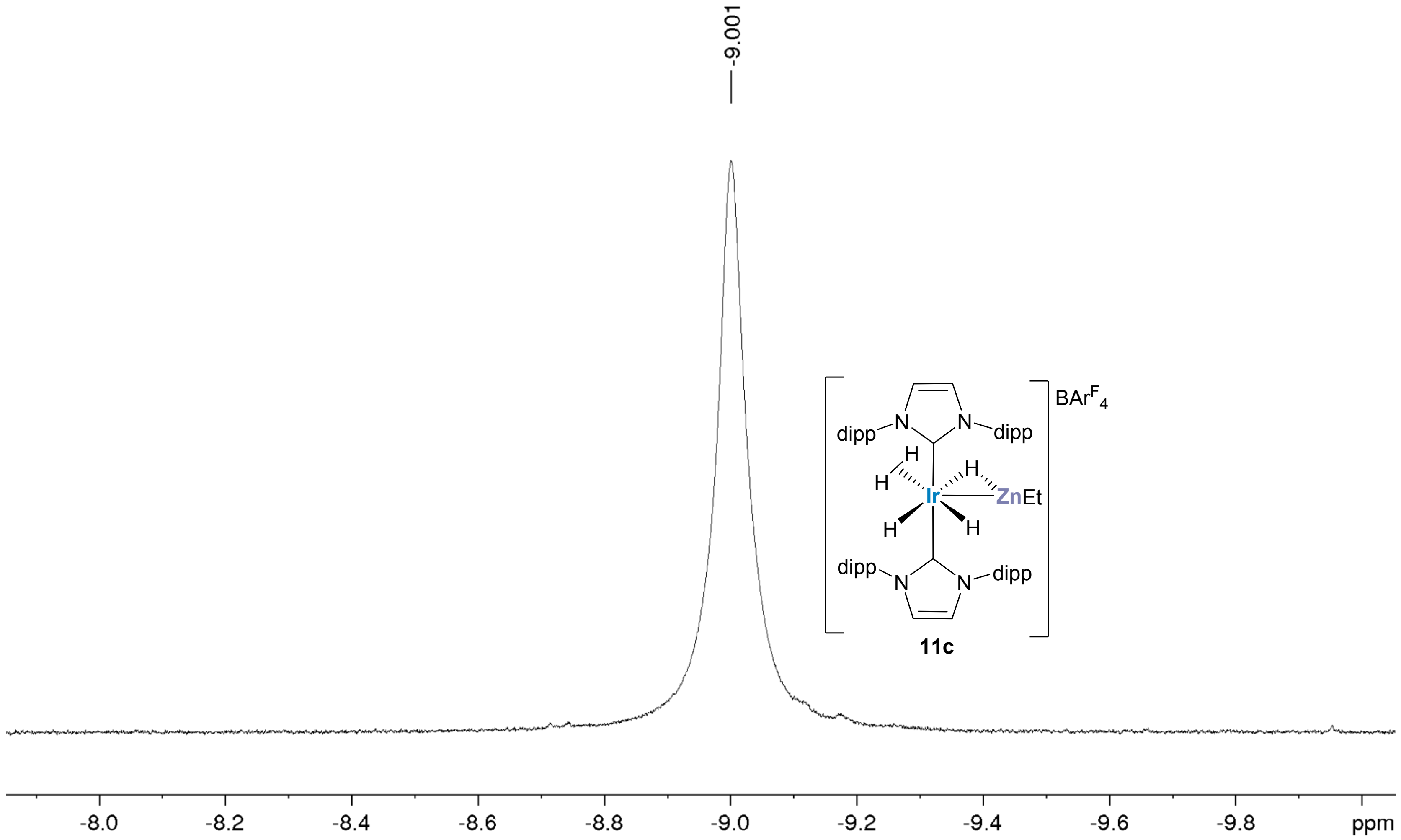

Figure S85. Hydride resonance (THF-d_8, 500 MHz, 226 K) of $[Ir(IPr)_2(ZnEt)(\eta^2\text{-}H_2)H_3][BAr^F{}_4]$ (**11c**).

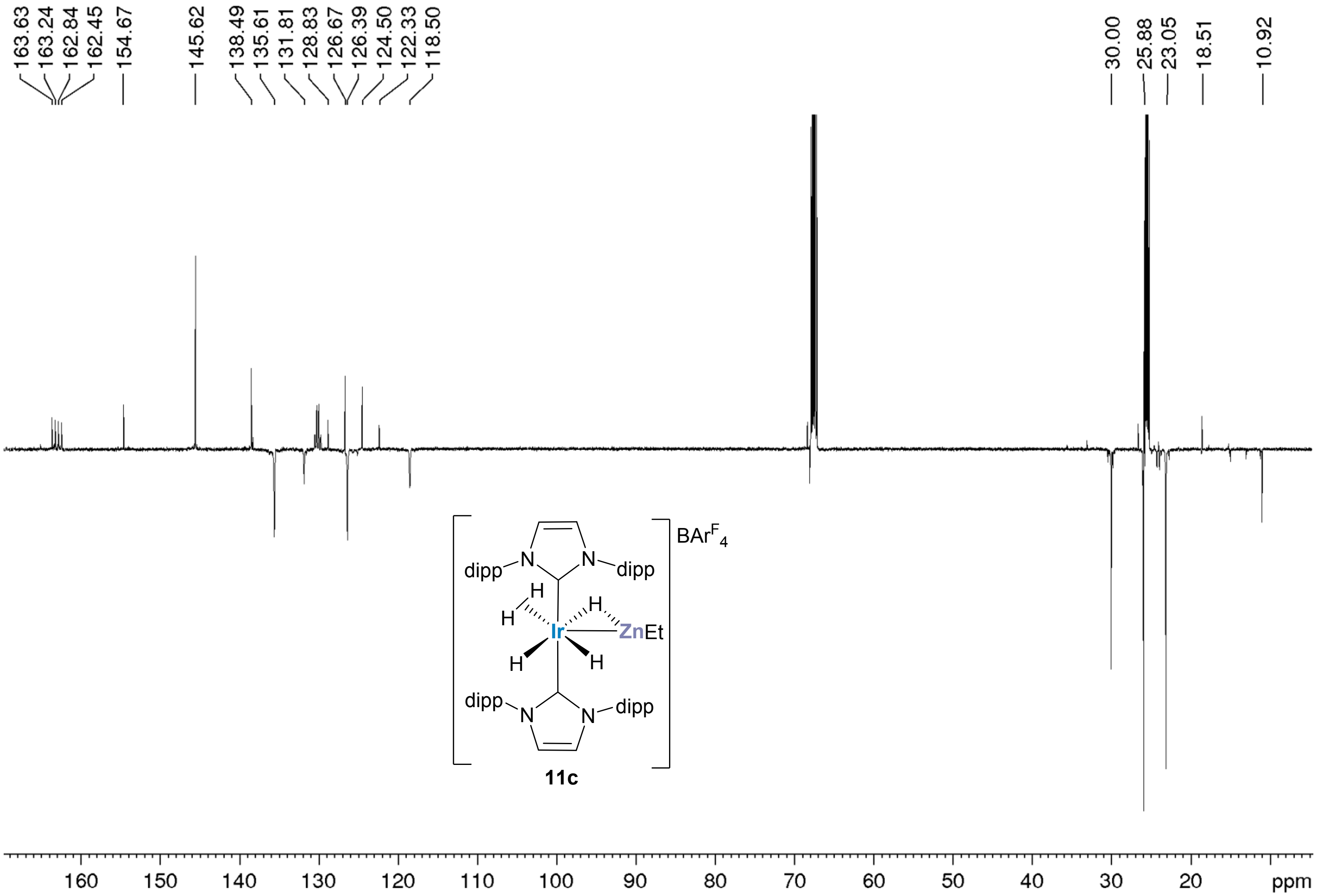

Figure S86. $^{13}C\{^1H\}$ DEPTQ NMR spectrum (THF-d_8, 126 MHz, 226 K) of $[Ir(IPr)_2(ZnEt)(\eta^2\text{-}H_2)H_3][BAr^F{}_4]$ (**11c**).

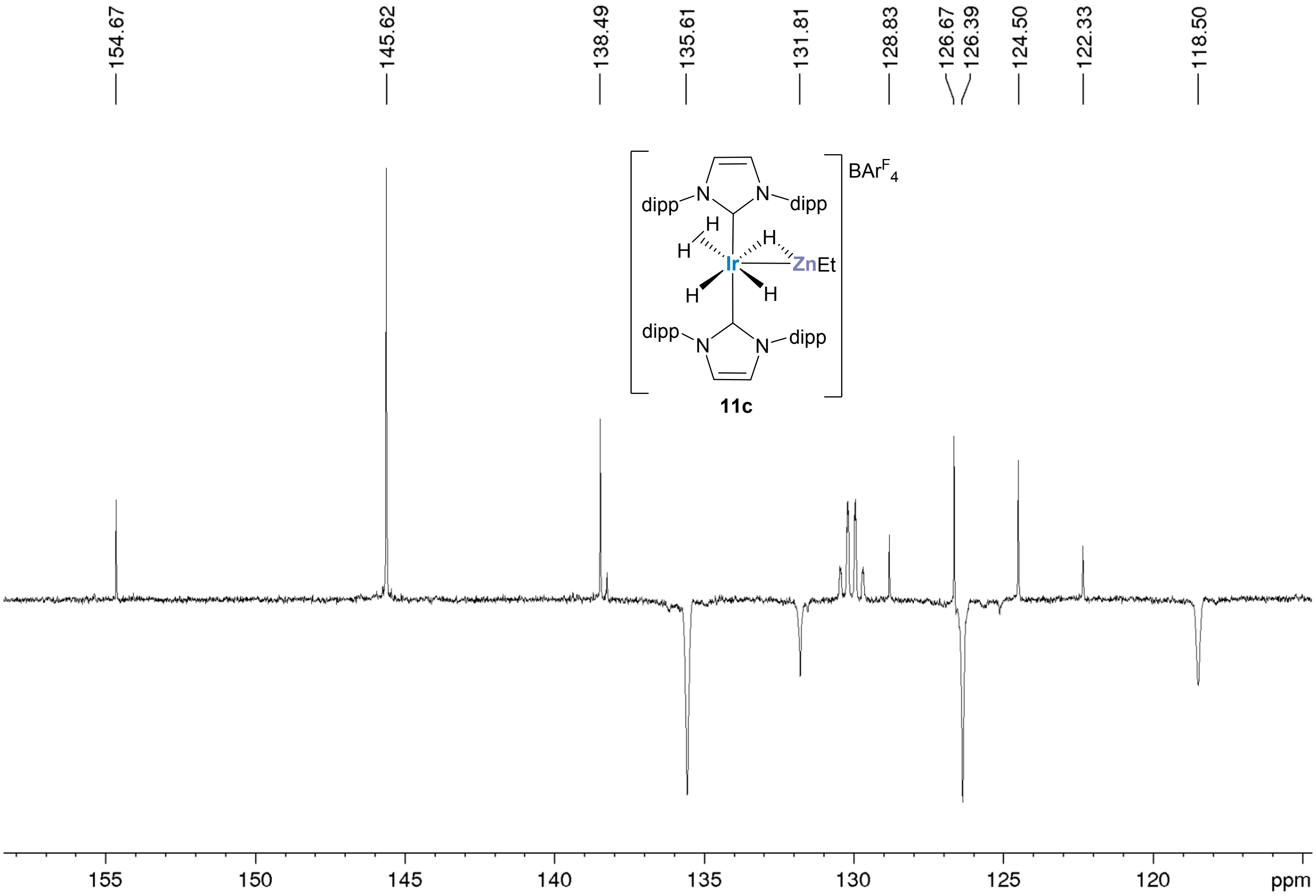

Figure S87. Aromatic region of the $^{13}C\{^{1}H\}$ DEPTQ NMR spectrum (THF-d_8, 126 MHz, 226 K) of $[Ir(IPr)_2(ZnEt)(\eta^2\text{-}H_2)H_3][BAr^F_4]$ (**11c**).

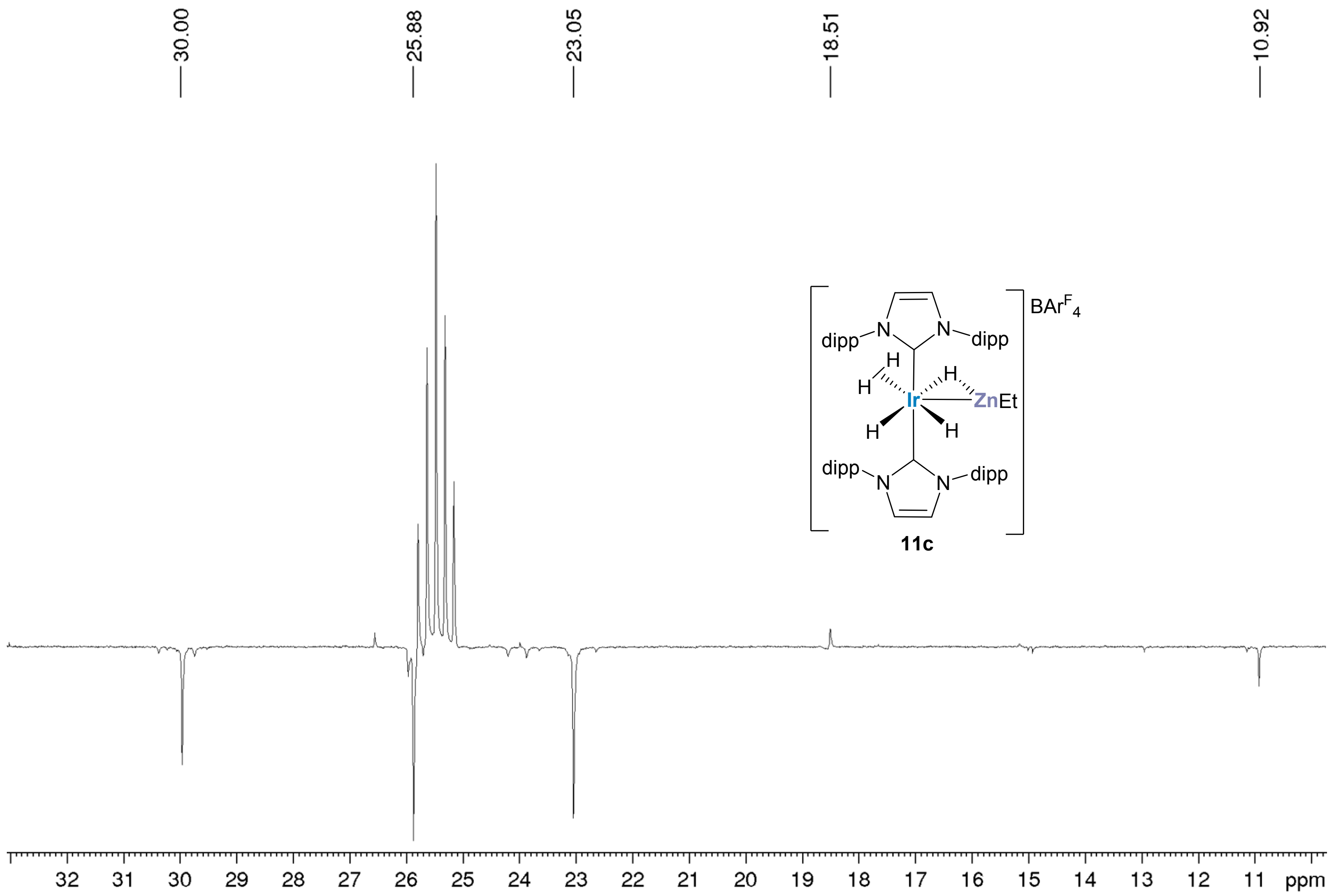

Figure S88. Alkyl region of the $^{13}C\{^{1}H\}$ DEPTQ NMR spectrum (THF-d_8, 126 MHz, 226 K) of $[Ir(IPr)_2(ZnEt)(\eta^2\text{-}H_2)H_3][BAr^F_4]$ (**11c**).

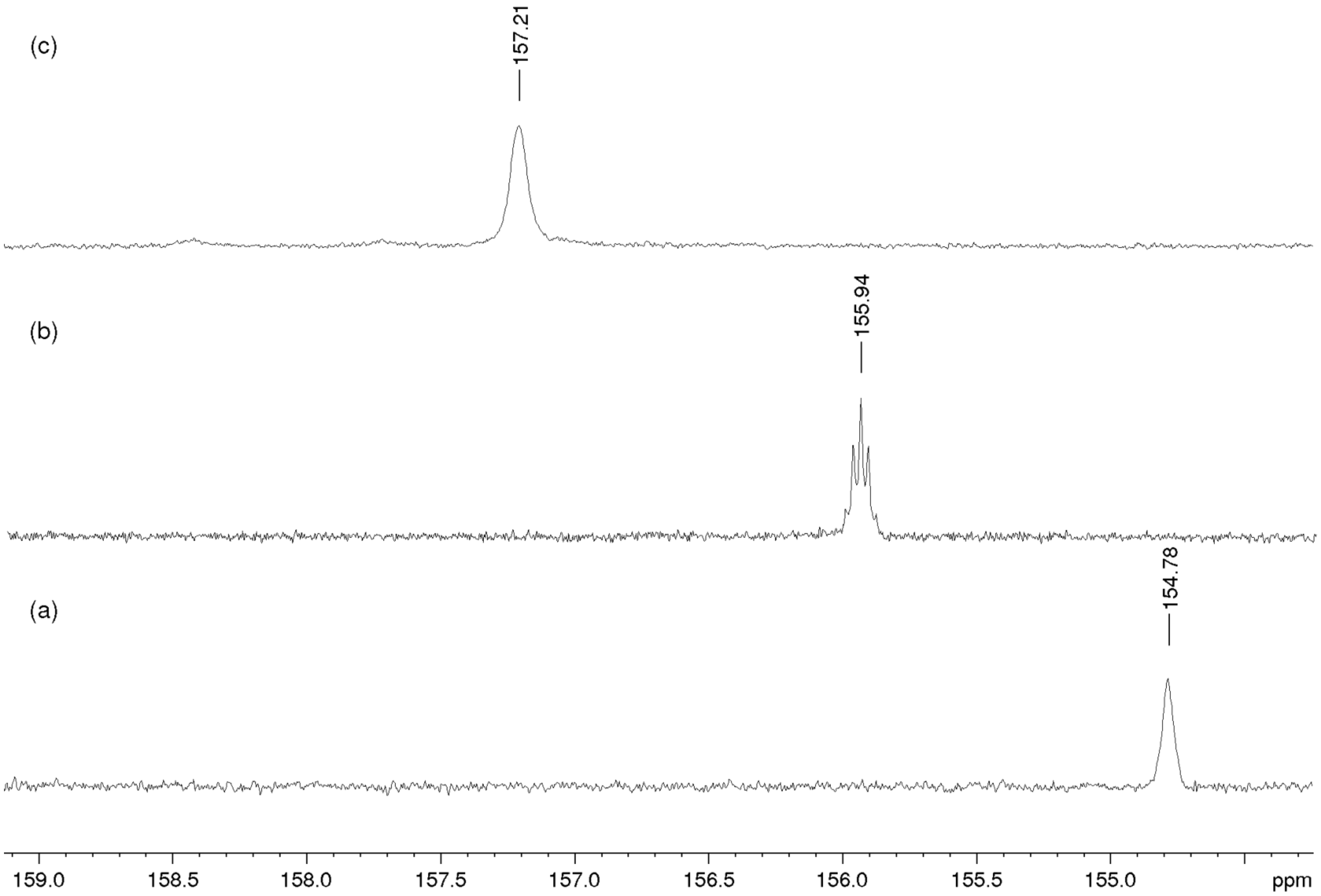

Figure S89. (a) Ir–C_{IPr} resonance in the $^{13}C\{selective\text{-}^{1}H\}^{*}$ (* ^{1}H decoupler set to decouple aromatic protons only (δ 6-9)) NMR spectrum (THF-d_8, 126 MHz, 278 K) of $[Ir(IPr)_2(ZnEt)(\eta^2\text{-}H_2)H_3][BAr^F_4]$ (**11c**). Spectra ((THF-d_8, 126 MHz, 278 K) for previously reported (b) classical tetrahydride $[Ir(IPr)_2(ZnMe)_2H_4][BAr^F_4]$ (**3**)[3] and (c) $[Ir(IPr)_2(\eta^2\text{-}H_2)_2H_2][BAr^F_4]$ (**6**)[3] are shown for comparison.

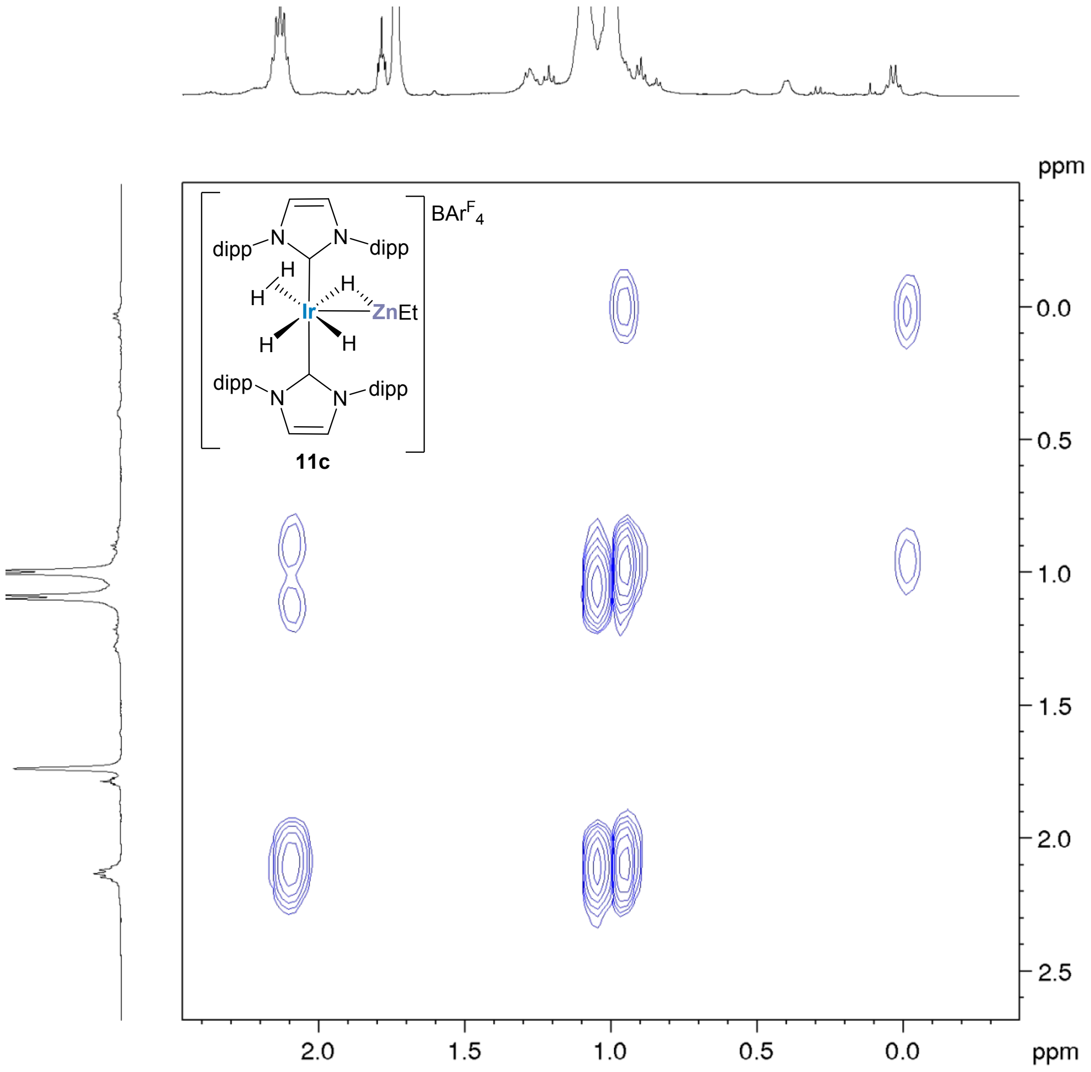

Figure S90. Alkyl region of the ^{1}H COSY spectrum (THF-d_8, 500 MHz, 226 K) of [Ir(IPr)$_2$(ZnEt)(η^2-H_2)H_3][BAr$^F_4$] (**11c**) emphasising the position of the Zn–CH_2Me resonance underneath the ^{i}Pr methyl resonances.

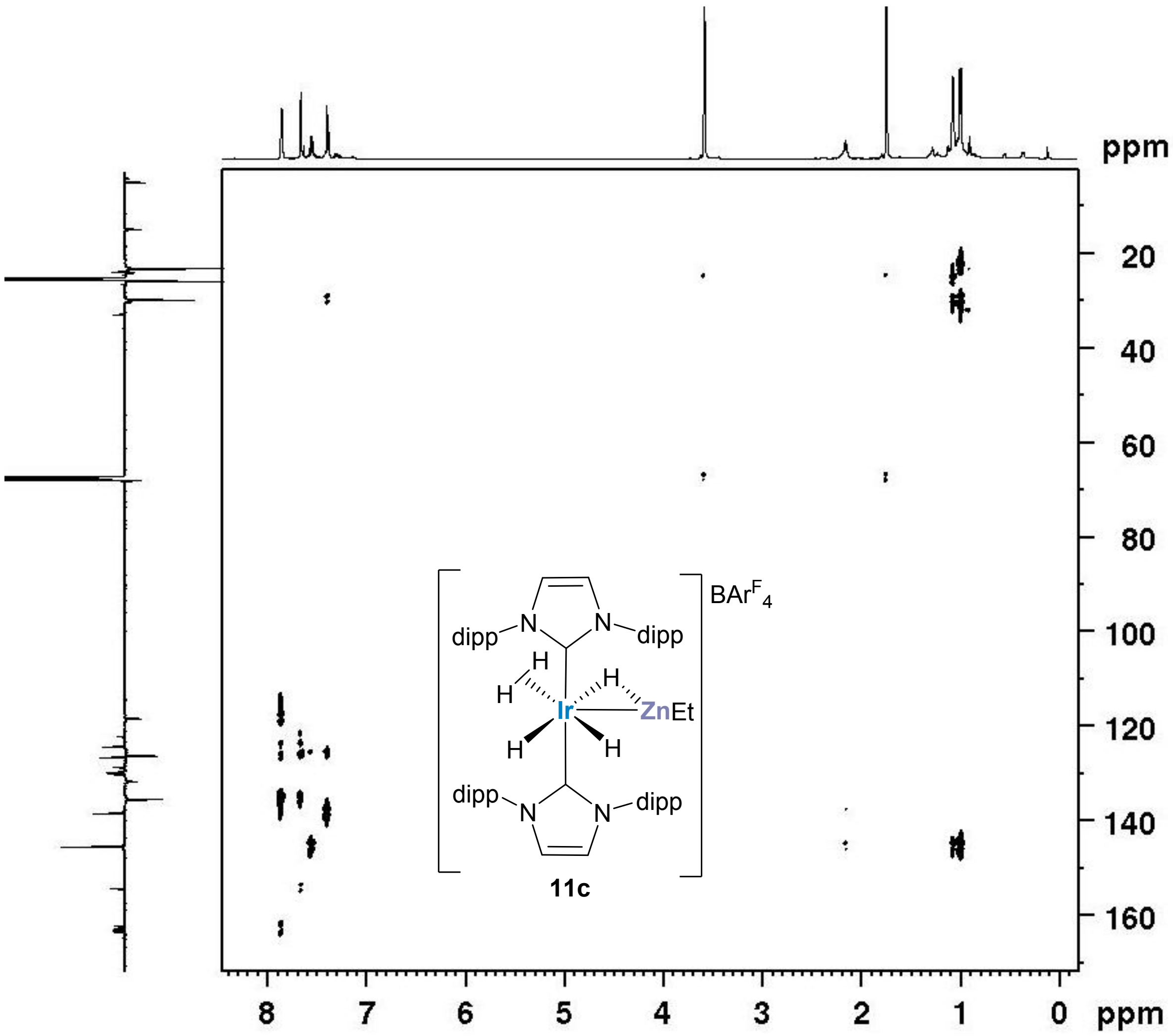

Figure S91. ^{13}C-^{1}H HMBC spectrum (THF-d_8, 226 K) of [Ir(IPr)$_2$(ZnEt)(η^2-H_2)H_3][BAr$^F{}_4$] (**11c**).

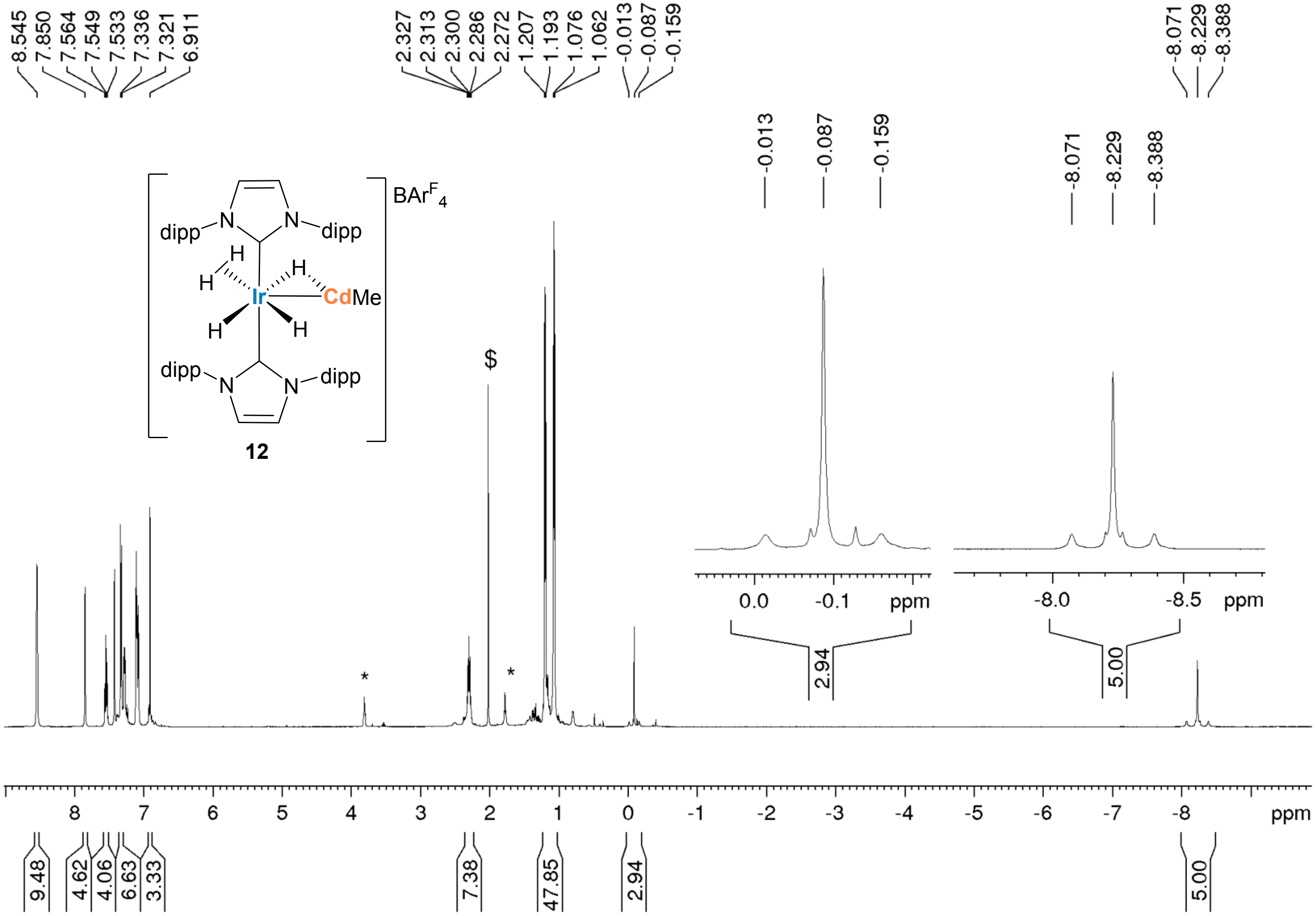

Figure S92. ^{1}H NMR spectrum (C_6D_5F, 500 MHz, 298 K) of [Ir(IPr)$_2$(CdMe)(η^2-H_2)H_3][BAr$^F_4$] (**12**). Expansions of the Cd–*Me* and hydride resonances are shown in the insets (* = THF, $ = acetone).

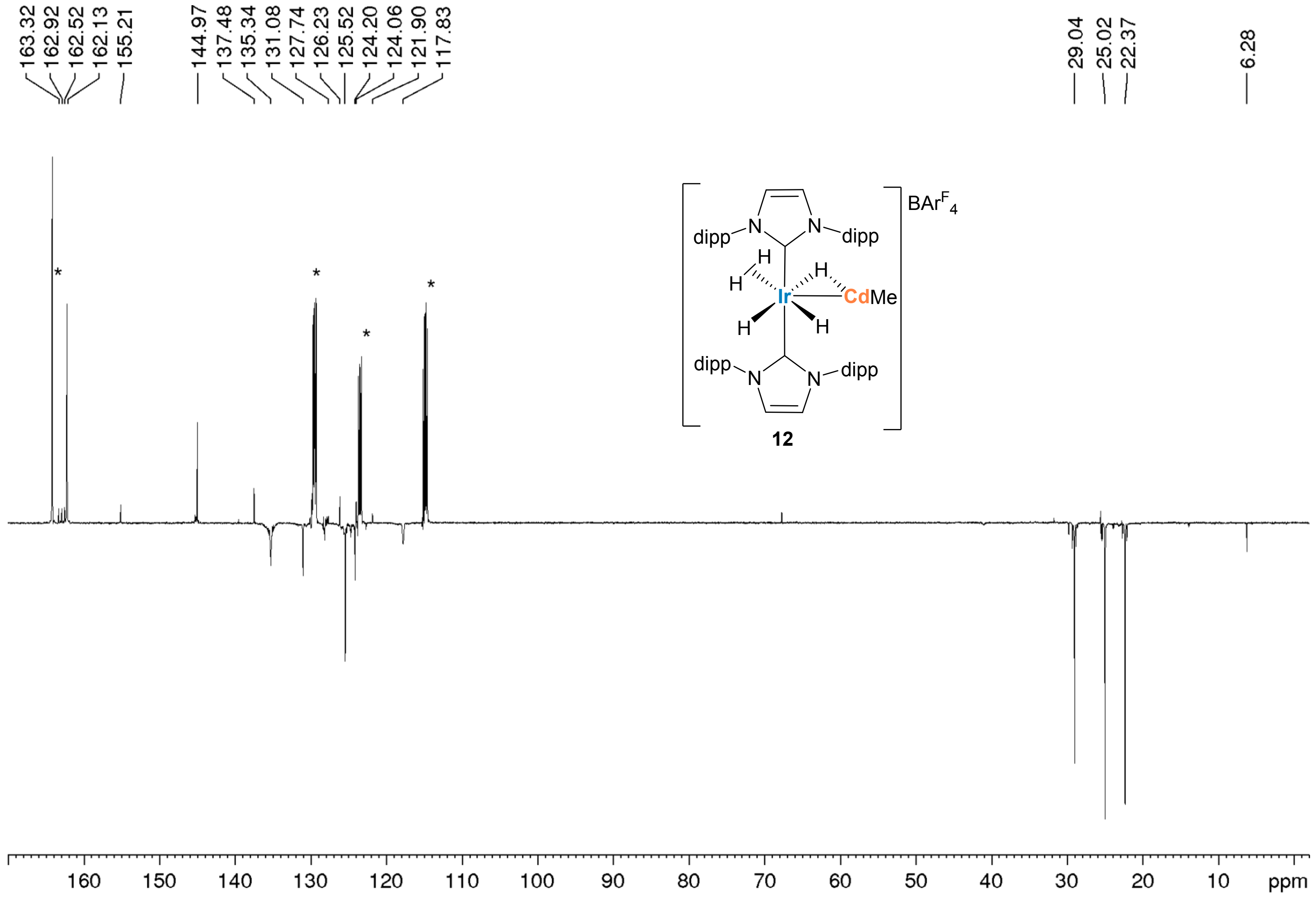

Figure 93. $^{13}C\{^{1}H\}$ DEPTQ spectrum (C_6D_5F, 126 MHz, 298 K) of $[Ir(IPr)_2(CdMe)(\eta^2\text{-}H_2)H_3][BAr^F{}_4]$ (**12**) (* = C_6D_5F).

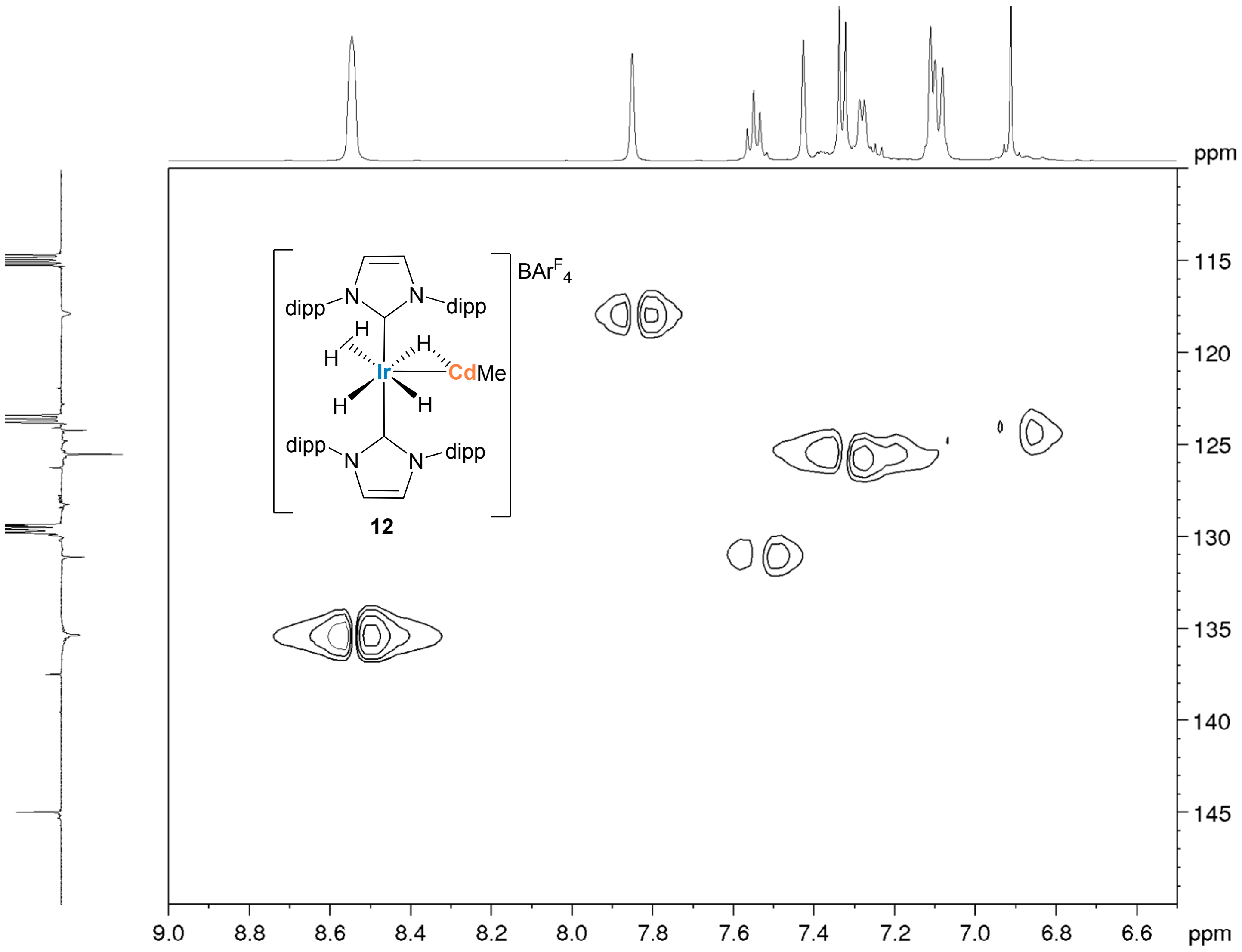

Figure S94. Aromatic region of the ^{13}C-^{1}H HSQC spectrum (C_6D_5F, 298 K) of $[Ir(IPr)_2(CdMe)(\eta^2\text{-}H_2)H_3][BAr^F{}_4]$ (**12**).

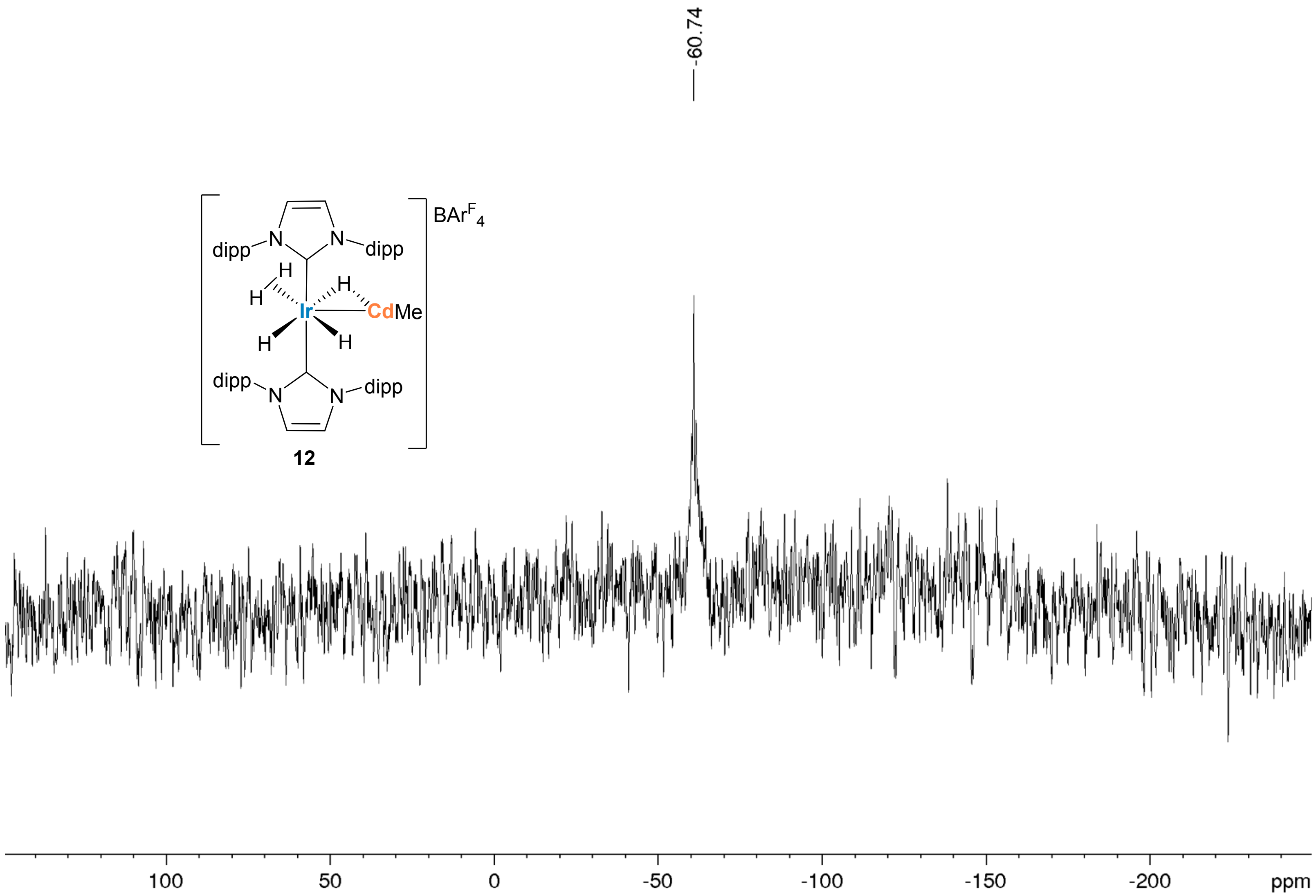

Figure S95. ^{113}Cd{^{1}H} NMR spectrum (C_6D_5F, 111 MHz, 298 K) of [Ir(IPr)$_2$(CdMe)(η^2-H_2)H_3][BAr$^F_4$] (**12**).

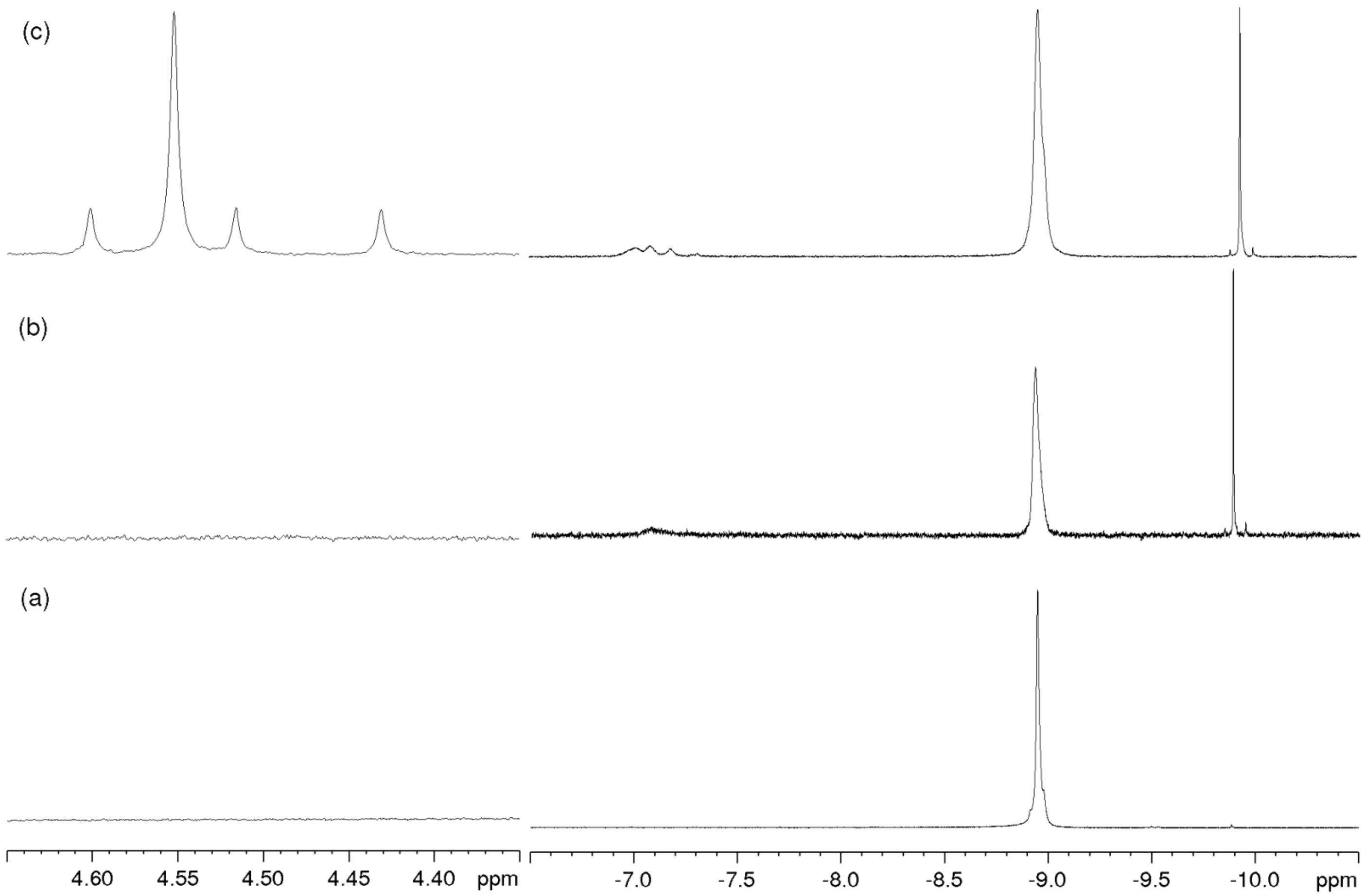

Figure S96. Free H_2 (left) and Ir–*H* (right) regions of the ^{1}H NMR spectrum (500 MHz, THF-d_8, 298 K) of [Ir(IPr)$_2$(ZnEt)(η^2-H_2)H_3][BAr$^F{}_4$] (**11c**) (a) before and then (b) 1 and (c) 3 h after addition of D_2 (1 atm). The spectrum in (c), recorded at 248 K, highlights the formation of both H_2 and HD. Traces of [Ir(IPr)$_2$(η^2-H_2)$_2$$H_2$][BAr$^F{}_4$] (**6**, with some D-incorporation apparent) and Ir(IPr)$_2$$H_5$ (**13**) appear at ca. δ –7 and ca. δ –10 respectively.

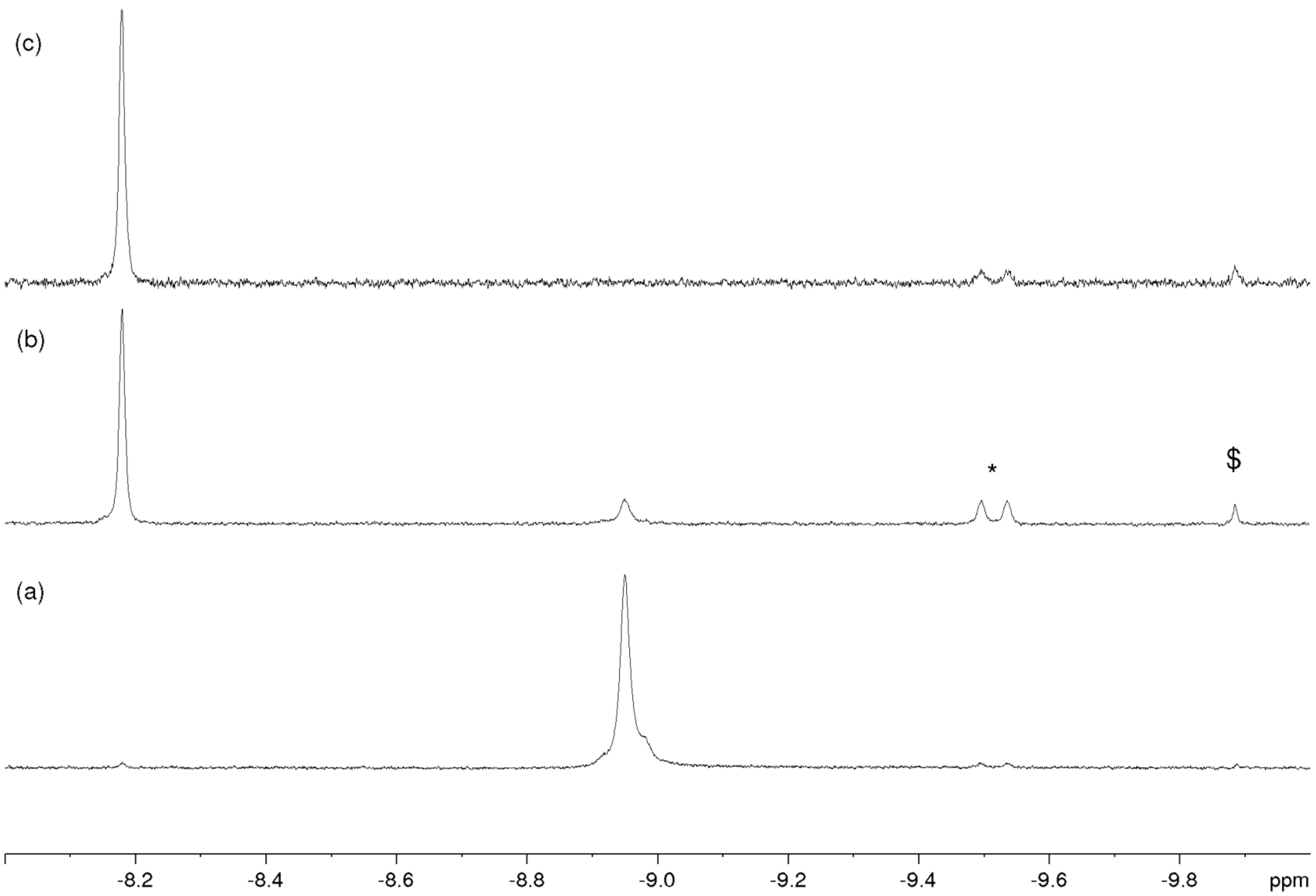

Figure S97. Partial hydride region of the ^{1}H NMR spectrum (THF-d_8, 400 MHz, 298 K) of (a) [Ir(IPr)$_2$(ZnEt)(η^2-H_2)H_3][BAr$^F{}_4$] (**11c**) and then (b) 5 min and (c) 20 min after addition of styrene (5 equiv) to generate [Ir(IPr)(IPr'')(ZnEt)H][BAr$^F{}_4$] (**4c**). * = [Ir(IPr)(IPr'')(ZnEt)H_3][BAr$^F{}_4$] (**9c**), $ = Ir(IPr)$_2H_5$ (**13**).

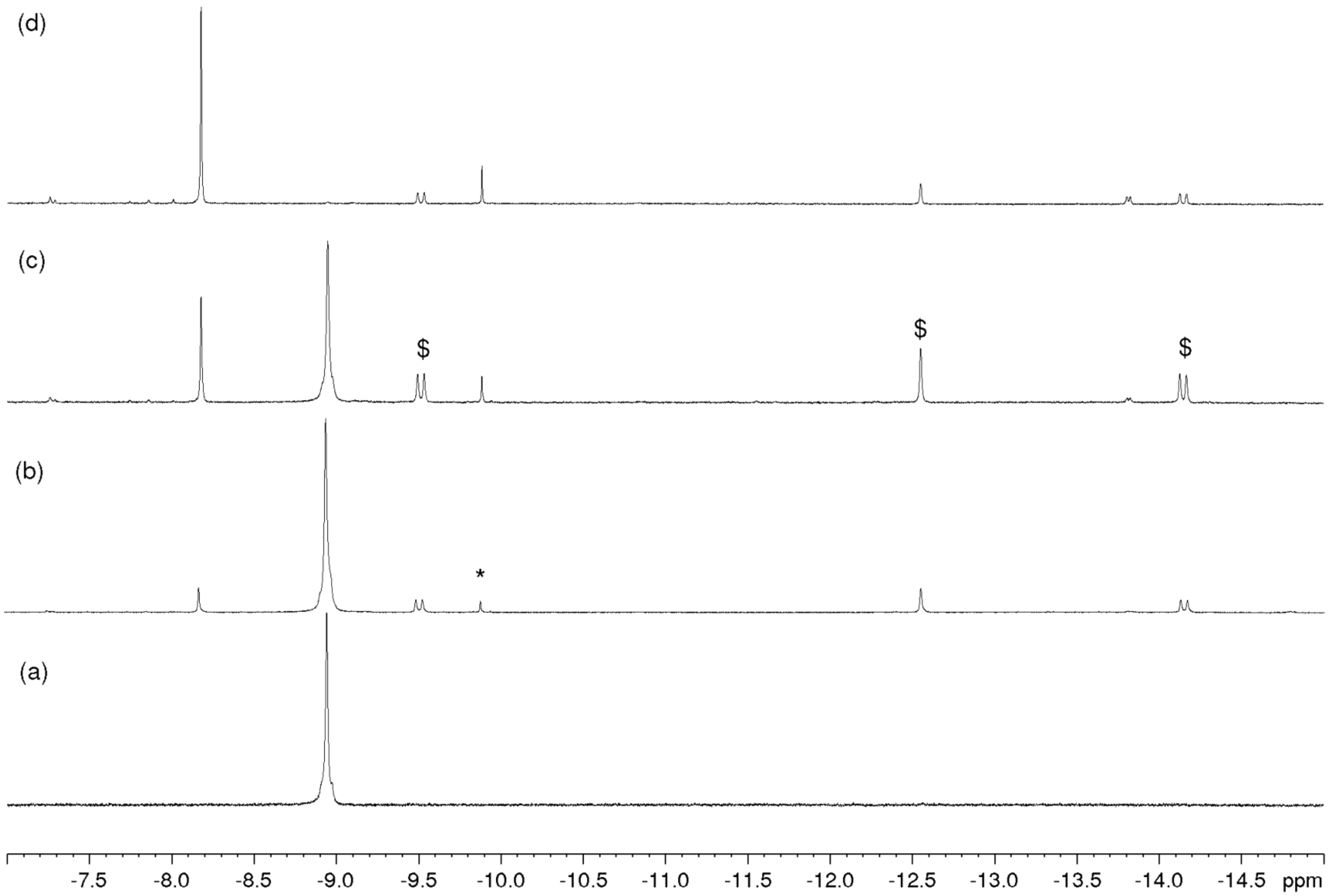

Figure S98. Hydride region of the ^{1}H NMR spectrum (THF-d_8, 400 MHz, 298 K) showing conversion of (a) [Ir(IPr)$_2$(ZnEt)(η^2-H$_2$)H$_3$][BAr$^F{}_4$] (**11c**) to [Ir(IPr)(IPr'')(ZnEt)H][BAr$^F{}_4$] (**4c**) upon heating (60 °C) under vacuum for (b) 1 h, (c) 3 h and (d) 7 h. $ = [Ir(IPr)(IPr'')(ZnEt)H$_3$][BAr$^F{}_4$] (**9c**), * = Ir(IPr)$_2$H$_5$ (**13**).

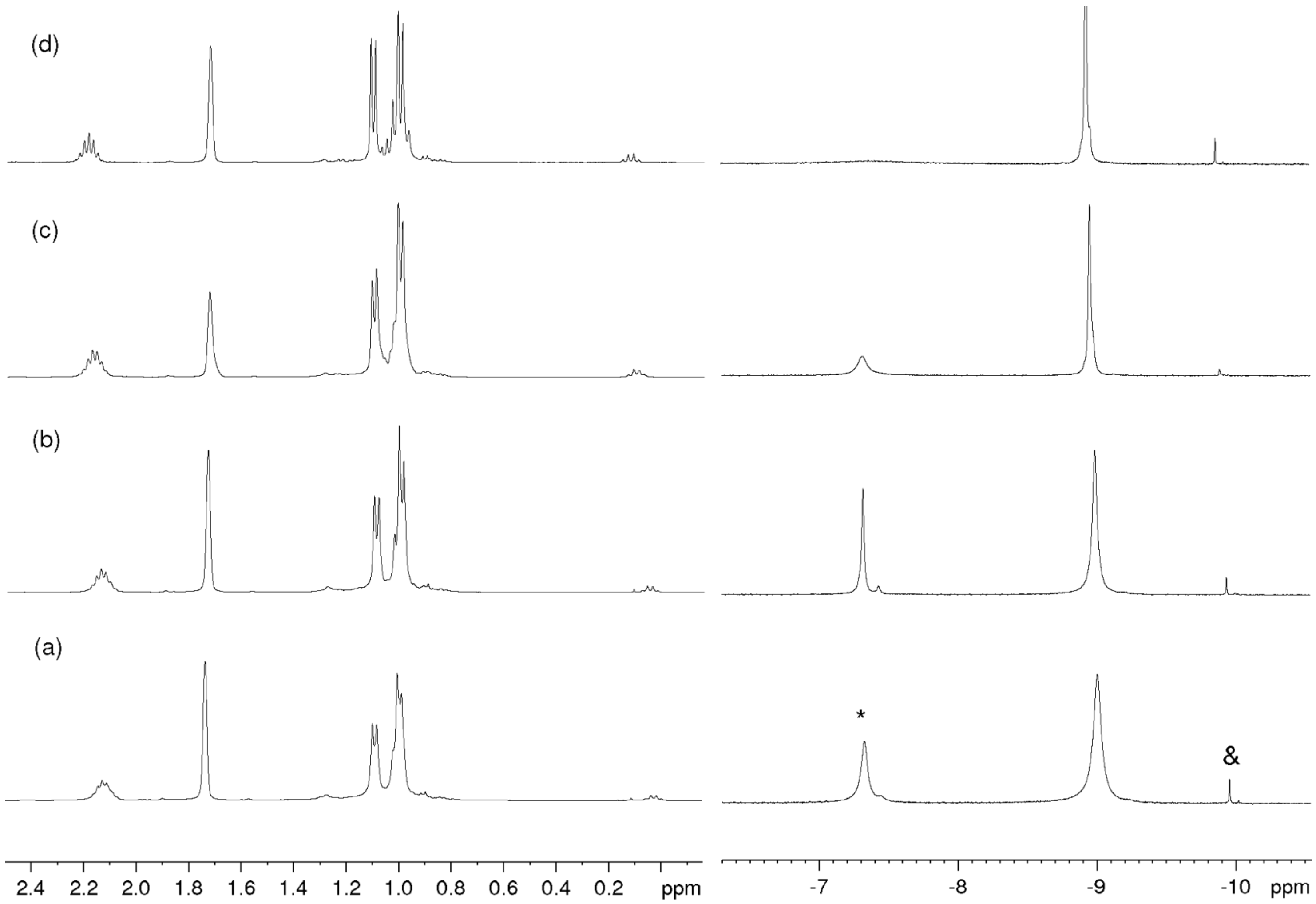

Figure S99. Alkyl (left) and hydride (right) regions of the ^1H NMR spectrum (THF-d_8, 400 MHz) of $[Ir(IPr)_2(ZnEt)(\eta^2\text{-}H_2)H_3][BAr^F_4]$ (**11c**) formed in-situ from [Ir(IPr)(IPr'')(ZnEt)H]$[BAr^F_4]$ (**4c**) and H_2 recorded at (a) 228 K, (b) 248 K, (c) 298 K and (d) 323 K (* = $[Ir(IPr)_2(\eta^2\text{-}H_2)_2H_2][BAr^F_4]$ (**6**), & = $Ir(IPr)_2H_5$ (**13**)).

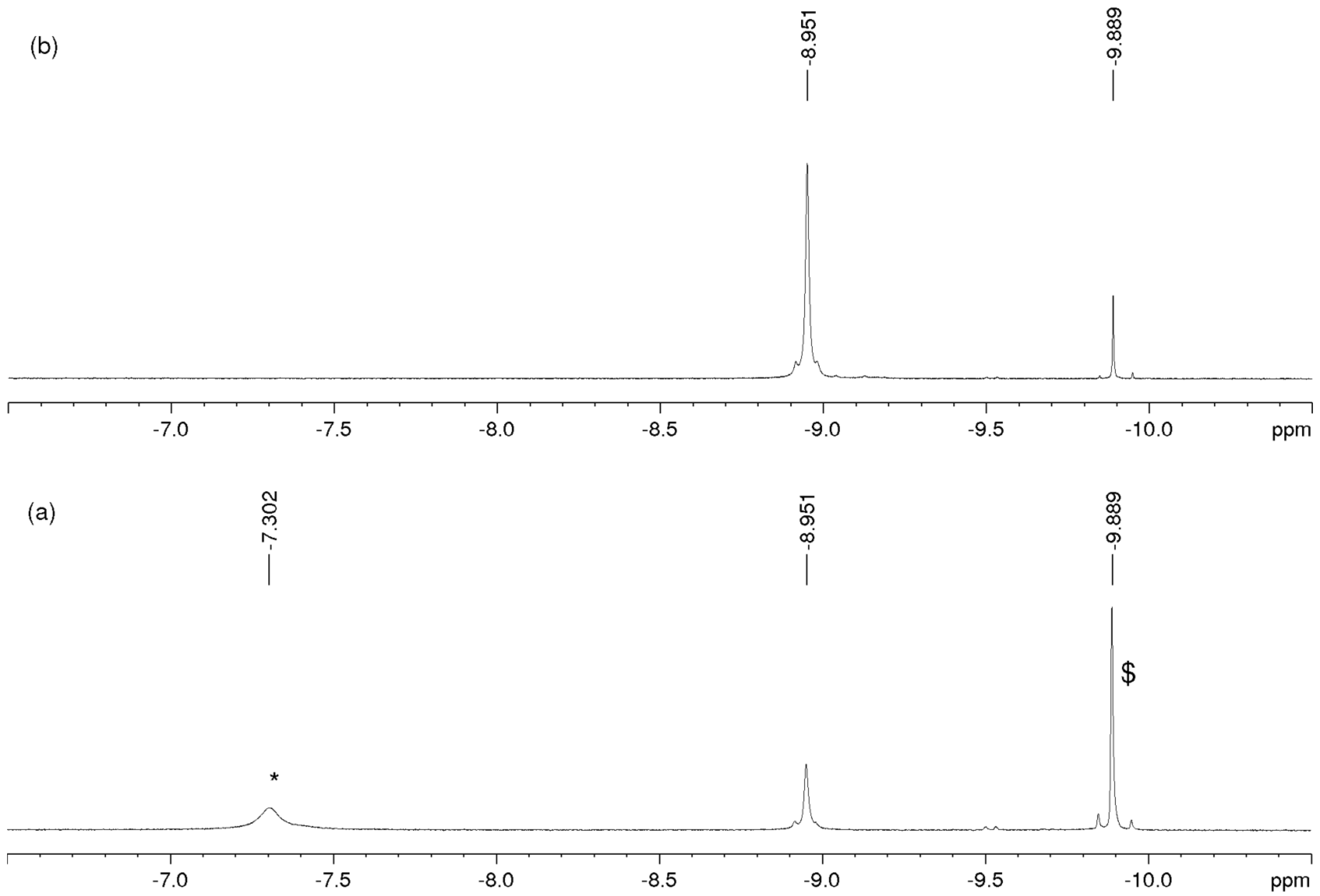

Figure S100. Hydride region of the ^1H NMR spectra (500 MHz, 298 K) of (a) a reaction of [Ir(IPr)(IPr'')(ZnEt)H][BAr$^F_4$] (**4c**) and H_2 in degassed, undried THF-d_8 and (b) a sample of [Ir(IPr$_2$)(ZnEt)(η^2-H_2)H_3][BAr$^F_4$] (**11c**) dissolved in degassed, undried THF-d_8 indicating the formation of [Ir(IPr)$_2$(η^2-H_2)$_2H_2$][BAr$^F_4$] (**6**, *) and Ir(IPr)$_2H_5$ (**13,** $).

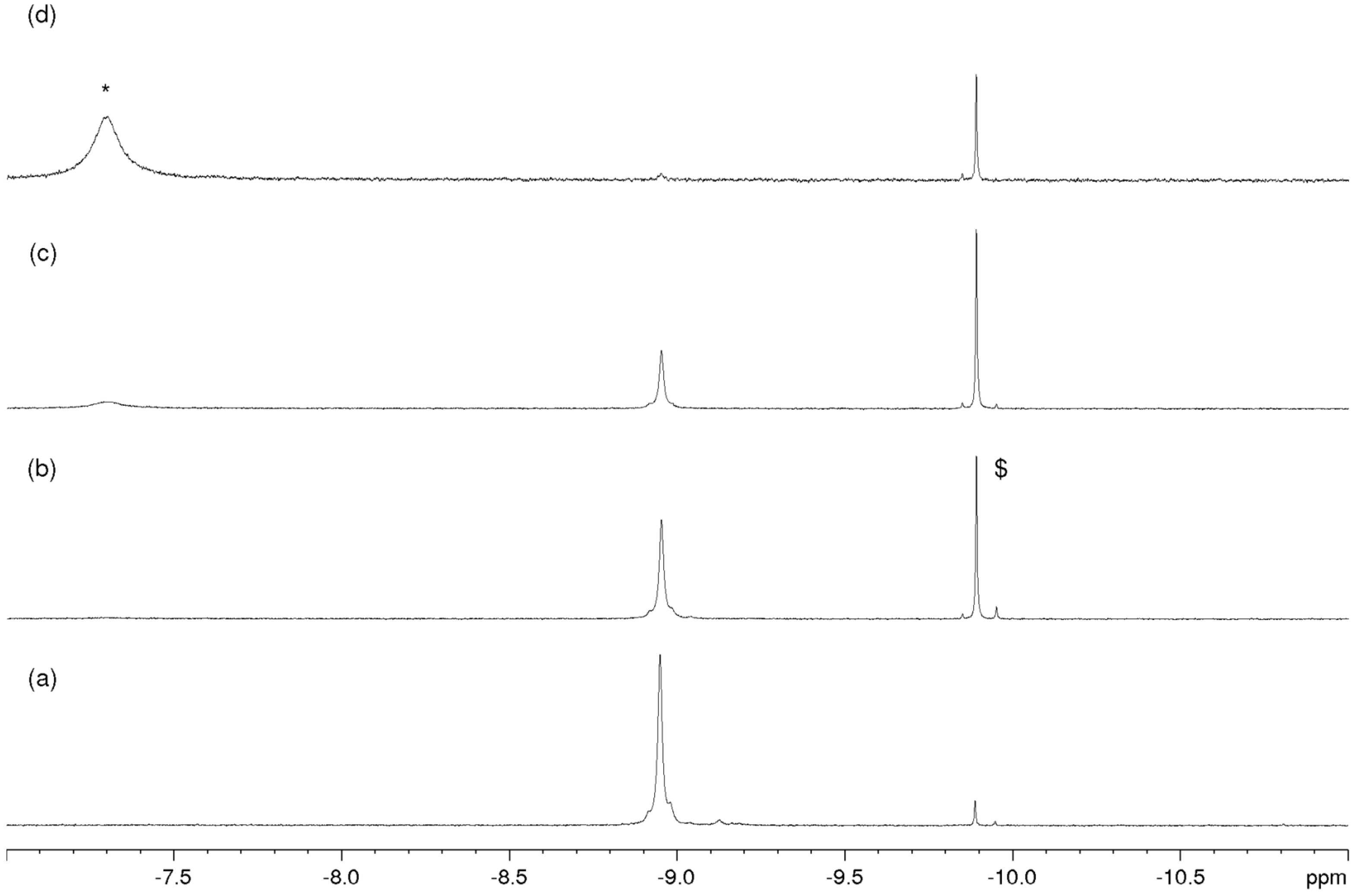

Figure S101. Hydride region of the ^{1}H NMR spectrum (400 MHz, 298 K) of crystalline $[Ir(IPr)_2(ZnEt)(\eta^2\text{-}H_2)H_3][BAr^F{}_4]$ (**11c**) redissolved in degassed/undried THF-d_8 after (a) 5 min, (b) 2 h, (c) 5 h and (d) 12 h ($ = $Ir(IPr)_2H_5$ (**13**), * = $[Ir(IPr)_2(\eta^2\text{-}H_2)_2H_2][BAr^F{}_4]$ (**6**)).

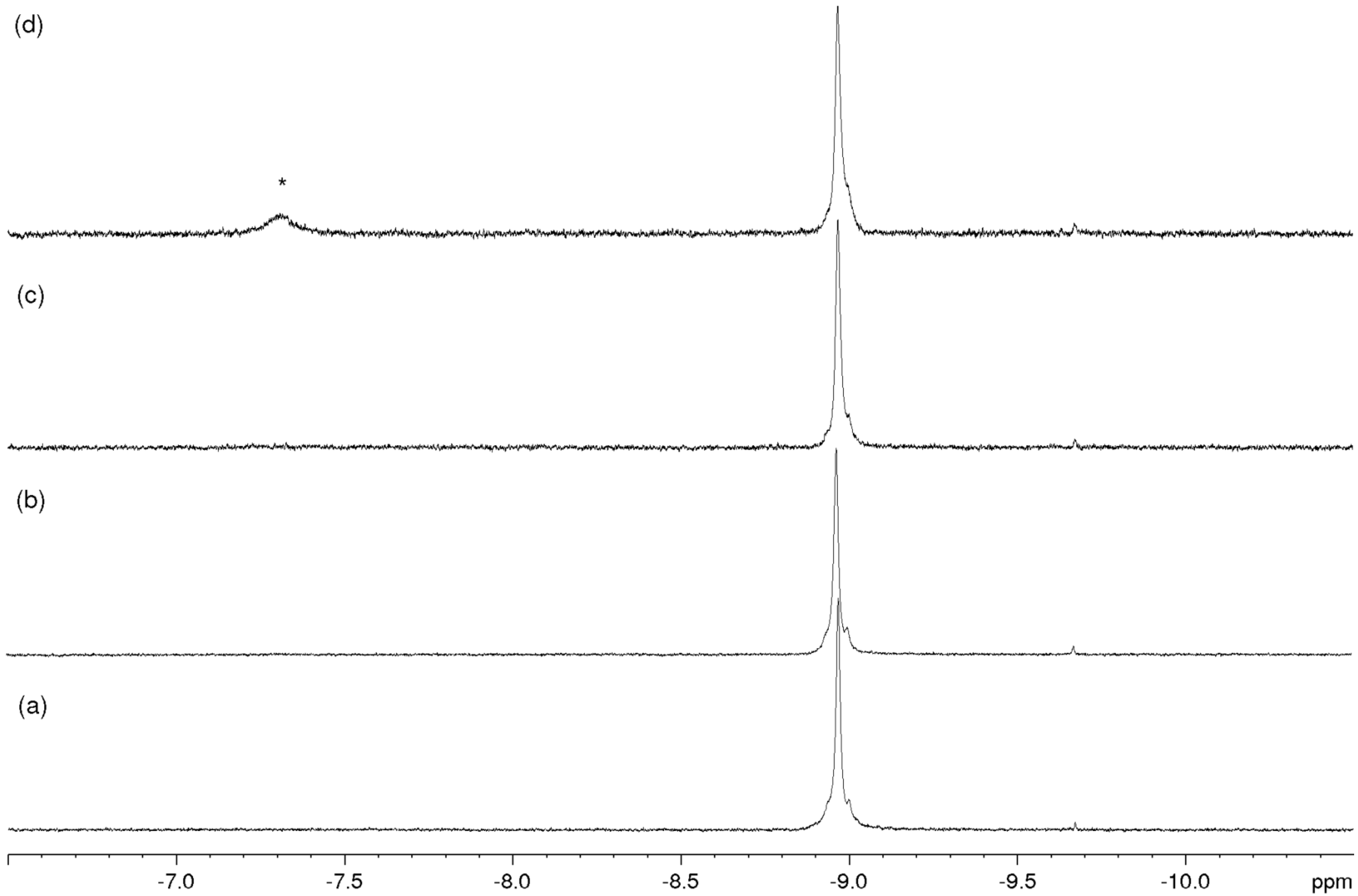

Figure S102. Hydride region of the ^{1}H NMR spectrum (400 MHz, 298 K) of crystalline $[Ir(IPr)_2(ZnEt)(\eta^2\text{-}H_2)H_3][BAr^F{}_4]$ (**11c**) redissolved in degassed/undried C_6H_5F after (a) 5 min, (b) 2 h, (c) 5 h and (d) 12 h (* = $[Ir(IPr)_2(\eta^2\text{-}H_2)_2H_2][BAr^F{}_4]$ (**6**)).

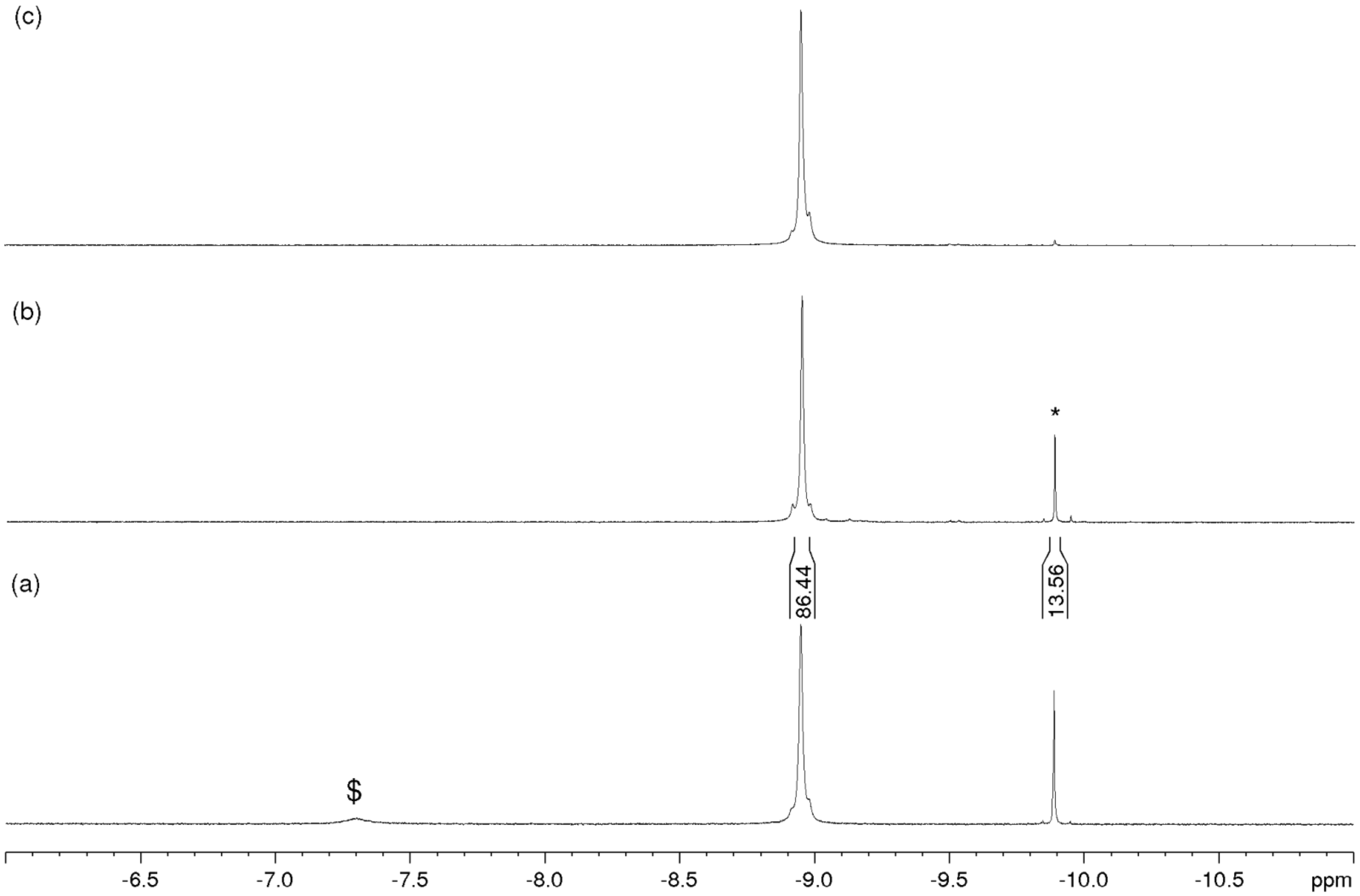

Figure S103. (a) Hydride region of the ^{1}H NMR spectrum (500 MHz, 298 K, THF-d_8) of $[Ir(IPr)_2(ZnEt)(\eta^2\text{-}H_2)H_3][BAr^F{}_4]$ (**11c**) formed in a reaction of $[Ir(IPr)(IPr'')(ZnEt)H][BAr^F{}_4]$ (**4c**) and H_2 in K-dried solvent. Spectrum (b) shows the effect of dissolving an isolated sample of **11c** in degassed, but undried THF-d_8 (integrals shown), while (c) shows the same experiment using Na/K-dried THF-d_8. \$ = $[Ir(IPr)_2(\eta^2\text{-}H_2)_2H_2][BAr^F{}_4]$ (**6**), \$ = $Ir(IPr)_2H_5$ (**13**).

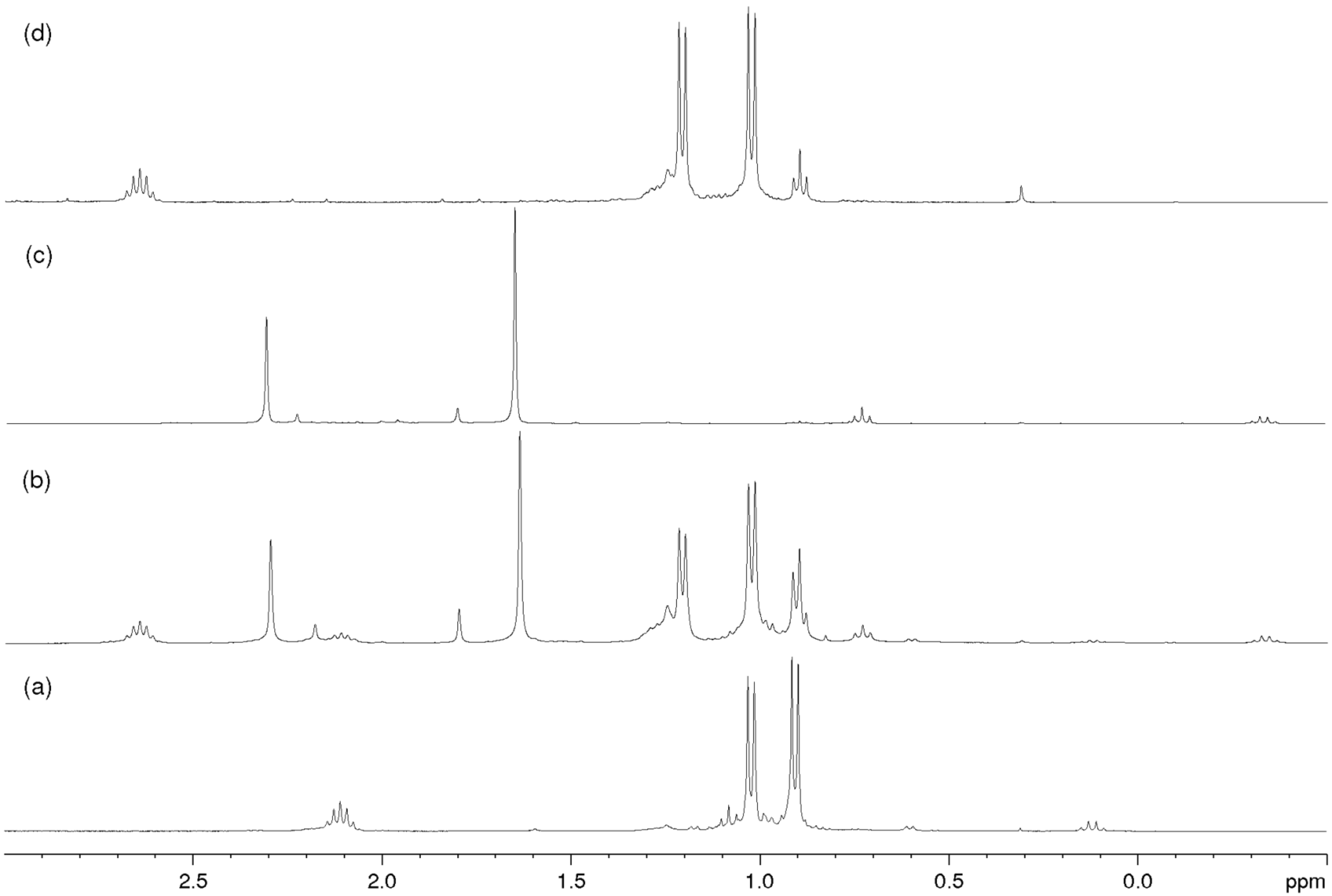

Figure S104. Alkyl region of the ^{1}H NMR spectrum (400 MHz, 298 K, C_6H_5F) of (a) $[Ir(IPr)_2(ZnEt)(\eta^2\text{-}H_2)H_3][BAr^F{}_4]$ (**11c**) and (b) 5 min after addition of IMes (2 equiv) to give $Ir(IPr)_2H_5$ (**13**) and $[(IMes)_2ZnEt][BAr^F{}_4]$. For comparison, spectra of authentic samples of $[(IMes)_2ZnEt][B(C_6F_5)_4]$ and **13** are shown in (c) and (d) respectively.

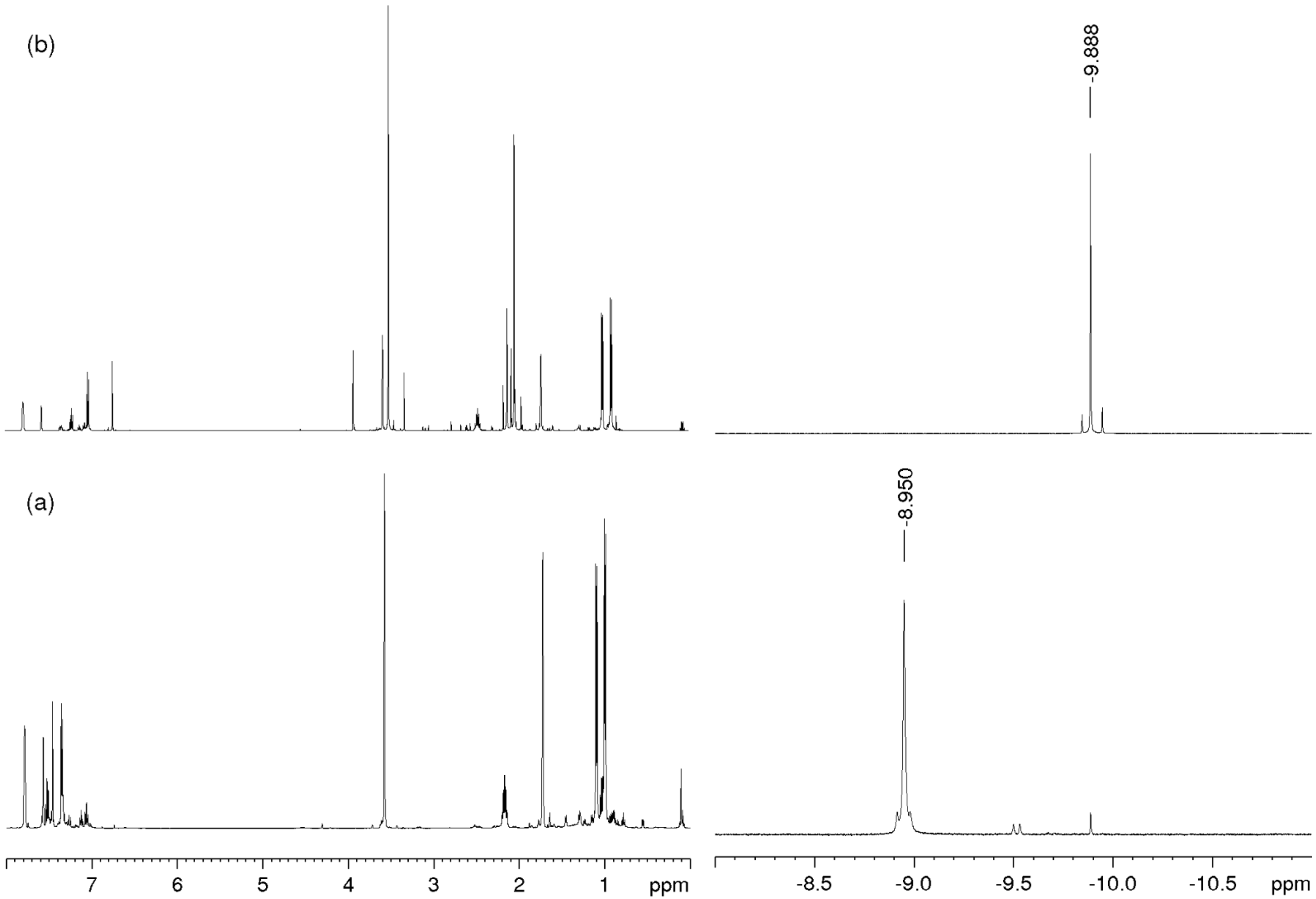

Figure S105. ^{1}H NMR spectrum (500 MHz, THF-d_8, 298 K) of (a) an in-situ prepared sample of [Ir(IPr)$_2$(ZnEt)(η^2-H$_2$)H$_3$][BAr$^F{}_4$] (**11c**) (note traces of residual [Ir(IPr)(IPr'')(ZnEt)H$_3$][BAr$^F{}_4$] (**9c**) at δ −9.5 and −9.7) and (b) 1 h after reaction with 2 equiv IMe$_4$ to give Ir(IPr)$_2$H$_5$ (**13**).

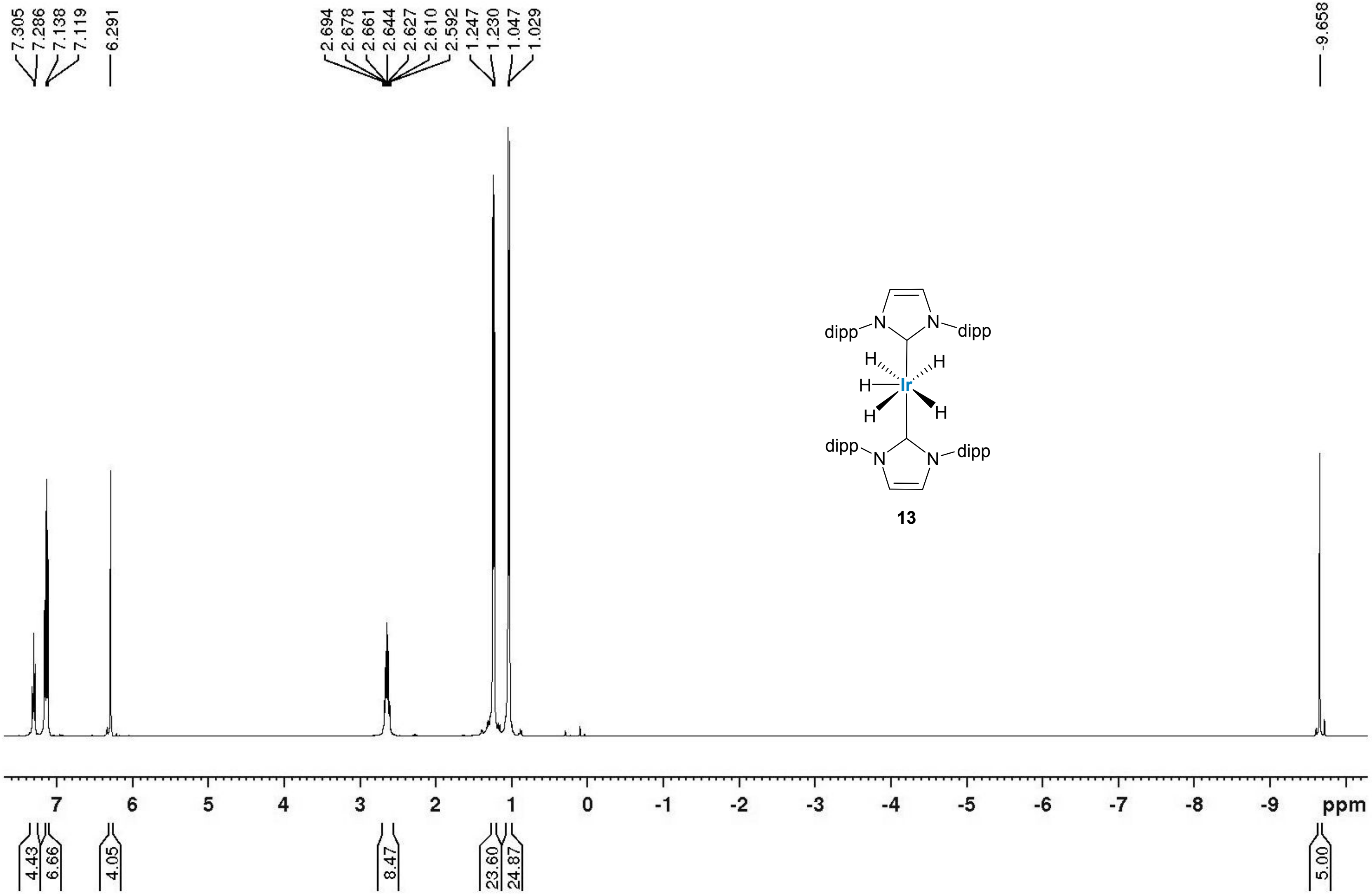

Figure S106. ^{1}H NMR spectrum (C_6D_6, 400 MHz, 298 K) of $Ir(IPr)_2H_5$ (**13**).

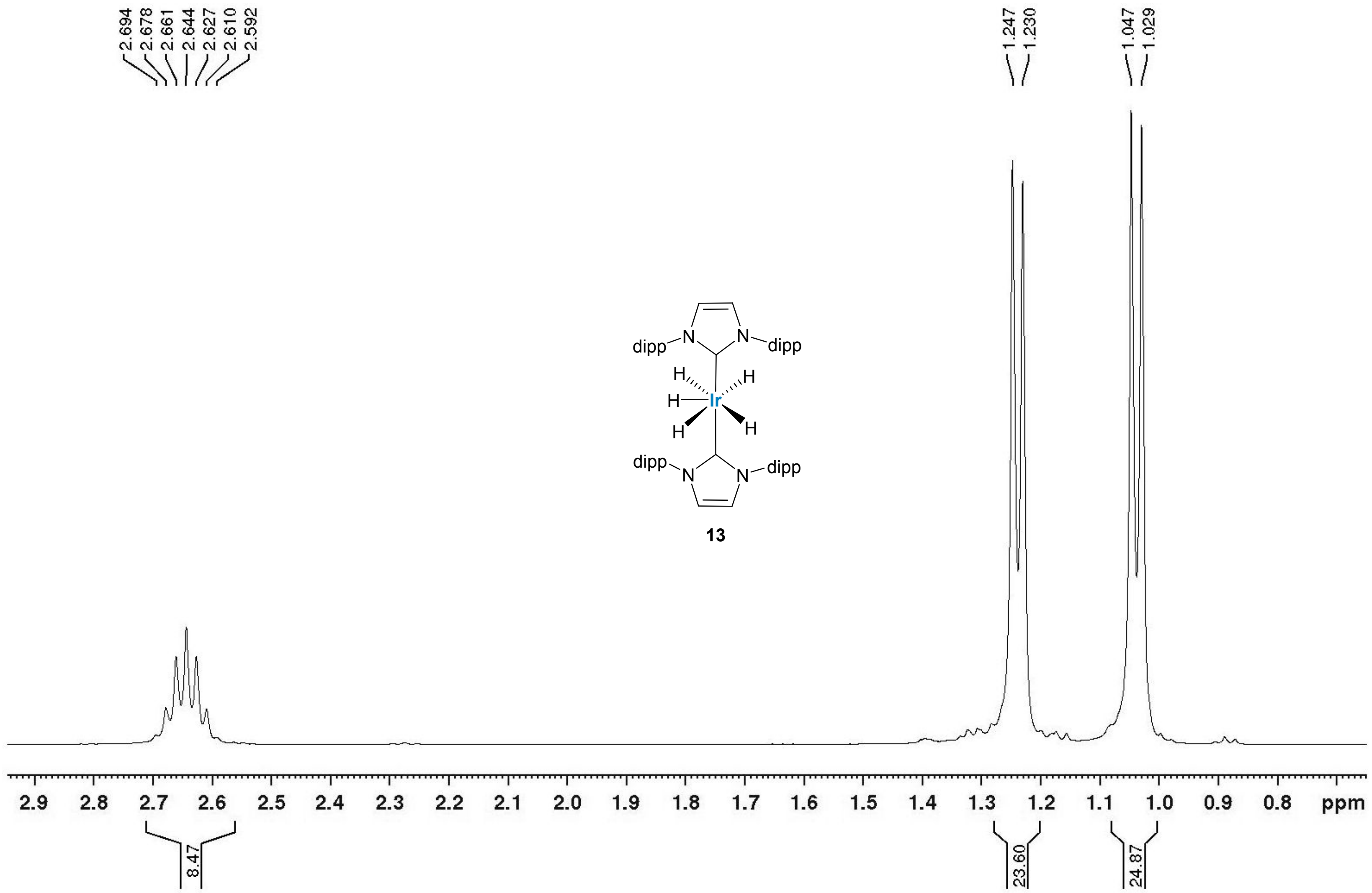

Figure S107. Alkyl region of the ^{1}H NMR spectrum (C_6D_6, 400 MHz, 298 K) of $Ir(IPr)_2H_5$ (**13**).

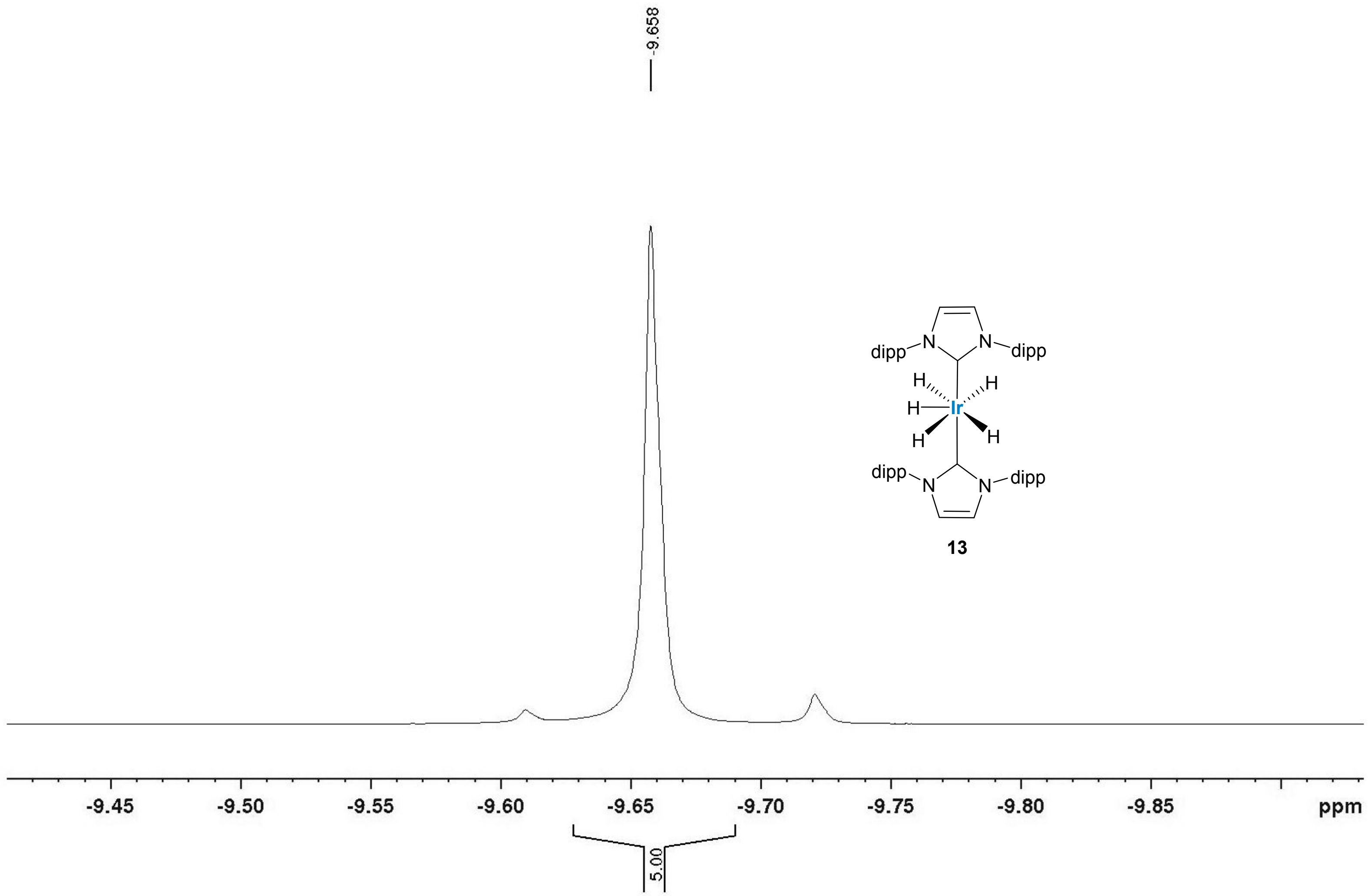

Figure S108. Hydride region of the ^{1}H NMR spectrum (C_6D_6, 400 MHz, 298 K) of $Ir(IPr)_2H_5$ (**13**).

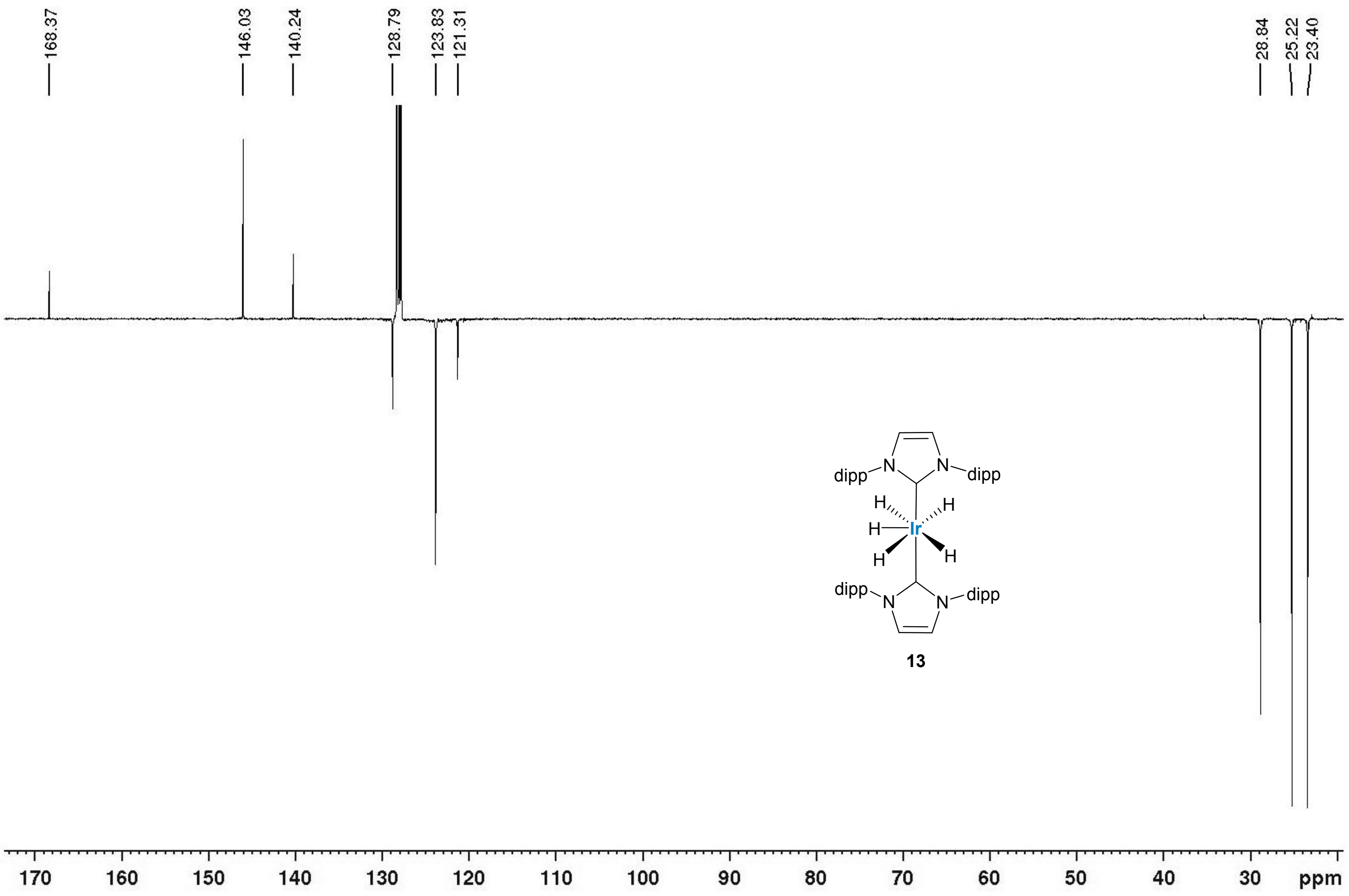

Figure S109. $^{13}C\{^{1}H\}$ DEPTQ NMR spectrum (C_6D_6, 126 MHz, 298 K) of $Ir(IPr)_2H_5$ (**13**).

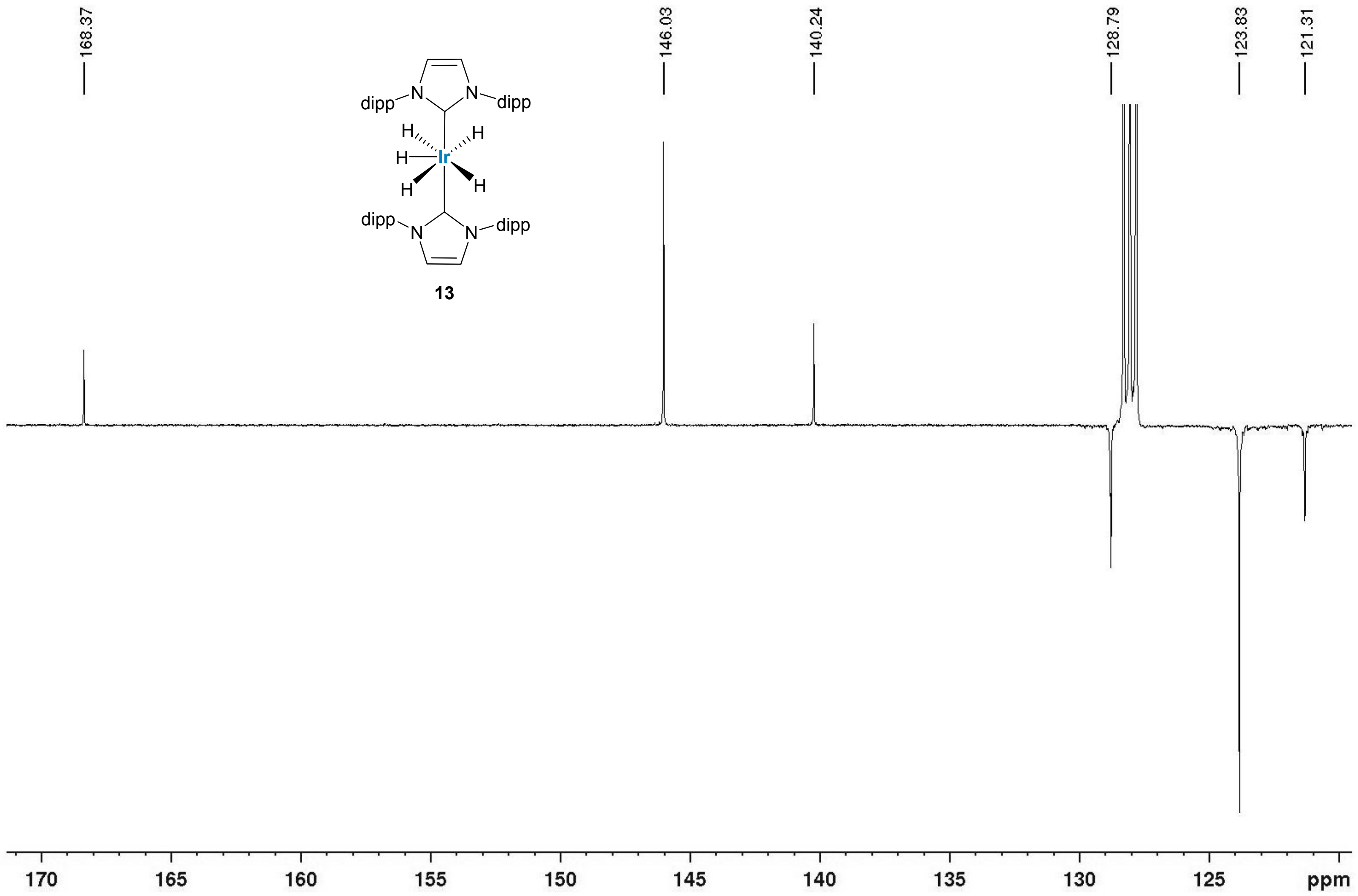

Figure S110. Aromatic region of the $^{13}C\{^{1}H\}$ DEPTQ NMR spectrum (C_6D_6, 126 MHz, 298 K) of $Ir(IPr)_2H_5$ (**13**).

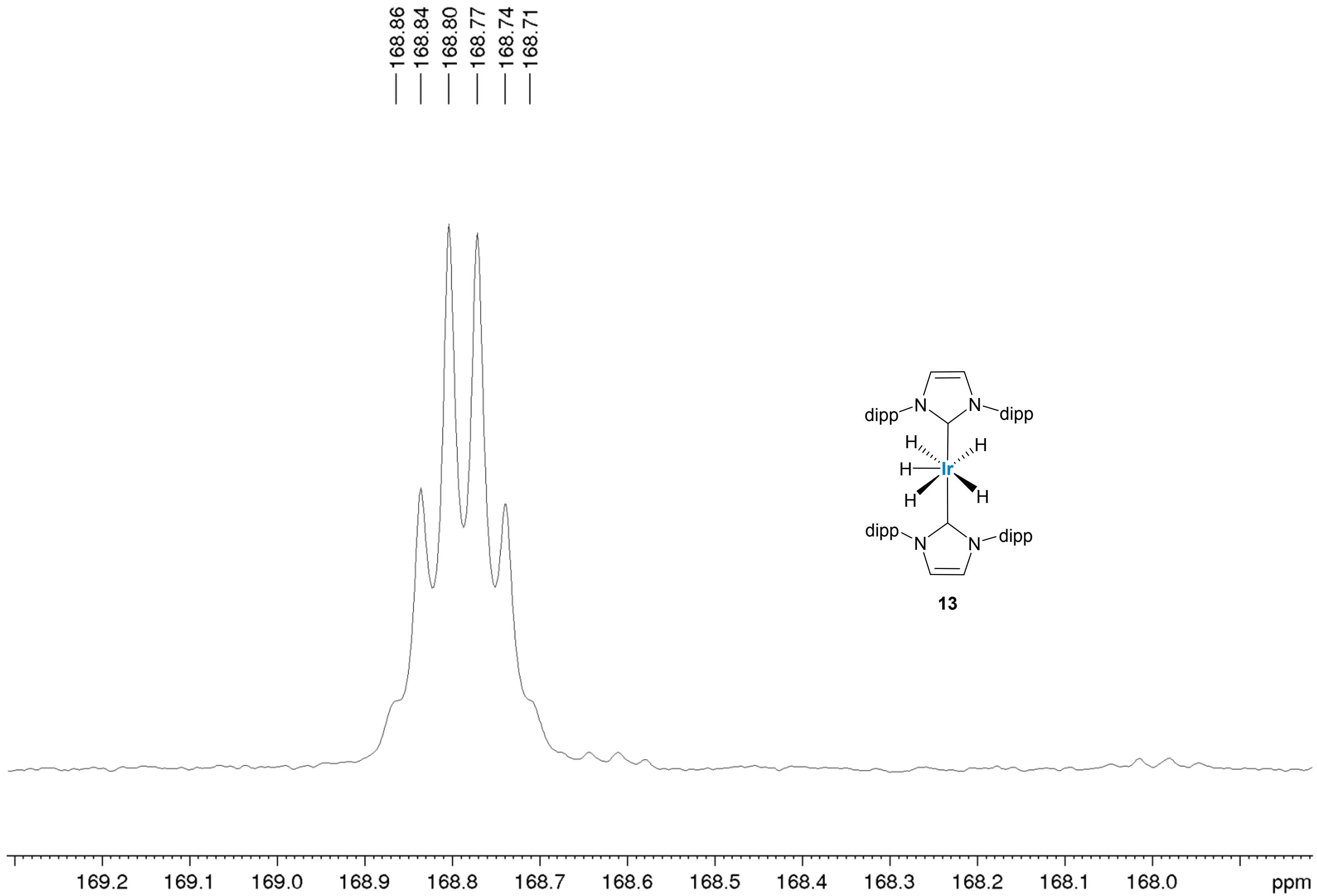

Figure S111. Sextet hydride splitting of the IrC_{IPr} resonance in the $^{13}C\{selective\text{-}^{1}H\}^{*}$ NMR spectrum ($C_6D_5CD_3$, 126 MHz, 278 K) of $Ir(IPr)_2H_5$ (**13**) (* ^{1}H decoupler set to decouple aromatic protons only (δ 6-9)).

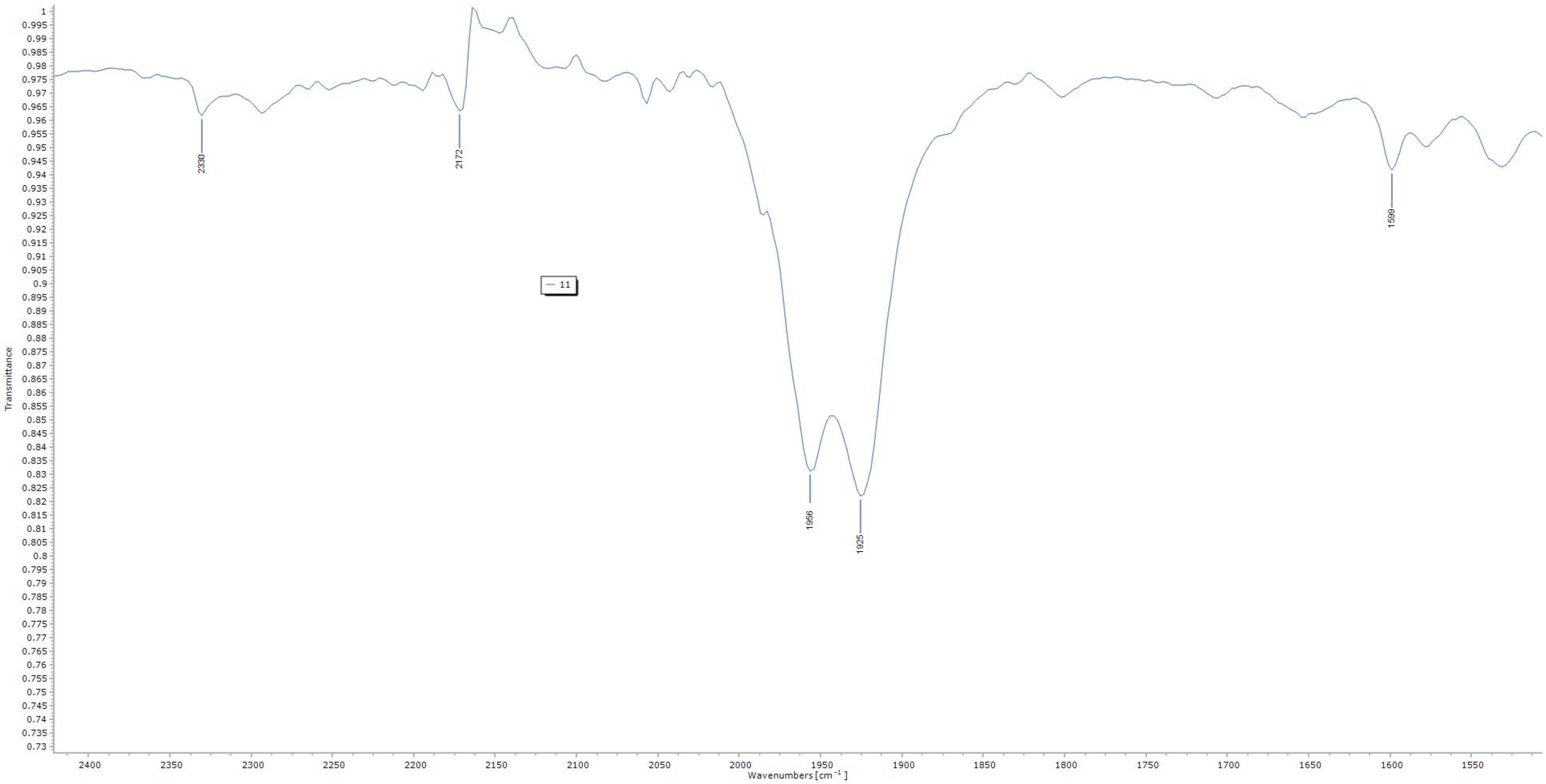

Figure S112. ATR-IR spectrum of $Ir(IPr)_2H_5$ (**13**)

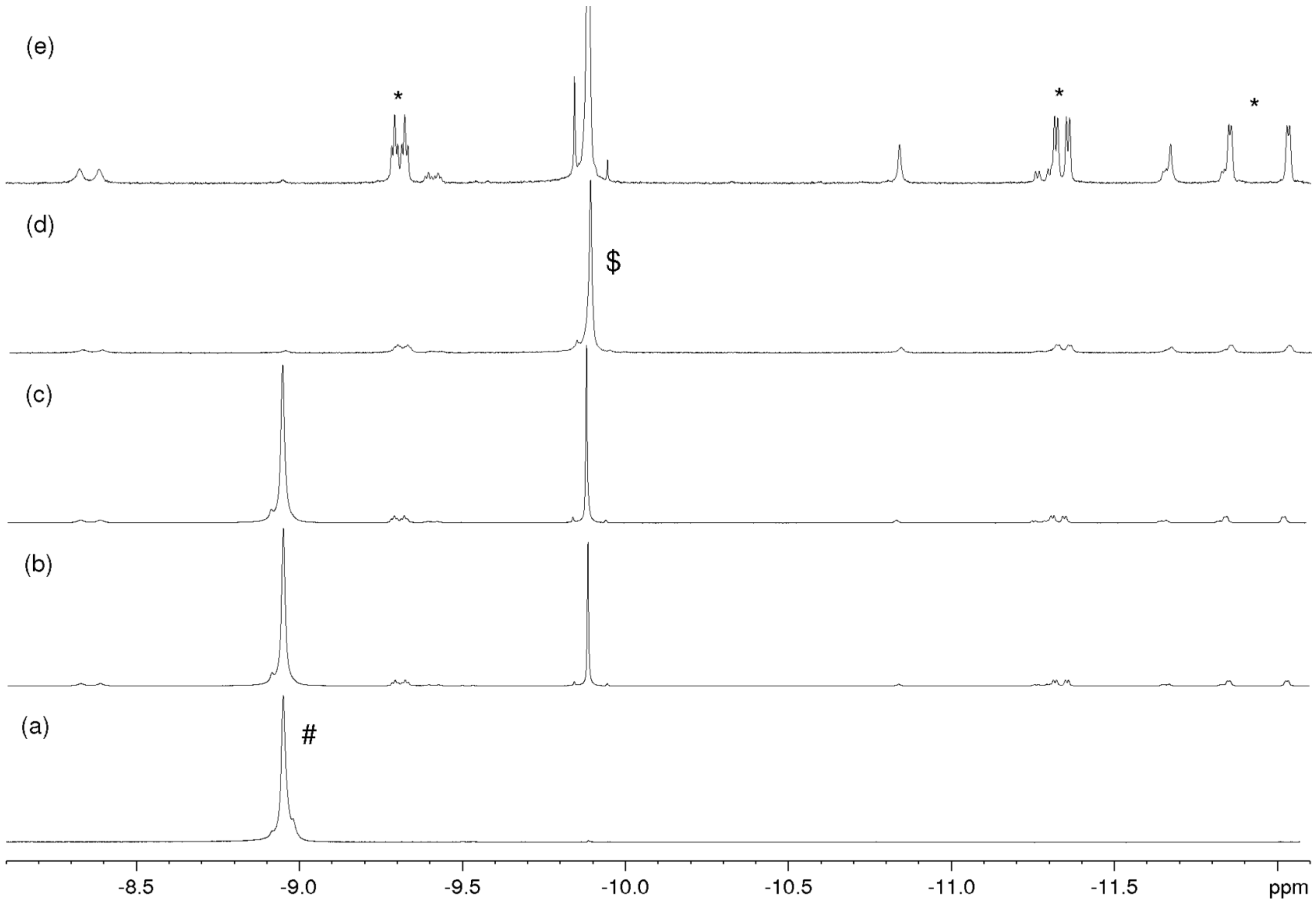

Figure S113. Hydride region of the ^1H NMR spectrum (500 MHz, THF-d_8, 298 K) of (a) $[Ir(IPr)_2(ZnEt)(\eta^2\text{-}H_2)H_3][BAr^F{}_4]$ (**11c**, #) recorded (b) 10 min, (c) 20 min and (d) 12 h after addition of 2 equiv PMe_3 to give $Ir(IPr)_2H_5$ (**13**, $). The spectrum in (e) is of the residue after removal of the volatiles and is expanded vertically to emphasize the formation of $[Ir(IPr)_2(ZnEt)(PMe_3)H_3][BAr^F{}_4]$ (**14**, *).

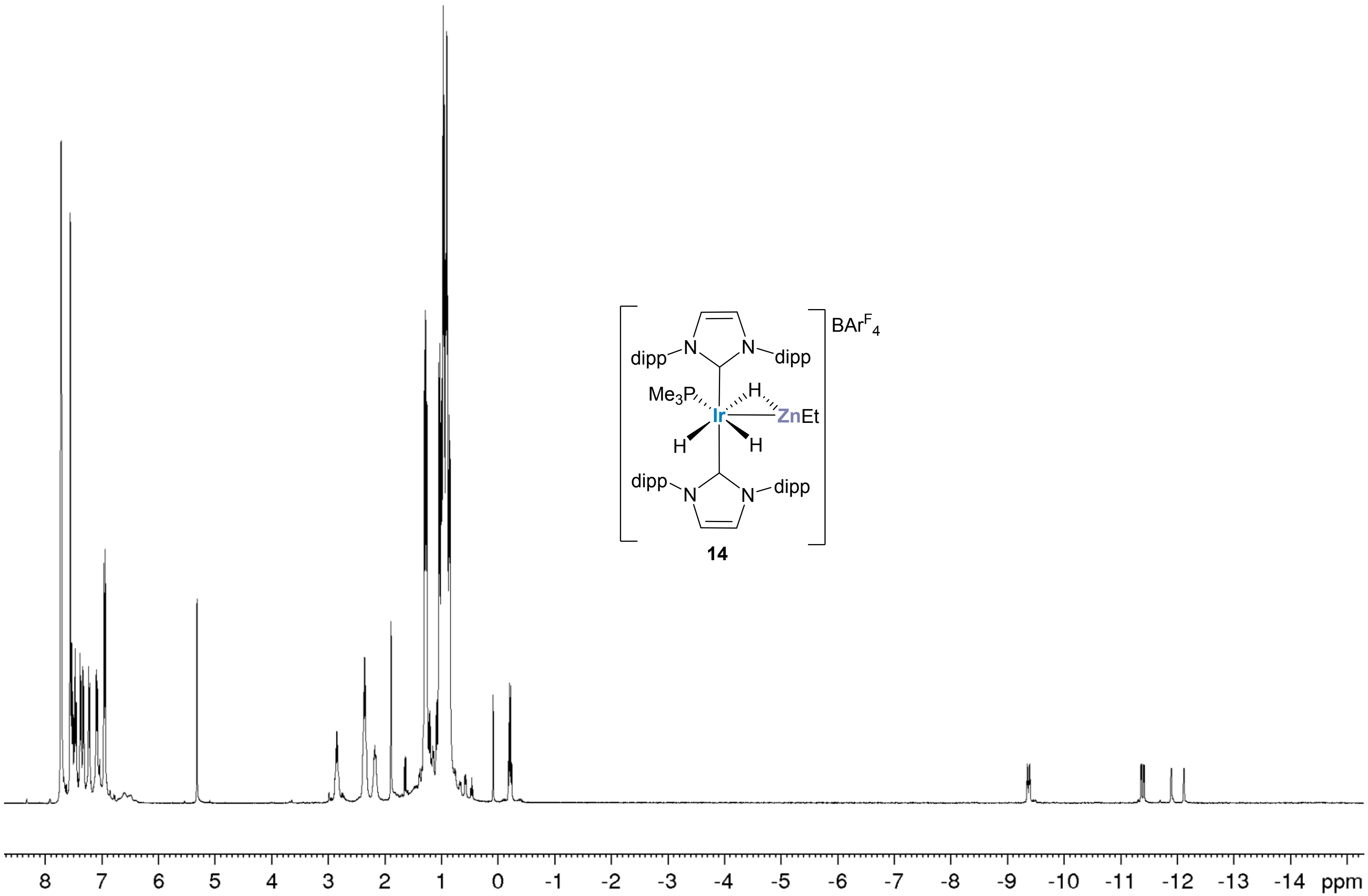

Figure S114. ^{1}H NMR spectrum (CD_2Cl_2, 400 MHz, 298 K) of $[Ir(IPr)_2(ZnEt)(PMe_3)H_3][BAr^F_4]$ (**14**).

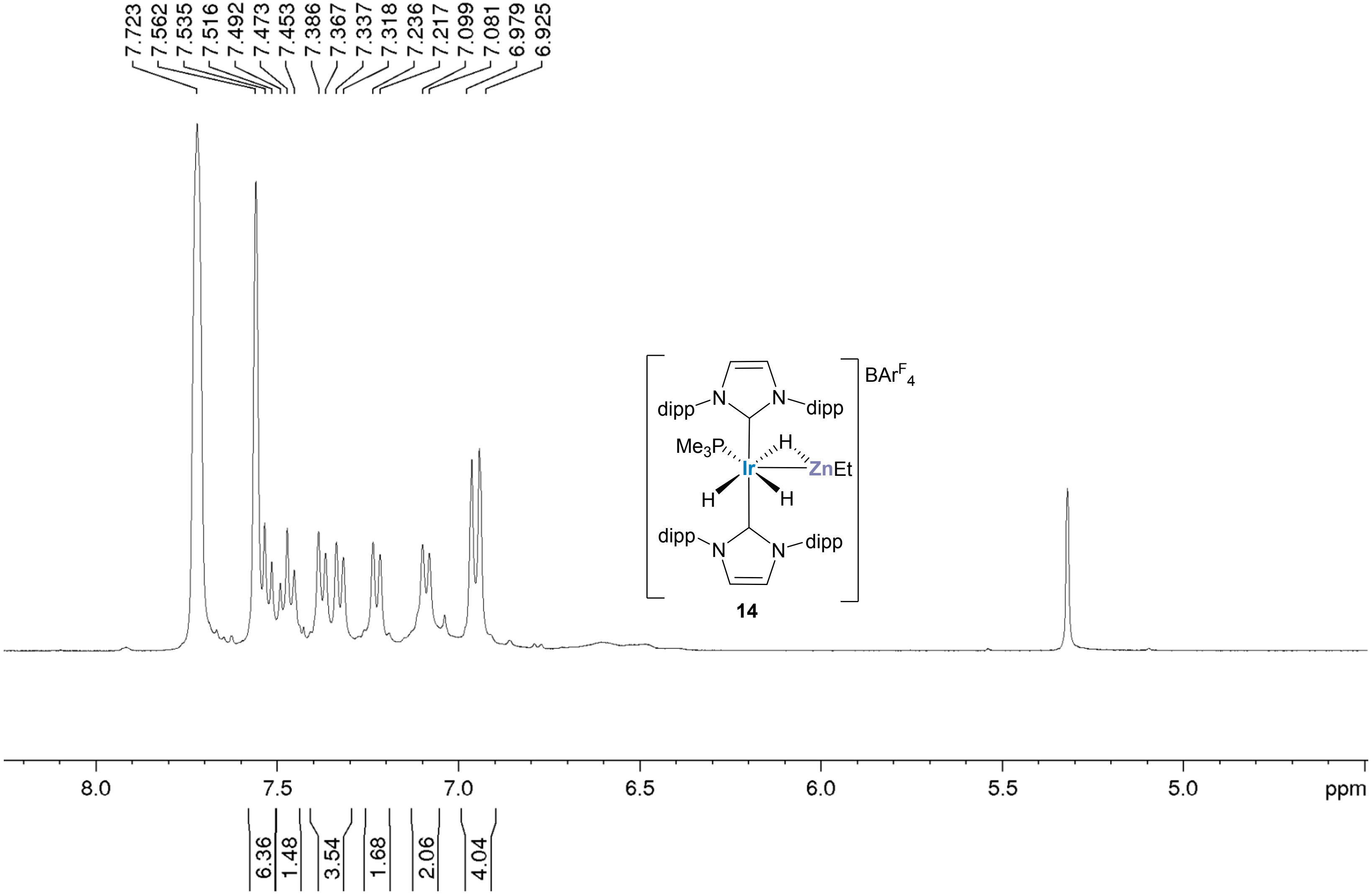

Figure S115. Aromatic region of ^{1}H NMR spectrum (CD_2Cl_2, 400 MHz, 298 K) of [Ir(IPr)$_2$(ZnEt)(PMe$_3$)H$_3$][BAr$^F{}_4$] (**14**).

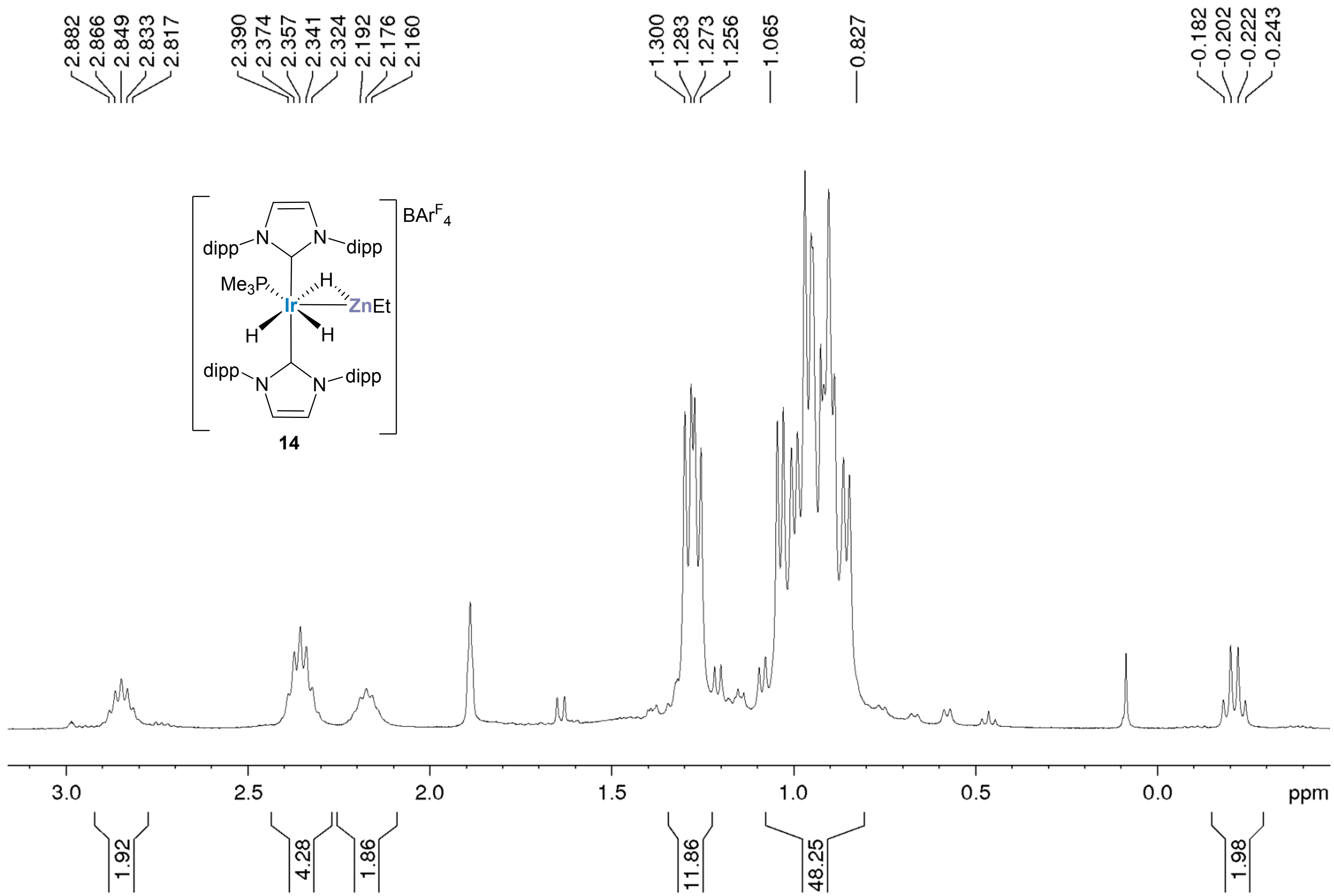

Figure S116. Alkyl region of ^{1}H NMR spectrum (CD_2Cl_2, 400 MHz, 298 K) of [Ir(IPr)$_2$(ZnEt)(PMe$_3$)H$_3$][BAr$^F{}_4$] (**14**).

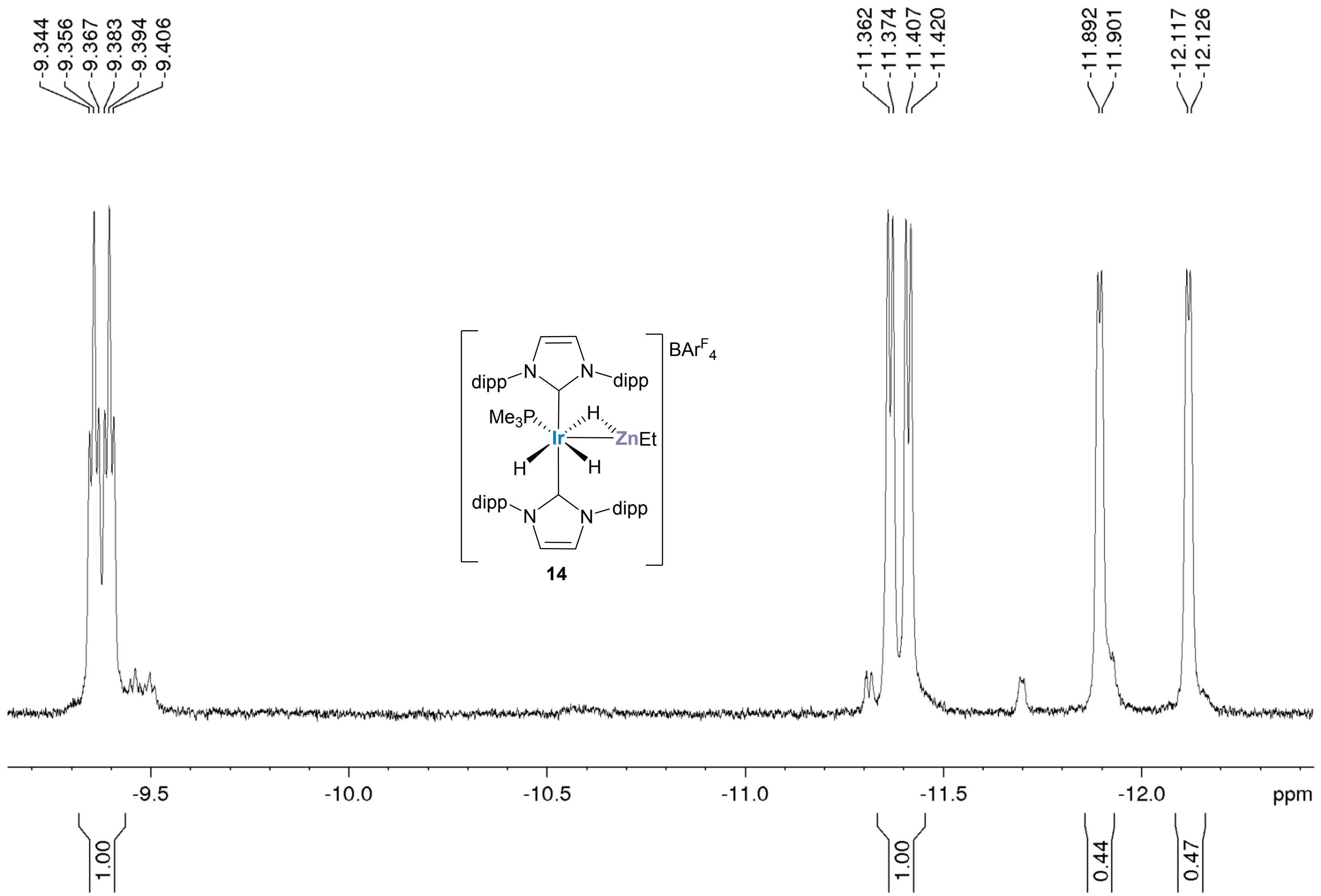

Figure S117. Hydride region of ^{1}H NMR spectrum (CD_2Cl_2, 400 MHz, 298 K) of $[Ir(IPr)_2(ZnEt)(PMe_3)H_3][BAr^F{}_4]$ (**14**).

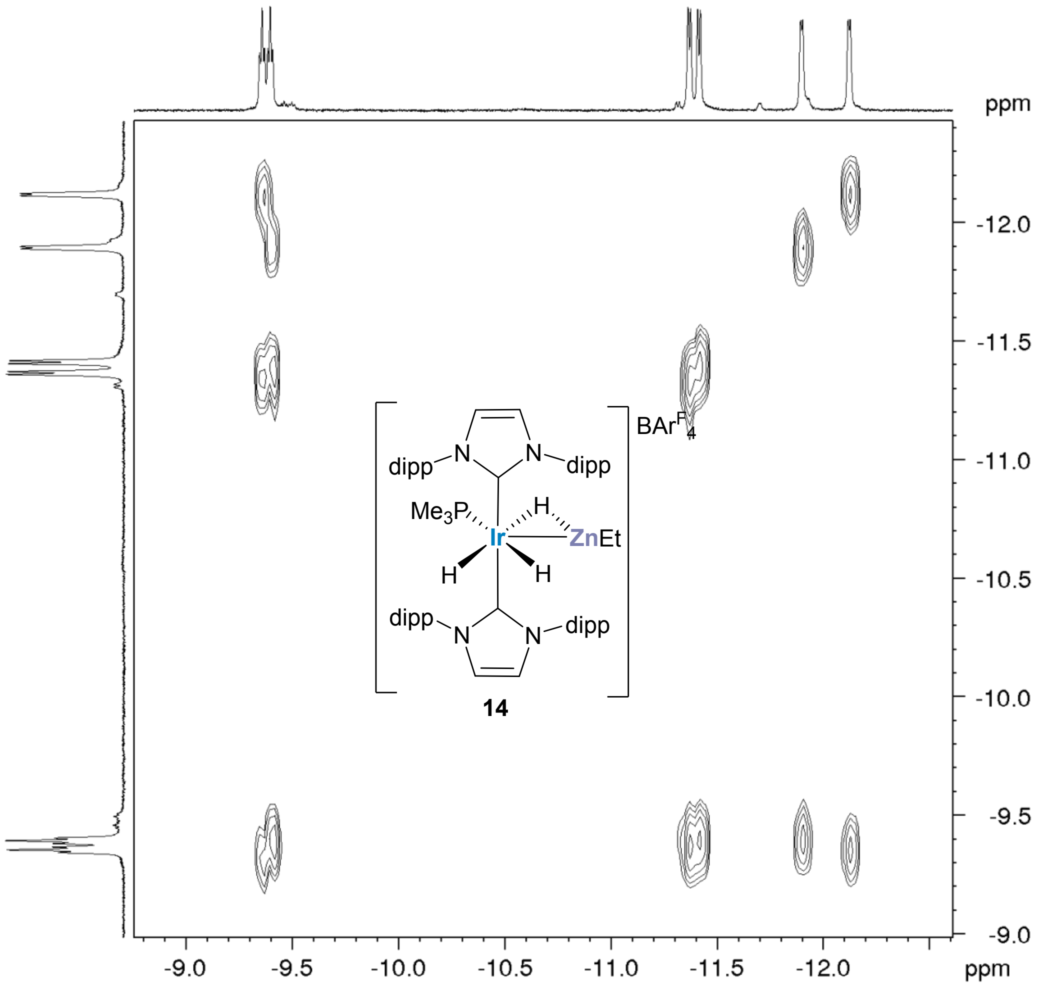

Figure S118. Hydride region of the ^{1}H COSY NMR spectrum (CD_2Cl_2, 400 MHz, 298 K) of $[Ir(IPr)_2(ZnEt)(PMe_3)H_3][BAr^F{}_4]$ (**14**).

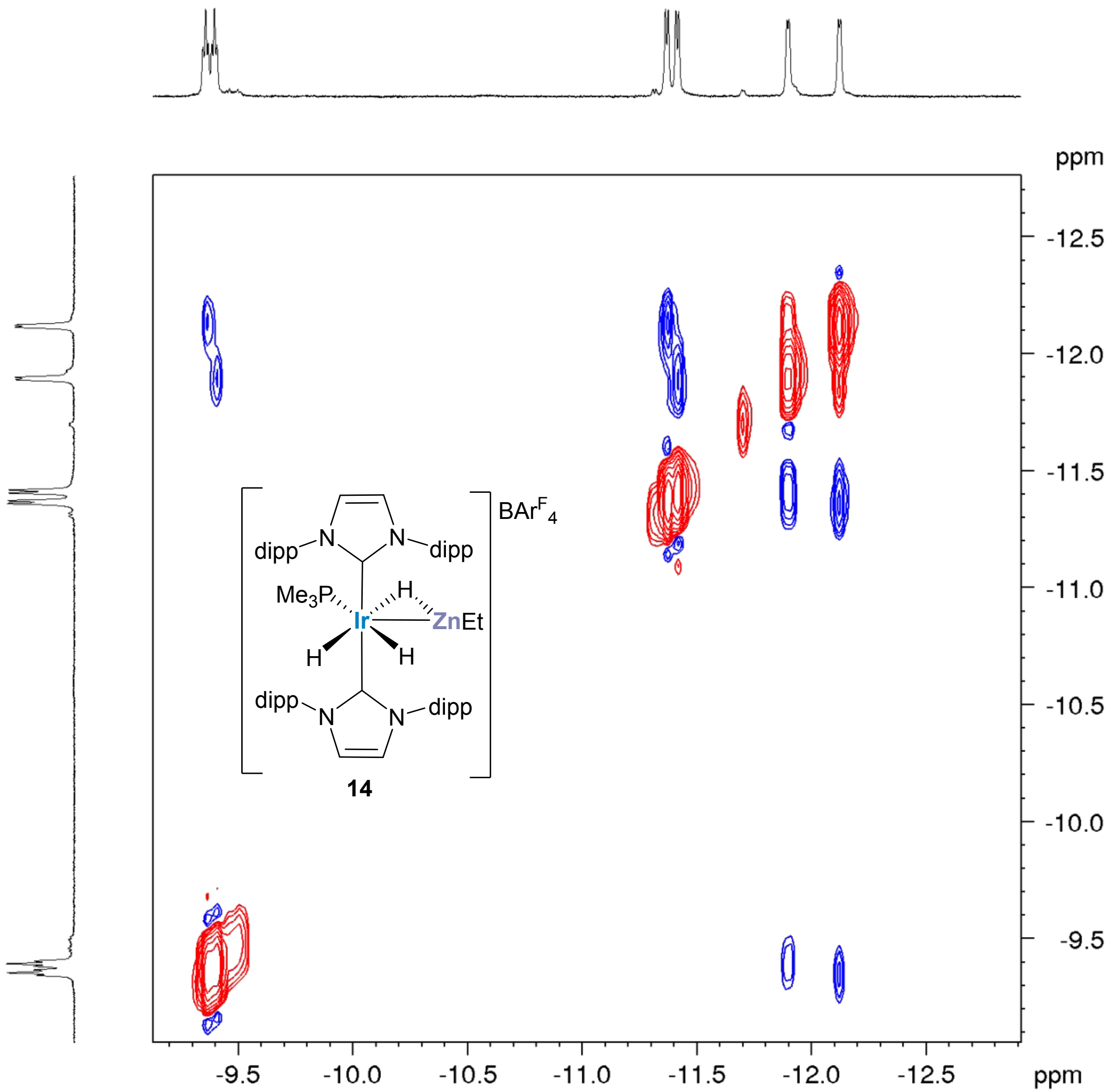

Figure S119. Hydride region of the ^{1}H NOESY NMR spectrum (CD_2Cl_2, 400 MHz, 298 K) of [Ir(IPr)$_2$(ZnEt)(PMe$_3$)H$_3$][BAr$^F{}_4$] (**14**).

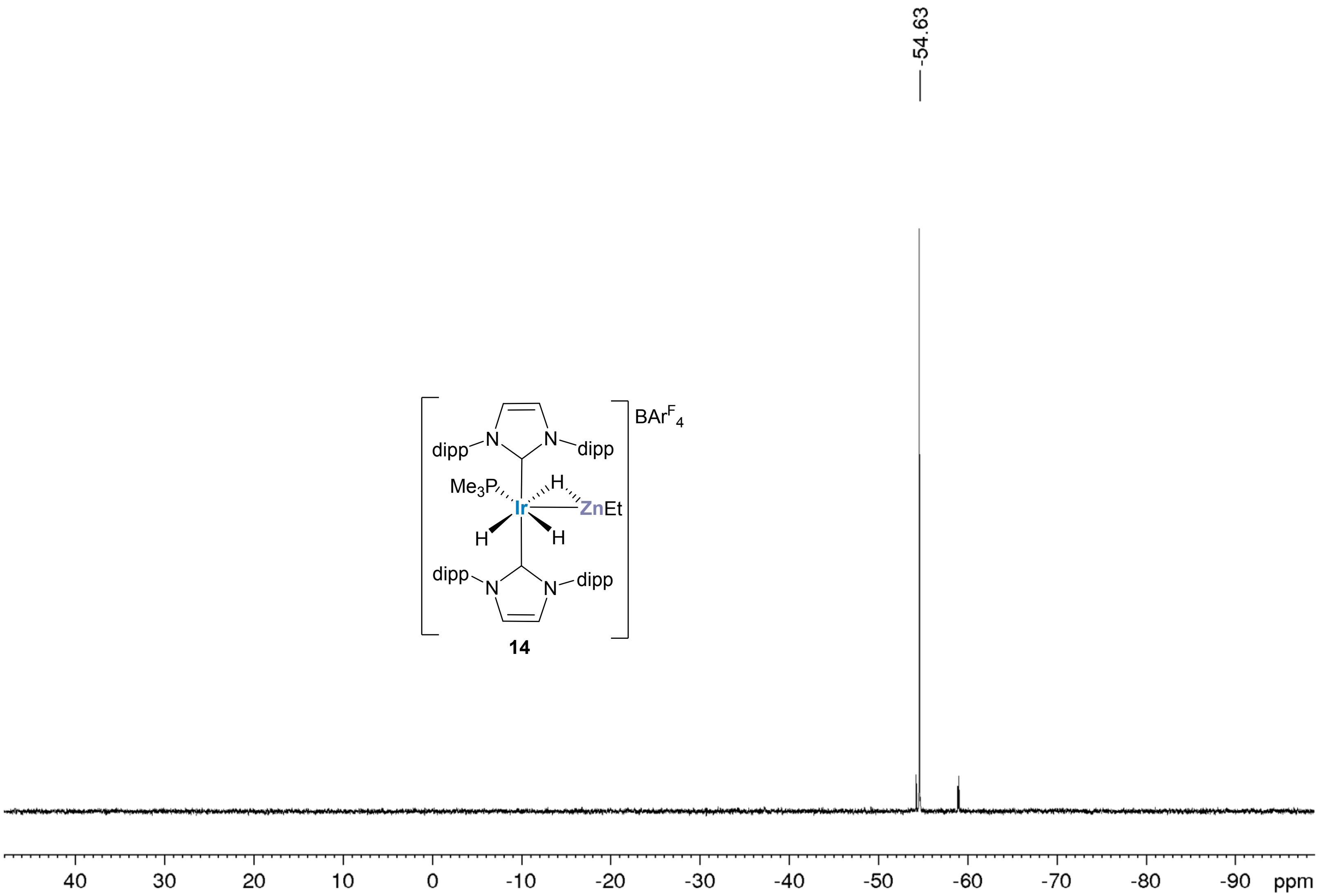

Figure S120. $^{31}P\{^1H\}$ NMR spectrum (CD_2Cl_2, 162 MHz, 298 K) of $[Ir(IPr)_2(ZnEt)(PMe_3)H_3][BAr^F_4]$ (**14**).

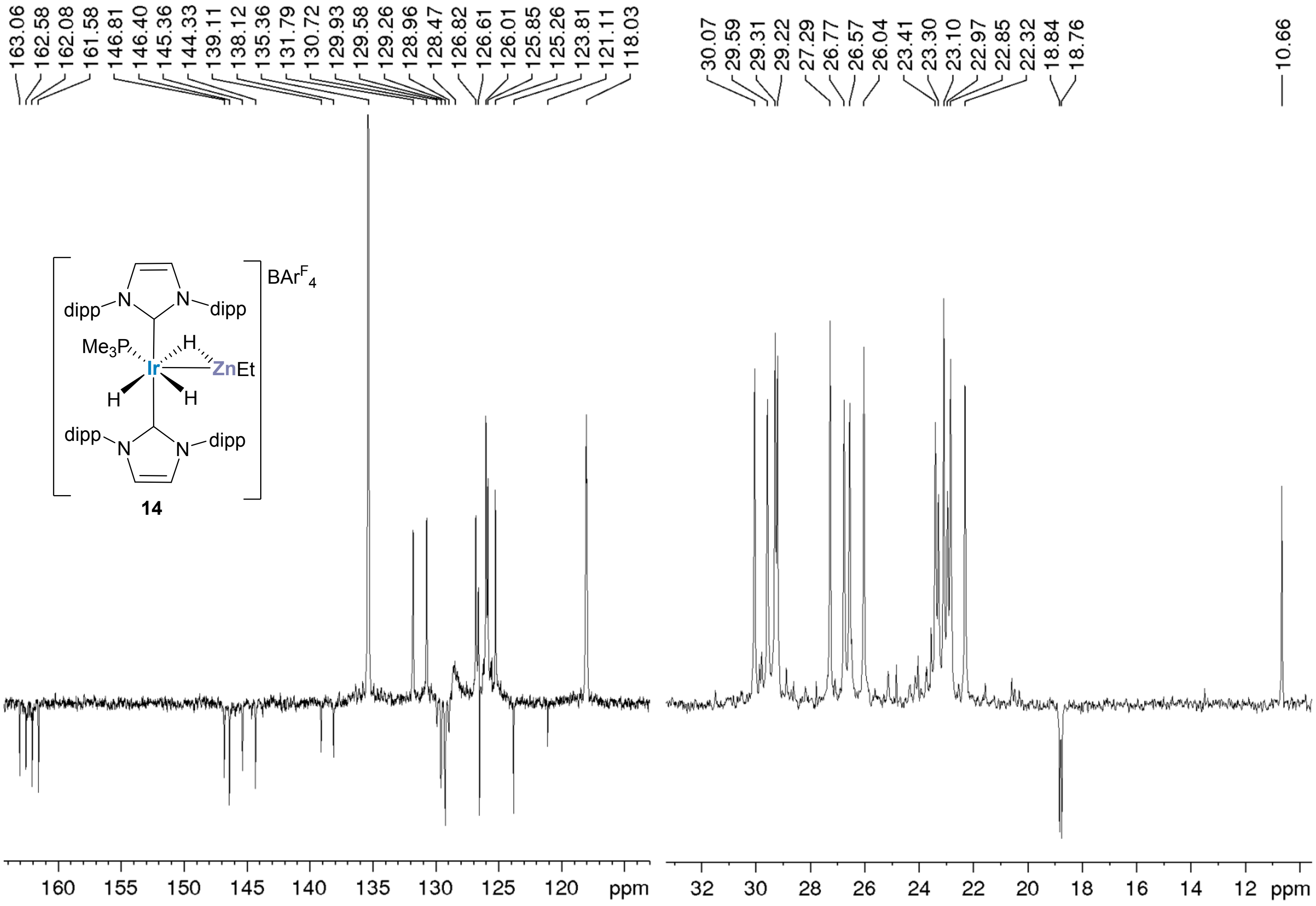

Figure S121. $^{13}C\{^{1}H\}$ DEPTQ NMR spectrum (CD_2Cl_2, 101 MHz, 298 K) of $[Ir(IPr)_2(ZnEt)(PMe_3)H_3][BAr^F_4]$ (**14**).

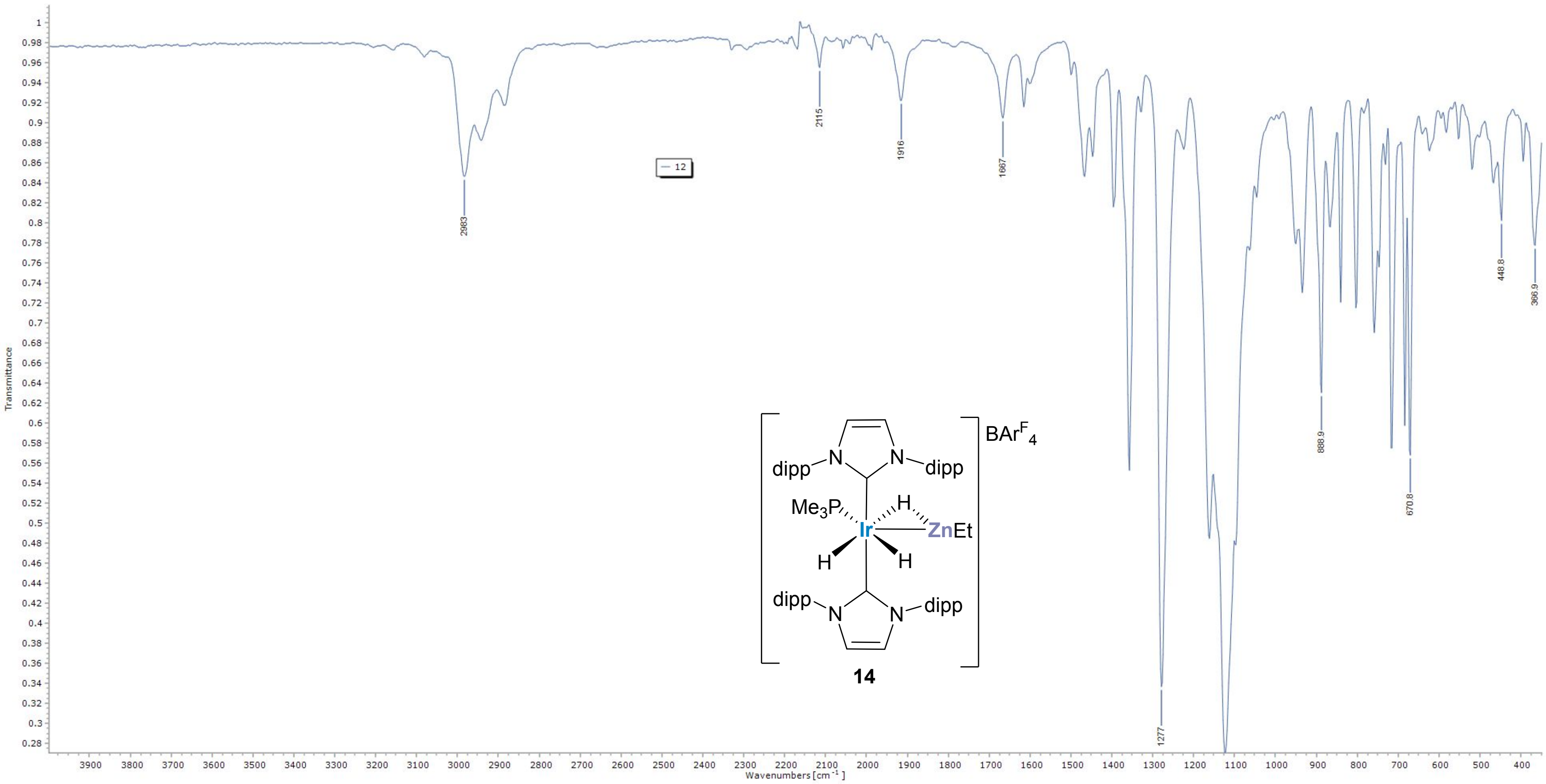

Figure S122. ATR-IR spectrum of $[Ir(IPr)_2(ZnEt)(PMe_3)H_3][BAr^F{}_4]$ (**14**).

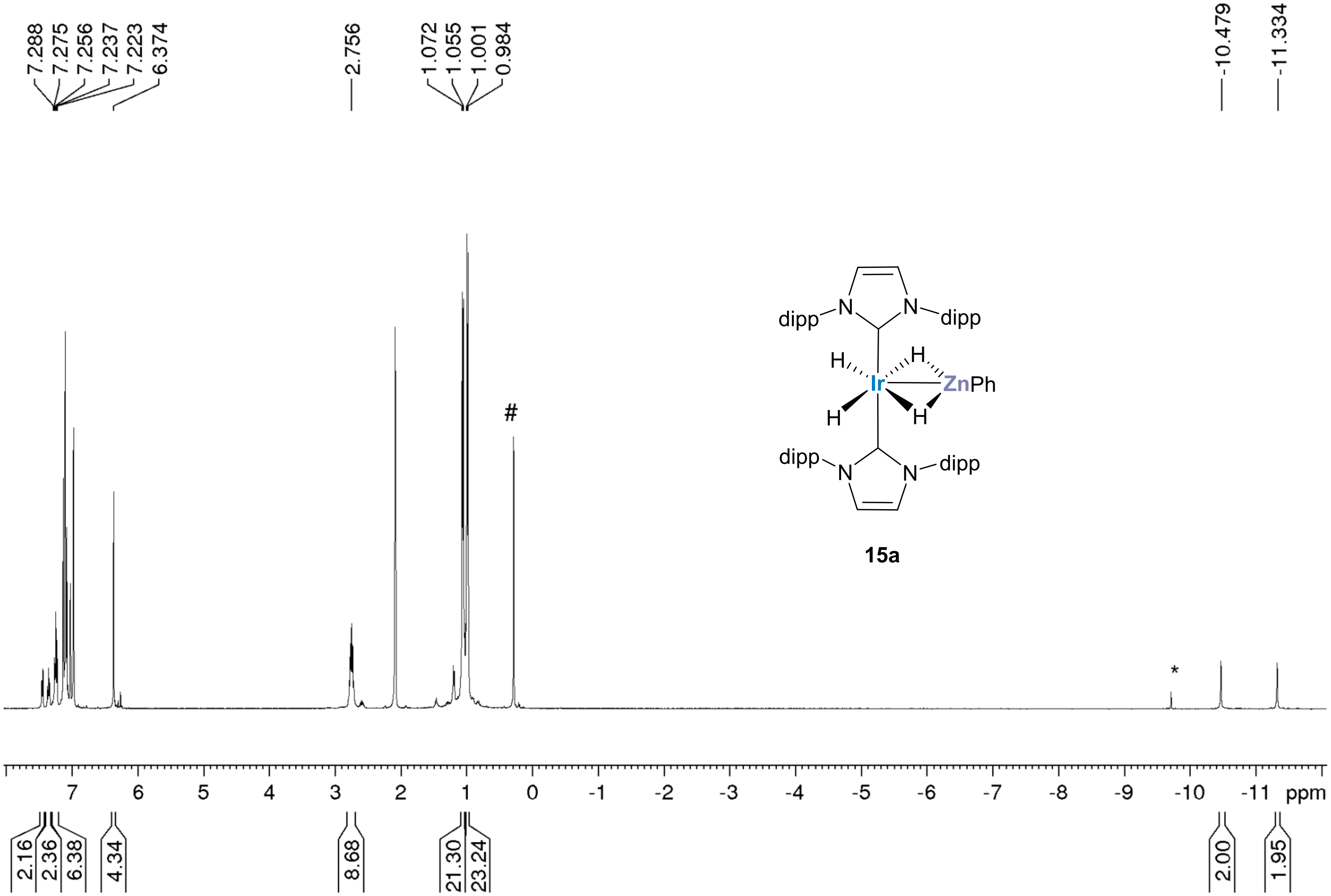

Figure S123. ^{1}H NMR spectrum ($C_6D_5CD_3$, 400 MHz, 278 K) of $Ir(IPr)_2(ZnPh)H_4$ (**15a**). (# = Si grease, * = $Ir(IPr)_2H_5$ (**13**)).

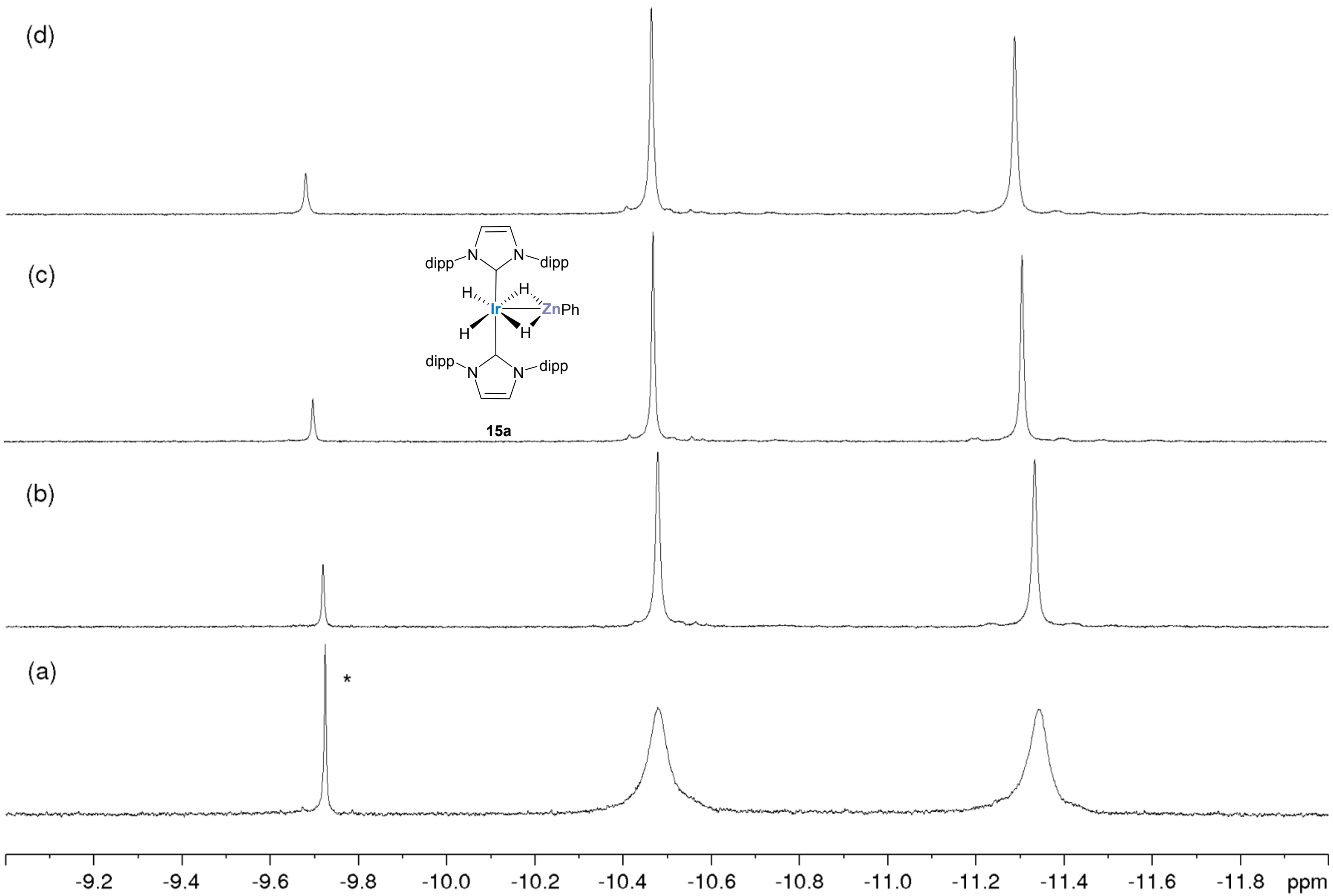

Figure S124. Hydride region of the ^{1}H NMR spectrum ($C_6D_5CD_3$, 400 MHz) of $Ir(IPr)_2(ZnPh)H_4$ (**15a**) at (a) 298, (b) 278, (c) 248 and (d) 228 K (* denotes $Ir(IPr)_2H_5$ (**13**)).

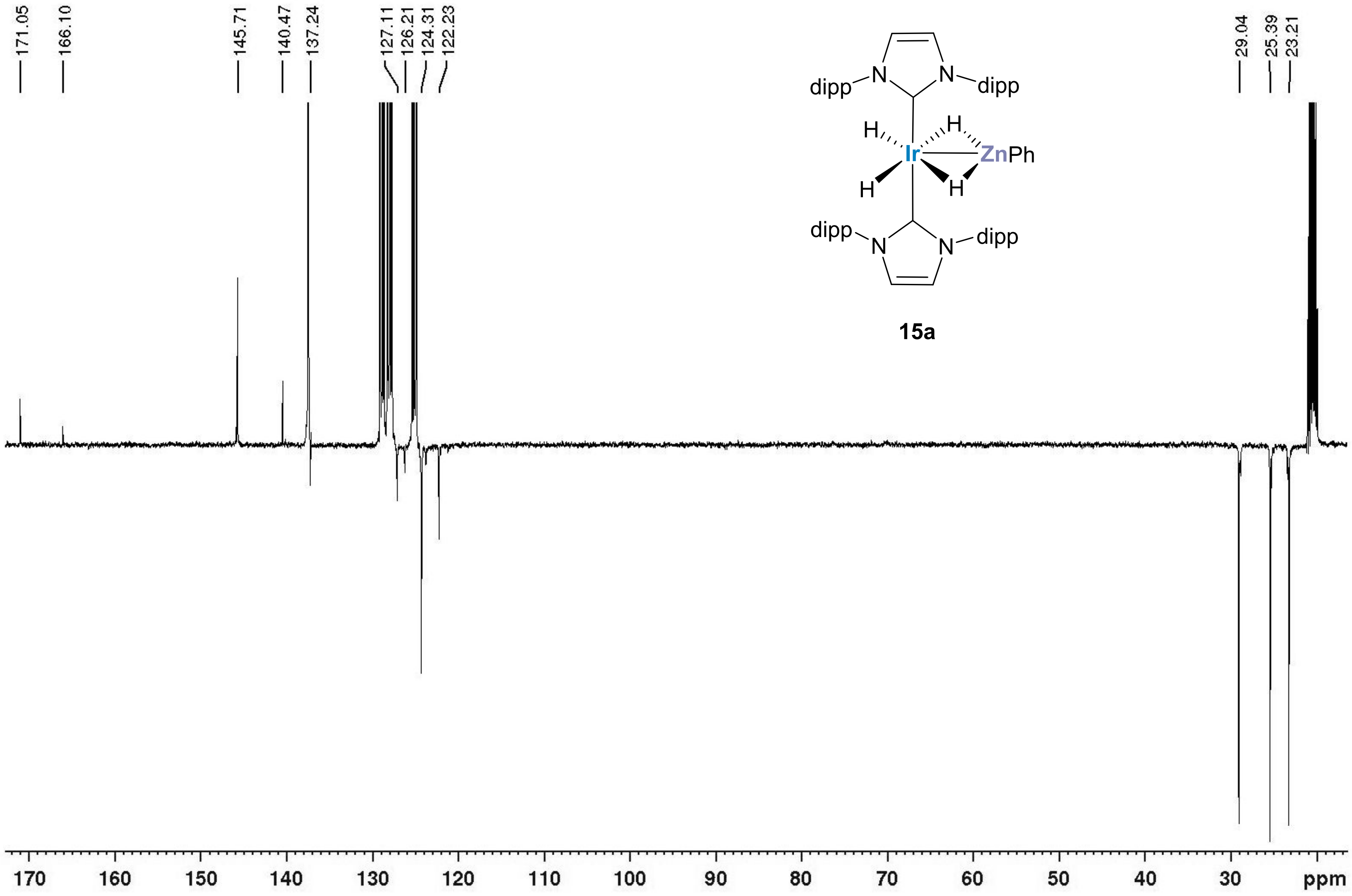

Figure S125. $^{13}C\{^1H\}$ DEPTQ NMR spectrum ($C_6D_5CD_3$, 101 MHz, 278 K) of $Ir(IPr)_2(ZnPh)H_4$ (**15a**).

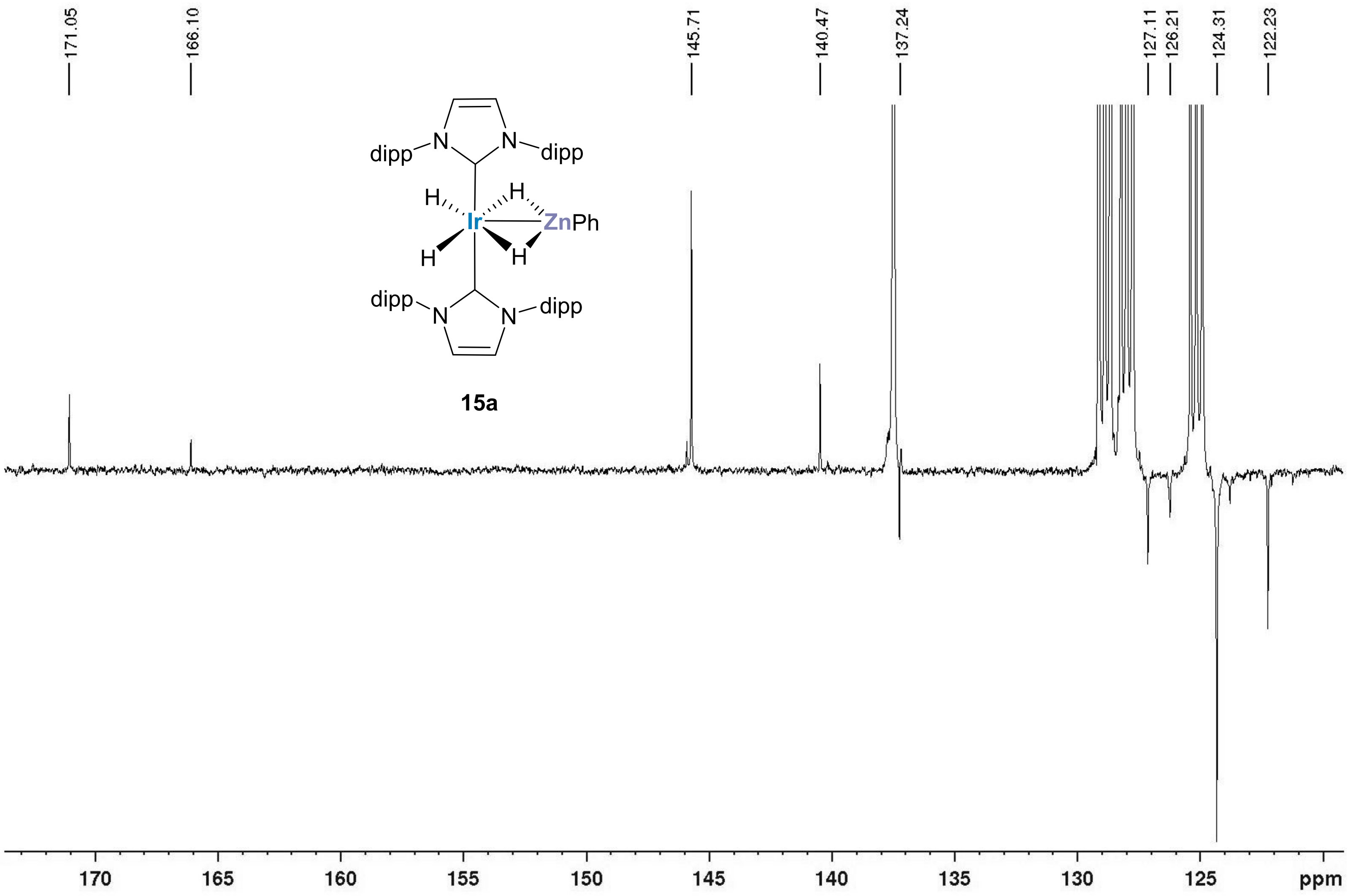

Figure S126. Aromatic region of the $^{13}C\{^1H\}$ DEPTQ NMR spectrum ($C_6D_5CD_3$, 101 MHz, 278 K) of $Ir(IPr)_2(ZnPh)H_4$ (**15a**).

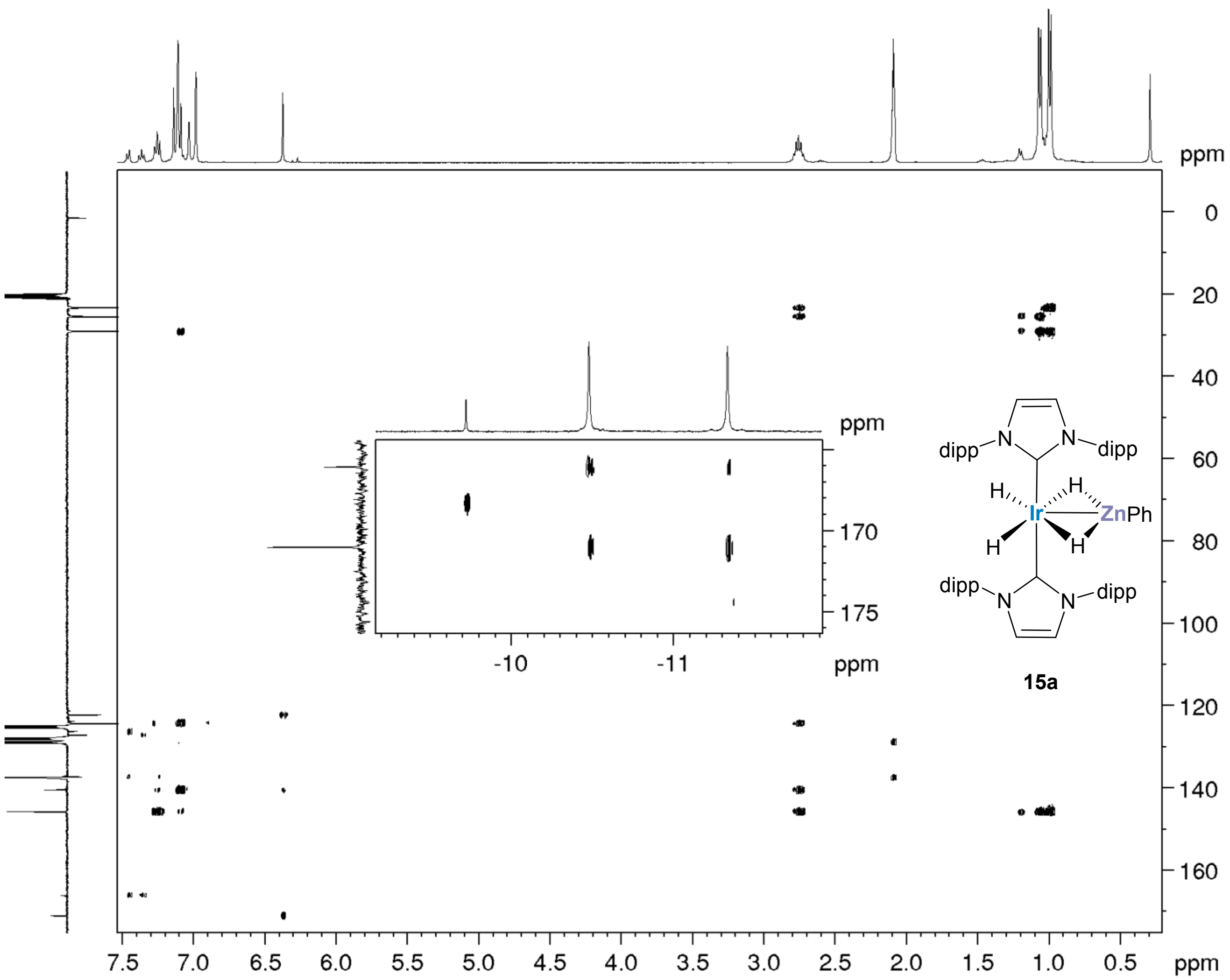

Figure S127. ^{13}C-^{1}H HMBC spectrum ($C_6D_5CD_3$, 278 K) of [Ir(IPr)$_2$(ZnPh)H$_4$ (**15a**). Inset shows correlations of hydride signals that allows identification of the Ir–C_{IPr} resonance at high frequency. Note trace of Ir(IPr)$_2$H$_5$ (**13**) mentioned in Figure S124.

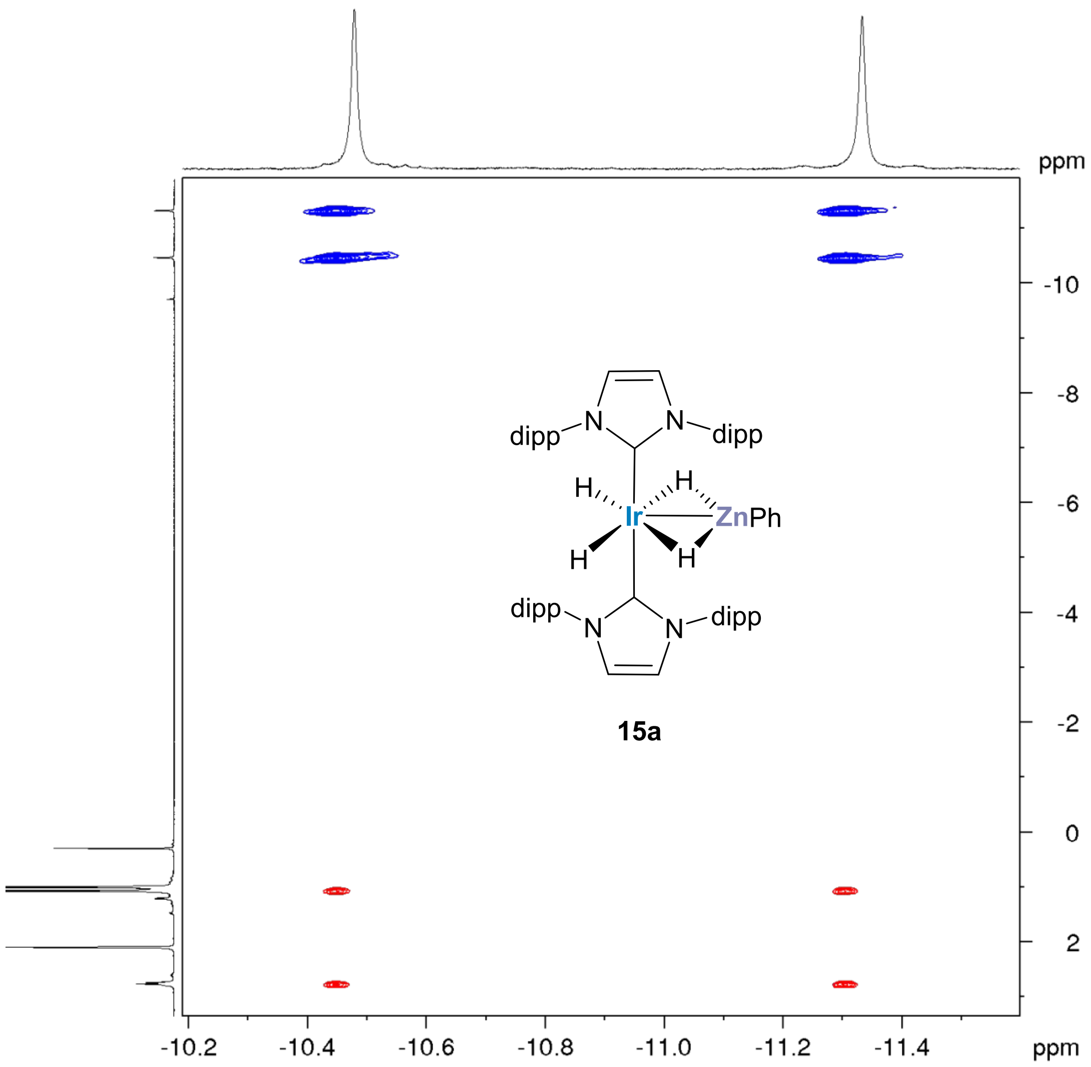

Figure S128. Hydride region of ^{1}H ROESY spectrum ($C_6D_5CD_3$, 400 MHz, 278 K) of $[Ir(IPr)_2(ZnPh)H_4$ (**15a**). The same phase of diagonal and cross-peaks indicates exchange between the hydrides. Note the change in phase to the ^{i}Pr resonances of the IPr ligands for NOE interactions.

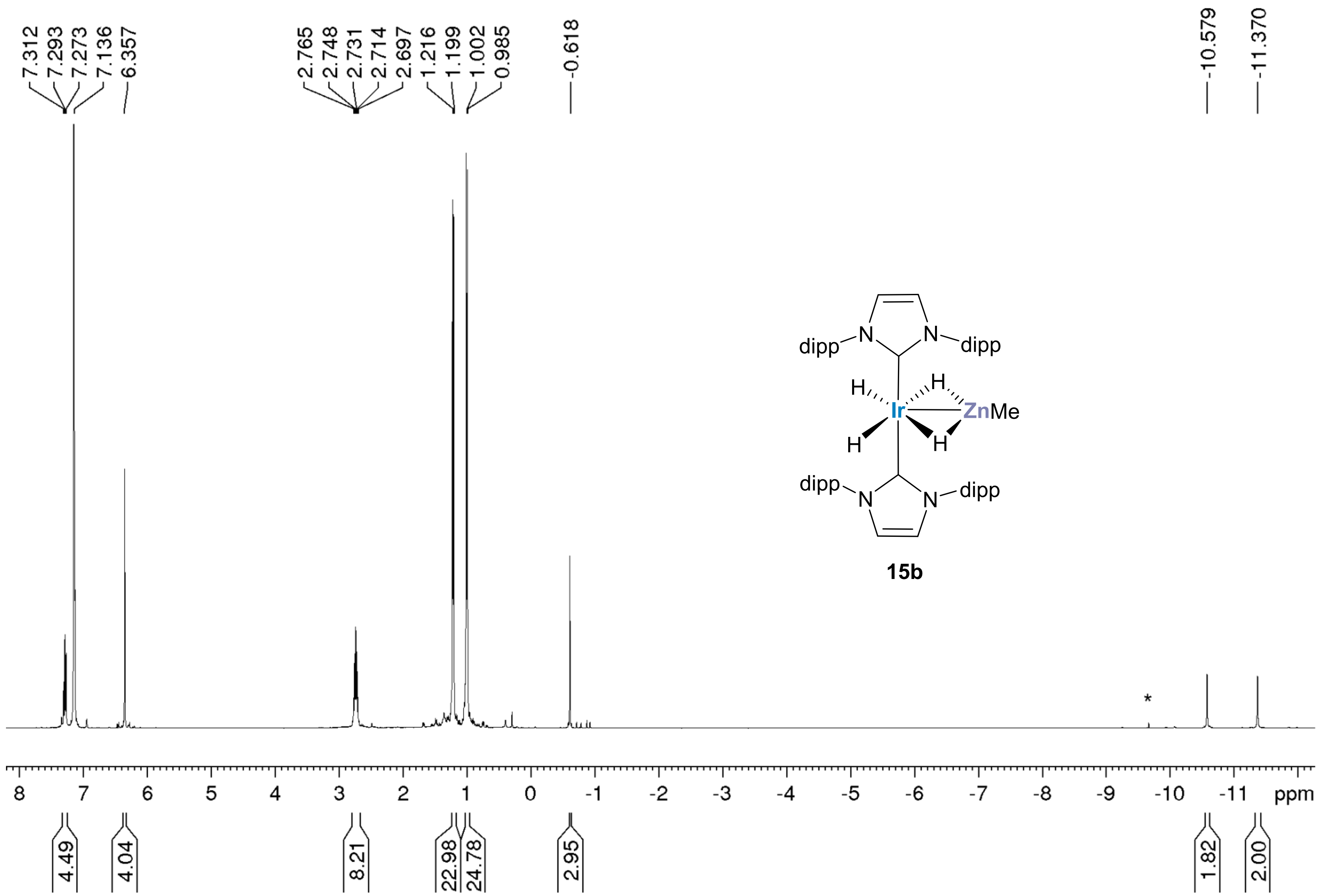

Figure S129. ^{1}H NMR spectrum (C_6D_6, 400 MHz, 298 K) of $Ir(IPr)_2(ZnMe)H_4$ (**15b**). (* = $Ir(IPr)_2H_5$ (**13**)).

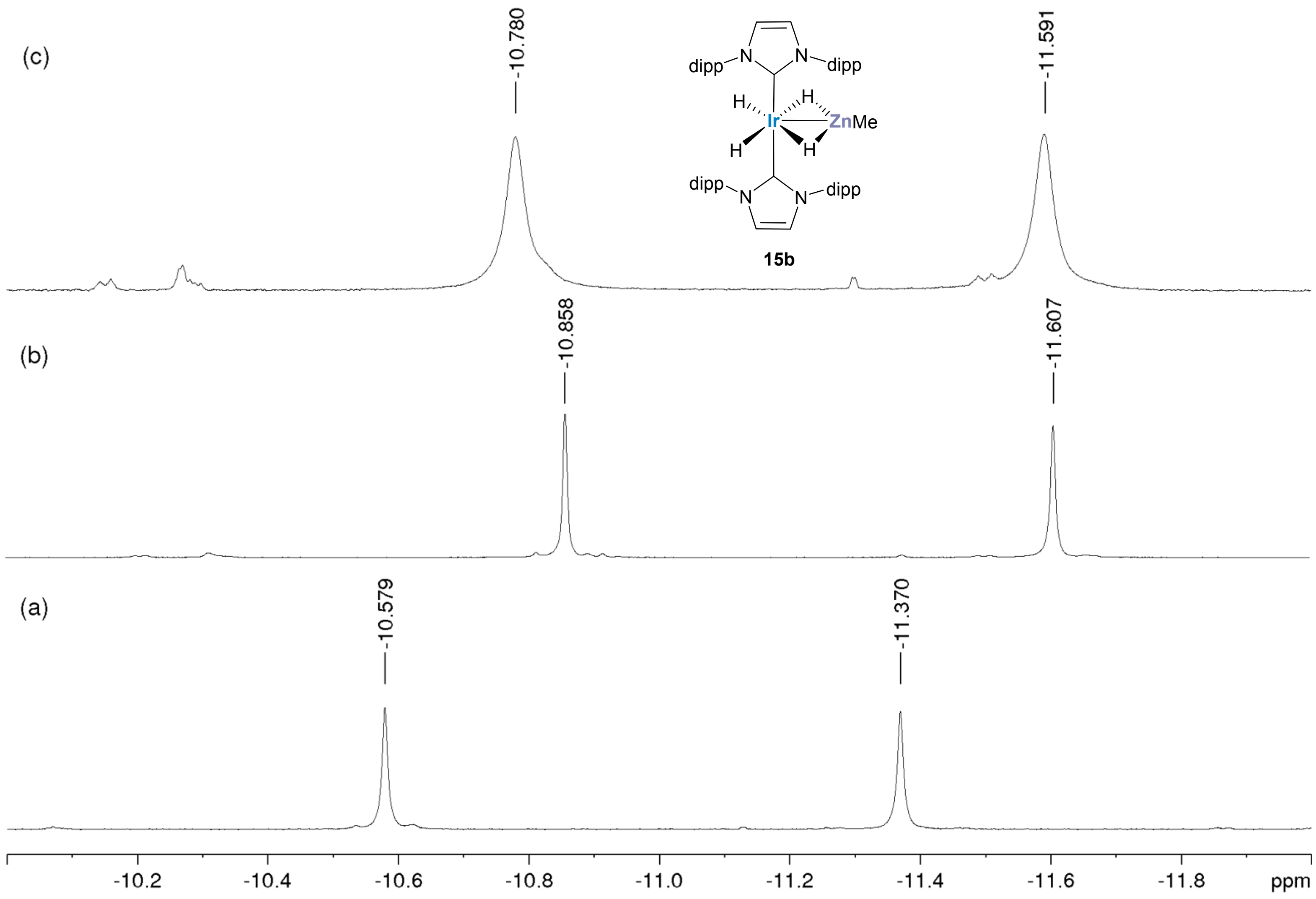

Figure S130. Hydride region of the ^1H NMR spectrum (400 MHz) of $Ir(IPr)_2(ZnMe)H_4$ (**15b**) in (a) C_6D_6 at 298 K and then in THF-d_8 at (b) 228 and (c) 318 K.

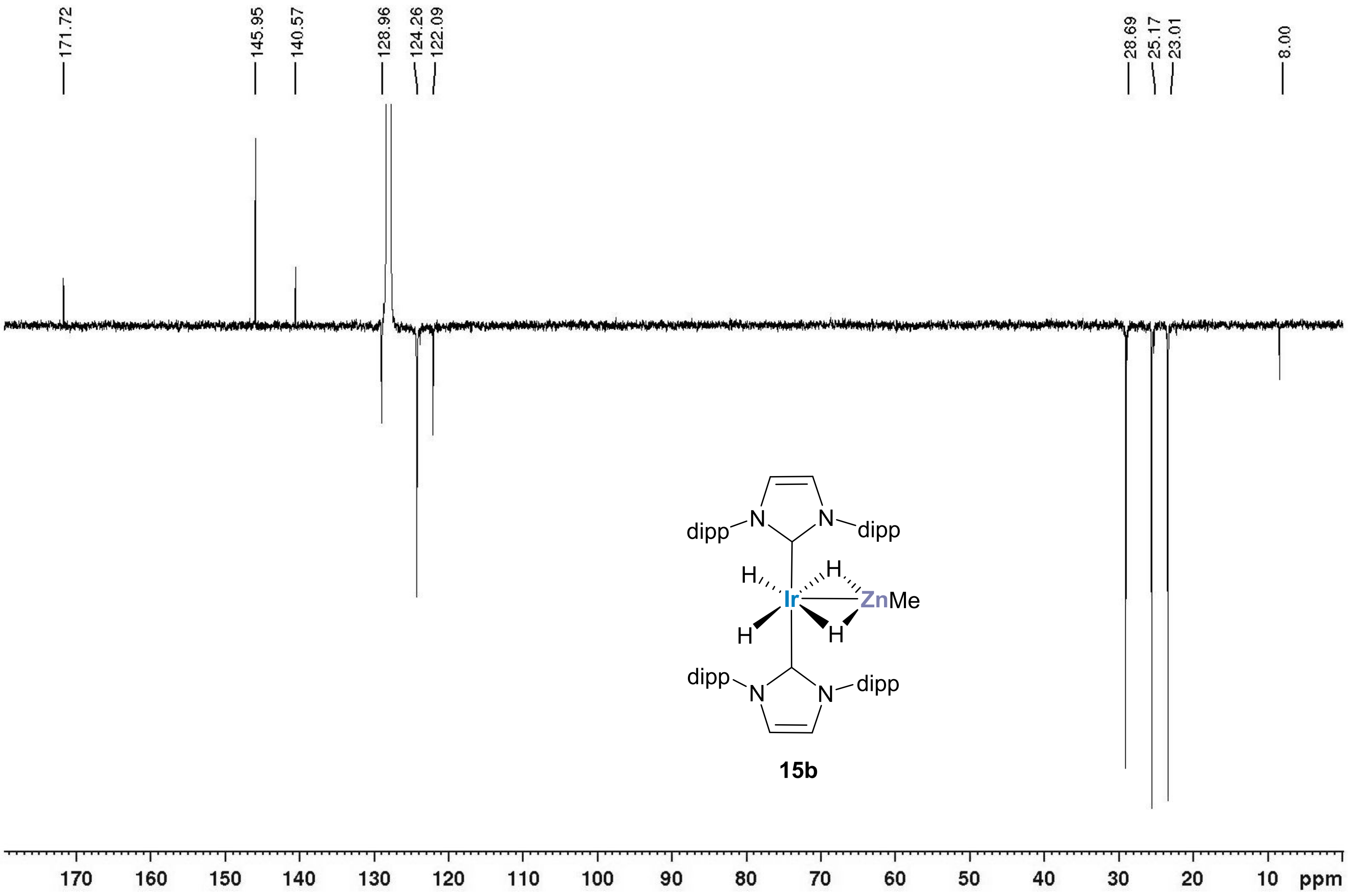

Figure S131. $^{13}C\{^{1}H\}$ DEPTQ NMR spectrum (C_6D_6, 101 MHz, 298 K) of $Ir(IPr)_2(ZnMe)H_4$ (**15b**).

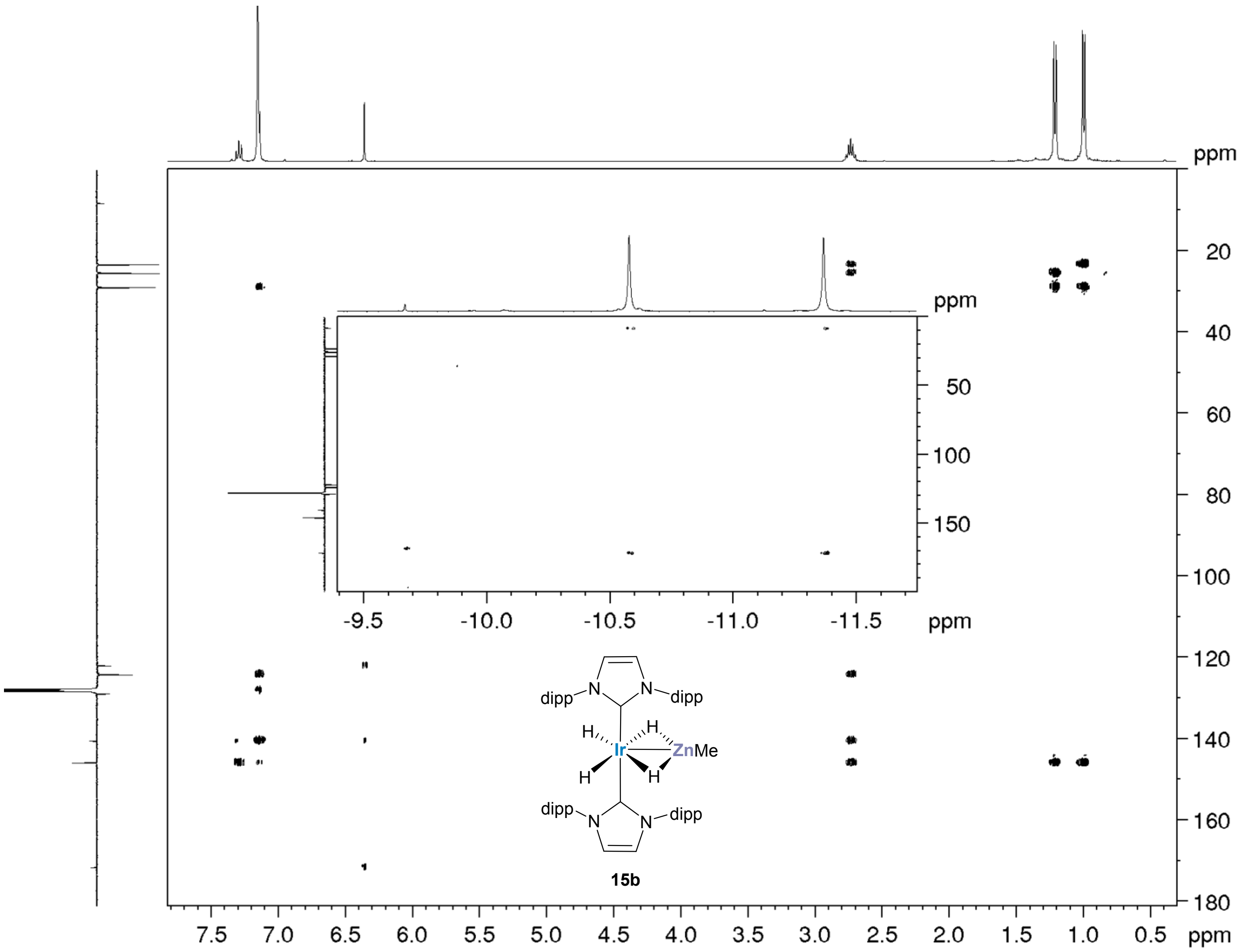

Figure S132. ^{13}C-^{1}H HMBC spectrum (C_6D_6, 298 K) of $Ir(IPr)_2(ZnMe)H_4$ (**15b**). Inset shows correlations of hydride signals that allows identification of the high frequency Ir–C_{IPr} resonance. Note trace of $Ir(IPr)_2H_5$ (**13**, δ –9.5) shown in Figure S129.

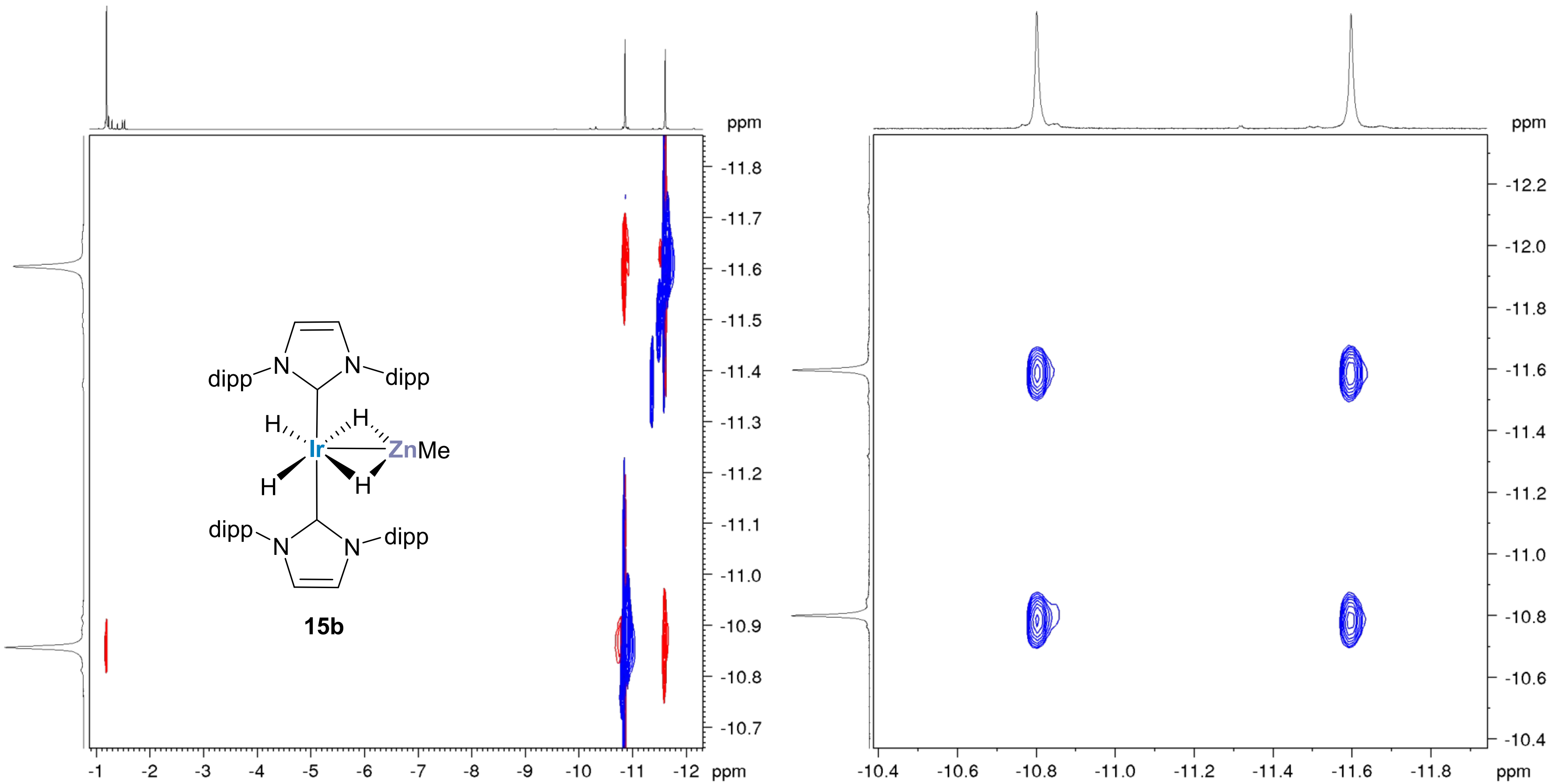

Figure S133. Hydride region of the ^{1}H ROESY spectrum (THF-d_8, 400 MHz) of Ir(IPr)$_2$(ZnMe)H$_4$ (**15b**) at (left) 228 K and (right) 298 K. At low temperature, the Ir–*H* show only NOE interactions (change in phase of the cross-peaks relative to the diagonal); not also the NOE between just the higher frequency hydride and Zn–*Me* resonance. At the lower temperature, the cross-peaks have the same phase as the diagonal, indicative of exchange.

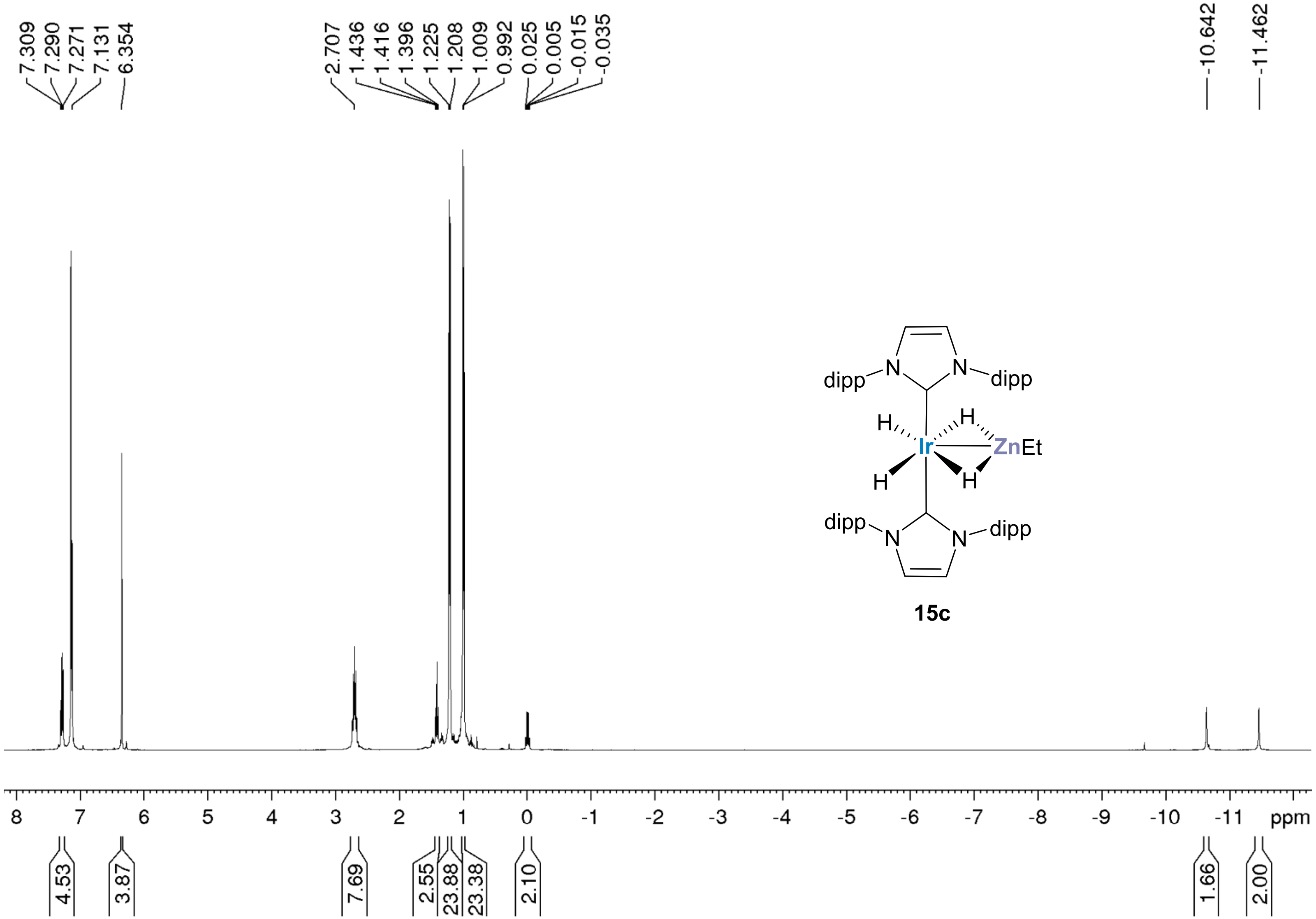

Figure S134. ^{1}H NMR spectrum (C_6D_6, 400 MHz, 298 K) of $Ir(IPr)_2(ZnEt)H_4$ (**15c**). A trace of $Ir(IPr)_2H_5$ (**13**) is present at δ –9.7.

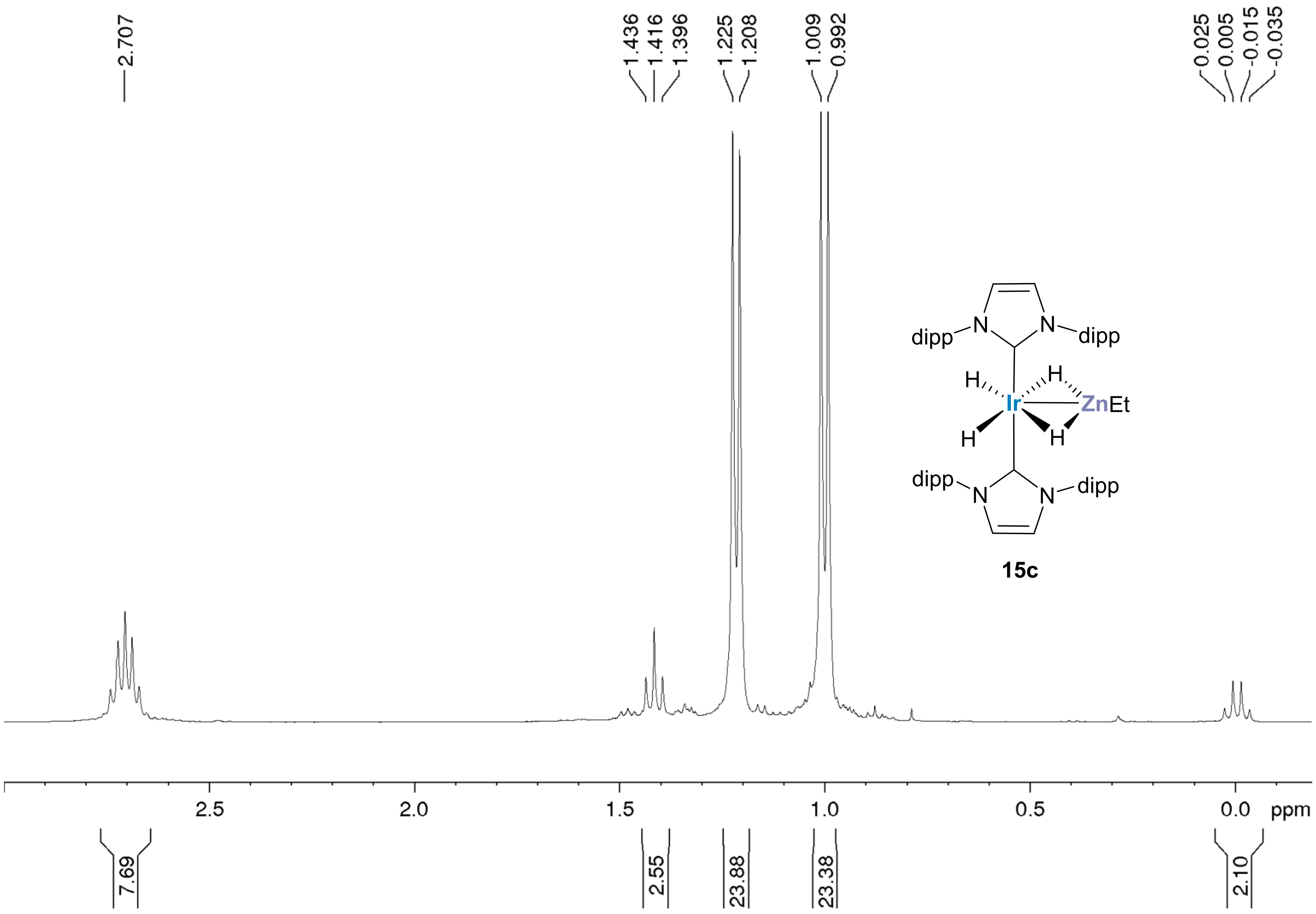

Figure S135. Alkyl region of the ^{1}H NMR spectrum (C_6D_6, 400 MHz, 298 K) of $Ir(IPr)_2(ZnEt)H_4$ (**15c**).

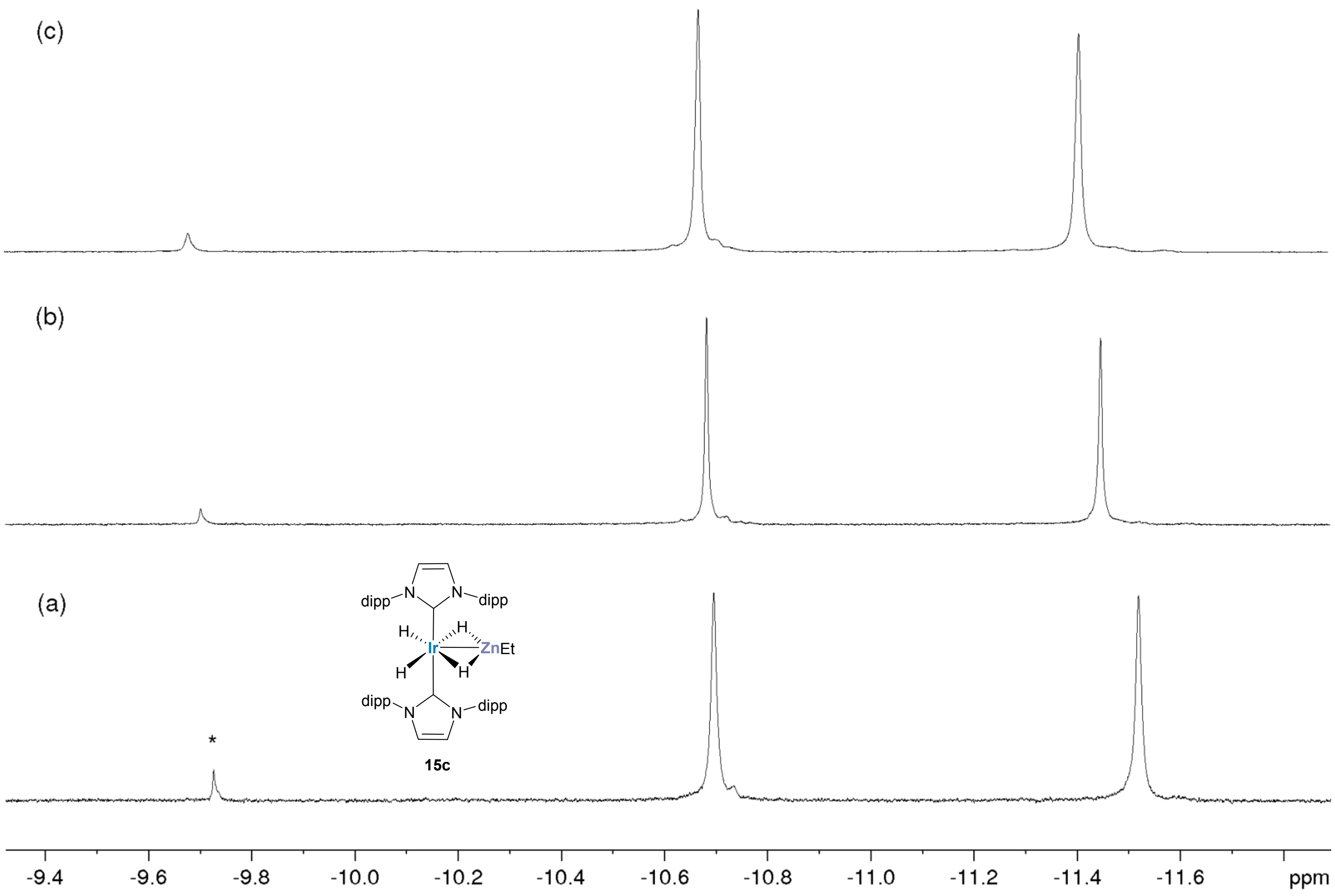

Figure S136. Hydride region of the ^{1}H NMR spectrum ($C_6D_5CD_3$, 400 MHz) of $Ir(IPr)_2(ZnEt)H_4$ (**15c**) at (a) 298, (b) 248 and (c) 228 K. (* = $Ir(IPr)_2H_5$ (**13**)).

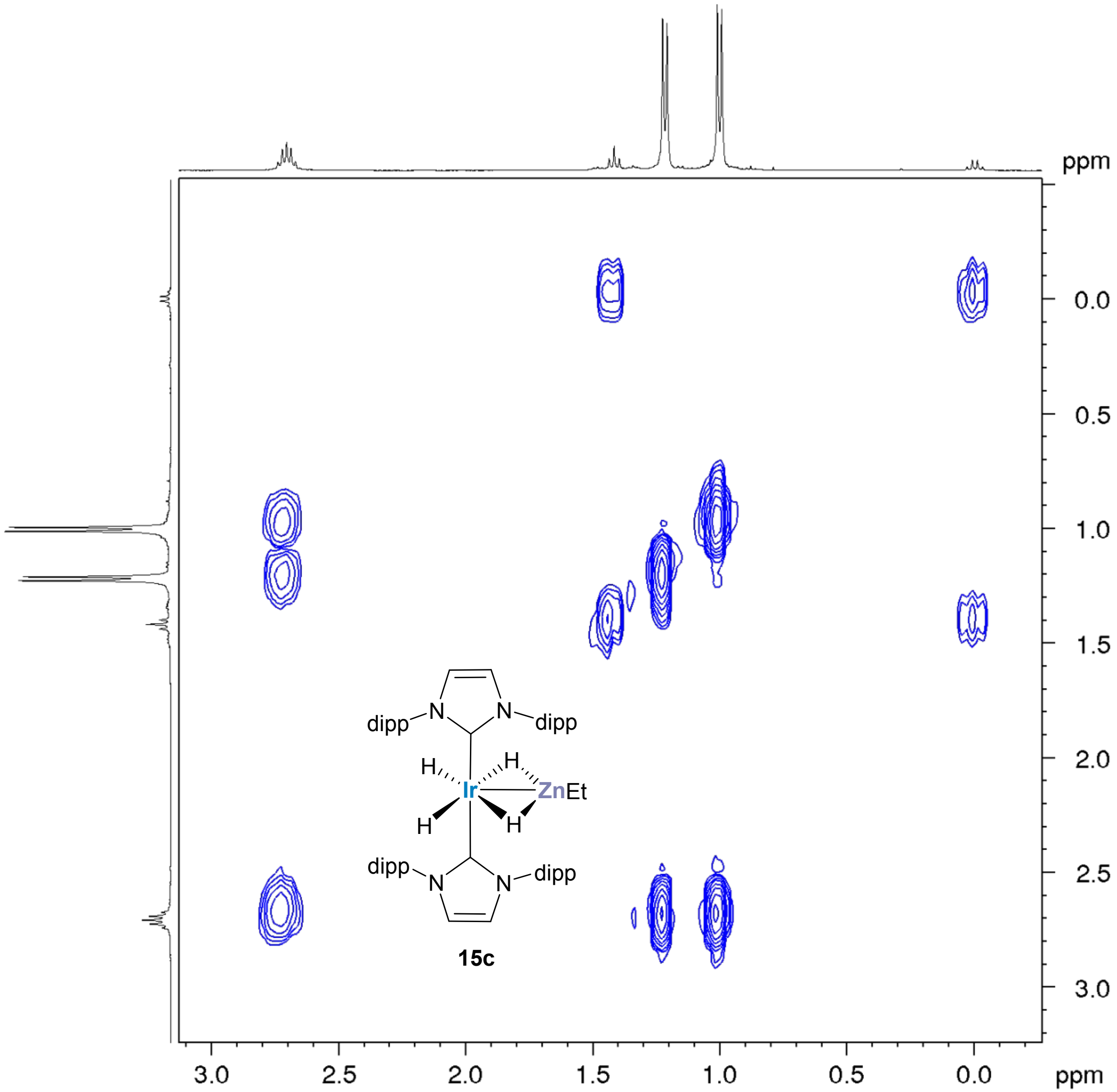

Figure S137. Alkyl region of the ^{1}H COSY NMR spectrum (C_6D_6, 400 MHz, 298 K) of $Ir(IPr)_2(ZnEt)H_4$ (**15c**).

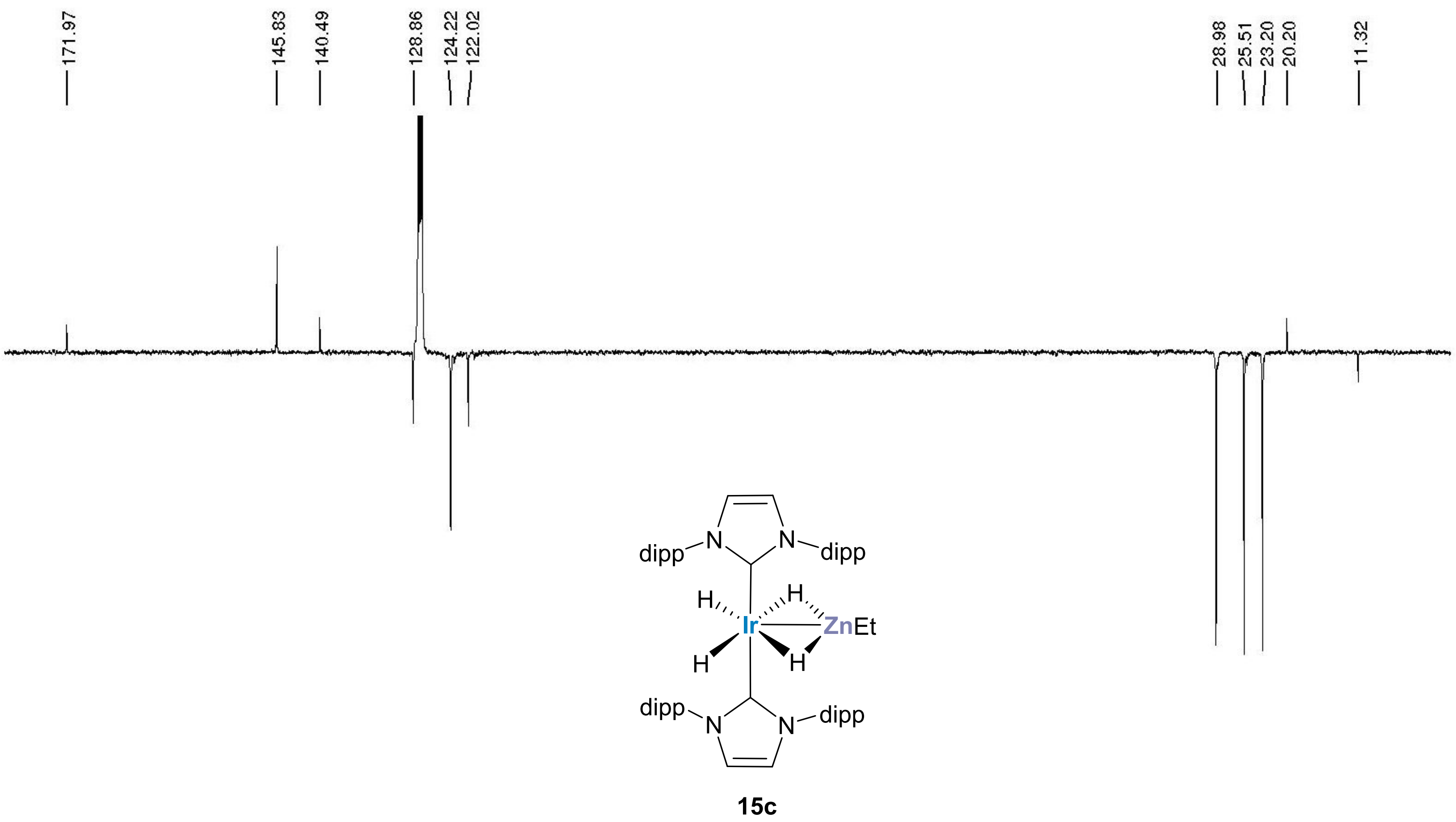

Figure S138. $^{13}C\{^1H\}$ DEPTQ NMR spectrum (C_6D_6, 101 MHz, 298 K) of $Ir(IPr)_2(ZnEt)H_4$ (**15c**).

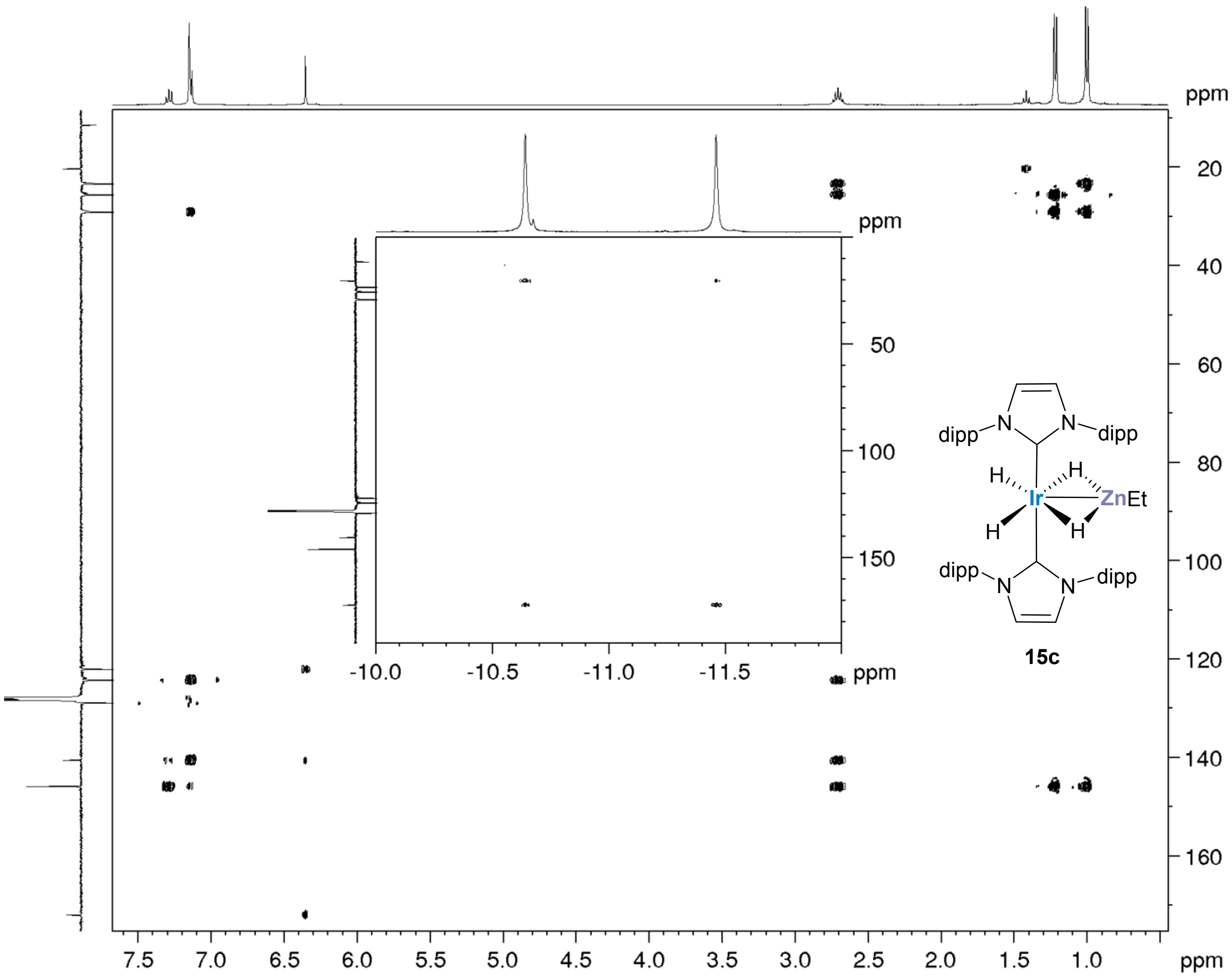

Figure S139. ^{13}C-^{1}H HMBC spectrum (C_6D_6, 298 K) of Ir(IPr)$_2$(ZnEt)H$_4$ (**15c**). Inset shows correlations of the hydride signals to the Zn−CH$_2$ and Ir−C_{IPr} resonances.

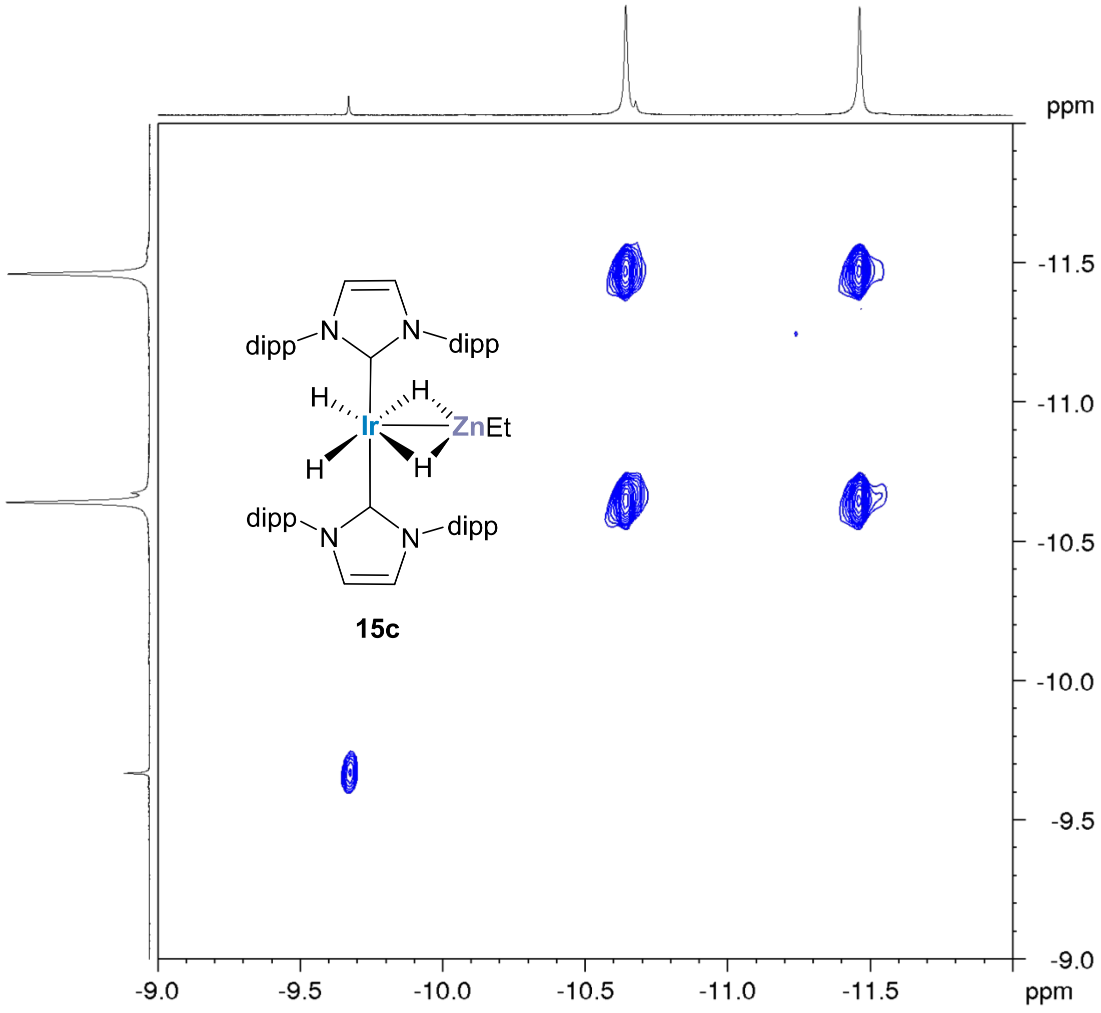

Figure S140. Hydride region of ^{1}H ROESY spectrum (C_6D_6, 400 MHz, 298 K) of [Ir(IPr)$_2$(ZnEt)H$_4$ (**15c**). The same phase of diagonal and cross-peaks indicates exchange between the hydrides. Note the small amount of Ir(IPr)$_2$H$_5$ (**13**) present at δ –9.7.

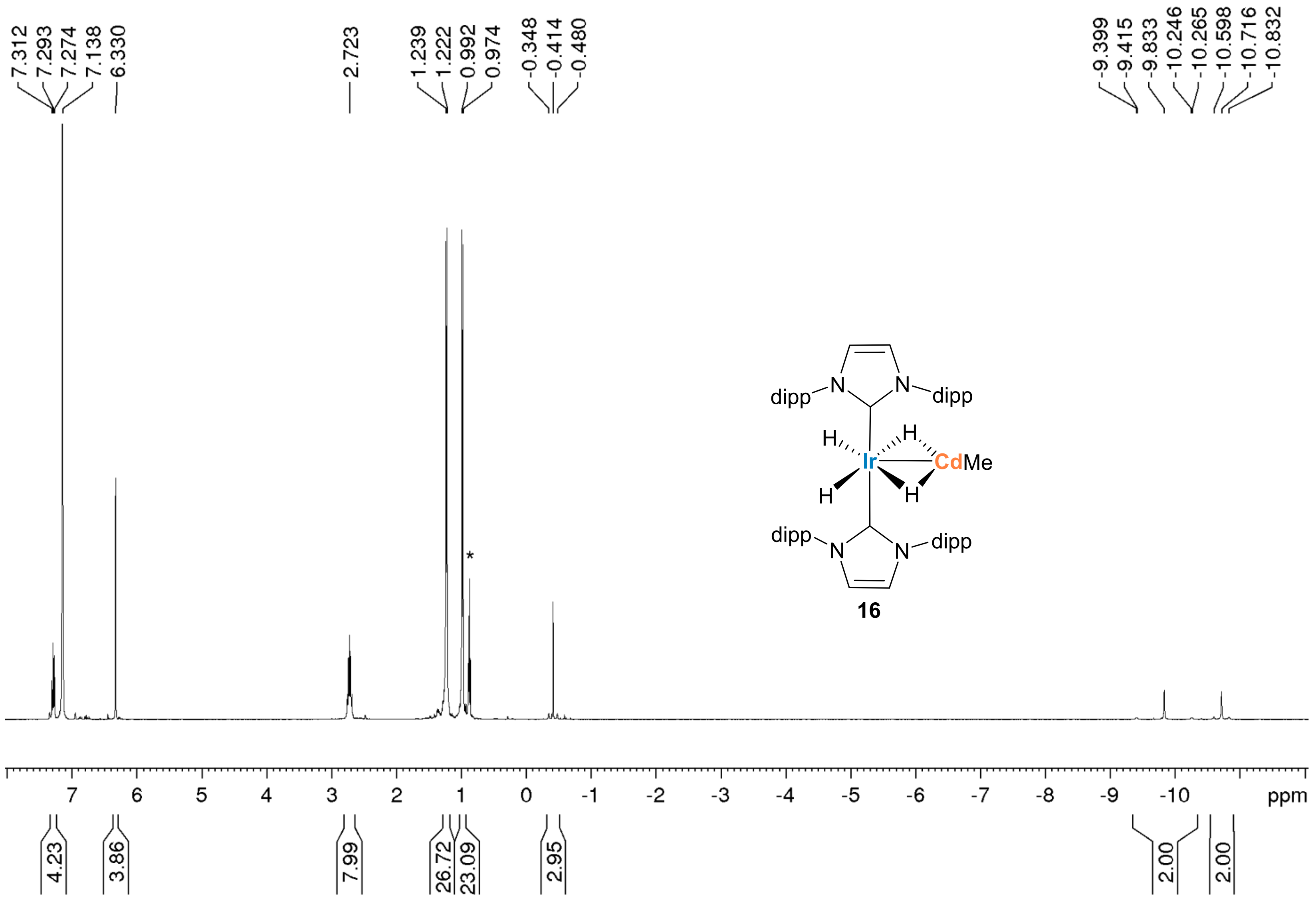

Figure S141. ^{1}H NMR spectrum (C_6D_6, 400 MHz, 298 K) of $Ir(IPr)_2(CdMe)H_4$ (**16**). (* = hexane).

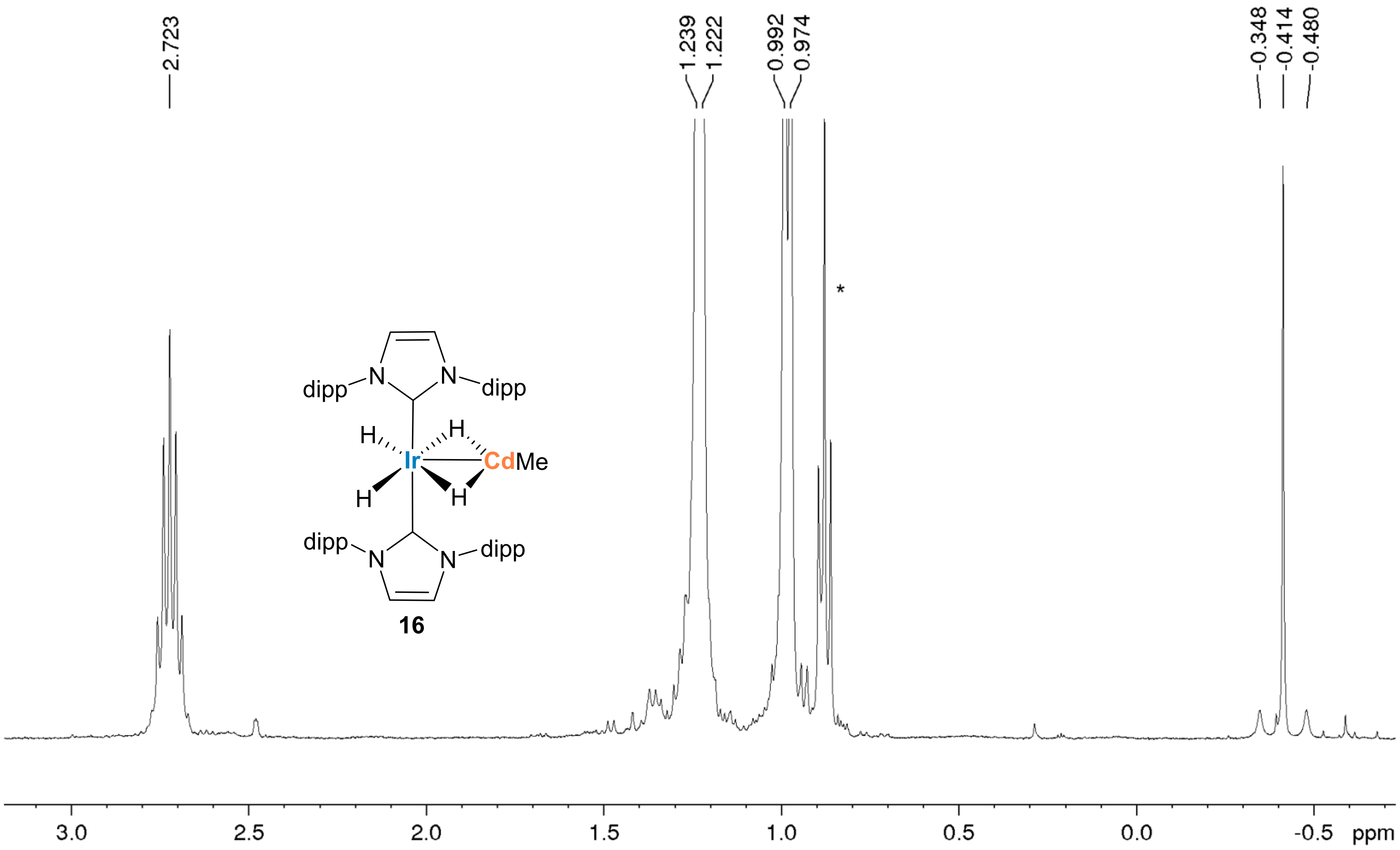

Figure S142. Alkyl region of the ^{1}H NMR spectrum (C_6D_6, 400 MHz, 298 K) of $Ir(IPr)_2(CdMe)H_4$ (**16**) (* = hexane) highlighting Cd–*Me* satellites.

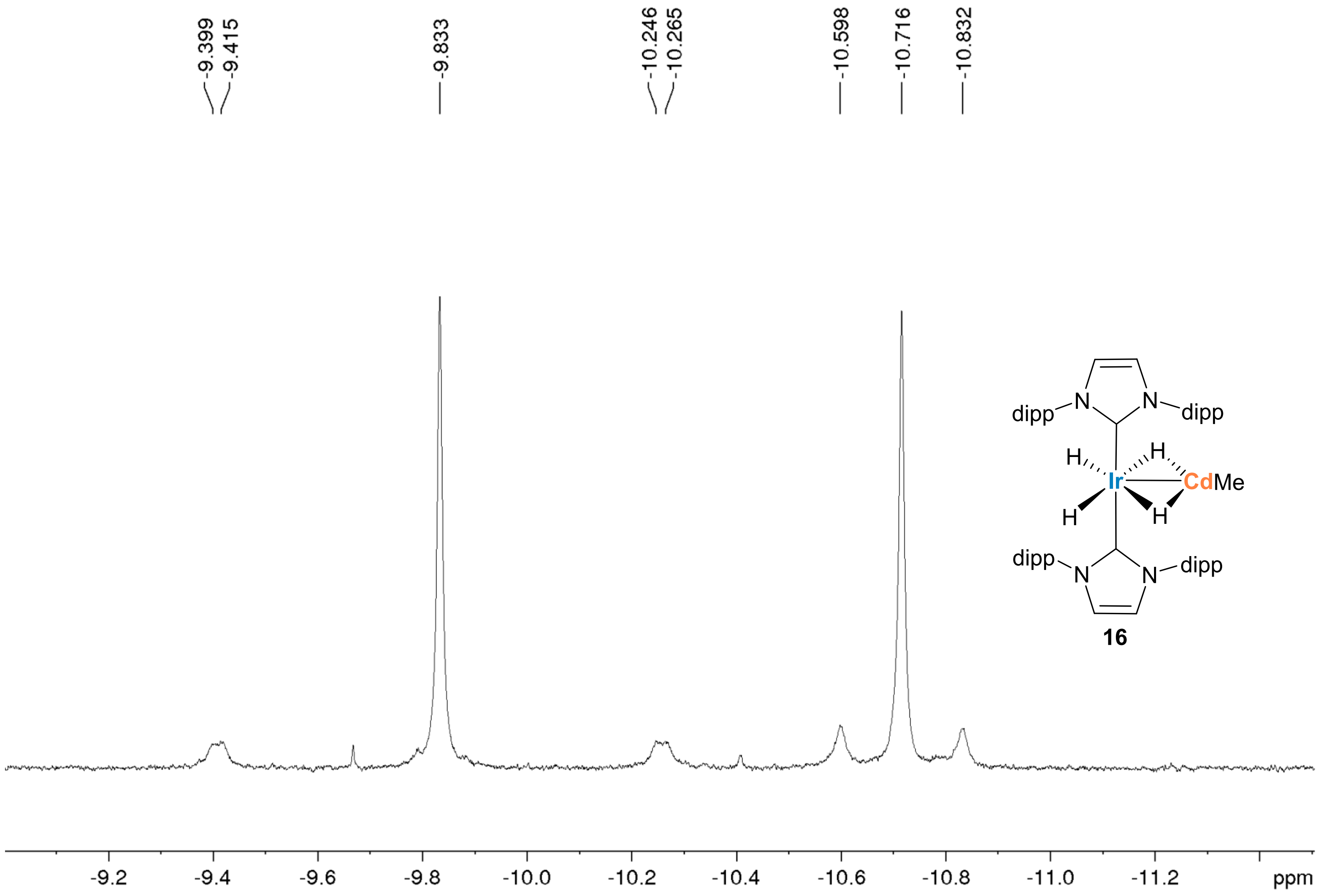

Figure S143. Hydride region of the ^{1}H NMR spectrum (C_6D_6, 400 MHz, 298 K) of $Ir(IPr)_2(CdMe)H_4$ (**16**) to highlight the Cd satellites on the Ir–*H* resonances.

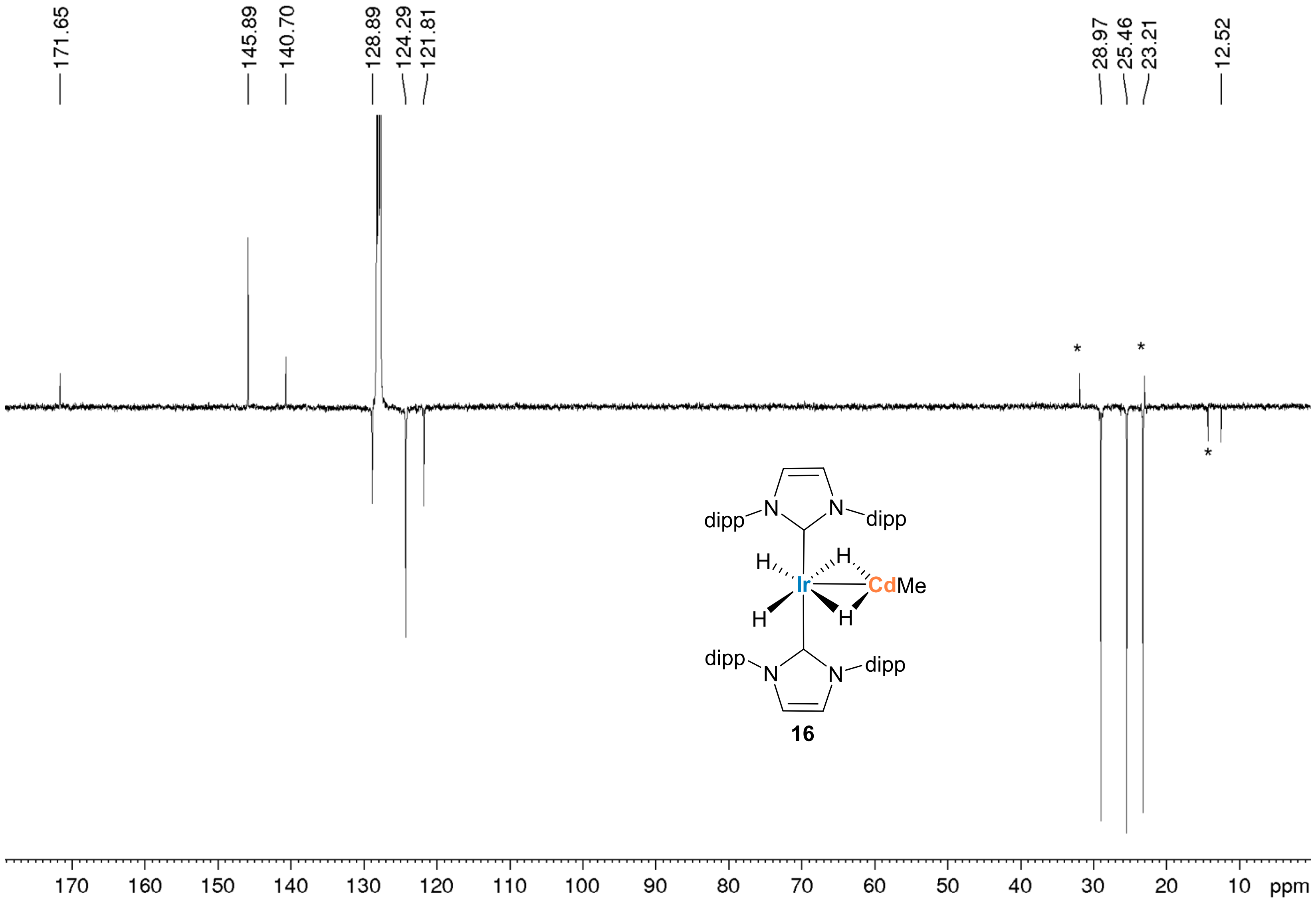

Figure S144. $^{13}C\{^{1}H\}$ DEPTQ NMR spectrum (C_6D_6, 101 MHz, 298 K) of $Ir(IPr)_2(CdMe)H_4$ (**16**). (* = hexane).

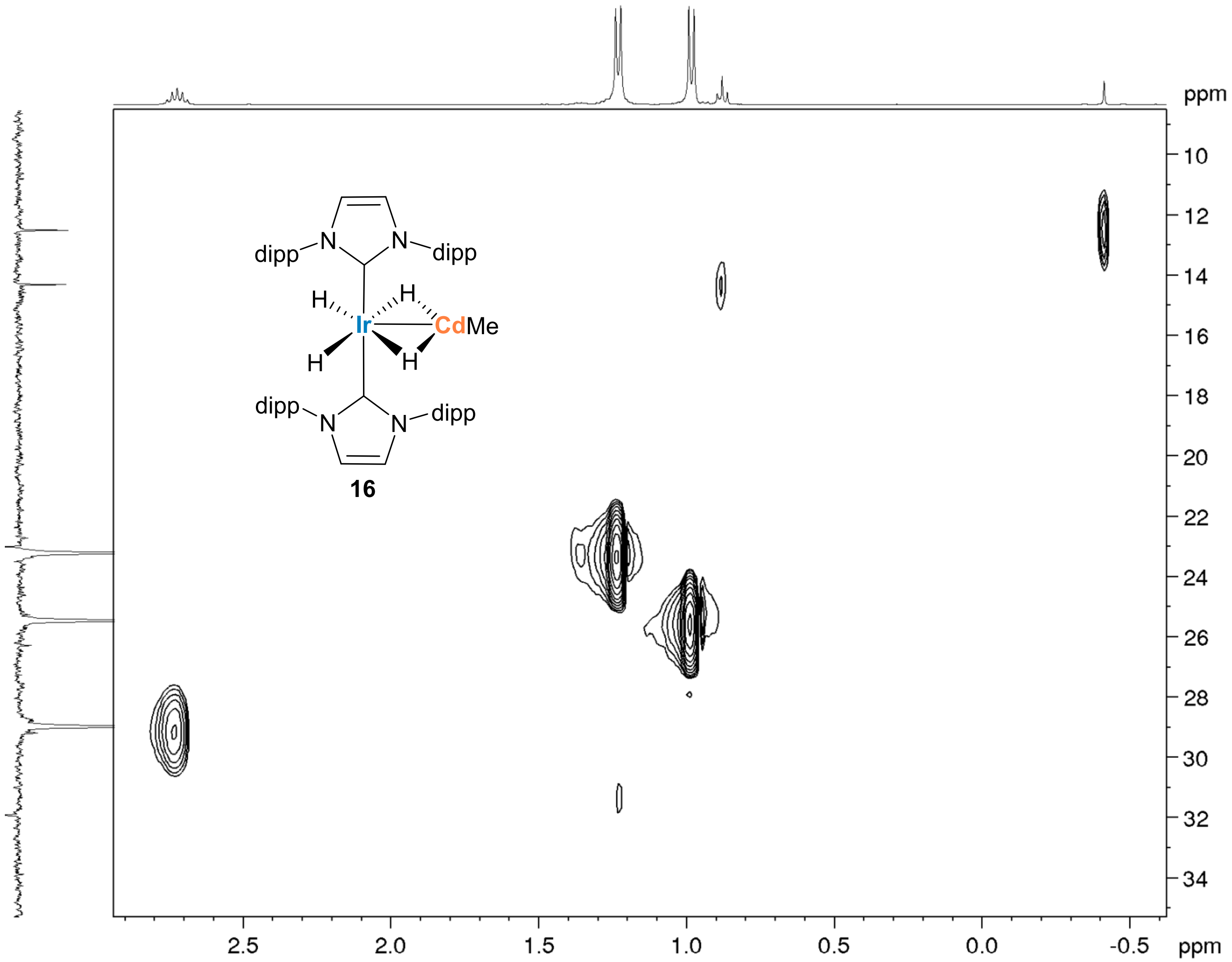

Figure S145. Alkyl region of ^{13}C-^{1}H HSQC spectrum (C_6D_6, 298 K) of $Ir(IPr)_2(CdMe)H_4$ (**16**) confirming Cd−*Me* assignment.

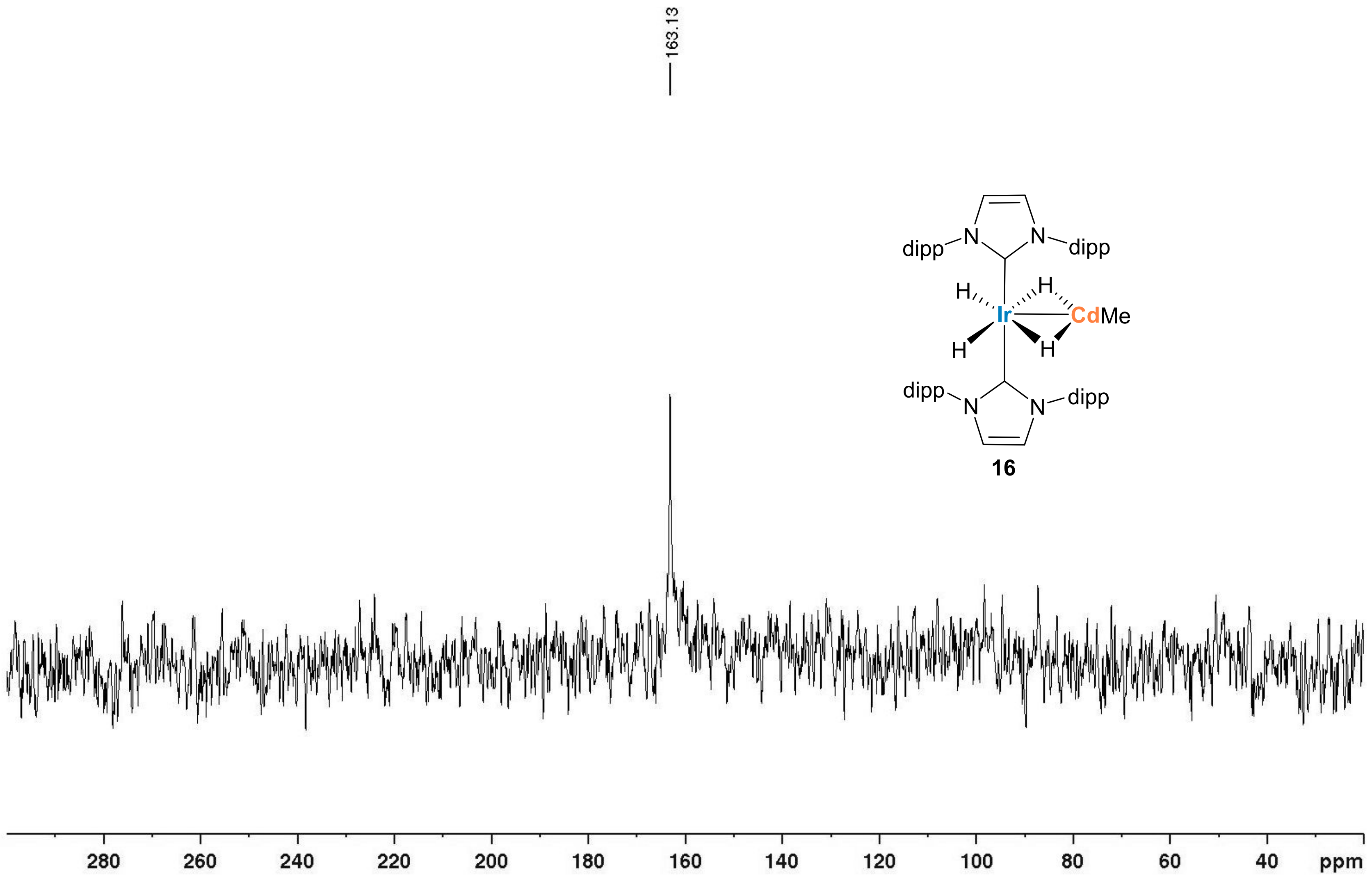

Figure S146. ^{113}Cd{^{1}H} NMR spectrum (C_6D_6, 111 MHz, 298 K) of Ir(IPr)$_2$(CdMe)H$_4$ (**16**).

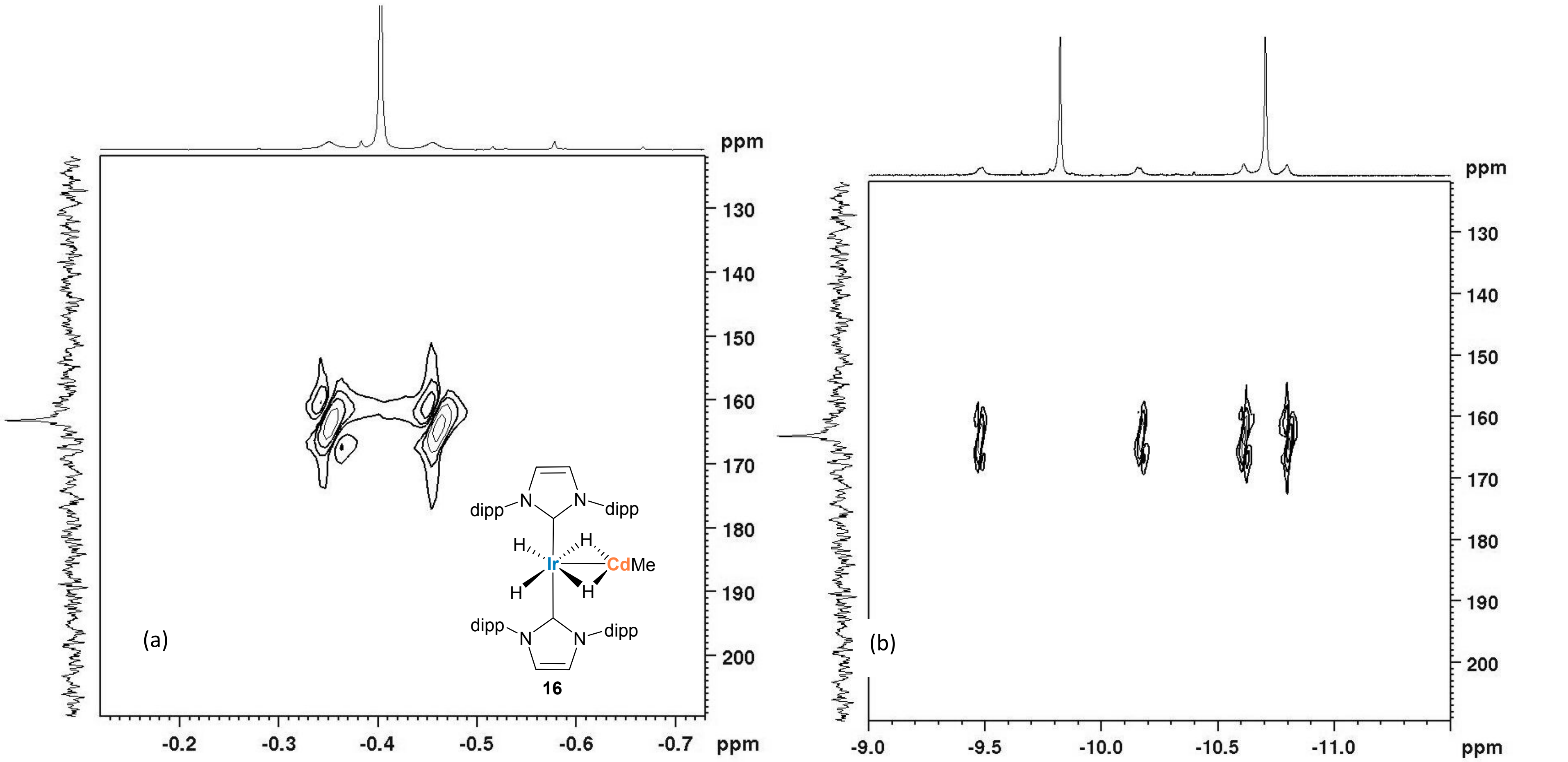

Figure S147. Correlation of (a) Cd–*Me* and (b) *H*–Ir–*H*–Cd regions in the ^{113}Cd-^{1}H HMBC spectrum (C_6D_6, 298 K) of Ir(IPr)$_2$(CdMe)H_4 (**16**).

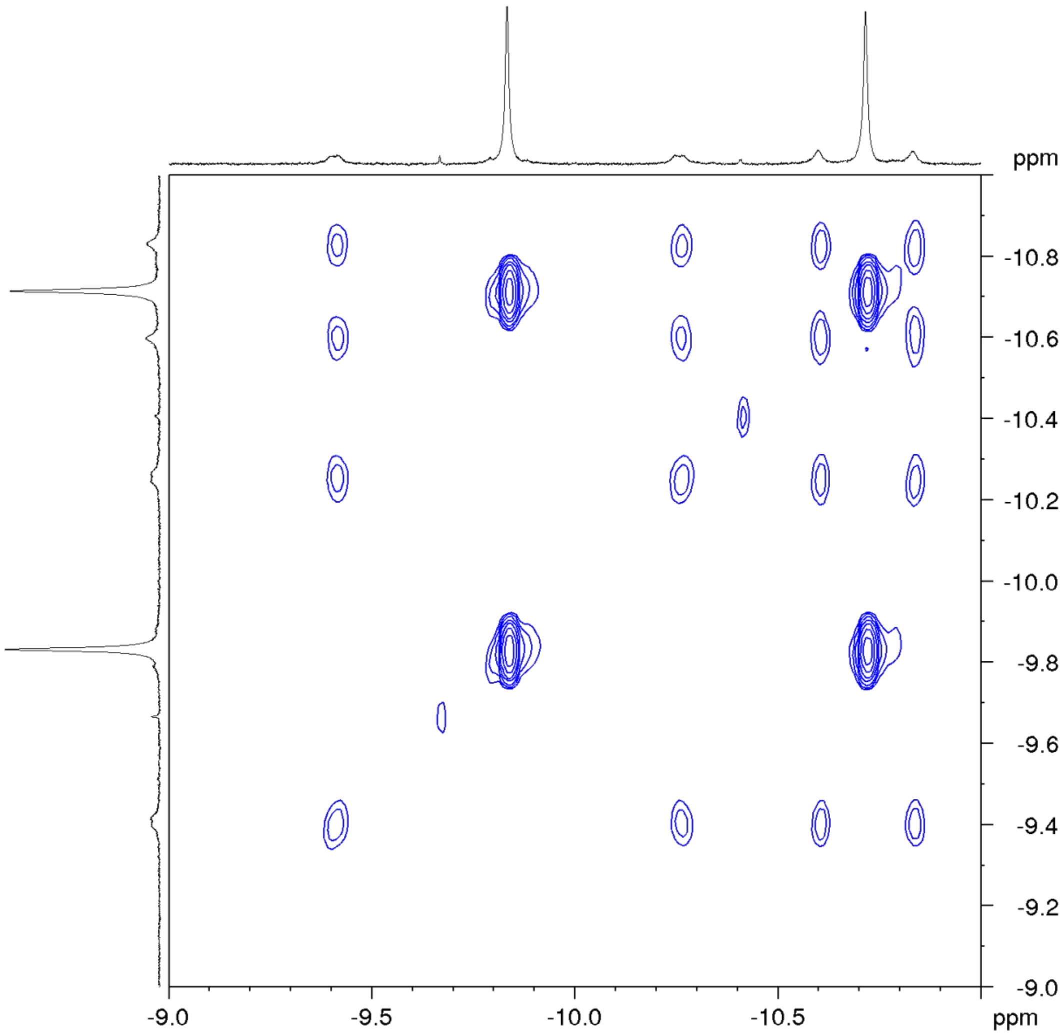

Figure S148. Hydride region of the ^{1}H ROESY NMR spectrum (C_6D_6, 400 MHz, 298 K) of $Ir(IPr)_2(CdMe)H_4$ (**16**). The same phase of diagonal and cross-peaks indicates exchange between the hydrides.

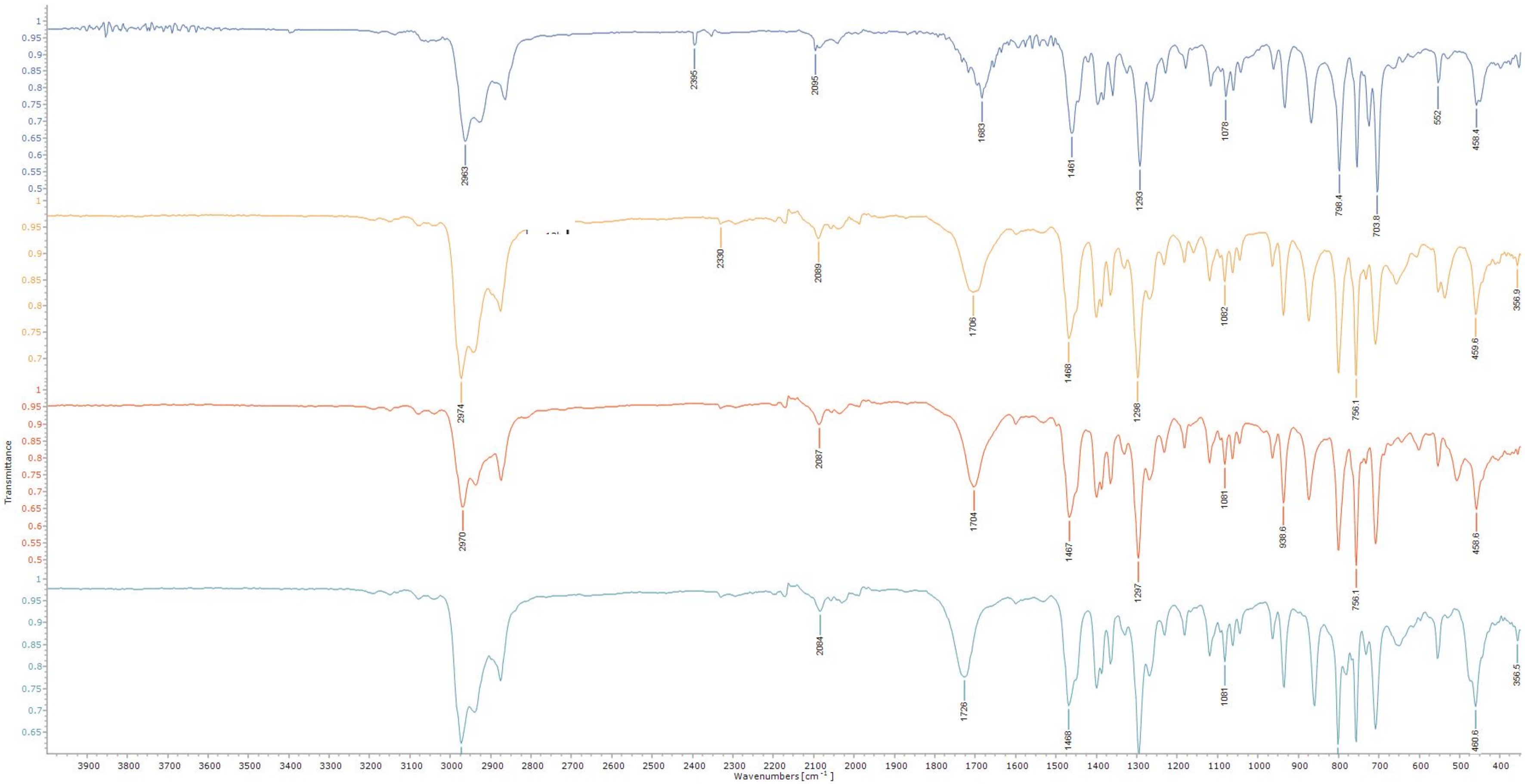

Figure S149. Overlaid ATR-IR spectra of (top-bottom) $Ir(IPr)_2(ZnPh)H_4$ (**15a**), $Ir(IPr)_2(ZMe)H_4$ (**15b**), $Ir(IPr)_2(ZnEt)H_4$ (**15c**) and $Ir(IPr)_2(CdMe)H_4$ (**16**).

S2 Computational studies

S2.1 Computational details

DFT calculations were run with Gaussian 16 (Revision C.01).[13] Zn, Cd, Ir and P centers were described with Stuttgart RECPs and associated basis sets,[14] while 6-31G** basis sets[15,16] were used for all other atoms (in this case H, C and N). An additional set of *d*-orbital polarization functions was added to P (ζ^{d}=0.387).[17] This basis set is also denoted BS1. The structures were optimized using the BP86 functional[18,19] using BS1, and all stationary points were fully characterized via analytical frequency calculations as minima (all positive eigenvalues) or transition states (one negative eigenvalue). The final reported free energies are computed considering refined electronic energies obtained with the def2-TZVP basis set (also denoted as BS2),[20,21] together with the corresponding D3(BJ) dispersion correction[22-24] and solvent effects described by the PCM model[25] (using THF or fluorobenzene as solvent). The geometries used for the quantum theory of atoms in molecules (QTAIM)[26] calculations were based on the crystal structures, with all heavy atoms remaining in their crystallographic positions and the H atoms optimized at the BP86/BS1 level of theory. The electronic densities generated at the same level of theory were then analyzed with the AIMAll program.[27]

S2.2 Computational studies on '$[ZnMe]^{+}$' abstraction

The formation of **13** from the reaction of **11b** with a Lewis base (L), shown in equation [S1], was studied computationally for the different bases considered experimentally (L = PMe_3, IMe_4, IMes; n = 0, 1, 2). The results can be found in Table S2.

$$[\mathbf{11b}]^{+} + n\,\mathrm{L} \rightarrow \mathbf{13} + [(\mathrm{L})_n\mathrm{ZnMe}]^{+} \qquad \text{[S1]}$$

L	n	ΔG (THF)	ΔG (C_6H_5F)
-	0	33.9	39.1
PMe_3	1	3.9	6.6
	2	−4.6	−3.2
IMe_4	1	−14.3	−12.5
	2	−32.5	−32.1
IMes	1	−18.3	−16.8
	2	−40.7	−40.7

Table S2. Free energy of formation of **13** and different $[(L)_nZnMe]^{+}$ adducts according to eq. [S1]. The free energy of the reaction has been obtained at the BP86-D3(BJ)(PCM=THF or C_6H_5F)/def2-TZVP//BP86/BS1 level. Energies in kcal/mol.

In agreement with experimental observation of $[(IMes)_2ZnEt][BAr^F_4]$, the computed reaction energies show the adduct with n = 2 to always be thermodynamically favored. In addition, the relative strength of the adduct (for a given n) increases as PMe_3 < IMe_4 < IMes, as expected from the ligand basicities.

Possible mechanisms for the abstraction of $[ZnMe]^{+}$ by the different Lewis bases, L, were also explored (Figures S150 and S151). In Pathway A, we considered a direct abstraction by L, where, based on the thermodynamics in Table S2, we propose that initially $[ZnMe]^{+}$ can be abstracted by a single L to form $[LZnMe]^{+}$, with a second L then adding to

drive the reaction thermodynamically. Pathway B considered initial deprotonation of the η^2-H_2 ligand in **11b** to yield **15b**, followed by the abstraction of $[ZnMe]^+$ to form the anionic $[Ir(IPr)_2(H)_4]^-$ species (**17**), which could then be re-protonated to give **13**. Finally, Pathway C assessed the possibility of abstracting neutral [(L)HZnMe] to form cationic $[Ir(IPr)_2(\eta^2$-$H_2)(H)_2]^+$ (**18**). Note that this pathway would necessitate a subsequent (formal) hydride transfer to form **13**.

Transition states for the deprotonation of **11b** (Pathway B) and for the abstraction of HZnMe by PMe_3 (Pathway C) were also located, at +10.8 and +32.9 kcal/mol, respectively. The latter was too high in free energy, and thus this pathway was not explored further. Following on the more accessible deprotonation step, we found that the subsequent abstraction of the $[ZnMe]^+$ yielding an anionic tetrahydride species was also very thermodynamically unfavored (+51.4 kcal/mol for L = PMe_3). Thus, the direct abstraction by a Lewis base seems a reasonably accessible mechanism; however, despite repeated attempts no transition state for this process could be located.

Calculations with L = IMe_4 and IMes only considered the thermodynamics of the different pathways. As with PMe_3, the abstraction of neutral [(L)HZnMe] along Pathway C is, albeit less so, an endergonic process – and certainly unfavorable compared to the alternative pathways. In contrast, the products from the deprotonation pathway along Pathway B are very thermodynamically favored, but the subsequent abstraction step would be uphill by at least □25-30 kcal/mol. Thus, in this case as well, the calculations suggest a mechanism involving direct abstraction of $[ZnMe]^+$ by the base is most likely (Pathway A).

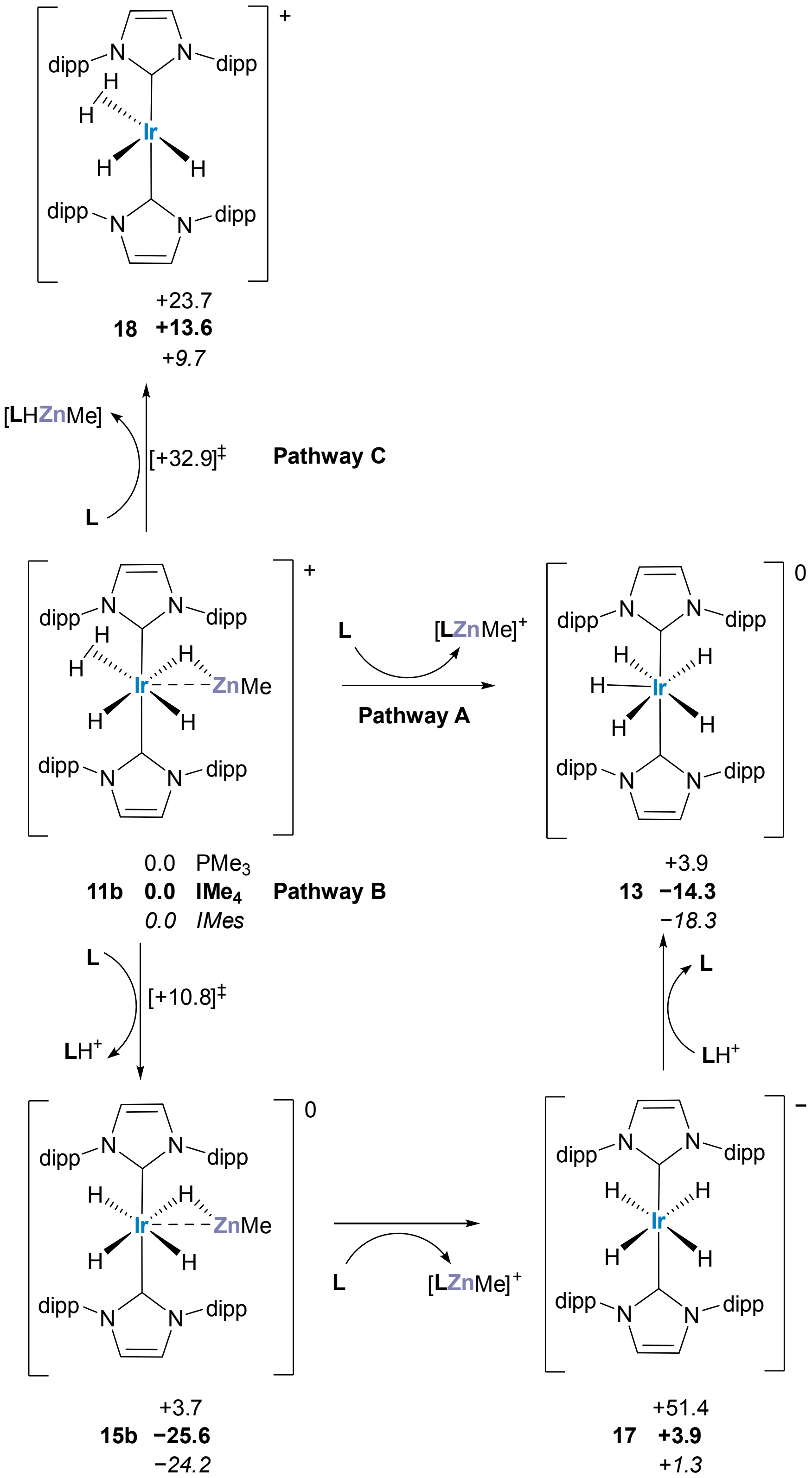

Figure S150. Possible pathways for the abstraction of $[ZnMe]^+$ from **11b** to form **13**. The free energies (in kcal/mol) are obtained at the BP86-D3(BJ)(PCM=THF)/def2-TZVP//BP86/BS1 level of theory and reported for PMe_3 (plain text), IMe_4 (bold) and IMes (italics).

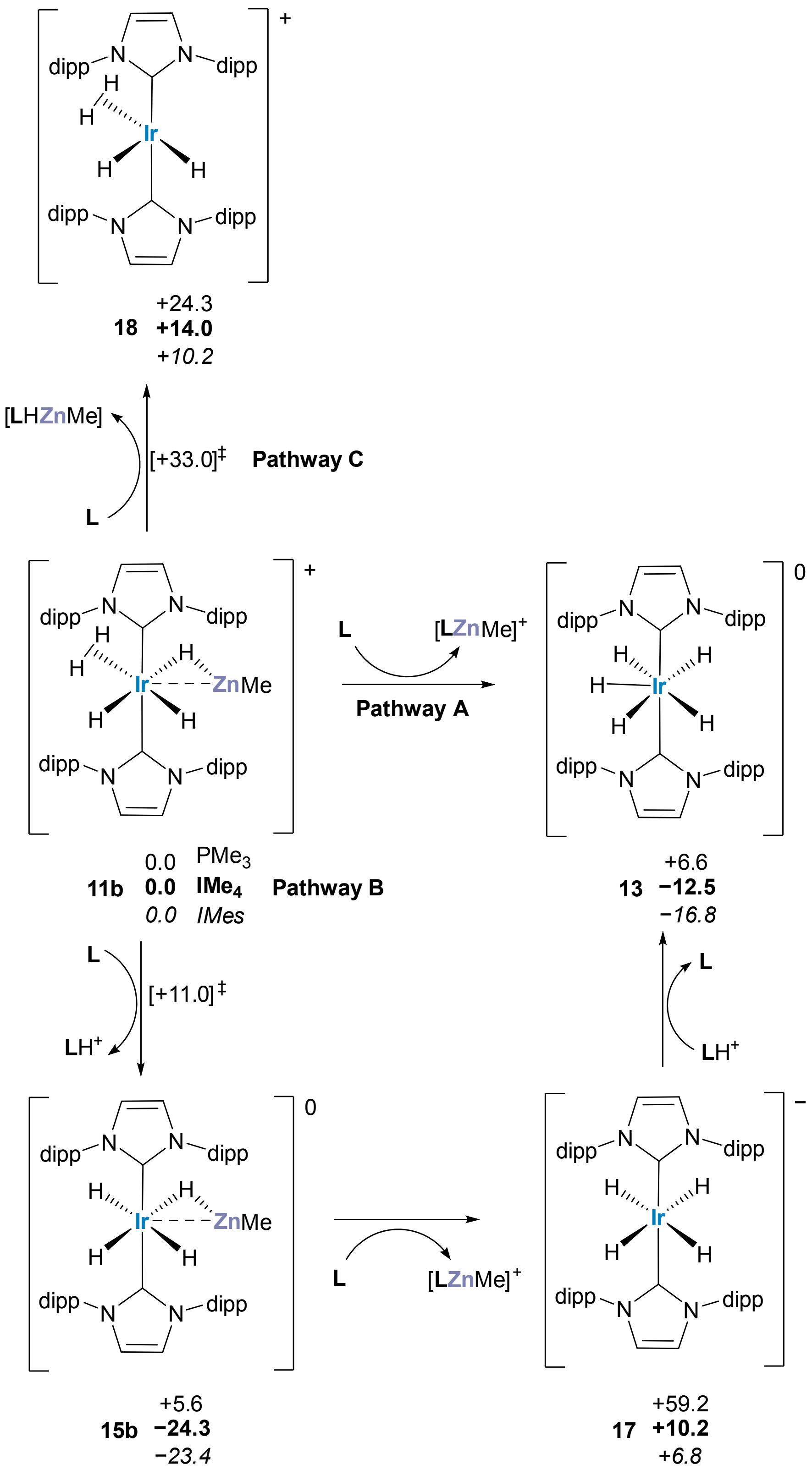

Figure S151. Possible pathways for the abstraction of $[ZnMe]^+$ from **11b** to form **13**. The free energies (in kcal/mol) are obtained at the BP86-D3(BJ)(PCM=C_6H_5F)/def2-TZVP//BP86/BS1 level of theory and reported for PMe_3 (plain text), IMe_4 (bold) and IMes (italics).

S2.3 Isomers of $[5]^+$

During our previous studies on $[Ir(IPr)_2(ZnMe)_2H_4][BAr^F{}_4]$,[3] the ZnMe analog of **5**, the interconversion between the experimentally observed 1,3-isomer and possible 1,2- and 1,4-isomers was studied, concluding that all hydrides were interchangeable. In contrast, the 1,4-isomer is the structure determined crystallographically for **5**. Another important difference between the ZnMe and ZnPh compounds is the relative disposition of the IPr ligands, being 'pseudo-staggered' for the ZnMe compound (IPr^IPr = 82°) and 'pseudo-eclipsed' for **5** (IPr^IPr = 0°). We thus explored the energetics of possible 1,2- and 1,3-isomers for this ZnPh compound. The ZnPh derivatives with staggered IPr ligands were built starting from the previously determined ZnMe isomers; for the derivatives with eclipsed IPr ligands the starting structure was taken from that determined crystallographically in this study. Different conformations of the ZnPh groups were also explored manually. The relative energies of the different isomers can be found in Table S3, and the structures are also provided. Surprisingly, we have found that a 1,3 disposition of the ZnPh groups appears more stable (computationally) than the 1,4-isomer obtained experimentally, regardless of the relative orientation of IPr ligands.

In order to verify the robustness of these results, we calculated the relative energies between the experimentally obtained 1,4-isomer and the computationally most stable 1,3-isomers with a broad set of different DFT functionals, namely PBE-D3(BJ),[28,29] BLYP-D3(BJ),[18,30] TPSS-D3(BJ),[31] B3LYP-D3(BJ),[18,30,32,33] B3PW91-D3(BJ),[18,32-35] PBE0-D3(BJ),[28,36] M06-L,[37] M06, M06-2X,[38] MN15,[39] PW6B95-D3(BJ),[40] B97-D,[41] B97-D3(BJ) and ωB97X-D,[42] always with the def2-TZVP basis set, and added the free energy and solvation corrections obtained at the same level of theory as the optimizations. The results can be found in Table S4. Notably, all functionals tested suggest the 1,3-isomer is more

stable, the smallest energy differences arising with the MN15, PW6B95-D3(BJ), M06-L and ωB97X-D functionals.

Isomer	Δ*G* (THF)	Δ*G* (C_6H_5F)	IPr^IPr (°)
1,3-stg	0.0	0.0	84.2
1,3-stg (C_2)	0.4	0.4	84.1
1,3-ecl	3.7	3.6	19.9
**1,4-ecl (C_i)**	**4.3**	**4.5**	**0.0**
1,4-stg	5.3	5.2	60.3
1,2-stg	5.7	5.9	70.2
1,2-stg (C_2)	5.8	6.1	70.4
1,3-stg'	8.7	8.5	89.0
1,3-stg' (C_2)	9.7	9.5	89.0
1,2-ecl	16.1	16.1	33.9
1,4-stg'	20.9	20.7	84.9
1,4-stg' (C_2)	21.8	21.6	83.8

Table S3. Relative free energies of different isomers of the cation of **5**. The isomers are labelled according to the relative distribution of H_4ZnPh as 1,2-; 1,3- or 1,4-; with the conformation of the IPr ligands also indicated as approximately eclipsed (ecl) or staggered (stg). For further clarity, the angle between the mean planes of the imidazole rings of the IPr ligands (denoted as IPr^IPr) is also reported. The experimentally determined isomer is highlighted in bold. The symmetry point group is denoted in parenthesis if different from C_1. The free energies have been obtained at the BP86-D3(BJ)(PCM = THF or C_6H_5F)/def2-TZVP//BP86/BS1 level. Energies in kcal/mol and angles in degrees.

	**1,3-stg**		**1,3-stg (C_2)**	
**Functional**	**Δ*G* (THF)**	**Δ*G* (C_6H_5F)**	**Δ*G* (THF)**	**Δ*G* (C_6H_5F)**
MN15	−1.4	−1.6	−1.0	−1.2
PW6B95-D3(BJ)	−1.4	−1.6	−1.1	−1.2
M06-L	−2.0	−2.2	−1.6	−1.8
ωB97X-D	−2.0	−2.2	−1.6	−1.8
PBE0-D3(BJ)	−2.6	−2.8	−2.2	−2.4
M06	−2.8	−2.9	−2.4	−2.5
PBE-D3(BJ)	−2.8	−3.0	−2.4	−2.6
TPSS-D3(BJ)	−3.0	−3.2	−2.6	−2.8
M06-2X	−3.3	−3.5	−2.9	−3.0
B3LYP-D3(BJ)	−3.3	−3.5	−2.9	−3.1
B97-D	−3.9	−4.1	−3.5	−3.6
B3PW91-D3(BJ)	−3.9	−4.1	−3.5	−3.7
BLYP-D3(BJ)	−4.0	−4.2	−3.6	−3.8
B97-D3(BJ)	−4.1	−4.3	−3.7	−3.9
BP86-D3(BJ)	−4.3	−4.5	−3.9	−4.1

Table S4. Relative free energy of the more stable 1,3-stg isomers with respect to the experimentally found 1,4-ecl (C_i) calculated with different functionals. Energies in kcal/mol.

S2.4. Alternative Isomers of [10]$^+$.

Two alternative isomers of the cation in [**10**]$^+$ have been located each as two distinct conformers with different relative orientations of ther NHC ligands. **[10b]**$^+$ has the Cd-Me unit adjacent to the alkene arm of the IPr′′ ligand and the more stable conformer is 6.5 kcal/mol above **[10]**$^+$ when computed in THF (6.4 kcal/mol higher in fluorobenzene). A dihydrogen hydride form, Ir(IPr)(IPr′′)(CdMe)(η^2-H_2)H, **[10c]**$^+$, lies 11.5 kcal/mol above **[10]**$^+$ in THF and 11.4 kcal/mol higher in fluorobenzene. The more stable form of each are shown in Figure S152.

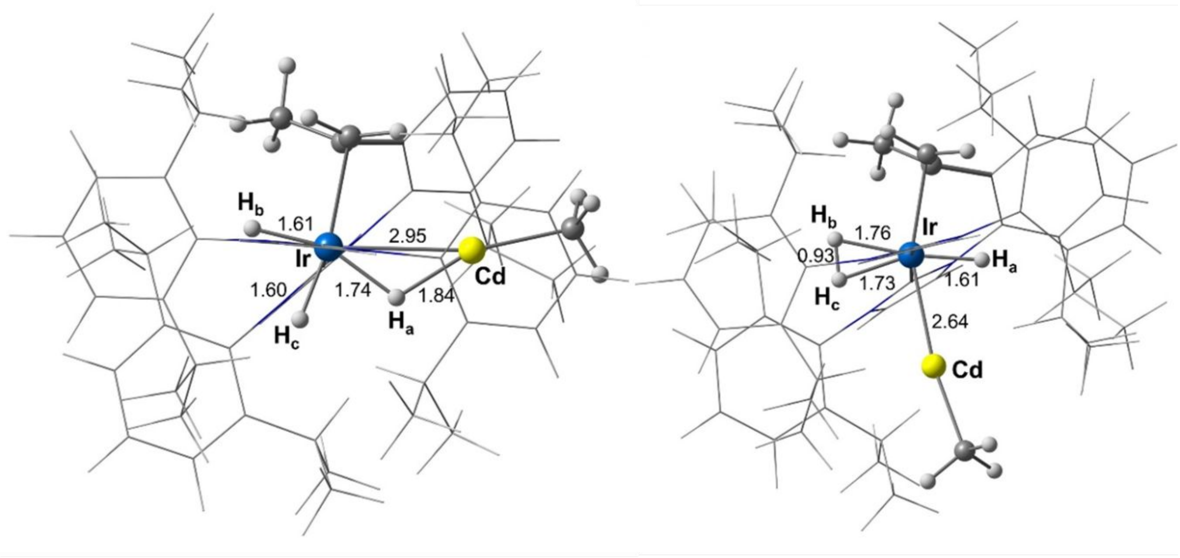

[10b]$^+$
+6.5 (THF)
+6.4 (fluorobenzene)

[10c]$^+$
+11.5 (THF)
+11.4 (fluorobenzene)

Figure S152. Computed structures of alternative isomers of the cation of **[10]**$^+$ with selected distances in Å and free energies are quoted relative to **[10]**$^+$ set to 0.0 kcal./mol (method: BP86-D3(BJ)(PCM = THF or C_6H_5F)/def2-TZVP//BP86/BS1; axial ligands shown in wireframe for clarity).

S2.5 Functional testing on alternative structures of 13 and 15b

Additional calculations with a range of functionals were run to test for $Ir(IPr)_2(\eta^2\text{-}H_2)H_3$ as an alternative isomer of $Ir(IPr)_2H_5$, **13**. With the standard BP86/BS1 protocol no local minimum structure was found, but a transition state for H/H exchange was located featuring an intact η^2-H_2 moiety oriented parallel to the C_{IPr}-Ir-C_{IPr} direction that linked directly to pentahydride structures. Equivalent behavior was seen when optimizations were performed with PBE as well as TPSS, and the same transition state was located with B3LYP and PBE0. In addition, both hybrid functionals an $Ir(IPr)_2(\eta^2\text{-}H_2)H_3$ form with an in-plane η^2-H_2 ligand was also located as a local minimum. The energies of these different stationary points are summarized in Table S5 which show that all calculations support a pentahydride as the global minimum with low barriers for H/H exchange.

Table S5. Computed free energies (kcal/mol) for alternative stationary points computed for **13** with different functionals.[a]

		$Ir(IPr)_2H_5$	$Ir(IPr)_2(\eta^2\text{-}H_2)H_3$	H/H exchange TS
BP86	THF	0.0	No minimum	+7.9[b]
	Fluorobenzene	0.0	located	+8.1
PBE	THF	0.0	No minimum	+7.3
	Fluorobenzene	0.0	located	+7.5
PBE0	THF	0.0	+1.6	+6.6
	Fluorobenzene	0.0	+1.8	+7.0
B3LYP	THF	0.0[b]	+3.0[b]	+7.0[b]
	Fluorobenzene	0.0	+3.1	+7.2
TPSS	THF	0.0	+2.9	+6.6
	Fluorobenzene	0.0	+3.1	+6.8

[a] XC-D3(BJ)(PCM=solvent)/def2-TZVP//XC/BS1, where XC is the functional of choice and solvent is either THF or fluorobenzene. [b]Structures included in Section S2.8.

For **15b**, a similar situation is seen, with an H/H exchange transition state featuring an η^2-H_2 moiety lying parallel to the C_{IPr}-Ir-C_{IPr} direction being located with all functionals; in this case, an η^2-H_2 minimum, $Ir(IPr)_2(ZnMe)(\eta^2\text{-}H_2)H_2$, was only located with B3LYP. The energetics of these species are summarized in Table S6, which shows that the tetrahydride

structure is clearly favored across a range of functionals. The much higher barriers for H/H exchange are also consistent with a lower degree of fluxionality in solution seen experimentally.

Table S6. Computed free energies (kcal/mol) for alternative stationary points computed for **15b** with different functionals.[a]

		$Ir(IPr)_2(ZnMe)H_4$	$Ir(IPr)_2(ZnMe)(\eta^2\text{-}H_2)H_2$	H/H exchange TS
BP86	THF	0.0	No minimum	+20.4[b]
	Fluorobenzene	0.0	located	+20.4
PBE	THF	0.0	No minimum	+20.2
	Fluorobenzene	0.0	located	+20.1
PBE0	THF	0.0	No minimum	+25.3
	Fluorobenzene	0.0	located	+25.3
B3LYP	THF	0.0[b]	+14.2[b]	+22.2[b]
	Fluorobenzene	0.0	+14.1	+22.1
TPSS	THF	0.0	No minimum	+23.3
	Fluorobenzene	0.0	located	+23.3

[a] XC-D3(BJ)(PCM=solvent)/def2-TZVP//XC/BS1 (XC: selected functional; solvent: THF or fluorobenzene). [b]Structures included in Section S2.8.

S2.6 Effect of solvent on the computed structure of 13

Geometries of **13** were reoptimized with BP86/BS1 and B3LYP/BS1 both with and without the effects of THF and fluorobenzene solvent included in the optimisation protocol (see Table S7). For each solvent and functional combination, three sets of free energies are provided: (i) the result derived from a gas-phase optimization to which the PCM solvent, dispersion and basis set corrections have been added; (ii) as for (i) but with an SMD[43] solvent correction; and (iii) optimization using SMD solvation with energies then including SMD solvent, dispersion and basis set corrections. SMD was adopted as we found in many cases optimizations with PCM solvent corrections failed to converge.

Table S7. Computed free energies (kcal/mol) for stationary points computed for **13** with variation in functional, solvent and inclusion of solvent included in the optimization.

			$Ir(IPr)_2H_5$	$Ir(IPr)_2(\eta^2\text{-}H_2)H_3$	H/H exchange TS
THF	BP86	Gas(PCM)	0.0	No minimum	+7.9
		Gas(SMD)	0.0	No minimum	+5.1
		Opt(SMD)	0.0	No minimum	+9.5
	B3LYP	Gas(PCM)	0.0	+3.0	+7.0
		Gas(SMD)	0.0	+1.8	+3.7
		Opt(SMD)	0.0	+1.3	+5.4[a]
Fluorobenzene	BP86	Gas(PCM)	0.0	No minimum	+8.1
		Gas(SMD)	0.0	No minimum	+5.4
		Opt(SMD)	0.0	No minimum	+8.2
	B3LYP	Gas(PCM)	0.0	+3.1	+7.2
		Gas(SMD)	0.0	+2.0	+4.1
		Opt(SMD)	0.0	+0.3	+7.4

[a] corresponds to a local minimum

In all cases, the pentahydride structure is the global minimum. With B3LYP a $Ir(IPr)_2(\eta^2\text{-}H_2)H_3$ isomer is located whether solvation is included within the optimization protocol or not and this form becomes close in energy to the pentahydride global minimum, especially when optimized with fluorobenzene. All calculations suggest a pentahydride global minimum that is undergoing rapid H/H exchange.

S2.7 QTAIM analyses

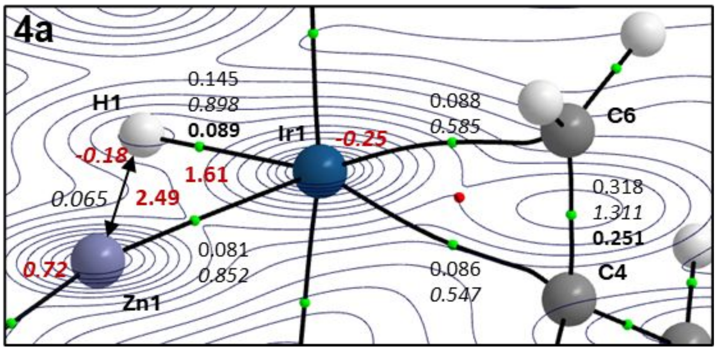

Atom Pair	Distance (Å)	$\rho(\mathbf{r})$	$\nabla^2\rho(\mathbf{r})$	ε	H($\mathbf{r}$)	δ(A,B)
Ir1–H1	1.622	0.145	0.083	0.089	−0.077	0.898
Ir1–Zn1	2.352	0.081	0.171	0.024	−0.026	0.852
Ir1–C4	2.248	0.088	0.158	0.325	−0.029	0.585
Ir1–C6	2.221	0.086	0.150	0.462	−0.027	0.547
C4–C6	1.377	0.318	−0.854	0.251	−0.329	1.311
Zn1–H1	2.486	-	-	-	-	0.065

Figure S153. QTAIM molecular graph (optimized H atom positions) with selected BCP metrics (in a.u.) tabulated for the cation of **4a**. Density contours in the {Zn1Ir1H1} plane, showing computed Ir–H and Zn–H distances (Å, in red, plain text) and QTAIM atomic charges (in red, italics). BCPs (green spheres) show the associated ρ(r) in plain text, delocalization indices in italics and, for bond paths to hydrogens, ellipticities in bold. Zn1···H1 delocalization indices are also indicated.

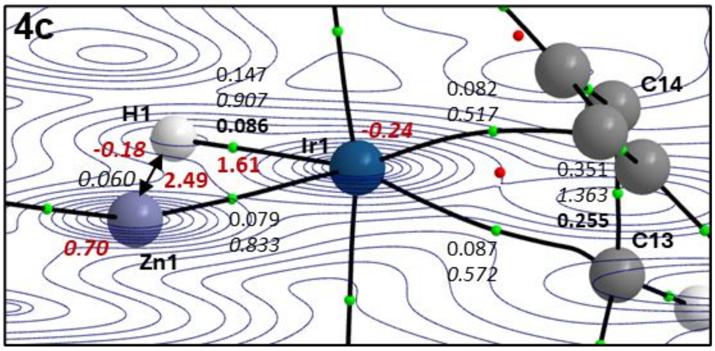

Atom Pair	Distance (Å)	$\rho(\mathbf{r})$	$\nabla^2\rho(\mathbf{r})$	ε	H($\mathbf{r}$)	δ(A,B)
Ir1−H1	1.618	0.147	0.081	0.086	−0.078	0.907
Ir1−Zn1	2.366	0.079	0.165	0.026	−0.025	0.833
Ir1−C13	2.224	0.087	0.168	0.359	−0.028	0.572
Ir1−C14	2.268	0.082	0.168	0.767	−0.024	0.517
C13−C14	1.326	0.351	−1.069	0.255	−0.404	1.363
Zn1−H1	2.516					0.060

Figure S154. QTAIM molecular graph (optimized H atom positions) with selected BCP metrics (in a.u.) tabulated for the cation of **4c**. Density contours in the {Zn1Ir1H1} plane, showing computed Ir−H and Zn−H distances (Å, in red, plain text) and QTAIM atomic charges (in red, italics). BCPs (green spheres) show the associated ρ(r) in plain text, delocalization indices in italics and, for bond paths to hydrogens, ellipticities in bold. Zn1···H1 delocalization indices are also indicated.

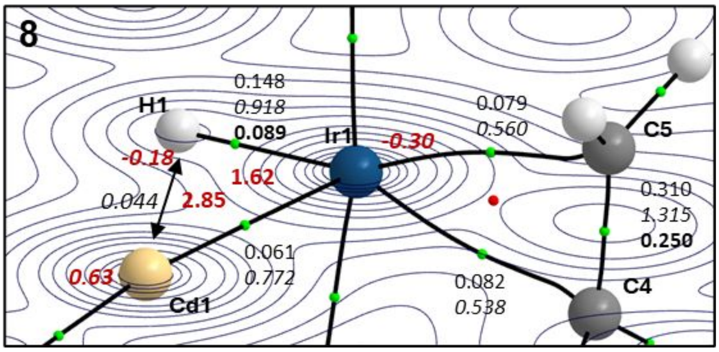

Atom Pair	Distance (Å)	$\rho(\mathbf{r})$	$\nabla^2\rho(\mathbf{r})$	ε	H($\mathbf{r}$)	δ(A,B)
Ir1–H1	1.616	0.148	0.075	0.089	−0.079	0.918
Ir1–Cd1	2.650	0.061	0.101	0.027	−0.016	0.772
Ir1–C4	2.271	0.082	0.137	0.250	−0.025	0.538
Ir1–C5	2.276	0.079	0.152	0.368	−0.023	0.560
C4–C5	1.390	0.310	−0.808	0.250	−0.312	1.315
Cd1–H1	2.853	-	-	-	-	0.044

Figure S155. QTAIM molecular graph (optimized H atom positions) with selected BCP metrics (in a.u.) tabulated for the cation of **8**. Density contours in the {Cd1Ir1H1} plane, showing computed Ir–H and Cd–H distances (Å, in red, plain text) and QTAIM atomic charges (in red, italics). BCPs (green spheres) show the associated ρ(r) in plain text, delocalization indices in italics and, for bond paths to hydrogens, ellipticities in bold. Cd1···H1 delocalization indices are also indicated.

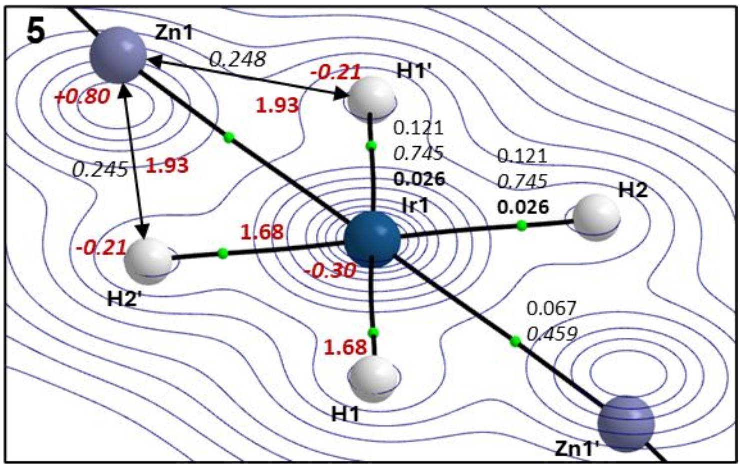

Atom Pair	Distance (Å)	$\rho(\mathbf{r})$	$\nabla^2\rho(\mathbf{r})$	ε	H($\mathbf{r}$)	δ(A,B)
Ir1–H1 (= Ir1–H1′)	1.684	0.121	0.147	0.026	−0.056	0.745
Ir1–H2 (= Ir1–H2′)	1.684	0.121	0.147	0.026	−0.056	0.745
Ir1–Zn1 (= Ir1–Zn1′)	2.461	0.067	0.115	0.527	−0.020	0.459
Zn1–H1′ (= Zn1′–H1)	1.936	-	-	-	-	0.248
Zn1–H2′ (= Zn1′–H2)	1.937	-	-	-	-	0.245

Figure S156. QTAIM molecular graph (optimized H atom positions) with selected BCP metrics (in a.u.) tabulated for the cation of **5**. Density contours in the {H1Ir1H2} plane, showing computed Ir−H and Zn−H distances (Å, in red, plain text) and QTAIM atomic charges (in red, italics). BCPs (green spheres) show the associated ρ(r) in plain text, delocalization indices in italics and, for bond paths to hydrogens, ellipticities in bold. Zn1···H1′/H2 delocalization indices also indicated.

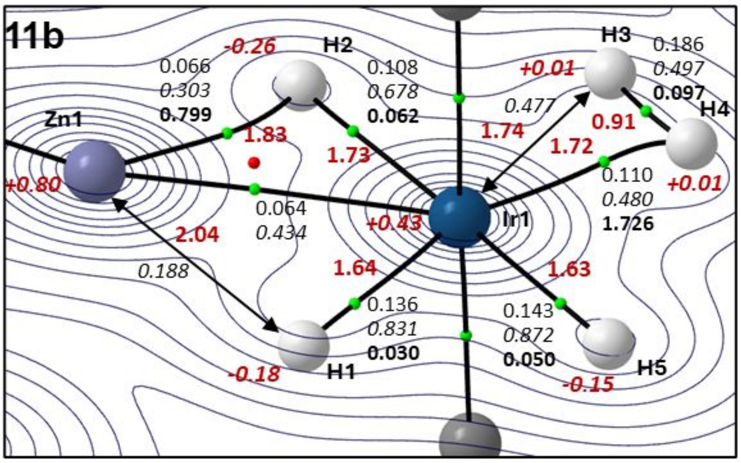

Atom Pair	Distance (Å)	$\rho(\mathbf{r})$	$\nabla^2\rho(\mathbf{r})$	ε	H($\mathbf{r}$)	δ(A,B)
Ir1–H1	1.637	0.136	0.124	0.030	−0.068	0.831
Ir1–H2	1.732	0.108	0.169	0.062	−0.045	0.678
Ir1–H3	1.738	-	-	-	-	0.477
Ir1–H4	1.724	0.110	0.303	1.726	−0.039	0.480
Ir1–H5	1.629	0.143	0.081	0.050	−0.075	0.872
H3–H4	0.908	0.186	−0.468	0.097	−0.145	0.497
Ir1–Zn1	2.477	0.064	0.115	0.877	−0.019	0.434
Zn1–H1	2.037	-	-	-	-	0.188
Zn1–H2	1.830	0.066	0.114	0.799	−0.017	0.303

Figure S157. QTAIM molecular graph (optimized H atom positions) with selected BCP metrics (in a.u.) tabulated for the cation of **11b**. Density contours in the {Zn1Ir1H2} plane, showing computed Ir−H and Zn−H distances (Å, in red, plain text) and QTAIM atomic charges (in red, italics). BCPs (green spheres) show the associated ρ(r) in plain text, delocalization indices in italics and, for bond paths to hydrogens, ellipticities in bold. Zn1···H1 delocalization indices are also indicated.

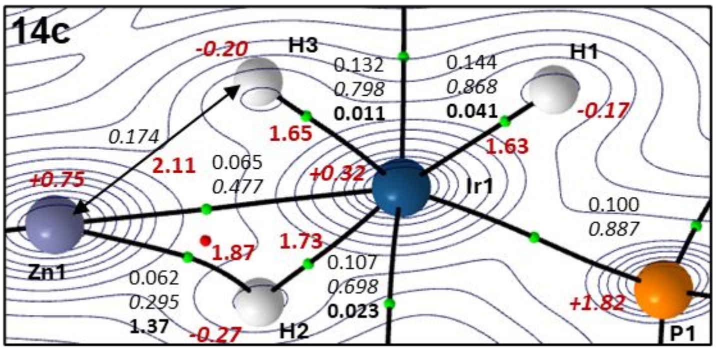

Atom Pair	Distance (Å)	$\rho(\mathbf{r})$	$\nabla^2\rho(\mathbf{r})$	ε	H($\mathbf{r}$)	δ(A,B)
Ir1−H1	1.625	0.144	0.093	0.041	−0.076	0.868
Ir1−H2	1.732	0.107	0.169	0.023	−0.045	0.698
Ir1−H3	1.654	0.132	0.115	0.011	−0.065	0.798
Ir1−Zn1	2.480	0.065	0.107	0.407	−0.020	0.477
Ir1−P1	2.350	0.100	0.114	0.075	−0.041	0.887
Zn1−H2	1.874	0.062	0.096	1.366	−0.016	0.295
Zn1−H3	2.113	-	-	-	-	0.174

Figure S158. QTAIM molecular graph (optimized H atom positions) with selected BCP metrics (in a.u.) tabulated for the cation of **14c**. Density contours in the {Zn1Ir1H2} plane, showing computed Ir−H and Zn−H distances (Å, in red, plain text) and QTAIM atomic charges (in red, italics). BCPs (green spheres) show the associated ρ(r) in plain text, delocalization indices in italics and, for bond paths to hydrogens, ellipticities in bold. Zn1···H3 delocalization indices are also indicated.

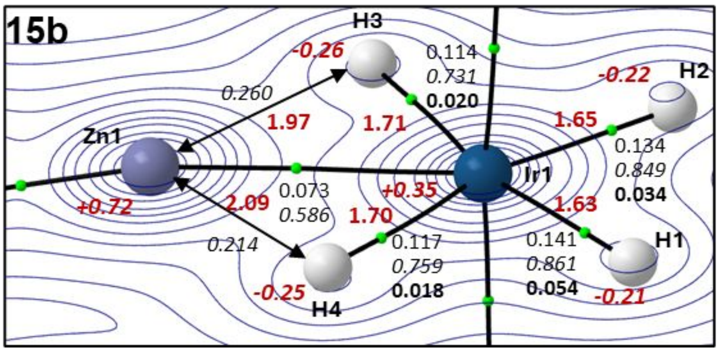

Atom Pair	Distance (Å)	$\rho(\mathbf{r})$	$\nabla^2\rho(\mathbf{r})$	ε	H($\mathbf{r}$)	δ(A,B)
Ir1–H1	1.633	0.141	0.100	0.054	−0.072	0.861
Ir1–H2	1.650	0.134	0.101	0.034	−0.066	0.849
Ir1–H3	1.709	0.114	0.151	0.020	−0.050	0.731
Ir1–H4	1.700	0.117	0.134	0.018	−0.052	0.759
Ir1–Zn1	2.418	0.073	0.121	0.050	−0.024	0.586
Zn1–H3	1.969	-	-	-	-	0.260
Zn1–H4	2.085	-	-	-	-	0.214

Figure S159. QTAIM molecular graph (optimized H atom positions) with selected BCP metrics (in a.u.) tabulated for **15b**. Density contours in the {Zn1Ir1H3} plane, showing computed Ir−H and Zn−H distances (Å, in red, plain text) and QTAIM atomic charges (in red, italics). BCPs (green spheres) show the associated ρ(r) in plain text, delocalization indices in italics and, for bond paths to hydrogens, ellipticities in bold. Zn1···H3/H4 delocalization indices are also indicated.

S2.8 Cartesian coordinates (Å) and energies (a.u.) for the computed structures.

(i) Structures used in QTAIM studies (heavy atoms fixed at X-ray positions; H atoms optimized).

[4a]+

```
142
SCF = -2882.77753445
Ir  0.39090 -0.00452 -0.38862
H   0.51919  0.37548  1.18294
Zn -1.73944 -0.42199  0.51711
N  -1.37145  2.27434 -1.47491
N   0.01524  3.08519 -0.04405
N   0.63326 -2.95143  0.62834
N   2.54231 -2.18322  0.04342
C  -0.38174  1.89905 -0.60046
C  -2.04051  1.39488 -2.38834
C  -1.24636  0.40836 -2.99195
C   0.18494  0.23179 -2.61433
C   1.12341  1.27034 -3.20350
H   0.77934  2.30196 -3.02881
H   1.17132  1.13111 -4.30057
H   2.14573  1.17191 -2.80410
C   0.64469 -1.03672 -2.33899
H  -0.02748 -1.89739 -2.38884
H   1.70162 -1.26157 -2.49086
C  -1.80637 -0.38871 -3.98498
H  -1.18164 -1.13648 -4.48404
C  -3.13244 -0.21674 -4.34970
H  -3.56452 -0.83247 -5.14607
C  -3.91096  0.71603 -3.70280
H  -4.96566  0.81889 -3.97783
C  -3.39772  1.53826 -2.69008
C  -4.35191  2.51533 -2.00749
H  -3.81659  3.03082 -1.19178
C  -4.85875  3.55749 -3.00110
H  -4.04283  4.12191 -3.48912
H  -5.51870  4.28663 -2.50003
H  -5.44480  3.08651 -3.80925
C  -5.53387  1.78968 -1.36570
H  -6.15665  1.27636 -2.11948
H  -6.18418  2.51575 -0.84771
H  -5.20560  1.04164 -0.63060
C  -1.52333  3.65638 -1.50677
H  -2.20386  4.14916 -2.19341
C  -0.66249  4.15653 -0.61384
H  -0.45161  5.18003 -0.31712
C   1.04530  3.35468  0.94053
C   2.28625  3.80345  0.47575
C   2.62395  3.92485 -0.99706
H   1.75498  3.58049 -1.58144
C   2.89080  5.38527 -1.38908
H   2.03246  6.03795 -1.15475
H   3.09004  5.46476 -2.47181
H   3.76964  5.79384 -0.86052
C   3.80687  3.02351 -1.36747
H   4.72746  3.32794 -0.83940
H   4.01586  3.08210 -2.45032
H   3.61064  1.97188 -1.10553
C   3.22469  4.20072  1.44209
H   4.20735  4.55992  1.11847
C   2.91449  4.15762  2.78061
H   3.65811  4.47985  3.51881
C   1.67168  3.72862  3.21443
H   1.44817  3.71733  4.28452
C   0.69873  3.32534  2.29835
C  -0.68778  2.93044  2.76701
H  -1.09172  2.20293  2.03801
C  -1.61819  4.13912  2.80438
H  -1.23984  4.90130  3.50859
H  -2.62788  3.84541  3.14190
H  -1.72781  4.62066  1.81838
C  -0.67362  2.25926  4.13591
H   0.05420  1.43338  4.18392
H  -1.67013  1.84867  4.37268
H  -0.43169  2.97400  4.94217
C  -3.56216 -0.29158  1.16168
C  -3.89998  0.64733  2.11703
H  -3.13222  1.30130  2.55117
C  -5.21404  0.80540  2.56276
H  -5.43927  1.56084  3.32466
C  -6.21230  0.01544  2.04835
H  -7.24530  0.13742  2.39448
C  -5.90885 -0.91529  1.09291
H  -6.69834 -1.54619  0.66659
C  -4.60192 -1.07475  0.65632
H  -4.39755 -1.83016 -0.11118
C   1.22684 -1.82282  0.16013
C  -0.73852 -3.17786  1.02236
C  -1.58772 -3.84104  0.12500
C  -1.17939 -4.20343 -1.28808
H  -0.19989 -3.73523 -1.49074
C  -2.18285 -3.65534 -2.30757
H  -3.17704 -4.12123 -2.19254
```

H	-1.84563	-3.86986	-3.33709
H	-2.31215	-2.56396	-2.21278
C	-0.99614	-5.71829	-1.43386
H	-0.23991	-6.10943	-0.73236
H	-0.67076	-5.97476	-2.45706
H	-1.93914	-6.25876	-1.23973
C	-2.84775	-4.22681	0.60257
H	-3.53136	-4.75823	-0.06822
C	-3.22832	-3.95798	1.89200
H	-4.21789	-4.26983	2.24363
C	-2.37836	-3.30310	2.75339
H	-2.70494	-3.09615	3.77565
C	-1.10224	-2.89830	2.34451
C	-0.16048	-2.23733	3.32801
H	0.52544	-1.58348	2.75852
C	0.66146	-3.27690	4.07329
H	0.00479	-3.96347	4.63711
H	1.33531	-2.79189	4.80143
H	1.28757	-3.88920	3.40363
C	-0.89623	-1.37410	4.33937
H	-1.59006	-0.66231	3.86175
H	-0.17397	-0.79665	4.94063
H	-1.47927	-1.98090	5.05434
C	1.55113	-3.98902	0.78441
H	1.24617	-4.96726	1.14430
C	2.74136	-3.50285	0.42817
H	3.72985	-3.95277	0.42473
C	3.65654	-1.30987	-0.25865
C	4.13618	-0.49558	0.77295
C	3.57629	-0.57087	2.18430
H	2.62096	-1.11969	2.15661
C	3.31250	0.78246	2.79931
H	4.23859	1.36824	2.93257
H	2.86460	0.66695	3.80185
H	2.62066	1.37838	2.18607
C	4.54202	-1.34195	3.09124
H	4.74867	-2.36160	2.72133
H	4.12655	-1.43242	4.10993
H	5.51108	-0.81849	3.17238
C	5.23951	0.30623	0.51174
H	5.63563	0.95186	1.30202
C	5.84233	0.27977	-0.72185
H	6.71119	0.91769	-0.91990
C	5.39227	-0.57445	-1.70687
H	5.91775	-0.61196	-2.66656
C	4.29053	-1.41424	-1.49806
C	3.89906	-2.43511	-2.54233
H	2.89309	-2.82026	-2.29676
C	4.86048	-3.63367	-2.50223
H	5.89118	-3.31885	-2.74189
H	4.56149	-4.39680	-3.24155
H	4.88818	-4.11829	-1.51233
C	3.86044	-1.85284	-3.95802
H	3.22473	-0.95435	-4.02901
H	3.47085	-2.60126	-4.66933
H	4.86775	-1.57237	-4.31061

[4c]$^+$

138

SCF = -2730.34146703

N	-1.93676	2.31701	-0.97046
N	-0.25230	3.09520	0.10828
N	2.34647	-2.05957	-0.34848
N	0.55920	-2.94548	0.41121
C	-0.75522	1.92798	-0.36084
C	-1.04853	4.17748	-0.24947
H	-0.77839	5.19310	0.02415
C	-2.08419	3.69036	-0.92809
H	-2.90918	4.20070	-1.41198
C	-2.81389	1.43780	-1.68655
C	-4.20111	1.56746	-1.59312
C	-6.06698	1.95760	0.07616
H	-6.87225	1.58442	-0.57896
H	-5.73367	1.12293	0.71057
H	-6.52055	2.72011	0.73273
C	-4.91768	2.57489	-0.71482
H	-4.19944	2.98469	0.01722
C	-5.46039	3.71924	-1.57479
H	-6.24287	3.34737	-2.25938
H	-5.91642	4.50463	-0.94752
H	-4.69603	4.19658	-2.21292
C	-4.94407	0.69512	-2.38022
H	-6.03621	0.77292	-2.35085
C	-4.37566	-0.24472	-3.18956
H	-5.00678	-0.89823	-3.80083
C	-3.00503	-0.37627	-3.22440
H	-2.53344	-1.12670	-3.86713
C	-2.20003	0.46677	-2.46886
C	-0.67340	0.32620	-2.53123
C	-0.12644	-0.87641	-2.41811
H	0.86936	-1.05862	-2.83503
H	-0.74071	-1.77572	-2.31577
C	-0.01442	1.42524	-3.28190
H	1.08419	1.38346	-3.19909
H	-0.26819	1.33549	-4.35636
H	-0.34159	2.42902	-2.96862
C	0.94428	3.34462	0.88335
C	0.83018	3.40728	2.24940

C	-0.46067	3.07917	2.98774
H	-1.15865	2.62554	2.26097
C	-1.10706	4.31667	3.55338
H	-1.34438	5.06332	2.77524
H	-2.05017	4.06763	4.07105
H	-0.45115	4.81339	4.29075
C	-0.23162	2.06281	4.08942
H	0.38401	2.47548	4.90864
H	-1.19177	1.75224	4.53707
H	0.27800	1.16363	3.71022
C	1.94763	3.82783	2.95643
H	1.88523	3.89754	4.04835
C	3.11398	4.18006	2.32773
H	3.97449	4.52424	2.91084
C	3.19152	4.09982	0.95927
H	4.12095	4.38834	0.45509
C	2.10627	3.69136	0.18022
C	2.19817	3.70601	-1.31883
H	1.20478	3.45465	-1.72676
C	3.17869	2.66085	-1.81443
H	2.90710	1.64734	-1.47313
H	3.22401	2.65569	-2.91819
H	4.19976	2.86370	-1.44910
C	2.57993	5.07998	-1.86123
H	3.59302	5.37994	-1.54130
H	2.57601	5.07256	-2.96487
H	1.88304	5.86822	-1.52806
C	-3.45663	-0.38545	1.96701
H	-3.21676	-0.71929	2.99245
H	-3.70456	0.69168	2.03608
C	-4.62340	-1.18402	1.39225
H	-4.41851	-2.26543	1.40279
H	-5.55273	-1.02659	1.97476
H	-4.84823	-0.91048	0.34673
C	1.03931	-1.76760	-0.06357
C	-0.70205	-3.24605	1.06564
C	-1.69874	-3.90542	0.31854
C	-1.60140	-4.16460	-1.15034
H	-0.74867	-3.58406	-1.54556
C	-1.31421	-5.63251	-1.41643
H	-2.12841	-6.27559	-1.03828
H	-1.21801	-5.82443	-2.49938
H	-0.37984	-5.96916	-0.93553
C	-2.85915	-3.72703	-1.88878
H	-3.10835	-2.67045	-1.69643
H	-2.72971	-3.85411	-2.97800
H	-3.73520	-4.33369	-1.60130
C	-2.79145	-4.39036	1.03201
H	-3.58404	-4.91819	0.49084
C	-2.88922	-4.24724	2.39068
H	-3.75565	-4.65445	2.92301
C	-1.89640	-3.60043	3.08891
H	-1.97971	-3.49858	4.17506
C	-0.77756	-3.09790	2.44413
C	0.32301	-2.46500	3.26693
H	0.95904	-1.86888	2.58952
C	-0.20833	-1.52771	4.33996
H	-0.73273	-2.07314	5.14429
H	0.62509	-0.98738	4.81963
H	-0.90837	-0.77990	3.93402
C	1.17989	-3.53387	3.92203
H	1.65411	-4.20666	3.18796
H	1.98717	-3.07736	4.52105
H	0.57672	-4.15953	4.60394
C	1.52924	-3.93704	0.40493
H	1.31738	-4.94311	0.75413
C	2.64265	-3.37553	-0.06520
H	3.64086	-3.77786	-0.21154
C	3.42925	-1.14829	-0.70462
C	3.89987	-1.15456	-2.01059
C	5.02869	-0.36289	-2.26676
H	5.43299	-0.32642	-3.28347
C	5.64575	0.33406	-1.27117
H	6.53572	0.93209	-1.49836
C	5.18344	0.26849	0.02563
H	5.71272	0.80341	0.82066
C	4.05840	-0.48889	0.34247
C	3.64361	-0.63008	1.80184
H	2.67366	-1.15402	1.82952
C	4.64879	-1.46764	2.57378
H	5.64236	-0.98536	2.59637
H	4.32398	-1.59768	3.62090
H	4.78270	-2.47317	2.13841
C	3.44579	0.71188	2.47783
H	2.70011	1.32396	1.94902
H	3.09740	0.57015	3.51602
H	4.38323	1.29210	2.53286
C	3.30537	-2.01139	-3.11635
H	2.31581	-2.36773	-2.77913
C	4.17827	-3.25279	-3.34277
H	4.27649	-3.86780	-2.43340
H	3.74888	-3.89238	-4.13355
H	5.19624	-2.96649	-3.66003
C	3.13358	-1.24522	-4.41100
H	4.10322	-0.96089	-4.85449
H	2.61335	-1.86455	-5.16236
H	2.55089	-0.31767	-4.27768
H	0.54020	0.38961	1.10341

Ir 0.06711 0.04913 -0.40554
Zn -1.82068 -0.46179 0.92599

[8]$^+$
135
SCF = -2631.64000559
Ir 0.11862 0.08349 -0.41030
Cd -2.15269 -0.53098 0.80827
N -1.82239 2.38476 -1.02779
N -0.13626 3.13572 0.06257
N 0.45781 -2.91831 0.47010
N 2.29821 -2.15116 -0.31647
C -0.66333 1.98253 -0.41034
C -2.71208 1.50720 -1.72625
C -2.12104 0.50239 -2.50038
C -0.62572 0.35573 -2.53873
C -0.08271 -0.92014 -2.44266
H -0.72681 -1.79522 -2.33161
H 0.90660 -1.11067 -2.86588
C 0.07313 1.41478 -3.32772
H -0.21499 2.43719 -3.03552
H -0.19075 1.30814 -4.39786
H 1.16919 1.33236 -3.24639
C -2.93969 -0.33466 -3.23968
H -2.48613 -1.10298 -3.87322
C -4.30973 -0.17386 -3.19879
H -4.95116 -0.82523 -3.80263
C -4.87679 0.79070 -2.40440
H -5.96617 0.88801 -2.37085
C -4.09546 1.65523 -1.62477
C -4.79640 2.67516 -0.73801
H -4.05904 3.08139 -0.02254
C -5.93002 2.06963 0.08265
H -6.76666 1.72898 -0.55114
H -6.34177 2.82859 0.76992
H -5.59507 1.21281 0.68634
C -5.34959 3.83164 -1.57882
H -4.59388 4.31177 -2.22477
H -5.78907 4.61171 -0.93384
H -6.14689 3.47139 -2.25247
C -1.95390 3.76786 -1.00305
H -2.76614 4.28624 -1.49916
C -0.89957 4.23915 -0.31201
H -0.60696 5.25004 -0.04444
C 1.07722 3.36112 0.83770
C 0.95205 3.50355 2.21275
C -0.33969 3.29935 2.94790
H -1.09209 2.91058 2.23919
C -0.16945 2.24393 4.06216
H 0.24744 1.30950 3.65751
H -1.14179 2.02004 4.53413
H 0.50635 2.60270 4.85767
C 2.11510 3.88739 2.90321
H 2.06643 4.01899 3.98944
C 3.29180 4.08898 2.25119
H 4.17768 4.39286 2.82125
C 3.38513 3.96190 0.87732
H 4.33442 4.16424 0.37151
C 2.26850 3.58147 0.12801
C 2.38331 3.57551 -1.39060
H 1.38567 3.34284 -1.80076
C 3.33462 2.52247 -1.85387
H 4.35902 2.70522 -1.48521
H 3.39484 2.49752 -2.95709
H 3.04666 1.51484 -1.50627
C 2.80045 4.93975 -1.97274
H 2.11853 5.75101 -1.66618
H 2.79973 4.89309 -3.07492
H 3.81863 5.22270 -1.65397
C 1.00551 -1.76681 -0.04747
C -0.81793 -3.15346 1.12331
C -0.89620 -2.96458 2.51600
C 0.23563 -2.37730 3.32304
H 0.90695 -1.83319 2.63731
C -0.28061 -1.36867 4.36719
H -0.94519 -0.61287 3.91839
H 0.56679 -0.84290 4.83781
H -0.83654 -1.86801 5.17925
C 1.02800 -3.46187 4.03773
H 0.38319 -4.03044 4.73087
H 1.84514 -3.01907 4.63293
H 1.48184 -4.18394 3.33824
C -2.06258 -3.39220 3.16097
H -2.14710 -3.26245 4.24403
C -3.09292 -3.99116 2.46660
H -3.98782 -4.33075 2.99888
C -0.85449 4.60382 3.52913
H -0.13923 5.03307 4.25240
H -1.80825 4.44865 4.06317
H -1.02902 5.36615 2.75006
C -2.99298 -4.16662 1.10266
H -3.81043 -4.64984 0.55884
C -1.85254 -3.76541 0.40070
C -1.74517 -4.06437 -1.07603
H -0.87161 -3.52270 -1.47564
C -1.49513 -5.56695 -1.27896
H -0.57489 -5.90542 -0.77400
H -1.39501 -5.80491 -2.35215

H -2.33301 -6.16494 -0.88067
C -2.99145 -3.62789 -1.84629
H -3.88112 -4.20965 -1.55073
H -2.84960 -3.79313 -2.92855
H -3.22212 -2.56069 -1.69370
C 1.38366 -3.95845 0.49978
H 1.12111 -4.94067 0.88127
C 2.53009 -3.46969 0.01681
H 3.50747 -3.92516 -0.11129
C 3.42782 -1.32463 -0.67531
C 4.08716 -0.65142 0.36566
C 3.64934 -0.73291 1.81217
H 2.66544 -1.22777 1.85207
C 3.51089 0.62614 2.45694
H 4.47224 1.16697 2.49436
H 3.16203 0.52366 3.49944
H 2.78891 1.26018 1.91969
C 4.63917 -1.58194 2.64826
H 4.75473 -2.60419 2.24925
H 4.28668 -1.66102 3.69067
H 5.63956 -1.11595 2.66709
C 5.25382 0.05109 0.02575
H 5.79894 0.58337 0.81223
C 5.72783 0.06835 -1.26392
H 6.64365 0.62069 -1.50167
C 5.07489 -0.62999 -2.25795
H 5.48566 -0.63152 -3.27254
C 3.91654 -1.35934 -1.99105
C 3.28427 -2.22112 -3.06728
H 2.27237 -2.50765 -2.73226
C 3.16120 -1.47111 -4.40358
H 2.63862 -0.50608 -4.29260
H 2.60365 -2.07952 -5.13628
H 4.14975 -1.26382 -4.84676
C 4.08547 -3.50822 -3.25506
H 5.11909 -3.28817 -3.57461
H 3.62568 -4.14689 -4.02940
H 4.14764 -4.10268 -2.32869
C -4.05168 -0.74333 1.71681
H -4.32726 0.19377 2.22528
H -4.01144 -1.55756 2.45198
H -4.80062 -0.97964 0.94578
H 0.51858 0.42486 1.11771

[5]$^+$

159
SCF = -3344.68271572
Ir 0.00000 0.00000 0.00000
H -1.63689 0.25972 -0.29628
H 0.01689 -1.63557 0.40233
Zn 1.90294 1.45317 -0.56855
N -0.22594 0.12946 -3.11638
N 1.07817 -1.46780 -2.51454
C 0.25548 -0.50563 -2.00833
C -1.35867 1.04246 -3.17880
C -2.62860 0.46583 -3.26361
C -2.84418 -1.03364 -3.36106
H -1.91107 -1.53375 -3.05481
C -3.12366 -1.44785 -4.81469
H -4.05576 -0.98464 -5.18348
H -3.23971 -2.54251 -4.89223
H -2.31214 -1.14599 -5.49767
C -3.95370 -1.55109 -2.47003
H -3.80149 -1.29325 -1.40815
H -4.02301 -2.64996 -2.54735
H -4.94315 -1.15442 -2.75797
C -3.71299 1.33202 -3.34315
H -4.72542 0.92007 -3.40981
C -3.53233 2.69804 -3.36765
H -4.40138 3.36171 -3.43339
C -2.26297 3.23185 -3.34684
H -2.13032 4.31650 -3.40781
C -1.13344 2.41224 -3.26466
C 0.25560 3.00397 -3.35869
H 0.96338 2.25318 -2.96083
C 0.63986 3.25200 -4.82575
H 0.58514 2.33289 -5.43289
H 1.67035 3.64095 -4.89221
H -0.03416 3.99471 -5.28761
C 0.42537 4.27053 -2.52718
H -0.12022 5.12593 -2.96373
H 1.48969 4.55440 -2.47942
H 0.05285 4.13155 -1.49888
C 0.31514 -0.41043 -4.27291
H 0.04684 -0.03509 -5.25592
C 1.12913 -1.40089 -3.89600
H 1.74731 -2.08054 -4.47527
C 1.68517 -2.57541 -1.79629
C 0.91362 -3.73054 -1.61798
C -0.51366 -3.84247 -2.11920
H -0.95285 -2.82834 -2.08391
C -0.56793 -4.32844 -3.56334
H -0.01549 -3.67712 -4.25829
H -1.61199 -4.38107 -3.91819
H -0.13915 -5.34307 -3.64677
C -1.40592 -4.74517 -1.26978
H -1.09726 -5.80379 -1.32398
H -2.44176 -4.70540 -1.64671

H	-1.42302	-4.45425	-0.20722
C	1.53296	-4.81819	-1.00587
H	0.96894	-5.74378	-0.85819
C	2.84858	-4.75146	-0.58577
H	3.31300	-5.62016	-0.10690
C	3.58004	-3.59444	-0.77330
H	4.62363	-3.55485	-0.44648
C	3.01979	-2.48149	-1.40332
C	3.87076	-1.25648	-1.69331
H	3.19044	-0.41169	-1.90684
C	4.72733	-1.48562	-2.94465
H	5.43073	-2.32233	-2.78892
H	5.31874	-0.58342	-3.17715
H	4.11848	-1.72708	-3.83246
C	4.75642	-0.85990	-0.52071
H	4.18691	-0.79438	0.42122
H	5.22532	0.11947	-0.71273
H	5.57410	-1.58496	-0.36256
C	3.38428	2.41731	-1.37690
C	3.67349	2.20824	-2.72287
H	3.06197	1.52451	-3.32705
C	4.74145	2.84660	-3.34869
H	4.93719	2.66253	-4.41130
C	5.54284	3.70020	-2.63122
H	6.38671	4.20077	-3.11988
C	5.29280	3.91549	-1.29902
H	5.93014	4.59163	-0.71785
C	4.21566	3.28981	-0.68613
H	4.04028	3.49481	0.37518
H	1.63689	-0.25972	0.29628
H	-0.01689	1.63557	-0.40233
Zn	-1.90294	-1.45317	0.56855
N	0.22594	-0.12946	3.11638
N	-1.07817	1.46780	2.51454
C	-0.25548	0.50563	2.00833
C	1.35867	-1.04246	3.17880
C	2.62860	-0.46583	3.26361
C	2.84418	1.03364	3.36106
H	1.91107	1.53375	3.05481
C	3.12366	1.44785	4.81469
H	4.05576	0.98464	5.18348
H	3.23971	2.54251	4.89223
H	2.31214	1.14599	5.49767
C	3.95370	1.55109	2.47003
H	3.80149	1.29325	1.40815
H	4.02301	2.64996	2.54735
H	4.94315	1.15442	2.75797
C	3.71299	-1.33202	3.34315
H	4.72542	-0.92007	3.40981
C	3.53233	-2.69804	3.36765
H	4.40138	-3.36171	3.43339
C	2.26297	-3.23185	3.34684
H	2.13032	-4.31650	3.40781
C	1.13344	-2.41224	3.26466
C	-0.25560	-3.00397	3.35869
H	-0.96338	-2.25318	2.96083
C	-0.63986	-3.25200	4.82575
H	-0.58514	-2.33289	5.43289
H	-1.67035	-3.64095	4.89221
H	0.03416	-3.99471	5.28761
C	-0.42537	-4.27053	2.52718
H	0.12022	-5.12593	2.96373
H	-1.48969	-4.55440	2.47942
H	-0.05285	-4.13155	1.49888
C	-0.31514	0.41043	4.27291
H	-0.04684	0.03509	5.25592
C	-1.12913	1.40089	3.89600
H	-1.74731	2.08054	4.47527
C	-1.68517	2.57541	1.79629
C	-0.91362	3.73054	1.61798
C	0.51366	3.84247	2.11920
H	0.95285	2.82834	2.08391
C	0.56793	4.32844	3.56334
H	0.01549	3.67712	4.25829
H	1.61199	4.38107	3.91819
H	0.13915	5.34307	3.64677
C	1.40592	4.74517	1.26978
H	1.09726	5.80379	1.32398
H	2.44176	4.70540	1.64671
H	1.42302	4.45425	0.20722
C	-1.53296	4.81819	1.00587
H	-0.96894	5.74378	0.85819
C	-2.84858	4.75146	0.58577
H	-3.31300	5.62016	0.10690
C	-3.58004	3.59444	0.77330
H	-4.62363	3.55485	0.44648
C	-3.01979	2.48149	1.40332
C	-3.87076	1.25648	1.69331
H	-3.19044	0.41169	1.90684
C	-4.72733	1.48562	2.94465
H	-5.43073	2.32233	2.78892
H	-5.31874	0.58342	3.17715
H	-4.11848	1.72708	3.83246
C	-4.75642	0.85990	0.52071
H	-4.18691	0.79438	-0.42122
H	-5.22532	-0.11947	0.71273
H	-5.57410	1.58496	0.36256
C	-3.38428	-2.41731	1.37690

C	-3.67349	-2.20824	2.72287
H	-3.06197	-1.52451	3.32705
C	-4.74145	-2.84660	3.34869
H	-4.93719	-2.66253	4.41130
C	-5.54284	-3.70020	2.63122
H	-6.38671	-4.20077	3.11988
C	-5.29280	-3.91549	1.29902
H	-5.93014	-4.59163	0.71785
C	-4.21566	-3.28981	0.68613
H	-4.04028	-3.49481	-0.37518

[11b]$^{+}$

141

SCF = -2694.63630056

Ir	-0.02420	0.06191	-0.08714
H	0.31059	-0.79444	-1.44175
H	0.86890	-0.81339	1.11072
H	-0.29333	0.88877	1.41826
H	-0.73809	1.36541	0.78655
H	-0.83931	0.90347	-1.21844
Zn	1.14361	-2.12134	-0.13974
N	2.92978	0.89382	-0.58467
N	1.64306	2.59890	-0.66524
N	-2.22147	-1.48479	1.48151
N	-2.54366	-1.67423	-0.62761
C	1.61953	1.25324	-0.44868
C	3.72748	1.99014	-0.89291
H	4.79931	1.89047	-1.03538
C	2.92901	3.05159	-0.93682
H	3.13290	4.10015	-1.13197
C	3.49585	-0.40058	-0.32097
C	3.77947	-0.72743	1.01803
C	4.39709	-1.94983	1.23593
H	4.63722	-2.25381	2.25979
C	4.73346	-2.77661	0.18821
H	5.23324	-3.73007	0.39120
C	4.47000	-2.41483	-1.10383
H	4.77210	-3.09035	-1.90741
C	3.83392	-1.21892	-1.40672
C	3.51839	0.24903	2.15140
H	2.66297	0.87816	1.84842
C	4.71519	1.15707	2.37253
H	5.59747	0.57673	2.69719
H	4.49939	1.89790	3.16183
H	5.00321	1.71472	1.46622
C	3.13064	-0.40307	3.46947
H	2.23099	-1.02955	3.37516
H	2.91280	0.37665	4.21889
H	3.94247	-1.02405	3.88804
C	3.59611	-0.80051	-2.84591
H	2.81230	-0.03046	-2.91511
C	0.54155	3.50855	-0.46625
C	0.33650	3.98614	0.82837
C	-0.71917	4.87272	1.00724
H	-0.92105	5.27906	2.00362
C	-1.51122	5.25404	-0.05719
H	-2.34172	5.94921	0.10834
C	-1.24786	4.79848	-1.32922
H	-1.86317	5.14721	-2.16512
C	-0.19362	3.92135	-1.57544
C	1.26109	3.62835	1.97790
H	1.83346	2.72888	1.69422
C	2.26131	4.75623	2.21611
H	2.85812	4.98222	1.31640
H	2.96376	4.49433	3.02638
H	1.74486	5.68692	2.51056
C	0.52873	3.30577	3.27762
H	-0.01446	4.18151	3.67424
H	1.25315	3.00418	4.05353
H	-0.19748	2.48495	3.16543
C	0.18573	3.52025	-2.98993
H	0.86290	2.64988	-2.92957
C	0.93461	4.67542	-3.63762
H	1.27001	4.40794	-4.65509
H	1.82679	4.97846	-3.06275
H	0.28772	5.56621	-3.72538
C	-0.99942	3.12211	-3.85647
H	-1.72117	3.94810	-3.98228
H	-1.53696	2.25340	-3.44499
H	-0.64993	2.84838	-4.86706
C	-1.70399	-1.09240	0.28214
C	-3.33470	-2.29168	1.31444
H	-3.87838	-2.69360	2.16440
C	-3.52749	-2.41483	0.00692
H	-4.28401	-2.95335	-0.55632
C	-1.81221	-1.00576	2.78247
C	-0.97189	-1.79327	3.56114
C	-0.57128	-1.27536	4.79491
H	0.09172	-1.86498	5.43598
C	-1.01976	-0.04660	5.22381
H	-0.69302	0.34216	6.19471
C	-1.90969	0.67496	4.46607
H	-2.30271	1.62339	4.84906
C	-2.34743	0.21250	3.22327
C	-0.59438	-3.21243	3.16617
H	-0.82285	-3.34395	2.09256
C	0.87639	-3.52794	3.36357
H	1.52391	-2.87237	2.76026

```
H   1.08695 -4.56802  3.06130
H   1.18725 -3.43311  4.41866
C  -1.47000 -4.19115  3.95649
H  -1.27462 -4.10872  5.04003
H  -1.26277 -5.23329  3.65711
H  -2.54643 -4.00494  3.80350
C  -3.41450  0.95541  2.45388
H  -3.57308  0.43340  1.49615
C  -3.04836  2.39304  2.13336
H  -2.86504  2.98220  3.04955
H  -3.87714  2.88677  1.59718
H  -2.15642  2.48856  1.49704
C  -4.73681  0.91563  3.20817
H  -5.05569 -0.11672  3.43230
H  -5.53697  1.39406  2.61743
H  -4.67083  1.45396  4.17019
C  -2.59737 -1.36830 -2.03885
C  -3.28399 -0.20563 -2.40203
C  -3.34795  0.08963 -3.76320
H  -3.88079  0.98608 -4.09502
C  -2.75188 -0.73259 -4.69302
H  -2.80613 -0.47409 -5.75662
C  -2.12473 -1.89296 -4.30934
H  -1.69444 -2.55276 -5.06928
C  -2.04000 -2.25542 -2.95934
C  -4.02088  0.63886 -1.38076
H  -3.52397  0.49366 -0.40777
C  -4.00689  2.13158 -1.65745
H  -2.98280  2.53273 -1.70979
H  -4.53456  2.66708 -0.84966
H  -4.52693  2.39099 -2.59671
C  -5.47063  0.14963 -1.24870
H  -6.01877  0.29637 -2.19620
H  -6.00338  0.71540 -0.46440
H  -5.53540 -0.91976 -0.99188
C  -1.47026 -3.59727 -2.55869
H  -1.21159 -3.55656 -1.48347
C  -0.22071 -3.99462 -3.32539
H  -0.43675 -4.20140 -4.38820
H   0.21225 -4.91616 -2.90205
H   0.54943 -3.20831 -3.29351
C  -2.53389 -4.67534 -2.75368
H  -3.45250 -4.47668 -2.17601
H  -2.15411 -5.66500 -2.44482
H  -2.82626 -4.74536 -3.81609
C   1.55651 -4.00063 -0.09105
H   2.31751 -4.18771  0.68159
H   1.94444 -4.36878 -1.05380
H   0.65303 -4.58247  0.16121
C   4.93794 -0.31124 -3.38676
H   5.28837  0.60732 -2.88726
H   4.87613 -0.09049 -4.46759
H   5.71355 -1.08239 -3.24795
C   3.19693 -2.08365 -3.57622
H   4.05598 -2.75641 -3.72251
H   2.81055 -1.85995 -4.58717
H   2.41610 -2.64193 -3.03780
```

[14c]$^{+}$

```
155
SCF = -2859.08188820
Ir  0.01036  0.00439 -0.24725
H   0.74518 -1.44102 -0.36118
H   0.52863 -0.16768  1.31409
H  -0.81772  1.52371 -0.31704
Zn -0.55171  1.63471  1.53453
P  -0.35393 -0.21796 -2.55794
N   3.03269  0.22512 -1.02450
N   2.16436  2.19578 -0.80247
N  -1.69929 -1.91665  1.45999
N  -3.06861 -0.71694  0.31924
C   1.85465  0.85597 -0.68114
C   3.35696 -1.18748 -0.92485
C   3.66190 -1.91968 -2.07580
C   3.97404 -1.29508 -3.42989
H   3.58539 -0.26497 -3.46024
C   5.51367 -1.17349 -3.58335
H   5.98445 -0.63243 -2.74569
H   5.76905 -0.64722 -4.51901
H   5.97990 -2.17319 -3.61858
C   3.41719 -2.06915 -4.62807
H   3.89502 -3.05697 -4.73553
H   3.61472 -1.51709 -5.56250
H   2.32907 -2.23185 -4.55464
C   3.84696 -3.29654 -1.93286
H   4.06739 -3.90632 -2.81356
C   3.77031 -3.89567 -0.68629
H   3.89071 -4.98073 -0.59637
C   3.63783 -3.12083  0.44609
H   3.68490 -3.59514  1.42904
C   3.47558 -1.74940  0.35857
C   3.61901 -0.85213  1.59848
H   2.85454 -0.05847  1.56013
C   3.49499 -1.60338  2.89854
H   4.32651 -2.31765  3.03708
H   2.55272 -2.16362  2.98079
H   3.54264 -0.90274  3.74927
C   5.05298 -0.19414  1.63221
```

H	5.82329	-0.98308	1.66158
H	5.15672	0.41875	2.54328
H	5.25655	0.45143	0.76298
C	4.00733	1.16183	-1.38151
H	5.00431	0.86115	-1.68569
C	3.46292	2.36343	-1.23883
H	3.88811	3.35310	-1.38141
C	1.46625	3.33019	-0.20610
C	0.51879	4.06279	-0.92830
C	1.23889	4.31592	-3.31009
H	1.24979	5.41712	-3.23468
H	1.01072	4.05680	-4.35891
H	2.25799	3.95662	-3.09221
C	-1.19681	4.17862	-2.81758
H	-2.00617	3.79850	-2.17357
H	-1.38778	3.82840	-3.84627
H	-1.27848	5.27941	-2.84771
C	-0.05851	5.14731	-0.29297
H	-0.81224	5.74423	-0.81334
C	0.30975	5.49975	1.00402
H	-0.16823	6.36245	1.48090
C	1.27853	4.80007	1.66951
H	1.58072	5.11527	2.67354
C	1.89588	3.70798	1.08550
C	3.04868	3.01761	1.81612
H	3.32716	2.10415	1.26880
C	4.28538	3.94546	1.84298
H	4.07638	4.86855	2.41067
H	4.60089	4.24355	0.82937
H	5.13655	3.43809	2.32793
C	2.66596	2.59768	3.24439
H	3.52357	2.10786	3.73727
H	1.82361	1.88897	3.24833
H	2.38407	3.46111	3.87077
C	0.18645	3.72166	-2.36061
H	0.21953	2.62369	-2.43310
C	-1.04022	2.66391	3.15684
H	-0.13399	2.70376	3.78634
H	-1.18969	3.69794	2.79421
C	-2.21772	2.20403	3.96518
H	-2.42056	2.87668	4.82256
H	-2.07196	1.19762	4.39591
H	-3.15224	2.15808	3.38445
C	0.96221	0.39180	-3.65594
H	1.13275	1.47013	-3.54154
H	0.69483	0.18755	-4.70635
H	1.89524	-0.12689	-3.42674
C	-1.75779	0.60027	-3.37858
H	-2.71814	0.19840	-3.04473
H	-1.67957	0.47592	-4.47263
H	-1.73911	1.67175	-3.13885
C	-0.44639	-1.93752	-3.09981
H	0.49276	-2.43990	-2.82058
H	-0.59646	-2.02203	-4.19024
H	-1.26651	-2.45682	-2.58594
C	-1.71884	-0.97848	0.46471
C	-0.62058	-2.72339	2.00068
C	-0.21119	-3.84116	1.26343
C	-0.77880	-4.16529	-0.10441
H	-1.05958	-3.20916	-0.57760
C	0.22155	-4.85464	-1.03568
H	1.16249	-4.28805	-1.11926
H	-0.21315	-4.95735	-2.04494
H	0.46720	-5.87657	-0.69690
C	-2.04118	-5.02361	0.04027
H	-1.80440	-5.98840	0.52282
H	-2.47757	-5.24757	-0.94952
H	-2.81876	-4.52991	0.64622
C	0.67838	-4.71476	1.87992
H	1.02327	-5.59962	1.33729
C	1.09268	-4.50638	3.16961
H	1.77054	-5.22727	3.64189
C	0.66257	-3.41804	3.88182
H	0.99452	-3.26864	4.91405
C	-0.20375	-2.49244	3.29642
C	-0.68473	-1.29657	4.11440
H	-1.26717	-0.63469	3.44951
C	0.50074	-0.47844	4.65653
H	1.10465	-1.06070	5.37336
H	0.13619	0.41814	5.18633
H	1.16639	-0.15140	3.84274
C	-1.58683	-1.74123	5.26704
H	-2.47374	-2.29687	4.91871
H	-1.94516	-0.87000	5.84260
H	-1.04302	-2.39829	5.96801
C	-2.97840	-2.17344	1.94925
H	-3.14161	-2.88366	2.75396
C	-3.82490	-1.42701	1.24517
H	-4.90339	-1.31720	1.29735
C	-3.80735	-0.04195	-0.74298
C	-4.32327	-0.82309	-1.78398
C	-4.07091	-2.31909	-1.88356
H	-3.15775	-2.55850	-1.31118
C	-3.89181	-2.80510	-3.32598
H	-4.84654	-2.79036	-3.87945
H	-3.53988	-3.85064	-3.33486
H	-3.17068	-2.19828	-3.89636
C	-5.25324	-3.12000	-1.25151

H -5.39291 -2.89831 -0.18399
H -5.07209 -4.20364 -1.34973
H -6.19450 -2.88381 -1.77690
C -5.11795 -0.17363 -2.71621
H -5.53760 -0.74400 -3.55084
C -5.37883 1.18477 -2.62967
H -5.99996 1.66372 -3.39425
C -4.91050 1.90792 -1.57489
H -5.17623 2.96642 -1.48356
C -4.12357 1.32037 -0.58379
C -3.82914 2.05571 0.69564
H -3.04892 1.50562 1.25102
C -3.32381 3.50386 0.47413
H -2.38719 3.52053 -0.10386
H -3.12943 3.98566 1.44587
H -4.07106 4.12138 -0.05261
C -5.10212 2.09601 1.56991
H -5.92620 2.59440 1.03030
H -4.92494 2.65911 2.50076
H -5.45008 1.08617 1.84552

[15b]
140
SCF = -2694.21668880
Ir 0.02221 0.03245 0.03180
H 1.59135 -0.20427 0.41908
H -0.18454 -0.11833 1.66232
H -1.67311 0.24724 0.02879
H 0.65531 0.09755 -1.54474
Zn -1.39135 0.23313 -1.92008
N -1.21596 -2.78658 0.39925
N 0.57730 -2.88973 -0.76091
N -0.47577 3.08999 0.21503
N 1.55353 2.62407 0.68355
C 3.14054 1.87352 2.36773
C -2.42398 0.10195 -3.56363
H -3.35757 0.68542 -3.48873
H -2.69869 -0.94642 -3.78163
H -1.85547 0.48117 -4.43016
C -0.22400 -1.98861 -0.11397
C -2.33738 -2.42712 1.24135
C -3.58605 -2.25572 0.64422
C -3.76534 -2.24393 -0.86494
H -2.80153 -1.93212 -1.30800
C -4.09743 -3.64054 -1.41276
H -3.30985 -4.37795 -1.19023
H -4.22000 -3.60396 -2.50958
H -5.04359 -4.01519 -0.98148
C -4.82545 -1.25141 -1.32872
H -5.84178 -1.55876 -1.02250
H -4.82664 -1.18730 -2.42934
H -4.63861 -0.23907 -0.93384
C -4.69487 -2.12043 1.49106
H -5.68923 -1.98602 1.05479
C -4.54732 -2.16885 2.85236
H -5.43089 -2.07610 3.49566
C -3.30518 -2.31951 3.42705
H -3.20475 -2.34455 4.51664
C -2.16351 -2.44954 2.62591
C -0.80347 -2.65452 3.25745
H -0.03901 -2.45536 2.48849
C -0.62997 -4.10390 3.75841
H -1.37895 -4.34488 4.53414
H 0.37145 -4.24299 4.20201
H -0.74265 -4.83923 2.94366
C -0.54894 -1.68617 4.40682
H -0.65452 -0.64056 4.07535
H 0.47519 -1.81744 4.79617
H -1.23763 -1.85376 5.25455
C -1.01609 -4.12405 0.08892
H -1.69834 -4.89532 0.43400
C 0.10351 -4.19469 -0.63646
H 0.62953 -5.03592 -1.07797
C 1.88081 -2.67686 -1.34664
C 2.98318 -2.81338 -0.49616
C 2.85634 -2.94450 1.01067
H 1.80431 -2.74161 1.27222
C 3.18201 -4.37302 1.47936
H 4.23024 -4.64003 1.25235
H 2.53432 -5.12371 0.99484
H 3.04480 -4.46248 2.57131
C 3.69292 -1.92663 1.76404
H 4.77599 -2.06684 1.59356
H 3.52134 -2.02425 2.85068
H 3.42888 -0.90041 1.46837
C 4.25143 -2.84521 -1.10205
H 5.14328 -2.96395 -0.47789
C 4.36637 -2.73533 -2.46243
H 5.36355 -2.77486 -2.91933
C 3.27435 -2.55066 -3.26527
H 3.40132 -2.44195 -4.34698
C 1.98430 -2.52314 -2.71363
C 0.77609 -2.34408 -3.61760
H -0.09333 -2.11544 -2.97567
C 0.46851 -3.63680 -4.39051
H 1.31639 -3.92132 -5.03930
H -0.41860 -3.50489 -5.03477
H 0.26915 -4.48347 -3.71151

```
C   0.96249 -1.17642 -4.57320
H   1.19071 -0.24713 -4.02558
H   0.04154 -1.00800 -5.15751
H   1.77775 -1.35640 -5.29652
C   0.38421  2.02229  0.32076
C  -1.85542  3.02686 -0.19970
C  -2.17618  3.40532 -1.50743
C  -1.15535  4.02409 -2.46812
H  -0.21490  4.08161 -1.88617
C  -0.83405  3.27758 -3.65551
H  -0.43104  2.27565 -3.42753
H  -0.06913  3.80197 -4.25821
H  -1.70233  3.11842 -4.32493
C  -1.52014  5.48303 -2.73513
H  -2.45262  5.57520 -3.32265
H  -0.72074  5.98366 -3.31013
H  -1.66905  6.04524 -1.79775
C  -3.50200  3.26253 -1.89514
H  -3.78543  3.53205 -2.91988
C  -4.46531  2.79964 -1.02407
H  -5.50319  2.69131 -1.35753
C  -4.11984  2.49373  0.28694
H  -4.89486  2.14912  0.97821
C  -2.81332  2.60346  0.72882
C  -2.42226  2.29960  2.16693
H  -1.41647  1.84601  2.13558
C  -2.35601  3.59296  2.98774
H  -1.63565  4.31691  2.57147
H  -2.04830  3.37686  4.02647
H  -3.34388  4.08743  3.02914
C  -3.33533  1.28650  2.82634
H  -4.35361  1.68306  2.99884
H  -2.93615  1.00441  3.81578
H  -3.41768  0.36774  2.22630
C   0.15017  4.29568  0.50388
H  -0.38298  5.24138  0.46269
C   1.41699  4.00716  0.78851
H   2.25642  4.63851  1.06425
C   2.83266  2.03522  1.01502
C   3.75074  1.81806 -0.00584
C   3.38356  2.00893 -1.46547
H   2.28275  2.01013 -1.53407
C   3.88086  0.86189 -2.33123
H   4.98414  0.80753 -2.36535
H   3.53539  0.99197 -3.37216
H   3.49914 -0.10208 -1.96562
C   3.90712  3.35438 -1.97321
H   3.50483  4.20101 -1.39006
H   3.62634  3.51147 -3.03017
H   5.00959  3.40210 -1.91082
C   5.05258  1.45883  0.36609
H   5.80658  1.28719 -0.40866
C   5.39697  1.33664  1.70438
H   6.42597  1.07313  1.97600
C   4.45660  1.52392  2.68918
H   4.73678  1.40122  3.74111
C   2.10194  2.07752  3.45600
H   1.11181  2.13842  2.97384
C   2.06570  0.90090  4.42470
H   1.89996 -0.04626  3.88836
H   1.24767  1.02775  5.15523
H   3.00387  0.81039  5.00143
C   2.35611  3.39271  4.21233
H   3.34842  3.38355  4.69801
H   1.59849  3.54355  5.00174
H   2.32022  4.26738  3.54157
```

(ii) Fully optimized structures for SCXRD determined species and the isomers of $[10]^+$.

$[4a]^+$

142
SCF(BS1) = -2882.80324356
H(0 K) = -2881.542969
G(298 K) = -2881.725272
SCF(BS1)+D3(BJ) = -2883.21799848
SCF(PCM=THF) = -2882.85227209
SCF(PCM=C6H5F) = -2882.84904638
SCF(BS2) = -4436.05996757
Lowest Freq. = 10.8770cm-1, 14.2263cm-1, 22.3918cm-1

```
Ir  0.36274  0.01992 -0.43096
H   0.61515  0.36187  1.12918
Zn -1.81889 -0.40402  0.44959
N  -1.34566  2.46270 -1.36947
N   0.17738  3.14509  0.02867
N   0.57484 -3.00930  0.53722
N   2.52113 -2.22481 -0.02071
C  -0.31926  1.98714 -0.54904
C  -2.16564  1.65236 -2.23127
C  -1.51175  0.58656 -2.90012
C  -0.05214  0.31102 -2.67971
C   0.91570  1.29220 -3.33690
H   0.65780  2.34198 -3.12507
H   0.87371  1.16313 -4.43480
```

H	1.95370	1.11697	-3.00996
C	0.35489	-1.01387	-2.43440
H	-0.38094	-1.82112	-2.40773
H	1.37471	-1.31308	-2.69139
C	-2.24682	-0.19517	-3.80852
H	-1.73642	-0.99589	-4.35229
C	-3.60575	0.06734	-4.03427
H	-4.16945	-0.53307	-4.75515
C	-4.23888	1.09605	-3.33149
H	-5.30485	1.27904	-3.49763
C	-3.55222	1.90733	-2.40018
C	-4.34745	2.98082	-1.64790
H	-3.71452	3.38174	-0.83753
C	-4.73301	4.14833	-2.58916
H	-3.86086	4.60316	-3.09180
H	-5.25665	4.94050	-2.02794
H	-5.41088	3.79783	-3.38645
C	-5.61092	2.40556	-0.96897
H	-6.34564	2.04264	-1.70788
H	-6.10531	3.19648	-0.37977
H	-5.36416	1.57306	-0.29250
C	-1.42187	3.85630	-1.33841
H	-2.10739	4.41484	-1.96601
C	-0.47775	4.28042	-0.45613
H	-0.19449	5.27761	-0.13346
C	1.27155	3.34404	0.96737
C	2.53737	3.71545	0.44654
C	2.82062	3.82083	-1.05211
H	1.94476	3.42419	-1.59255
C	3.00030	5.29938	-1.47281
H	2.11809	5.90996	-1.21718
H	3.16022	5.37239	-2.56213
H	3.87434	5.75205	-0.97366
C	4.04043	2.98034	-1.48033
H	4.96945	3.33908	-1.00450
H	4.18461	3.05010	-2.57241
H	3.91658	1.91936	-1.21152
C	3.53542	4.08290	1.37134
H	4.52299	4.37622	1.00074
C	3.27476	4.11436	2.74554
H	4.05901	4.42189	3.44484
C	2.00599	3.76762	3.22785
H	1.80959	3.81210	4.30313
C	0.97207	3.37879	2.35406
C	-0.42922	3.09348	2.89549
H	-0.96557	2.49520	2.13675
C	-1.21430	4.41181	3.10488
H	-0.70481	5.05663	3.84173
H	-2.22941	4.20051	3.48282
H	-1.31553	4.98421	2.16813
C	-0.40547	2.27315	4.20115
H	0.19091	1.35367	4.08937
H	-1.43191	1.98687	4.48751
H	0.01052	2.85166	5.04390
C	-3.65657	-0.35698	1.09045
C	-3.99287	0.41006	2.23057
H	-3.22035	0.98036	2.76134
C	-5.31400	0.45973	2.71071
H	-5.55128	1.05965	3.59637
C	-6.32682	-0.25832	2.05502
H	-7.35606	-0.22187	2.42718
C	-6.01419	-1.01997	0.91681
H	-6.80177	-1.57736	0.39728
C	-4.69260	-1.06597	0.43877
H	-4.47406	-1.66610	-0.45098
C	1.18753	-1.84927	0.08107
C	-0.81185	-3.24701	0.90495
C	-1.65855	-3.91376	-0.02374
C	-1.19877	-4.32765	-1.42216
H	-0.24324	-3.81556	-1.63154
C	-2.20663	-3.92587	-2.52140
H	-3.15813	-4.47559	-2.42134
H	-1.79741	-4.16742	-3.51738
H	-2.43986	-2.84775	-2.49647
C	-0.92888	-5.85166	-1.47690
H	-0.16787	-6.16122	-0.74170
H	-0.57059	-6.14291	-2.47899
H	-1.84924	-6.42320	-1.26699
C	-2.94480	-4.28161	0.41858
H	-3.61903	-4.79740	-0.27236
C	-3.36360	-4.02836	1.72915
H	-4.36594	-4.32697	2.05080
C	-2.49824	-3.40367	2.63385
H	-2.83251	-3.22674	3.65963
C	-1.20082	-3.00812	2.25477
C	-0.24124	-2.43348	3.29877
H	0.53307	-1.85448	2.76409
C	0.46050	-3.57581	4.07541
H	-0.28099	-4.19646	4.60773
H	1.15419	-3.15902	4.82552
H	1.04017	-4.23535	3.40970
C	-0.93465	-1.47673	4.28993
H	-1.50598	-0.69023	3.77062
H	-0.18015	-0.99161	4.93117
H	-1.62928	-2.01083	4.96086
C	1.49618	-4.04993	0.70076
H	1.18800	-5.03092	1.04950
C	2.71505	-3.55669	0.35484

H 3.70177 -4.00992 0.35124
C 3.67763 -1.38594 -0.29456
C 4.25309 -0.68515 0.79915
C 3.72433 -0.81370 2.22798
H 2.72347 -1.27580 2.18350
C 3.56488 0.54470 2.93340
H 4.53193 1.06010 3.06248
H 3.13951 0.39800 3.94107
H 2.89485 1.21038 2.36897
C 4.64319 -1.75563 3.04475
H 4.72561 -2.75331 2.58177
H 4.24801 -1.88618 4.06672
H 5.66218 -1.33961 3.12762
C 5.43208 0.04488 0.55354
H 5.89915 0.59534 1.37607
C 6.03325 0.04933 -0.71077
H 6.95433 0.61714 -0.87718
C 5.47723 -0.69980 -1.75438
H 5.98148 -0.72622 -2.72536
C 4.29926 -1.45190 -1.57063
C 3.82628 -2.38689 -2.68653
H 2.81458 -2.74672 -2.42606
C 4.74703 -3.63025 -2.77064
H 5.78007 -3.33691 -3.02439
H 4.38896 -4.32242 -3.55174
H 4.78282 -4.18344 -1.81763
C 3.75108 -1.69699 -4.06647
H 3.12988 -0.78647 -4.04392
H 3.32182 -2.38705 -4.81245
H 4.75150 -1.40790 -4.43062

[4c]$^{+}$
138
SCF(BS1) = -2730.38133006
H(0 K) = -2729.147362
G(298 K) = -2729.325789
SCF(BS1)+D3(BJ) = -2730.77583519
SCF(PCM=THF) = -2730.42912380
SCF(PCM=C6H5F) = -2730.42615413
SCF(BS2) = -4283.58385444
Lowest Freq. = 10.2904cm-1, 14.2805cm-1, 21.8917cm-1

N -1.93989 2.35972 -0.96852
N -0.24749 3.13028 0.16328
N 2.40935 -2.04530 -0.31383
N 0.64605 -2.98962 0.52945
C -0.76606 1.94320 -0.33010
C -1.03150 4.22650 -0.20652
H -0.75107 5.23936 0.06596
C -2.08247 3.74802 -0.92422
H -2.88642 4.26680 -1.43426
C -2.85453 1.50127 -1.67295
C -4.26131 1.68837 -1.60484
C -6.08075 2.14095 0.14041
H -6.90801 1.72280 -0.45756
H -5.69159 1.34006 0.78818
H -6.50907 2.92690 0.78524
C -4.96954 2.74551 -0.74777
H -4.23075 3.19245 -0.06046
C -5.55656 3.87345 -1.63173
H -6.34028 3.47617 -2.29943
H -6.01408 4.65807 -1.00585
H -4.79917 4.34901 -2.27963
C -5.05679 0.82788 -2.39534
H -6.14304 0.95991 -2.38193
C -4.50307 -0.18467 -3.18413
H -5.15172 -0.82544 -3.78945
C -3.11422 -0.37628 -3.19484
H -2.66556 -1.16084 -3.81157
C -2.27326 0.45717 -2.43648
C -0.78475 0.26383 -2.47646
C -0.26907 -1.03600 -2.31078
H 0.70097 -1.28449 -2.75067
H -0.94421 -1.87993 -2.15070
C -0.00548 1.29191 -3.29432
H 1.08154 1.18332 -3.14824
H -0.22598 1.14691 -4.36863
H -0.28534 2.32731 -3.04312
C 0.97222 3.39900 0.91017
C 0.90649 3.41179 2.32788
C -0.36091 3.02083 3.08965
H -0.94675 2.34752 2.43779
C -1.22800 4.26815 3.39178
H -1.53198 4.79297 2.47118
H -2.14441 3.97775 3.93395
H -0.67344 4.98458 4.02231
C -0.06097 2.25588 4.39464
H 0.41065 2.90439 5.15304
H -0.99963 1.87942 4.83492
H 0.60596 1.39730 4.21593
C 2.04182 3.87490 3.02063
H 2.02636 3.90487 4.11395
C 3.18462 4.31091 2.33695
H 4.05099 4.67411 2.89920
C 3.21464 4.29519 0.93841
H 4.10434 4.65533 0.41154
C 2.10368 3.85767 0.18925

```
C   2.12625  3.97459 -1.33511
H   1.18591  3.54842 -1.72366
C   3.28361  3.17320 -1.96408
H   3.23210  2.10737 -1.69060
H   3.24862  3.25436 -3.06434
H   4.26649  3.55441 -1.63765
C   2.18287  5.45758 -1.77319
H   3.11429  5.94027 -1.43065
H   2.15038  5.53454 -2.87342
H   1.33836  6.03898 -1.36652
C  -3.65385 -0.62950  1.85601
H  -3.44688 -1.11773  2.82395
H  -3.92919  0.41935  2.07541
C  -4.77984 -1.35580  1.10052
H  -4.53130 -2.41520  0.92810
H  -5.72614 -1.33103  1.67505
H  -4.98407 -0.90089  0.11644
C   1.08192 -1.78021  0.00093
C  -0.63597 -3.35196  1.11141
C  -1.55052 -4.10782  0.32731
C  -1.29138 -4.47530 -1.13412
H  -0.43839 -3.87144 -1.49013
C  -0.88908 -5.96541 -1.26006
H  -1.70089 -6.62505 -0.90837
H  -0.67984 -6.21887 -2.31328
H   0.01224 -6.19954 -0.66992
C  -2.50364 -4.17211 -2.04255
H  -2.83946 -3.12538 -1.95013
H  -2.24240 -4.36102 -3.09788
H  -3.36424 -4.81921 -1.80156
C  -2.70222 -4.60404  0.97009
H  -3.42195 -5.19412  0.39412
C  -2.92620 -4.38485  2.33417
H  -3.82187 -4.79220  2.81391
C  -1.99616 -3.66118  3.08964
H  -2.16888 -3.51851  4.16038
C  -0.82499 -3.13796  2.50624
C   0.22811 -2.46254  3.38676
H   0.88099 -1.85832  2.73234
C  -0.38556 -1.50836  4.43149
H  -0.98174 -2.04803  5.18720
H   0.41688 -0.98103  4.97338
H  -1.03480 -0.75088  3.96182
C   1.10468 -3.52913  4.08926
H   1.61924 -4.18161  3.36518
H   1.87391 -3.04300  4.71328
H   0.49065 -4.16947  4.74601
C   1.66464 -3.94955  0.53476
H   1.49724 -4.95186  0.91724
C   2.76863 -3.35650  0.00860
H   3.77616 -3.72593 -0.15696
C   3.44104 -1.12214 -0.76121
C   3.84331 -1.13725 -2.12373
C   4.92514 -0.30963 -2.48667
H   5.26089 -0.29600 -3.52826
C   5.60025  0.46474 -1.53585
H   6.44427  1.09116 -1.84204
C   5.21917  0.41243 -0.18973
H   5.78139  0.98562  0.55397
C   4.14545 -0.39396  0.23453
C   3.86955 -0.56648  1.72845
H   2.90472 -1.08990  1.84085
C   4.96668 -1.45381  2.36682
H   5.95786 -0.97477  2.28706
H   4.75523 -1.61506  3.43778
H   5.03242 -2.44164  1.88062
C   3.74648  0.77418  2.47373
H   2.95360  1.40023  2.03811
H   3.50263  0.59428  3.53492
H   4.68856  1.34830  2.45131
C   3.24974 -2.09329 -3.16121
H   2.32347 -2.52317 -2.73928
C   4.22235 -3.27031 -3.42420
H   4.45700 -3.82623 -2.50150
H   3.78222 -3.97979 -4.14558
H   5.17505 -2.90563 -3.84487
C   2.89385 -1.39932 -4.49416
H   3.79520 -1.03398 -5.01486
H   2.39515 -2.11278 -5.17188
H   2.22187 -0.53781 -4.34801
H   0.55860  0.39474  1.14139
Ir  0.04428  0.02108 -0.34613
Zn -1.93124 -0.53143  0.90081
```

[8]$^{+}$

135
SCF(BS1) = -2631.66510797
H(0 K) = -2630.460774
G(298 K) = -2630.636648
SCF(BS1)+D3(BJ) = -2632.05510028
SCF(PCM=THF) = -2631.71853145
SCF(PCM=C6H5F) = -2631.71500521
SCF(BS2) = -2632.47388551
Lowest Freq. = 13.0684cm-1, 18.0559cm-1, 20.1851cm-1

```
Ir  0.08236  0.03422 -0.39134
Cd -1.98421 -0.67369  1.01214
```

N	-2.11486	2.14835	-1.05770	H	3.62650	5.03810	0.50688
N	-0.54668	3.09289	0.12159	C	1.72107	4.05113	0.22039
N	0.88978	-2.92476	0.51443	C	1.77673	4.18268	-1.30175
N	2.58959	-1.85223	-0.30661	H	0.91893	3.63130	-1.72219
C	-0.91490	1.86051	-0.39679	C	3.05548	3.56222	-1.90001
C	-2.89953	1.20336	-1.80734	H	3.96315	4.08551	-1.55270
C	-2.17694	0.25865	-2.57889	H	3.03508	3.63988	-3.00083
C	-0.67635	0.22269	-2.55523	H	3.15703	2.50004	-1.62682
C	-0.04006	-1.02033	-2.37519	C	1.63668	5.66396	-1.72808
H	-0.63427	-1.92926	-2.25471	H	0.70351	6.11441	-1.35018
H	0.96900	-1.16397	-2.77101	H	1.63396	5.74886	-2.82825
C	0.02696	1.33204	-3.33375	H	2.47687	6.26913	-1.34611
H	-0.35972	2.33202	-3.08031	C	1.24185	-1.68563	-0.01170
H	-0.14423	1.18252	-4.41650	C	-0.36934	-3.36868	1.08970
H	1.11416	1.32484	-3.15268	C	-0.56049	-3.20466	2.49242
C	-2.88995	-0.63533	-3.39649	C	0.47631	-2.53104	3.39376
H	-2.33286	-1.34203	-4.01898	H	1.12463	-1.90508	2.75538
C	-4.29152	-0.60474	-3.43167	C	-0.15979	-1.60775	4.45315
H	-4.84108	-1.29103	-4.08342	H	-0.83083	-0.86164	3.99612
C	-4.98425	0.30101	-2.62373	H	0.62914	-1.06651	5.00149
H	-6.07851	0.30117	-2.63887	H	-0.74037	-2.17417	5.20141
C	-4.31938	1.21652	-1.77654	C	1.36497	-3.59626	4.08288
C	-5.17187	2.12921	-0.88699	H	0.75665	-4.25922	4.72220
H	-4.50713	2.63948	-0.16844	H	2.12059	-3.10917	4.72242
C	-6.19475	1.32783	-0.04877	H	1.89675	-4.22625	3.35157
H	-6.95824	0.84515	-0.68191	C	-1.72102	-3.76887	3.05915
H	-6.72431	2.00560	0.64208	H	-1.89446	-3.66483	4.13430
H	-5.70316	0.54353	0.54841	C	-2.64598	-4.47213	2.27842
C	-5.90158	3.20823	-1.72357	H	-3.53933	-4.90144	2.74297
H	-5.21047	3.81950	-2.33033	C	-1.70353	4.14313	3.36360
H	-6.47246	3.88667	-1.06750	H	-1.21646	4.87929	4.02633
H	-6.61362	2.74178	-2.42591	H	-2.60629	3.76986	3.87730
C	-2.42075	3.50932	-0.99611	H	-2.02742	4.67201	2.45216
H	-3.27267	3.93733	-1.51300	C	-2.41637	-4.64800	0.90919
C	-1.44646	4.09582	-0.25009	H	-3.12722	-5.23029	0.31488
H	-1.29269	5.13000	0.04249	C	-1.26544	-4.12504	0.28512
C	0.61673	3.48205	0.90438	C	-0.97573	-4.47770	-1.17371
C	0.50749	3.48183	2.31940	H	-0.14543	-3.83741	-1.51898
C	-0.74003	2.97374	3.04351	C	-0.50222	-5.94844	-1.28552
H	-1.26332	2.27917	2.36108	H	0.39812	-6.13774	-0.67792
C	-0.40510	2.19293	4.33063	H	-0.26238	-6.19569	-2.33369
H	0.32699	1.39243	4.13835	H	-1.28897	-6.64332	-0.94491
H	-1.32020	1.73537	4.74337	C	-2.18610	-4.23458	-2.10089
H	0.00571	2.85061	5.11595	H	-3.02245	-4.91522	-1.86692
C	1.57253	4.04511	3.04882	H	-1.90145	-4.42340	-3.15018
H	1.52033	4.06736	4.14131	H	-2.56485	-3.20130	-2.02627
C	2.68835	4.59234	2.40215	C	1.97692	-3.80622	0.53459
H	3.49897	5.03281	2.99166	H	1.87945	-4.81912	0.91341
C	2.76072	4.58973	1.00494	C	3.04148	-3.13232	0.02387

```
H   4.07610 -3.42621 -0.12542
C   3.54759 -0.84786 -0.74261
C   4.15101 -0.03841  0.25683
C   3.85485 -0.21397  1.74641
H   2.93728 -0.81891  1.84426
C   3.59563  1.11994  2.46880
H   4.48371  1.77477  2.46075
H   3.34226  0.93217  3.52634
H   2.76132  1.66776  2.00566
C   5.01013 -0.99288  2.42267
H   5.17382 -1.97706  1.95239
H   4.78846 -1.15846  3.49096
H   5.95696 -0.42928  2.35816
C   5.15111  0.86329 -0.15518
H   5.63470  1.50041  0.59170
C   5.55899  0.93396 -1.49261
H   6.34352  1.63742 -1.78948
C   4.98899  0.08152 -2.44531
H   5.34814  0.11310 -3.47864
C   3.98551 -0.84418 -2.09438
C   3.51567 -1.86703 -3.13193
H   2.62181 -2.37731 -2.72998
C   3.13663 -1.22847 -4.48649
H   2.38766 -0.42720 -4.37481
H   2.72179 -1.99422 -5.16361
H   4.01649 -0.79325 -4.98994
C   4.60283 -2.94962 -3.34863
H   5.52845 -2.50133 -3.74846
H   4.25327 -3.70747 -4.07040
H   4.86273 -3.46835 -2.41117
C  -3.78063 -0.90652  2.15317
H  -4.21092  0.08424  2.36575
H  -3.54142 -1.41720  3.09716
H  -4.50140 -1.51145  1.58239
H   0.51331  0.44417  1.11287
```

[5]$^{+}$

159

SCF(BS1) = -3344.71220724

H(0 K) = -3343.310393

G(298 K) = -3343.514853

SCF(BS1)+D3(BJ) = -3345.19354626

SCF(PCM=THF) = -3344.76737298

SCF(PCM=C6H5F) = -3344.76322394

SCF(BS2) = -6450.46855468

Lowest Freq. = 18.5368cm-1, 21.9220cm-1, 24.5938cm-1

```
Ir  0.00000  0.00000  0.00000
H  -1.64816  0.27481 -0.21859
H  -0.00899 -1.61771  0.47252
Zn  1.94053  1.44044 -0.65905
N  -0.43365 -0.02047 -3.12916
N   0.94343 -1.61091 -2.54897
C   0.13873 -0.61289 -2.00751
C  -1.53145  0.93933 -3.18351
C  -2.85362  0.42544 -3.26345
C  -3.16328 -1.06997 -3.35332
H  -2.26678 -1.62699 -3.02639
C  -3.45333 -1.46697 -4.82241
H  -4.34591 -0.93877 -5.19966
H  -3.64288 -2.55127 -4.90035
H  -2.61240 -1.22009 -5.49086
C  -4.33419 -1.50659 -2.45018
H  -4.16258 -1.24699 -1.39244
H  -4.47408 -2.59889 -2.51808
H  -5.28759 -1.04322 -2.75624
C  -3.90718  1.35489 -3.36976
H  -4.93665  0.98972 -3.43643
C  -3.65999  2.73114 -3.41953
H  -4.49427  3.43466 -3.50630
C  -2.34422  3.20515 -3.39129
H  -2.15687  4.27998 -3.46666
C  -1.24988  2.32426 -3.28571
C   0.17178  2.86880 -3.38014
H   0.85013  2.11657 -2.93422
C   0.59351  3.04352 -4.85965
H   0.53121  2.09731 -5.42276
H   1.63307  3.40809 -4.91791
H  -0.05712  3.77831 -5.36470
C   0.36907  4.19001 -2.61134
H  -0.14405  5.03080 -3.10941
H   1.44163  4.44016 -2.56806
H  -0.01829  4.12189 -1.58109
C   0.02468 -0.61537 -4.30642
H  -0.31509 -0.28001 -5.28167
C   0.88458 -1.60832 -3.94413
H   1.45161 -2.31918 -4.53755
C   1.62357 -2.70036 -1.85605
C   0.91119 -3.91709 -1.67992
C  -0.49792 -4.14600 -2.23009
H  -0.94322 -3.16033 -2.45610
C  -0.42970 -4.94602 -3.55503
H   0.18763 -4.43769 -4.31355
H  -1.44025 -5.08449 -3.97614
H   0.00638 -5.94539 -3.38443
C  -1.43467 -4.85935 -1.23449
H  -1.09517 -5.88474 -1.00942
```

H	-2.44601	-4.94627	-1.66686
H	-1.51516	-4.31758	-0.27769
C	1.59085	-4.98465	-1.06058
H	1.07003	-5.93587	-0.91419
C	2.92430	-4.86023	-0.65556
H	3.43333	-5.70474	-0.17996
C	3.61550	-3.66660	-0.88807
H	4.66779	-3.58858	-0.60050
C	2.98934	-2.56482	-1.50356
C	3.80853	-1.32552	-1.84959
H	3.10345	-0.48307	-1.98351
C	4.55041	-1.51807	-3.19490
H	5.26426	-2.35726	-3.12800
H	5.11566	-0.60601	-3.45104
H	3.85581	-1.73366	-4.02395
C	4.80643	-0.92796	-0.74424
H	4.32042	-0.88274	0.24461
H	5.23837	0.06077	-0.96946
H	5.64383	-1.64373	-0.67588
C	3.41533	2.42662	-1.46961
C	3.72916	2.19877	-2.83377
H	3.14970	1.47663	-3.42246
C	4.77987	2.88214	-3.47047
H	4.99863	2.68483	-4.52577
C	5.54685	3.81550	-2.75464
H	6.36592	4.34957	-3.24727
C	5.25574	4.05861	-1.40356
H	5.84855	4.78505	-0.83691
C	4.20363	3.37193	-0.77222
H	4.00298	3.58523	0.28255
H	1.64816	-0.27481	0.21859
H	0.00899	1.61771	-0.47252
Zn	-1.94053	-1.44044	0.65905
N	0.43365	0.02047	3.12916
N	-0.94343	1.61091	2.54897
C	-0.13873	0.61289	2.00751
C	1.53145	-0.93933	3.18351
C	2.85362	-0.42544	3.26345
C	3.16328	1.06997	3.35332
H	2.26678	1.62699	3.02639
C	3.45333	1.46697	4.82241
H	4.34591	0.93877	5.19966
H	3.64288	2.55127	4.90035
H	2.61240	1.22009	5.49086
C	4.33419	1.50659	2.45018
H	4.16258	1.24699	1.39244
H	4.47408	2.59889	2.51808
H	5.28759	1.04322	2.75624
C	3.90718	-1.35489	3.36976
H	4.93665	-0.98972	3.43643
C	3.65999	-2.73114	3.41953
H	4.49427	-3.43466	3.50630
C	2.34422	-3.20515	3.39129
H	2.15687	-4.27998	3.46666
C	1.24988	-2.32426	3.28571
C	-0.17178	-2.86880	3.38014
H	-0.85013	-2.11657	2.93422
C	-0.59351	-3.04352	4.85965
H	-0.53121	-2.09731	5.42276
H	-1.63307	-3.40809	4.91791
H	0.05712	-3.77831	5.36470
C	-0.36907	-4.19001	2.61134
H	0.14405	-5.03080	3.10941
H	-1.44163	-4.44016	2.56806
H	0.01829	-4.12189	1.58109
C	-0.02468	0.61537	4.30642
H	0.31509	0.28001	5.28167
C	-0.88458	1.60832	3.94413
H	-1.45161	2.31918	4.53755
C	-1.62357	2.70036	1.85605
C	-0.91119	3.91709	1.67992
C	0.49792	4.14600	2.23009
H	0.94322	3.16033	2.45610
C	0.42970	4.94602	3.55503
H	-0.18763	4.43769	4.31355
H	1.44025	5.08449	3.97614
H	-0.00638	5.94539	3.38443
C	1.43467	4.85935	1.23449
H	1.09517	5.88474	1.00942
H	2.44601	4.94627	1.66686
H	1.51516	4.31758	0.27769
C	-1.59085	4.98465	1.06058
H	-1.07003	5.93587	0.91419
C	-2.92430	4.86023	0.65556
H	-3.43333	5.70474	0.17996
C	-3.61550	3.66660	0.88807
H	-4.66779	3.58858	0.60050
C	-2.98934	2.56482	1.50356
C	-3.80853	1.32552	1.84959
H	-3.10345	0.48307	1.98351
C	-4.55041	1.51807	3.19490
H	-5.26426	2.35726	3.12800
H	-5.11566	0.60601	3.45104
H	-3.85581	1.73366	4.02395
C	-4.80643	0.92796	0.74424
H	-4.32042	0.88274	-0.24461
H	-5.23837	-0.06077	0.96946
H	-5.64383	1.64373	0.67588

```
C  -3.41533 -2.42662  1.46961
C  -3.72916 -2.19877  2.83377
H  -3.14970 -1.47663  3.42246
C  -4.77987 -2.88214  3.47047
H  -4.99863 -2.68483  4.52577
C  -5.54685 -3.81550  2.75464
H  -6.36592 -4.34957  3.24727
C  -5.25574 -4.05861  1.40356
H  -5.84855 -4.78505  0.83691
C  -4.20363 -3.37193  0.77222
H  -4.00298 -3.58523 -0.28255
```

[10]$^+$

137
SCF(BS1) = -2632.86163438
H(0 K) = -2631.640497
G(298 K) = -2631.818201
SCF(BS1)+D3(BJ) = -2633.25968379
SCF(PCM=THF) = -2632.91299504
SCF(PCM=C6H5F) = -2632.90951419
SCF(BS2) = -2633.67091768
Lowest Freq. = 9.1405cm-1, 19.1248cm-1, 23.5773cm-1

```
Ir  0.10540  0.03445 -0.38415
Cd -1.94122 -0.92143  1.21480
N  -2.41462  1.58822 -1.33770
N  -1.26088  2.88922 -0.02676
N   1.43468 -2.65896  0.66326
N   2.95377 -1.33981 -0.15860
C  -1.26647  1.60559 -0.54704
C  -2.83130  0.49990 -2.18097
C  -1.81634 -0.16586 -2.91259
C  -0.37008  0.19101 -2.72958
C   0.55843 -0.82500 -2.47848
H   0.24232 -1.87052 -2.45604
H   1.61232 -0.63457 -2.68420
C   0.08304  1.49582 -3.36712
H  -0.57051  2.34348 -3.10859
H   0.03742  1.38085 -4.46657
H   1.11580  1.74297 -3.07968
C  -2.18663 -1.16446 -3.82861
H  -1.40914 -1.65975 -4.41824
C  -3.53641 -1.50941 -3.99536
H  -3.82153 -2.27493 -4.72359
C  -4.51339 -0.88677 -3.21375
H  -5.55982 -1.18828 -3.32480
C  -4.19290  0.11779 -2.27217
C  -5.31376  0.68584 -1.39555
H  -4.86671  1.37049 -0.65408
C  -6.02063 -0.43345 -0.59539
H  -6.53620 -1.14834 -1.25874
H  -6.78027  0.00387  0.07448
H  -5.30473 -1.00012  0.02326
C  -6.34010  1.48875 -2.22881
H  -5.87047  2.31179 -2.79543
H  -7.11395  1.92312 -1.57392
H  -6.84827  0.84069 -2.96331
C  -3.05238  2.83278 -1.34390
H  -3.92529  3.03275 -1.95675
C  -2.33707  3.63925 -0.51489
H  -2.47608  4.67765 -0.22997
C  -0.32062  3.54638  0.87194
C  -0.56849  3.49632  2.26886
C  -1.73613  2.71243  2.86962
H  -1.98510  1.88951  2.17336
C  -1.39654  2.07896  4.23402
H  -0.45438  1.50893  4.19582
H  -2.20534  1.39570  4.54279
H  -1.30025  2.84026  5.02722
C   0.25684  4.27783  3.10085
H   0.09283  4.26336  4.18212
C   1.26462  5.09243  2.56993
H   1.88388  5.70056  3.23714
C   1.46983  5.13883  1.18721
H   2.24748  5.79103  0.77690
C   0.67981  4.37596  0.30366
C   0.88254  4.52735 -1.20300
H   0.24226  3.78466 -1.70608
C   2.33780  4.23810 -1.62683
H   3.04060  4.97388 -1.19874
H   2.43210  4.29888 -2.72490
H   2.65961  3.23497 -1.30386
C   0.44058  5.93150 -1.68010
H  -0.61401  6.13780 -1.43085
H   0.55485  6.01945 -2.77411
H   1.05346  6.72217 -1.21392
C   1.58481 -1.40271  0.08122
C   0.25229 -3.28549  1.22922
C   0.01924 -3.13743  2.62555
C   0.96210 -2.35528  3.54308
H   1.58510 -1.69756  2.91159
C   0.21570 -1.45907  4.55188
H  -0.48913 -0.77815  4.04788
H   0.93779 -0.84708  5.11809
H  -0.35409 -2.05090  5.28877
C   1.90538 -3.32752  4.29465
H   1.32835 -4.01366  4.93841
```

```
H   2.60233 -2.76453  4.93832
H   2.50501 -3.94056  3.60240
C  -1.07899 -3.83198  3.17601
H  -1.28546 -3.74123  4.24652
C  -1.89548 -4.64767  2.38364
H  -2.74011 -5.17825  2.83457
C  -2.99054  3.61130  3.00444
H  -2.78459  4.46626  3.67149
H  -3.82978  3.03879  3.43569
H  -3.31962  4.01265  2.03217
C  -1.61573 -4.80676  1.02033
H  -2.23811 -5.47531  0.41747
C  -0.52846 -4.14621  0.41372
C  -0.17720 -4.43967 -1.04360
H   0.56928 -3.69235 -1.36518
C   0.48131 -5.83613 -1.16427
H   1.38771 -5.91711 -0.54137
H   0.76843 -6.03674 -2.21058
H  -0.21597 -6.63047 -0.84666
C  -1.39415 -4.32866 -1.98525
H  -2.15705 -5.09382 -1.76101
H  -1.07829 -4.48697 -3.03072
H  -1.87364 -3.33838 -1.91604
C   2.65543 -3.33246  0.76433
H   2.72117 -4.32914  1.18982
C   3.60533 -2.50346  0.25570
H   4.68016 -2.61784  0.15613
C   3.75329 -0.22433 -0.64470
C   4.10912  0.78837  0.28530
C   3.71572  0.71795  1.76121
H   2.83580  0.05752  1.84706
C   3.31156  2.08170  2.34979
H   4.16206  2.78429  2.39057
H   2.95465  1.95131  3.38534
H   2.50502  2.54899  1.76495
C   4.87015  0.09015  2.58181
H   5.13047 -0.91988  2.22334
H   4.58776  0.01116  3.64586
H   5.77891  0.71357  2.51892
C   4.94853  1.82174 -0.17217
H   5.23956  2.61801  0.51926
C   5.43555  1.83601 -1.48525
H   6.08854  2.64911 -1.81838
C   5.11564  0.79353 -2.36124
H   5.53729  0.79148 -3.37107
C   4.28402 -0.27312 -1.96159
C   4.09299 -1.46223 -2.90994
H   3.26182 -2.08122 -2.52594
C   3.74650 -1.04048 -4.35623
H   2.88002 -0.36026 -4.40091
H   3.51402 -1.93179 -4.96312
H   4.59430 -0.53010 -4.84368
C   5.36444 -2.34839 -2.92598
H   6.23166 -1.77903 -3.30190
H   5.21818 -3.21991 -3.58673
H   5.62479 -2.72428 -1.92307
C  -3.51055 -1.01842  2.65301
H  -4.20200 -0.16993  2.53082
H  -3.08844 -0.99517  3.66942
H  -4.06830 -1.95891  2.52220
H   0.30039  0.49833  1.14711
H  -1.07929 -1.25698 -0.43919
H   1.29917  1.10163 -0.58750
```

[10b]⁺

```
137
SCF(BS1) = -2632.85276270
H(0 K) = -2631.631511
G(298 K) = -2631.808590
SCF(BS1)+D3(BJ) = -2633.25179883
SCF(PCM=THF) = -2632.90282236
SCF(PCM=C6H5F) = -2632.89939035
SCF(BS2) = -2633.66167777
Lowest Freq. = 13.1832cm-1, 19.6936cm-1, 22.6155cm-1

Ir  0.10834  0.05976 -0.26733
Cd -2.72245 -0.14996  0.51831
N  -1.07252  2.88084 -0.92329
N   0.86845  3.11270  0.02687
N  -0.20098 -2.98695  0.65780
N   1.79129 -2.61470 -0.12171
C  -0.03614  2.13798 -0.35285
C  -2.26693  2.35244 -1.51901
C  -2.13336  1.17283 -2.29776
C  -0.81090  0.47548 -2.43601
C  -0.74936 -0.90553 -2.21294
H  -1.65405 -1.46604 -1.96584
H   0.06261 -1.48405 -2.65999
C   0.18587  1.16439 -3.35891
H   0.33518  2.22391 -3.09774
H  -0.20868  1.13654 -4.39214
H   1.16353  0.66172 -3.33611
C  -3.25786  0.68501 -2.98990
H  -3.14753 -0.20347 -3.61921
C  -4.48923  1.35099 -2.90380
H  -5.35302  0.98384 -3.46644
C  -4.61306  2.47372 -2.08028
```

H	-5.58757	2.96350	-1.98757
C	-3.52407	2.98957	-1.34129
C	-3.78638	4.14944	-0.37317
H	-2.85970	4.35372	0.18937
C	-4.86149	3.77226	0.67322
H	-5.84599	3.59797	0.20711
H	-4.98003	4.59206	1.40181
H	-4.58403	2.85839	1.22434
C	-4.18713	5.44258	-1.12106
H	-3.42917	5.75568	-1.86041
H	-4.33022	6.27111	-0.40745
H	-5.13497	5.30692	-1.66962
C	-0.77409	4.24818	-0.94481
H	-1.42256	4.97542	-1.42154
C	0.43216	4.39105	-0.33675
H	1.03325	5.27181	-0.13305
C	2.15253	3.00868	0.70228
C	2.17450	3.10528	2.11742
C	0.89664	3.15253	2.95626
H	0.07127	2.76279	2.33449
C	0.98314	2.25987	4.21183
H	1.25728	1.22514	3.94882
H	0.00868	2.24049	4.72845
H	1.72561	2.63671	4.93614
C	3.43416	3.22840	2.73570
H	3.48923	3.31718	3.82495
C	4.61468	3.25209	1.98183
H	5.58140	3.35721	2.48490
C	4.55922	3.15053	0.58717
H	5.48579	3.18242	0.00495
C	3.32759	3.04411	-0.08941
C	3.29646	3.05573	-1.61754
H	2.25754	2.86454	-1.93588
C	4.17066	1.94179	-2.22791
H	5.23783	2.07879	-1.98183
H	4.08519	1.95451	-3.32836
H	3.86480	0.94810	-1.86527
C	3.71239	4.44216	-2.16505
H	3.06609	5.24870	-1.77959
H	3.65099	4.45505	-3.26677
H	4.75157	4.68516	-1.88355
C	0.58008	-1.97074	0.10555
C	-1.57629	-2.99417	1.13121
C	-1.83632	-2.59237	2.47289
C	-0.74006	-2.05449	3.39384
H	0.06544	-1.64719	2.75800
C	-1.22737	-0.91082	4.30670
H	-1.66827	-0.08397	3.72422
H	-0.37855	-0.50437	4.88036
H	-1.97732	-1.25304	5.04026
C	-0.14803	-3.20556	4.24533
H	-0.92250	-3.65445	4.89114
H	0.65715	-2.82509	4.89667
H	0.27499	-4.00625	3.61661
C	-3.14130	-2.78985	2.96761
H	-3.37340	-2.49156	3.99402
C	-4.13555	-3.39279	2.18590
H	-5.13551	-3.55216	2.60148
C	0.55231	4.60948	3.35019
H	1.35694	5.05109	3.96333
H	-0.37937	4.64082	3.94112
H	0.41387	5.25164	2.46435
C	-3.84786	-3.79812	0.87901
H	-4.62685	-4.28100	0.28096
C	-2.56664	-3.61383	0.31777
C	-2.28575	-4.14461	-1.09045
H	-1.30858	-3.74622	-1.41644
C	-2.18675	-5.69021	-1.09078
H	-1.38637	-6.05407	-0.42661
H	-1.97525	-6.06023	-2.10836
H	-3.13477	-6.14531	-0.75620
C	-3.35163	-3.69095	-2.11443
H	-4.33075	-4.15978	-1.91753
H	-3.04862	-3.98795	-3.13270
H	-3.50263	-2.59791	-2.10396
C	0.51095	-4.18596	0.76831
H	0.05823	-5.07764	1.19085
C	1.75562	-3.95189	0.27730
H	2.62460	-4.59541	0.18091
C	3.06232	-2.08914	-0.59846
C	3.98453	-1.61804	0.37057
C	3.67983	-1.62773	1.86891
H	2.59443	-1.78412	1.99629
C	4.03082	-0.29595	2.55657
H	5.11405	-0.08765	2.52343
H	3.74089	-0.33288	3.62087
H	3.50782	0.54837	2.08379
C	4.41367	-2.80638	2.55473
H	4.13490	-3.77899	2.11579
H	4.17091	-2.83720	3.63078
H	5.50788	-2.69790	2.45855
C	5.26113	-1.23384	-0.08401
H	5.99544	-0.86383	0.63831
C	5.61597	-1.34754	-1.43336
H	6.61737	-1.05239	-1.76292
C	4.70116	-1.86597	-2.35803
H	5.00221	-1.98707	-3.40331
C	3.40832	-2.26364	-1.96325

```
C   2.49000 -2.95920 -2.96959
H   1.47885 -3.00767 -2.52666
C   2.38531 -2.20894 -4.31445
H   2.07878 -1.15924 -4.17739
H   1.64607 -2.70183 -4.96882
H   3.34623 -2.20769 -4.85646
C   2.95860 -4.41580 -3.21039
H   3.97490 -4.43425 -3.64003
H   2.28265 -4.92813 -3.91641
H   2.98021 -5.00068 -2.27602
C  -4.85280 -0.20863  0.56057
H  -5.25931  0.47650 -0.19732
H  -5.20825  0.09409  1.55847
H  -5.19349 -1.23447  0.35683
H   1.02083  0.32929  1.01924
H  -0.98623  0.03824  1.08516
H   1.52563  0.14355 -1.02200
```

[10c]⁺

```
137
SCF(BS1) = -2632.84501969
H(0 K) = -2631.624483
G(298 K) = -2631.799354
SCF(BS1)+D3(BJ) = -2633.24398544
SCF(PCM=THF) = -2632.89577681
SCF(PCM=C6H5F) = -2632.89240196
SCF(BS2) = -2633.65455471
Lowest  Freq.  =  13.1124cm-1,
25.1406cm-1, 27.3916cm-1

Ir -0.10977  0.04888 -0.42888
Cd  0.67592  0.20445  2.08675
N   0.79934  2.89973 -1.22725
N   2.58557  1.68579 -0.94726
N  -2.56187 -1.46246  0.93797
N  -0.89336 -2.82943  0.61233
C   1.20063  1.63232 -0.82322
C  -0.56487  3.32327 -1.39772
C  -1.43672  2.42049 -2.05645
C  -0.96508  1.05895 -2.48018
C  -1.71218 -0.06391 -2.10950
H  -2.64546  0.07081 -1.56122
H  -1.59399 -0.99829 -2.66524
C  -0.03901  1.01548 -3.68387
H   0.84456  1.66362 -3.57175
H  -0.59450  1.38553 -4.56652
H   0.29531 -0.01193 -3.89660
C  -2.75726  2.82462 -2.31177
H  -3.43246  2.14109 -2.83515
C  -3.20087  4.09318 -1.90834
H  -4.22667  4.40804 -2.12335
C  -2.33622  4.94749 -1.21871
H  -2.70098  5.92401 -0.88431
C  -1.00245  4.58311 -0.92598
C  -0.13645  5.53720 -0.09825
H   0.82842  5.04225  0.10841
C  -0.78463  5.83868  1.27306
H  -1.74103  6.37769  1.16331
H  -0.11450  6.47346  1.87740
H  -0.98225  4.91230  1.83725
C   0.15573  6.84971 -0.86237
H   0.64302  6.66824 -1.83626
H   0.81462  7.50464 -0.26775
H  -0.77499  7.40684 -1.06526
C   1.88451  3.67832 -1.64222
H   1.75582  4.67642 -2.04888
C   2.99917  2.92010 -1.46306
H   4.04617  3.13281 -1.65343
C   3.58250  0.69719 -0.56047
C   4.26152  0.87472  0.67718
C   4.02445  2.07423  1.60109
H   3.01723  2.47959  1.38727
C   4.08133  1.70921  3.09989
H   3.43236  0.85446  3.34385
H   3.75466  2.56986  3.70722
H   5.10831  1.46287  3.41969
C   5.26342 -0.06215  1.00429
H   5.80321  0.04306  1.94977
C   5.59350 -1.10987  0.13745
H   6.37654 -1.82282  0.41518
C   4.94687 -1.22623 -1.09815
H   5.24021 -2.02393 -1.78646
C   3.94187 -0.31866 -1.48571
C   3.36398 -0.37302 -2.89918
H   2.37668  0.11678 -2.87386
C   3.16140 -1.80564 -3.43167
H   4.12278 -2.31507 -3.61797
H   2.62911 -1.77301 -4.39782
H   2.57814 -2.43000 -2.73478
C   4.26322  0.43209 -3.87120
H   4.36479  1.48552 -3.56236
H   3.83949  0.41727 -4.89012
H   5.27583 -0.00447 -3.91799
C  -1.27856 -1.50841  0.38572
C  -3.58481 -0.42594  0.82848
C  -3.64424  0.61043  1.79390
C  -2.70589  0.67769  2.99509
H  -1.84845  0.01037  2.78931
```

C -2.15314 2.09623 3.24381
H -1.67195 2.50181 2.33729
H -1.40906 2.07664 4.05723
H -2.94891 2.79743 3.54761
C -3.41408 0.13523 4.26033
H -4.29054 0.75517 4.51667
H -2.72635 0.14744 5.12332
H -3.76586 -0.90044 4.11872
C -4.68539 1.55351 1.68116
H -4.75163 2.36384 2.41339
C -5.64829 1.45816 0.67201
H -6.45136 2.19937 0.60758
C 5.06046 3.19335 1.32088
H 6.08370 2.82451 1.50784
H 4.88273 4.05331 1.98896
H 5.02065 3.55998 0.28354
C -5.60319 0.39287 -0.23379
H -6.38407 0.30096 -0.99486
C -4.58628 -0.58068 -0.17467
C -4.66417 -1.78760 -1.11834
H -3.68532 -2.30015 -1.10607
C -5.73110 -2.79343 -0.61606
H -5.52402 -3.14620 0.40681
H -5.77006 -3.67564 -1.27752
H -6.73172 -2.32814 -0.61223
C -4.97580 -1.40624 -2.58343
H -5.99353 -0.99435 -2.69120
H -4.92555 -2.30452 -3.22170
H -4.26673 -0.66381 -2.98390
C -2.91537 -2.68981 1.50738
H -3.87190 -2.82922 2.00154
C -1.87929 -3.54539 1.29654
H -1.74632 -4.59167 1.55444
C 0.27563 -3.53298 0.10621
C 1.36743 -3.78289 0.97720
C 1.39070 -3.32854 2.43656
H 0.62378 -2.53941 2.56064
C 2.75283 -2.73351 2.85202
H 3.54802 -3.49843 2.85202
H 2.69133 -2.33283 3.87727
H 3.07176 -1.92008 2.17825
C 1.01152 -4.49640 3.38029
H 0.01437 -4.90727 3.15240
H 1.00588 -4.15664 4.42994
H 1.74078 -5.32035 3.29335
C 2.43651 -4.55303 0.47558
H 3.29121 -4.76513 1.12458
C 2.41695 -5.06747 -0.82481
H 3.25533 -5.67042 -1.18824
C 1.31351 -4.83072 -1.65322
H 1.29193 -5.26329 -2.65829
C 0.21530 -4.06845 -1.20900
C -1.01453 -3.92922 -2.10712
H -1.64734 -3.12472 -1.69333
C -0.65738 -3.53663 -3.55660
H -0.03502 -2.62622 -3.58935
H -1.57677 -3.35139 -4.13800
H -0.10430 -4.33716 -4.07679
C -1.84443 -5.23624 -2.08856
H -1.25487 -6.08202 -2.48244
H -2.74611 -5.13138 -2.71612
H -2.16791 -5.49836 -1.06764
C 0.95585 0.34899 4.21864
H 1.13071 1.39643 4.51259
H 1.80798 -0.25938 4.55856
H 0.04393 -0.01361 4.72203
H 1.23542 -1.02779 -0.52960
H -1.13029 1.07810 0.27413
H 0.85179 -1.07817 -1.37244

[11b]+
141
SCF(BS1) = -2694.67458120
H(0 K) = -2693.413848
G(298 K) = -2693.596767
SCF(BS1)+D3(BJ) = -2695.06809965
SCF(PCM=THF) = -2694.72002589
SCF(PCM=C6H5F) = -2694.71719643
SCF(BS2) = -4247.86811792
Lowest Freq. = 9.3321cm-1, 21.7667cm-1, 23.7186cm-1

Ir -0.00530 0.06305 0.02110
H 0.20918 -0.29356 -1.54621
H 0.92736 -1.21868 0.77567
H -0.16753 0.33123 1.76222
H -0.58796 1.00853 1.37385
H -0.82250 1.27427 -0.67821
Zn 1.08420 -2.11309 -0.74286
N 3.02286 0.92783 -0.31501
N 1.77853 2.63485 0.17365
N -2.28586 -1.83207 1.07433
N -2.77013 -1.16572 -0.93871
C 1.70393 1.26855 -0.04797
C 3.86604 2.04156 -0.26095
H 4.93320 1.95697 -0.44216
C 3.08359 3.11235 0.04523
H 3.32204 4.16234 0.18476

C	3.58967	-0.38368	-0.55967
C	4.03362	-1.14394	0.55834
C	4.68827	-2.36216	0.28880
H	5.04566	-2.97310	1.12365
C	4.92755	-2.78389	-1.02645
H	5.45411	-3.72611	-1.20939
C	4.51457	-1.99509	-2.10649
H	4.72931	-2.32417	-3.12766
C	3.83807	-0.77428	-1.90357
C	3.92926	-0.62532	1.99399
H	3.21542	0.21690	1.99944
C	5.30538	-0.08373	2.45600
H	6.05990	-0.88901	2.47434
H	5.23128	0.33378	3.47454
H	5.67915	0.71096	1.78920
C	3.40089	-1.67609	2.99084
H	2.39303	-2.02462	2.71360
H	3.33824	-1.23509	4.00007
H	4.06615	-2.55382	3.06161
C	3.48204	0.11802	-3.09395
H	2.76539	0.88096	-2.74176
C	0.70318	3.53001	0.55653
C	0.49861	3.77795	1.93889
C	-0.52644	4.68197	2.28358
H	-0.71435	4.90256	3.33918
C	-1.28373	5.32811	1.29819
H	-2.06883	6.03341	1.58942
C	-1.01799	5.10129	-0.05872
H	-1.59067	5.64286	-0.81785
C	-0.01414	4.20054	-0.46570
C	1.42053	3.20815	3.02187
H	2.01042	2.38855	2.57354
C	2.41590	4.30029	3.48817
H	3.00537	4.70510	2.64894
H	3.11974	3.89007	4.23236
H	1.88046	5.14418	3.95627
C	0.67197	2.62245	4.23665
H	0.06913	3.38575	4.75774
H	1.39748	2.22742	4.96791
H	-0.00014	1.79733	3.95087
C	0.34471	4.04798	-1.94493
H	0.94997	3.13071	-2.05147
C	1.21219	5.24717	-2.40238
H	1.50928	5.12708	-3.45830
H	2.13097	5.34594	-1.80015
H	0.65170	6.19396	-2.31376
C	-0.88324	3.89016	-2.86286
H	-1.52372	4.78923	-2.85523
H	-1.49823	3.02370	-2.57343
H	-0.55221	3.73800	-3.90423
C	-1.79494	-1.04258	0.04009
C	-3.51483	-2.40593	0.74432
H	-4.05470	-3.04004	1.44094
C	-3.81667	-1.98929	-0.51653
H	-4.67864	-2.17985	-1.14849
C	-1.74354	-1.95221	2.41685
C	-0.87046	-3.02899	2.71795
C	-0.36994	-3.10420	4.03268
H	0.30783	-3.91914	4.30282
C	-0.73857	-2.16605	5.00577
H	-0.33492	-2.24541	6.02035
C	-1.65198	-1.15181	4.69462
H	-1.97404	-0.45710	5.47681
C	-2.19072	-1.02768	3.39794
C	-0.56567	-4.13456	1.70556
H	-0.70293	-3.71225	0.69150
C	0.87211	-4.68370	1.79456
H	1.62729	-3.88448	1.71178
H	1.04861	-5.40352	0.97858
H	1.04903	-5.21974	2.74273
C	-1.57889	-5.29664	1.86542
H	-1.49660	-5.74672	2.86979
H	-1.38017	-6.08593	1.12049
H	-2.61990	-4.96056	1.73243
C	-3.30837	-0.01546	3.12865
H	-3.45223	0.05681	2.03623
C	-3.00698	1.40662	3.64306
H	-2.86248	1.43005	4.73676
H	-3.85531	2.07338	3.41365
H	-2.10874	1.83661	3.17143
C	-4.63385	-0.53385	3.74175
H	-4.90197	-1.53177	3.35707
H	-5.46266	0.15592	3.50825
H	-4.55503	-0.61050	4.83991
C	-2.90078	-0.42508	-2.18508
C	-3.63266	0.78995	-2.15133
C	-3.82110	1.46573	-3.37359
H	-4.38431	2.40429	-3.38333
C	-3.31977	0.94837	-4.57280
H	-3.47845	1.48931	-5.51129
C	-2.63851	-0.27518	-4.57899
H	-2.27917	-0.68289	-5.52778
C	-2.42430	-1.00102	-3.39055
C	-4.29682	1.32727	-0.88186
H	-3.90316	0.75831	-0.02155
C	-3.98649	2.81470	-0.61902
H	-2.90327	2.99297	-0.53144
H	-4.46330	3.13687	0.32257

```
H  -4.37885  3.46426 -1.42052
C  -5.82650  1.09423 -0.94148
H  -6.27736  1.64991 -1.78174
H  -6.30670  1.44227 -0.01090
H  -6.07596  0.02837 -1.07557
C  -1.78471 -2.39010 -3.43759
H  -1.26564 -2.55603 -2.47441
C  -0.74479 -2.54375 -4.56505
H  -1.22232 -2.56956 -5.55982
H  -0.20025 -3.49485 -4.44657
H  -0.01181 -1.72129 -4.56405
C  -2.86608 -3.49055 -3.58699
H  -3.57907 -3.49492 -2.74749
H  -2.39435 -4.48724 -3.63163
H  -3.43936 -3.34403 -4.51869
C   1.44462 -3.75994 -1.70697
H   2.30815 -4.27237 -1.25467
H   1.67839 -3.54784 -2.76184
H   0.57171 -4.43254 -1.66624
C   4.74181  0.85123 -3.61707
H   5.21350  1.47206 -2.83775
H   4.47815  1.51078 -4.46120
H   5.49655  0.12929 -3.97399
C   2.80395 -0.65985 -4.23971
H   3.49096 -1.38173 -4.71387
H   2.47218  0.03877 -5.02608
H   1.92370 -1.21649 -3.88033
```

[14c]⁺

155
SCF(BS1) = -2859.11097430
H(0 K) = -2857.717675
G(298 K) = -2857.909232
SCF(BS1)+D3(BJ) = -2859.57503198
SCF(PCM=THF) = -2859.15508611
SCF(PCM=C6H5F) = -2859.15225705
SCF(BS2) = -4747.18594049
Lowest Freq. = 17.6957cm-1, 26.2387cm-1, 29.0649cm-1

```
Ir -0.01104 -0.01536 -0.25221
H   0.59854 -1.51460 -0.38572
H   0.51333 -0.23934  1.28384
H  -0.70166  1.58199 -0.31029
Zn -0.39890  1.76181  1.49926
P  -0.50017 -0.21834 -2.61406
N   3.04893 -0.05769 -1.11193
N   2.35581  2.00502 -0.87064
N  -1.86963 -1.82999  1.49585
N  -3.19243 -0.54434  0.34087
C   1.91711  0.68103 -0.72665
C   3.31601 -1.48689 -0.95101
C   3.57137 -2.30418 -2.08759
C   3.80952 -1.77898 -3.50846
H   3.52930 -0.71261 -3.54593
C   5.32208 -1.85046 -3.84312
H   5.93868 -1.31195 -3.10329
H   5.51748 -1.41200 -4.83665
H   5.67339 -2.89612 -3.85853
C   3.00189 -2.52811 -4.59068
H   3.31535 -3.58249 -4.67107
H   3.16693 -2.06390 -5.57777
H   1.91998 -2.51719 -4.38355
C   3.78548 -3.68187 -1.86518
H   3.96871 -4.33398 -2.72488
C   3.80396 -4.22180 -0.57786
H   3.95939 -5.29571 -0.43317
C   3.69500 -3.37021  0.52809
H   3.79681 -3.78290  1.53446
C   3.49150 -1.98703  0.37254
C   3.65956 -1.05852  1.57747
H   2.94699 -0.22185  1.47387
C   3.39226 -1.72957  2.93491
H   4.16220 -2.48320  3.17782
H   2.40729 -2.21737  2.96864
H   3.42966 -0.97109  3.73465
C   5.10478 -0.49180  1.57595
H   5.83679 -1.31444  1.65346
H   5.25598  0.17415  2.44326
H   5.33639  0.08211  0.66431
C   4.09193  0.78170 -1.51583
H   5.04564  0.38396 -1.84928
C   3.66170  2.06209 -1.36893
H   4.16623  3.00936 -1.53374
C   1.81475  3.22357 -0.26929
C   0.93467  4.06653 -1.00331
C   1.63206  4.16230 -3.43418
H   1.81541  5.24911 -3.37699
H   1.32891  3.92739 -4.46945
H   2.58531  3.64638 -3.23759
C  -0.81346  4.38904 -2.86679
H  -1.63576  4.15024 -2.17259
H  -1.09547  4.02730 -3.87022
H  -0.73604  5.48752 -2.94156
C   0.52030  5.26594 -0.39377
H  -0.16463  5.92812 -0.92964
C   0.98479  5.64237  0.87346
H   0.65148  6.58411  1.32124
```

C	1.89792	4.82928	1.54847
H	2.29068	5.14751	2.51919
C	2.34694	3.61133	0.99489
C	3.46570	2.85085	1.71442
H	3.63300	1.89636	1.19013
C	4.78483	3.65994	1.63955
H	4.69661	4.61536	2.18476
H	5.06620	3.89563	0.59986
H	5.61089	3.08733	2.09430
C	3.13486	2.51212	3.18261
H	3.98614	1.98645	3.64780
H	2.24956	1.86064	3.25877
H	2.94255	3.41694	3.78424
C	0.52003	3.74668	-2.43757
H	0.39772	2.65118	-2.48774
C	-0.62694	2.86576	3.11954
H	0.29850	2.74001	3.70853
H	-0.61230	3.91172	2.76146
C	-1.85091	2.62899	4.02260
H	-1.83631	3.29691	4.90481
H	-1.89954	1.59500	4.40511
H	-2.80118	2.81918	3.49725
C	0.87482	0.23369	-3.78527
H	1.09484	1.30896	-3.72729
H	0.58245	-0.01885	-4.81759
H	1.77818	-0.32122	-3.51607
C	-1.86621	0.74329	-3.43421
H	-2.84401	0.47938	-3.01718
H	-1.85976	0.54423	-4.51911
H	-1.69225	1.81500	-3.25983
C	-0.81252	-1.97250	-3.14814
H	0.07636	-2.56776	-2.88942
H	-1.00632	-2.03690	-4.23169
H	-1.66910	-2.38209	-2.59511
C	-1.82970	-0.86732	0.48352
C	-0.82947	-2.67369	2.07084
C	-0.46370	-3.85950	1.38076
C	-1.02930	-4.21455	0.00606
H	-1.34033	-3.27192	-0.47614
C	0.00420	-4.88353	-0.92139
H	0.92061	-4.27829	-1.01229
H	-0.42842	-5.01955	-1.92757
H	0.29219	-5.88703	-0.56272
C	-2.27977	-5.11602	0.15871
H	-2.01749	-6.06778	0.65265
H	-2.70635	-5.35701	-0.83074
H	-3.06667	-4.63049	0.75971
C	0.36448	-4.77557	2.05706
H	0.66030	-5.69963	1.55179
C	0.77798	-4.54937	3.37559
H	1.39873	-5.28986	3.89047
C	0.38561	-3.38167	4.03837
H	0.70331	-3.21506	5.07271
C	-0.42200	-2.41519	3.40445
C	-0.85555	-1.17591	4.18919
H	-1.38313	-0.49873	3.49299
C	0.35560	-0.40292	4.75510
H	0.91741	-1.00601	5.48869
H	0.01792	0.51232	5.26942
H	1.05226	-0.11165	3.95255
C	-1.83455	-1.54419	5.32944
H	-2.73733	-2.05350	4.95362
H	-2.15831	-0.63691	5.86781
H	-1.35409	-2.21539	6.06208
C	-3.16410	-2.04875	1.96921
H	-3.36465	-2.76345	2.76125
C	-3.98728	-1.23949	1.25682
H	-5.06072	-1.08637	1.30167
C	-3.91940	0.21676	-0.67086
C	-4.57567	-0.51134	-1.70870
C	-4.51129	-2.03588	-1.85899
H	-3.63677	-2.40147	-1.28981
C	-4.35759	-2.48883	-3.33055
H	-5.29430	-2.34624	-3.89587
H	-4.12323	-3.56596	-3.37032
H	-3.56177	-1.94300	-3.86043
C	-5.77792	-2.70767	-1.26904
H	-5.90233	-2.50554	-0.19447
H	-5.72471	-3.80178	-1.40086
H	-6.68266	-2.34782	-1.78865
C	-5.37040	0.21838	-2.61629
H	-5.88048	-0.31445	-3.42404
C	-5.51844	1.60554	-2.50830
H	-6.12974	2.15108	-3.23429
C	-4.91852	2.28418	-1.44370
H	-5.09213	3.35799	-1.32518
C	-4.14365	1.60444	-0.48114
C	-3.75501	2.33955	0.79935
H	-3.00637	1.72683	1.33616
C	-3.14307	3.73212	0.55510
H	-2.24294	3.67199	-0.07856
H	-2.85148	4.19071	1.51419
H	-3.85966	4.41838	0.07223
C	-5.00994	2.45011	1.70384
H	-5.80272	3.02870	1.19911
H	-4.76787	2.96269	2.65006
H	-5.42439	1.45876	1.95154

[15b]
140
SCF(BS1) = -2694.24722524
H(0 K) = -2692.998590
G(298 K) = -2693.180983
SCF(BS1)+D3(BJ) = -2694.63869152
SCF(PCM=THF) = -2694.26319273
SCF(PCM=C6H5F) = -2694.26125818
SCF(BS2) = -4247.44791876
Lowest Freq. = 12.5358cm-1, 19.1061cm-1, 22.4832cm-1

Ir	0.01096	0.00587	0.05689
H	1.58350	-0.14553	0.47909
H	-0.23136	-0.30402	1.66023
H	-1.69921	0.10439	0.01051
H	0.67772	0.25079	-1.48957
Zn	-1.38063	0.28853	-1.93993
N	-0.99498	-2.96754	0.17068
N	0.85459	-2.82698	-0.96180
N	-0.77149	3.01098	0.58900
N	1.31059	2.64726	1.08550
C	3.09804	1.77134	2.53671
C	-2.41774	0.29688	-3.61198
H	-3.35328	0.86693	-3.48046
H	-2.68374	-0.73365	-3.90920
H	-1.85307	0.74872	-4.44529
C	-0.05186	-2.02823	-0.25897
C	-2.15793	-2.79074	1.02082
C	-3.43678	-2.68043	0.41795
C	-3.62620	-2.57363	-1.09482
H	-2.65766	-2.26935	-1.53134
C	-4.01221	-3.94226	-1.70544
H	-3.24330	-4.70856	-1.51196
H	-4.13693	-3.85596	-2.79904
H	-4.96589	-4.30521	-1.28242
C	-4.66597	-1.50036	-1.48019
H	-5.68360	-1.78086	-1.15540
H	-4.69352	-1.37713	-2.57544
H	-4.41867	-0.52306	-1.03441
C	-4.56491	-2.72023	1.26189
H	-5.56414	-2.64670	0.82110
C	-4.43128	-2.86319	2.64761
H	-5.32217	-2.90040	3.28380
C	-3.15700	-2.95478	3.21860
H	-3.05869	-3.06712	4.30315
C	-1.99473	-2.92540	2.42340
C	-0.62423	-3.10710	3.07451
H	0.13691	-2.78450	2.34386
C	-0.37490	-4.59568	3.41626
H	-1.12586	-4.96647	4.13662
H	0.62311	-4.72692	3.87011
H	-0.42514	-5.23356	2.51790
C	-0.44894	-2.22348	4.32579
H	-0.61046	-1.16206	4.07827
H	0.57479	-2.33210	4.72242
H	-1.14388	-2.50613	5.13639
C	-0.67327	-4.26404	-0.24880
H	-1.29831	-5.11486	0.00543
C	0.48369	-4.17527	-0.95855
H	1.08434	-4.93227	-1.45401
C	2.12444	-2.44719	-1.54842
C	3.29030	-2.56986	-0.75149
C	3.24164	-3.01753	0.70900
H	2.19080	-2.96769	1.04077
C	3.71772	-4.48375	0.84415
H	4.76710	-4.59047	0.51625
H	3.10486	-5.17052	0.23606
H	3.65719	-4.81504	1.89569
C	4.05246	-2.08966	1.63457
H	5.13203	-2.11550	1.40271
H	3.93680	-2.41023	2.68489
H	3.70614	-1.04836	1.54888
C	4.53281	-2.32678	-1.36890
H	5.44928	-2.41440	-0.77611
C	4.61488	-1.99025	-2.72467
H	5.59152	-1.81190	-3.18761
C	3.44779	-1.88735	-3.49178
H	3.52163	-1.63175	-4.55347
C	2.17826	-2.11387	-2.92477
C	0.92569	-2.05879	-3.79828
H	0.05816	-1.93521	-3.12555
C	0.73922	-3.38422	-4.57543
H	1.59517	-3.56734	-5.24920
H	-0.17651	-3.34641	-5.19117
H	0.65469	-4.24816	-3.89513
C	0.92827	-0.85974	-4.76814
H	1.08345	0.08707	-4.22571
H	-0.03891	-0.79952	-5.29448
H	1.71577	-0.95186	-5.53697
C	0.19004	1.99147	0.56798
C	-2.11019	2.98131	0.04200
C	-2.32881	3.57651	-1.22835
C	-1.21592	4.26599	-2.02410
H	-0.25128	4.01091	-1.55264
C	-1.14443	3.80603	-3.49521
H	-1.00302	2.71643	-3.57017
H	-0.29950	4.30159	-4.00423

H	-2.06178	4.06490	-4.05244
C	-1.38112	5.80399	-1.95567
H	-2.33154	6.11852	-2.42192
H	-0.55783	6.30586	-2.49335
H	-1.38254	6.17218	-0.91619
C	-3.65054	3.59137	-1.72180
H	-3.85082	4.04042	-2.70025
C	-4.70515	3.04998	-0.97972
H	-5.72350	3.06787	-1.38294
C	-4.46387	2.49849	0.28711
H	-5.29972	2.09498	0.86604
C	-3.16714	2.45954	0.83229
C	-2.91417	1.94063	2.24737
H	-1.90250	1.49793	2.25246
C	-2.95030	3.10863	3.26419
H	-2.19784	3.88100	3.03400
H	-2.74820	2.73480	4.28344
H	-3.94375	3.59221	3.27169
C	-3.89181	0.83237	2.67548
H	-4.92355	1.20976	2.79876
H	-3.58140	0.41929	3.64981
H	-3.90442	0.00409	1.95007
C	-0.26237	4.20837	1.10256
H	-0.87821	5.09820	1.19188
C	1.04261	3.98130	1.40984
H	1.80881	4.63122	1.82160
C	2.66591	2.16066	1.24480
C	3.55381	2.26119	0.14427
C	3.09474	2.74969	-1.22886
H	1.99323	2.69454	-1.25004
C	3.60732	1.85603	-2.37546
H	4.70800	1.88503	-2.46457
H	3.19428	2.20697	-3.33749
H	3.29768	0.80992	-2.22736
C	3.51095	4.22437	-1.44510
H	3.09031	4.88409	-0.66713
H	3.15823	4.58710	-2.42672
H	4.61007	4.33266	-1.42023
C	4.90880	1.95193	0.37026
H	5.61776	2.02134	-0.46128
C	5.36647	1.57738	1.63961
H	6.42756	1.35456	1.79594
C	4.46714	1.48668	2.70893
H	4.83156	1.19016	3.69807
C	2.13871	1.68397	3.72354
H	1.11139	1.71081	3.32093
C	2.29151	0.35563	4.49319
H	2.16634	-0.50487	3.81757
H	1.52628	0.28723	5.28545
H	3.27846	0.27352	4.98228
C	2.31877	2.88762	4.67842
H	3.34255	2.91326	5.09254
H	1.61305	2.81986	5.52497
H	2.13973	3.84741	4.16558

(iii) Fully optimized structures relevant to the $[ZnMe]^+$ abstraction studies

$[ZnMe]^+$
5
SCF(BS1) = -266.838109676
H(0 K) = -266.799265
G(298 K) = -266.827225
SCF(BS1)+D3(BJ) = -266.841896160
SCF(PCM=THF) = -266.978833479
SCF(PCM=C6H5F) = -266.969309178
SCF(BS2) = -1819.26944505
Lowest Freq. = 460.1281cm-1, 770.1870cm-1, 770.1874cm-1

Zn	0.00000	0.00000	0.47737
C	0.00000	0.00000	-1.50555
H	0.00000	1.06863	-1.76258
H	0.92546	-0.53432	-1.76258
H	-0.92546	-0.53432	-1.76258

PMe_3
13
SCF(BS1) = -126.314147354
H(0 K) = -126.196627
G(298 K) = -126.232979
SCF(BS1)+D3(BJ) = -126.330027951
SCF(PCM=THF) = -126.316456203
SCF(PCM=C6H5F) = -126.316244972
SCF(BS2) = -461.185656969
Lowest Freq. = 174.9715cm-1, 194.9019cm-1, 194.9021cm-1

P	0.00000	0.00000	0.62009
C	-0.61375	1.53506	-0.28845
H	-0.57041	1.42170	-1.38586
H	-1.65482	1.74192	0.00941
H	0.00000	2.40197	0.00668
C	1.63628	-0.23600	-0.28845
H	2.08017	-1.20098	0.00668
H	1.51643	-0.21686	-1.38586
H	2.33596	0.56215	0.00941

```
C  -1.02252 -1.29906 -0.28845
H  -0.94602 -1.20484 -1.38586
H  -0.68114 -2.30407  0.00941
H  -2.08017 -1.20098  0.00668
```

PMe_3H^+

14
SCF(BS1) = -126.683882549
H(0 K) = -126.555436
G(298 K) = -126.592269
SCF(BS1)+D3(BJ) = -126.700998391
SCF(PCM=THF) = -126.756729789
SCF(PCM=C6H5F) = -126.752570719
SCF(BS2) = -461.558574175
Lowest Freq. = 156.1508cm-1, 178.6846cm-1, 178.6846cm-1

```
P   0.00000  0.00000  0.33364
C   0.64788  1.62162 -0.21370
H   0.66182  1.65636 -1.31461
H   0.00000  2.42437  0.17134
H   1.67050  1.75703  0.17143
C   1.08042 -1.37189 -0.21370
H   0.68639 -2.32521  0.17143
H   1.10353 -1.40133 -1.31461
H   2.09956 -1.21218  0.17134
C  -1.72830 -0.24973 -0.21370
H  -1.76536 -0.25502 -1.31461
H  -2.09956 -1.21218  0.17134
H  -2.35688  0.56818  0.17143
H   0.00000  0.00000  1.75766
```

$[(PMe_3)ZnMe]^+$

18
SCF(BS1) = -393.264045239
H(0 K) = -393.104663
G(298 K) = -393.157525
SCF(BS1)+D3(BJ) = -393.290597326
SCF(PCM=THF) = -393.349185236
SCF(PCM=C6H5F) = -393.343413975
SCF(BS2) = -2280.56842002
Lowest Freq. = 3.9265cm-1, 70.8629cm-1, 71.0961cm-1

```
Zn -1.25435  0.00007  0.00006
C  -3.17990 -0.00009 -0.00004
H  -3.54386  0.63216  0.82511
H  -3.54375  0.39835 -0.96021
H  -3.54368 -1.03088  0.13494
P   1.12353  0.00005  0.00002
C   1.82272 -0.79414 -1.52033
H   2.92351 -0.76998 -1.47462
H   1.48415 -1.83981 -1.58452
H   1.48381 -0.24975 -2.41530
C   1.82299  1.71368  0.07246
H   1.48392  2.21663  0.99130
H   2.92379  1.66199  0.07078
H   1.48467  2.29210 -0.80113
C   1.82295 -0.91974  1.44772
H   2.92374 -0.89284  1.40340
H   1.48508 -0.45227  2.38554
H   1.48348 -1.96682  1.42395
```

$[(PMe_3)_2ZnMe]^+$

31
SCF(BS1) = -519.618175554
H(0 K) = -519.338899
G(298 K) = -519.413263
SCF(BS1)+D3(BJ) = -519.673240898
SCF(PCM=THF) = -519.682325187
SCF(PCM=C6H5F) = -519.678341885
SCF(BS2) = -2741.79551553
Lowest Freq. = 9.3131cm-1, 15.3736cm-1, 24.3325cm-1

```
Zn  0.00038 -0.90411 -0.01277
C  -0.00128 -2.86839 -0.00119
H   0.38944 -3.24981 -0.96002
H   0.64780 -3.24996  0.80475
H  -1.01313 -3.27777  0.14637
P   2.06711  0.41962 -0.00954
C   3.39070 -0.47817 -0.95632
H   4.35308  0.05210 -0.87391
H   3.49814 -1.49727 -0.55394
H   3.10381 -0.54828 -2.01715
C   2.11294  2.15050 -0.69162
H   1.43021  2.79499 -0.11686
H   3.13408  2.56023 -0.62871
H   1.79368  2.14196 -1.74548
C   2.76901  0.58468  1.70596
H   3.76003  1.06514  1.66964
H   2.09504  1.19255  2.32945
H   2.86734 -0.41375  2.15979
P  -2.06113  0.42599 -0.00099
C  -3.08359  0.02499 -1.50206
H  -4.07298  0.50514 -1.43280
H  -2.57059  0.38131 -2.40897
H  -3.21251 -1.06602 -1.57495
```

```
C  -2.06682  2.28582  0.07197
H  -3.10121  2.66616  0.06909
H  -1.56351  2.62322  0.99143
H  -1.53181  2.69328 -0.79974
C  -3.13330 -0.08955  1.42836
H  -2.65071  0.19282  2.37704
H  -4.12011  0.39657  1.36447
H  -3.26324 -1.18267  1.41084
```

PMe$_3$-ZnHMe

19
SCF(BS1) = -394.054968199
H(0 K) = -393.890150
G(298 K) = -393.944581
SCF(BS1)+D3(BJ) = -394.082656248
SCF(PCM=THF) = -394.064910677
SCF(PCM=C6H5F) = -394.064016365
SCF(BS2) = -2281.36251749
Lowest Freq. = 23.5785cm-1, 44.0897cm-1, 57.5596cm-1

```
Zn -1.52234 -0.55490  0.00000
H  -1.07311 -2.05086  0.00000
C  -2.66576  1.03741  0.00000
H  -3.73726  0.77403  0.00000
H  -2.47162  1.66370  0.88903
H  -2.47162  1.66370 -0.88903
P   1.12093  0.19994  0.00000
C   1.97052  1.87468  0.00000
H   3.06994  1.78215  0.00000
H   1.65517  2.44003 -0.89157
H   1.65517  2.44003  0.89157
C   1.97052 -0.63871 -1.44404
H   3.06920 -0.61106 -1.34928
H   1.63251 -1.68603 -1.49053
H   1.67572 -0.13947 -2.38116
C   1.97052 -0.63871  1.44404
H   1.63251 -1.68603  1.49053
H   3.06920 -0.61106  1.34928
H   1.67572 -0.13947  2.38116
```

IMe$_4$

21
SCF(BS1) = -383.432445632
H(0 K) = -383.243956
G(298 K) = -383.289580
SCF(BS1)+D3(BJ) = -383.462870674
SCF(PCM=THF) = -383.439334654
SCF(PCM=C6H5F) = -383.438706955
SCF(BS2) = -383.569570744
Lowest Freq. = 121.9694cm-1, 125.6595cm-1, 141.2335cm-1

```
C   0.00000  0.00000  1.59173
C   0.00000  0.68850 -0.64440
C   0.00000  1.66776 -1.77570
H   0.88896  2.32591 -1.75823
H   0.00000  1.13997 -2.74313
H  -0.88896  2.32591 -1.75823
C   0.00000 -0.68850 -0.64440
C   0.00000 -1.66776 -1.77570
H   0.00000 -1.13997 -2.74313
H   0.88896 -2.32591 -1.75823
H  -0.88896 -2.32591 -1.75823
N   0.00000  1.06523  0.71077
C   0.00000  2.44349  1.18031
H   0.89635  2.98862  0.83464
H  -0.89635  2.98862  0.83464
H   0.00000  2.40354  2.27843
N   0.00000 -1.06523  0.71077
C   0.00000 -2.44349  1.18031
H  -0.89635 -2.98862  0.83464
H   0.89635 -2.98862  0.83464
H   0.00000 -2.40354  2.27843
```

IMe$_4$-H$^+$

22
SCF(BS1) = -383.880247095
H(0 K) = -383.677816
G(298 K) = -383.724347
SCF(BS1)+D3(BJ) = -383.911763884
SCF(PCM=THF) = -383.942436103
SCF(PCM=C6H5F) = -383.938940755
SCF(BS2) = -384.007096889
Lowest Freq. = 98.6957cm-1, 103.8804cm-1, 119.7554cm-1

```
C   0.00000  0.00000  1.46016
C   0.00000  0.69343 -0.66978
C   0.00000  1.67444 -1.79625
H   0.89259  2.32352 -1.77368
H   0.00000  1.14490 -2.75956
H  -0.89259  2.32352 -1.77368
C   0.00000 -0.69343 -0.66978
C   0.00000 -1.67444 -1.79625
H   0.00000 -1.14490 -2.75956
H   0.89259 -2.32352 -1.77368
H  -0.89259 -2.32352 -1.77368
```

N   0.00000 1.09284 0.67215
C   0.00000 2.48211 1.15228
H   0.89936 3.00228 0.78991
H -0.89936 3.00228 0.78991
H   0.00000 2.48130 2.25047
N   0.00000 -1.09284 0.67215
C   0.00000 -2.48211 1.15228
H -0.89936 -3.00228 0.78991
H   0.89936 -3.00228 0.78991
H   0.00000 -2.48130 2.25047
H   0.00000 0.00000 2.54715

[(IMe$_4$)ZnMe]$^+$
26
SCF(BS1) = -650.440407816
H(0 K) = -650.209225
G(298 K) = -650.269052
SCF(BS1)+D3(BJ) = -650.482210833
SCF(PCM=THF) = -650.513807359
SCF(PCM=C6H5F) = -650.508950065
SCF(BS2) = -2203.00004827
Lowest Freq. = 23.8226cm-1, 55.6318cm-1, 65.9832cm-1

Zn 0.00071 2.06439 0.00000
C   0.00003 3.98029 0.00000
H   0.51957 4.36051 0.89471
H   0.51957 4.36051 -0.89471
H -1.03386 4.36139 0.00000
C   0.00005 0.09150 0.00000
C -0.00038 -2.07837 -0.69287
C -0.00066 -3.20816 -1.67077
H   0.89116 -3.18820 -2.32128
H -0.00049 -4.17072 -1.13934
H -0.89289 -3.18824 -2.32074
C -0.00038 -2.07837 0.69287
C -0.00066 -3.20816 1.67077
H -0.00049 -4.17072 1.13934
H   0.89116 -3.18820 2.32128
H -0.89289 -3.18824 2.32074
N -0.00019 -0.73823 -1.08441
C -0.00038 -0.29421 -2.48306
H   0.89910 -0.66274 -2.99982
H -0.89849 -0.66542 -3.00027
H -0.00217 0.80486 -2.51476
N -0.00019 -0.73823 1.08441
C -0.00038 -0.29421 2.48306
H -0.89849 -0.66542 3.00027
H   0.89910 -0.66274 2.99982
H -0.00217 0.80486 2.51476

[(IMe$_4$)$_2$ZnMe]$^+$
47
SCF(BS1) = -1033.94354623
H(0 K) = -1033.520940
G(298 K) = -1033.607866
SCF(BS1)+D3(BJ) = -1034.03063846
SCF(PCM=THF) = -1033.99649194
SCF(PCM=C6H5F) = -1033.99331507
SCF(BS2) = -2586.63256474
Lowest Freq. = 16.2966cm-1, 24.9086cm-1, 28.2649cm-1

Zn -0.01253 -1.30984 0.00428
C -0.02670 -3.28933 -0.05502
H -0.07754 -3.63860 -1.10363
H   0.88608 -3.73873 0.37201
H -0.89103 -3.72192 0.47741
C   1.72869 -0.19627 -0.04255
C   3.93431 0.36016 0.25714
C   5.29660 0.28593 0.86834
H   5.76475 -0.70234 0.71347
H   5.95899 1.03869 0.41607
H   5.27154 0.47625 1.95578
C   3.43702 1.16491 -0.74878
C   4.09803 2.22119 -1.57494
H   5.15585 2.32008 -1.28993
H   4.06735 1.98062 -2.65234
H   3.62593 3.21068 -1.44006
N   2.87291 -0.45863 0.66101
C   2.97492 -1.46494 1.72082
H   3.84073 -2.11961 1.53751
H   3.08867 -0.98431 2.70619
H   2.06161 -2.07588 1.71168
N   2.09183 0.80612 -0.90256
C   1.20002 1.39003 -1.90527
H   1.23582 2.48946 -1.85346
H   1.48998 1.06999 -2.91951
H   0.17446 1.05282 -1.69741
C -1.73881 -0.17343 0.06322
C -3.95497 0.37407 -0.16820
C -5.35875 0.23222 -0.66241
H -5.82434 -0.70797 -0.31736
H -5.97899 1.06210 -0.29290
H -5.41039 0.24445 -1.76534
C -3.37862 1.31933 0.65760
C -3.96838 2.50836 1.34578
H -5.04227 2.58443 1.12010

```
H  -3.86534  2.44223  2.44337
H  -3.49521  3.45261  1.02238
N  -2.93435 -0.52088 -0.50693
C  -3.12650 -1.68299 -1.37899
H  -3.98466 -2.27736 -1.02958
H  -3.30868 -1.36354 -2.41803
H  -2.22193 -2.30496 -1.33626
N  -2.03031  0.96129  0.77426
C  -1.06641  1.68359  1.60517
H  -1.07383  2.75595  1.35394
H  -1.30901  1.56550  2.67399
H  -0.06574  1.27239  1.41141
```

IMe$_4$-ZnHMe

27

SCF(BS1) = -651.199804733

H(0 K) = -650.963539

G(298 K) = -651.023428

SCF(BS1)+D3(BJ) = -651.243947839

SCF(PCM=THF) = -651.213585327

SCF(PCM=C6H5F) = -651.212396997

SCF(BS2) = -2203.76573511

Lowest Freq. = 16.6361cm-1, 54.1303cm-1, 120.0623cm-1

```
Zn  1.66799  1.54167  0.00000
H   1.05341  3.01211  0.00000
C   3.48407  0.70839  0.00000
H   4.26682  1.48705  0.00000
H   3.66901  0.07442  0.88917
H   3.66901  0.07442 -0.88917
C   0.00000  0.15977  0.00000
C  -1.29715 -1.74366  0.00000
C  -1.57632 -3.21278  0.00000
H  -1.15505 -3.71461  0.88993
H  -2.66184 -3.39603  0.00000
H  -1.15505 -3.71461 -0.88993
C  -2.15139 -0.66083  0.00000
C  -3.64514 -0.59205  0.00000
H  -4.07559 -1.60535  0.00000
H  -4.03458 -0.06500  0.88970
H  -4.03458 -0.06500 -0.88970
N  -0.00193 -1.21168  0.00000
C   1.21364 -2.02429  0.00000
H   1.24977 -2.66443  0.89740
H   1.24977 -2.66443 -0.89740
H   2.07515 -1.34336  0.00000
N  -1.33479  0.47636  0.00000
C  -1.84519  1.84864  0.00000
H  -2.45938  2.02878 -0.89812
H  -2.45938  2.02878  0.89812
H  -0.97528  2.52538  0.00000
```

IMes

47

SCF(BS1) = -924.161333862

H(0 K) = -923.750794

G(298 K) = -923.831436

SCF(BS1)+D3(BJ) = -924.257140855

SCF(PCM=THF) = -924.168954895

SCF(PCM=C6H5F) = -924.168163733

SCF(BS2) = -924.476044744

Lowest Freq. = 19.5135cm-1, 25.9098cm-1, 32.2848cm-1

```
C   0.00000  0.00000  0.28194
C  -0.00153  0.68295 -1.95024
H  -0.01290  1.39817 -2.76954
C   0.00153 -0.68295 -1.95024
H   0.01290 -1.39817 -2.76954
N   0.00000  1.06908 -0.59966
N   0.00000 -1.06908 -0.59966
C  -0.00268  2.44315 -0.16206
C  -1.16836  2.97135  0.43997
C   1.16063  3.22541 -0.33851
C  -1.14883  4.31700  0.84673
C   1.12562  4.56893  0.07977
C  -0.01845  5.13630  0.66754
H  -2.04585  4.73509  1.32000
H   2.02604  5.18282 -0.04617
C   0.00268 -2.44315 -0.16206
C   1.16836 -2.97135  0.43997
C  -1.16063 -3.22541 -0.33851
C   1.14883 -4.31700  0.84673
C  -1.12562 -4.56893  0.07977
C   0.01845 -5.13630  0.66754
H   2.04585 -4.73509  1.32000
H  -2.02604 -5.18282 -0.04617
C  -2.39285  2.11122  0.65528
H  -2.81513  1.76517 -0.30567
H  -2.12960  1.20738  1.23047
H  -3.17627  2.66991  1.19157
C  -0.04077  6.59189  1.08475
H  -0.49688  7.22630  0.30207
H  -0.63034  6.74003  2.00514
H   0.97657  6.97643  1.26537
C   2.42507  2.63450 -0.92423
H   2.66505  1.66752 -0.45068
```

```
H   2.33499  2.44289 -2.00845
H   3.27740  3.31664 -0.77635
C  -2.42507 -2.63450 -0.92423
H  -2.66505 -1.66752 -0.45068
H  -2.33499 -2.44289 -2.00845
H  -3.27740 -3.31664 -0.77635
C   2.39285 -2.11122  0.65528
H   2.81513 -1.76517 -0.30567
H   2.12960 -1.20738  1.23047
H   3.17627 -2.66991  1.19157
C   0.04077 -6.59189  1.08475
H  -0.97657 -6.97643  1.26537
H   0.49688 -7.22630  0.30207
H   0.63034 -6.74003  2.00514
```

IMes-H$^+$

48
SCF(BS1) = -924.613506713
H(0 K) = -924.189183
G(298 K) = -924.274119
SCF(BS1)+D3(BJ) = -924.710292733
SCF(PCM=THF) = -924.665414844
SCF(PCM=C6H5F) = -924.662380270
SCF(BS2) = -924.919047785
Lowest Freq. = 6.1849cm-1, 19.7682cm-1, 30.6751cm-1

```
C   0.00000  0.00000  0.14268
C   0.50302  0.46799 -1.97367
H   1.02950  0.95909 -2.78769
C  -0.50302 -0.46799 -1.97367
H  -1.02950 -0.95909 -2.78769
N   0.80456  0.74837 -0.64297
N  -0.80456 -0.74837 -0.64297
C   1.81347  1.68812 -0.17009
C   1.42379  3.02447  0.07145
C   3.13333  1.21774  0.01226
C   2.42212  3.90720  0.51935
C   4.08440  2.14992  0.46224
C   3.75410  3.49507  0.71854
H   2.14774  4.94940  0.71717
H   5.11526  1.81192  0.61609
C  -1.81347 -1.68812 -0.17009
C  -1.42379 -3.02447  0.07145
C  -3.13333 -1.21774  0.01226
C  -2.42212 -3.90720  0.51935
C  -4.08440 -2.14992  0.46224
C  -3.75410 -3.49507  0.71854
H  -2.14774 -4.94940  0.71717
H  -5.11526 -1.81192  0.61609
C   0.00000  3.49727 -0.12803
H  -0.37894  3.26340 -1.13806
H  -0.69348  3.03304  0.59641
H  -0.06712  4.58671  0.00852
C   4.80949  4.47847  1.16989
H   5.29552  4.95453  0.29888
H   4.37664  5.28434  1.78355
H   5.60068  3.98344  1.75510
C   3.51897 -0.22142 -0.25307
H   2.98117 -0.92230  0.41037
H   3.30088 -0.52525 -1.29191
H   4.59564 -0.36964 -0.08350
C  -3.51897  0.22142 -0.25307
H  -2.98117  0.92230  0.41037
H  -3.30088  0.52525 -1.29191
H  -4.59564  0.36964 -0.08350
C   0.00000 -3.49727 -0.12803
H   0.37894 -3.26340 -1.13806
H   0.69348 -3.03304  0.59641
H   0.06712 -4.58671  0.00852
C  -4.80949 -4.47847  1.16989
H  -5.60068 -3.98344  1.75510
H  -5.29552 -4.95453  0.29888
H  -4.37664 -5.28434  1.78355
H   0.00000  0.00000  1.22947
```

[(IMes)ZnMe]$^+$

52
SCF(BS1) = -1191.17750556
H(0 K) = -1190.724328
G(298 K) = -1190.821685
SCF(BS1)+D3(BJ) = -1191.29030865
SCF(PCM=THF) = -1191.24173733
SCF(PCM=C6H5F) = -1191.23733385
SCF(BS2) = -2743.91457931
Lowest Freq. = 12.5761cm-1, 17.6002cm-1, 25.0356cm-1

```
Zn  0.00000  1.62515 -0.19110
C   0.00001  3.52853 -0.41379
H  -0.89626  3.84682 -0.97043
H   0.00080  4.02792  0.56872
H   0.89548  3.84666 -0.97179
C   0.00000 -0.32777  0.03773
C  -0.68721 -2.46753  0.28911
H  -1.40451 -3.27874  0.38453
C   0.68720 -2.46753  0.28911
H   1.40450 -3.27874  0.38453
```

N -1.08948 -1.14547 0.13385
N   1.08947 -1.14547 0.13385
C -2.46362 -0.67154 0.07860
C -3.09979 -0.30165 1.28675
C -3.10409 -0.60211 -1.18074
C -4.42370 0.16400 1.19879
C -4.42800 -0.12859 -1.20249
C -5.10633 0.25650 -0.02976
H -4.93658 0.46011 2.12082
H -4.94424 -0.06223 -2.16684
C   2.46361 -0.67155 0.07861
C   3.10409 -0.60211 -1.18074
C   3.09979 -0.30165 1.28675
C   4.42800 -0.12859 -1.20249
C   4.42369 0.16400 1.19879
C   5.10633 0.25650 -0.02976
H   4.94424 -0.06224 -2.16684
H   4.93657 0.46011 2.12082
C -2.40248 -0.40870 2.62611
H -2.13049 -1.45211 2.86424
H -1.46900 0.18094 2.65967
H -3.05528 -0.04276 3.43242
C -6.54096 0.72990 -0.08464
H -7.23637 -0.12176 0.02755
H -6.76415 1.44218 0.72559
H -6.77145 1.21506 -1.04642
C -2.41098 -1.02675 -2.45779
H -1.47506 -0.46634 -2.63133
H -2.14427 -2.09823 -2.44261
H -3.06443 -0.85909 -3.32675
C   2.40248 -0.40870 2.62611
H   1.46900 0.18095 2.65967
H   2.13049 -1.45211 2.86425
H   3.05527 -0.04275 3.43242
C   2.41098 -1.02676 -2.45778
H   2.14428 -2.09825 -2.44260
H   1.47506 -0.46636 -2.63133
H   3.06442 -0.85910 -3.32675
C   6.54096 0.72990 -0.08464
H   6.76415 1.44217 0.72559
H   7.23637 -0.12176 0.02754
H   6.77144 1.21507 -1.04642

[(IMes)$_2$ZnMe]$^+$
99
SCF(BS1) = -2115.39279361
H(0 K) = -2114.526346
G(298 K) = -2114.679371
SCF(BS1)+D3(BJ) = -2115.64384448
SCF(PCM=THF) = -2115.43905113
SCF(PCM=C6H5F) = -2115.43617324
SCF(BS2) = -3668.43716747
Lowest Freq. = 3.4191cm-1, 13.2611cm-1, 18.7650cm-1

Zn 0.03541 0.06904 -0.88523
C   0.07829 0.17492 -2.87217
H   0.47502 1.13096 -3.24659
H   0.70238 -0.62662 -3.30593
H -0.93985 0.04696 -3.28112
C   0.36330 1.79075 0.23305
C   0.13062 3.77710 1.35194
H -0.37823 4.51215 1.96973
C   1.34902 3.79853 0.73501
H   2.13383 4.55000 0.71443
N -0.45619 2.54835 1.03690
N   1.47143 2.58744 0.05550
C -1.79227 2.22524 1.49718
C -2.88470 2.44753 0.62750
C -1.96388 1.83500 2.84611
C -4.17829 2.26896 1.15252
C -3.27964 1.66959 3.31478
C -4.39958 1.89684 2.49170
H -5.03777 2.44721 0.49611
H -3.43140 1.36882 4.35807
C   2.63263 2.25817 -0.74882
C   3.68238 1.52652 -0.15221
C   2.70708 2.75805 -2.07004
C   4.81114 1.24786 -0.94506
C   3.85677 2.44532 -2.81559
C   4.91418 1.68555 -2.27826
H   5.63975 0.68618 -0.49865
H   3.93167 2.81770 -3.84390
C -2.68598 2.88991 -0.80584
H -2.03100 3.77612 -0.87014
H -2.22007 2.10004 -1.42173
H -3.65062 3.14726 -1.26891
C -5.80282 1.77343 3.04443
H -6.10380 2.70359 3.55990
H -6.54066 1.59009 2.24700
H -5.87943 0.95746 3.78183
C -0.78274 1.62870 3.76984
H   0.01182 1.03036 3.29358
H -0.32424 2.58917 4.06664
H -1.09694 1.11702 4.69254
C   1.62270 3.63743 -2.65303
H   0.61524 3.23699 -2.45437
H   1.65927 4.65635 -2.22626

```
H   1.74419  3.73338 -3.74288
C   3.61869  1.09262  1.29475
H   3.36043  1.93585  1.95882
H   2.86242  0.30571  1.45879
H   4.58898  0.68977  1.61987
C   6.12737  1.35135 -3.11818
H   5.91175  0.51644 -3.80958
H   6.43817  2.20919 -3.73727
H   6.98364  1.05151 -2.49337
C  -0.35948 -1.80391 -0.05877
C  -0.16464 -3.95768  0.70168
H   0.32780 -4.79583  1.18738
C  -1.37664 -3.85398  0.08082
H  -2.17064 -4.57911 -0.07695
N   0.44160 -2.70165  0.60849
N  -1.47617 -2.54145 -0.37995
C   1.77484 -2.48058  1.13361
C   2.87723 -2.56369  0.25162
C   1.93220 -2.32525  2.53075
C   4.16516 -2.48895  0.81343
C   3.24333 -2.25263  3.03529
C   4.37194 -2.34880  2.19877
H   5.03159 -2.56113  0.14594
H   3.38402 -2.13251  4.11591
C  -2.63150 -2.06092 -1.11399
C  -3.69737 -1.48336 -0.39070
C  -2.68683 -2.27534 -2.51135
C  -4.82305 -1.05895 -1.12134
C  -3.83431 -1.82808 -3.18889
C  -4.90780 -1.21031 -2.51739
H  -5.66383 -0.61357 -0.57722
H  -3.89564 -1.98145 -4.27259
C   2.68997 -2.74584 -1.23871
H   2.06545 -3.62743 -1.46562
H   2.19007 -1.87150 -1.69165
H   3.66052 -2.87788 -1.74012
C   5.76904 -2.33229  2.77989
H   6.06648 -3.34338  3.11271
H   6.51495 -2.00154  2.03942
H   5.83626 -1.67060  3.65900
C   0.74157 -2.26842  3.46347
H  -0.04995 -1.60296  3.08055
H   0.28272 -3.26440  3.59930
H   1.04557 -1.90987  4.45897
C  -1.58051 -2.99525 -3.24944
H  -0.59544 -2.54661 -3.04331
H  -1.52619 -4.05903 -2.95574
H  -1.75081 -2.95872 -4.33613
C  -3.65222 -1.35881  1.11529
H  -3.39659 -2.32025  1.59384
H  -2.90139 -0.62070  1.44599
H  -4.62828 -1.03529  1.50548
C  -6.11976 -0.72683 -3.28321
H  -5.90530  0.22335 -3.80539
H  -6.42649 -1.45426 -4.05308
H  -6.97863 -0.55217 -2.61587
```

IMes-ZnHMe

53
SCF(BS1) = -1191.92498677
H(0 K) = -1191.467044
G(298 K) = -1191.563087
SCF(BS1)+D3(BJ) = -1192.04098122
SCF(PCM=THF) = -1191.93987182
SCF(PCM=C6H5F) = -1191.93847597
SCF(BS2) = -2744.67005022
Lowest Freq. = 19.9547cm-1, 23.7594cm-1, 28.4101cm-1

```
Zn -0.00118  0.44085  1.83645
H  -0.00176  2.03494  1.90550
C  -0.00131 -1.16425  3.01118
H  -0.00153 -0.90361  4.08346
H  -0.88854 -1.79884  2.82521
H   0.88619 -1.79852  2.82545
C   0.00018 -0.00426 -0.26688
C  -0.68328 -0.26750 -2.44527
H  -1.40288 -0.35838 -3.25490
C   0.68434 -0.26755 -2.44503
H   1.40423 -0.35845 -3.25440
N  -1.07797 -0.10704 -1.11167
N   1.07859 -0.10710 -1.11127
C  -2.45681 -0.04524 -0.67639
C  -3.14996 -1.25385 -0.44105
C  -3.06091  1.22006 -0.50016
C  -4.49244 -1.16850 -0.03171
C  -4.40552  1.24610 -0.08632
C  -5.13886  0.06866  0.14774
H  -5.04342 -2.09675  0.16151
H  -4.88902  2.21913  0.06144
C   2.45730 -0.04532 -0.67558
C   3.06175  1.21991 -0.50013
C   3.15008 -1.25395 -0.43934
C   4.40642  1.24588 -0.08643
C   4.49263 -1.16867 -0.03019
C   5.13951  0.06842  0.14820
H   4.89018  2.21890  0.06055
H   5.04332 -2.09694  0.16377
```

C	-2.46500	-2.59465	-0.58805
H	-1.62807	-2.68863	0.12596
H	-3.17213	-3.41660	-0.39600
H	-2.04273	-2.73635	-1.59822
C	-6.59383	0.13038	0.56166
H	-7.25930	0.14364	-0.32146
H	-6.87782	-0.74328	1.17108
H	-6.80900	1.04017	1.14579
C	-2.28640	2.50044	-0.71402
H	-1.51152	2.61914	0.06677
H	-1.77134	2.50971	-1.69027
H	-2.95737	3.37270	-0.67040
C	2.46469	-2.59459	-0.58560
H	1.62454	-2.68596	0.12495
H	2.04683	-2.73869	-1.59730
H	3.17037	-3.41655	-0.38830
C	2.28766	2.50040	-0.71502
H	1.77431	2.50991	-1.69217
H	1.51147	2.61914	0.06440
H	2.95863	3.37258	-0.67013
C	6.59460	0.13005	0.56167
H	6.87755	-0.74122	1.17498
H	7.25992	0.13839	-0.32163
H	6.81103	1.04219	1.14163

[11b]$^{+}$

141

SCF(BS1) = -2694.67458120

H(0 K) = -2693.413848

G(298 K) = -2693.596767

SCF(BS1)+D3(BJ) = -2695.06809965

SCF(PCM=THF) = -2694.72002589

SCF(PCM=C6H5F) = -2694.71719643

SCF(BS2) = -4247.86811792

Lowest Freq. = 9.3321cm-1, 21.7667cm-1, 23.7186cm-1

Ir	-0.00530	0.06305	0.02110
H	0.20918	-0.29356	-1.54621
H	0.92736	-1.21868	0.77567
H	-0.16753	0.33123	1.76222
H	-0.58796	1.00853	1.37385
H	-0.82250	1.27427	-0.67821
Zn	1.08420	-2.11309	-0.74286
N	3.02286	0.92783	-0.31501
N	1.77853	2.63485	0.17365
N	-2.28586	-1.83207	1.07433
N	-2.77013	-1.16572	-0.93871
C	1.70393	1.26855	-0.04797
C	3.86604	2.04156	-0.26095
H	4.93320	1.95697	-0.44216
C	3.08359	3.11235	0.04523
H	3.32204	4.16234	0.18476
C	3.58967	-0.38368	-0.55967
C	4.03362	-1.14394	0.55834
C	4.68827	-2.36216	0.28880
H	5.04566	-2.97310	1.12365
C	4.92755	-2.78389	-1.02645
H	5.45411	-3.72611	-1.20939
C	4.51457	-1.99509	-2.10649
H	4.72931	-2.32417	-3.12766
C	3.83807	-0.77428	-1.90357
C	3.92926	-0.62532	1.99399
H	3.21542	0.21690	1.99944
C	5.30538	-0.08373	2.45600
H	6.05990	-0.88901	2.47434
H	5.23128	0.33378	3.47454
H	5.67915	0.71096	1.78920
C	3.40089	-1.67609	2.99084
H	2.39303	-2.02462	2.71360
H	3.33824	-1.23509	4.00007
H	4.06615	-2.55382	3.06161
C	3.48204	0.11802	-3.09395
H	2.76539	0.88096	-2.74176
C	0.70318	3.53001	0.55653
C	0.49861	3.77795	1.93889
C	-0.52644	4.68197	2.28358
H	-0.71435	4.90256	3.33918
C	-1.28373	5.32811	1.29819
H	-2.06883	6.03341	1.58942
C	-1.01799	5.10129	-0.05872
H	-1.59067	5.64286	-0.81785
C	-0.01414	4.20054	-0.46570
C	1.42053	3.20815	3.02187
H	2.01042	2.38855	2.57354
C	2.41590	4.30029	3.48817
H	3.00537	4.70510	2.64894
H	3.11974	3.89007	4.23236
H	1.88046	5.14418	3.95627
C	0.67197	2.62245	4.23665
H	0.06913	3.38575	4.75774
H	1.39748	2.22742	4.96791
H	-0.00014	1.79733	3.95087
C	0.34471	4.04798	-1.94493
H	0.94997	3.13071	-2.05147
C	1.21219	5.24717	-2.40238
H	1.50928	5.12708	-3.45830
H	2.13097	5.34594	-1.80015

H	0.65170	6.19396	-2.31376
C	-0.88324	3.89016	-2.86286
H	-1.52372	4.78923	-2.85523
H	-1.49823	3.02370	-2.57343
H	-0.55221	3.73800	-3.90423
C	-1.79494	-1.04258	0.04009
C	-3.51483	-2.40593	0.74432
H	-4.05470	-3.04004	1.44094
C	-3.81667	-1.98929	-0.51653
H	-4.67864	-2.17985	-1.14849
C	-1.74354	-1.95221	2.41685
C	-0.87046	-3.02899	2.71795
C	-0.36994	-3.10420	4.03268
H	0.30783	-3.91914	4.30282
C	-0.73857	-2.16605	5.00577
H	-0.33492	-2.24541	6.02035
C	-1.65198	-1.15181	4.69462
H	-1.97404	-0.45710	5.47681
C	-2.19072	-1.02768	3.39794
C	-0.56567	-4.13456	1.70556
H	-0.70293	-3.71225	0.69150
C	0.87211	-4.68370	1.79456
H	1.62729	-3.88448	1.71178
H	1.04861	-5.40352	0.97858
H	1.04903	-5.21974	2.74273
C	-1.57889	-5.29664	1.86542
H	-1.49660	-5.74672	2.86979
H	-1.38017	-6.08593	1.12049
H	-2.61990	-4.96056	1.73243
C	-3.30837	-0.01546	3.12865
H	-3.45223	0.05681	2.03623
C	-3.00698	1.40662	3.64306
H	-2.86248	1.43005	4.73676
H	-3.85531	2.07338	3.41365
H	-2.10874	1.83661	3.17143
C	-4.63385	-0.53385	3.74175
H	-4.90197	-1.53177	3.35707
H	-5.46266	0.15592	3.50825
H	-4.55503	-0.61050	4.83991
C	-2.90078	-0.42508	-2.18508
C	-3.63266	0.78995	-2.15133
C	-3.82110	1.46573	-3.37359
H	-4.38431	2.40429	-3.38333
C	-3.31977	0.94837	-4.57280
H	-3.47845	1.48931	-5.51129
C	-2.63851	-0.27518	-4.57899
H	-2.27917	-0.68289	-5.52778
C	-2.42430	-1.00102	-3.39055
C	-4.29682	1.32727	-0.88186
H	-3.90316	0.75831	-0.02155
C	-3.98649	2.81470	-0.61902
H	-2.90327	2.99297	-0.53144
H	-4.46330	3.13687	0.32257
H	-4.37885	3.46426	-1.42052
C	-5.82650	1.09423	-0.94148
H	-6.27736	1.64991	-1.78174
H	-6.30670	1.44227	-0.01090
H	-6.07596	0.02837	-1.07557
C	-1.78471	-2.39010	-3.43759
H	-1.26564	-2.55603	-2.47441
C	-0.74479	-2.54375	-4.56505
H	-1.22232	-2.56956	-5.55982
H	-0.20025	-3.49485	-4.44657
H	-0.01181	-1.72129	-4.56405
C	-2.86608	-3.49055	-3.58699
H	-3.57907	-3.49492	-2.74749
H	-2.39435	-4.48724	-3.63163
H	-3.43936	-3.34403	-4.51869
C	1.44462	-3.75994	-1.70697
H	2.30815	-4.27237	-1.25467
H	1.67839	-3.54784	-2.76184
H	0.57171	-4.43254	-1.66624
C	4.74181	0.85123	-3.61707
H	5.21350	1.47206	-2.83775
H	4.47815	1.51078	-4.46120
H	5.49655	0.12929	-3.97399
C	2.80395	-0.65985	-4.23971
H	3.49096	-1.38173	-4.71387
H	2.47218	0.03877	-5.02608
H	1.92370	-1.21649	-3.88033

13

136

SCF(BS1) = -2427.66972583

H(0 K) = -2426.451809

G(298 K) = -2426.629731

SCF(BS1)+D3(BJ) = -2428.03080802

SCF(PCM=THF) = -2427.68322217

SCF(PCM=C6H5F) = -2427.68164810

SCF(BS2) = -2428.43763147

Lowest Freq. = 12.3826cm-1, 20.9243cm-1, 21.2535cm-1

Ir	0.00299	-0.00006	0.00047
H	0.52633	0.03755	-1.56325
H	-1.34319	-0.00128	-0.94475
H	-1.33873	0.00439	0.95201
H	0.53482	-0.03902	1.56144

H	1.64490	-0.00181	-0.00360
N	-1.02370	-2.91823	-0.43042
N	1.01245	-2.94047	0.31580
N	-1.01733	2.92067	0.42923
N	1.01927	2.93800	-0.31587
C	-0.00300	-2.05349	-0.03804
C	-0.64680	-4.26126	-0.32565
H	-1.32419	-5.06563	-0.59679
C	0.63083	-4.27529	0.14402
H	1.30635	-5.09451	0.37191
C	-2.35106	-2.58087	-0.89880
C	-3.40801	-2.52603	0.04325
C	-4.71179	-2.32966	-0.45176
H	-5.54955	-2.28557	0.25179
C	-4.95429	-2.19843	-1.82463
H	-5.97725	-2.05336	-2.18865
C	-3.88935	-2.25243	-2.73256
H	-4.08654	-2.14924	-3.80495
C	-2.56495	-2.44354	-2.29235
C	-3.17277	-2.72332	1.54056
H	-2.09104	-2.60542	1.72395
C	-3.58186	-4.15127	1.97439
H	-4.65793	-4.32482	1.79479
H	-3.39051	-4.29906	3.05183
H	-3.02005	-4.92290	1.42131
C	-3.89512	-1.66360	2.39680
H	-3.60752	-0.64453	2.09424
H	-3.63078	-1.79686	3.46043
H	-4.99379	-1.75023	2.32145
C	-1.42528	-2.53266	-3.30641
H	-0.47625	-2.50651	-2.74457
C	2.34287	-2.63114	0.79447
C	2.56589	-2.57082	2.19213
C	3.89374	-2.40609	2.63288
H	4.09911	-2.36247	3.70763
C	4.95269	-2.30362	1.72201
H	5.97817	-2.17994	2.08683
C	4.70112	-2.35931	0.34574
H	5.53403	-2.27807	-0.36040
C	3.39370	-2.52664	-0.15014
C	1.43186	-2.72517	3.20506
H	0.48059	-2.61127	2.65775
C	1.45400	-4.13441	3.84294
H	1.36223	-4.92679	3.08085
H	0.61952	-4.25091	4.55663
H	2.39643	-4.30495	4.39332
C	1.46215	-1.62970	4.29101
H	2.36410	-1.69962	4.92468
H	0.58637	-1.73181	4.95507
H	1.43332	-0.62747	3.83439
C	3.14826	-2.63950	-1.65434
H	2.06244	-2.53785	-1.82160
C	3.58781	-4.02777	-2.17724
H	3.38677	-4.11590	-3.25942
H	3.05232	-4.84507	-1.66490
H	4.66997	-4.18487	-2.02059
C	3.83435	-1.50936	-2.44788
H	4.93536	-1.56451	-2.37756
H	3.51346	-0.52065	-2.08501
H	3.57194	-1.58847	-3.51745
C	0.00174	2.05344	0.03810
C	-0.63761	4.26281	0.32343
H	-1.31334	5.06882	0.59381
C	0.64027	4.27377	-0.14563
H	1.31745	5.09139	-0.37435
C	-2.34520	2.58626	0.89821
C	-3.40255	2.53266	-0.04347
C	-4.70648	2.33882	0.45214
H	-5.54456	2.29562	-0.25107
C	-4.94869	2.20883	1.82518
H	-5.97177	2.06568	2.18963
C	-3.88336	2.26154	2.73273
H	-4.08036	2.15930	3.80524
C	-2.55880	2.45016	2.29192
C	-3.16714	2.72838	-1.54097
H	-2.08616	2.60446	-1.72493
C	-3.89579	1.67262	-2.39675
H	-3.61480	0.65190	-2.09351
H	-3.63018	1.80352	-3.46035
H	-4.99393	1.76625	-2.32200
C	-3.56884	4.15841	-1.97494
H	-4.64385	4.33769	-1.79459
H	-3.37751	4.30482	-3.05257
H	-3.00252	4.92723	-1.42258
C	-1.41856	2.53833	3.30543
H	-0.46980	2.50994	2.74322
C	-1.40601	1.33327	4.26911
H	-2.31171	1.30016	4.90047
H	-0.53584	1.39854	4.94532
H	-1.33587	0.38928	3.70590
C	-1.47567	3.87353	4.08387
H	-1.42124	4.74167	3.40506
H	-0.63431	3.94429	4.79541
H	-2.41372	3.95755	4.66107
C	2.34918	2.62581	-0.79415
C	3.39927	2.51804	0.15100
C	4.70658	2.34826	-0.34425
H	5.53888	2.26447	0.36229

C 4.95890 2.29348 -1.72046
H 5.98435 2.16790 -2.08475
C 3.90080 2.39951 -2.63182
H 4.10668 2.35687 -3.70653
C 2.57301 2.56676 -2.19170
C 3.15306 2.63040 1.65512
H 2.06712 2.52894 1.82173
C 3.83841 1.49990 2.44875
H 3.51746 0.51135 2.08552
H 3.57540 1.57888 3.51819
H 4.93948 1.55475 2.37917
C 3.59277 4.01839 2.17867
H 4.67505 4.17520 2.02255
H 3.39125 4.10621 3.26078
H 3.05773 4.83602 1.66638
C 1.44056 2.72473 -3.20582
H 0.48807 2.62248 -2.65836
C 1.46229 1.62203 -4.28470
H 2.36780 1.67724 -4.91471
H 0.59056 1.73012 -4.95317
H 1.41972 0.62328 -3.82155
C 1.47490 4.12977 -3.85227
H 1.39155 4.92754 -3.09481
H 0.64061 4.24965 -4.56560
H 2.41807 4.28850 -4.40491
C -1.48500 -3.86689 -4.08630
H -1.43187 -4.73588 -3.40847
H -0.64396 -3.93835 -4.79817
H -2.42334 -3.94864 -4.66334
C -1.41122 -1.32657 -4.26882
H -2.31746 -1.29107 -4.89929
H -0.54182 -1.39280 -4.94592
H -1.33879 -0.38327 -3.70473

[15b]$^{+}$
140
SCF(BS1) = -2694.24722524
H(0 K) = -2692.998590
G(298 K) = -2693.180983
SCF(BS1)+D3(BJ) = -2694.63869152
SCF(PCM=THF) = -2694.26319273
SCF(PCM=C6H5F) = -2694.26125818
SCF(BS2) = -4247.44791876
Lowest Freq. = 12.5358cm-1, 19.1061cm-1, 22.4832cm-1

Ir 0.01096 0.00587 0.05689
H 1.58350 -0.14553 0.47909
H -0.23136 -0.30402 1.66023
H -1.69921 0.10439 0.01051
H 0.67772 0.25079 -1.48957
Zn -1.38063 0.28853 -1.93993
N -0.99498 -2.96754 0.17068
N 0.85459 -2.82698 -0.96180
N -0.77149 3.01098 0.58900
N 1.31059 2.64726 1.08550
C 3.09804 1.77134 2.53671
C -2.41774 0.29688 -3.61198
H -3.35328 0.86693 -3.48046
H -2.68374 -0.73365 -3.90920
H -1.85307 0.74872 -4.44529
C -0.05186 -2.02823 -0.25897
C -2.15793 -2.79074 1.02082
C -3.43678 -2.68043 0.41795
C -3.62620 -2.57363 -1.09482
H -2.65766 -2.26935 -1.53134
C -4.01221 -3.94226 -1.70544
H -3.24330 -4.70856 -1.51196
H -4.13693 -3.85596 -2.79904
H -4.96589 -4.30521 -1.28242
C -4.66597 -1.50036 -1.48019
H -5.68360 -1.78086 -1.15540
H -4.69352 -1.37713 -2.57544
H -4.41867 -0.52306 -1.03441
C -4.56491 -2.72023 1.26189
H -5.56414 -2.64670 0.82110
C -4.43128 -2.86319 2.64761
H -5.32217 -2.90040 3.28380
C -3.15700 -2.95478 3.21860
H -3.05869 -3.06712 4.30315
C -1.99473 -2.92540 2.42340
C -0.62423 -3.10710 3.07451
H 0.13691 -2.78450 2.34386
C -0.37490 -4.59568 3.41626
H -1.12586 -4.96647 4.13662
H 0.62311 -4.72692 3.87011
H -0.42514 -5.23356 2.51790
C -0.44894 -2.22348 4.32579
H -0.61046 -1.16206 4.07827
H 0.57479 -2.33210 4.72242
H -1.14388 -2.50613 5.13639
C -0.67327 -4.26404 -0.24880
H -1.29831 -5.11486 0.00543
C 0.48369 -4.17527 -0.95855
H 1.08434 -4.93227 -1.45401
C 2.12444 -2.44719 -1.54842
C 3.29030 -2.56986 -0.75149
C 3.24164 -3.01753 0.70900

H	2.19080	-2.96769	1.04077
C	3.71772	-4.48375	0.84415
H	4.76710	-4.59047	0.51625
H	3.10486	-5.17052	0.23606
H	3.65719	-4.81504	1.89569
C	4.05246	-2.08966	1.63457
H	5.13203	-2.11550	1.40271
H	3.93680	-2.41023	2.68489
H	3.70614	-1.04836	1.54888
C	4.53281	-2.32678	-1.36890
H	5.44928	-2.41440	-0.77611
C	4.61488	-1.99025	-2.72467
H	5.59152	-1.81190	-3.18761
C	3.44779	-1.88735	-3.49178
H	3.52163	-1.63175	-4.55347
C	2.17826	-2.11387	-2.92477
C	0.92569	-2.05879	-3.79828
H	0.05816	-1.93521	-3.12555
C	0.73922	-3.38422	-4.57543
H	1.59517	-3.56734	-5.24920
H	-0.17651	-3.34641	-5.19117
H	0.65469	-4.24816	-3.89513
C	0.92827	-0.85974	-4.76814
H	1.08345	0.08707	-4.22571
H	-0.03891	-0.79952	-5.29448
H	1.71577	-0.95186	-5.53697
C	0.19004	1.99147	0.56798
C	-2.11019	2.98131	0.04200
C	-2.32881	3.57651	-1.22835
C	-1.21592	4.26599	-2.02410
H	-0.25128	4.01091	-1.55264
C	-1.14443	3.80603	-3.49521
H	-1.00302	2.71643	-3.57017
H	-0.29950	4.30159	-4.00423
H	-2.06178	4.06490	-4.05244
C	-1.38112	5.80399	-1.95567
H	-2.33154	6.11852	-2.42192
H	-0.55783	6.30586	-2.49335
H	-1.38254	6.17218	-0.91619
C	-3.65054	3.59137	-1.72180
H	-3.85082	4.04042	-2.70025
C	-4.70515	3.04998	-0.97972
H	-5.72350	3.06787	-1.38294
C	-4.46387	2.49849	0.28711
H	-5.29972	2.09498	0.86604
C	-3.16714	2.45954	0.83229
C	-2.91417	1.94063	2.24737
H	-1.90250	1.49793	2.25246
C	-2.95030	3.10863	3.26419
H	-2.19784	3.88100	3.03400
H	-2.74820	2.73480	4.28344
H	-3.94375	3.59221	3.27169
C	-3.89181	0.83237	2.67548
H	-4.92355	1.20976	2.79876
H	-3.58140	0.41929	3.64981
H	-3.90442	0.00409	1.95007
C	-0.26237	4.20837	1.10256
H	-0.87821	5.09820	1.19188
C	1.04261	3.98130	1.40984
H	1.80881	4.63122	1.82160
C	2.66591	2.16066	1.24480
C	3.55381	2.26119	0.14427
C	3.09474	2.74969	-1.22886
H	1.99323	2.69454	-1.25004
C	3.60732	1.85603	-2.37546
H	4.70800	1.88503	-2.46457
H	3.19428	2.20697	-3.33749
H	3.29768	0.80992	-2.22736
C	3.51095	4.22437	-1.44510
H	3.09031	4.88409	-0.66713
H	3.15823	4.58710	-2.42672
H	4.61007	4.33266	-1.42023
C	4.90880	1.95193	0.37026
H	5.61776	2.02134	-0.46128
C	5.36647	1.57738	1.63961
H	6.42756	1.35456	1.79594
C	4.46714	1.48668	2.70893
H	4.83156	1.19016	3.69807
C	2.13871	1.68397	3.72354
H	1.11139	1.71081	3.32093
C	2.29151	0.35563	4.49319
H	2.16634	-0.50487	3.81757
H	1.52628	0.28723	5.28545
H	3.27846	0.27352	4.98228
C	2.31877	2.88762	4.67842
H	3.34255	2.91326	5.09254
H	1.61305	2.81986	5.52497
H	2.13973	3.84741	4.16558

[17]$^{-}$

135

SCF(BS1) = -2427.08001337

H(0 K) = -2425.876510

G(298 K) = -2426.053190

SCF(BS1)+D3(BJ) = -2427.44089155

SCF(PCM=THF) = -2427.14863950

SCF(PCM=C6H5F) = -2427.14288961

SCF(BS2) = -2427.85955052

Lowest Freq. = 11.5218cm-1, 17.5546cm-1, 19.0315cm-1

Ir	-0.01484	0.00554	0.02066
H	1.34627	-0.12047	1.00434
H	-1.06688	-0.12136	1.33373
H	-1.36633	0.24878	-0.95399
H	1.03482	0.00838	-1.29809
N	-1.19824	-2.86469	0.13941
N	0.82034	-2.90683	-0.67059
N	-0.82517	3.00664	-0.11573
N	1.19099	2.79253	0.67592
C	2.66428	2.02794	2.49745
C	-0.13502	-1.98800	-0.17687
C	-2.49521	-2.52022	0.66314
C	-3.55151	-2.25884	-0.24529
C	-3.30634	-2.18879	-1.75162
H	-2.22664	-2.01021	-1.89026
C	-3.68370	-3.52588	-2.43199
H	-3.10149	-4.36795	-2.02009
H	-3.48743	-3.47768	-3.51852
H	-4.75694	-3.75350	-2.29190
C	-4.03535	-1.00447	-2.41399
H	-5.13568	-1.10333	-2.35893
H	-3.76558	-0.95091	-3.48385
H	-3.73674	-0.05730	-1.93851
C	-4.84925	-2.10763	0.28057
H	-5.68272	-1.90928	-0.40216
C	-5.09115	-2.20949	1.65723
H	-6.11009	-2.09509	2.04564
C	-4.02686	-2.44464	2.53688
H	-4.21682	-2.50654	3.61471
C	-2.71015	-2.59840	2.06102
C	-1.56492	-2.85350	3.03891
H	-0.62648	-2.79982	2.46242
C	-1.66999	-4.25983	3.67143
H	-2.60530	-4.37030	4.25071
H	-0.82490	-4.44140	4.35992
H	-1.65693	-5.05056	2.90121
C	-1.48733	-1.75235	4.11638
H	-1.36016	-0.77283	3.62688
H	-0.62077	-1.92752	4.77896
H	-2.39342	-1.72788	4.74989
C	-0.89755	-4.20513	-0.13364
H	-1.60943	-5.00286	0.06091
C	0.36515	-4.23126	-0.64176
H	0.98396	-5.05696	-0.98271
C	2.15806	-2.62415	-1.12505
C	3.22731	-2.72572	-0.20162
C	2.98040	-2.97981	1.28439
H	1.91412	-2.77109	1.47382
C	3.26748	-4.45571	1.64715
H	4.32392	-4.71569	1.44883
H	2.63419	-5.14619	1.06391
H	3.07046	-4.63851	2.71917
C	3.78522	-2.01923	2.18086
H	4.87665	-2.16430	2.07719
H	3.53120	-2.19359	3.24215
H	3.53921	-0.97453	1.93392
C	4.54102	-2.62127	-0.69982
H	5.38408	-2.70023	-0.00433
C	4.78439	-2.41951	-2.06364
H	5.81412	-2.34548	-2.43293
C	3.70944	-2.30057	-2.95495
H	3.90565	-2.12813	-4.01893
C	2.37811	-2.39386	-2.50634
C	1.21254	-2.28248	-3.48776
H	0.31430	-2.05378	-2.88935
C	0.98660	-3.61838	-4.23383
H	1.87877	-3.89873	-4.82420
H	0.13204	-3.53626	-4.92980
H	0.77240	-4.44156	-3.53111
C	1.39161	-1.11859	-4.48188
H	1.53986	-0.17444	-3.93438
H	0.48779	-1.01528	-5.10798
H	2.24780	-1.27630	-5.16396
C	0.11825	2.00115	0.20165
C	-2.14702	2.84046	-0.66815
C	-2.32917	3.03572	-2.05925
C	-1.14860	3.19652	-3.01514
H	-0.23563	3.29131	-2.40363
C	-0.98318	1.92421	-3.87518
H	-0.83263	1.05255	-3.21504
H	-0.10775	2.02132	-4.54295
H	-1.87518	1.75117	-4.50586
C	-1.26534	4.46677	-3.88515
H	-2.14563	4.42747	-4.55236
H	-0.37219	4.57616	-4.52616
H	-1.35554	5.37707	-3.26641
C	-3.64557	3.02685	-2.56218
H	-3.80839	3.16335	-3.63765
C	-4.74158	2.83581	-1.71335
H	-5.75924	2.83338	-2.12146
C	-4.53562	2.63338	-0.34112
H	-5.39677	2.46725	0.31399
C	-3.24128	2.63221	0.21132
C	-3.01455	2.43287	1.70923
H	-2.04280	1.91338	1.80667

```
C  -2.94736  3.79053  2.44887
H  -2.13773  4.42983  2.05944
H  -2.76262  3.63239  3.52700
H  -3.90088  4.34251  2.34689
C  -4.06728  1.52029  2.36432
H  -5.07054  1.98716  2.39294
H  -3.77783  1.31324  3.40946
H  -4.14046  0.55577  1.83812
C  -0.33909  4.29966  0.11830
H  -0.93667  5.18246 -0.09295
C   0.92115  4.16546  0.61493
H   1.64983  4.90682  0.93191
C   2.48645  2.35031  1.12730
C   3.57221  2.39381  0.22047
C   3.37738  2.70312 -1.26258
H   2.29314  2.79780 -1.43963
C   3.87306  1.53885 -2.14497
H   4.95635  1.35856 -2.01825
H   3.69440  1.76691 -3.21173
H   3.32629  0.61691 -1.88965
C   4.04828  4.03919 -1.65427
H   3.64814  4.88186 -1.06360
H   3.87704  4.26100 -2.72319
H   5.14119  4.00429 -1.49097
C   4.86393  2.13473  0.72254
H   5.71847  2.16078  0.03628
C   5.06862  1.84398  2.07541
H   6.08096  1.65093  2.44967
C   3.97366  1.78409  2.95059
H   4.13861  1.53547  4.00435
C   1.47122  1.97067  3.45013
H   0.58653  1.74315  2.82953
C   1.58748  0.83202  4.48118
H   1.73902 -0.13218  3.97041
H   0.65585  0.76427  5.06974
H   2.41574  0.99160  5.19703
C   1.25898  3.33101  4.15524
H   2.13894  3.60071  4.76899
H   0.37978  3.28898  4.82351
H   1.09017  4.14165  3.42644
```

[18]⁺

```
135
SCF(BS1) = -2426.89564351
H(0 K) = -2425.682883
G(298 K) = -2425.860817
SCF(BS1)+D3(BJ) = -2427.25122401
SCF(PCM=THF) = -2426.94243426
SCF(PCM=C6H5F) = -2426.93941677
SCF(BS2) = -2427.65683257
Lowest Freq. = 11.2669cm-1, 19.6399cm-1, 20.7129cm-1

Ir  0.06703  0.04230 -0.08015
H  -0.97746 -0.14145  1.10240
H   1.40405 -0.10657 -1.32611
H   0.99290  0.59662 -1.54508
H  -1.16284 -0.16999 -0.98419
N   1.47368 -2.74981 -0.30434
N  -0.57196 -2.99297  0.38577
N  -1.50170  2.76585 -0.29695
N   0.52989  3.06798  0.40141
C   0.33622 -2.01479  0.01162
C   1.26943 -4.11690 -0.12933
H   2.05398 -4.84262 -0.32226
C  -0.01612 -4.26836  0.30279
H  -0.58839 -5.15522  0.55849
C   2.75355 -2.22785 -0.74532
C   2.98200 -2.07810 -2.13955
C   4.25525 -1.62371 -2.53709
H   4.47074 -1.49934 -3.60260
C   5.25497 -1.34898 -1.59474
H   6.23904 -1.00506 -1.92930
C   5.00694 -1.53239 -0.22912
H   5.80372 -1.33668  0.49481
C   3.75374 -1.98253  0.23134
C   1.93447 -2.45897 -3.18827
H   0.94920 -2.48600 -2.68873
C   2.22140 -3.87672 -3.74188
H   3.20407 -3.90968 -4.24323
H   1.45355 -4.16546 -4.47966
H   2.22668 -4.63583 -2.94233
C   1.83751 -1.44234 -4.34551
H   1.66241 -0.41710 -3.97935
H   1.00291 -1.71327 -5.01352
H   2.75266 -1.43013 -4.96185
C   3.53872 -2.27608  1.71712
H   2.45154 -2.36463  1.89282
C  -1.95965 -2.83100  0.78548
C  -2.95987 -2.92261 -0.21493
C  -4.30216 -2.88234  0.21081
H  -5.10128 -2.95403 -0.53344
C  -4.62995 -2.77574  1.56756
H  -5.68019 -2.76028  1.87633
C  -3.61758 -2.70280  2.53263
H  -3.88602 -2.63503  3.59150
C  -2.25705 -2.73004  2.16758
C  -2.63056 -3.14204 -1.69237
```

H -1.55837 -2.91948 -1.83849
C -2.86328 -4.62165 -2.08466
H -2.25622 -5.30995 -1.47317
H -2.60121 -4.78691 -3.14378
H -3.92256 -4.90125 -1.95065
C -3.42473 -2.20499 -2.62567
H -4.50608 -2.42452 -2.60283
H -3.08955 -2.34006 -3.66827
H -3.29154 -1.14578 -2.35197
C -1.17271 -2.71618 3.24550
H -0.20567 -2.51788 2.74965
C -1.07552 -4.09627 3.93946
H -0.27535 -4.09231 4.69937
H -0.85660 -4.90249 3.21940
H -2.02243 -4.34940 4.44687
C -1.39054 -1.59842 4.28716
H -2.30593 -1.76259 4.88117
H -1.46946 -0.61047 3.80489
H -0.54486 -1.57176 4.99514
C -0.34524 2.07479 0.00507
C -1.34282 4.13868 -0.09095
H -2.14919 4.83976 -0.28513
C -0.06747 4.32922 0.35466
H 0.46810 5.22784 0.64684
C -2.75084 2.21813 -0.79314
C -2.90777 2.04908 -2.19238
C -4.16177 1.59781 -2.65020
H -4.32219 1.46382 -3.72484
C -5.20985 1.33999 -1.75781
H -6.17827 0.99863 -2.13759
C -5.02752 1.53364 -0.38310
H -5.85882 1.34475 0.30321
C -3.79724 1.98341 0.13462
C -1.81189 2.40230 -3.19883
H -0.88747 2.61877 -2.63460
C -1.50018 1.23473 -4.15953
H -1.20783 0.32665 -3.60579
H -0.67341 1.50923 -4.83690
H -2.36903 0.98098 -4.79071
C -2.18748 3.67988 -3.98704
H -3.10552 3.52580 -4.57983
H -1.37843 3.95569 -4.68490
H -2.36443 4.53518 -3.31368
C -3.64994 2.26422 1.63041
H -2.57592 2.40774 1.84423
C -4.13880 1.09106 2.50455
H -5.22610 0.93048 2.40351
H -3.94110 1.30759 3.56840
H -3.63166 0.14953 2.24046
C -4.38707 3.57091 2.01248
H -4.01349 4.43778 1.44195
H -4.25532 3.78855 3.08617
H -5.46966 3.48418 1.81561
C 1.89949 2.87011 0.83683
C 2.14786 2.69349 2.22274
C 3.49458 2.60958 2.63054
H 3.72438 2.48700 3.69374
C 4.54018 2.70016 1.70383
H 5.57855 2.64143 2.04577
C 4.26329 2.87952 0.34241
H 5.09026 2.96195 -0.36920
C 2.93912 2.97934 -0.12565
C 1.03051 2.66617 3.26602
H 0.06620 2.60046 2.73230
C 1.12561 1.43461 4.19148
H 1.10332 0.49550 3.61375
H 0.27610 1.42356 4.89489
H 2.05010 1.44334 4.79406
C 1.02480 3.97561 4.09087
H 1.96618 4.09444 4.65460
H 0.19486 3.96924 4.81773
H 0.90684 4.86294 3.44661
C 2.66170 3.27628 -1.60035
H 1.60454 3.02359 -1.80295
C 3.53416 2.44000 -2.56051
H 4.59794 2.72750 -2.50128
H 3.21399 2.61078 -3.60231
H 3.46723 1.35982 -2.34914
C 2.84328 4.78683 -1.89097
H 2.17758 5.40811 -1.26954
H 2.62151 5.00551 -2.94942
H 3.88187 5.10096 -1.68928
C 4.18827 -3.63070 2.09430
H 3.78404 -4.46347 1.49535
H 4.00995 -3.86015 3.15863
H 5.27954 -3.60035 1.93187
C 4.06535 -1.15556 2.63671
H 5.16479 -1.06994 2.59043
H 3.80363 -1.37830 3.68501
H 3.63916 -0.17385 2.37450

TS(11b-15b)PMe3
154
SCF(BS1) = -2820.95356359
H(0 K) = -2819.577780
G(298 K) = -2819.775749
SCF(BS1)+D3(BJ) = -2821.39749995
SCF(PCM=THF) = -2821.00299410

SCF(PCM=C6H5F) = -2820.99956400
SCF(BS2) = -4709.01919300
Lowest Freq. = -215.7124cm-1, 6.5413cm-1, 19.3379cm-1

Ir	0.00524	0.03369	0.02119
H	1.02678	0.97007	-0.88696
H	-0.74906	-1.14786	-1.01790
H	-1.46399	-1.86969	1.37302
H	-0.92391	-0.65764	1.27841
H	0.56296	0.98759	1.21109
Zn	0.02742	-0.19675	-2.44995
N	2.28762	-2.11676	-0.38140
N	2.28537	-1.36181	1.66891
N	-2.84126	1.42964	-0.44134
N	-1.22378	2.87202	-0.52378
C	1.60596	-1.21089	0.45105
C	3.31088	-2.78403	0.30486
H	3.96227	-3.49538	-0.19433
C	3.30682	-2.31500	1.57955
H	3.94466	-2.54439	2.42773
C	2.30297	-2.18013	-1.83710
C	1.51776	-3.14874	-2.52374
C	1.65621	-3.23362	-3.92293
H	1.06107	-3.96259	-4.47868
C	2.55162	-2.41375	-4.62049
H	2.63705	-2.49842	-5.70842
C	3.35091	-1.50698	-3.92080
H	4.07878	-0.89575	-4.46418
C	3.26666	-1.38363	-2.51869
C	0.62120	-4.13940	-1.78084
H	0.20884	-3.59647	-0.90857
C	1.44535	-5.34699	-1.26581
H	1.93383	-5.86662	-2.10797
H	0.79048	-6.07884	-0.75999
H	2.22866	-5.04433	-0.55329
C	-0.55377	-4.65719	-2.63463
H	-1.14402	-3.83576	-3.07510
H	-1.22461	-5.28202	-2.02123
H	-0.20389	-5.29968	-3.46051
C	4.27910	-0.48978	-1.80191
H	4.07316	-0.53393	-0.72083
C	2.24888	-0.53419	2.87085
C	1.56543	-1.00159	4.02160
C	1.67915	-0.25248	5.20941
H	1.16283	-0.59291	6.11191
C	2.46240	0.90448	5.26533
H	2.54428	1.46767	6.20052
C	3.15852	1.32576	4.12771
H	3.79169	2.21618	4.18324
C	3.07938	0.61889	2.91178
C	0.80582	-2.32589	4.03685
H	0.55802	-2.56477	2.98680
C	1.69558	-3.46328	4.59741
H	2.60791	-3.60888	3.99785
H	1.14894	-4.42373	4.61550
H	2.00480	-3.23790	5.63248
C	-0.50695	-2.25176	4.84284
H	-0.32050	-2.08134	5.91639
H	-1.05160	-3.21126	4.77916
H	-1.16171	-1.44128	4.48369
C	3.94844	1.05706	1.73434
H	3.52595	0.60352	0.82254
C	5.39838	0.54393	1.91685
H	6.02359	0.84475	1.05816
H	5.44465	-0.55426	2.00115
H	5.85166	0.97202	2.82803
C	3.94836	2.58159	1.51434
H	4.43645	3.12171	2.34461
H	2.92694	2.97225	1.39618
H	4.51148	2.82579	0.59778
C	-1.45060	1.50703	-0.31820
C	-3.42218	2.67759	-0.70056
H	-4.49585	2.79324	-0.81348
C	-2.40732	3.57728	-0.74899
H	-2.40612	4.65107	-0.90777
C	-3.76390	0.33555	-0.21541
C	-4.28059	-0.36224	-1.33801
C	-5.36836	-1.23468	-1.12145
H	-5.80215	-1.76888	-1.97299
C	-5.92787	-1.39862	0.15335
H	-6.79005	-2.05960	0.29211
C	-5.40173	-0.69167	1.24385
H	-5.87548	-0.78523	2.22765
C	-4.32066	0.20108	1.08496
C	-3.76472	-0.12657	-2.75728
H	-2.80718	0.41842	-2.68070
C	-3.48813	-1.44250	-3.51367
H	-2.77057	-2.07503	-2.96496
H	-3.05518	-1.22258	-4.50317
H	-4.40935	-2.02779	-3.67913
C	-4.75145	0.75774	-3.55806
H	-5.73341	0.26343	-3.65968
H	-4.36003	0.94513	-4.57230
H	-4.91345	1.73394	-3.07245
C	-3.87545	1.07120	2.26179
H	-2.96210	1.60867	1.95774
C	-3.51359	0.24236	3.51006

H	-4.37726	-0.32600	3.89849
H	-3.16923	0.90786	4.31944
H	-2.69647	-0.46237	3.28475
C	-4.96062	2.12152	2.60082
H	-5.19302	2.76081	1.73354
H	-4.61763	2.77474	3.42097
H	-5.89877	1.63801	2.92466
C	-0.00120	3.65233	-0.41399
C	0.31329	4.20772	0.85301
C	1.40094	5.10086	0.91878
H	1.66313	5.55270	1.88086
C	2.13586	5.43550	-0.22473
H	2.97525	6.13470	-0.15119
C	1.78136	4.89690	-1.46721
H	2.34115	5.19177	-2.35984
C	0.69399	4.00999	-1.59831
C	-0.53792	3.95620	2.09881
H	-1.20689	3.10519	1.88430
C	0.30249	3.56785	3.33099
H	0.90941	2.67073	3.13460
H	-0.36103	3.35233	4.18629
H	0.97871	4.38307	3.64274
C	-1.41691	5.19306	2.40630
H	-0.79213	6.07650	2.62498
H	-2.05362	5.00507	3.28822
H	-2.07585	5.45078	1.56027
C	0.23427	3.56724	-2.98931
H	-0.38728	2.66086	-2.86894
C	1.39760	3.21081	-3.93654
H	2.01195	4.09414	-4.18266
H	0.99836	2.81866	-4.88638
H	2.05838	2.44488	-3.50285
C	-0.64367	4.66377	-3.64417
H	-1.53801	4.89772	-3.04468
H	-0.98328	4.33811	-4.64255
H	-0.06856	5.59773	-3.76910
C	-0.12626	-0.04220	-4.39262
H	-0.47584	-0.99451	-4.82501
H	0.84921	0.19911	-4.84380
H	-0.84261	0.74970	-4.67209
C	5.71521	-1.02672	-2.01607
H	5.81237	-2.07554	-1.68858
H	6.43711	-0.42228	-1.44110
H	6.01106	-0.97947	-3.07821
C	4.17421	0.98842	-2.22756
H	4.33472	1.11535	-3.31232
H	4.94087	1.58905	-1.70861
H	3.18598	1.40166	-1.96902
P	-2.15537	-3.26772	1.48685
C	-3.48069	-3.43191	2.76038
H	-3.87949	-4.45918	2.74777
H	-3.07671	-3.20302	3.75672
H	-4.28282	-2.71957	2.51644
C	-2.94080	-3.62630	-0.13025
H	-2.20699	-3.43500	-0.92565
H	-3.28068	-4.67325	-0.16995
H	-3.79633	-2.94739	-0.26792
C	-0.94948	-4.62965	1.80808
H	-1.44581	-5.60189	1.65734
H	-0.09958	-4.54079	1.11539
H	-0.57813	-4.56720	2.84113

TS(11b-18)PMe3

154

SCF(BS1) = -2820.94333347

H(0 K) = -2819.564682

G(298 K) = -2819.770328

SCF(BS1)+D3(BJ) = -2821.35913952

SCF(PCM=THF) = -2820.99163764

SCF(PCM=C6H5F) = -2820.98844674

SCF(BS2) = -4709.00847545

Lowest Freq. = -28.1576cm-1, 9.0260cm-1, 18.9068cm-1

Ir	0.62055	0.06928	-0.13342
H	0.83896	0.41532	1.40122
H	-2.49200	-0.36096	-0.13169
H	0.24388	0.05134	-1.91790
H	0.58411	-0.71079	-1.78190
H	2.16121	0.08045	-0.23611
Zn	-3.83712	-0.48240	-0.96888
N	-0.24079	3.07165	-0.73068
N	1.91021	2.88600	-0.59661
N	0.92592	-3.06464	-0.30938
N	0.62726	-2.44266	1.75408
C	0.74975	2.13215	-0.49885
C	0.28532	4.34447	-0.96290
H	-0.35532	5.20034	-1.15390
C	1.63929	4.22525	-0.88112
H	2.43529	4.95563	-0.99134
C	-1.67169	2.86346	-0.70646
C	-2.32540	2.54175	-1.92108
C	-3.72950	2.42329	-1.88290
H	-4.27517	2.19061	-2.80199
C	-4.44041	2.64107	-0.69145
H	-5.53313	2.57246	-0.69373
C	-3.76389	2.99323	0.48385
H	-4.33316	3.19605	1.39679

C	-2.36237	3.12701	0.50315
C	-1.57140	2.42544	-3.24623
H	-0.49645	2.32131	-3.01383
C	-1.74612	3.72009	-4.07779
H	-2.80740	3.88271	-4.33389
H	-1.17689	3.65190	-5.02051
H	-1.39238	4.60982	-3.53077
C	-1.98738	1.19204	-4.07271
H	-1.89627	0.26449	-3.48393
H	-1.34682	1.10291	-4.96657
H	-3.03060	1.26369	-4.42442
C	-1.65733	3.63682	1.76122
H	-0.57026	3.62418	1.57200
C	3.27690	2.44494	-0.40010
C	3.99610	1.94701	-1.51689
C	5.34225	1.58922	-1.30527
H	5.93316	1.20680	-2.14341
C	5.94318	1.73894	-0.04812
H	6.99188	1.45704	0.09142
C	5.21817	2.27984	1.02135
H	5.71180	2.43073	1.98685
C	3.87016	2.66174	0.87022
C	3.39088	1.91011	-2.92179
H	2.29455	1.99471	-2.81887
C	3.88224	3.13229	-3.73693
H	3.63223	4.08437	-3.23960
H	3.41967	3.13966	-4.73881
H	4.97761	3.10146	-3.86918
C	3.67861	0.60210	-3.68517
H	4.75735	0.46254	-3.87282
H	3.18060	0.62652	-4.66948
H	3.30973	-0.28097	-3.13933
C	3.14210	3.38520	2.00638
H	2.08029	3.48498	1.72172
C	3.71945	4.81249	2.17586
H	3.16190	5.36361	2.95236
H	3.66476	5.39348	1.23983
H	4.77913	4.77700	2.48250
C	3.18060	2.62551	3.34758
H	4.21390	2.47281	3.70437
H	2.69341	1.64115	3.26968
H	2.65062	3.20756	4.12095
C	0.71369	-1.92405	0.46603
C	0.96010	-4.21915	0.47220
H	1.13641	-5.19791	0.03653
C	0.77025	-3.82955	1.76363
H	0.74136	-4.39886	2.68763
C	1.31936	-3.13064	-1.70781
C	0.32398	-3.28765	-2.70888

C	0.76556	-3.39184	-4.04313
H	0.02946	-3.50147	-4.84361
C	2.12886	-3.37511	-4.36440
H	2.44384	-3.46315	-5.40926
C	3.08761	-3.27423	-3.35096
H	4.15125	-3.30068	-3.60808
C	2.71006	-3.15899	-1.99799
C	-1.16220	-3.41300	-2.36639
H	-1.36806	-2.74974	-1.50444
C	-2.08818	-2.98828	-3.52178
H	-1.83405	-1.98929	-3.91284
H	-3.13159	-2.95856	-3.17200
H	-2.04829	-3.70403	-4.36154
C	-1.51008	-4.86460	-1.94708
H	-1.28066	-5.56934	-2.76508
H	-2.58736	-4.94652	-1.72174
H	-0.95664	-5.18815	-1.05183
C	3.78773	-3.17639	-0.91160
H	3.31100	-2.92097	0.05047
C	4.90942	-2.14546	-1.14873
H	5.46184	-2.34516	-2.08322
H	5.63984	-2.19047	-0.32306
H	4.51586	-1.11803	-1.19588
C	4.38306	-4.60013	-0.77942
H	3.60759	-5.35315	-0.56120
H	5.12759	-4.63202	0.03428
H	4.88898	-4.90401	-1.71208
C	0.58124	-1.71220	3.00907
C	1.81018	-1.45325	3.66882
C	1.74846	-0.76221	4.89578
H	2.67727	-0.54148	5.43177
C	0.52233	-0.37736	5.45012
H	0.49844	0.15895	6.40439
C	-0.67693	-0.71153	4.80681
H	-1.62884	-0.44589	5.27537
C	-0.67859	-1.40125	3.57891
C	3.15253	-1.98953	3.16507
H	3.00062	-2.39534	2.15006
C	4.24999	-0.91227	3.06034
H	3.96751	-0.11300	2.35786
H	5.18804	-1.36784	2.69925
H	4.46736	-0.45019	4.03880
C	3.62302	-3.15627	4.06860
H	3.81356	-2.80723	5.09817
H	4.56045	-3.59052	3.68094
H	2.87161	-3.96169	4.12569
C	-1.98103	-1.89805	2.95221
H	-1.83094	-1.96205	1.85944
C	-3.17946	-0.96394	3.18875

```
H  -3.46010 -0.90836  4.25550
H  -4.05158 -1.35785  2.64230
H  -2.98622  0.05431  2.81904
C  -2.31765 -3.31549  3.48219
H  -1.52017 -4.04353  3.26413
H  -3.24639 -3.68984  3.01728
H  -2.46747 -3.29712  4.57576
C  -4.90484 -0.87975 -2.58880
H  -5.40357 -1.86364 -2.51541
H  -4.28436 -0.89055 -3.49956
H  -5.69541 -0.12225 -2.73078
C  -2.06417  5.10259  2.04799
H  -1.85193  5.76180  1.18945
H  -1.51127  5.48997  2.92056
H  -3.14211  5.18316  2.27143
C  -1.91166  2.74354  2.99194
H  -2.98608  2.68224  3.23849
H  -1.39467  3.15876  3.87384
H  -1.53306  1.72234  2.82687
P  -6.12542 -0.53214  0.63818
C  -6.57190  0.37649  2.21960
H  -6.53403  1.46211  2.03517
H  -7.58536  0.10789  2.56219
H  -5.84553  0.13522  3.01028
C  -7.61415 -0.10109 -0.41632
H  -7.58858 -0.69388 -1.34314
H  -8.55491 -0.30414  0.12196
H  -7.58265  0.96596 -0.68946
C  -6.55622 -2.29959  1.09755
H  -7.57839 -2.37301  1.50487
H  -6.48119 -2.93290  0.19911
H  -5.84346 -2.67792  1.84740
```

(iv) Fully optimized isomers of [5]$^+$

1,3-stg

159
SCF(BS1) = -3344.71302827
H(0 K) = -3343.310514
G(298 K) = -3343.518448
SCF(BS1)+D3(BJ) = -3345.19905018
SCF(PCM=THF) = -3344.76806008
SCF(PCM=C6H5F) = -3344.76420928
SCF(BS2) = -6450.46812205
Lowest Freq. = 11.9286cm-1, 13.4073cm-1, 17.0413cm-1

```
Ir  0.00110  0.34631  0.00240
H  -1.46356  0.62827 -0.77158
H  -0.00320 -1.42669 -0.02258
H   1.46807  0.59996  0.78547
Zn -1.90065 -1.27240 -0.41289
Zn  1.89528 -1.28678  0.39748
N   0.68272 -0.10724 -3.06089
N   2.05310  1.33444 -2.17162
N  -2.04901  1.30305  2.19154
N  -0.68393 -0.15824  3.05698
C   0.95463  0.53616 -1.85961
C   2.43882  1.17030 -3.50407
H   3.27112  1.72551 -3.92570
C   1.58461  0.26901 -4.06099
H   1.51536 -0.13100 -5.06810
C  -0.47989 -0.89849 -3.43386
C  -0.39617 -2.31584 -3.39730
C   0.84517 -3.04906 -2.89728
H   1.42304 -2.34355 -2.27424
C   0.51258 -4.27273 -2.01985
H  -0.18705 -4.01493 -1.20715
H   1.43731 -4.66606 -1.56683
H   0.05882 -5.08977 -2.60699
C   1.74730 -3.46862 -4.08319
H   1.21895 -4.17473 -4.74706
H   2.65688 -3.96654 -3.70742
H   2.05862 -2.60312 -4.69180
C  -1.47817 -3.04339 -3.93413
H  -1.43268 -4.13674 -3.93902
C  -2.59459 -2.39435 -4.47714
H  -3.42085 -2.98093 -4.89157
C  -2.64714 -0.99615 -4.50581
H  -3.51181 -0.49780 -4.95558
C  -1.58505 -0.21295 -4.01052
C  -1.61346  1.30213 -4.21711
H  -0.73990  1.73468 -3.70135
C  -1.48131  1.63675 -5.72323
H  -0.56780  1.20156 -6.16176
H  -1.44295  2.72938 -5.87069
H  -2.34297  1.25061 -6.29455
C  -2.86768  1.97071 -3.61842
H  -3.79772  1.57713 -4.06366
H  -2.84428  3.05624 -3.81404
H  -2.91683  1.82816 -2.52715
C   2.64680  2.41526 -1.39102
C   3.82734  2.17549 -0.64185
C   4.54385  0.82780 -0.65530
H   3.80077  0.05638 -0.92956
C   5.15157  0.42399  0.70202
H   5.98572  1.08658  0.99053
```

H	5.54998	-0.60128	0.63893
H	4.40470	0.44687	1.51224
C	5.64498	0.81169	-1.74493
H	5.24280	1.03142	-2.74758
H	6.12886	-0.17907	-1.78131
H	6.42091	1.56465	-1.52298
C	4.39597	3.26863	0.04153
H	5.30499	3.11484	0.63034
C	3.83762	4.54952	-0.04242
H	4.30140	5.38490	0.49207
C	2.70927	4.76846	-0.84032
H	2.30647	5.78135	-0.93975
C	2.09246	3.71395	-1.54273
C	0.95228	4.02551	-2.51352
H	0.51043	3.06872	-2.83959
C	-0.18376	4.85277	-1.88038
H	-0.62986	4.33491	-1.01736
H	-0.98098	5.02390	-2.62388
H	0.16622	5.84470	-1.54577
C	1.50951	4.73967	-3.76973
H	1.95532	5.71385	-3.50445
H	0.70133	4.92596	-4.49767
H	2.28908	4.14240	-4.27159
C	-0.95304	0.50625	1.86673
C	-2.43595	1.11783	3.52086
H	-3.26624	1.66915	3.95156
C	-1.58511	0.20429	4.06286
H	-1.51783	-0.21318	5.06301
C	-2.63606	2.40087	1.42978
C	-3.81513	2.18078	0.67230
C	-4.53865	0.83702	0.65966
H	-3.80066	0.05744	0.92408
C	-5.14217	0.45897	-0.70686
H	-5.97177	1.13040	-0.98796
H	-5.54566	-0.56532	-0.66251
H	-4.39143	0.49170	-1.51317
C	-5.64426	0.80842	1.74445
H	-5.24496	1.01001	2.75210
H	-6.13297	-0.18046	1.76281
H	-6.41565	1.56870	1.53180
C	-4.37565	3.28874	0.00634
H	-5.28330	3.14996	-0.58831
C	-3.81116	4.56509	0.11501
H	-4.26856	5.41218	-0.40638
C	-2.68489	4.76388	0.92098
H	-2.27714	5.77273	1.04014
C	-2.07615	3.69382	1.60681
C	-0.93701	3.98341	2.58561
H	-0.51073	3.01878	2.90922

C	-1.49004	4.69842	3.84304
H	-1.91944	5.68106	3.58197
H	-0.68306	4.86771	4.57641
H	-2.28150	4.10998	4.33673
C	0.21390	4.79689	1.96144
H	0.65770	4.27498	1.09958
H	1.00922	4.95570	2.70975
H	-0.12128	5.79391	1.62683
C	0.47502	-0.96055	3.41796
C	0.38377	-2.37683	3.36219
C	-0.85843	-3.09728	2.84585
H	-1.43107	-2.37991	2.23151
C	-1.76687	-3.53291	4.02095
H	-1.24402	-4.25239	4.67474
H	-2.67710	-4.02044	3.63334
H	-2.07684	-2.67651	4.64298
C	-0.52538	-4.30774	1.95023
H	0.17798	-4.03864	1.14442
H	-1.44898	-4.69221	1.48742
H	-0.07555	-5.13489	2.52612
C	1.45938	-3.11733	3.89410
H	1.40772	-4.21037	3.88457
C	2.57739	-2.48192	4.44972
H	3.39818	-3.07863	4.86055
C	2.63878	-1.08452	4.49472
H	3.50509	-0.59689	4.95299
C	1.58322	-0.28889	4.00530
C	1.62127	1.22356	4.22852
H	0.75613	1.66851	3.70917
C	2.88609	1.88856	3.64841
H	3.80907	1.48364	4.09817
H	2.86889	2.97221	3.85462
H	2.94437	1.75667	2.55624
C	1.47797	1.54325	5.73689
H	0.55636	1.11186	6.16198
H	1.44808	2.63454	5.89583
H	2.33069	1.14302	6.31191
H	0.00792	1.95774	0.01968
C	3.31108	-2.60233	0.10886
C	3.67923	-3.56712	1.07512
C	3.98375	-2.64445	-1.13915
C	4.67446	-4.52561	0.81292
H	3.18701	-3.57401	2.05170
C	4.97927	-3.60039	-1.40706
H	3.73826	-1.92210	-1.92757
C	5.32820	-4.54514	-0.42841
H	4.93905	-5.25915	1.58267
H	5.48312	-3.60486	-2.38009
H	6.10393	-5.29036	-0.63298

C -3.32473 -2.58344 -0.14443
C -3.69637 -3.53168 -1.12556
C -3.99902 -2.64102 1.10207
C -4.69668 -4.48907 -0.87907
H -3.20296 -3.52589 -2.10155
C -4.99979 -3.59578 1.35420
H -3.75089 -1.93192 1.90163
C -5.35220 -4.52392 0.36099
H -4.96393 -5.20961 -1.66010
H -5.50493 -3.61238 2.32643
H -6.13200 -5.26813 0.55334

1,3-stg (C2)
159
SCF(BS1) = -3344.71300021
H(0 K) = -3343.310550
G(298 K) = -3343.517850
SCF(BS1)+D3(BJ) = -3345.19911910
SCF(PCM=THF) = -3344.76788673
SCF(PCM=C6H5F) = -3344.76399984
SCF(BS2) = -6450.46809118
Lowest Freq. = 12.0984cm-1, 12.1520cm-1, 18.1678cm-1

Ir 0.00000 0.00000 0.34626
H -1.14054 -1.20605 0.61403
H   0.00000 0.00000 -1.42675
H   1.14054 1.20605 0.61403
Zn -0.89127 -1.72292 -1.28004
Zn 0.89127 1.72292 -1.28004
N -2.76496 1.47732 -0.13326
N -1.55218 2.56082 1.31698
N   1.55218 -2.56082 1.31698
N   2.76496 -1.47732 -0.13326
C -1.54039 1.41749 0.52071
C -2.73042 3.29029 1.14164
H -2.91763 4.20590 1.69433
C -3.48881 2.61566 0.23495
H -4.47398 2.81911 -0.17389
C -3.43019 0.45618 -0.92823
C -3.36027 0.52366 -2.34523
C -2.53671 1.58134 -3.07440
H -1.78486 1.96918 -2.36429
C -1.77419 1.01997 -4.29153
H -1.18558 0.12527 -4.02787
H -1.08292 1.78501 -4.68127
H -2.45678 0.74266 -5.11331
C -3.42979 2.77097 -3.50250
H -4.20667 2.44153 -4.21410
H -2.81745 3.54381 -3.99666
H -3.93774 3.23693 -2.64152
C -4.16348 -0.37457 -3.07753
H -4.14712 -0.33177 -4.17088
C -4.99325 -1.30152 -2.43357
H -5.61091 -1.98599 -3.02392
C -5.04609 -1.34196 -1.03574
H -5.71620 -2.05254 -0.54148
C -4.28879 -0.45162 -0.24801
C -4.50676 -0.42183 1.26542
H -3.77589 0.27839 1.70323
C -5.92248 0.11542 1.58925
H -6.09212 1.11392 1.15288
H -6.06238 0.19239 2.68084
H -6.70384 -0.55802 1.19691
C -4.27696 -1.79141 1.93629
H -4.95619 -2.56520 1.53876
H -4.46595 -1.71491 3.02054
H -3.23948 -2.13621 1.80078
C -0.65124 2.92258 2.40674
C   0.39144 3.85682 2.17777
C   0.58622 4.55083 0.83242
H   0.12495 3.91263 0.05639
C   2.06156 4.76236 0.44093
H   2.56319 5.48368 1.10882
H   2.11885 5.16423 -0.58334
H   2.63456 3.82115 0.46697
C -0.15955 5.90850 0.81166
H -1.23575 5.79607 1.02285
H -0.05413 6.38474 -0.17785
H   0.26045 6.59319 1.56868
C   1.19193 4.21963 3.27907
H   2.00570 4.93607 3.13348
C   0.94778 3.70434 4.55750
H   1.57940 4.00620 5.39925
C -0.12737 2.83344 4.76534
H -0.34216 2.47185 5.77596
C -0.95993 2.42991 3.70237
C -2.20440 1.59293 4.00246
H -2.63957 1.27098 3.04128
C -1.90191 0.31495 4.80946
H -1.19490 -0.34109 4.27852
H -2.83372 -0.25227 4.97585
H -1.48012 0.54587 5.80303
C -3.26128 2.45982 4.73014
H -2.88513 2.80185 5.70974
H -4.18169 1.87750 4.90753
H -3.53180 3.35555 4.14630
C   1.54039 -1.41749 0.52071

```
C   2.73042 -3.29029  1.14164
H   2.91763 -4.20590  1.69433
C   3.48881 -2.61566  0.23495
H   4.47398 -2.81911 -0.17389
C   0.65124 -2.92258  2.40674
C  -0.39144 -3.85682  2.17777
C  -0.58622 -4.55083  0.83242
H  -0.12495 -3.91263  0.05639
C  -2.06156 -4.76236  0.44093
H  -2.56319 -5.48368  1.10882
H  -2.11885 -5.16423 -0.58334
H  -2.63456 -3.82115  0.46697
C   0.15955 -5.90850  0.81166
H   1.23575 -5.79607  1.02285
H   0.05413 -6.38474 -0.17785
H  -0.26045 -6.59319  1.56868
C  -1.19193 -4.21963  3.27907
H  -2.00570 -4.93607  3.13348
C  -0.94778 -3.70434  4.55750
H  -1.57940 -4.00620  5.39925
C   0.12737 -2.83344  4.76534
H   0.34216 -2.47185  5.77596
C   0.95993 -2.42991  3.70237
C   2.20440 -1.59293  4.00246
H   2.63957 -1.27098  3.04128
C   3.26128 -2.45982  4.73014
H   2.88513 -2.80185  5.70974
H   4.18169 -1.87750  4.90753
H   3.53180 -3.35555  4.14630
C   1.90191 -0.31495  4.80946
H   1.19490  0.34109  4.27852
H   2.83372  0.25227  4.97585
H   1.48012 -0.54587  5.80303
C   3.43019 -0.45618 -0.92823
C   3.36027 -0.52366 -2.34523
C   2.53671 -1.58134 -3.07440
H   1.78486 -1.96918 -2.36429
C   3.42979 -2.77097 -3.50250
H   4.20667 -2.44153 -4.21410
H   2.81745 -3.54381 -3.99666
H   3.93774 -3.23693 -2.64152
C   1.77419 -1.01997 -4.29153
H   1.18558 -0.12527 -4.02787
H   1.08292 -1.78501 -4.68127
H   2.45678 -0.74266 -5.11331
C   4.16348  0.37457 -3.07753
H   4.14712  0.33177 -4.17088
C   4.99325  1.30152 -2.43357
H   5.61091  1.98599 -3.02392
C   5.04609  1.34196 -1.03574
H   5.71620  2.05254 -0.54148
C   4.28879  0.45162 -0.24801
C   4.50676  0.42183  1.26542
H   3.77589 -0.27839  1.70323
C   4.27696  1.79141  1.93629
H   4.95619  2.56520  1.53876
H   4.46595  1.71491  3.02054
H   3.23948  2.13621  1.80078
C   5.92248 -0.11542  1.58925
H   6.09212 -1.11392  1.15288
H   6.06238 -0.19239  2.68084
H   6.70384  0.55802  1.19691
H   0.00000  0.00000  1.95787
C   1.00239  3.16548 -2.59341
C   2.03824  3.25906 -3.55167
C  -0.01732  4.15017 -2.64167
C   2.06089  4.28855 -4.50958
H   2.84554  2.52121 -3.55350
C   0.00000  5.18153 -3.59699
H  -0.84747  4.12644 -1.92474
C   1.04260  5.25349 -4.53503
H   2.87815  4.33560 -5.23792
H  -0.80067  5.92953 -3.60634
H   1.06008  6.05605 -5.27970
C  -1.00239 -3.16548 -2.59341
C  -2.03824 -3.25906 -3.55167
C   0.01732 -4.15017 -2.64167
C  -2.06089 -4.28855 -4.50958
H  -2.84554 -2.52121 -3.55350
C   0.00000 -5.18153 -3.59699
H   0.84747 -4.12644 -1.92474
C  -1.04260 -5.25349 -4.53503
H  -2.87815 -4.33560 -5.23792
H   0.80067 -5.92953 -3.60634
H  -1.06008 -6.05605 -5.27970
```

1,3-ecl

159

SCF(BS1) = -3344.71073607

H(0 K) = -3343.308560

G(298 K) = -3343.514806

SCF(BS1)+D3(BJ) = -3345.19578200

SCF(PCM=THF) = -3344.76318253

SCF(PCM=C6H5F) = -3344.75945029

SCF(BS2) = -6450.46714577

Lowest Freq. = 12.7469cm-1, 15.1409cm-1, 19.4728cm-1

Ir	0.24709	-0.08532	-0.32760
H	-0.84107	-0.03097	1.06261
H	1.78403	0.05971	0.29597
Zn	-2.32682	-0.21661	-0.22099
N	-0.39782	-3.19414	-0.14205
N	1.74612	-2.86336	-0.14671
C	0.53748	-2.16841	-0.18102
C	-1.84801	-3.15946	-0.14086
C	-2.51408	-3.27918	1.10969
C	-1.76921	-3.25962	2.44611
H	-0.72157	-2.97106	2.24752
C	-1.75604	-4.66953	3.08457
H	-2.78040	-5.01183	3.31121
H	-1.18731	-4.65942	4.03001
H	-1.29317	-5.41579	2.41780
C	-2.35449	-2.22534	3.43115
H	-2.31557	-1.20554	3.01286
H	-1.78123	-2.22939	4.37414
H	-3.40506	-2.44673	3.68476
C	-3.90369	-3.50591	1.08158
H	-4.44993	-3.60268	2.02488
C	-4.59358	-3.63386	-0.12887
H	-5.67261	-3.81420	-0.12452
C	-3.91006	-3.52341	-1.34566
H	-4.46143	-3.63745	-2.28405
C	-2.52150	-3.29473	-1.38712
C	-1.78063	-3.29652	-2.72567
H	-0.75603	-2.92584	-2.54709
C	-1.67643	-4.73641	-3.28517
H	-1.15713	-5.41137	-2.58485
H	-1.11812	-4.74014	-4.23681
H	-2.67784	-5.15811	-3.47907
C	-2.43129	-2.36081	-3.76606
H	-3.45016	-2.68721	-4.03635
H	-1.83328	-2.35221	-4.69303
H	-2.49277	-1.32470	-3.39342
C	0.20373	-4.45368	-0.09233
H	-0.38703	-5.36387	-0.05913
C	1.54540	-4.24409	-0.09594
H	2.38106	-4.93597	-0.07542
C	3.12349	-2.39433	-0.21708
C	3.85048	-2.23299	0.99311
C	3.23176	-2.46673	2.37619
H	2.14653	-2.25188	2.30206
C	3.39083	-3.94619	2.81249
H	2.88849	-4.64674	2.12795
H	2.96294	-4.09455	3.81878
H	4.45932	-4.21931	2.85579
C	3.82412	-1.55959	3.47488

H	4.86789	-1.83431	3.70546
H	3.24666	-1.67347	4.40643
H	3.80058	-0.49391	3.20025
C	5.22264	-1.92955	0.88338
H	5.81534	-1.79850	1.79292
C	5.84555	-1.81291	-0.36484
H	6.91447	-1.58276	-0.42150
C	5.11094	-2.01695	-1.53761
H	5.61412	-1.95341	-2.50653
C	3.73841	-2.33117	-1.49523
C	2.99853	-2.69446	-2.78515
H	1.91813	-2.54204	-2.61016
C	3.22804	-4.18512	-3.14347
H	4.30239	-4.38340	-3.30130
H	2.69676	-4.43959	-4.07668
H	2.87067	-4.86870	-2.35723
C	3.40477	-1.81531	-3.98413
H	3.34312	-0.74372	-3.74236
H	2.74014	-2.02064	-4.84024
H	4.43400	-2.03280	-4.31949
C	-4.26730	-0.23901	-0.05447
C	-4.88631	-0.17630	1.21554
H	-4.27567	-0.10446	2.12295
C	-6.28562	-0.20096	1.35485
H	-6.73629	-0.15213	2.35277
C	-7.10403	-0.28599	0.21726
H	-8.19421	-0.30465	0.32152
C	-6.51354	-0.34786	-1.05544
H	-7.14356	-0.41601	-1.94974
C	-5.11359	-0.32635	-1.18438
H	-4.68381	-0.38405	-2.19082
H	1.18128	-0.16717	-1.64027
H	-0.94067	-0.29756	-1.51199
Zn	0.89870	0.43417	2.02039
N	1.35074	2.74673	-1.10134
N	-0.69060	2.99293	-0.40033
C	0.26157	2.00719	-0.64807
C	2.60875	2.30346	-1.68212
C	2.65062	2.10331	-3.08609
C	1.42482	2.26374	-3.98737
H	0.54092	2.39314	-3.33964
C	1.55847	3.53460	-4.86032
H	2.42152	3.45945	-5.54408
H	0.65420	3.67616	-5.47673
H	1.69750	4.43977	-4.24570
C	1.15931	1.02201	-4.86438
H	1.00669	0.12334	-4.24541
H	0.25347	1.17931	-5.47484
H	1.99322	0.82423	-5.55962

C	3.90754	1.83080	-3.66182
H	3.97721	1.68324	-4.74461
C	5.06749	1.77348	-2.88027
H	6.03439	1.56863	-3.35114
C	4.99692	2.00369	-1.50119
H	5.91311	1.98161	-0.90417
C	3.77039	2.28970	-0.87037
C	3.73804	2.65954	0.61295
H	2.70094	2.50776	0.97111
C	4.09225	4.15360	0.81728
H	3.39231	4.82524	0.29457
H	4.07006	4.41179	1.89023
H	5.10797	4.36451	0.44047
C	4.66699	1.77929	1.47236
H	5.73067	2.00286	1.27997
H	4.48491	1.97178	2.54312
H	4.50769	0.70722	1.27565
C	1.07580	4.11465	-1.12654
H	1.81060	4.83357	-1.47544
C	-0.19942	4.27041	-0.68397
H	-0.81797	5.15393	-0.55922
C	-2.11503	2.89155	-0.11681
C	-3.00426	2.97731	-1.22483
C	-2.52969	3.02541	-2.67915
H	-1.43766	2.86701	-2.69208
C	-2.80722	4.41606	-3.29908
H	-2.32751	5.22402	-2.72210
H	-2.42145	4.46029	-4.33177
H	-3.88951	4.62817	-3.33557
C	-3.15943	1.91252	-3.54314
H	-4.25887	1.99423	-3.58165
H	-2.78326	1.97545	-4.57845
H	-2.90813	0.91300	-3.15075
C	-4.37862	3.10495	-0.94469
H	-5.08919	3.16633	-1.77466
C	-4.84757	3.17754	0.37001
H	-5.91949	3.28043	0.56298
C	-3.94799	3.11615	1.44088
H	-4.32658	3.19049	2.46426
C	-2.56259	2.98315	1.22914
C	-1.59887	3.03890	2.41323
H	-0.63875	2.59331	2.08746
C	-1.30927	4.50371	2.82257
H	-2.23546	5.00677	3.15043
H	-0.59133	4.52824	3.65987
H	-0.88318	5.08861	1.99069
C	-2.09484	2.23544	3.63195
H	-2.35017	1.19741	3.35877
H	-1.30970	2.19972	4.40361
H	-2.98857	2.69442	4.08823
C	1.27605	0.95606	3.85171
C	1.88792	2.19545	4.15937
H	2.18888	2.87741	3.35466
C	2.11997	2.58520	5.49032
H	2.59571	3.54908	5.70242
C	1.74296	1.74001	6.54634
H	1.92337	2.04128	7.58342
C	1.13200	0.50696	6.26590
H	0.83286	-0.15568	7.08550
C	0.90126	0.12274	4.93358
H	0.41902	-0.84408	4.74371

1,4-ecl (Ci)

159
SCF(BS1) = -3344.71220724
H(0 K) = -3343.310393
G(298 K) = -3343.514853
SCF(BS1)+D3(BJ) = -3345.19354626
SCF(PCM=THF) = -3344.76737298
SCF(PCM=C6H5F) = -3344.76322394
SCF(BS2) = -6450.46855468
Lowest Freq. = 18.5368cm-1, 21.9220cm-1, 24.5938cm-1

Ir	0.00000	0.00000	0.00000
H	-1.64816	0.27481	-0.21859
H	-0.00899	-1.61771	0.47252
Zn	1.94053	1.44044	-0.65905
N	-0.43365	-0.02047	-3.12916
N	0.94343	-1.61091	-2.54897
C	0.13873	-0.61289	-2.00751
C	-1.53145	0.93933	-3.18351
C	-2.85362	0.42544	-3.26345
C	-3.16328	-1.06997	-3.35332
H	-2.26678	-1.62699	-3.02639
C	-3.45333	-1.46697	-4.82241
H	-4.34591	-0.93877	-5.19966
H	-3.64288	-2.55127	-4.90035
H	-2.61240	-1.22009	-5.49086
C	-4.33419	-1.50659	-2.45018
H	-4.16258	-1.24699	-1.39244
H	-4.47408	-2.59889	-2.51808
H	-5.28759	-1.04322	-2.75624
C	-3.90718	1.35489	-3.36976
H	-4.93665	0.98972	-3.43643
C	-3.65999	2.73114	-3.41953
H	-4.49427	3.43466	-3.50630
C	-2.34422	3.20515	-3.39129

H	-2.15687	4.27998	-3.46666
C	-1.24988	2.32426	-3.28571
C	0.17178	2.86880	-3.38014
H	0.85013	2.11657	-2.93422
C	0.59351	3.04352	-4.85965
H	0.53121	2.09731	-5.42276
H	1.63307	3.40809	-4.91791
H	-0.05712	3.77831	-5.36470
C	0.36907	4.19001	-2.61134
H	-0.14405	5.03080	-3.10941
H	1.44163	4.44016	-2.56806
H	-0.01829	4.12189	-1.58109
C	0.02468	-0.61537	-4.30642
H	-0.31509	-0.28001	-5.28167
C	0.88458	-1.60832	-3.94413
H	1.45161	-2.31918	-4.53755
C	1.62357	-2.70036	-1.85605
C	0.91119	-3.91709	-1.67992
C	-0.49792	-4.14600	-2.23009
H	-0.94322	-3.16033	-2.45610
C	-0.42970	-4.94602	-3.55503
H	0.18763	-4.43769	-4.31355
H	-1.44025	-5.08449	-3.97614
H	0.00638	-5.94539	-3.38443
C	-1.43467	-4.85935	-1.23449
H	-1.09517	-5.88474	-1.00942
H	-2.44601	-4.94627	-1.66686
H	-1.51516	-4.31758	-0.27769
C	1.59085	-4.98465	-1.06058
H	1.07003	-5.93587	-0.91419
C	2.92430	-4.86023	-0.65556
H	3.43333	-5.70474	-0.17996
C	3.61550	-3.66660	-0.88807
H	4.66779	-3.58858	-0.60050
C	2.98934	-2.56482	-1.50356
C	3.80853	-1.32552	-1.84959
H	3.10345	-0.48307	-1.98351
C	4.55041	-1.51807	-3.19490
H	5.26426	-2.35726	-3.12800
H	5.11566	-0.60601	-3.45104
H	3.85581	-1.73366	-4.02395
C	4.80643	-0.92796	-0.74424
H	4.32042	-0.88274	0.24461
H	5.23837	0.06077	-0.96946
H	5.64383	-1.64373	-0.67588
C	3.41533	2.42662	-1.46961
C	3.72916	2.19877	-2.83377
H	3.14970	1.47663	-3.42246
C	4.77987	2.88214	-3.47047
H	4.99863	2.68483	-4.52577
C	5.54685	3.81550	-2.75464
H	6.36592	4.34957	-3.24727
C	5.25574	4.05861	-1.40356
H	5.84855	4.78505	-0.83691
C	4.20363	3.37193	-0.77222
H	4.00298	3.58523	0.28255
H	1.64816	-0.27481	0.21859
H	0.00899	1.61771	-0.47252
Zn	-1.94053	-1.44044	0.65905
N	0.43365	0.02047	3.12916
N	-0.94343	1.61091	2.54897
C	-0.13873	0.61289	2.00751
C	1.53145	-0.93933	3.18351
C	2.85362	-0.42544	3.26345
C	3.16328	1.06997	3.35332
H	2.26678	1.62699	3.02639
C	3.45333	1.46697	4.82241
H	4.34591	0.93877	5.19966
H	3.64288	2.55127	4.90035
H	2.61240	1.22009	5.49086
C	4.33419	1.50659	2.45018
H	4.16258	1.24699	1.39244
H	4.47408	2.59889	2.51808
H	5.28759	1.04322	2.75624
C	3.90718	-1.35489	3.36976
H	4.93665	-0.98972	3.43643
C	3.65999	-2.73114	3.41953
H	4.49427	-3.43466	3.50630
C	2.34422	-3.20515	3.39129
H	2.15687	-4.27998	3.46666
C	1.24988	-2.32426	3.28571
C	-0.17178	-2.86880	3.38014
H	-0.85013	-2.11657	2.93422
C	-0.59351	-3.04352	4.85965
H	-0.53121	-2.09731	5.42276
H	-1.63307	-3.40809	4.91791
H	0.05712	-3.77831	5.36470
C	-0.36907	-4.19001	2.61134
H	0.14405	-5.03080	3.10941
H	-1.44163	-4.44016	2.56806
H	0.01829	-4.12189	1.58109
C	-0.02468	0.61537	4.30642
H	0.31509	0.28001	5.28167
C	-0.88458	1.60832	3.94413
H	-1.45161	2.31918	4.53755
C	-1.62357	2.70036	1.85605
C	-0.91119	3.91709	1.67992
C	0.49792	4.14600	2.23009

H 0.94322 3.16033 2.45610
C 0.42970 4.94602 3.55503
H -0.18763 4.43769 4.31355
H 1.44025 5.08449 3.97614
H -0.00638 5.94539 3.38443
C 1.43467 4.85935 1.23449
H 1.09517 5.88474 1.00942
H 2.44601 4.94627 1.66686
H 1.51516 4.31758 0.27769
C -1.59085 4.98465 1.06058
H -1.07003 5.93587 0.91419
C -2.92430 4.86023 0.65556
H -3.43333 5.70474 0.17996
C -3.61550 3.66660 0.88807
H -4.66779 3.58858 0.60050
C -2.98934 2.56482 1.50356
C -3.80853 1.32552 1.84959
H -3.10345 0.48307 1.98351
C -4.55041 1.51807 3.19490
H -5.26426 2.35726 3.12800
H -5.11566 0.60601 3.45104
H -3.85581 1.73366 4.02395
C -4.80643 0.92796 0.74424
H -4.32042 0.88274 -0.24461
H -5.23837 -0.06077 0.96946
H -5.64383 1.64373 0.67588
C -3.41533 -2.42662 1.46961
C -3.72916 -2.19877 2.83377
H -3.14970 -1.47663 3.42246
C -4.77987 -2.88214 3.47047
H -4.99863 -2.68483 4.52577
C -5.54685 -3.81550 2.75464
H -6.36592 -4.34957 3.24727
C -5.25574 -4.05861 1.40356
H -5.84855 -4.78505 0.83691
C -4.20363 -3.37193 0.77222
H -4.00298 -3.58523 -0.28255

1,4-stg
159
SCF(BS1) = -3344.70776931
H(0 K) = -3343.305391
G(298 K) = -3343.509603
SCF(BS1)+D3(BJ) = -3345.19681722
SCF(PCM=THF) = -3344.75963673
SCF(PCM=C6H5F) = -3344.75600646
SCF(BS2) = -6450.46339032
Lowest Freq. = 14.2744cm-1, 18.8349cm-1, 25.1643cm-1

Ir 0.05196 0.23010 0.00789
Zn 2.47584 -0.16236 -0.59201
Zn -2.46012 0.03375 0.24103
N 1.09514 3.19375 -0.11939
N -1.05694 3.16455 -0.37103
N 0.87368 -2.55670 1.27080
N 0.01155 -2.88193 -0.71367
C 0.02033 2.31258 -0.16394
C 0.68882 4.52083 -0.28431
H 1.40125 5.33944 -0.26200
C -0.65951 4.50223 -0.44699
H -1.37995 5.29989 -0.60002
C 2.50446 2.94308 0.11356
C 3.38428 2.97588 -1.00403
C 4.76920 2.95159 -0.73254
H 5.47584 3.00733 -1.56666
C 5.25212 2.88971 0.58020
H 6.33112 2.87740 0.76476
C 4.35904 2.86327 1.65964
H 4.75005 2.84113 2.68131
C 2.96603 2.91297 1.45799
C 2.89083 3.14462 -2.44446
H 1.80572 2.93703 -2.45661
C 3.10376 4.60043 -2.92880
H 2.57955 5.33284 -2.29390
H 2.72937 4.71845 -3.95979
H 4.17629 4.86083 -2.92724
C 3.56576 2.16407 -3.42761
H 4.64343 2.37331 -3.54049
H 3.10937 2.26275 -4.42682
H 3.46325 1.11545 -3.10328
C 2.01788 3.05128 2.64980
H 0.99878 2.81131 2.30052
C 2.34224 2.07907 3.80049
H 2.32715 1.03067 3.46426
H 1.59104 2.18692 4.60091
H 3.32789 2.28527 4.25287
C 2.02074 4.51494 3.15806
H 3.02389 4.80518 3.51566
H 1.31636 4.62904 3.99967
H 1.72545 5.22697 2.36940
C -2.47906 2.88332 -0.42927
C -3.24955 3.14246 0.73907
C -4.65173 3.08640 0.61170
H -5.27647 3.27690 1.48975
C -5.25767 2.80892 -0.61775
H -6.34864 2.77215 -0.69152
C -4.47398 2.57222 -1.75448

H	-4.96372	2.36990	-2.71157
C	-3.06832	2.61537	-1.69671
C	-2.62252	3.54653	2.07585
H	-1.52546	3.47234	1.97782
C	-2.96624	5.01562	2.42046
H	-2.63333	5.71071	1.63176
H	-2.47763	5.31304	3.36373
H	-4.05394	5.15124	2.54798
C	-3.04190	2.60797	3.22745
H	-4.13219	2.62533	3.39540
H	-2.55486	2.91808	4.16758
H	-2.74589	1.56584	3.02005
C	-2.23269	2.48263	-2.97039
H	-1.19293	2.26387	-2.66894
C	-2.23254	3.82051	-3.75092
H	-3.25491	4.09098	-4.06707
H	-1.60893	3.73691	-4.65748
H	-1.83727	4.65121	-3.14279
C	-2.69967	1.32779	-3.87954
H	-2.69249	0.36117	-3.34969
H	-2.02507	1.24108	-4.74799
H	-3.71566	1.49740	-4.27577
C	0.23881	-1.87674	0.22964
C	1.07297	-3.90124	0.94951
H	1.55813	-4.58359	1.64073
C	0.54549	-4.10071	-0.28959
H	0.47385	-4.99501	-0.90056
C	1.13914	-2.10798	2.63704
C	0.03862	-2.01463	3.53453
C	0.31454	-1.66038	4.86890
H	-0.51262	-1.57367	5.57875
C	1.62524	-1.45732	5.31618
H	1.81381	-1.19586	6.36239
C	2.69191	-1.62264	4.42978
H	3.71794	-1.50261	4.79081
C	2.48026	-1.95477	3.07503
C	-1.38432	-2.42043	3.14899
H	-1.51545	-2.24440	2.06606
C	-2.47508	-1.61504	3.88070
H	-2.51850	-1.85944	4.95607
H	-3.46280	-1.85438	3.45488
H	-2.31026	-0.52831	3.78726
C	-1.58480	-3.93432	3.41552
H	-0.86691	-4.55270	2.85158
H	-2.60374	-4.24444	3.12708
H	-1.45187	-4.15775	4.48831
C	3.69643	-2.20343	2.18629
H	3.35577	-2.28023	1.13973
C	4.72428	-1.05411	2.25108
H	4.26363	-0.07868	2.02073
H	5.52822	-1.22780	1.51777
H	5.18985	-0.97991	3.24913
C	4.38194	-3.54333	2.54996
H	4.78122	-3.51598	3.57872
H	5.22197	-3.73831	1.86231
H	3.68861	-4.39912	2.48957
C	-0.93288	-2.86169	-1.83037
C	-2.21198	-3.45576	-1.61591
C	-3.13181	-3.40765	-2.68350
H	-4.12483	-3.84583	-2.54605
C	-2.79244	-2.84160	-3.91707
H	-3.52528	-2.81842	-4.73019
C	-1.49754	-2.35394	-4.12594
H	-1.21752	-1.97569	-5.11345
C	-0.53365	-2.36799	-3.09831
C	-2.57529	-4.27586	-0.37048
H	-1.84762	-4.04772	0.42715
C	-3.98044	-3.99524	0.19915
H	-4.10848	-2.94295	0.49106
H	-4.14743	-4.62795	1.08782
H	-4.77696	-4.24338	-0.52282
C	-2.45494	-5.78651	-0.70441
H	-3.20652	-6.07553	-1.45914
H	-2.63219	-6.39415	0.19928
H	-1.46583	-6.05695	-1.10944
C	0.91357	-2.00916	-3.42127
H	1.43447	-1.80739	-2.46959
C	1.06025	-0.75691	-4.30667
H	0.64714	-0.91618	-5.31758
H	2.12780	-0.51094	-4.43138
H	0.55192	0.11429	-3.86115
C	1.61301	-3.22305	-4.08183
H	1.54961	-4.12449	-3.44892
H	2.67989	-3.00356	-4.25516
H	1.14562	-3.46387	-5.05240
H	0.87135	0.22942	-1.46995
H	1.35595	0.37610	1.04843
H	-0.84313	0.34429	1.42721
H	-1.20382	0.08489	-1.12098
C	4.09586	-1.08705	-1.17828
C	4.00662	-2.47839	-1.43867
C	5.35356	-0.47175	-1.37317
C	5.12017	-3.21852	-1.87421
H	3.05784	-3.00998	-1.29447
C	6.47028	-1.20591	-1.80964
H	5.47136	0.59777	-1.17685
C	6.35697	-2.58201	-2.06258
H	5.01947	-4.29240	-2.06678

H 7.43221 -0.70056 -1.95106
H 7.22599 -3.15436 -2.40351
C -4.35747 -0.36645 0.39095
C -5.08915 -0.27592 1.59858
C -5.07176 -0.72693 -0.77827
C -6.46937 -0.54147 1.64204
H -4.58485 0.01249 2.52689
C -6.45264 -0.98778 -0.74295
H -4.54674 -0.81802 -1.73637
C -7.15502 -0.89772 0.46971
H -7.00966 -0.46804 2.59261
H -6.98035 -1.26406 -1.66292
H -8.22997 -1.10495 0.50129

1,2-stg
159
SCF(BS1) = -3344.70531119
H(0 K) = -3343.301708
G(298 K) = -3343.507695
SCF(BS1)+D3(BJ) = -3345.19149449
SCF(PCM=THF) = -3344.76375326
SCF(PCM=C6H5F) = -3344.75952686
SCF(BS2) = -6450.45856723
Lowest Freq. = 15.9530cm-1, 18.8478cm-1, 22.3504cm-1

Ir 0.50747 0.01631 -0.00220
Zn -1.67111 1.38816 0.30136
Zn -1.57599 -1.49805 -0.28716
N 0.10799 -1.10984 2.93829
N 1.39562 -2.43036 1.77816
N 1.21486 2.51769 -1.78667
N 0.00552 1.11663 -2.93668
C 0.68202 -1.24273 1.67635
C 0.44748 -2.18244 3.76706
H 0.09532 -2.24085 4.79238
C 1.24885 -3.00917 3.04053
H 1.74501 -3.93994 3.29860
C -0.59737 0.03241 3.50120
C -2.01363 -0.00262 3.59663
C -2.64788 1.08800 4.22708
H -3.73720 1.08301 4.33143
C -1.91030 2.15701 4.74896
H -2.42545 2.99250 5.23343
C -0.51222 2.14149 4.67979
H 0.05796 2.96039 5.12877
C 0.18066 1.07572 4.07174
C -2.83923 -1.20772 3.15011
H -2.21627 -1.80657 2.46136
C -3.18643 -2.10736 4.36214
H -2.28609 -2.44727 4.89993
H -3.74064 -2.99987 4.02597
H -3.82052 -1.56300 5.08316
C -4.12925 -0.83091 2.39503
H -4.84786 -0.30241 3.04492
H -4.62354 -1.74256 2.02343
H -3.92685 -0.18566 1.52205
C 1.70666 1.01851 4.15212
H 2.05868 0.26961 3.42206
C 2.39192 2.35156 3.79487
H 2.11511 2.69440 2.78583
H 3.48740 2.22486 3.81933
H 2.14332 3.15138 4.51387
C 2.13990 0.54908 5.56362
H 1.81274 1.26887 6.33381
H 3.23886 0.46848 5.62123
H 1.71301 -0.43451 5.82104
C 2.38853 -2.96735 0.85768
C 3.74069 -2.57986 1.05438
C 4.70474 -3.14230 0.19425
H 5.75615 -2.86231 0.31650
C 4.34798 -4.07668 -0.78486
H 5.11608 -4.50931 -1.43404
C 3.01419 -4.48441 -0.91215
H 2.75142 -5.24554 -1.65293
C 2.00272 -3.94488 -0.09304
C 4.19527 -1.68935 2.21340
H 3.29697 -1.29878 2.72119
C 4.98247 -2.53097 3.24797
H 4.39177 -3.38745 3.61416
H 5.26013 -1.91093 4.11738
H 5.91252 -2.93193 2.80967
C 5.02379 -0.47011 1.76054
H 5.93842 -0.77211 1.22184
H 5.34035 0.11647 2.64012
H 4.43920 0.19112 1.10197
C 0.57675 -4.48288 -0.17595
H -0.08923 -3.74718 0.31158
C 0.45316 -5.80789 0.61569
H 1.09330 -6.58940 0.17081
H -0.59008 -6.16585 0.59597
H 0.75472 -5.68901 1.67006
C 0.06929 -4.67482 -1.61842
H 0.15840 -3.74981 -2.21187
H -0.99378 -4.96529 -1.60121
H 0.62209 -5.47226 -2.14470
C 0.58188 1.28547 -1.68019
C 1.01770 3.08716 -3.04641

```
H   1.44917  4.04877 -3.30763
C   0.26531  2.21039 -3.76662
H  -0.10069  2.24697 -4.78809
C   2.18064  3.11700 -0.87576
C   3.55299  2.81844 -1.08725
C   4.48743  3.44124 -0.23602
H   5.55345  3.23036 -0.36978
C   4.08096  4.34884  0.74889
H   4.82608  4.82941  1.39111
C   2.72492  4.66896  0.89076
H   2.42090  5.41015  1.63599
C   1.74223  4.06632  0.08074
C   4.05211  1.95989 -2.25188
H   3.17547  1.51526 -2.75301
C   4.95810  0.79341 -1.80826
H   5.85721  1.15062 -1.27725
H   5.30227  0.22828 -2.69153
H   4.42117  0.09682 -1.14552
C   4.77693  2.84976 -3.29164
H   4.13127  3.66855 -3.65123
H   5.08454  2.24898 -4.16448
H   5.68443  3.30684 -2.86103
C   0.28543  4.51096  0.17861
H  -0.33645  3.73298 -0.30135
C  -0.21802  4.67164  1.62630
H  -0.05813  3.75705  2.22099
H  -1.29882  4.88753  1.62047
H   0.28373  5.50735  2.14430
C   0.06783  5.82412 -0.61251
H   0.66053  6.64606 -0.17480
H  -0.99619  6.11366 -0.58225
H   0.36553  5.72377 -1.66989
C  -0.62672 -0.06948 -3.49593
C   0.21481 -1.05984 -4.07093
C  -0.41009 -2.16729 -4.67808
H   0.21014 -2.94694 -5.13058
C  -1.80456 -2.27303 -4.74203
H  -2.26648 -3.13922 -5.22628
C  -2.60758 -1.25522 -4.21450
H  -3.69545 -1.32097 -4.31379
C  -2.04263 -0.12688 -3.58445
C   1.73368 -0.90456 -4.15648
H   2.03981 -0.13867 -3.42339
C   2.50414 -2.19290 -3.80891
H   2.25222 -2.55927 -2.80164
H   3.58913 -1.99574 -3.83422
H   2.30630 -3.00256 -4.53265
C   2.13091 -0.40126 -5.56690
H   1.84659 -1.13619 -6.33979
H   3.22243 -0.25160 -5.62797
H   1.64220  0.55497 -5.81734
C  -2.94339  1.01966 -3.12922
H  -2.35515  1.66037 -2.44779
C  -4.19540  0.55554 -2.35880
H  -4.88326 -0.02231 -2.99982
H  -4.74720  1.43116 -1.98148
H  -3.93821 -0.07325 -1.48818
C  -3.36535  1.89249 -4.33701
H  -2.49654  2.29256 -4.88522
H  -3.97511  2.74536 -3.99415
H  -3.96928  1.30594 -5.05079
H   0.08095 -1.20577 -1.07249
H   0.00821  1.20866  1.07015
H   1.79267  0.82273  0.56027
H   1.83964 -0.70195 -0.57461
C  -2.90409 -2.93914 -0.21089
C  -3.90774 -3.08812 -1.19762
C  -2.86637 -3.90956  0.82296
C  -4.83344 -4.14580 -1.15326
H  -3.97133 -2.37401 -2.02496
C  -3.78737 -4.97039  0.87343
H  -2.10370 -3.85236  1.61004
C  -4.77763 -5.08885 -0.11559
H  -5.60021 -4.23200 -1.93121
H  -3.73119 -5.70434  1.68516
H  -5.49894 -5.91180 -0.07787
C  -3.09009  2.74036  0.23281
C  -4.09608  2.82362  1.22483
C  -3.12087  3.71156 -0.80059
C  -5.08856  3.81914  1.18627
H  -4.10909  2.10639  2.05174
C  -4.10886  4.71059 -0.84524
H  -2.36070  3.70375 -1.59211
C  -5.09936  4.76436  0.14917
H  -5.85518  3.85506  1.96828
H  -4.10467  5.44692 -1.65675
H  -5.87254  5.53898  0.11592
```

1,2-stg (C2)

```
159
SCF(BS1) = -3344.70529764
H(0 K) = -3343.301746
G(298 K) = -3343.507008
SCF(BS1)+D3(BJ) = -3345.19171021
SCF(PCM=THF) = -3344.76394826
SCF(PCM=C6H5F) = -3344.75968719
SCF(BS2) = -6450.45853848
```

Lowest Freq. = 16.4654cm-1, 19.1255cm-1, 21.9392cm-1

Ir	0.00000	0.00000	0.50628
Zn	1.44217	0.31490	-1.62400
Zn	-1.44217	-0.31490	-1.62400
N	-1.15652	2.92095	0.05376
N	-2.49994	1.74789	1.30567
N	2.49994	-1.74789	1.30567
N	1.15652	-2.92095	0.05376
C	-1.28856	1.66037	0.63099
C	-2.25210	3.73494	0.35337
H	-2.31488	4.75720	-0.00690
C	-3.09295	3.00032	1.13243
H	-4.04303	3.24685	1.59684
C	0.00000	3.49783	-0.61629
C	0.01149	3.58492	-2.03338
C	1.11187	4.23082	-2.63437
H	1.14179	4.32946	-3.72385
C	2.14641	4.77490	-1.86438
H	2.99022	5.27131	-2.35398
C	2.08545	4.71189	-0.46725
H	2.87715	5.17734	0.12762
C	1.00747	4.08911	0.19289
C	-1.15644	3.11150	-2.89623
H	-1.76431	2.41736	-2.28823
C	-2.06453	4.30452	-3.28438
H	-2.44435	4.84319	-2.40065
H	-2.93163	3.94818	-3.86568
H	-1.51157	5.03019	-3.90560
C	-0.72180	2.35223	-4.16538
H	-0.18034	3.00518	-4.87136
H	-1.60885	1.95946	-4.68726
H	-0.06811	1.49320	-3.93222
C	0.89880	4.17513	1.71574
H	0.14854	3.43653	2.04633
C	2.21328	3.83860	2.44592
H	2.57861	2.83341	2.18420
H	2.05036	3.86484	3.53656
H	3.01106	4.56758	2.22097
C	0.39616	5.58182	2.12669
H	1.11634	6.36043	1.82103
H	0.27719	5.64264	3.22200
H	-0.57555	5.82419	1.66515
C	-3.05363	0.82532	2.28749
C	-2.71047	1.03525	3.64959
C	-3.28805	0.17112	4.60108
H	-3.04193	0.30331	5.65975
C	-4.19490	-0.82527	4.22174
H	-4.63971	-1.47773	4.98006
C	-4.56020	-0.96588	2.87704
H	-5.30068	-1.72053	2.59532
C	-4.00405	-0.14278	1.87792
C	-1.85097	2.20944	4.12418
H	-1.44632	2.72216	3.23497
C	-2.72820	3.23190	4.88788
H	-3.57513	3.58358	4.27501
H	-2.12888	4.11137	5.17898
H	-3.14548	2.78939	5.80872
C	-0.64573	1.77712	4.98365
H	-0.96222	1.23446	5.89102
H	-0.08138	2.66626	5.31367
H	0.04036	1.12858	4.41667
C	-4.49758	-0.24103	0.43700
H	-3.75031	0.25585	-0.20879
C	-5.83105	0.52807	0.26971
H	-6.62431	0.07277	0.88769
H	-6.15665	0.49851	-0.78386
H	-5.73884	1.58539	0.57023
C	-4.65071	-1.68879	-0.06856
H	-3.72053	-2.26785	0.05459
H	-4.90590	-1.68140	-1.14076
H	-5.45821	-2.22418	0.46039
C	1.28856	-1.66037	0.63099
C	3.09295	-3.00032	1.13243
H	4.04303	-3.24685	1.59684
C	2.25210	-3.73494	0.35337
H	2.31488	-4.75720	-0.00690
C	3.05363	-0.82532	2.28749
C	2.71047	-1.03525	3.64959
C	3.28805	-0.17112	4.60108
H	3.04193	-0.30331	5.65975
C	4.19490	0.82527	4.22174
H	4.63971	1.47773	4.98006
C	4.56020	0.96588	2.87704
H	5.30068	1.72053	2.59532
C	4.00405	0.14278	1.87792
C	1.85097	-2.20944	4.12418
H	1.44632	-2.72216	3.23497
C	0.64573	-1.77712	4.98365
H	0.96222	-1.23446	5.89102
H	0.08138	-2.66626	5.31367
H	-0.04036	-1.12858	4.41667
C	2.72820	-3.23190	4.88788
H	3.57513	-3.58358	4.27501
H	2.12888	-4.11137	5.17898
H	3.14548	-2.78939	5.80872
C	4.49758	0.24103	0.43700

H	3.75031	-0.25585	-0.20879
C	4.65071	1.68879	-0.06856
H	3.72053	2.26785	0.05459
H	4.90590	1.68140	-1.14076
H	5.45821	2.22418	0.46039
C	5.83105	-0.52807	0.26971
H	6.62431	-0.07277	0.88769
H	6.15665	-0.49851	-0.78386
H	5.73884	-1.58539	0.57023
C	0.00000	-3.49783	-0.61629
C	-1.00747	-4.08911	0.19289
C	-2.08545	-4.71189	-0.46725
H	-2.87715	-5.17734	0.12762
C	-2.14641	-4.77490	-1.86438
H	-2.99022	-5.27131	-2.35398
C	-1.11187	-4.23082	-2.63437
H	-1.14179	-4.32946	-3.72385
C	-0.01149	-3.58492	-2.03338
C	-0.89880	-4.17513	1.71574
H	-0.14854	-3.43653	2.04633
C	-2.21328	-3.83860	2.44592
H	-2.57861	-2.83341	2.18420
H	-2.05036	-3.86484	3.53656
H	-3.01106	-4.56758	2.22097
C	-0.39616	-5.58182	2.12669
H	-1.11634	-6.36043	1.82103
H	-0.27719	-5.64264	3.22200
H	0.57555	-5.82419	1.66515
C	1.15644	-3.11150	-2.89623
H	1.76431	-2.41736	-2.28823
C	0.72180	-2.35223	-4.16538
H	0.18034	-3.00518	-4.87136
H	1.60885	-1.95946	-4.68726
H	0.06811	-1.49320	-3.93222
C	2.06453	-4.30452	-3.28438
H	2.44435	-4.84319	-2.40065
H	2.93163	-3.94818	-3.86568
H	1.51157	-5.03019	-3.90560
H	-1.19282	-1.08858	0.04442
H	1.19282	1.08858	0.04442
H	0.75446	0.57702	1.81626
H	-0.75446	-0.57702	1.81626
C	-2.84376	-0.26049	-2.99503
C	-2.94997	-1.24935	-4.00192
C	-3.82864	0.76028	-2.98644
C	-3.98052	-1.21939	-4.95831
H	-2.22307	-2.06685	-4.04376
C	-4.86258	0.79632	-3.93818
H	-3.80406	1.54848	-2.22314
C	-4.93868	-0.19448	-4.93080
H	-4.03368	-1.99854	-5.72685
H	-5.60864	1.59818	-3.90409
H	-5.74055	-0.16802	-5.67596
C	2.84376	0.26049	-2.99503
C	2.94997	1.24935	-4.00192
C	3.82864	-0.76028	-2.98644
C	3.98052	1.21939	-4.95831
H	2.22307	2.06685	-4.04376
C	4.86258	-0.79632	-3.93818
H	3.80406	-1.54848	-2.22314
C	4.93868	0.19448	-4.93080
H	4.03368	1.99854	-5.72685
H	5.60864	-1.59818	-3.90409
H	5.74055	0.16802	-5.67596

1,3-stg'

159
SCF(BS1) = -3344.71106543
H(0 K) = -3343.308148
G(298 K) = -3343.513652
SCF(BS1)+D3(BJ) = -3345.19138599
SCF(PCM=THF) = -3344.76185833
SCF(PCM=C6H5F) = -3344.75831822
SCF(BS2) = -6450.46703986
Lowest Freq. = 7.6750cm-1, 17.3207cm-1, 18.0794cm-1

Ir	0.00021	0.37600	0.00045
H	-1.64925	0.69193	0.02805
H	-0.00093	-1.39456	-0.00044
H	1.64983	0.69037	-0.02659
Zn	-1.98695	-1.19963	0.11474
Zn	1.98491	-1.20206	-0.11712
N	-0.86032	-0.12862	-3.01297
N	0.70680	1.37586	-2.91529
N	-0.70619	1.37211	2.91743
N	0.86288	-0.13040	3.01304
C	-0.04350	0.53252	-2.09965
C	0.34338	1.24996	-4.25831
H	0.80289	1.86018	-5.02935
C	-0.63470	0.30954	-4.32167
H	-1.20435	-0.08229	-5.15853
C	-1.99889	-1.00466	-2.78837
C	-1.83046	-2.41251	-2.92533
C	-0.46345	-3.06094	-3.15623
H	0.30056	-2.42121	-2.67523
C	-0.35961	-4.47213	-2.54195
H	-0.65369	-4.48457	-1.47884

H	0.67708	-4.83537	-2.62505
H	-0.99654	-5.19843	-3.07544
C	-0.13249	-3.13959	-4.66850
H	-0.89181	-3.73841	-5.20078
H	0.84768	-3.62393	-4.81593
H	-0.08810	-2.14576	-5.14055
C	-2.99644	-3.20350	-2.91745
H	-2.90671	-4.28859	-3.01023
C	-4.26864	-2.63192	-2.80519
H	-5.15519	-3.27310	-2.80425
C	-4.40874	-1.24469	-2.69708
H	-5.40944	-0.80548	-2.63533
C	-3.28564	-0.39449	-2.70131
C	-3.49184	1.12127	-2.72838
H	-2.50716	1.60446	-2.61062
C	-4.07582	1.55886	-4.09403
H	-3.42979	1.25334	-4.93355
H	-4.18408	2.65616	-4.13035
H	-5.07317	1.11617	-4.25949
C	-4.38195	1.62448	-1.57382
H	-5.39735	1.19375	-1.61609
H	-4.48702	2.72107	-1.62767
H	-3.94016	1.38046	-0.59388
C	1.59713	2.46889	-2.54759
C	3.00162	2.25571	-2.52669
C	3.64189	0.91257	-2.89482
H	3.00151	0.10394	-2.48913
C	5.06126	0.73061	-2.32132
H	5.78553	1.39954	-2.81804
H	5.39969	-0.30210	-2.49953
H	5.10735	0.92641	-1.23752
C	3.71658	0.72546	-4.43288
H	2.72445	0.70089	-4.90743
H	4.22180	-0.22590	-4.67149
H	4.29787	1.54392	-4.89182
C	3.81344	3.37147	-2.23914
H	4.89869	3.24822	-2.20220
C	3.26416	4.64211	-2.02485
H	3.92123	5.49118	-1.81033
C	1.88270	4.83251	-2.12301
H	1.46422	5.83703	-2.00400
C	1.01626	3.75597	-2.40011
C	-0.46770	4.03594	-2.65033
H	-0.99049	3.06828	-2.73671
C	-1.14716	4.81374	-1.50627
H	-1.09465	4.26301	-0.55466
H	-2.21170	4.97841	-1.74616
H	-0.69015	5.80722	-1.35615
C	-0.64273	4.78636	-3.99390
H	-0.15608	5.77635	-3.96005
H	-1.71321	4.94499	-4.20975
H	-0.20372	4.22938	-4.83847
C	0.04444	0.53009	2.10075
C	-0.34159	1.24592	4.26011
H	-0.80145	1.85500	5.03184
C	0.63778	0.30674	4.32216
H	1.20866	-0.08478	5.15833
C	-1.59808	2.46427	2.55107
C	-3.00222	2.24894	2.52945
C	-3.64036	0.90415	2.89511
H	-2.99884	0.09731	2.48762
C	-5.05947	0.72101	2.32146
H	-5.78499	1.38687	2.82049
H	-5.39559	-0.31306	2.49612
H	-5.10617	0.92025	1.23830
C	-3.71418	0.71377	4.43279
H	-2.72179	0.68975	4.90688
H	-4.21786	-0.23885	4.66963
H	-4.29654	1.53039	4.89365
C	-3.81571	3.36384	2.24337
H	-4.90076	3.23894	2.20603
C	-3.26833	4.63565	2.03108
H	-3.92667	5.48403	1.81770
C	-1.88718	4.82803	2.12971
H	-1.47022	5.83334	2.01212
C	-1.01913	3.75242	2.40542
C	0.46445	4.03426	2.65593
H	0.98867	3.06727	2.74133
C	0.63849	4.78365	4.00019
H	0.15046	5.77299	3.96730
H	1.70876	4.94358	4.21614
H	0.20030	4.22529	4.84427
C	1.14271	4.81416	1.51258
H	1.09104	4.26422	0.56047
H	2.20700	4.98024	1.75260
H	0.68416	5.80706	1.36337
C	2.00141	-1.00603	2.78683
C	1.83355	-2.41400	2.92335
C	0.46710	-3.06289	3.15651
H	-0.29790	-2.42337	2.67682
C	0.13875	-3.14176	4.66936
H	0.89924	-3.74031	5.20029
H	-0.84095	-3.62653	4.81846
H	0.09474	-2.14800	5.14157
C	0.36251	-4.47414	2.54251
H	0.65418	-4.48656	1.47875
H	-0.67381	-4.83791	2.62803
H	1.00092	-5.20018	3.07459

C   2.99976 -3.20464 2.91290
H   2.91055 -4.28981 3.00524
C   4.27159 -2.63264 2.79847
H   5.15830 -3.27359 2.79535
C   4.41114 -1.24530 2.69110
H   5.41161 -0.80579 2.62780
C   3.28780 -0.39542 2.69813
C   3.49348 1.12039 2.72672
H   2.50842 1.60339 2.61143
C   4.38128 1.62550 1.57123
H   5.39659 1.19428 1.61043
H   4.48689 2.72195 1.62699
H   3.93716 1.38356 0.59184
C   4.07980 1.55631 4.09191
H   3.43559 1.24910 4.93222
H   4.18743 2.65361 4.12967
H   5.07774 1.11400 4.25481
H   0.00095 1.98837 0.00133
C   3.28045 -2.58175 -0.56812
C   4.67738 -2.36651 -0.62916
C   2.81461 -3.89426 -0.82015
C   5.56682 -3.40839 -0.94542
H   5.08876 -1.37554 -0.41390
C   3.69768 -4.94327 -1.13254
H   1.74254 -4.11471 -0.76599
C   5.07834 -4.69990 -1.20146
H   6.64419 -3.21211 -0.98733
H   3.30736 -5.95024 -1.31875
H   5.77014 -5.51231 -1.44765
C -3.28333 -2.57886 0.56470
C -4.68097 -2.36567 0.61680
C -2.81678 -3.88939 0.82554
C -5.57045 -3.40754 0.93292
H -5.09275 -1.37638 0.39464
C -3.69990 -4.93843 1.13774
H -1.74407 -4.10828 0.77853
C -5.08130 -4.69705 1.19770
H -6.64836 -3.21286 0.96789
H -3.30900 -5.94388 1.33084
H -5.77317 -5.50946 1.44372

1,3-stg' (C2)
159
SCF(BS1) = -3344.71107160
H(0 K) = -3343.308047
G(298 K) = -3343.511570
SCF(BS1)+D3(BJ) = -3345.19137196
SCF(PCM=THF) = -3344.76234024
SCF(PCM=C6H5F) = -3344.75881708
SCF(BS2) = -6450.46704964
Lowest Freq. = 11.2889cm-1, 20.0257cm-1, 21.2064cm-1

Ir 0.00000 0.00000 0.37612
H   0.06851 1.64842 0.69105
H   0.00000 0.00000 -1.39448
H -0.06851 -1.64842 0.69105
Zn 0.00000 1.98900 -1.20118
Zn 0.00000 -1.98900 -1.20118
N   3.05803 0.68491 -0.12931
N   2.87031 -0.87448 1.37462
N -2.87031 0.87448 1.37462
N -3.05803 -0.68491 -0.12931
C   2.09922 -0.07817 0.53158
C   4.23206 -0.58912 1.24861
H   4.97538 -1.09264 1.85849
C   4.35160 0.38404 0.30854
H   5.21985 0.90464 -0.08321
C   2.89922 1.83424 -1.00565
C   3.02668 1.65783 -2.41341
C   3.17947 0.27964 -3.06128
H   2.65646 -0.45565 -2.42088
C   2.55948 0.20988 -4.47207
H   1.51471 0.56363 -4.48410
H   2.58352 -0.83008 -4.83468
H   3.12770 0.81519 -5.19908
C   4.67059 -0.13627 -3.14069
H   5.24452 0.59147 -3.74026
H   4.76213 -1.12345 -3.62457
H   5.14004 -0.20671 -2.14713
C   3.08548 2.82211 -3.20474
H   3.17330 2.72690 -4.28979
C   3.04569 4.09882 -2.63354
H   3.09529 4.98380 -3.27497
C   2.94562 4.24527 -1.24636
H   2.94096 5.24801 -0.80748
C   2.88586 3.12401 -0.39582
C   2.92528 3.32862 1.11989
H   2.75159 2.35239 1.60335
C   4.32233 3.83358 1.55671
H   5.12339 3.14064 1.25071
H   4.36539 3.93956 2.65398
H   4.54427 4.81985 1.11390
C   1.82380 4.28344 1.62343
H   1.92339 5.29444 1.19179
H   1.88460 4.38612 2.71988
H   0.82009 3.89792 1.38066
C   2.45223 -1.74275 2.46725

C	2.34970	-3.14351	2.25313
C	2.67881	-3.80301	0.90917
H	2.30973	-3.13971	0.10146
C	2.02417	-5.18677	0.72686
H	2.47899	-5.93901	1.39472
H	2.18129	-5.53409	-0.30632
H	0.93973	-5.17035	0.92391
C	4.20978	-3.96607	0.72011
H	4.74071	-3.00292	0.69583
H	4.41774	-4.48332	-0.23198
H	4.63540	-4.57363	1.53749
C	2.01643	-3.93808	3.36858
H	1.91654	-5.01928	3.24465
C	1.83534	-3.37820	4.63976
H	1.58377	-4.02234	5.48857
C	2.01354	-2.00489	4.83100
H	1.91968	-1.58092	5.83589
C	2.33961	-1.15520	3.75482
C	2.67558	0.31159	4.03558
H	2.79182	0.82911	3.06820
C	1.57305	1.05571	4.81419
H	0.61995	1.05914	4.26354
H	1.87445	2.10435	4.97981
H	1.39668	0.60725	5.80724
C	4.02726	0.40799	4.78565
H	3.96553	-0.07639	5.77541
H	4.30488	1.46407	4.94475
H	4.84479	-0.07898	4.22819
C	-2.09922	0.07817	0.53158
C	-4.23206	0.58912	1.24861
H	-4.97538	1.09264	1.85849
C	-4.35160	-0.38404	0.30854
H	-5.21985	-0.90464	-0.08321
C	-2.45223	1.74275	2.46725
C	-2.34970	3.14351	2.25313
C	-2.67881	3.80301	0.90917
H	-2.30973	3.13971	0.10146
C	-2.02417	5.18677	0.72686
H	-2.47899	5.93901	1.39472
H	-2.18129	5.53409	-0.30632
H	-0.93973	5.17035	0.92391
C	-4.20978	3.96607	0.72011
H	-4.74071	3.00292	0.69583
H	-4.41774	4.48332	-0.23198
H	-4.63540	4.57363	1.53749
C	-2.01643	3.93808	3.36858
H	-1.91654	5.01928	3.24465
C	-1.83534	3.37820	4.63976
H	-1.58377	4.02234	5.48857

C	-2.01354	2.00489	4.83100
H	-1.91968	1.58092	5.83589
C	-2.33961	1.15520	3.75482
C	-2.67558	-0.31159	4.03558
H	-2.79182	-0.82911	3.06820
C	-4.02726	-0.40799	4.78565
H	-3.96553	0.07639	5.77541
H	-4.30488	-1.46407	4.94475
H	-4.84479	0.07898	4.22819
C	-1.57305	-1.05571	4.81419
H	-0.61995	-1.05914	4.26354
H	-1.87445	-2.10435	4.97981
H	-1.39668	-0.60725	5.80724
C	-2.89922	-1.83424	-1.00565
C	-3.02668	-1.65783	-2.41341
C	-3.17947	-0.27964	-3.06128
H	-2.65646	0.45565	-2.42088
C	-4.67059	0.13627	-3.14069
H	-5.24452	-0.59147	-3.74026
H	-4.76213	1.12345	-3.62457
H	-5.14004	0.20671	-2.14713
C	-2.55948	-0.20988	-4.47207
H	-1.51471	-0.56363	-4.48410
H	-2.58352	0.83008	-4.83468
H	-3.12770	-0.81519	-5.19908
C	-3.08548	-2.82211	-3.20474
H	-3.17330	-2.72690	-4.28979
C	-3.04569	-4.09882	-2.63354
H	-3.09529	-4.98380	-3.27497
C	-2.94562	-4.24527	-1.24636
H	-2.94096	-5.24801	-0.80748
C	-2.88586	-3.12401	-0.39582
C	-2.92528	-3.32862	1.11989
H	-2.75159	-2.35239	1.60335
C	-1.82380	-4.28344	1.62343
H	-1.92339	-5.29444	1.19179
H	-1.88460	-4.38612	2.71988
H	-0.82009	-3.89792	1.38066
C	-4.32233	-3.83358	1.55671
H	-5.12339	-3.14064	1.25071
H	-4.36539	-3.93956	2.65398
H	-4.54427	-4.81985	1.11390
H	0.00000	0.00000	1.98848
C	0.37373	-3.30884	-2.58091
C	0.35114	-4.70698	-2.36600
C	0.65403	-2.85857	-3.89311
C	0.61443	-5.61347	-3.40790
H	0.11110	-5.10501	-1.37526
C	0.91382	-3.75849	-4.94216

H 0.66418 -1.78514 -4.11332
C 0.90002 -5.14085 -4.69912
H 0.59186 -6.69146 -3.21188
H 1.12367 -3.37975 -5.94890
H 1.10499 -5.84593 -5.51157
C -0.37373 3.30884 -2.58091
C -0.35114 4.70698 -2.36600
C -0.65403 2.85857 -3.89311
C -0.61443 5.61347 -3.40790
H -0.11110 5.10501 -1.37526
C -0.91382 3.75849 -4.94216
H -0.66418 1.78514 -4.11332
C -0.90002 5.14085 -4.69912
H -0.59186 6.69146 -3.21188
H -1.12367 3.37975 -5.94890
H -1.10499 5.84593 -5.51157

1,2-ecl
159
SCF(BS1) = -3344.69461949
H(0 K) = -3343.291952
G(298 K) = -3343.497732
SCF(BS1)+D3(BJ) = -3345.17656468
SCF(PCM=THF) = -3344.74887873
SCF(PCM=C6H5F) = -3344.74497996
SCF(BS2) = -6450.44963346
Lowest Freq. = 9.6300cm-1, 15.4935cm-1, 17.4710cm-1

Ir 0.47269 -0.00600 -0.37718
H -0.66671 -0.17378 -1.63312
H 1.54494 0.12734 0.88348
Zn -2.05065 -0.07378 -0.32416
N -0.02915 -3.13460 -0.83550
N 1.73542 -2.80697 0.39149
C 0.69444 -2.11161 -0.22557
C -1.22963 -3.06857 -1.65084
C -2.45056 -3.53588 -1.09116
C -2.54813 -4.15697 0.30737
H -1.62677 -3.90358 0.86190
C -2.62547 -5.70157 0.19009
H -3.55178 -6.00396 -0.32810
H -2.63479 -6.16068 1.19325
H -1.78009 -6.13184 -0.37159
C -3.74611 -3.65265 1.13784
H -3.74389 -2.55859 1.24777
H -3.71755 -4.10985 2.14182
H -4.70950 -3.93660 0.68090
C -3.58265 -3.53481 -1.93200
H -4.54267 -3.87307 -1.53163
C -3.49769 -3.12396 -3.26671
H -4.39161 -3.13524 -3.89888
C -2.26352 -2.73931 -3.80902
H -2.19862 -2.48086 -4.87045
C -1.09545 -2.72442 -3.02314
C 0.27089 -2.51212 -3.67872
H 0.99890 -2.28499 -2.88062
C 0.72139 -3.82551 -4.36700
H 0.76182 -4.66743 -3.65600
H 1.72500 -3.70480 -4.80996
H 0.02440 -4.10311 -5.17643
C 0.30914 -1.34105 -4.67887
H -0.36154 -1.50831 -5.53915
H 1.32914 -1.22739 -5.08387
H 0.02081 -0.39351 -4.19689
C 0.55134 -4.38416 -0.61730
H 0.12240 -5.28995 -1.03315
C 1.65000 -4.17997 0.15458
H 2.38797 -4.86968 0.55146
C 2.93220 -2.32556 1.07119
C 2.92606 -2.20172 2.48637
C 1.68988 -2.50055 3.34002
H 0.79708 -2.18453 2.76325
C 1.55438 -4.02036 3.61588
H 1.43315 -4.60965 2.69361
H 0.67500 -4.21113 4.25442
H 2.44624 -4.39528 4.14729
C 1.67596 -1.74471 4.68367
H 2.43506 -2.14513 5.37822
H 0.69373 -1.86407 5.16752
H 1.85400 -0.66508 4.55927
C 4.14585 -1.86358 3.10599
H 4.17916 -1.75407 4.19299
C 5.31933 -1.68336 2.36286
H 6.25389 -1.42774 2.87276
C 5.30349 -1.85899 0.97612
H 6.23221 -1.75360 0.40714
C 4.11684 -2.20238 0.29806
C 4.17610 -2.53343 -1.19585
H 3.14230 -2.56207 -1.58518
C 4.80264 -3.93352 -1.41521
H 5.83966 -3.95834 -1.03832
H 4.82737 -4.17933 -2.49065
H 4.24082 -4.72899 -0.89983
C 4.95416 -1.48464 -2.01538
H 4.57630 -0.46476 -1.84478
H 4.87521 -1.71340 -3.09194
H 6.02885 -1.48702 -1.76405

C	-3.97172	-0.21347	-0.00535
C	-4.49764	-0.06640	1.30097
H	-3.82475	0.10060	2.15152
C	-5.87947	-0.14350	1.55184
H	-6.25634	-0.02775	2.57428
C	-6.77273	-0.37232	0.49333
H	-7.84946	-0.43519	0.68393
C	-6.27563	-0.52305	-0.81168
H	-6.96689	-0.70256	-1.64330
C	-4.89300	-0.44327	-1.05421
H	-4.53409	-0.56506	-2.08222
H	2.07553	0.01210	-1.14239
H	1.51956	-0.09517	-1.82325
Zn	-0.36996	0.16991	1.92145
N	1.63754	2.87840	-0.64794
N	-0.52285	3.02404	-0.81449
C	0.48865	2.09186	-0.59698
C	3.03836	2.49765	-0.57418
C	3.75463	2.37259	-1.79396
C	3.09736	2.53702	-3.16786
H	2.00211	2.55351	-3.02472
C	3.50895	3.88406	-3.81069
H	4.59805	3.92132	-3.98498
H	3.00671	4.01639	-4.78408
H	3.24405	4.74317	-3.17268
C	3.41817	1.37179	-4.12926
H	3.11375	0.39867	-3.71010
H	2.88535	1.51456	-5.08465
H	4.49561	1.31569	-4.36046
C	5.14587	2.16242	-1.70643
H	5.73181	2.07261	-2.62681
C	5.79122	2.08604	-0.46681
H	6.87382	1.92895	-0.42362
C	5.05893	2.23207	0.71811
H	5.57867	2.19365	1.67965
C	3.66939	2.45894	0.69544
C	2.91167	2.75768	1.98933
H	1.84226	2.54729	1.80499
C	3.04396	4.25735	2.35485
H	2.65536	4.91324	1.55837
H	2.48379	4.47946	3.27946
H	4.10099	4.52457	2.52679
C	3.36334	1.87745	3.17131
H	4.38926	2.12219	3.49696
H	2.70498	2.04677	4.04042
H	3.33404	0.80596	2.91597
C	1.34136	4.21943	-0.89510
H	2.12364	4.96808	-0.97295
C	-0.00946	4.30976	-1.00470
H	-0.66119	5.15476	-1.20179
C	-1.96896	2.87262	-0.88499
C	-2.55596	2.60062	-2.15475
C	-1.72341	2.34925	-3.41433
H	-0.72908	1.98574	-3.09756
C	-1.52475	3.67012	-4.19981
H	-1.01642	4.43881	-3.59529
H	-0.91590	3.49234	-5.10295
H	-2.49678	4.08157	-4.52253
C	-2.33918	1.27804	-4.33783
H	-3.30430	1.60463	-4.76142
H	-1.66576	1.08745	-5.18996
H	-2.50135	0.32265	-3.81121
C	-3.95863	2.67854	-2.24723
H	-4.44338	2.47551	-3.20622
C	-4.74270	3.03646	-1.14517
H	-5.83096	3.09111	-1.24403
C	-4.13902	3.32570	0.08191
H	-4.76347	3.61300	0.93190
C	-2.74109	3.26768	0.24532
C	-2.12145	3.70054	1.57820
H	-1.10230	3.27199	1.63614
C	-1.99687	5.24387	1.65903
H	-2.99062	5.71514	1.56624
H	-1.57346	5.53884	2.63425
H	-1.34887	5.66109	0.87285
C	-2.91852	3.20022	2.80166
H	-3.10563	2.11692	2.76147
H	-2.35544	3.40745	3.72573
H	-3.89086	3.71476	2.88848
C	-0.96192	0.31417	3.77126
C	-0.45771	1.31147	4.64124
H	0.27521	2.04173	4.27540
C	-0.87472	1.39446	5.98082
H	-0.46721	2.17398	6.63435
C	-1.81330	0.47741	6.48249
H	-2.13992	0.54006	7.52579
C	-2.33144	-0.51894	5.63954
H	-3.06481	-1.23629	6.02452
C	-1.90901	-0.59674	4.30012
H	-2.33086	-1.38568	3.66446

1,4-stg'

159

SCF(BS1) = -3344.69180853

H(0 K) = -3343.288559

G(298 K) = -3343.493830

SCF(BS1)+D3(BJ) = -3345.17227436

SCF(PCM=THF) = -3344.74239010

SCF(PCM=C6H5F) = -3344.73885886
SCF(BS2) = -6450.44822981
Lowest Freq. = 5.1300cm-1, 7.0130cm-1, 13.6743cm-1

Ir	-0.00242	-0.08879	0.00019
Zn	-2.55015	-0.02428	-0.09141
Zn	2.54360	-0.04535	0.08738
N	-1.09311	-3.07278	0.05123
N	1.06423	-3.07940	-0.03342
N	-0.02522	2.91798	1.08771
N	0.08421	2.91414	-1.09487
C	-0.01201	-2.19819	0.00557
C	-0.69578	-4.41228	0.04178
H	-1.42262	-5.21811	0.07239
C	0.65916	-4.41645	-0.01402
H	1.38124	-5.22673	-0.03886
C	-2.52230	-2.83660	0.07938
C	-3.23297	-2.96967	-1.14783
C	-4.63695	-3.04207	-1.07221
H	-5.21811	-3.14306	-1.99406
C	-5.29981	-3.01608	0.16025
H	-6.39145	-3.08116	0.19211
C	-4.57452	-2.89692	1.35217
H	-5.10786	-2.88921	2.30780
C	-3.16925	-2.81864	1.34813
C	-2.52712	-3.11713	-2.49796
H	-1.44348	-2.98784	-2.33208
C	-2.75037	-4.53310	-3.08106
H	-2.40025	-5.31942	-2.39175
H	-2.20300	-4.64663	-4.03211
H	-3.81912	-4.71763	-3.28490
C	-2.96331	-2.03722	-3.51166
H	-4.04443	-2.08900	-3.72676
H	-2.42701	-2.17484	-4.46594
H	-2.73298	-1.02426	-3.14040
C	-2.39152	-2.81605	2.66546
H	-1.33085	-2.61729	2.43289
C	-2.86061	-1.70598	3.62964
H	-2.74285	-0.70680	3.17792
H	-2.25746	-1.72861	4.55295
H	-3.91731	-1.83221	3.92196
C	-2.47862	-4.20331	3.34692
H	-3.52167	-4.45362	3.60680
H	-1.88811	-4.21113	4.27885
H	-2.09366	-5.00425	2.69389
C	2.49464	-2.85133	-0.06540
C	3.20663	-2.97765	1.16186
C	4.61017	-3.05745	1.08485
H	5.19217	-3.15351	2.00668
C	5.27133	-3.04501	-0.14871
H	6.36259	-3.11567	-0.18161
C	4.54481	-2.93288	-1.34060
H	5.07672	-2.93623	-2.29706
C	3.13996	-2.84776	-1.33523
C	2.50199	-3.11086	2.51406
H	1.41920	-2.97316	2.34930
C	2.71437	-4.52523	3.10513
H	2.35567	-5.31261	2.42149
H	2.16838	-4.62824	4.05816
H	3.78202	-4.71772	3.30738
C	2.94954	-2.02883	3.52045
H	4.03044	-2.08912	3.73447
H	2.41327	-2.15558	4.47624
H	2.72815	-1.01616	3.14309
C	2.36036	-2.85326	-2.65145
H	1.30070	-2.64907	-2.41904
C	2.44187	-4.24635	-3.32160
H	3.48365	-4.50220	-3.58120
H	1.84974	-4.25990	-4.25242
H	2.05545	-5.04065	-2.66136
C	2.83174	-1.75266	-3.62529
H	2.71706	-0.74946	-3.18179
H	2.22776	-1.78132	-4.54789
H	3.88783	-1.88437	-3.91743
C	0.01811	2.04309	-0.00265
C	0.01372	4.25026	0.67266
H	-0.00817	5.06659	1.38646
C	0.07779	4.24792	-0.68300
H	0.11716	5.06180	-1.39885
C	-0.08959	2.67239	2.53081
C	1.12834	2.63178	3.26845
C	1.03706	2.47347	4.66671
H	1.95249	2.42026	5.25951
C	-0.19613	2.40765	5.31721
H	-0.23916	2.28596	6.40437
C	-1.37422	2.53721	4.57914
H	-2.33003	2.53328	5.10652
C	-1.35991	2.69432	3.17739
C	2.51474	2.84774	2.64730
H	2.53813	2.35412	1.65540
C	3.65405	2.26002	3.50474
H	3.80580	2.84837	4.42637
H	4.59740	2.29825	2.93957
H	3.46571	1.21377	3.79848
C	2.82395	4.35435	2.43945
H	2.15653	4.83439	1.71013
H	3.85719	4.47531	2.07201

H	2.73877	4.89771	3.39656
C	-2.69174	2.96244	2.45986
H	-2.68645	2.41754	1.49449
C	-3.91537	2.49215	3.27428
H	-3.80999	1.45867	3.64415
H	-4.81792	2.54522	2.64667
H	-4.08817	3.14708	4.14618
C	-2.90392	4.47006	2.15667
H	-2.81736	5.06313	3.08370
H	-3.91720	4.62666	1.74934
H	-2.19048	4.86829	1.42238
C	0.12571	2.66365	-2.53790
C	1.38596	2.65765	-3.20432
C	1.37349	2.49606	-4.60572
H	2.32010	2.46999	-5.14871
C	0.18130	2.38970	-5.32478
H	0.20474	2.26441	-6.41213
C	-1.03973	2.48320	-4.65510
H	-1.96590	2.44832	-5.23255
C	-1.10414	2.64615	-3.25602
C	2.73520	2.90050	-2.51033
H	2.73807	2.35381	-1.54597
C	3.93614	2.41093	-3.34714
H	3.81288	1.37601	-3.70767
H	4.85162	2.45906	-2.73821
H	4.09867	3.05850	-4.22643
C	2.97952	4.40335	-2.20750
H	2.88520	5.00096	-3.13087
H	4.00307	4.54095	-1.81950
H	2.28788	4.81142	-1.45793
C	-2.47464	2.89133	-2.61192
H	-2.48604	2.41092	-1.61341
C	-3.63780	2.30882	-3.43994
H	-3.80525	2.89332	-4.36133
H	-4.56819	2.35537	-2.85422
H	-3.46285	1.25994	-3.73221
C	-2.75593	4.40523	-2.41853
H	-2.06451	4.88492	-1.71149
H	-3.77873	4.54781	-2.03020
H	-2.68367	4.93365	-3.38501
H	-1.05733	-0.08324	-1.31994
H	-1.14538	-0.07370	1.24312
H	1.05064	-0.09402	1.32093
H	1.13784	-0.09429	-1.24482
C	-4.48699	0.24309	-0.13812
C	-5.22493	0.22737	-1.34533
C	-5.22977	0.34948	1.06088
C	-6.62570	0.35249	-1.35989
H	-4.70984	0.09548	-2.30018
C	-6.63077	0.47565	1.05893
H	-4.71657	0.31086	2.02492
C	-7.33395	0.48730	-0.15548
H	-7.16353	0.33869	-2.31468
H	-7.17192	0.55784	2.00843
H	-8.42431	0.58937	-0.16305
C	4.47978	0.22167	0.12759
C	5.22012	0.22276	1.33322
C	5.21918	0.31154	-1.07473
C	6.62087	0.34864	1.34272
H	4.70681	0.10238	2.29043
C	6.62013	0.43810	-1.07785
H	4.70302	0.25918	-2.03662
C	7.32608	0.46700	0.13474
H	7.16123	0.34774	2.29619
H	7.15925	0.50683	-2.02959
H	8.41644	0.56934	0.13826

1,4-stg' (C2)

159
SCF(BS1) = -3344.69183375
H(0 K) = -3343.288598
G(298 K) = -3343.492159
SCF(BS1)+D3(BJ) = -3345.17251015
SCF(PCM=THF) = -3344.74238938
SCF(PCM=C6H5F) = -3344.73891566
SCF(BS2) = -6450.44824019
Lowest Freq. = 7.2852cm-1, 8.7355cm-1, 17.1018cm-1

Ir	0.00000	0.00000	0.08788
Zn	0.00000	2.54836	0.03446
Zn	0.00000	-2.54836	0.03446
N	0.09766	1.07514	3.07538
N	-0.09766	-1.07514	3.07538
N	1.09249	0.01977	-2.91676
N	-1.09249	-0.01977	-2.91676
C	0.00000	0.00000	2.19747
C	0.06290	0.67512	4.41365
H	0.12800	1.39716	5.22173
C	-0.06290	-0.67512	4.41365
H	-0.12800	-1.39716	5.22173
C	0.19743	2.50182	2.84321
C	-0.99166	3.27322	2.98520
C	-0.84502	4.67145	3.06015
H	-1.73582	5.29802	3.16796
C	0.41901	5.27148	3.02731
H	0.50622	6.36000	3.09405
C	1.57214	4.48729	2.89952

H	2.55336	4.97184	2.88713
C	1.49697	3.08415	2.81911
C	-2.37514	2.63622	3.13811
H	-2.26511	1.54583	3.00591
C	-2.93990	2.88569	4.55712
H	-2.26608	2.49911	5.33974
H	-3.91711	2.38726	4.67373
H	-3.08816	3.96302	4.74471
C	-3.37065	3.12578	2.06411
H	-3.53170	4.21603	2.12042
H	-4.34973	2.63689	2.20409
H	-3.01561	2.88132	1.04861
C	2.77324	2.24089	2.80976
H	2.48694	1.19444	2.60605
C	3.75856	2.66598	1.70063
H	3.30041	2.57585	0.70156
H	4.65013	2.01672	1.71928
H	4.10438	3.70585	1.83150
C	3.45966	2.28649	4.19660
H	3.77205	3.31386	4.45171
H	4.36060	1.64964	4.20033
H	2.78877	1.93100	4.99642
C	-0.19743	-2.50182	2.84321
C	0.99166	-3.27322	2.98520
C	0.84502	-4.67145	3.06015
H	1.73582	-5.29802	3.16796
C	-0.41901	-5.27148	3.02731
H	-0.50622	-6.36000	3.09405
C	-1.57214	-4.48729	2.89952
H	-2.55336	-4.97184	2.88713
C	-1.49697	-3.08415	2.81911
C	2.37514	-2.63622	3.13811
H	2.26511	-1.54583	3.00591
C	2.93990	-2.88569	4.55712
H	2.26608	-2.49911	5.33974
H	3.91711	-2.38726	4.67373
H	3.08816	-3.96302	4.74471
C	3.37065	-3.12578	2.06411
H	3.53170	-4.21603	2.12042
H	4.34973	-2.63689	2.20409
H	3.01561	-2.88132	1.04861
C	-2.77324	-2.24089	2.80976
H	-2.48694	-1.19444	2.60605
C	-3.45966	-2.28649	4.19660
H	-3.77205	-3.31386	4.45171
H	-4.36060	-1.64964	4.20033
H	-2.78877	-1.93100	4.99642
C	-3.75856	-2.66598	1.70063
H	-3.30041	-2.57585	0.70156
H	-4.65013	-2.01672	1.71928
H	-4.10438	-3.70585	1.83150
C	0.00000	0.00000	-2.04387
C	0.67853	0.00978	-4.24996
H	1.39403	0.01664	-5.06511
C	-0.67853	-0.00978	-4.24996
H	-1.39403	-0.01664	-5.06511
C	2.53646	0.01313	-2.66841
C	3.20943	-1.24163	-2.64112
C	4.61020	-1.22760	-2.48098
H	5.15305	-2.17424	-2.43872
C	5.32411	-0.03114	-2.39987
H	6.41187	-0.04607	-2.27693
C	4.64868	1.18561	-2.51541
H	5.22641	2.11157	-2.49867
C	3.24830	1.24855	-2.67422
C	2.51514	-2.58913	-2.87467
H	1.51118	-2.55480	-2.40713
C	3.28949	-3.77499	-2.26505
H	4.21244	-3.98553	-2.83283
H	2.66937	-4.68292	-2.30889
H	3.57240	-3.59687	-1.21412
C	2.32970	-2.88292	-4.38714
H	1.66090	-2.16710	-4.88568
H	1.89804	-3.88930	-4.52178
H	3.30537	-2.86056	-4.90296
C	2.60308	2.62105	-2.92367
H	1.65003	2.66847	-2.35925
C	3.49481	3.79462	-2.46537
H	3.86767	3.67214	-1.43473
H	2.92188	4.73295	-2.51707
H	4.36884	3.91253	-3.12957
C	2.28126	2.85928	-4.42351
H	3.18791	2.71901	-5.03752
H	1.93410	3.89659	-4.56691
H	1.49528	2.19514	-4.80802
C	-2.53646	-0.01313	-2.66841
C	-3.24830	-1.24855	-2.67422
C	-4.64868	-1.18561	-2.51541
H	-5.22641	-2.11157	-2.49867
C	-5.32411	0.03114	-2.39987
H	-6.41187	0.04607	-2.27693
C	-4.61020	1.22760	-2.48098
H	-5.15305	2.17424	-2.43872
C	-3.20943	1.24163	-2.64112
C	-2.60308	-2.62105	-2.92367
H	-1.65003	-2.66847	-2.35925
C	-3.49481	-3.79462	-2.46537
H	-3.86767	-3.67214	-1.43473

H -2.92188 -4.73295 -2.51707
H -4.36884 -3.91253 -3.12957
C -2.28126 -2.85928 -4.42351
H -3.18791 -2.71901 -5.03752
H -1.93410 -3.89659 -4.56691
H -1.49528 -2.19514 -4.80802
C -2.51514 2.58913 -2.87467
H -1.51118 2.55480 -2.40713
C -3.28949 3.77499 -2.26505
H -4.21244 3.98553 -2.83283
H -2.66937 4.68292 -2.30889
H -3.57240 3.59687 -1.21412
C -2.32970 2.88292 -4.38714
H -1.66090 2.16710 -4.88568
H -1.89804 3.88930 -4.52178
H -3.30537 2.86056 -4.90296
H -1.28196 1.10082 0.08791
H   1.28406 1.09603 0.08211
H   1.28196 -1.10082 0.08791
H -1.28406 -1.09603 0.08211
C   0.04565 4.48493 -0.23153
C -1.11713 5.29066 -0.20443
C   1.28425 5.15740 -0.34948
C -1.05198 6.68991 -0.32992
H -2.09873 4.83139 -0.06292
C   1.36204 6.55625 -0.47570
H   2.21703 4.58871 -0.31972
C   0.18991 7.32788 -0.47615
H -1.97399 7.28190 -0.30729
H   2.34029 7.04202 -0.56654
H   0.24391 8.41692 -0.57830
C -0.04565 -4.48493 -0.23153
C   1.11713 -5.29066 -0.20443
C -1.28425 -5.15740 -0.34948
C   1.05198 -6.68991 -0.32992
H   2.09873 -4.83139 -0.06292
C -1.36204 -6.55625 -0.47570
H -2.21703 -4.58871 -0.31972
C -0.18991 -7.32788 -0.47615
H   1.97399 -7.28190 -0.30729
H -2.34029 -7.04202 -0.56654
H -0.24391 -8.41692 -0.57830

(v) Fully optimized isomers of [13] at BP86/BS1 level.

[13]
136
SCF(BS1) = -2427.66972583
H(0 K) = -2426.451809
G(298 K) = -2426.629731
SCF(BS1)+D3(BJ) = -2428.03080802
SCF(PCM=THF) = -2427.68322217
SCF(PCM=C6H5F) = -2427.68164810
SCF(SMD=THF) = -2427.71506186
SCF(SMD=C6H5F) = -2427.72398206
SCF(BS2) = -2428.43763147
Lowest Freq. = 12.3826cm-1, 20.9243cm-1, 21.2535cm-1

Ir 0.00299 -0.00006 0.00047
H   0.52633 0.03755 -1.56325
H -1.34319 -0.00128 -0.94475
H -1.33873 0.00439 0.95201
H   0.53482 -0.03902 1.56144
H   1.64490 -0.00181 -0.00360
N -1.02370 -2.91823 -0.43042
N   1.01245 -2.94047 0.31580
N -1.01733 2.92067 0.42923
N   1.01927 2.93800 -0.31587
C -0.00300 -2.05349 -0.03804
C -0.64680 -4.26126 -0.32565
H -1.32419 -5.06563 -0.59679
C   0.63083 -4.27529 0.14402
H   1.30635 -5.09451 0.37191
C -2.35106 -2.58087 -0.89880
C -3.40801 -2.52603 0.04325
C -4.71179 -2.32966 -0.45176
H -5.54955 -2.28557 0.25179
C -4.95429 -2.19843 -1.82463
H -5.97725 -2.05336 -2.18865
C -3.88935 -2.25243 -2.73256
H -4.08654 -2.14924 -3.80495
C -2.56495 -2.44354 -2.29235
C -3.17277 -2.72332 1.54056
H -2.09104 -2.60542 1.72395
C -3.58186 -4.15127 1.97439
H -4.65793 -4.32482 1.79479
H -3.39051 -4.29906 3.05183
H -3.02005 -4.92290 1.42131
C -3.89512 -1.66360 2.39680
H -3.60752 -0.64453 2.09424
H -3.63078 -1.79686 3.46043
H -4.99379 -1.75023 2.32145
C -1.42528 -2.53266 -3.30641
H -0.47625 -2.50651 -2.74457
C   2.34287 -2.63114 0.79447
C   2.56589 -2.57082 2.19213

C	3.89374	-2.40609	2.63288
H	4.09911	-2.36247	3.70763
C	4.95269	-2.30362	1.72201
H	5.97817	-2.17994	2.08683
C	4.70112	-2.35931	0.34574
H	5.53403	-2.27807	-0.36040
C	3.39370	-2.52664	-0.15014
C	1.43186	-2.72517	3.20506
H	0.48059	-2.61127	2.65775
C	1.45400	-4.13441	3.84294
H	1.36223	-4.92679	3.08085
H	0.61952	-4.25091	4.55663
H	2.39643	-4.30495	4.39332
C	1.46215	-1.62970	4.29101
H	2.36410	-1.69962	4.92468
H	0.58637	-1.73181	4.95507
H	1.43332	-0.62747	3.83439
C	3.14826	-2.63950	-1.65434
H	2.06244	-2.53785	-1.82160
C	3.58781	-4.02777	-2.17724
H	3.38677	-4.11590	-3.25942
H	3.05232	-4.84507	-1.66490
H	4.66997	-4.18487	-2.02059
C	3.83435	-1.50936	-2.44788
H	4.93536	-1.56451	-2.37756
H	3.51346	-0.52065	-2.08501
H	3.57194	-1.58847	-3.51745
C	0.00174	2.05344	0.03810
C	-0.63761	4.26281	0.32343
H	-1.31334	5.06882	0.59381
C	0.64027	4.27377	-0.14563
H	1.31745	5.09139	-0.37435
C	-2.34520	2.58626	0.89821
C	-3.40255	2.53266	-0.04347
C	-4.70648	2.33882	0.45214
H	-5.54456	2.29562	-0.25107
C	-4.94869	2.20883	1.82518
H	-5.97177	2.06568	2.18963
C	-3.88336	2.26154	2.73273
H	-4.08036	2.15930	3.80524
C	-2.55880	2.45016	2.29192
C	-3.16714	2.72838	-1.54097
H	-2.08616	2.60446	-1.72493
C	-3.89579	1.67262	-2.39675
H	-3.61480	0.65190	-2.09351
H	-3.63018	1.80352	-3.46035
H	-4.99393	1.76625	-2.32200
C	-3.56884	4.15841	-1.97494
H	-4.64385	4.33769	-1.79459
H	-3.37751	4.30482	-3.05257
H	-3.00252	4.92723	-1.42258
C	-1.41856	2.53833	3.30543
H	-0.46980	2.50994	2.74322
C	-1.40601	1.33327	4.26911
H	-2.31171	1.30016	4.90047
H	-0.53584	1.39854	4.94532
H	-1.33587	0.38928	3.70590
C	-1.47567	3.87353	4.08387
H	-1.42124	4.74167	3.40506
H	-0.63431	3.94429	4.79541
H	-2.41372	3.95755	4.66107
C	2.34918	2.62581	-0.79415
C	3.39927	2.51804	0.15100
C	4.70658	2.34826	-0.34425
H	5.53888	2.26447	0.36229
C	4.95890	2.29348	-1.72046
H	5.98435	2.16790	-2.08475
C	3.90080	2.39951	-2.63182
H	4.10668	2.35687	-3.70653
C	2.57301	2.56676	-2.19170
C	3.15306	2.63040	1.65512
H	2.06712	2.52894	1.82173
C	3.83841	1.49990	2.44875
H	3.51746	0.51135	2.08552
H	3.57540	1.57888	3.51819
H	4.93948	1.55475	2.37917
C	3.59277	4.01839	2.17867
H	4.67505	4.17520	2.02255
H	3.39125	4.10621	3.26078
H	3.05773	4.83602	1.66638
C	1.44056	2.72473	-3.20582
H	0.48807	2.62248	-2.65836
C	1.46229	1.62203	-4.28470
H	2.36780	1.67724	-4.91471
H	0.59056	1.73012	-4.95317
H	1.41972	0.62328	-3.82155
C	1.47490	4.12977	-3.85227
H	1.39155	4.92754	-3.09481
H	0.64061	4.24965	-4.56560
H	2.41807	4.28850	-4.40491
C	-1.48500	-3.86689	-4.08630
H	-1.43187	-4.73588	-3.40847
H	-0.64396	-3.93835	-4.79817
H	-2.42334	-3.94864	-4.66334
C	-1.41122	-1.32657	-4.26882
H	-2.31746	-1.29107	-4.89929
H	-0.54182	-1.39280	-4.94592
H	-1.33879	-0.38327	-3.70473

[13]-TS H/H
136
SCF(BS1) = -2427.65223266
H(0 K) = -2426.435281
G(298 K) = -2426.613722
SCF(BS1)+D3(BJ) = -2428.01287544
SCF(PCM=THF) = -2427.66768137
SCF(PCM=C6H5F) = -2427.66578841
SCF(SMD=THF) = -2427.70397285
SCF(SMD=C6H5F) = -2427.71244382
SCF(BS2) = -2428.42210673
Lowest Freq. = -515.9041cm-1, 10.4332cm-1, 15.8949cm-1

```
Ir -0.12291  0.00007 -0.00000
H   1.46909 -0.00007 -0.00118
H  -0.07090  0.02620  1.68325
H  -1.86457  0.42503 -0.02106
H  -1.86461 -0.42456  0.02363
H  -0.07364 -0.02612 -1.68334
N  -0.99253  2.98173  0.30036
N   1.06634  2.87241 -0.37613
N  -0.99320 -2.98136 -0.30065
N   1.06569 -2.87261  0.37586
C  -0.00232  2.04782 -0.02604
C  -0.54923  4.29985  0.15077
H  -1.19463  5.14748  0.36156
C   0.74169  4.23058 -0.27363
H   1.46226  5.00590 -0.51609
C  -2.34153  2.70318  0.73636
C  -3.37694  2.67935 -0.23366
C  -4.69680  2.49732  0.22505
H  -5.51723  2.47725 -0.49987
C  -4.97423  2.34654  1.58875
H  -6.00786  2.20988  1.92490
C  -3.93220  2.37255  2.52512
H  -4.16043  2.25813  3.58972
C  -2.59403  2.55138  2.12413
C  -3.10148  2.88032 -1.72436
H  -2.01328  2.78207 -1.87882
C  -3.52059  4.30154 -2.16988
H  -4.60425  4.45828 -2.02419
H  -3.29738  4.45366 -3.24037
H  -2.98879  5.08067 -1.59813
C  -3.78467  1.80914 -2.60045
H  -3.48751  0.79265 -2.29662
H  -3.49766  1.94952 -3.65705
H  -4.88598  1.87575 -2.54938
C  -1.47654  2.63555  3.16309
H  -0.52223  2.47729  2.63290
C   2.39048  2.49230 -0.81814
C   2.61961  2.31461 -2.20522
C   3.94851  2.10310 -2.62195
H   4.15954  1.97124 -3.68833
C   5.00214  2.06757 -1.69935
H   6.02832  1.90570 -2.04696
C   4.74476  2.23996 -0.33419
H   5.57374  2.21194  0.38056
C   3.43624  2.46148  0.13789
C   1.49296  2.40910 -3.23363
H   0.53870  2.32633 -2.68637
C   1.52812  3.77747 -3.95477
H   1.43814  4.61513 -3.24229
H   0.69755  3.85596 -4.67831
H   2.47384  3.90907 -4.51046
C   1.52157  1.24817 -4.24805
H   2.43805  1.25421 -4.86479
H   0.66278  1.33070 -4.93681
H   1.44955  0.28109 -3.72566
C   3.18502  2.71539  1.62438
H   2.09926  2.62192  1.79797
C   3.61400  4.15000  2.01553
H   3.41416  4.33580  3.08552
H   3.07232  4.91374  1.43258
H   4.69486  4.29850  1.84239
C   3.88128  1.67729  2.52679
H   4.98191  1.75052  2.46611
H   3.58998  0.65160  2.25339
H   3.60175  1.84838  3.58111
C  -0.00277 -2.04773  0.02584
C  -0.55026 -4.29960 -0.15109
H  -1.19590 -5.14705 -0.36188
C   0.74068 -4.23068  0.27331
H   1.46103 -5.00620  0.51579
C  -2.34219 -2.70242 -0.73644
C  -3.37750 -2.67869  0.23369
C  -4.69737 -2.49623 -0.22482
H  -5.51772 -2.47623  0.50019
C  -4.97491 -2.34496 -1.58844
H  -6.00854 -2.20796 -1.92444
C  -3.93298 -2.37092 -2.52493
H  -4.16131 -2.25614 -3.58946
C  -2.59481 -2.55017 -2.12415
C  -3.10199 -2.88026  1.72431
H  -2.01370 -2.78283  1.87869
C  -3.78436 -1.80884  2.60075
H  -3.48646 -0.79247  2.29724
```

H	-3.49743	-1.94975	3.65731
H	-4.88571	-1.87461	2.54968
C	-3.52203	-4.30130	2.16950
H	-4.60582	-4.45725	2.02393
H	-3.29876	-4.45387	3.23991
H	-2.99086	-5.08064	1.59745
C	-1.47743	-2.63442	-3.16322
H	-0.52310	-2.47580	-2.63316
C	-1.57788	-1.53144	-4.23557
H	-2.49109	-1.62576	-4.85002
H	-0.71538	-1.59727	-4.92105
H	-1.56479	-0.53513	-3.76568
C	-1.44929	-4.03855	-3.81322
H	-1.30570	-4.83259	-3.06086
H	-0.62445	-4.10996	-4.54373
H	-2.39373	-4.24702	-4.34712
C	2.38986	-2.49282	0.81811
C	3.43573	-2.46186	-0.13779
C	4.74421	-2.24057	0.33450
H	5.57328	-2.21242	-0.38013
C	5.00143	-2.06854	1.69974
H	6.02759	-1.90683	2.04751
C	3.94770	-2.10423	2.62221
H	4.15863	-1.97266	3.68865
C	2.61884	-2.31553	2.20527
C	3.18461	-2.71546	-1.62436
H	2.09902	-2.62061	-1.79823
C	3.88247	-1.67837	-2.52670
H	3.59282	-0.65223	-2.25323
H	3.60263	-1.84895	-3.58101
H	4.98299	-1.75337	-2.46610
C	3.61190	-4.15064	-2.01535
H	4.69250	-4.30049	-1.84176
H	3.41227	-4.33615	-3.08544
H	3.06899	-4.91369	-1.43267
C	1.49207	-2.41026	3.23354
H	0.53788	-2.32699	2.68623
C	1.52086	-1.24988	4.24859
H	2.43722	-1.25656	4.86550
H	0.66192	-1.33252	4.93715
H	1.44925	-0.28249	3.72673
C	1.52684	-3.77901	3.95398
H	1.43667	-4.61629	3.24108
H	0.69622	-3.85765	4.67745
H	2.47250	-3.91113	4.50964
C	-1.44860	4.03949	3.81351
H	-1.30531	4.83379	3.06135
H	-0.62366	4.11088	4.54391
H	-2.39301	4.24762	4.34762
C	-1.57669	1.53221	4.23510
H	-2.48999	1.62602	4.84949
H	-0.71426	1.59811	4.92066
H	-1.56321	0.53605	3.76489

(vi) Fully optimized isomers of [13] at B3LYP/BS1 level.

[13]
136
SCF(BS1) = -2427.62679237
H(0 K) = -2426.375805
G(298 K) = -2426.549315
SCF(BS1)+D3(BJ) = -2427.99532388
SCF(PCM=THF) = -2427.64007372
SCF(PCM=C6H5F) = -2427.63852338
SCF(SMD=THF) = -2427.67170562
SCF(SMD=C6H5F) = -2427.68071036
SCF(BS2) = -2428.39863272
Lowest Freq. = 13.6525cm-1, 16.6303cm-1, 22.3177cm-1

Ir	0.00212	0.00000	0.00002
H	0.51823	-0.02852	1.55821
H	-1.33495	-0.00604	0.94416
H	-1.33062	0.00517	-0.95032
H	0.52565	0.02896	-1.55567
H	1.63604	0.00049	0.00375
N	-1.01421	2.92409	0.42322
N	1.00942	2.94175	-0.32023
N	-1.01219	-2.92476	-0.42314
N	1.01145	-2.94108	0.32032
C	-0.00113	2.06962	0.03421
C	-0.63589	4.26086	0.31526
H	-1.30548	5.06198	0.58124
C	0.63025	4.27197	-0.15119
H	1.29997	5.08480	-0.37850
C	-2.33996	2.59336	0.89109
C	-3.39289	2.54636	-0.04392
C	-4.68888	2.34707	0.44766
H	-5.52155	2.30828	-0.24773
C	-4.92855	2.20725	1.81151
H	-5.94282	2.05977	2.17249
C	-3.86968	2.25869	2.71385
H	-4.06708	2.15181	3.77617
C	-2.55342	2.45179	2.27638
C	-3.16624	2.75350	-1.53841
H	-2.09447	2.65423	-1.72826
C	-3.59446	4.17257	-1.96643

H	-4.66474	4.33015	-1.79106
H	-3.40340	4.32573	-3.03443
H	-3.04911	4.94409	-1.41362
C	-3.87177	1.68967	-2.39654
H	-3.57495	0.68214	-2.09725
H	-3.60640	1.82673	-3.45079
H	-4.96286	1.76203	-2.32551
C	-1.41869	2.54100	3.29192
H	-0.47549	2.49925	2.74183
C	2.33624	2.63342	-0.79999
C	2.55455	2.55917	-2.18992
C	3.87257	2.38682	-2.63103
H	4.07459	2.33178	-3.69629
C	4.92813	2.29134	-1.72831
H	5.94357	2.16091	-2.09252
C	4.68369	2.36580	-0.36028
H	5.51356	2.29363	0.33596
C	3.38592	2.54178	0.13525
C	1.42335	2.70734	-3.20287
H	0.47900	2.59803	-2.66384
C	1.44286	4.10893	-3.84694
H	1.35429	4.89929	-3.09470
H	0.61109	4.22003	-4.55151
H	2.37485	4.27649	-4.39862
C	1.44990	1.61024	-4.28152
H	2.33787	1.68133	-4.91945
H	0.57365	1.70516	-4.93201
H	1.42960	0.61717	-3.82571
C	3.15462	2.67477	1.63725
H	2.07902	2.60137	1.81587
C	3.62578	4.05315	2.14486
H	3.42852	4.15518	3.21782
H	3.11217	4.87104	1.62921
H	4.70243	4.18457	1.98813
C	3.81696	1.53976	2.43648
H	4.90991	1.57000	2.36374
H	3.47649	0.56349	2.08543
H	3.55980	1.63241	3.49766
C	0.00034	-2.06962	-0.03415
C	-0.63300	-4.26128	-0.31515
H	-1.30207	-5.06284	-0.58111
C	0.63315	-4.27156	0.15131
H	1.30340	-5.08394	0.37864
C	-2.33813	-2.59489	-0.89107
C	-3.39113	-2.54855	0.04388
C	-4.68723	-2.35012	-0.44776
H	-5.51996	-2.31186	0.24758
C	-4.92692	-2.21047	-1.81163
H	-5.94126	-2.06366	-2.17266
C	-3.86796	-2.26122	-2.71392
H	-4.06539	-2.15447	-3.77624
C	-2.55161	-2.45346	-2.27637
C	-3.16442	-2.75553	1.53838
H	-2.09272	-2.65551	1.72829
C	-3.87074	-1.69220	2.39649
H	-3.57461	-0.68446	2.09721
H	-3.60533	-1.82907	3.45074
H	-4.96177	-1.76532	2.32540
C	-3.59167	-4.17491	1.96638
H	-4.66183	-4.33323	1.79096
H	-3.40055	-4.32793	3.03439
H	-3.04574	-4.94604	1.41360
C	-1.41675	-2.54194	-3.29185
H	-0.47362	-2.49943	-2.74171
C	-1.41358	-1.35355	-4.26958
H	-2.30605	-1.34182	-4.90516
H	-0.54278	-1.41626	-4.93135
H	-1.36139	-0.40727	-3.72579
C	-1.46167	-3.88288	-4.05236
H	-1.39905	-4.73553	-3.36837
H	-0.62534	-3.95362	-4.75670
H	-2.39099	-3.98264	-4.62456
C	2.33811	-2.63192	0.80000
C	3.38768	-2.53975	-0.13531
C	4.68538	-2.36297	0.36012
H	5.51516	-2.29036	-0.33618
C	4.92987	-2.28829	1.72813
H	5.94526	-2.15724	2.09227
C	3.87442	-2.38434	2.63092
H	4.07648	-2.32913	3.69616
C	2.55647	-2.55748	2.18991
C	3.15639	-2.67291	-1.63730
H	2.08070	-2.60064	-1.81583
C	3.81745	-1.53711	-2.43646
H	3.47583	-0.56126	-2.08537
H	3.56043	-1.63001	-3.49766
H	4.91043	-1.56609	-2.36367
C	3.62896	-4.05075	-2.14503
H	4.70577	-4.18105	-1.98844
H	3.43167	-4.15295	-3.21797
H	3.11629	-4.86921	-1.62935
C	1.42544	-2.70627	3.20295
H	0.48097	-2.59763	2.66398
C	1.45137	-1.60902	4.28146
H	2.33944	-1.67943	4.91931
H	0.57524	-1.70442	4.93204
H	1.43037	-0.61601	3.82552
C	1.44589	-4.10776	3.84718

H 1.35777 -4.89827 3.09505
H 0.61424 -4.21931 4.55183
H 2.37802 -4.27466 4.39883
C -1.46463 3.88182 4.05258
H -1.40260 4.73459 3.36868
H -0.62837 3.95310 4.75696
H -2.39404 3.98083 4.62475
C -1.41470 1.35250 4.26950
H -2.30722 1.34002 4.90500
H -0.54400 1.41578 4.93136
H -1.36176 0.40632 3.72560

[13](η^2H_2)H_3 isomer
136
SCF(BS1) = -2427.62162394
H(0 K) = -2426.370475
G(298 K) = -2426.541989
SCF(BS1)+D3(BJ) = -2427.99034253
SCF(PCM=THF) = -2427.63576901
SCF(PCM=C6H5F) = -2427.63398600
SCF(SMD=THF) = -2427.66923835
SCF(SMD=C6H5F) = -2427.67799360
SCF(BS2) = -2428.39499775
Lowest Freq. = 13.9968cm-1, 21.2293cm-1, 22.8873cm-1

Ir 0.04852 0.00004 0.00002
H -1.54499 0.00023 0.00060
H -0.13301 0.03639 1.66166
H 1.73584 0.00209 0.44732
H 1.73556 -0.00244 -0.44837
H -0.13417 -0.03624 -1.66151
N -1.03506 -2.91743 -0.28523
N 1.00143 -2.94224 0.42395
N -1.03426 2.91779 0.28536
N 1.00222 2.94211 -0.42386
C -0.00119 -2.06304 0.04659
C -0.68077 -4.25531 -0.11628
H -1.37216 -5.05412 -0.32778
C 0.59309 -4.27161 0.32711
H 1.24899 -5.08679 0.58465
C -2.36053 -2.58699 -0.75204
C -3.40228 -2.49832 0.19225
C -4.70320 -2.31006 -0.29074
H -5.52699 -2.23933 0.41288
C -4.95814 -2.22405 -1.65605
H -5.97580 -2.08562 -2.01110
C -3.91035 -2.31820 -2.56819
H -4.12104 -2.25452 -3.63126
C -2.58949 -2.50041 -2.13977
C -3.15891 -2.65068 1.69052
H -2.08102 -2.59100 1.86024
C -3.64146 -4.02888 2.18791
H -4.72092 -4.14619 2.03939
H -3.43609 -4.14430 3.25801
H -3.14215 -4.84753 1.65963
C -3.80168 -1.51763 2.50743
H -3.45393 -0.54169 2.16341
H -3.53660 -1.62386 3.56539
H -4.89551 -1.53463 2.44324
C -1.46721 -2.65225 -3.16202
H -0.51923 -2.54058 -2.63039
C 2.33753 -2.62358 0.86407
C 2.57156 -2.42098 2.23976
C 3.89427 -2.20580 2.64729
H 4.10824 -2.05217 3.70029
C 4.93986 -2.19363 1.72774
H 5.95876 -2.02782 2.06705
C 4.68204 -2.39883 0.37563
H 5.50506 -2.39295 -0.33226
C 3.37869 -2.62007 -0.08725
C 1.45114 -2.48608 3.27318
H 0.50314 -2.39301 2.73841
C 1.46148 -3.84684 4.00019
H 1.35518 -4.68010 3.29818
H 0.63571 -3.90423 4.71798
H 2.39706 -3.99265 4.55224
C 1.50347 -1.32551 4.28112
H 2.40165 -1.36021 4.90793
H 0.63828 -1.37726 4.95080
H 1.47227 -0.36379 3.76341
C 3.13155 -2.89237 -1.56881
H 2.05603 -2.81381 -1.74759
C 3.56712 -4.32484 -1.94132
H 3.36020 -4.52660 -2.99809
H 3.03785 -5.07525 -1.34586
H 4.64143 -4.46488 -1.77644
C 3.81640 -1.86294 -2.48512
H 4.90874 -1.92389 -2.42608
H 3.51951 -0.84200 -2.23232
H 3.53704 -2.04978 -3.52777
C -0.00063 2.06315 -0.04656
C -0.67960 4.25560 0.11653
H -1.37077 5.05458 0.32810
C 0.59425 4.27158 -0.32689
H 1.25038 5.08660 -0.58437
C -2.35986 2.58769 0.75202
C -3.40154 2.49936 -0.19236

C -4.70255 2.31131 0.29049
H -5.52628 2.24081 -0.41322
C -4.95763 2.22526 1.65577
H -5.97536 2.08702 2.01072
C -3.90991 2.31913 2.56801
H -4.12071 2.25542 3.63107
C -2.58897 2.50105 2.13972
C -3.15806 2.65171 -1.69061
H -2.08013 2.59253 -1.86022
C -3.80020 1.51819 -2.50738
H -3.45187 0.54251 -2.16321
H -3.53519 1.62442 -3.56535
H -4.89403 1.53457 -2.44314
C -3.64122 4.02960 -2.18823
H -4.72077 4.14640 -2.03992
H -3.43574 4.14499 -3.25831
H -3.14241 4.84856 -1.65995
C -1.46676 2.65250 3.16210
H -0.51874 2.54086 2.63053
C -1.49813 1.55623 4.24087
H -2.39711 1.61520 4.86461
H -0.63382 1.66353 4.90551
H -1.45356 0.56525 3.78281
C -1.49554 4.05534 3.80313
H -1.40278 4.84476 3.05027
H -0.66997 4.17004 4.51454
H -2.43236 4.22177 4.34706
C 2.33822 2.62311 -0.86403
C 3.37942 2.61939 0.08724
C 4.68269 2.39779 -0.37568
H 5.50574 2.39173 0.33219
C 4.94042 2.19249 -1.72779
H 5.95926 2.02640 -2.06713
C 3.89479 2.20490 -2.64729
H 4.10868 2.05118 -3.70030
C 2.57215 2.42041 -2.23972
C 3.13244 2.89186 1.56880
H 2.05689 2.81385 1.74761
C 3.81678 1.86208 2.48510
H 3.51925 0.84130 2.23239
H 3.53764 2.04914 3.52778
H 4.90915 1.92237 2.42594
C 3.56876 4.32410 1.94128
H 4.64313 4.46360 1.77639
H 3.36194 4.52599 2.99805
H 3.03987 5.07478 1.34581
C 1.45173 2.48571 -3.27312
H 0.50369 2.39331 -2.73830
C 1.50346 1.32473 -4.28063
H 2.40180 1.35857 -4.90725
H 0.63845 1.37682 -4.95051
H 1.47147 0.36323 -3.76254
C 1.46272 3.84619 -4.00064
H 1.35700 4.67977 -3.29893
H 0.63687 3.90377 -4.71833
H 2.39829 3.99128 -4.55289
C -1.49610 -4.05526 -3.80269
H -1.40354 -4.84450 -3.04963
H -0.67046 -4.17025 -4.51398
H -2.43289 -4.22169 -4.34669
C -1.49835 -1.55630 -4.24111
H -2.39726 -1.61536 -4.86494
H -0.63397 -1.66390 -4.90562
H -1.45372 -0.56518 -3.78335

[13]-TS H/H
136
SCF(BS1) = -2427.61271404
H(0 K) = -2426.362417
G(298 K) = -2426.534889
SCF(BS1)+D3(BJ) = -2427.98109082
SCF(PCM=THF) = -2427.62810664
SCF(PCM=C6H5F) = -2427.62621615
SCF(SMD=THF) = -2427.66492781
SCF(SMD=C6H5F) = -2427.67336982
SCF(BS2) = -2428.38594100
Lowest Freq. = -526.5992cm-1, 13.1901cm-1, 18.9276cm-1

Ir -0.10769 -0.00000 -0.00000
H 1.47094 0.00000 -0.00001
H -0.06114 0.00861 1.68021
H -1.88385 0.40625 -0.01409
H -1.88385 -0.40625 0.01409
H -0.06115 -0.00861 -1.68022
N -0.98683 2.97810 0.33893
N 1.05266 2.88540 -0.35615
N -0.98682 -2.97810 -0.33892
N 1.05267 -2.88539 0.35615
C -0.00208 2.06350 -0.00648
C -0.54965 4.29430 0.20250
H -1.18924 5.13159 0.42813
C 0.72569 4.23551 -0.23191
H 1.43531 5.01172 -0.46564
C -2.33120 2.69712 0.77677
C -3.36518 2.68300 -0.18341
C -4.67524 2.48990 0.27403

H	-5.49239	2.47639	-0.44059
C	-4.94596	2.32150	1.62844
H	-5.96949	2.17651	1.96334
C	-3.90775	2.34379	2.55658
H	-4.13278	2.21879	3.61095
C	-2.57938	2.53262	2.15590
C	-3.10271	2.90709	-1.67049
H	-2.02497	2.82947	-1.83522
C	-3.54266	4.32318	-2.09583
H	-4.61987	4.46211	-1.95019
H	-3.32267	4.49112	-3.15582
H	-3.02596	5.09627	-1.51839
C	-3.77049	1.84048	-2.55698
H	-3.46060	0.83241	-2.27018
H	-3.48794	1.99741	-3.60365
H	-4.86375	1.88960	-2.50451
C	-1.46565	2.61183	3.19537
H	-0.51716	2.46921	2.67257
C	2.37421	2.51937	-0.80661
C	2.60328	2.37046	-2.18918
C	3.92362	2.16342	-2.60770
H	4.13473	2.05261	-3.66682
C	4.97050	2.10445	-1.69142
H	5.98762	1.94531	-2.03930
C	4.71570	2.25333	-0.33159
H	5.53911	2.21147	0.37474
C	3.41554	2.47096	0.14142
C	1.48284	2.48801	-3.21795
H	0.53385	2.40449	-2.68333
C	1.52446	3.86325	-3.91570
H	1.43972	4.68371	-3.19573
H	0.69927	3.95636	-4.63070
H	2.46214	3.99888	-4.46676
C	1.50492	1.34771	-4.24991
H	2.40868	1.36633	-4.86940
H	0.64646	1.44064	-4.92425
H	1.43954	0.37844	-3.75011
C	3.17347	2.70305	1.62996
H	2.09575	2.65093	1.80409
C	3.65502	4.10669	2.05213
H	3.45169	4.27841	3.11503
H	3.15416	4.89607	1.48270
H	4.73409	4.21642	1.89529
C	3.82041	1.61935	2.50780
H	4.91426	1.63879	2.44458
H	3.47976	0.62392	2.21723
H	3.55288	1.78137	3.55799
C	-0.00208	-2.06350	0.00648
C	-0.54964	-4.29431	-0.20249

H	-1.18923	-5.13160	-0.42811
C	0.72570	-4.23551	0.23192
H	1.43532	-5.01172	0.46565
C	-2.33120	-2.69713	-0.77677
C	-3.36518	-2.68301	0.18341
C	-4.67523	-2.48991	-0.27402
H	-5.49238	-2.47641	0.44059
C	-4.94596	-2.32152	-1.62844
H	-5.96948	-2.17653	-1.96334
C	-3.90775	-2.34381	-2.55658
H	-4.13278	-2.21881	-3.61095
C	-2.57937	-2.53263	-2.15590
C	-3.10270	-2.90710	1.67049
H	-2.02496	-2.82948	1.83523
C	-3.77047	-1.84048	2.55698
H	-3.46059	-0.83241	2.27018
H	-3.48792	-1.99741	3.60365
H	-4.86374	-1.88960	2.50452
C	-3.54266	-4.32319	2.09584
H	-4.61987	-4.46212	1.95020
H	-3.32267	-4.49112	3.15582
H	-3.02596	-5.09628	1.51840
C	-1.46564	-2.61184	-3.19537
H	-0.51716	-2.46921	-2.67257
C	-1.55581	-1.49863	-4.25244
H	-2.45527	-1.58533	-4.87243
H	-0.69255	-1.55618	-4.92409
H	-1.54758	-0.51507	-3.77653
C	-1.44537	-4.00309	-3.86235
H	-1.31031	-4.80206	-3.12627
H	-0.62444	-4.06944	-4.58489
H	-2.38136	-4.19749	-4.39858
C	2.37422	-2.51936	0.80661
C	3.41555	-2.47096	-0.14142
C	4.71571	-2.25331	0.33158
H	5.53912	-2.21146	-0.37475
C	4.97051	-2.10444	1.69140
H	5.98763	-1.94530	2.03929
C	3.92364	-2.16341	2.60770
H	4.13475	-2.05260	3.66681
C	2.60330	-2.37045	2.18918
C	3.17347	-2.70304	-1.62996
H	2.09575	-2.65094	-1.80409
C	3.82039	-1.61933	-2.50780
H	3.47972	-0.62391	-2.21724
H	3.55287	-1.78136	-3.55800
H	4.91424	-1.63875	-2.44459
C	3.65503	-4.10668	-2.05213
H	4.73411	-4.21640	-1.89530

H 3.45170 -4.27840 -3.11503
H 3.15419 -4.89606 -1.48270
C 1.48286 -2.48801 3.21796
H 0.53387 -2.40452 2.68334
C 1.50492 -1.34768 4.24989
H 2.40869 -1.36626 4.86937
H 0.64648 -1.44063 4.92424
H 1.43951 -0.37842 3.75007
C 1.52451 -3.86323 3.91573
H 1.43979 -4.68370 3.19578
H 0.69932 -3.95635 4.63073
H 2.46219 -3.99883 4.46679
C -1.44538 4.00308 3.86236
H -1.31032 4.80205 3.12628
H -0.62446 4.06942 4.58490
H -2.38137 4.19748 4.39858
C -1.55582 1.49861 4.25244
H -2.45527 1.58531 4.87243
H -0.69256 1.55617 4.92409
H -1.54758 0.51505 3.77652

(vii) Fully optimized isomers of [15b] at BP86/BS1 level.

[15b]
140
SCF(BS1) = -2694.24722524
H(0 K) = -2692.998590
G(298 K) = -2693.180983
SCF(BS1)+D3(BJ) = -2694.63869152
SCF(PCM=THF) = -2694.26319273
SCF(PCM=C6H5F) = -2694.26125818
SCF(BS2) = -4247.44791876
Lowest Freq. = 12.5358cm-1, 19.1061cm-1, 22.4832cm-1

Ir 0.01096 0.00587 0.05689
H 1.58350 -0.14553 0.47909
H -0.23136 -0.30402 1.66023
H -1.69921 0.10439 0.01051
H 0.67772 0.25079 -1.48957
Zn -1.38063 0.28853 -1.93993
N -0.99498 -2.96754 0.17068
N 0.85459 -2.82698 -0.96180
N -0.77149 3.01098 0.58900
N 1.31059 2.64726 1.08550
C 3.09804 1.77134 2.53671
C -2.41774 0.29688 -3.61198
H -3.35328 0.86693 -3.48046
H -2.68374 -0.73365 -3.90920
H -1.85307 0.74872 -4.44529
C -0.05186 -2.02823 -0.25897
C -2.15793 -2.79074 1.02082
C -3.43678 -2.68043 0.41795
C -3.62620 -2.57363 -1.09482
H -2.65766 -2.26935 -1.53134
C -4.01221 -3.94226 -1.70544
H -3.24330 -4.70856 -1.51196
H -4.13693 -3.85596 -2.79904
H -4.96589 -4.30521 -1.28242
C -4.66597 -1.50036 -1.48019
H -5.68360 -1.78086 -1.15540
H -4.69352 -1.37713 -2.57544
H -4.41867 -0.52306 -1.03441
C -4.56491 -2.72023 1.26189
H -5.56414 -2.64670 0.82110
C -4.43128 -2.86319 2.64761
H -5.32217 -2.90040 3.28380
C -3.15700 -2.95478 3.21860
H -3.05869 -3.06712 4.30315
C -1.99473 -2.92540 2.42340
C -0.62423 -3.10710 3.07451
H 0.13691 -2.78450 2.34386
C -0.37490 -4.59568 3.41626
H -1.12586 -4.96647 4.13662
H 0.62311 -4.72692 3.87011
H -0.42514 -5.23356 2.51790
C -0.44894 -2.22348 4.32579
H -0.61046 -1.16206 4.07827
H 0.57479 -2.33210 4.72242
H -1.14388 -2.50613 5.13639
C -0.67327 -4.26404 -0.24880
H -1.29831 -5.11486 0.00543
C 0.48369 -4.17527 -0.95855
H 1.08434 -4.93227 -1.45401
C 2.12444 -2.44719 -1.54842
C 3.29030 -2.56986 -0.75149
C 3.24164 -3.01753 0.70900
H 2.19080 -2.96769 1.04077
C 3.71772 -4.48375 0.84415
H 4.76710 -4.59047 0.51625
H 3.10486 -5.17052 0.23606
H 3.65719 -4.81504 1.89569
C 4.05246 -2.08966 1.63457
H 5.13203 -2.11550 1.40271
H 3.93680 -2.41023 2.68489
H 3.70614 -1.04836 1.54888
C 4.53281 -2.32678 -1.36890

H	5.44928	-2.41440	-0.77611
C	4.61488	-1.99025	-2.72467
H	5.59152	-1.81190	-3.18761
C	3.44779	-1.88735	-3.49178
H	3.52163	-1.63175	-4.55347
C	2.17826	-2.11387	-2.92477
C	0.92569	-2.05879	-3.79828
H	0.05816	-1.93521	-3.12555
C	0.73922	-3.38422	-4.57543
H	1.59517	-3.56734	-5.24920
H	-0.17651	-3.34641	-5.19117
H	0.65469	-4.24816	-3.89513
C	0.92827	-0.85974	-4.76814
H	1.08345	0.08707	-4.22571
H	-0.03891	-0.79952	-5.29448
H	1.71577	-0.95186	-5.53697
C	0.19004	1.99147	0.56798
C	-2.11019	2.98131	0.04200
C	-2.32881	3.57651	-1.22835
C	-1.21592	4.26599	-2.02410
H	-0.25128	4.01091	-1.55264
C	-1.14443	3.80603	-3.49521
H	-1.00302	2.71643	-3.57017
H	-0.29950	4.30159	-4.00423
H	-2.06178	4.06490	-4.05244
C	-1.38112	5.80399	-1.95567
H	-2.33154	6.11852	-2.42192
H	-0.55783	6.30586	-2.49335
H	-1.38254	6.17218	-0.91619
C	-3.65054	3.59137	-1.72180
H	-3.85082	4.04042	-2.70025
C	-4.70515	3.04998	-0.97972
H	-5.72350	3.06787	-1.38294
C	-4.46387	2.49849	0.28711
H	-5.29972	2.09498	0.86604
C	-3.16714	2.45954	0.83229
C	-2.91417	1.94063	2.24737
H	-1.90250	1.49793	2.25246
C	-2.95030	3.10863	3.26419
H	-2.19784	3.88100	3.03400
H	-2.74820	2.73480	4.28344
H	-3.94375	3.59221	3.27169
C	-3.89181	0.83237	2.67548
H	-4.92355	1.20976	2.79876
H	-3.58140	0.41929	3.64981
H	-3.90442	0.00409	1.95007
C	-0.26237	4.20837	1.10256
H	-0.87821	5.09820	1.19188
C	1.04261	3.98130	1.40984
H	1.80881	4.63122	1.82160
C	2.66591	2.16066	1.24480
C	3.55381	2.26119	0.14427
C	3.09474	2.74969	-1.22886
H	1.99323	2.69454	-1.25004
C	3.60732	1.85603	-2.37546
H	4.70800	1.88503	-2.46457
H	3.19428	2.20697	-3.33749
H	3.29768	0.80992	-2.22736
C	3.51095	4.22437	-1.44510
H	3.09031	4.88409	-0.66713
H	3.15823	4.58710	-2.42672
H	4.61007	4.33266	-1.42023
C	4.90880	1.95193	0.37026
H	5.61776	2.02134	-0.46128
C	5.36647	1.57738	1.63961
H	6.42756	1.35456	1.79594
C	4.46714	1.48668	2.70893
H	4.83156	1.19016	3.69807
C	2.13871	1.68397	3.72354
H	1.11139	1.71081	3.32093
C	2.29151	0.35563	4.49319
H	2.16634	-0.50487	3.81757
H	1.52628	0.28723	5.28545
H	3.27846	0.27352	4.98228
C	2.31877	2.88762	4.67842
H	3.34255	2.91326	5.09254
H	1.61305	2.81986	5.52497
H	2.13973	3.84741	4.16558

[15b] – TS H/H

140
SCF(BS1) = -2694.21734278
H(0 K) = -2692.968214
G(298 K) = -2693.149444
SCF(BS1)+D3(BJ) = -2694.60798137
SCF(PCM=THF) = -2694.23411513
SCF(PCM=C6H5F) = -2694.23222487
SCF(BS2) = -4247.41712664
Lowest Freq. = -568.9625cm-1, 14.6036cm-1, 20.9319cm-1

Ir	0.11689	0.03072	0.08973
H	1.89452	0.14863	0.19440
H	0.00297	-0.09270	1.75503
H	-1.39994	-0.45863	0.02637
H	1.63346	0.96454	0.20545
Zn	0.04743	0.17066	-2.39878
N	-0.17385	-3.08602	0.10081

N	1.91635	-2.54173	-0.17679
N	-1.96137	2.41721	0.07307
N	0.06865	3.14008	0.38322
C	1.82149	3.30430	2.09793
C	-0.04439	0.31567	-4.39559
H	-0.75605	-0.41334	-4.82625
H	0.94213	0.13408	-4.85695
H	-0.37645	1.32481	-4.70211
C	0.64576	-1.95642	-0.01720
C	-1.56775	-3.14794	0.48332
C	-2.56319	-3.14044	-0.52556
C	-2.19379	-3.00472	-2.00220
H	-1.26785	-2.40176	-2.04759
C	-1.89788	-4.39067	-2.62588
H	-1.06906	-4.90506	-2.11218
H	-1.62014	-4.28207	-3.68913
H	-2.78904	-5.04142	-2.57196
C	-3.26447	-2.26892	-2.82949
H	-4.18230	-2.87290	-2.94827
H	-2.87870	-2.06663	-3.84340
H	-3.54268	-1.30886	-2.36570
C	-3.89949	-3.32323	-0.12286
H	-4.69245	-3.31421	-0.87631
C	-4.23007	-3.52456	1.22388
H	-5.27612	-3.67274	1.51352
C	-3.22481	-3.54613	2.19755
H	-3.48907	-3.71878	3.24678
C	-1.87089	-3.36047	1.85157
C	-0.79184	-3.44953	2.93262
H	0.17969	-3.22846	2.46004
C	-0.71637	-4.87953	3.51718
H	-1.66041	-5.15866	4.01776
H	0.09274	-4.94891	4.26513
H	-0.52002	-5.63007	2.73254
C	-0.99420	-2.40456	4.04973
H	-0.96772	-1.38557	3.63108
H	-0.18932	-2.49350	4.80090
H	-1.95531	-2.54579	4.57503
C	0.55340	-4.27955	0.01897
H	0.06717	-5.24660	0.10600
C	1.85831	-3.94048	-0.14987
H	2.75175	-4.54939	-0.24754
C	3.19976	-1.88292	-0.29392
C	3.88293	-1.50638	0.89475
C	3.27807	-1.73402	2.28148
H	2.17941	-1.69272	2.17400
C	3.66675	-3.13237	2.82204
H	4.76354	-3.22299	2.91878
H	3.31449	-3.94130	2.16132
H	3.22384	-3.29467	3.82040
C	3.67525	-0.64626	3.29926
H	4.74449	-0.70411	3.57225
H	3.09793	-0.77976	4.22972
H	3.47086	0.36528	2.91295
C	5.17439	-0.96022	0.76135
H	5.72173	-0.65629	1.65843
C	5.77169	-0.80726	-0.49524
H	6.77923	-0.38470	-0.57481
C	5.08484	-1.19883	-1.64971
H	5.56390	-1.08179	-2.62648
C	3.78763	-1.74561	-1.57974
C	3.08722	-2.21598	-2.85629
H	2.00056	-2.22696	-2.65329
C	3.52323	-3.65323	-3.23521
H	4.61282	-3.69448	-3.41175
H	3.01631	-3.97500	-4.16165
H	3.27880	-4.38399	-2.44828
C	3.33258	-1.27587	-4.05455
H	3.08921	-0.23016	-3.80915
H	2.69966	-1.58082	-4.90458
H	4.38118	-1.31565	-4.39939
C	-0.64357	1.94608	0.17591
C	-3.19241	1.67461	-0.11017
C	-3.81164	1.69113	-1.38732
C	-3.20436	2.40681	-2.59716
H	-2.13209	2.57287	-2.38827
C	-3.30476	1.57813	-3.89478
H	-2.85023	0.58294	-3.77761
H	-2.77580	2.09498	-4.71313
H	-4.35306	1.44604	-4.21601
C	-3.87333	3.78653	-2.81652
H	-4.95328	3.66729	-3.01517
H	-3.42341	4.29828	-3.68528
H	-3.76456	4.44873	-1.94262
C	-5.07175	1.06997	-1.51146
H	-5.57427	1.07079	-2.48395
C	-5.69093	0.46146	-0.41557
H	-6.66861	-0.01815	-0.53499
C	-5.06729	0.47682	0.83823
H	-5.56592	0.00804	1.69075
C	-3.81691	1.09502	1.02746
C	-3.20056	1.19790	2.42457
H	-2.10537	1.10242	2.30590
C	-3.51704	2.57217	3.06730
H	-3.12324	3.41363	2.47515
H	-3.07023	2.63230	4.07557
H	-4.60836	2.70905	3.17389
C	-3.65931	0.07754	3.37669

H -4.72279 0.18626 3.65873
H -3.07401 0.12303 4.31061
H -3.51724 -0.91965 2.93130
C -2.03067 3.81127 0.20059
H -2.97936 4.33644 0.15278
C -0.76390 4.26329 0.39358
H -0.37203 5.26351 0.55292
C 1.46561 3.27129 0.72448
C 2.41701 3.45141 -0.31291
C 1.99971 3.48564 -1.78253
H 1.03044 2.96032 -1.85805
C 2.99188 2.74975 -2.70522
H 3.96143 3.27450 -2.77553
H 2.57871 2.68729 -3.72627
H 3.18147 1.72413 -2.34760
C 1.79246 4.94409 -2.25687
H 1.02486 5.46194 -1.65767
H 1.46964 4.96446 -3.31245
H 2.73111 5.52090 -2.17638
C 3.76116 3.64353 0.06095
H 4.51893 3.78293 -0.71631
C 4.14247 3.66157 1.40892
H 5.19376 3.81269 1.67708
C 3.18117 3.49725 2.41387
H 3.48582 3.52906 3.46563
C 0.78440 3.19995 3.21680
H -0.18793 2.96139 2.75457
C 1.10105 2.06230 4.20943
H 1.12042 1.09210 3.68731
H 0.32374 2.01826 4.99251
H 2.07139 2.21350 4.71500
C 0.64067 4.55510 3.94922
H 1.58516 4.84548 4.44266
H -0.14001 4.49192 4.72710
H 0.36367 5.36570 3.25377

(viii) Fully optimized isomers of [15b] at B3LYP/BS1 level.

[15b]
140
SCF(BS1) = -2694.11575845
H(0 K) = -2692.833220
G(298 K) = -2693.010571
SCF(BS1)+D3(BJ) = -2694.51116901
SCF(PCM=THF) = -2694.13272907
SCF(PCM=C6H5F) = -2694.13063141
SCF(BS2) = -4247.19984832
Lowest Freq. = 11.9347cm-1, 21.2633cm-1, 24.1289cm-1

Ir 0.00199 0.01406 0.03923
H 1.56367 0.02303 0.48274
H -0.26530 -0.27171 1.63110
H -1.71794 -0.04213 -0.14009
H 0.59215 0.30599 -1.54901
Zn -1.34799 0.09575 -2.01537
N -0.69590 -3.04555 0.21320
N 1.15128 -2.75173 -0.86869
N -1.07905 2.93856 0.52876
N 1.01868 2.79918 1.01396
C 2.87359 2.15230 2.48242
C -2.32105 -0.05524 -3.72207
H -3.30982 0.40958 -3.64829
H -2.46493 -1.10850 -3.99169
H -1.77996 0.42362 -4.54477
C 0.15596 -2.04018 -0.21811
C -1.88700 -2.97091 1.03426
C -3.15090 -3.01200 0.40997
C -3.32479 -2.97590 -1.10519
H -2.39065 -2.60737 -1.53923
C -3.57770 -4.38897 -1.67139
H -2.75591 -5.07304 -1.43984
H -3.68569 -4.35069 -2.76095
H -4.49735 -4.81724 -1.25697
C -4.44619 -2.01682 -1.54459
H -5.43352 -2.37977 -1.23889
H -4.45536 -1.92642 -2.63473
H -4.30506 -1.01769 -1.12370
C -4.28058 -3.14026 1.22919
H -5.26485 -3.18233 0.77414
C -4.16340 -3.22854 2.61221
H -5.05208 -3.33642 3.22801
C -2.90615 -3.17718 3.20528
H -2.82340 -3.24989 4.28492
C -1.74302 -3.05160 2.43570
C -0.38043 -3.07403 3.12117
H 0.35585 -2.69388 2.40949
C 0.01755 -4.51456 3.50540
H -0.69602 -4.94284 4.21858
H 1.00814 -4.52658 3.97369
H 0.05218 -5.17157 2.63101
C -0.32527 -2.15151 4.35049
H -0.59884 -1.12844 4.08006
H 0.69074 -2.13544 4.75784
H -0.98993 -2.49055 5.15272
C -0.23721 -4.30814 -0.16173

H	-0.77635	-5.20437	0.09623
C	0.91436	-4.12414	-0.83791
H	1.59260	-4.82602	-1.29385
C	2.39539	-2.26973	-1.42817
C	3.53364	-2.24855	-0.59602
C	3.48423	-2.66114	0.87210
H	2.43600	-2.68174	1.17895
C	4.06107	-4.08027	1.05871
H	5.11620	-4.11734	0.76496
H	3.52197	-4.82026	0.45902
H	3.99437	-4.38583	2.10887
C	4.19863	-1.65985	1.79343
H	5.27753	-1.62491	1.60531
H	4.06259	-1.95454	2.84003
H	3.79568	-0.65393	1.66486
C	4.75865	-1.89735	-1.17627
H	5.65099	-1.87148	-0.55882
C	4.85407	-1.59658	-2.53107
H	5.81523	-1.33205	-2.96342
C	3.71934	-1.64473	-3.33541
H	3.80738	-1.42194	-4.39374
C	2.46758	-1.98441	-2.80634
C	1.25854	-2.09783	-3.72928
H	0.36086	-2.05857	-3.10574
C	1.25648	-3.45256	-4.46875
H	2.14696	-3.55444	-5.09934
H	0.37495	-3.53573	-5.11407
H	1.24253	-4.29570	-3.77140
C	1.16547	-0.93902	-4.73708
H	1.20105	0.02869	-4.22970
H	0.22266	-0.99994	-5.28900
H	1.97663	-0.97051	-5.47263
C	-0.02411	2.03509	0.51756
C	-2.41042	2.76910	-0.00311
C	-2.69206	3.29939	-1.28115
C	-1.65939	4.07163	-2.10386
H	-0.67460	3.89782	-1.66177
C	-1.58227	3.62193	-3.57364
H	-1.37032	2.55412	-3.65899
H	-0.78588	4.17095	-4.08772
H	-2.51458	3.82124	-4.11270
C	-1.94176	5.58818	-2.03447
H	-2.91535	5.82451	-2.47800
H	-1.17682	6.14571	-2.58594
H	-1.94905	5.95638	-1.00447
C	-4.00391	3.17315	-1.76097
H	-4.25236	3.56649	-2.74177
C	-4.99263	2.56306	-0.99681
H	-6.00231	2.47312	-1.38810
C	-4.69552	2.08364	0.27778
H	-5.48186	1.62986	0.87121
C	-3.40435	2.18247	0.80779
C	-3.10501	1.73562	2.23527
H	-2.05130	1.44752	2.27132
C	-3.32755	2.90171	3.22283
H	-2.69896	3.76521	2.98590
H	-3.08958	2.58556	4.24468
H	-4.37270	3.23224	3.20834
C	-3.91560	0.50724	2.67169
H	-4.98381	0.73087	2.77739
H	-3.56258	0.16111	3.64826
H	-3.80042	-0.31661	1.96481
C	-0.69657	4.18102	1.02984
H	-1.39473	4.99643	1.11733
C	0.61474	4.09521	1.32819
H	1.30321	4.82003	1.72952
C	2.41272	2.45894	1.18721
C	3.28791	2.61593	0.09312
C	2.79420	3.02334	-1.29144
H	1.71790	2.84029	-1.32609
C	3.41883	2.18300	-2.41718
H	4.49756	2.35235	-2.50983
H	2.96644	2.45555	-3.37734
H	3.24967	1.11728	-2.25001
C	3.03236	4.52886	-1.53172
H	2.53294	5.14265	-0.77518
H	2.64880	4.82652	-2.51422
H	4.10170	4.76761	-1.50318
C	4.65726	2.44501	0.32826
H	5.35608	2.55980	-0.49431
C	5.14023	2.15012	1.60031
H	6.20842	2.03434	1.76265
C	4.25490	2.00532	2.66404
H	4.64024	1.77588	3.65292
C	1.93074	2.00742	3.67310
H	0.90866	1.97866	3.28641
C	2.15905	0.68857	4.43222
H	2.06471	-0.16670	3.75940
H	1.41519	0.58088	5.22885
H	3.14870	0.65224	4.90108
C	2.04660	3.21170	4.62950
H	3.05715	3.28930	5.04631
H	1.34671	3.10626	5.46587
H	1.82306	4.15548	4.12230

[15b] (η^2-H_2)H_2 isomer

140

SCF(BS1) = -2694.09539699

H(0 K) = -2692.812334
G(298 K) = -2692.990615
SCF(BS1)+D3(BJ) = -2694.49038430
SCF(PCM=THF) = -2694.11206923
SCF(PCM=C6H5F) = -2694.11020155
SCF(BS2) = -4247.17755811
Lowest Freq. = 9.9648cm-1, 21.3227cm-1, 24.5748cm-1

Ir	0.04818	0.03338	0.04836
H	1.59884	0.58036	0.55902
H	-0.24454	-0.10020	1.67404
H	-1.46429	-0.49642	-0.06275
H	1.61388	0.58637	-0.39226
Zn	-0.11427	-0.12429	-2.43183
N	-0.03521	-3.08790	0.22046
N	1.99407	-2.43686	-0.15475
N	-2.00250	2.43410	0.00875
N	0.01825	3.14024	0.31805
C	1.75921	3.33043	2.02757
C	-0.24132	-0.44193	-4.40700
H	-1.27668	-0.48679	-4.76066
H	0.23886	-1.39191	-4.67386
H	0.26744	0.35176	-4.96969
C	0.70924	-1.93245	0.03463
C	-1.40868	-3.21304	0.65058
C	-2.42720	-3.33853	-0.31468
C	-2.13106	-3.29473	-1.80965
H	-1.17687	-2.77639	-1.93952
C	-1.97230	-4.72045	-2.37881
H	-1.16907	-5.27009	-1.87833
H	-1.73703	-4.68166	-3.44819
H	-2.89772	-5.29504	-2.25738
C	-3.18664	-2.51209	-2.60826
H	-4.14961	-3.03431	-2.63650
H	-2.85297	-2.39077	-3.64389
H	-3.35143	-1.51974	-2.18046
C	-3.73046	-3.57051	0.14108
H	-4.53679	-3.66864	-0.57824
C	-4.00883	-3.68830	1.49996
H	-5.02689	-3.87379	1.83154
C	-2.98174	-3.58101	2.43261
H	-3.20480	-3.69137	3.48979
C	-1.66023	-3.34481	2.03182
C	-0.55136	-3.30593	3.08093
H	0.37472	-3.01324	2.58152
C	-0.32857	-4.70393	3.69377
H	-1.22147	-5.05228	4.22472
H	0.49887	-4.68024	4.41159
H	-0.08767	-5.44668	2.92643
C	-0.81153	-2.26092	4.17999
H	-0.90841	-1.26339	3.74522
H	0.02441	-2.24842	4.88872
H	-1.72051	-2.48268	4.74996
C	0.75121	-4.23575	0.13289
H	0.33002	-5.21974	0.25560
C	2.01538	-3.83077	-0.09727
H	2.92871	-4.38796	-0.22065
C	3.23664	-1.71391	-0.31161
C	3.91940	-1.28303	0.84872
C	3.37642	-1.53774	2.25326
H	2.28605	-1.57487	2.18437
C	3.87542	-2.89578	2.79365
H	4.96930	-2.90751	2.86217
H	3.56813	-3.72867	2.15563
H	3.47404	-3.07731	3.79704
C	3.72565	-0.42075	3.25069
H	4.79257	-0.41259	3.50151
H	3.17844	-0.57792	4.18579
H	3.45767	0.56465	2.86207
C	5.16265	-0.66188	0.68091
H	5.70678	-0.31524	1.55255
C	5.71941	-0.49003	-0.58280
H	6.68786	-0.00889	-0.68927
C	5.04235	-0.94666	-1.70775
H	5.49462	-0.82486	-2.68628
C	3.79127	-1.57066	-1.60162
C	3.12219	-2.12641	-2.85700
H	2.06032	-2.27221	-2.63574
C	3.71969	-3.49898	-3.23656
H	4.78908	-3.40867	-3.45819
H	3.22312	-3.89700	-4.12819
H	3.60580	-4.23372	-2.43502
C	3.21738	-1.17266	-4.06098
H	2.82661	-0.18064	-3.82255
H	2.63313	-1.56825	-4.89654
H	4.24919	-1.05944	-4.41061
C	-0.69720	1.96574	0.10948
C	-3.23416	1.69614	-0.16838
C	-3.83048	1.66625	-1.44713
C	-3.19974	2.33038	-2.67031
H	-2.12700	2.43435	-2.47698
C	-3.36416	1.49778	-3.95458
H	-3.05517	0.46165	-3.80750
H	-2.75024	1.92159	-4.75556
H	-4.40142	1.49832	-4.30729
C	-3.78155	3.74161	-2.90606
H	-4.86288	3.68866	-3.07564

```
H  -3.32444  4.19803 -3.79107
H  -3.60738  4.41042 -2.05990
C  -5.07986  1.04223 -1.56906
H  -5.56232  1.00176 -2.53999
C  -5.71507  0.48000 -0.46757
H  -6.68254 -0.00067 -0.58506
C  -5.11810  0.54499  0.78790
H  -5.63043  0.11595  1.64180
C  -3.87565  1.16298  0.97121
C  -3.29018  1.31341  2.37320
H  -2.20163  1.33177  2.27304
C  -3.74890  2.63987  3.01845
H  -3.44362  3.51132  2.43331
H  -3.31830  2.74361  4.02097
H  -4.84027  2.66543  3.11849
C  -3.63440  0.14123  3.30527
H  -4.69630  0.13020  3.57780
H  -3.06740  0.23469  4.23726
H  -3.38518 -0.81954  2.85014
C  -2.06681  3.82101  0.14930
H  -3.00457  4.34844  0.10969
C  -0.80838  4.26193  0.33859
H  -0.41627  5.25237  0.50040
C   1.41185  3.27954  0.66160
C   2.36111  3.45851 -0.36532
C   1.95457  3.49134 -1.83572
H   1.01107  2.94530 -1.92485
C   2.96942  2.79567 -2.75819
H   3.91046  3.35250 -2.83015
H   2.56065  2.72232 -3.77156
H   3.19848  1.78550 -2.40821
C   1.71094  4.94203 -2.30269
H   0.93028  5.43165 -1.71282
H   1.39856  4.95924 -3.35276
H   2.62497  5.54009 -2.21279
C   3.69451  3.66068  0.01084
H   4.45026  3.79866 -0.75531
C   4.06765  3.69359  1.35226
H   5.10815  3.85335  1.62159
C   3.10796  3.53578  2.34765
H   3.40574  3.58226  3.39108
C   0.72237  3.23438  3.14466
H  -0.24011  2.98777  2.69127
C   1.03676  2.11506  4.15234
H   1.07279  1.14550  3.65027
H   0.25545  2.07446  4.91965
H   1.99106  2.28009  4.66471
C   0.56580  4.59237  3.85943
H   1.49644  4.89232  4.35389
H  -0.21537  4.53333  4.62517
H   0.29135  5.38727  3.15833
```

[15b] – TS H/H

140
SCF(BS1) = -2694.08432907
H(0 K) = -2692.800945
G(298 K) = -2692.977927
SCF(BS1)+D3(BJ) = -2694.47884701
SCF(PCM=THF) = -2694.10166817
SCF(PCM=C6H5F) = -2694.09974570
SCF(BS2) = -4247.16622763
Lowest Freq. = -597.0238cm-1, 16.9932cm-1, 19.4752cm-1

```
Ir  0.10537  0.03278  0.07332
H   1.90309  0.24008  0.17159
H  -0.00173 -0.07840  1.73402
H  -1.38035 -0.50437  0.00643
H   1.62787  1.00985  0.18211
Zn  0.02374  0.14237 -2.42934
N  -0.05366 -3.09132  0.11605
N   2.00244 -2.47687 -0.16734
N  -2.03684  2.36030  0.05570
N  -0.04264  3.14244  0.36302
C   1.69871  3.36352  2.07058
C  -0.08686  0.25146 -4.43657
H  -0.79170 -0.48394 -4.84645
H   0.88813  0.07087 -4.90316
H  -0.42679  1.24443 -4.75830
C   0.72074 -1.94554 -0.01023
C  -1.43934 -3.20609  0.50527
C  -2.43319 -3.27219 -0.49159
C  -2.08622 -3.17192 -1.97341
H  -1.16064 -2.59359 -2.05096
C  -1.82600 -4.57029 -2.57325
H  -1.00728 -5.08714 -2.06416
H  -1.56172 -4.48772 -3.63344
H  -2.72047 -5.19923 -2.49675
C  -3.15470 -2.43417 -2.79554
H  -4.07656 -3.01947 -2.89021
H  -2.78277 -2.25402 -3.80955
H  -3.40848 -1.47127 -2.34568
C  -3.75184 -3.49831 -0.08000
H  -4.54132 -3.54819 -0.82219
C  -4.06745 -3.67054  1.26536
H  -5.09715 -3.85095  1.56202
C  -3.06394 -3.62546  2.22808
H  -3.31649 -3.78028  3.27311
```

C	-1.72809	-3.39665	1.87217
C	-0.64493	-3.42942	2.94845
H	0.30378	-3.15951	2.47991
C	-0.49027	-4.85131	3.52623
H	-1.40607	-5.17847	4.03094
H	0.32299	-4.87980	4.25985
H	-0.26371	-5.58240	2.74337
C	-0.89074	-2.40506	4.06976
H	-0.92427	-1.39220	3.66171
H	-0.07827	-2.45369	4.80392
H	-1.82737	-2.59890	4.60401
C	0.71274	-4.25326	0.02987
H	0.26700	-5.23023	0.11558
C	1.99253	-3.87175	-0.14191
H	2.89792	-4.44605	-0.24238
C	3.26268	-1.77963	-0.27770
C	3.92513	-1.38287	0.90708
C	3.33477	-1.63936	2.29202
H	2.24639	-1.65757	2.18986
C	3.79459	-3.00956	2.83576
H	4.88534	-3.04060	2.93948
H	3.49429	-3.83129	2.17992
H	3.35772	-3.19317	3.82376
C	3.66982	-0.53384	3.30728
H	4.72916	-0.53970	3.58835
H	3.09402	-0.69073	4.22499
H	3.42370	0.45776	2.91919
C	5.18657	-0.78818	0.78077
H	5.71620	-0.46834	1.67147
C	5.77851	-0.60917	-0.46571
H	6.76019	-0.14926	-0.53983
C	5.11895	-1.02876	-1.61587
H	5.59715	-0.89716	-2.58065
C	3.85225	-1.62496	-1.55190
C	3.19245	-2.13425	-2.83142
H	2.11890	-2.22571	-2.63847
C	3.72968	-3.53060	-3.21480
H	4.80935	-3.49289	-3.39878
H	3.24418	-3.88580	-4.13028
H	3.54870	-4.27225	-2.43281
C	3.37101	-1.17468	-4.02104
H	3.05381	-0.15917	-3.77338
H	2.76768	-1.51722	-4.86707
H	4.41186	-1.13571	-4.36047
C	-0.71442	1.93879	0.15720
C	-3.24442	1.58200	-0.11441
C	-3.86027	1.55232	-1.38421
C	-3.28469	2.26862	-2.60553
H	-2.22471	2.46256	-2.41338

C	-3.37579	1.43160	-3.89377
H	-2.91311	0.45072	-3.77376
H	-2.85732	1.94609	-4.70850
H	-4.41409	1.28604	-4.21105
C	-3.98590	3.62632	-2.83159
H	-5.05540	3.48184	-3.02157
H	-3.55617	4.13641	-3.70073
H	-3.88815	4.29372	-1.97158
C	-5.09038	0.88863	-1.49647
H	-5.58809	0.85046	-2.45989
C	-5.68813	0.28740	-0.39533
H	-6.64055	-0.22410	-0.50550
C	-5.07489	0.35497	0.85238
H	-5.56020	-0.10370	1.70626
C	-3.85276	1.01399	1.02795
C	-3.25465	1.17112	2.42474
H	-2.16601	1.16213	2.31725
C	-3.67987	2.51470	3.05848
H	-3.35747	3.37481	2.46675
H	-3.24330	2.61601	4.05870
H	-4.77004	2.56471	3.16302
C	-3.62317	0.02427	3.37863
H	-4.68120	0.05309	3.66498
H	-3.04071	0.11341	4.30122
H	-3.41229	-0.95137	2.93633
C	-2.14902	3.74608	0.18199
H	-3.10475	4.23939	0.13486
C	-0.90848	4.23389	0.37160
H	-0.55300	5.23930	0.52545
C	1.34513	3.32701	0.70435
C	2.28437	3.55571	-0.32355
C	1.87469	3.59923	-1.79241
H	0.92297	3.06777	-1.88268
C	2.87876	2.89055	-2.71757
H	3.83107	3.42873	-2.78142
H	2.47089	2.83001	-3.73177
H	3.08544	1.87347	-2.37288
C	1.64937	5.05459	-2.25315
H	0.87907	5.55290	-1.65657
H	1.33109	5.07910	-3.30112
H	2.57175	5.64015	-2.16663
C	3.61312	3.79160	0.05065
H	4.35974	3.96902	-0.71651
C	3.99173	3.80791	1.39052
H	5.02836	3.99320	1.65841
C	3.04234	3.60145	2.38749
H	3.34460	3.63668	3.42995
C	0.66870	3.22232	3.18858
H	-0.28046	2.92773	2.73595

C	1.03539	2.12499	4.20227
H	1.11461	1.15531	3.70531
H	0.25654	2.05276	4.96970
H	1.98056	2.33650	4.71462
C	0.44990	4.57628	3.89514
H	1.36678	4.92196	4.38557
H	-0.32640	4.48544	4.66266
H	0.13715	5.35303	3.18980

S3 References

(1) Doddrell, D.; Barfield, M.; Adcock, W.; Aurangzeb, M.; Jordan, D. ^{13}C nuclear magnetic resonance studies of some fluorinated and trifluoromethylated aromatic compounds. Studies on ^{13}C-^{19}F coupling constants. *J. Chem. Soc., Perkin Trans. 2* **1976**, 402-412.

(2) Tang, C. Y.; Thompson, A. L.; Aldridge, S. Dehydrogenation of saturated CC and BN bonds at cationic N-heterocyclic carbene stabilized M(III) centers (M = Rh, Ir). *J. Am. Chem. Soc.* **2010**, *132*, 10578-10591.

(3) Walsh, A. M.; Sotorrios, L.; Cameron, R. G.; Pécharman, A.-F.; Procacci, B.; Lowe, J. P.; Macgregor, S. A.; Mahon, M. F.; Hunt, N. T.; Whittlesey, M. K. Isolobal cationic iridium dihydride and dizinc complexes: A dual role for the ZnR ligand enhances H_2 activation. *Inorg. Chem.* **2024**, *63*, 22944-22954.

(4) Bantreil, X.; Nolan, S. P. Synthesis of N-heterocyclic carbene ligands and derived ruthenium olefin metathesis catalysts. *Nat. Protoc.* **2011**, *6*, 69-77.

(5) Ansell, M. B.; Roberts, D. E.; Cloke, F. G. N.; Navarro, O.; Spencer, J. Synthesis of an $(NHC)_2Pd(SiMe_3)_2$ complex and catalytic cis-bis(silyl)ations of alkynes with unactivated disilanes. *Angew. Chem. Int. Ed.* **2015**, *54*, 5577-5582.

(6) https://pubchem.ncbi.nlm.nih.gov/compound/10479.

(7) Attempts to access only the second hydride species by condensing $CdMe_2$ into a frozen fluorobenzene solution of **7** and introducing the sample into an NMR probe pre-cooled to 248 K still showed that **8** was present (1:2.10 ratio with the second hydride).

(8) Specklin, D.; Fliedel, C.; Gourlaouen, C.; Bruyere, J. C.; Avilés, T.; Boudon, C.; Ruhlmann, L.; Dagorne, S. N-Heterocyclic carbene based tri-organyl-Zn-alkyl cations: Synthesis, structures, and use in CO_2 functionalization. *Chem. Eur. J.* **2017**, *23*, 5509-5519.

(9) Arduengo, A. J., III; Dias, H. V. R.; Davidson, F.; Harlow, R. L. Carbene adducts of magnesium and zinc. *J. Organomet. Chem.* **1993**, *462*, 13-18.

(10) Sheldrick, G. M. SHELXT - Integrated space-group and crystal structure determination. *Acta Crystallogr., Sect. A: Found. Adv.* **2015**, *A71*, 3-8.

(11) Sheldrick, G. M. Crystal structure refinement with SHELXL. *Acta Crystallogr., Sect. C: Struct. Chem.* **2015**, *C71*, 3-8.

(12) Dolomanov, O. V.; Bourhis, L. J.; Gildea, R. J.; Howard, J. A. K.; Puschmann, H. OLEX2: a complete structure solution, refinement and analysis program. *J. Appl. Crystallogr.* **2009**, *42*, 339-341.

(13) Frisch, M. J.; Trucks, G. W.; Schlegel, H. B.; Scuseria, G. E.; Robb, M. A.; Cheeseman, J. R.; Scalmani, G.; V. Barone; Petersson, G. A.; Nakatsuji, H.; Li, X.; Caricato, M.; Marenich, A. V.; Bloino, J.; Janesko, B. G.; Gomperts, R.; Mennucci, B.; Hratchian, H. P.; Ortiz, J. V.; Izmaylov, A. F.; Sonnenberg, J. L.; Williams-Young, D.; Ding, F.; Lipparini, F.; Egidi, F.; Goings, J.; Peng, B.; Petrone, A.; Henderson, T.; Ranasinghe, D.; Zakrzewski, V. G.; Gao, J.; Rega, N.; Zheng, G.; Liang, W.; Hada, M.; Ehara, M.; Toyota, K.; Fukuda, R.; Hasegawa, J.; Ishida, M.; Nakajima, T.; Honda, Y.; Kitao, O.; Nakai, H.; Vreven, T.; Throssell, K.; J. A. Montgomery, J.; Peralta, J. E.; Ogliaro, F.; Bearpark, M. J.; Heyd, J. J.; Brothers, E. N.; Kudin, K. N.; Staroverov, V. N.; Keith, T. A.; Kobayashi, R.; Normand, J.; Raghavachari, K.; Rendell, A. P.; Burant, J. C.; Iyengar, S. S.; Tomasi, J.; Cossi, M.; Millam, J. M.; Klene, M.; Adamo, C.; Cammi, R.; Ochterski, J. W.; Martin, R. L.; Morokuma, K.; Farkas, O.; Foresman, J. B.; Fox, D. J. *Gaussian 16,* rev. C.01; Gaussian, Inc.: Wallingford, CT, 2019.

(14) Andrae, D.; Häußermann, U.; Dolg, M.; Stoll, H.; Preuß, H. Energy-adjusted ab initio pseudopotentials for the second and third row transition elements. *Theor. Chim. Acta* **1990**, *77*, 123-141.

(15) Hariharan, P. C.; Pople, J. A. The influence of polarization functions on molecular orbital hydrogenation energies. *Theor. Chim. Acta* **1973**, *28*, 213-222.

(16) Hehre, W. J.; Ditchfield, R.; Pople, J. A. Self-consistent molecular orbital methods. XII. Further extensions of Gaussian-type basis sets for use in molecular orbital studies of organic molecules. *J. Chem. Phys.* **1972**, *56*, 2257-2261.

(17) Höllwarth, A.; Böhme, M.; Dapprich, S.; Ehlers, A. W.; Gobbi, A.; Jonas, V.; Köhler, K. F.; Stegmann, R.; Veldkamp, A.; Frenking, G. A set of d-polarization functions for pseudo-potential basis sets of the main group elements Al-Bi and f-type polarization functions for Zn, Cd, Hg. *Chem. Phys. Lett.* **1993**, *208*, 237-240.

(18) Becke, A. D. Density-functional exchange-energy approximation with correct asymptotic behavior. *Phys. Rev. A* **1988**, *38*, 3098-3100.

(19) Perdew, J. P. Density-functional approximation for the correlation energy of the inhomogeneous electron gas. *Phys. Rev. B* **1986**, *33*, 8822-8824.

(20) Weigend, F.; Ahlrichs, R. Balanced basis sets of split valence, triple zeta valence and quadruple zeta valence quality for H to Rn: Design and assessment of accuracy. *Phys. Chem. Chem. Phys.* **2005**, *7*, 3297-3305.

(21) Weigend, F. Accurate Coulomb-fitting basis sets for H to Rn. *Phys. Chem. Chem. Phys.* **2006**, *8*, 1057-1065.

(22) Grimme, S.; Antony, J.; Ehrlich, S.; Krieg, H. A consistent and accurate ab initio parametrization of density functional dispersion correction (DFT-D) for the 94 elements H-Pu. *J. Chem. Phys.* **2010**, *132*, 154104.

(23) Grimme, S.; Ehrlich, S.; Goerigk, L. Effect of the damping function in dispersion corrected density functional theory. *J. Comput. Chem.* **2011**, *32*, 1456-1465.

(24) Johnson, E. R.; Becke, A. D. A post-Hartree–Fock model of intermolecular interactions. *J. Chem. Phys.* **2005**, *123*, 024101.

(25) Tomasi, J.; Mennucci, B.; Cammi, R. Quantum mechanical continuum solvation models. *Chem. Rev.* **2005**, *105*, 2999-3094.

(26) Bader, R. F. W. *Atoms in Molecules: A Quantum Theory*; Clarendon Press, 1994.

(27) Keith, T. A. *AIMAll,* ver. 19.10.12; TK Gristmill Software: Overland Park, KS, 2019.

(28) Perdew, J. P.; Burke, K.; Ernzerhof, M. Generalized gradient approximation made simple. *Phys. Rev. Lett.* **1996**, *77*, 3865-3868.

(29) Perdew, J. P.; Burke, K.; Ernzerhof, M. Generalized gradient approximation made simple [Phys. Rev. Lett. 77, 3865 (1996)]. *Phys. Rev. Lett.* **1997**, *78*, 1396.

(30) Lee, C.; Yang, W.; Parr, R. G. Development of the Colle-Salvetti correlation-energy formula into a functional of the electron density. *Phys. Rev. B* **1988**, *37*, 785-789.

(31) Tao, J.; Perdew, J. P.; Staroverov, V. N.; Scuseria, G. E. Climbing the density functional ladder: Nonempirical meta-generalized gradient approximation designed for molecules and solids. *Phys. Rev. Lett.* **2003**, *91*, 146401.

(32) Becke, A. D. Density-functional thermochemistry. III. The role of exact exchange. *J. Chem. Phys.* **1993**, *98*, 5648-5652.

(33) Stephens, P. J.; Devlin, F. J.; Chabalowski, C. F.; Frisch, M. J. Ab-initio calculation of vibrational absorption and circular dichroism spectra using density functional force fields. *J. Phys. Chem.* **1994**, *98*, 11623-11627.

(34) Perdew, J. P.; Chevary, J. A.; Vosko, S. H.; Jackson, K. A.; Pederson, M. R.; Singh, D. J.; Fiolhais, C. Atoms, molecules, solids, and surfaces: Applications of the generalized gradient approximation for exchange and correlation. *Phys. Rev. B* **1992**, *46*, 6671-6687.

(35) Perdew, J. P.; Chevary, J. A.; Vosko, S. H.; Jackson, K. A.; Pederson, M. R.; Singh, D. J.; Fiolhais, C. Erratum: Atoms, molecules, solids, and surfaces: Applications of the generalized gradient approximation for exchange and correlation. *Phys. Rev. B* **1993**, *48*, 4978-4978.

(36) Adamo, C.; Barone, V. Toward reliable density functional methods without adjustable parameters: The PBE0 model. *J. Chem. Phys.* **1999**, *110*, 6158-6170.

(37) Zhao, Y.; Truhlar, D. G. A new local density functional for main-group thermochemistry, transition metal bonding, thermochemical kinetics, and noncovalent interactions. *J. Chem. Phys.* **2006**, *125*, 194101.

(38) Zhao, Y.; Truhlar, D. G. The M06 suite of density functionals for main group thermochemistry, thermochemical kinetics, noncovalent interactions, excited states, and transition elements: two new functionals and systematic testing of four M06-class functionals and 12 other functionals. *Theor. Chem. Acc.* **2008**, *120*, 215-241.

(39) Yu, H. S.; He, X.; Li, S. L.; Truhlar, D. G. MN15: A Kohn–Sham global-hybrid exchange–correlation density functional with broad accuracy for multi-reference and single-reference systems and noncovalent interactions. *Chem. Sci.* **2016**, *7*, 5032-5051.

(40) Zhao, Y.; Truhlar, D. G. Design of density functionals that are broadly accurate for thermochemistry, thermochemical kinetics, and nonbonded interactions. *J. Phys. Chem. A* **2005**, *109*, 5656-5667.

(41) Grimme, S. Semiempirical GGA-type density functional constructed with a long-range dispersion correction. *J. Comput. Chem.* **2006**, *27*, 1787-1799.

(42) Chai, J.-D.; Head-Gordon, M. Long-range corrected hybrid density functionals with damped atom–atom dispersion corrections. *Phys. Chem. Chem. Phys.* **2008**, *10*, 6615-6620.

(43) Marenich, A. V.; Cramer, C. J.; Truhlar, D. G. Universal solvation model based on solute electron density and a continuum model of the solvent defined by the bulk dielectric constant and atomic surface tensions. *J. Phys. Chem. B* **2009**, *113*, 6378-6396.
